# Evaluation of 309 Environmental Chemicals Using a Mouse Embryonic Stem Cell Adherent Cell Differentiation and Cytotoxicity Assay

**DOI:** 10.1371/journal.pone.0018540

**Published:** 2011-06-07

**Authors:** Kelly J. Chandler, Marianne Barrier, Susan Jeffay, Harriette P. Nichols, Nicole C. Kleinstreuer, Amar V. Singh, David M. Reif, Nisha S. Sipes, Richard S. Judson, David J. Dix, Robert Kavlock, Edward S. Hunter, Thomas B. Knudsen

**Affiliations:** 1 National Health and Environmental Effects Research Laboratory, Office of Research and Development, U.S. Environmental Protection Agency, Research Triangle Park, North Carolina, United States of America; 2 National Center for Computational Toxicology, Office of Research and Development, U.S. Environmental Protection Agency, Research Triangle Park, North Carolina, United States of America; 3 Lockheed Martin, Research Triangle Park, North Carolina, United States of America; Baylor College of Medicine, United States of America

## Abstract

The vast landscape of environmental chemicals has motivated the need for alternative methods to traditional whole-animal bioassays in toxicity testing. Embryonic stem (ES) cells provide an *in vitro* model of embryonic development and an alternative method for assessing developmental toxicity. Here, we evaluated 309 environmental chemicals, mostly food-use pesticides, from the ToxCast™ chemical library using a mouse ES cell platform. ES cells were cultured in the absence of pluripotency factors to promote spontaneous differentiation and in the presence of DMSO-solubilized chemicals at different concentrations to test the effects of exposure on differentiation and cytotoxicity. Cardiomyocyte differentiation (α,β myosin heavy chain; MYH6/MYH7) and cytotoxicity (DRAQ5™/Sapphire700™) were measured by In-Cell Western™ analysis. Half-maximal activity concentration (AC_50_) values for differentiation and cytotoxicity endpoints were determined, with 18% of the chemical library showing significant activity on either endpoint. Mining these effects against the ToxCast Phase I assays (∼500) revealed significant associations for a subset of chemicals (26) that perturbed transcription-based activities and impaired ES cell differentiation. Increased transcriptional activity of several critical developmental genes including *BMPR2*, *PAX6* and *OCT1* were strongly associated with decreased ES cell differentiation. Multiple genes involved in reactive oxygen species signaling pathways (*NRF2*, *ABCG2*, *GSTA2*, *HIF1A*) were strongly associated with decreased ES cell differentiation as well. A multivariate model built from these data revealed alterations in *ABCG2* transporter was a strong predictor of impaired ES cell differentiation. Taken together, these results provide an initial characterization of metabolic and regulatory pathways by which some environmental chemicals may act to disrupt ES cell growth and differentiation.

## Introduction

Over 82,000 chemicals are currently in commerce or in the environment, yet little is known about their potential toxicity to humans [1]. The data gaps between the environmental chemical landscape and chemical toxicity information is largely due to the low-throughput nature of traditional toxicity testing that relies on whole-animal studies and relatively high-dose exposure. These tests can be slow, costly, and ultimately provide only a gross estimation of the human response to chemicals. In an effort to bridge the gap between chemical space and toxicity information, the National Research Council released a report calling for a paradigm shift in toxicity testing [1]. Four primary objectives are outlined in “Toxicity Testing in the 21^st^ Century: A Vision and a Strategy”: 1) broad assessment of chemicals, 2) faster, more cost-effective approaches, 3) reduce animal use, and 4) reflect mechanism and dose in risk assessment [1]. Towards this end, *in vitro* and *in silico* methods are being implemented and evaluated with respect to their predictivity and relevance to *in vivo* toxicity pathways.

*In vitro* assays provide multiple benefits for pathways-based risk assessment. First, *in vitro* assays are amenable to high-throughput formats and thus can be scaled to the evaluation of thousands of chemicals across diverse cellular responses in a relatively short amount of time. Second, chemicals can be tested across a wide range of concentrations allowing the half-maximal activity concentration (AC_50_) to be calculated and considered in the context of environmentally realistic exposures. Third, human *in vitro* model systems provide a glimpse into the potential human cellular responses to chemical insult and may illuminate species-specific differences in toxicity. Finally, *in vitro* approaches allow us to interrogate the mechanism of chemical toxicity and assess pathway-based chemical perturbation by employing biochemical, molecular, and genetic techniques.

The US Environmental Protection Agency (EPA), in collaboration with the National Institutes of Environmental Health Sciences (NIEHS), the National Chemical Genomics Center (NCGC) and the Food and Drug Administration (FDA) are working together to establish alternative chemical testing methods that will characterize toxicity pathways [2]. Many of the EPA's ToxCast™ chemical library of 309 unique compounds, mostly well-characterized food-use pesticides, have been previously tested under the traditional *in vivo* toxicology testing paradigm. Therefore, these data-rich pesticides have been subjected to a variety of whole-animal tests including sub-chronic and chronic rodent bioassays, developmental toxicity, and multi-generation reproductive studies representing over 30 years of *in vivo* toxicity experiments assembled into a searchable database, ToxRefDB [3], [4], [5]. In Phase I of ToxCast, all 309 chemicals were tested over a wide-range of concentrations across a collection of ∼500 bioassays [6], [7], [8]. These include cell-free biochemical, cell-based activity, and embryonic stem cell screening platforms (described here). The power of ToxRefDB and ToxCast is the ability to develop predictive signatures of toxicity via *in silico* modeling, with the ultimate goal of using predictive signatures to interrogate the vast chemical space which currently lacks toxicity profiles. Chemicals that perturb toxicity pathways could then be prioritized for more in-depth testing.

Embryonic stem (ES) cells are potentially informative in the context of toxicity testing due to their reliance on many key pathways in morphogenesis and differentiation. ES cells are isolated from the inner cell mass of blastula-stage embryos and retain the ability to differentiate into all three germ layers of the embryo proper when cultured in the absence of pluripotent factors. Components of the three primary germ layers (ectoderm, endoderm, mesoderm) interact to contribute to all cell lineages of the adult mouse [9], [10]. Pluripotent ES cells have the ability to self-renew indefinitely when maintained in pluripotent culture conditions [11]. Given the dynamic potential of ES cells, this platform harbors key signaling pathways and biological networks that govern their pluripotency and/or differentiation. In addition, ES cells have been shown to mimic many aspects of early embryonic development [12] and are amenable to systems-level analysis utilizing high-throughput screening (HTS) and high-content screening (HCS) methods.

Taking advantage of the developmental potential of ES cells, the embryonic stem cell test (EST) was established over a decade ago as an alternative method for assessing chemical embryotoxicity [13]. The EST uses three separate endpoints (cardiomyocyte differentiation, ES cell viability, fibroblast viability) in a prediction model to evaluate chemical toxicity and affords up to 78% accuracy [14], [15], [16]. In spite of this predictive attribute, there are drawbacks to the EST assay: 1) subjective nature of measuring cardiomyocyte differentiation based on qualitative observations, 2) viability and differentiation measures are collected from separate cultures, and 3) limited throughput. Others have expanded on the original foundation of the EST using transcriptomics in mouse ES cells or metabolomics of pluripotent human ES cells to identify developmental toxicants [17], [18].

Here, we utilize a modification to deliver a high-throughput, sensitive method for quantitatively measuring cytotoxicity and cardiomyocyte differentiation. Furthermore, we have introduced this platform to the ToxCast portfolio for *in vitro* profiling of 309 environmental chemical compounds across ∼500 different assays. This confers a unique platform to the ToxCast portfolio for chemical screening as a complex cell culture system that mimics early mammalian embryonic development and maintains relevant signaling networks required for primary germ layer formation. We hypothesize that the ES cell assay may identify toxicity pathways in ES cell differentiation that are predictive of developmental activity of chemicals. Towards this end, concentration-response relationships for 309 environmental chemicals were measured in the ES cell assay. A subset of 56 of these chemicals showed a statistically significant response across one or more of the following four parameters: increased cytotoxicity, decreased cytotoxicity, decreased differentiation,μ and increased differentiation. For each chemical-by-endpoint combination showing significant activity, we calculate an AC_50_ (concentration at which activity is 50% of maximal change). This report describes the results of these experiments and provides the first computational (*in silico*) model of a toxicity-related pathway underlying ES-based developmental bioactivity across the chemical library.

## Materials and Methods

### ES cell maintenance and differentiation

J1 male murine ES cells from the 129S4/SvJae strain were obtained from ATCC (www.atcc.org) and maintained according to their recommendations for propagation and subculturing (ATCC, SCRC-1010™). In brief, J1 cells were cultured on Mitomycin C-treated primary mouse embryo fibroblasts (MEFs, Millipore, Cat. No. PMEF-CF) on 0.1% gelatin (Millipore, Cat. No. SF008) in ES cell media containing Knockout DMEM (Invitrogen, Cat. No. 10829), 15% ES cell-qualified fetal bovine serum (Invitrogen, Cat. No. 10439), 2 mM GlutaMAX (Invitrogen, Cat. No. 35050), 0.1 mM non-essential amino acids (Invitrogen, Cat. No. 11140), 50 U/50 ug/mL Pen/Strep (Invitrogen, Cat. No. 15140), β-mercaptoethanol (Gibco, Cat. No. 21985), and 1000 U/mL mouse leukemia inhibitory factor (mLIF, Millipore, Cat. No. ESG1107). Prior to evaluating the effects of chemicals, MEFs were removed from cultures prior to an experiment by passing ES cells onto 0.1% gelatin-coated flasks in MEF-conditioned media with mLIF to maintain pluripotency. On day 0, pluripotent MEF-depleted ES cells were collected and cell counts were determined with a NucleoCounter® NC-100 (ChemoMetec). Cells were seeded at a density of 1000 cells/well onto 0.1% gelatin-coated 96-well plates (Costar, Cat. No. 3596) in ES cell media without mLIF (differentiation conditions) and allowed to adhere to the plates overnight. ToxCast chemicals were introduced on day 1 and subsequently refreshed on days 6–8.

### Chemical Library

This study used the ToxCast Phase I, version 1 chemical library which contains 320 total chemicals with the following breakdown: 309 unique chemical structures, 5 independently sourced duplicates, and 3 triplicates that serve as plating replicates for internal quality control [6]. The majority of these chemicals had bioassay data available from ToxRefDB guideline animal studies (rat, mouse, rabbit), 97.5% are soluble in DMSO, 90% have a molecular weight between 250–1000, and 98.1% are commercially available with >90% purity. Information on the chemical library is publically available (http://www.epa.gov/NCCT/dsstox/sdf_toxcst.html). Chemical procurement and stock preparation was outsourced to BioFocus DPI (South San Francisco, CA) and certificates of analysis were provided with plated chemicals. Plated chemicals were subjected to a QC check and the results are publically available (http://www.epa.gov/ncct/toxcast). Due to the effects of DMSO on the ES cells and the maximum concentrations of chemicals plated by BioFocus DPI (20 mM), we were limited to 12.5 µM as the highest concentration evaluated.

### Cytotoxicity and Differentiation Measurements

In-Cell Western™ (Li-Cor Biosciences) assays were initiated on cell culture day 9 and performed according to the manufacturer's protocol. In brief, cells were fixed in the assay plate, incubated with primary antibody, secondary antibody, and DNA/cell stains, then assay plates were scanned on a Li-Cor Odyssey® Infrared Imaging System. This system allows for detection of two endpoints in each well by measuring signal intensity in two infrared channels. Cardiomyocyte differentiation was measured using a primary mouse antibody that recognizes α,β cardiac Myosin Heavy Chain (MYH6/MYH7)(Abcam, Cat. No. ab15; 1∶1000). Cells were incubated in primary antibody overnight at 4°C with gentle agitation. A goat α-mouse secondary antibody conjugated to IRDye® 800CW (Li-Cor, Cat. No. 926-32210; 1∶2000) was detected at 800 nm. Cytotoxicity was assessed using Sapphire700™ (1∶2000) relative cell and DRAQ5™ (1∶16,000) DNA stains (Li-Cor, Cat. No. 948-40022) were detected at 680 nm. Cell stain and MYH6/MYH7 intensities are corrected for background signal using intensity values from control wells, which were incubated without primary antibody or cell stains. Quality control checks were performed by visual inspection of the plate images and removal of obvious outlier data due to technical issues. MYH6/MYH7 signal was normalized to relative cell number (cytotoxicity) by dividing MYH6/MYH7 intensity by Sapphire700™/DRAQ5™ intensity from the same wells. Cytotoxicity and cytotoxicity-corrected cardiomyocyte differentiation values were made relative to the zero dose control.

### Dose-response curves and AC_50_ calculations

Relative cytotoxicity and cardiomyocyte differentiation values were analyzed to detect statistically significant concentration-dependent behavior. Concentration-response analysis used software developed in-house in the R language, run using R version 2.8.1 (software available upon request). First, the variation of 0-concentration control (i.e. DMSO vehicle) values was assessed for all experiments by calculation of the mean and standard deviation. Then, a cutoff was established by taking the mean of the 0-concentration controls +/−2 standard deviations. Values that fell outside the cutoff were considered different from control. If all values for a treatment fell within the established 0-concentration control variation, the treatment was said to have no effect, a curve fit was not performed, and a default AC_50_ value of 1 M was assigned to that combination. If there was a significant change from baseline, the concentration-response data were fit to a four-parameter Hill curve using nonlinear least-squares regression. The AC_50_ was then assigned as the dose along the curve that produced a 50% change from the mean of the 0-concentration control. Separate analyses were performed for curves that exhibited an upward trend versus a downward trend. For data that exhibited a downward trend, the 0-concentration control value of 1 was set at the top (100%) and complete reduction of signal was the bottom (0%). For the upward trend analysis, data were adjusted by subtracting 1 which made the 0-concentration equal to the bottom (0%). A 100% increase from the 0-concentration control represented the top and was set to 1 (100%). AC_F_ were calculated using the AC_50_ values in the context of the following equation (Eq.1), where AC_F_ is the concentration at which there was F percent change in activity relative to the 0-concentration. F is the chosen percentage of change to be evaluated and *Hillslope* is the calculated slope of the non-linear regression curve fit from the R analysis:
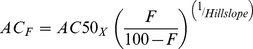
(1)Heatmaps were produced with Partek Genomic Suite 6.4 software (Partek, Inc., St. Louis MO). Unsupervised, two-dimensional hierarchical clustering was performed using Euclidean distance for measure and Ward's method for linkage analysis.

### Predictive models

Data (in the form of AC_50_ values) for the ES assays were analyzed by comparison with a large number of other *in vitro* assays measured in the same chemical library. These comprise the ToxCast data set described elsewhere [3], [4], [5], [6], [7], [19]. The complete data set consists of a matrix of 309 chemicals (rows) by 548 assays (columns). The values are AC_50_'s for the chemical-assay combinations, with a default value of 1 M in cases where no statistically significant chemical-assay activity was observed. Pathway, process and disease-based perturbation scores (PS) were constructed by mapping assays to genes and then to collections of genes in pathways from KEGG, Ingenuity, Pathway Commons, Gene Ontology (GO) and OMIM. In brief, a chemical-pathway PS corresponds to the minimum AC_50_ for any assay for that chemical mapping to the pathway. The PS form additional columns in the data matrix. The details for calculating PS for a chemical-pathway combination is described elsewhere [6].

In addition to the ToxCast *in vitro* assays, we also have *in vivo* animal-based endpoints for a majority of these chemicals (from chronic, cancer, reproductive developmental toxicity studies), extracted from the EPA Toxicity Reference Database (ToxRefDB, http://www.epa.gov/NCCT/toxrefdb/) [3], [4], [5]. These data are quantified as LEL (lowest effective level) values which give the lowest dose at which an endpoint was observed, and form extra columns to the data matrix.

We calculated univariate statistical associations between the ES cell data and both the ToxCast *in vitro* assays and ToxRefDB *in vivo* endpoints. Two statistical tests were used. In the first, the data matrix was dichotomized so that if activity was seen at any concentration (assays) or dose (endpoints), a value of 1 was assigned to the chemical- assay (endpoint) pair. Otherwise a value of 0 was assigned. Next, one assay was selected as the input (predictor variable) and another assay (or endpoint) as the output (predicted variable). A 2×2 contingency table was created with value TP (true positive, number of chemicals for which the input and output were both positive), FP (false positive, number of chemicals for which the input was positive and output negative), FN (false negative, number of chemicals for which the input was negative and the output positive) and TN (true negative, both input and output negative). The significance of association was tested using a Fisher's exact test. In the second statistical method, the input assay AC_50_ values were log transformed and scaled:
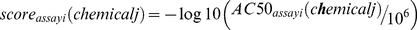
(2)This scaling yields a value of zero for inactive chemical-assay combinations. The output variable is dichotomized as before. We then perform a t-test comparing the score distribution for the output-positive vs. output-negative chemicals.

We also constructed multivariate models to predict activity of chemicals in the ES assays from data for multiple other *in vitro* assays. For this analysis, the original data matrix was log-transformed (Eq. 2). The model is of the form:
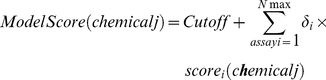
(3)where if 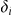
s 1, then assay *i* is included, otherwise it is not. If the model score for chemical *i* is >0, the chemical is predicted to be active in the output assay or endpoint, otherwise it is predicted to be inactive. Assays are added to the sum in a stepwise fashion, so that the one with the most significant univariate association (using either of the statistical tests described above) is added first, the second most significant is added next and so on. Model performance is evaluated using a 2×2 contingency table as described above where the true activity vector for the output assay is compared with the predicted activity vector. The model is implemented using a k-fold cross validation algorithm in which the data is divided into training (80%) and test (20%) portions and optimal values of *Nmax* and *Cutoff* are found which maximize the area under the curve (AUC) of the Receiver Operator Characteristic (ROC) curve. In addition to the AUC and Fisher's exact p-value, we also calculate the sensitivity, specificity, balanced accuracy (BA, average of sensitivity and specificity) and other metrics. The algorithm is implemented in R and is available upon request (“linmod.R” (NCCT, US EPA)). ROC curves are built to predict the ES assays as outputs and used the ToxCast assays and pathway perturbation scores as inputs.

## Results

### Mouse ES cell assay

**Figure 1:**
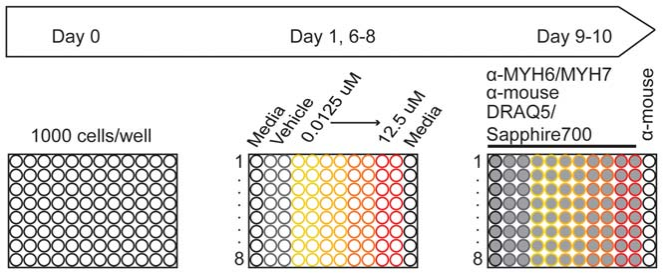
ES cell assay overview. ES cells are seeded onto gelatin-coated 96-well plates at Day 0 in the absence of pluripotent factors. Eight chemicals (color outlined circles) are introduced on Day 1 at four concentrations (yellow→red; 0.0125, 0.125, 1.25, 12.5 uM) and are subsequently refreshed on Days 6–8. In-Cell Western™ analysis is initiated on Day 9 to assess differentiation (MYH6/MYH7) and cytotoxicity (DRAQ5/Sapphire700). Vehicle (grey outlined circles) and antibody controls (black outlined wells, Day 9–10) are included on each plate.

**Figure 2:**
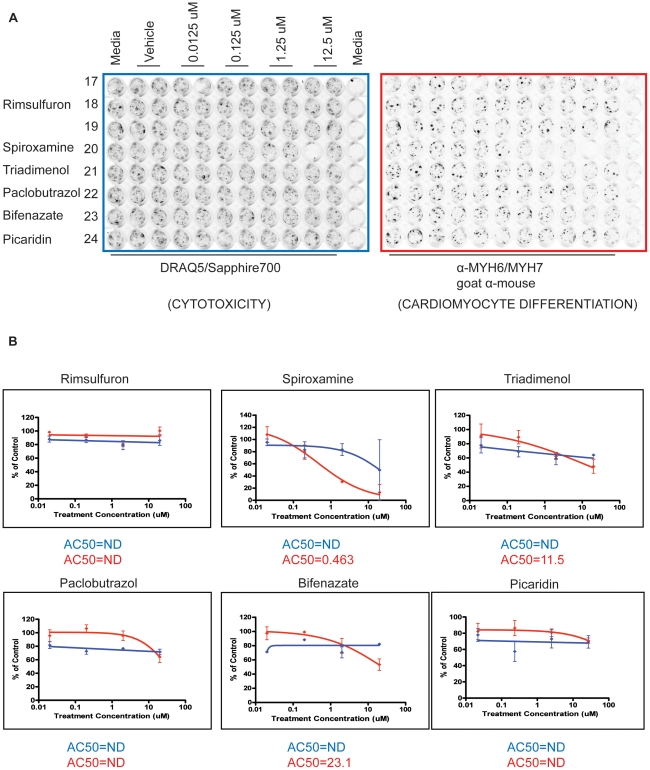
Dose-response curves generated for cytotoxicity and differentiation. Images of experimental plate show cell number and differentiation signal for chemicals 17–24 across the concentration range tested (A). Dose-response curves for six chemicals from the above plate as well as AC_50_ values are shown (B).

We evaluated the effects of 309 chemicals in duplicate across four concentrations on cardiomyocyte differentiation and cell number in a ten-day, high-throughput mouse ES cell adherent cell differentiation and cytotoxicity assay (Figure 1). Using a 96-well format, we tested eight chemicals per plate in duplicate, one per row, at concentrations ranging from 0.0125 to 12.5 µM (Figure 2A). Curves generated from the concentration-responses of the chemical library indicate that under the conditions employed here, the assay was responsive to a subset of 309 chemicals. For example, Rimsulfuron had no measurable biological activity in the ES cell system (Figure 2B). Spiroxamine was a potent inhibitor of cardiomyocyte differentiation, yet had no significant effect on cytotoxicity (Figure 2B). Other chemicals, such as Paclobutrazol and Picaridin, began to show effects on differentiation, but not enough to calculate an AC_50_ (Figure 2B). A small number of chemicals (n = 5) produced effects on cytotoxicity or differentiation that did not resemble traditional upward or downward curves. Instead, these chemicals generated a U-shaped or inverse U-shaped trend. All 320 concentration response curves are provided (Figure S1). Overall, these results show the assay is capable of detecting chemical effects on number and differentiation of ES cells.

Eighteen percent of the chemical library (56/309 chemicals) produced effects permitting AC_50_ values to be calculated for the ES cell assay (Table 1). Of the AC_50_ values calculated, 25% (18/69) exhibited chemical potencies below one micromole. The most potent chemical in our cytotoxicity assay was Captafol (AC_50_ = 0.017 µM), while the chemical with the most pronounced effect on decreased cardiomyocyte differentiation was Rotenone (AC_50_ = 0.13 µM) (Table 1). Thirty-two percent of AC_50_ values (22/69) were greater than 10 µM. The least potent chemical of those which produced a 50% change in the cytotoxicity and differentiation assays was Difenoconazole (AC_50(Cytotoxicity)_ = 17 µM, AC_50(Differentiation)_ = 18 µM) (Table 1). Most AC_50_ values were calculated from decreased cell number and/or decreased differentiation (82.6%, 57/69). However, four chemicals increased differentiation and eight chemicals increased cell number in our assay. Dicrotophos increased cardiomyocyte differentiation (AC_50_ = 0.45 µM). Likewise, Mancozeb increased cell number with similar potency (AC_50_ = 0.43 µM).

**Table 1:** AC50 values calculated and arranged according to potency.

	Cytotoxicity		Differentiation			Cytotoxicity		Differentiation	
Chemical	Decrease	Increase	Increase	Decrease	Chemical	Decrease	Increase	Increase	Decrease
Rotenone		0.20		0.13	Thiram		0.11		
Nitrapyrin		0.081		0.18	Fluazinam		0.22		
Trifloxystrobin		0.72		0.39	Cyromazine		1.3		
Fluoxastrobin		0.80		0.99	Dicamba		1.4		
Niclosamide		0.32		1.0	Diquat dibromide		2.1		
Propargite		1.5		1.2	Imazamox		2.5		
Maleic hydrazide		0.39		1.3	Naled		2.5		
IPBC		2.2		1.4	Phosalone		2.7		
Azoxystrobin		1.7		5.0	Milbemectin		2.9	2.7	
Etoxazole		14		6.6	Benomyl		3.0		
Folpet		0.65		10	Abamectin		3.4		
Fentin		1.3		12	Mepiquat chloride		5.4		
Thidiazuron		12		15	TCMTB		6		
Difenoconazole		17		18	Fludioxonil		11		
Spiroxamine				0.47	Maneb		12		
Diniconazole				1.2	Buprofezin		17		
Triflumizole				5.8	Dicrotophos			0.45	
Methoxyfenozide				6.3	Diuron			9.7	
Triadimenol				8.2	Permethrin			15	
Propiconazole				9	Mancozeb	0.43			
Triclosan				10	Captan	4.6			
Endosulfan				11	Cyazofamid	5.1			
Imazalil				11	Bensulide	9.1			
Prochloraz				12	Oxadiazon	12			
Bifenazate				15	Dicofol	13			
Butafenacil				16	Profenofos	14			
Captafol		0.017			Fluroxypyr-meptyl	15			
Acetamiprid		0.019			Sulfentrazone	17			

**Figure 3:**
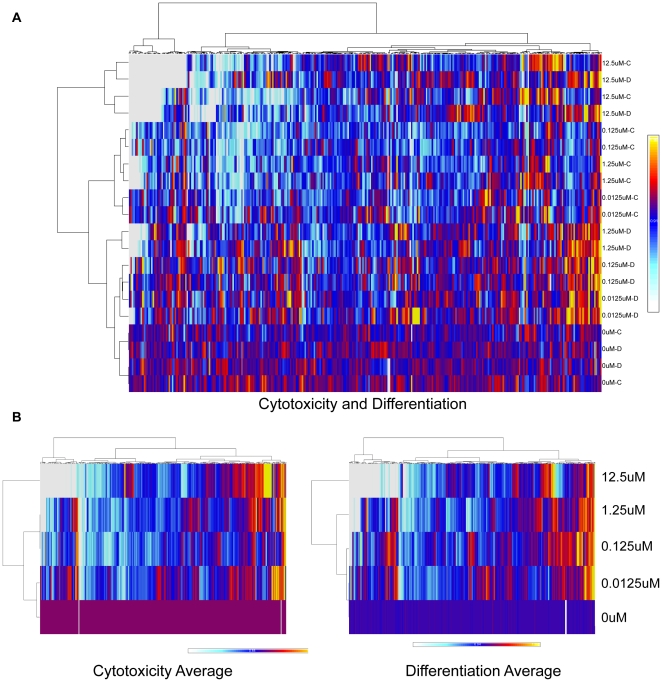
ToxCast™Phase I chemical activity across cytotoxicity and differentiation ES cell assay endpoints. Heatmaps depict chemical effects on cytotoxicity and differentiation across dose. Unsupervised, two-dimensional hierarchical clustering of 320 ToxCast chemicals across four ES cell assay endpoints (cytotoxicity increase, cytotoxicity decrease, differentiation increase, differentiation decrease) and 4 doses each using Euclidean distance for measure and Ward's method for linkage analysis (A). Two-dimensional individual clusters for averaged dose normalized to controls for cytotoxicity and differentiation endpoints (B). Heatmap scales represent relative activity based on AC_50_ = −log10(M), ranging from 0 (white) to 2 (yellow).

The chemical library included three chemicals run in triplicate to assess assay replicability. The coefficient of variation for the replicates on both cytotoxicity and differentiation measurements at each dose including vehicle controls was less than 22%, indicating good replicability. In addition, a global view of the chemical library demonstrated varied activity of chemicals across concentration (Figure 3). The most potent chemicals killed all of the cells as indicated by the white block in the top left corner of the heat map (Figure 3). The highest concentrations clustered together while the vehicle dose formed a separate cluster indicating their similar activity profiles (Figure 3A). Averages for cytotoxicity and differentiation measurements clustered the doses in order from lowest concentration to highest concentration for both parameters (Figure 3B).

### Univariate associations between ES cell assay endpoints and ToxCast assays

The ToxCast data set includes over 500 assays run using cell-free and cell-based format in nine separate technologies. The cell-based assays use a variety of primary cell types and cell lines [6]. Data from all 309 chemicals across these assays (as well as the derived pathway-based perturbation scores and whole animal toxicity endpoints) was used to build univariate and multivariate models using the ES cell assay as both input and output variables.

**Figure 4:**
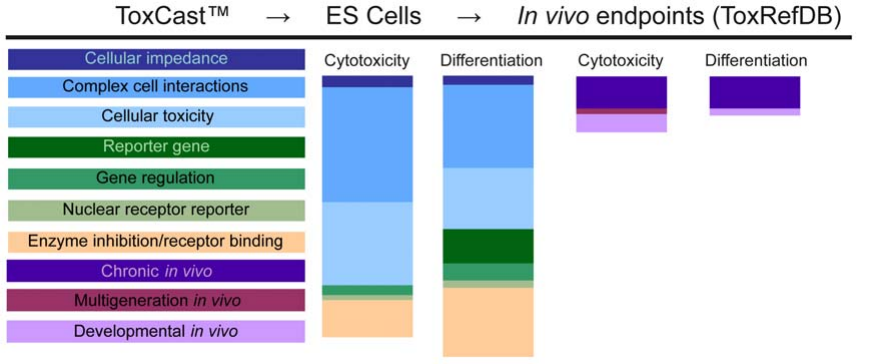
Associations between ToxCast™ assays and ES cell endpoints. Univariate associations revealed multiple ToxCast assays correlate with ES cell cytotoxicity and differentiation. ES cell cytotoxicity and differentiation correlated with limited *in vivo* endpoints in ToxRefDB.

We used the ES cell endpoints as inputs to mine the assay space for significant correlations (Figure 4). The ES cell cytotoxicity endpoint (decreased relative cell number) associated with 98 endpoints in the ToxCast assay suite (p≤0.1). The majority of endpoints that correlated with ES cell cytotoxicity were from human cell-based platforms that reveal disruption of cell-cell signaling (e.g. BioSeek) and cellular features (e.g. Cellumen) (Figure 4). Many of the former endpoints represented immune system responses or components such as cytokines (Figure S2). The latter endpoints that associated significantly with ES cell cytotoxicity were general measures of cytotoxicity such as apoptosis or DNA damage (Figure S2), indicating the cell number endpoint in the ES cell assay was consistent with other cell-based cytotoxicity endpoints. In addition, ES cell cytotoxicity had significant correlations to mouse, rat and rabbit ToxRefDB *in vivo* endpoints (Figure 4), including delayed pubertal development in multigenerational rat studies and cranial malformations in prenatal rabbit studies.

Decreased cardiomyocyte differentiation associated with slightly fewer endpoints than did cytotoxicity (k = 88, p≤0.1) (Figure 4). Again, more than 80% of associations were to the aforementioned human cell-based platforms (BioSeek, Cellumen). The ES cell differentiation endpoint associated with mouse and rat ToxRefDB *in vivo* endpoints, including renal defects in developmental rat studies. However, unlike the increased cytotoxicity and decreased differentiation endpoints, the decreased cytotoxicity and increased differentiation endpoints had limited univariate associations across the ToxCast assay space (cytotoxicity decrease: k = 16, p≤0.1; differentiation increase: k = 1, p≤0.1).

### Univariate associations between ToxCast assays and ES cell endpoints

Using the reversed strategy, the ToxCast assay targets (k>500) were used as inputs to look for associations with the ES cell endpoints (k = 4). One hundred seven ToxCast assay endpoints associated with ES cell cytotoxicity. All but two of these associations were from cell-based assay platforms (Figure 4). A similar number of ToxCast assay targets associated with ES cell differentiation (k = 112). We found 21 transcriptional activity-based endpoints from two different platforms (Attagene, CellzDirect) [6], [19] that correlated with ES cell differentiation. When we looked deeper into the reporter gene responses, a striking finding emerged that many of these responses represented critical developmental pathways (Figure S2). For example, Bone morphogenetic protein receptor type II (*BMPR2*) showed a significant association with decreased cardiomyocyte differentiation in the ES cell assay (p = 0.01). In mice, *Bmpr2* is required for normal mesoderm development [20]. Likewise, Paired box gene 6 (*PAX6*) had a significant association with an inhibition on ES cell differentiation (p = 0.12). *Pax6* mutations in mice produce a range of phenotypes including eye, craniofacial and brain defects [21]. Increased transcriptional activity of *OCT1* was significantly associated with decreased cardiomyocyte differentiation (p = 0.03). Oct1 has recently been shown to be required for normal trophoblast development in mice [22]. In addition to associations with developmentally regulated genes, multiple assay endpoints present in the oxidative stress signaling pathway were strongly associated with decreased ES cell differentiation. For example, increased transcriptional activity of *NRF2* (p = 0.008), a classic oxidative stress-responsive gene [23], [24], was associated with decreased cardiomyocyte differentiation. Likewise, *JUN* (p = 0.008), *ABCG2* (p = 0.001), *GSTA2* (p = 0.05), and *HIF1A* (p = 0.06) correlated with decreased ES cell differentiation. Finally, increased transcriptional activity of *EGR1* (p = 0.04) and *OCT1* (p = 0.03), which have been shown to play a role in oxidative stress pathways [25], [26], [27], are highly correlated with decreased ES cell differentiation. Taken together, these results predict that chemicals which perturbed cardiomyocyte differentiation in ES cells perturbed multiple components involved in reactive oxygen species signaling pathways in addition to critical developmental pathways.

### Multivariate associations

**Figure 5:**
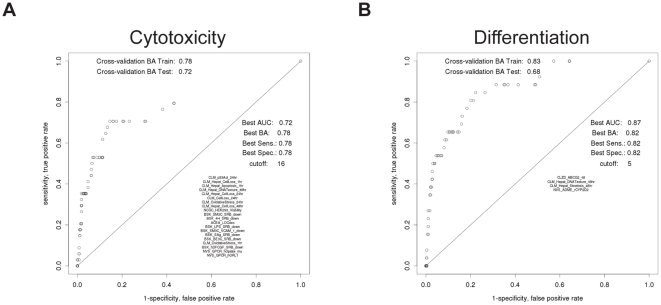
Multivariate models predict ES cell cytotoxicity and differentiation. ROC curves generated using data from ToxCast assays and ES cells to drive machine learning algorithms that produced predictive models of ES cell cytotoxicity (A) and differentiation (B).

In an effort to identify more complex patterns between the ToxCast assay collection and cytotoxicity and/or differentiation in our ES cell assay, machine learning algorithms were applied to generate multivariate models that allowed us to develop hypotheses for future testing. The predictors in the best model of ES cell cytotoxicity comprise a collection of cell-based assays that measured general cytotoxicity perturbations and decreased immune system components (Figure 5A). This model had a balanced accuracy of 0.78, indicating its strength. The top feature that arose from the predictive model of ES cell cytotoxicity was the activation of *p53*, the canonical DNA damage and oxidative stress response [28]. We also assessed the ability of the ToxCast assays to generate a model which predicted decreased cardiomyocyte differentiation in the ES cell assay. Although the predictive assays for this model also included several measuring general cytotoxicity, a significant feature was the gene expression-based assay for ATP-binding cassette sub-family G member 2 (*Abcg2*) (Figure 5B). Abcg2 is a xenobiotic half-transporter with wide substrate specificity and expression in multiple sites where protection from toxicants is critical [29]. In addition, *Abcg2* is highly expressed in the Harderian gland [30], a chronic *in vivo* endpoint effect highly correlated with decreased ES cell differentiation (Figure 4).

## Discussion

ES cells are an attractive platform to assess developmental toxicity because they are capable of recapitulating many of the differentiation states and rely on signaling pathways present in development. We used a mouse ES cell adherent cell differentiation and cytotoxicity assay to assess the activity of a group of chemicals (mainly pesticide active ingredients), most of which have *in vivo* toxicity data. The results of this study demonstrated the following: 1) a subset of tested chemicals were active in the ES cell assay, 2) general cytotoxicity assays from the ToxCast program were strongly associated with ES cell cytotoxicity assays, 3) transcriptional activity assays were strongly associated with ES cell differentiation assays, 4) statistical analysis identified predictive models of increased ES cell cytotoxicity and decreased ES cell differentiation for a subset of the tested chemicals.

This study examined the biological activity of 309 environmental chemicals in a mouse ES cell adherent cell differentiation and cytotoxicity assay. The AC_50_ cutoff was used across the entire ToxCast portfolio, therefore this was also the benchmark cutoff in the ES cell assay. In addition, we selected the AC_50_ as an appropriate level to determine the most potent developmental toxicants. Based on the magnitude of effects, AC_50_ values were calculated for 18% of the environmental chemicals tested. Because the current study used the same plating library as other assays in the ToxCast portfolio and because of the requirement to limit the DMSO vehicle concentration, the present analysis was limited to testing chemicals at a maximum concentration of 12.5 µM. As such, the results reported here focus on chemicals that were active with AC_50_'s at these relatively low concentrations. Chemicals that did not achieve a 50% change in cytotoxicity or cardiomyocyte differentiation at the concentration range tested may be biologically active, but not within this concentration range. Additionally, because these studies were limited to an assessment of cardiomyocyte differentiation, effects on ES cell differentiation to other cell lineages may have been produced but not evaluated. Other studies have shown a 30-fold change in chemical potency when tested with two distinct ES cell lineage markers [31]. Finally, many signaling pathways may be needed to fully evaluate developmental toxicity in ES cells [32]. Expanding the endpoints in our ES cell assay to monitor more diverse cell lineages and signaling pathways as well as assessing alternative cutoff values would be expected to identify a greater number of chemical actives.

ToxCast enables the unique ability to correlate chemical effects on the ES cell endpoints with a broader suite of ∼500 assays. Such an analyses revealed many novel relationships with ES cell cytotoxicity based on their strong correlation for chemicals that were active in human cell-based cytotoxicity assays. This bolsters confidence in the use of the cytotoxicity assessment reported here for evaluation of cytotoxicity between murine-human culture models and seems to indicate that there are fundamental cellular processes affected by these chemicals, rather than a developmentally-specific response. Future studies to compare the concentration-dependent effects between adult and ES cells may identify unique sensitivity in the ES cells. Additionally, evaluating chemical agonists and antagonists of known developmental signaling targets may be useful for determining the ability of the ES cell cytotoxicity endpoint to identify perturbations in developmental signaling.

A group of transcriptional activity human cell-based assays exhibited a strong association with decreased ES cell differentiation. This association was specific to chemicals that produced a 50% decrease in cardiomyocyte marker expression, but not chemicals that produced cytotoxicity. The developmental relevance of several genes that associated with inhibition of cardiomyocyte differentiation stands without question and furthermore suggests this endpoint does, as anticipated, identify key targets in mechanisms that are not detected by generic cytotoxic response.

Each ES cell endpoint was correlated with adverse developmental effects *in vivo*. In addition, each ES cell endpoint correlated to unique developmental effects indicating the strength of using both endpoints in future predictive models. However, the spectrum of adverse developmental effects associated with perturbation of ES cell endpoints was limited and effects on many organ systems were not correlated. The limited correlation between *in vivo* and *in vitro* surprising given the requirement of three independent assays to achieve predictivity using the EST [13]. By increasing the chemical concentration range evaluated, broadening the coverage of differentiation states assessed, and integrating the differentiation and cytotoxicity endpoints, we may expect to increase the ES cell assay's predictivity of *in vivo* toxicity. Further investigation into chemicals that produced effects *in vivo*, but not in the ES cell assay may provide valuable insight into species-specific toxicities and allow us to define potential limitations of mouse ES cells in toxicity testing. It is prudent to identify other assays that, in combination with the ES cell platform, strengthen the predictive model for *in vivo* associations.

**Figure 6:**
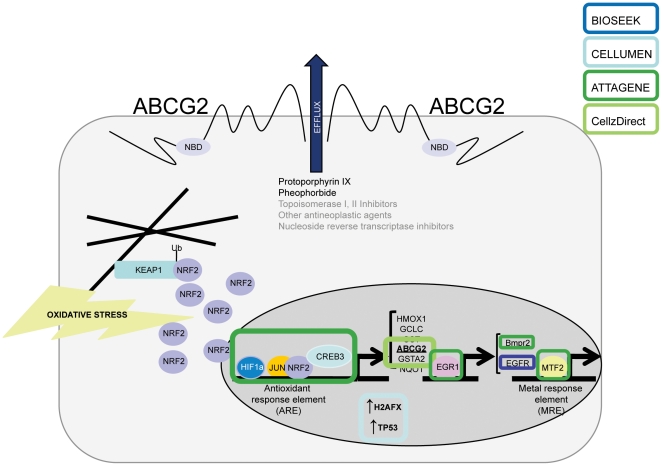
Cartoon depicting chemical targets across multiple components of ROS signaling in ToxCast platforms. Chemicals that decreased ES cell differentiation by at least 50% also targeted multiple components of ROS signaling. The statistical associations between decreased ES cell differentiation and ToxCast assay platforms are highlighted (p≤0.1).

The results reported here provide valuable information on the potential pathway-level responses of ES cells. Univariate and multivariate associations identified a collection of features that predict decreased differentiation in ES cells. Many of these features play a role in reactive oxygen species (ROS) signaling (Figure 6). For instance, *Oct1* has been identified as a critical regulator of gene transcription during oxidative stress [33] and knockout mutant mice showed hypersensitivity to oxidative stress [34]. *Bmpr2* is modulated under hypoxic conditions [35] and these knockout mice are more susceptible to hypoxic pulmonary hypertension [36]. *Hif1a* is responsive to oxidative stress [37], [38] and is required for maintenance of normal oxygen levels throughout development [39]. *Jun* has been shown to associate with *Nrf2*, an oxidative stress responder [40], and enhance activation of the antioxidant response element (ARE), a binding motif that plays a role in antioxidant gene regulation [41], [42], [43], [44], [45], [46]. *Gsta2*, an ARE-containing gene, exhibits significantly reduced expression in *Nrf2* ^−/−^ mice [47]. The top feature that predicted decreased ES cell differentiation, *Abcg2*, is also regulated by hypoxic conditions and contains multiple putative AREs [48]. *Abcg2* promoter constructs were mutated in putative ARE sites and tested *in vitro* resulting in decreased promoter activity suggesting functionality [48]. Furthermore, electromobility shift assays indicated *Hif1a*, a transcription factor assay highly associated with decreased ES cell differentiation (Figure 4), binds to the ARE sequence proximal to *Abcg2* [48]. In the context of stem cells, *Abcg2* is expressed in both mouse [49] and human [50] ES cells as well as numerous tissue-specific stem cell side populations [51]. *Abcg2* expression is widely used as a marker of stem cell populations, although its role in stem cells is not entirely known [52]. Overexpression of *Abcg2* inhibits differentiation while lack of *Abcg2* expression promotes differentiation of stem cells [53]. Identification of *Abcg2* as a target of Notch signaling as well as its role in preserving stem cell pluripotency suggests *Abcg2* may function to maintain self-renewing stem cell populations [53]. It would be interesting to assess gene expression in the ES cell assay and determine whether: 1) *Abcg2* is upregulated upon exposure to the chemicals driving this predictive model, 2) ES cells maintained in oxidative stress conditions mimic the gene expression profile from chemical-treated ES cells, and 3) whether ES cells exposed to this subset of chemicals exhibit stem-like features.

Efforts continue towards using ToxCast and ES cell assays to identify toxicity pathways and predict *in vivo* toxicity endpoints. Towards this end, Phase II of ToxCast is underway with the addition of approximately 700 chemicals including many compounds with toxicity data from failed clinical trials as well as chemicals used to assess the original EST.

## Supporting Information

Figure S1 **Concentration-response curves of ToxCast Phase I chemical library in mESCs.** Chemicals were evaluated for their potential effects on mESC cytotoxicity (increase, decrease) and/or differentiation (increase, decrease).
(PDF)

Figure S2 **Statistical associations between ES cell endpoints and ToxCast or ToxRefDB targets.**
(TIF)
