# Supplementary material for: Evaluation of 309 Environmental Chemicals Using a Mouse Embryonic Stem Cell Adherent Cell Differentiation and Cytotoxicity Assay
Source: PLoS One. 2011 Jun 7;6(6):e18540. doi: 10.1371/journal.pone.0018540 (PMC3110185; doi:10.1371/journal.pone.0018540)
Supplement: Figure S1 — Concentration-response curves of ToxCast Phase I chemical library in mESCs. Chemicals were evaluated for their potential effects on mESC cytotoxicity (increase, decrease) and/or differentiation (increase, decrease). (PDF) [file pone.0018540.s001.pdf]

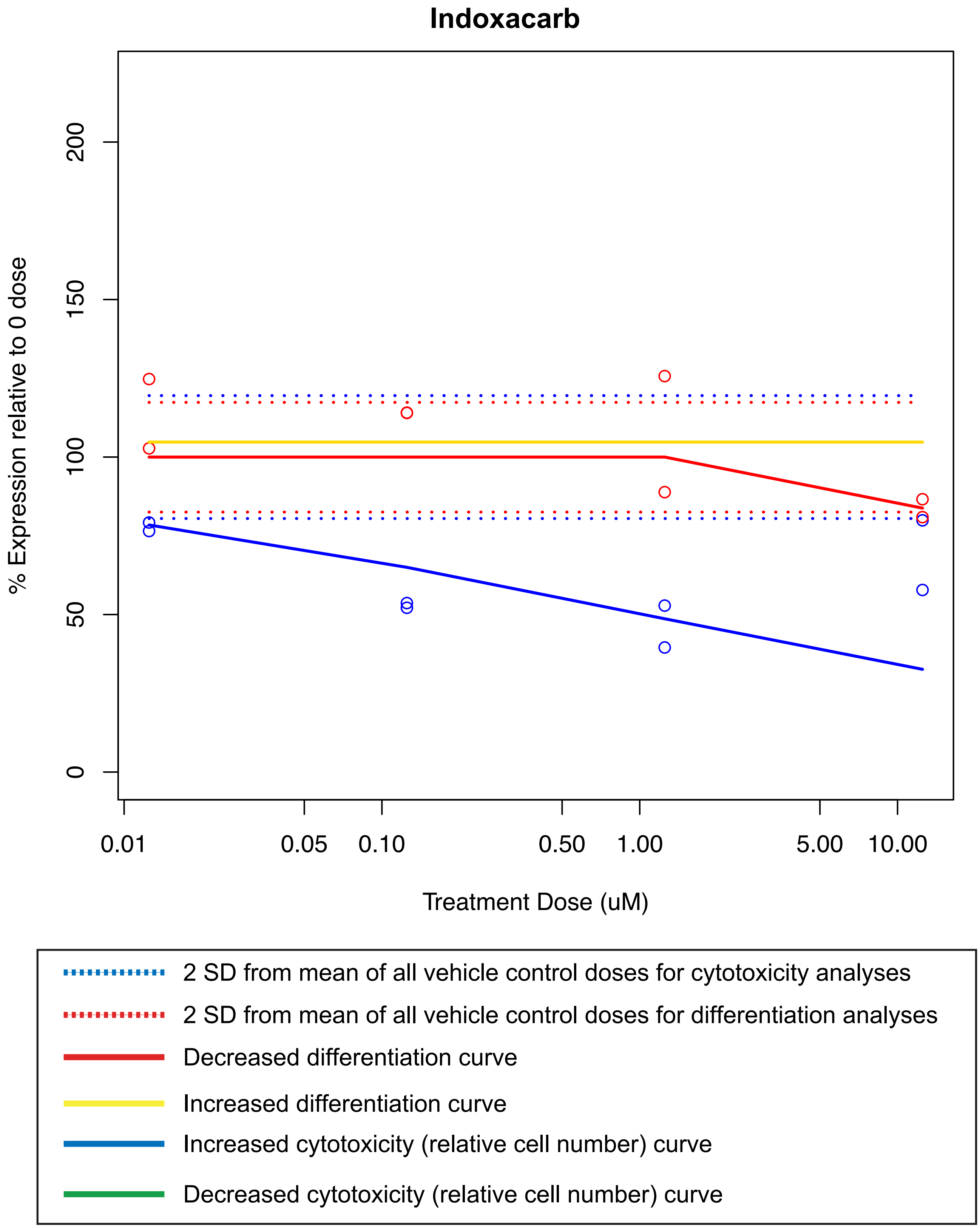
Indoxacarb
% Expression relative to 0 dose
0
50
100
150
200
0.01
0.05
0.10
0.50
1.00
5.00
10.00
Treatment Dose (uM)
2 SD from mean of all vehicle control doses for cytotoxicity analyses
2 SD from mean of all vehicle control doses for differentiation analyses
Decreased differentiation curve
Increased differentiation curve
Increased cytotoxicity (relative cell number) curve
Decreased cytotoxicity (relative cell number) curve

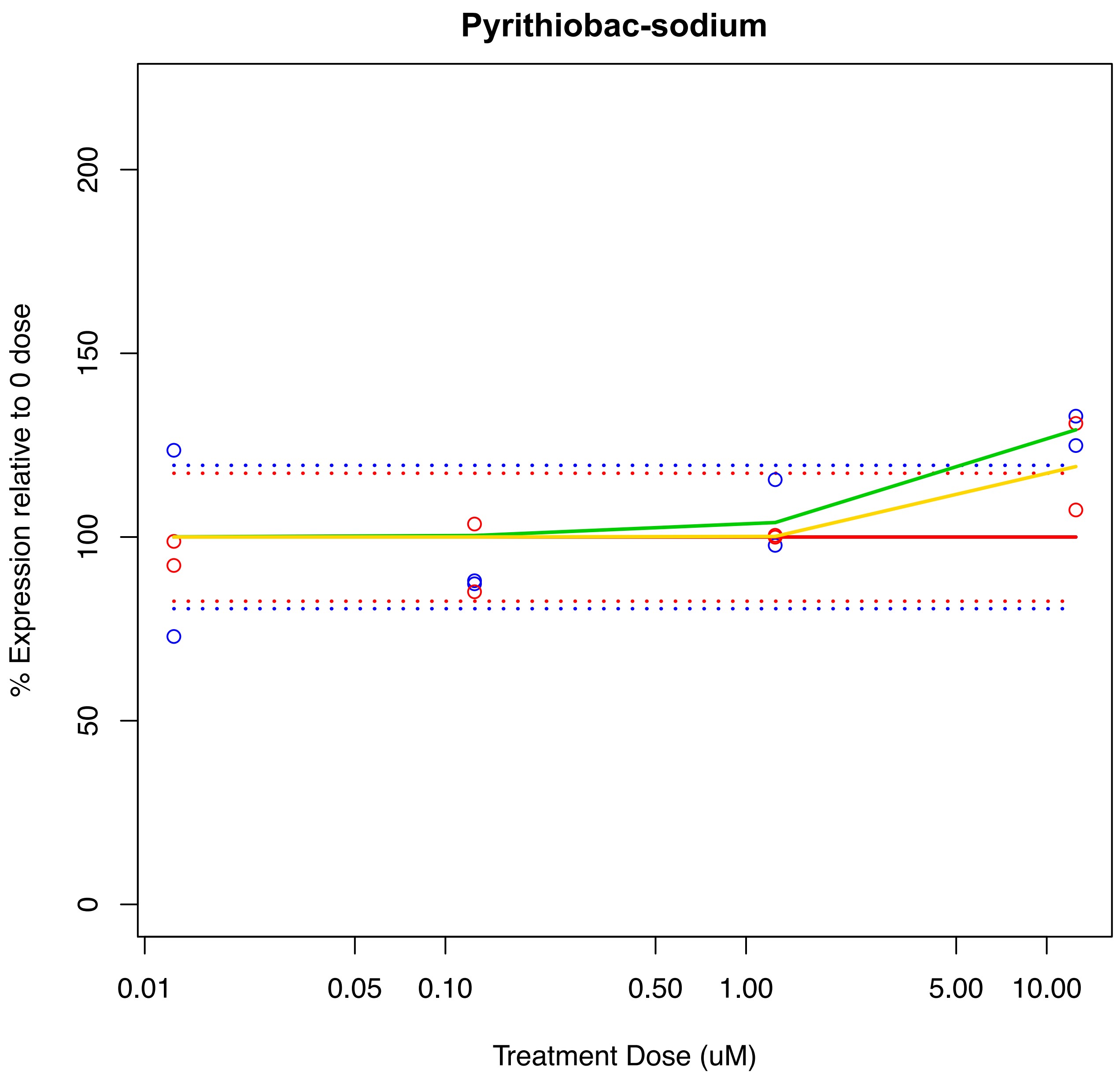

Pyrithiobac-sodium
% Expression relative to 0 dose
0
50
100
150
200
0.01
0.05
0.10
0.50
1.00
5.00
10.00
Treatment Dose (uM)

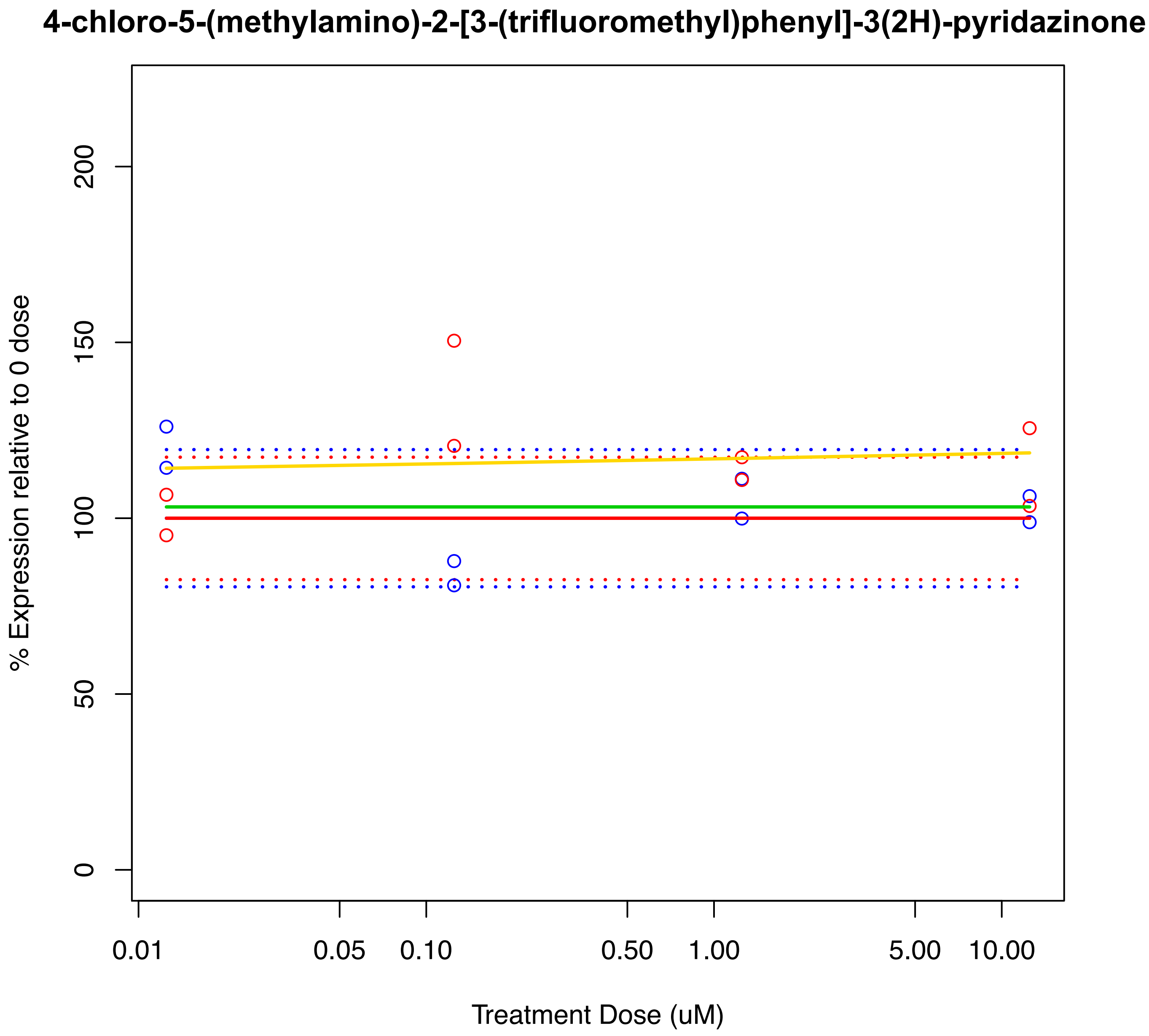
4-chloro-5-(methylamino)-2-[3-(trifluoromethyl)phenyl]-3(2H)-pyridazinone
% Expression relative to 0 dose
0
50
100
150
200
0.01
0.05
0.10
0.50
1.00
5.00
10.00
Treatment Dose (uM)

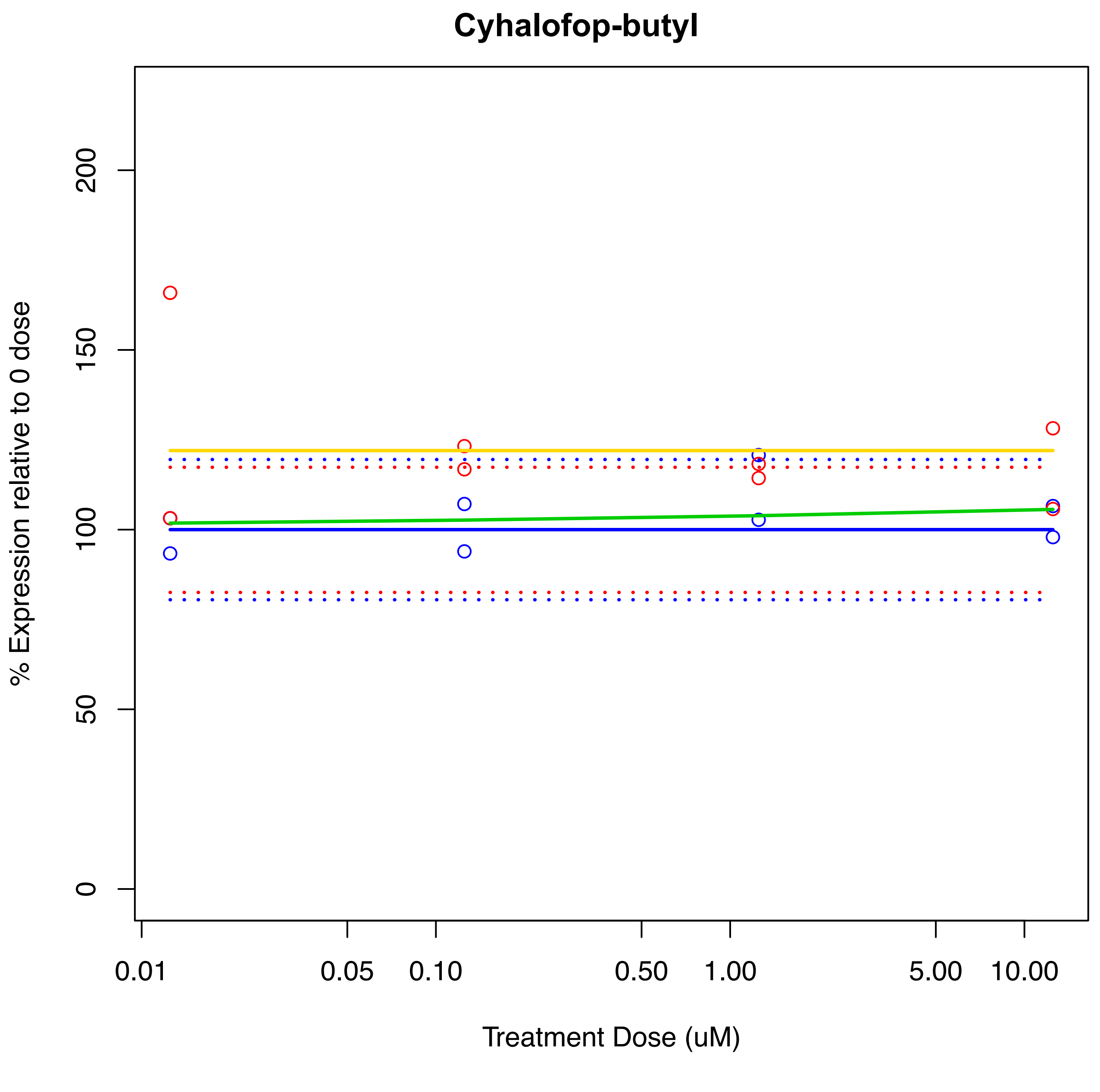

Cyhalofop-butyl
% Expression relative to 0 dose
0
50
100
150
200
0.01
0.05
0.10
0.50
1.00
5.00
10.00
Treatment Dose (uM)

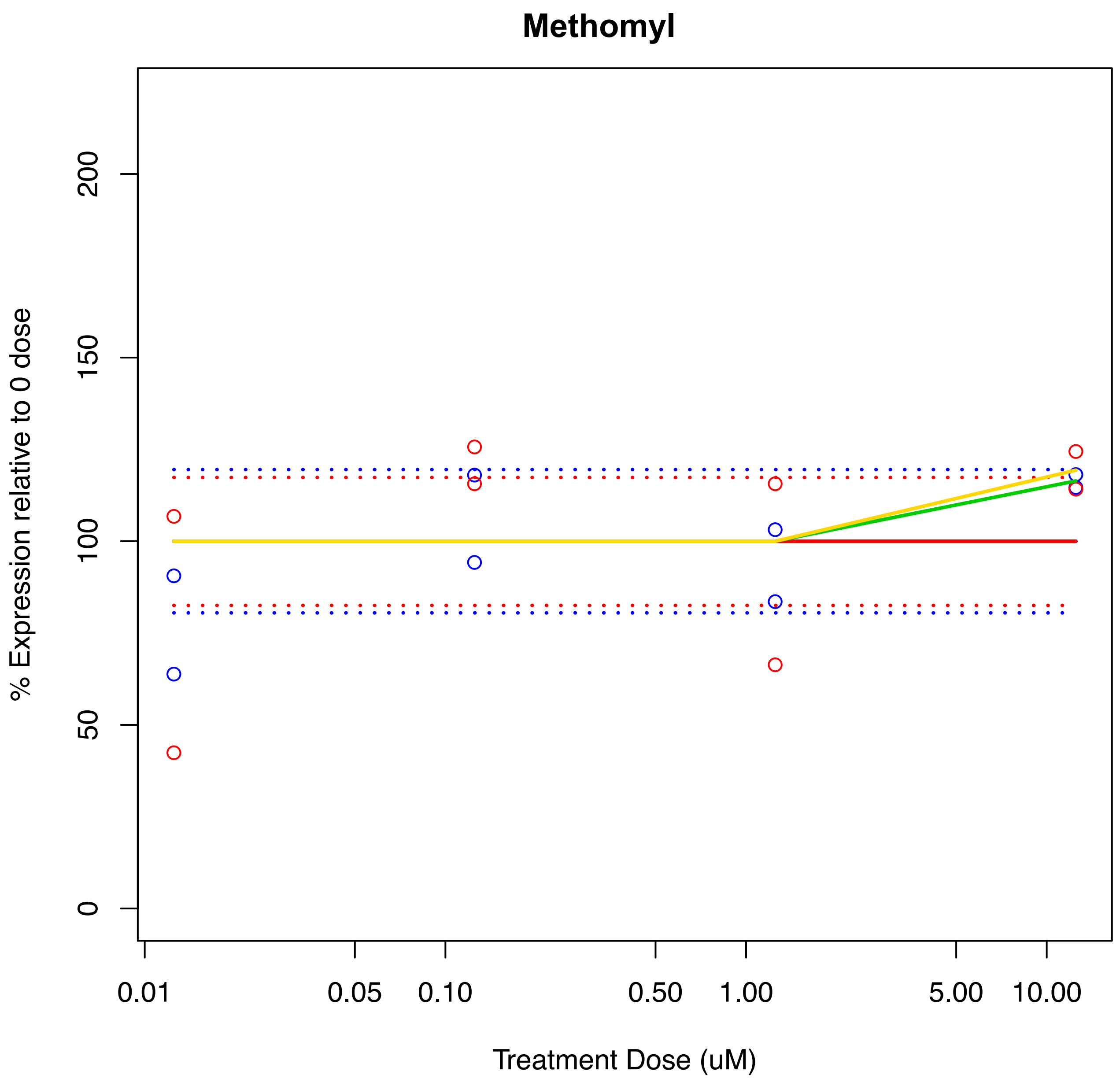

Methomyl
% Expression relative to 0 dose
0
50
100
150
200
0.01
0.05
0.10
0.50
1.00
5.00
10.00
Treatment Dose (uM)

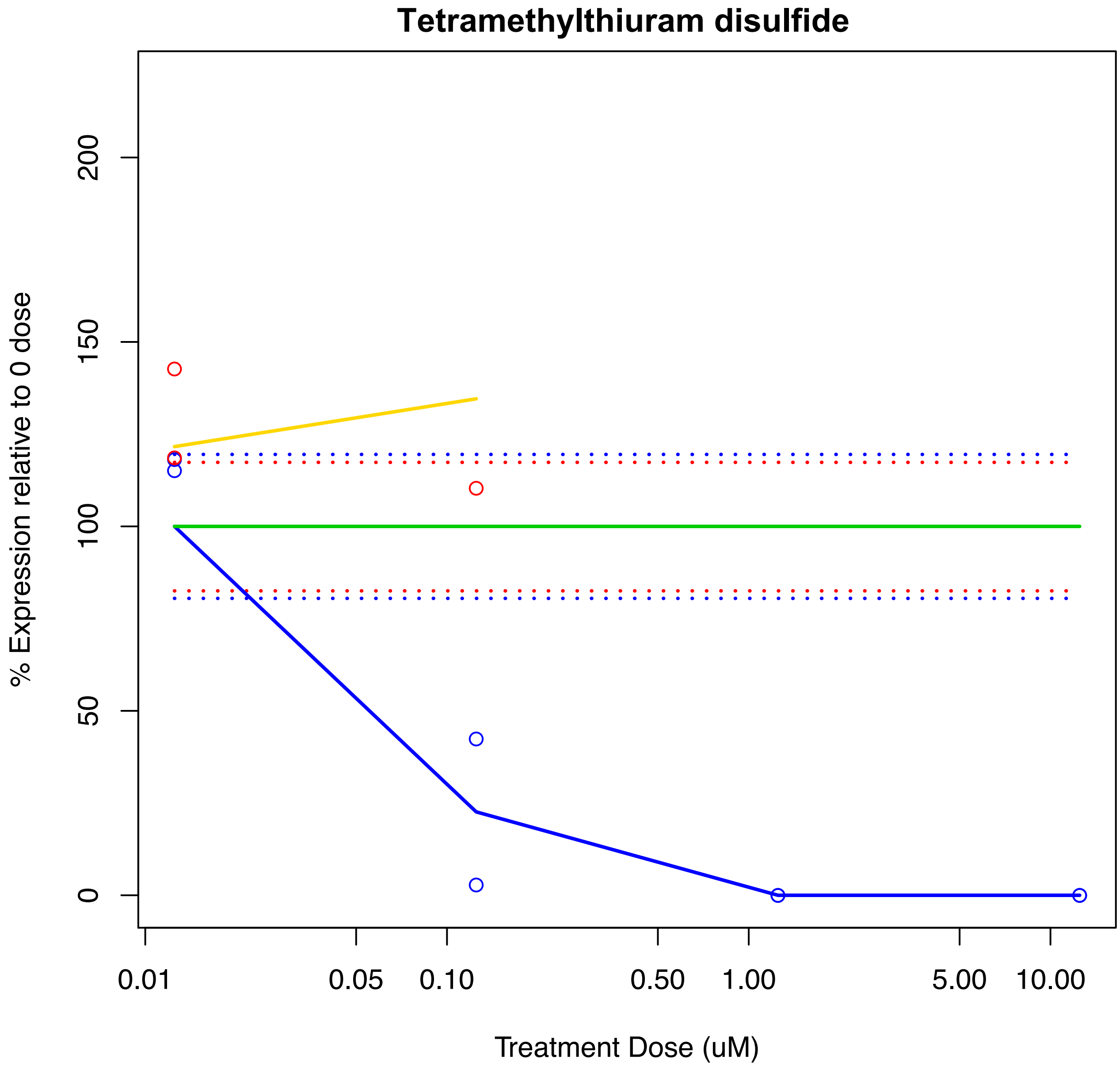
Tetramethylthiuram disulfide
% Expression relative to 0 dose
0
50
100
150
200
0.01
0.05
0.10
0.50
1.00
5.00
10.00
Treatment Dose (uM)

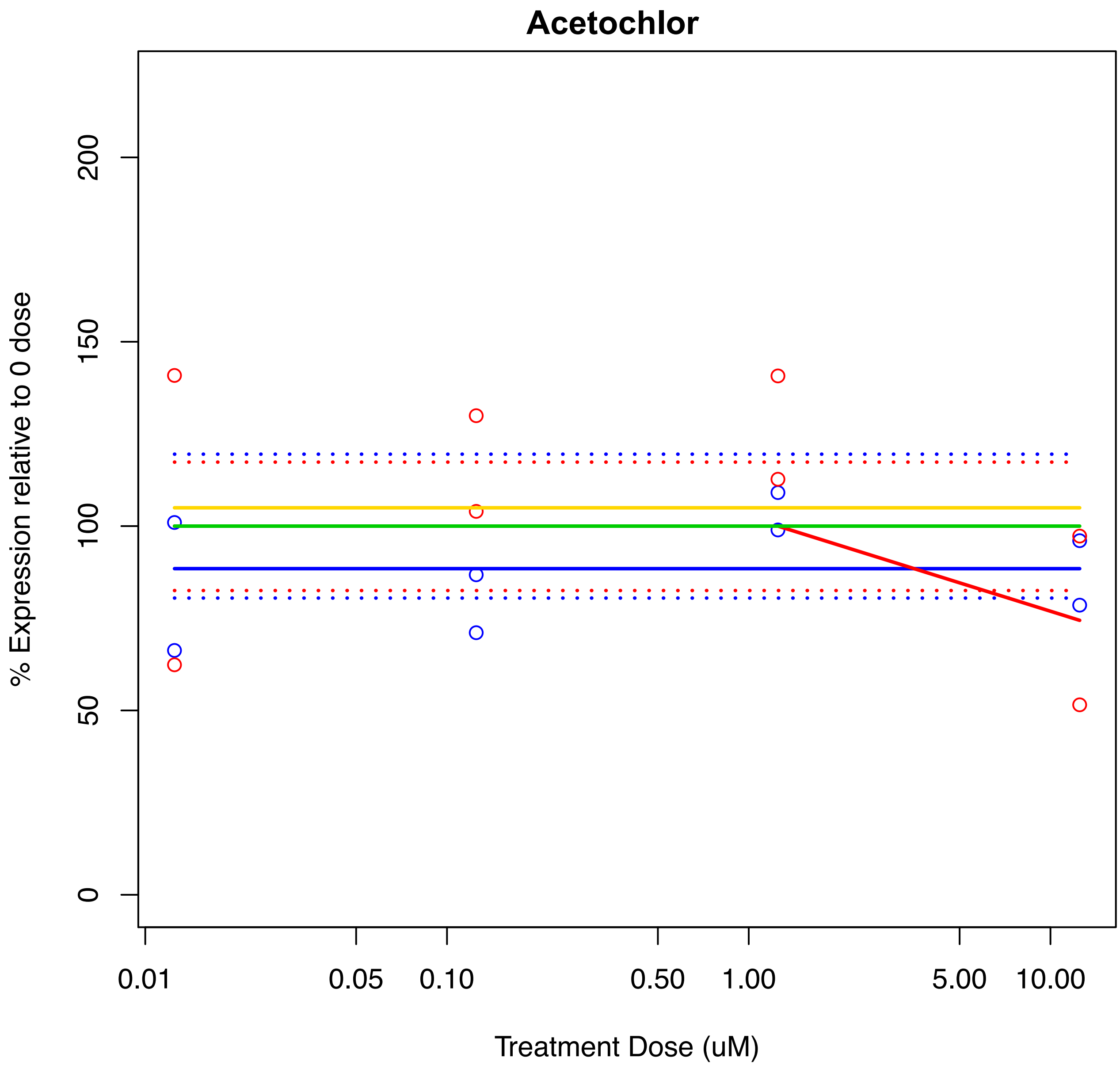
Acetochlor
% Expression relative to 0 dose
0
50
100
150
200
0.01
0.05
0.10
0.50
1.00
5.00
10.00
Treatment Dose (uM)

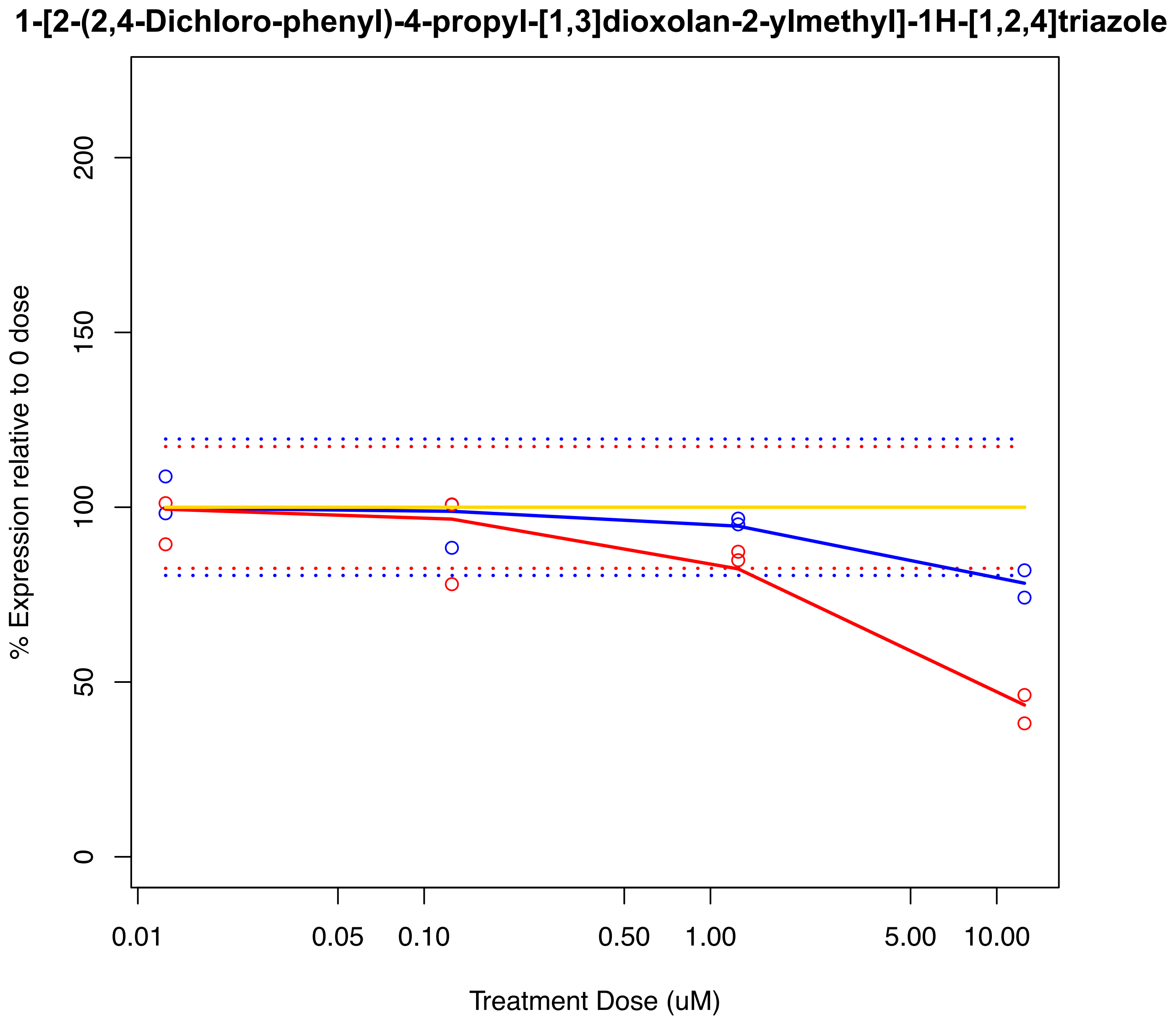
1-[2-(2,4-Dichloro-phenyl)-4-propyl-[1,3]dioxolan-2-ylmethyl]-1H-[1,2,4]triazole
% Expression relative to 0 dose
0
50
100
150
200
0.01
0.05
0.10
0.50
1.00
5.00
10.00
Treatment Dose (uM)

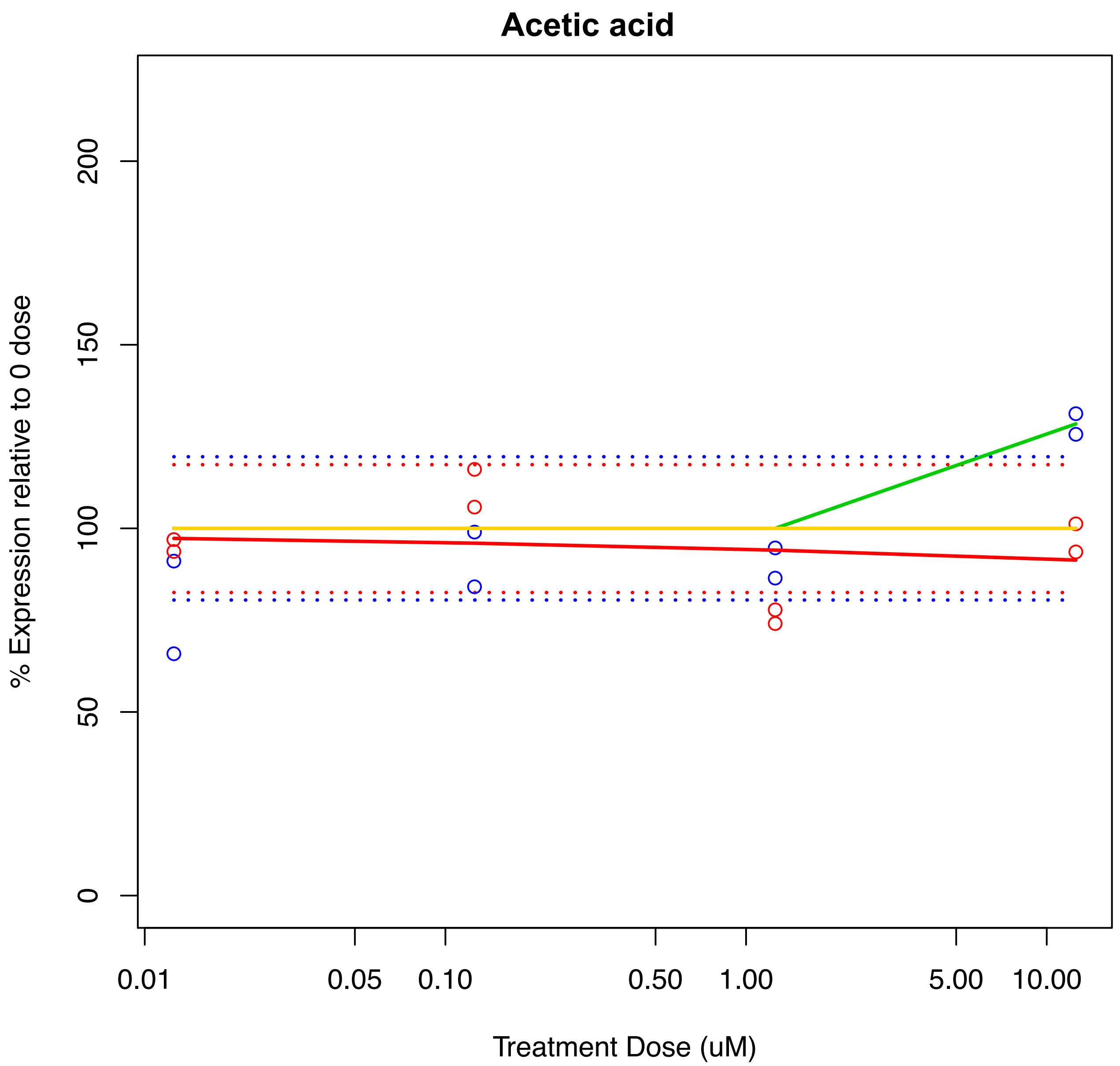

Acetic acid
% Expression relative to 0 dose
0
50
100
150
200
0.01
0.05
0.10
0.50
1.00
5.00
10.00
Treatment Dose (uM)

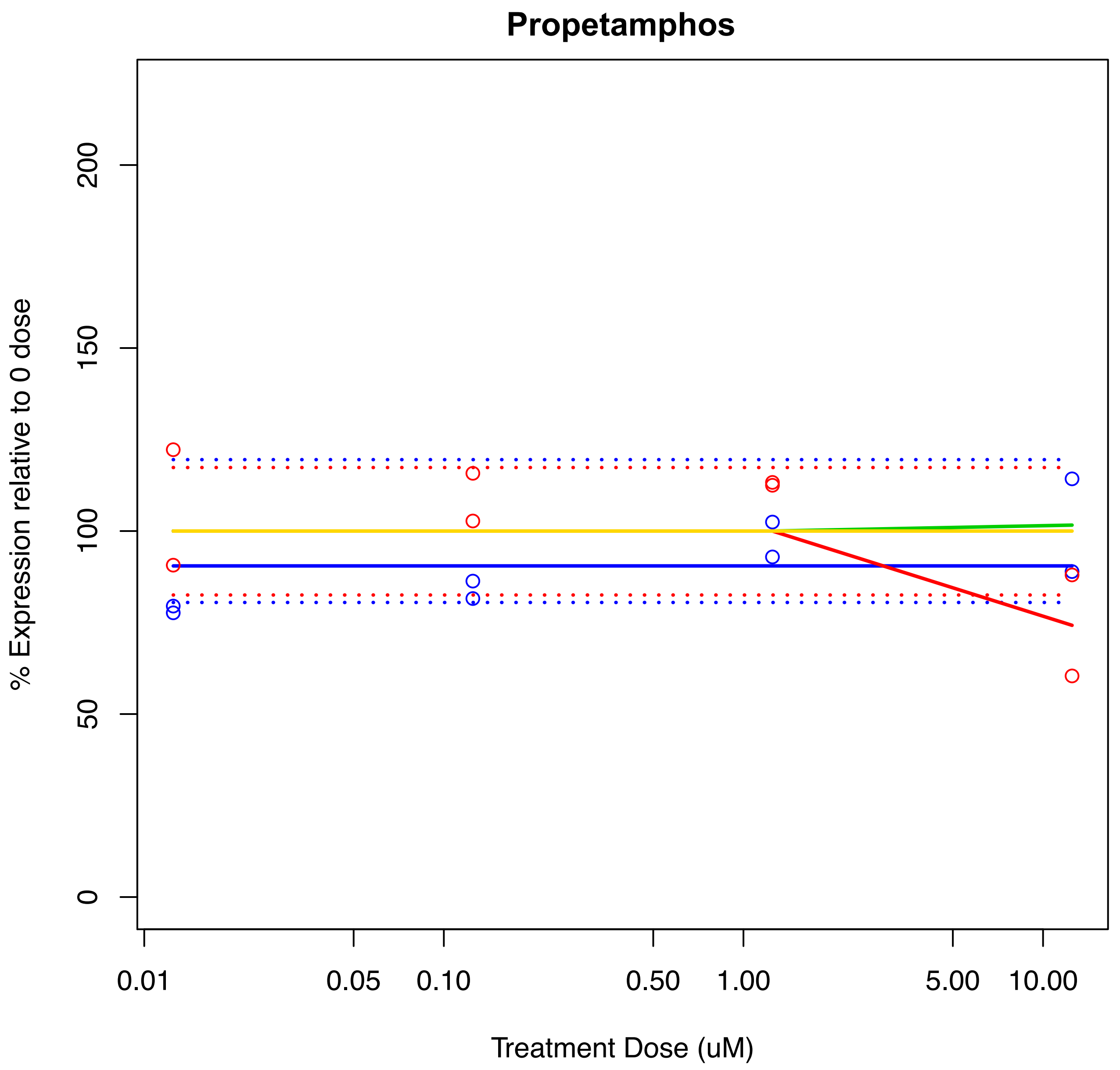

Propetamphos
% Expression relative to 0 dose
0
50
100
150
200
0.01
0.05
0.10
0.50
1.00
5.00
10.00
Treatment Dose (uM)

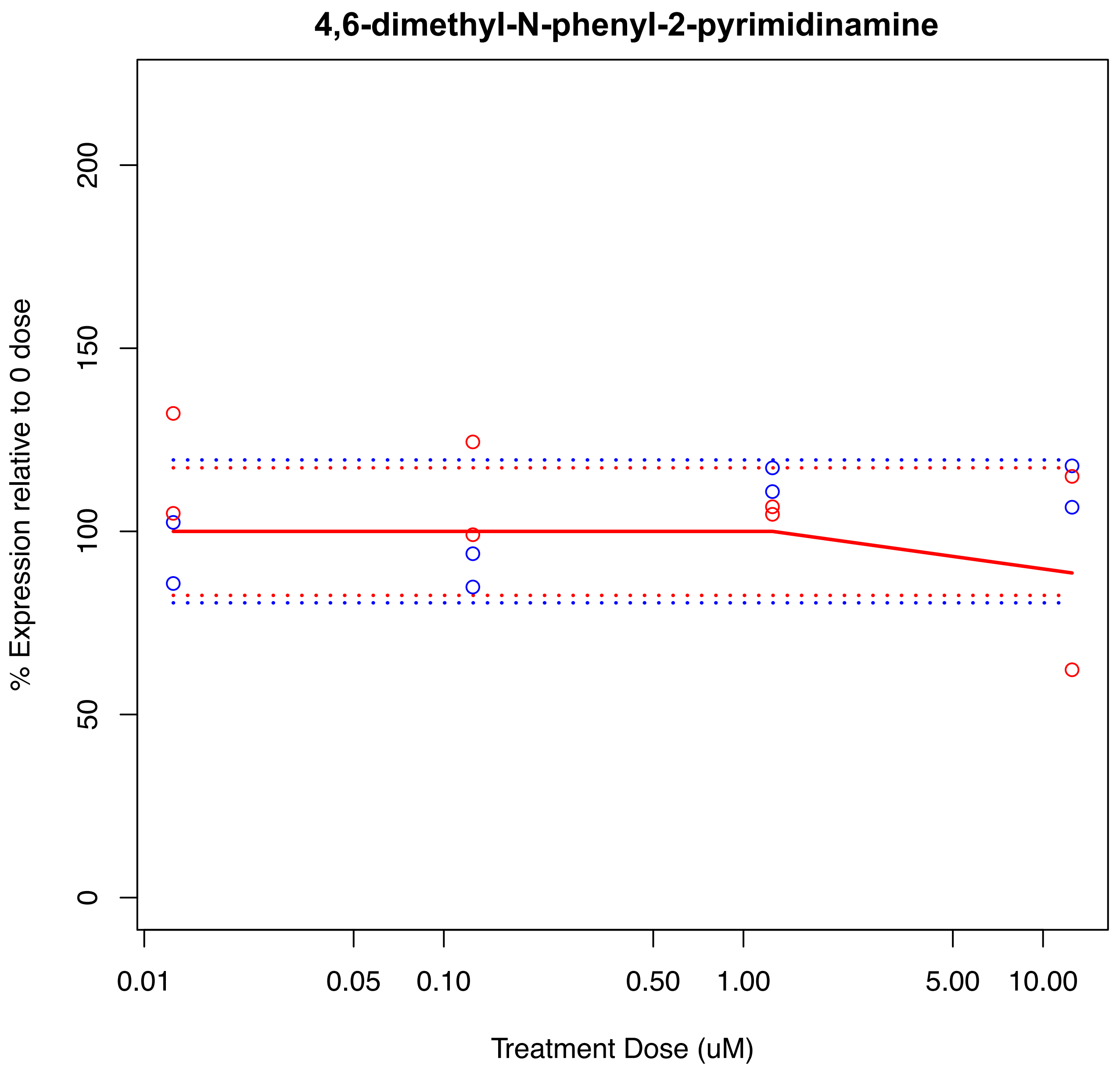
4,6-dimethyl-N-phenyl-2-pyrimidinamine
% Expression relative to 0 dose
0
50
100
150
200
0.01
0.05
0.10
0.50
1.00
5.00
10.00
Treatment Dose (uM)

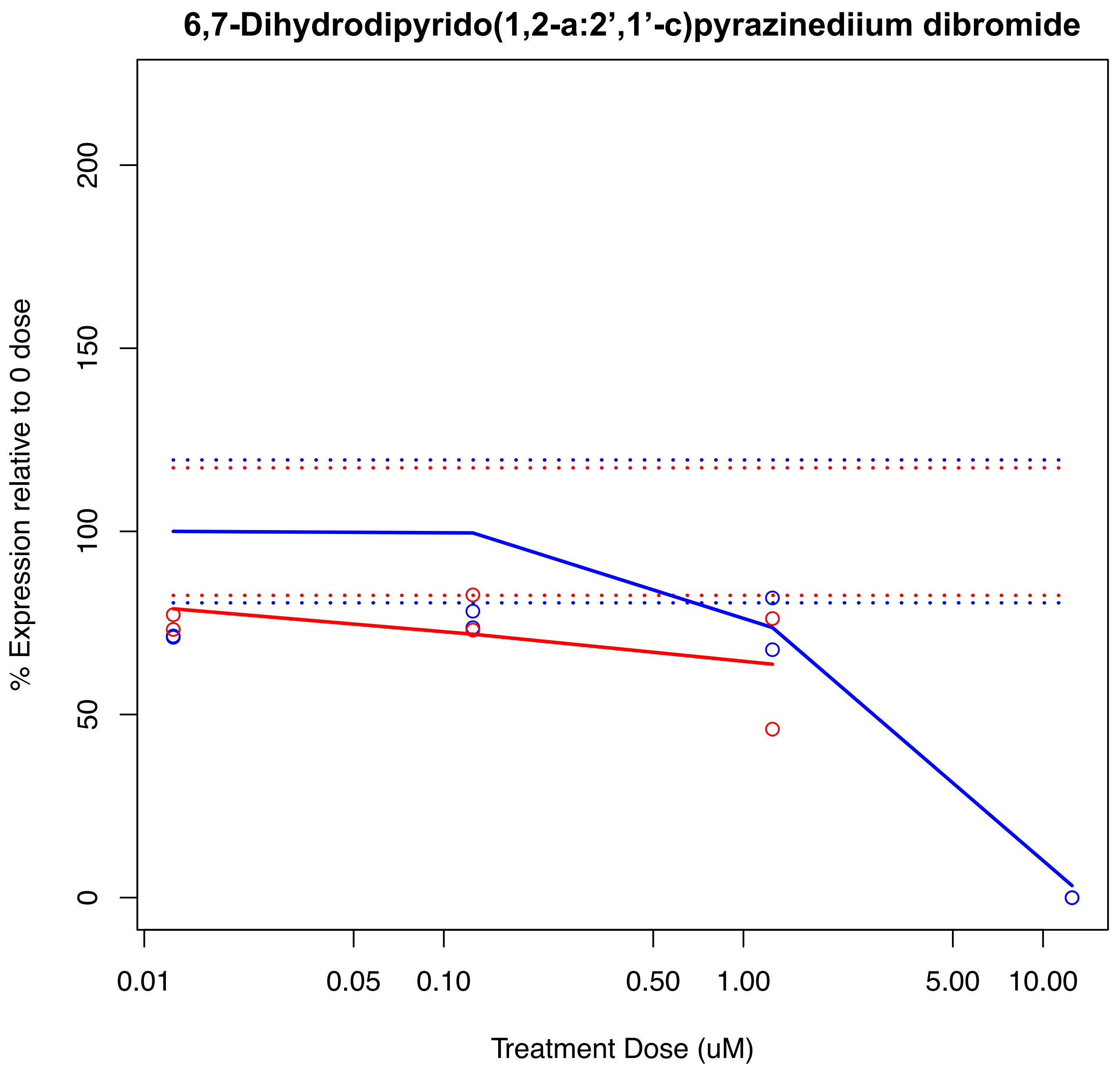
6,7-Dihydrodipyrido(1,2-a:2',1'-c)pyrazinediium dibromide
% Expression relative to 0 dose
0
50
100
150
200
0.01
0.05
0.10
0.50
1.00
5.00
10.00
Treatment Dose (uM)

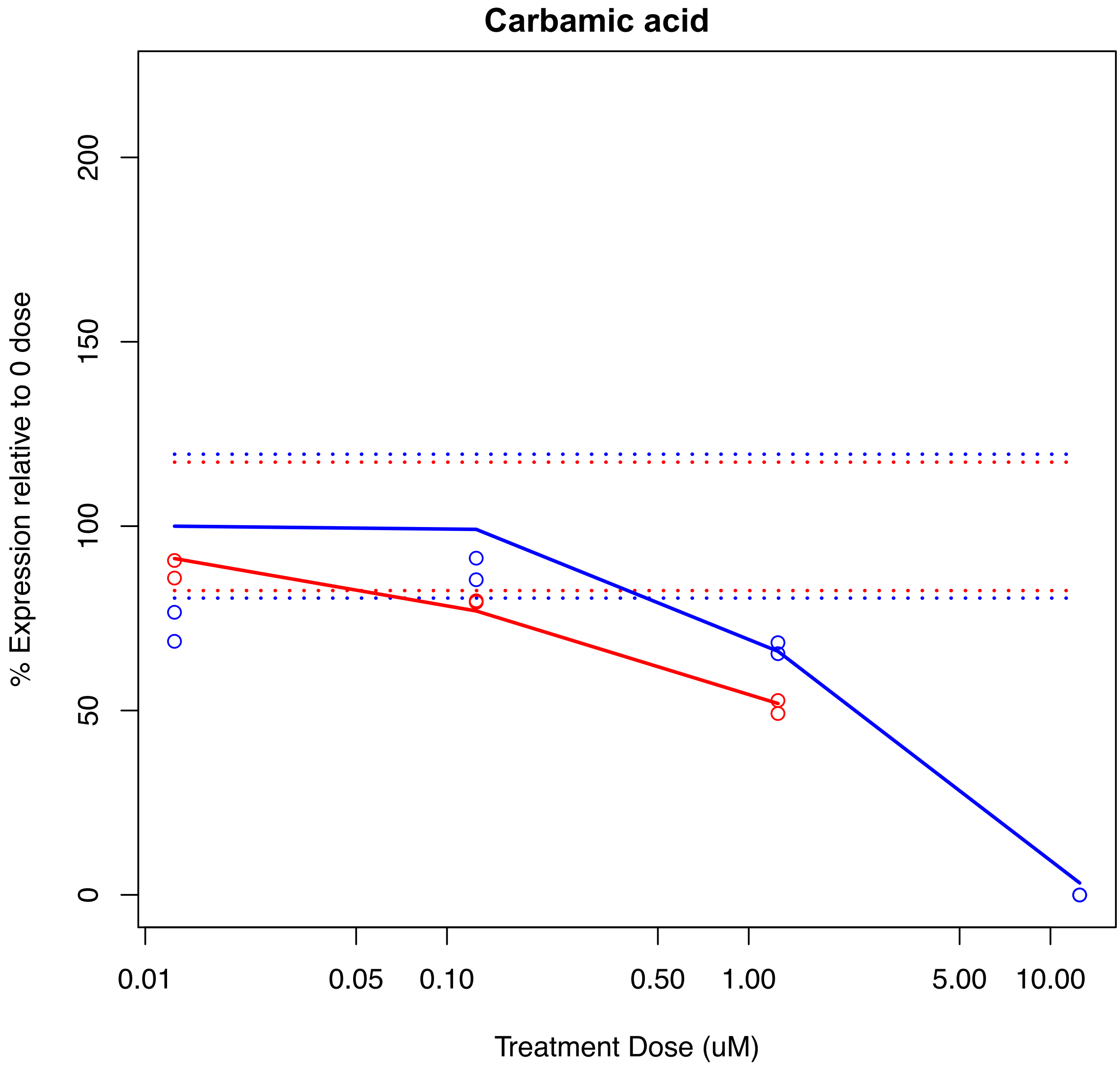
Carbamic acid
% Expression relative to 0 dose
0
50
100
150
200
0.01
0.05
0.10
0.50
1.00
5.00
10.00
Treatment Dose (uM)

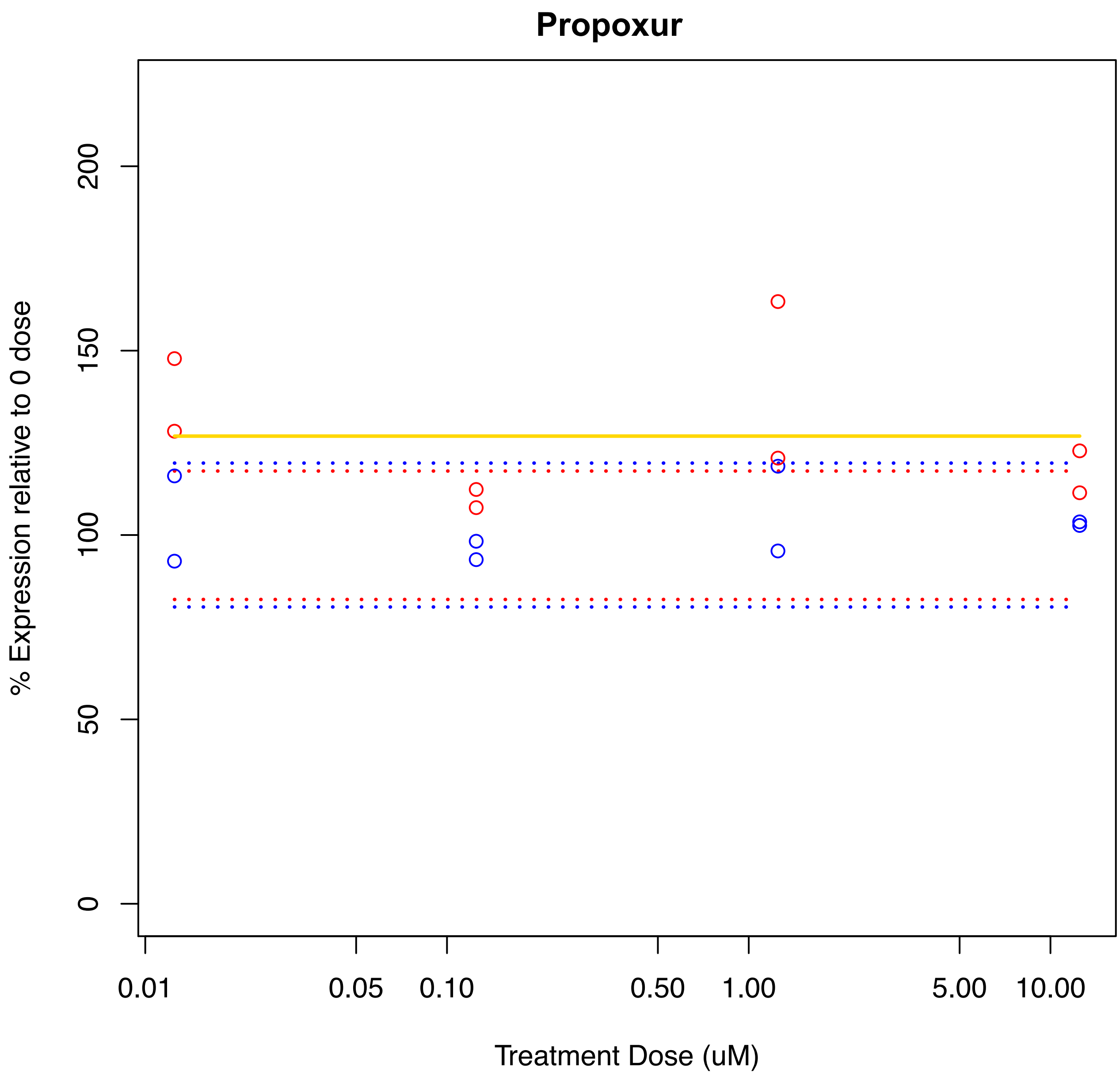

Propoxur
% Expression relative to 0 dose
0
50
100
150
200
0.01
0.05
0.10
0.50
1.00
5.00
10.00
Treatment Dose (uM)

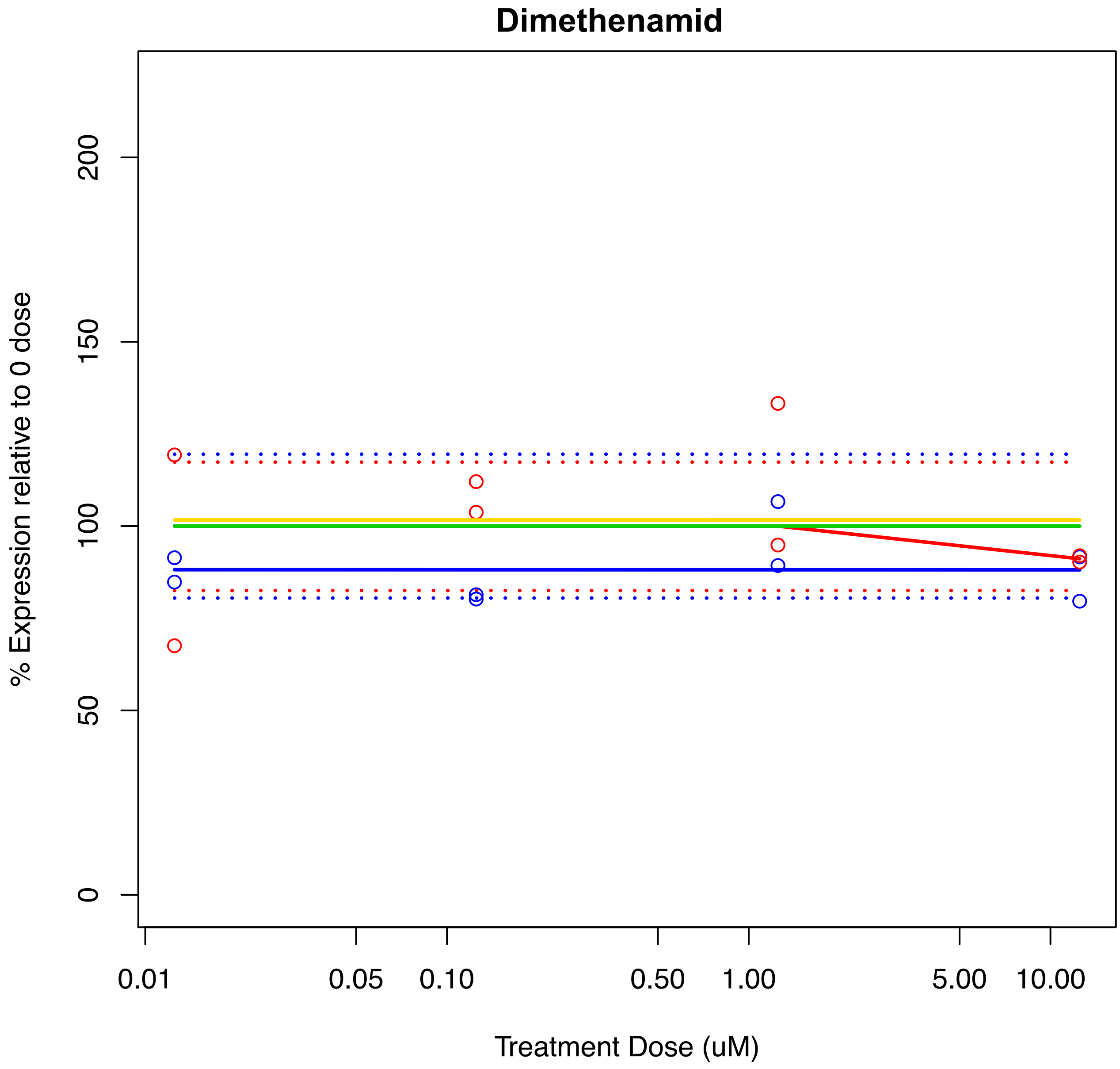

Dimethenamid
% Expression relative to 0 dose
0
50
100
150
200
0.01
0.05
0.10
0.50
1.00
5.00
10.00
Treatment Dose (uM)

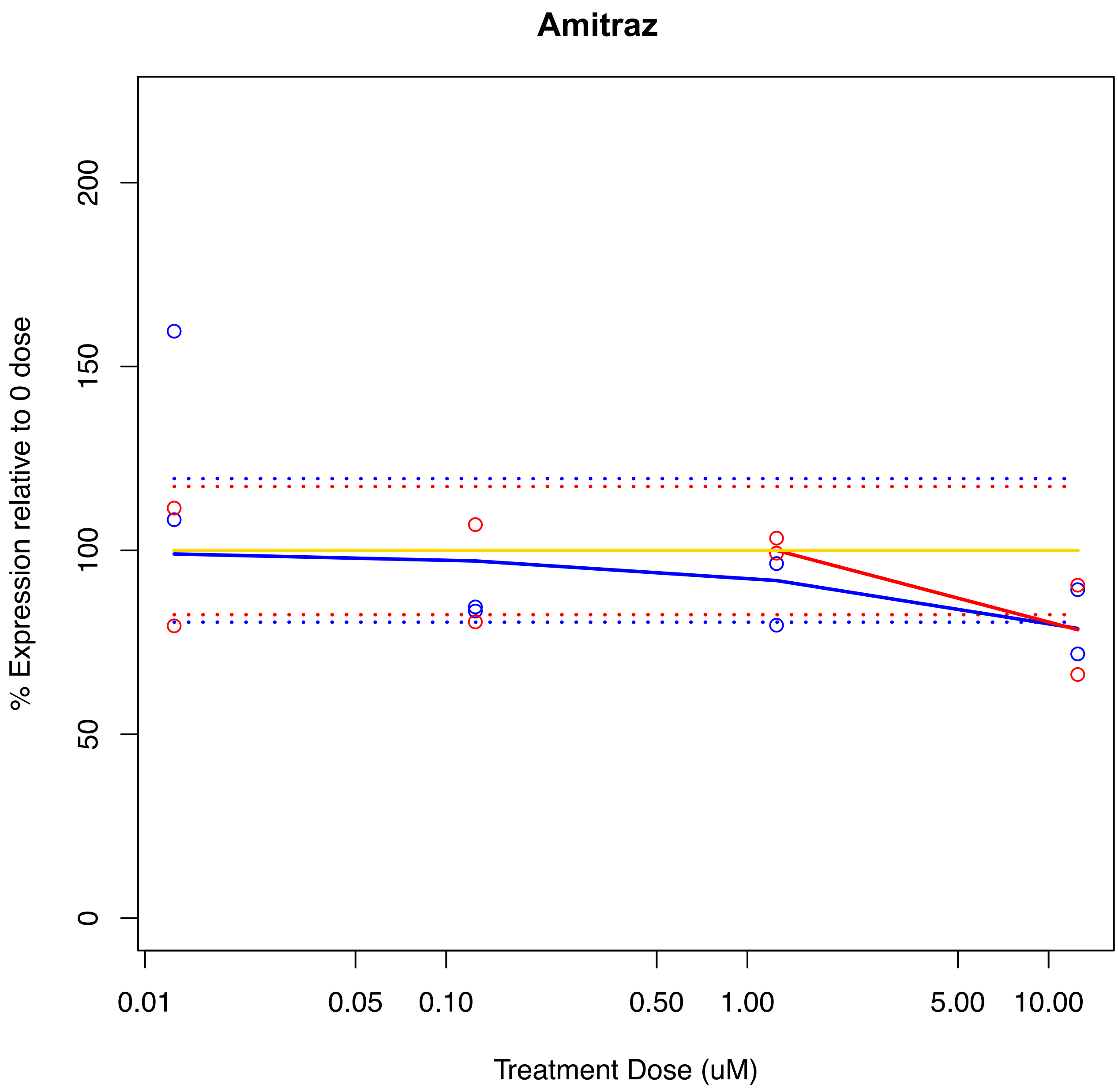

Amitraz
% Expression relative to 0 dose
0
50
100
150
200
0.01
0.05
0.10
0.50
1.00
5.00
10.00
Treatment Dose (uM)

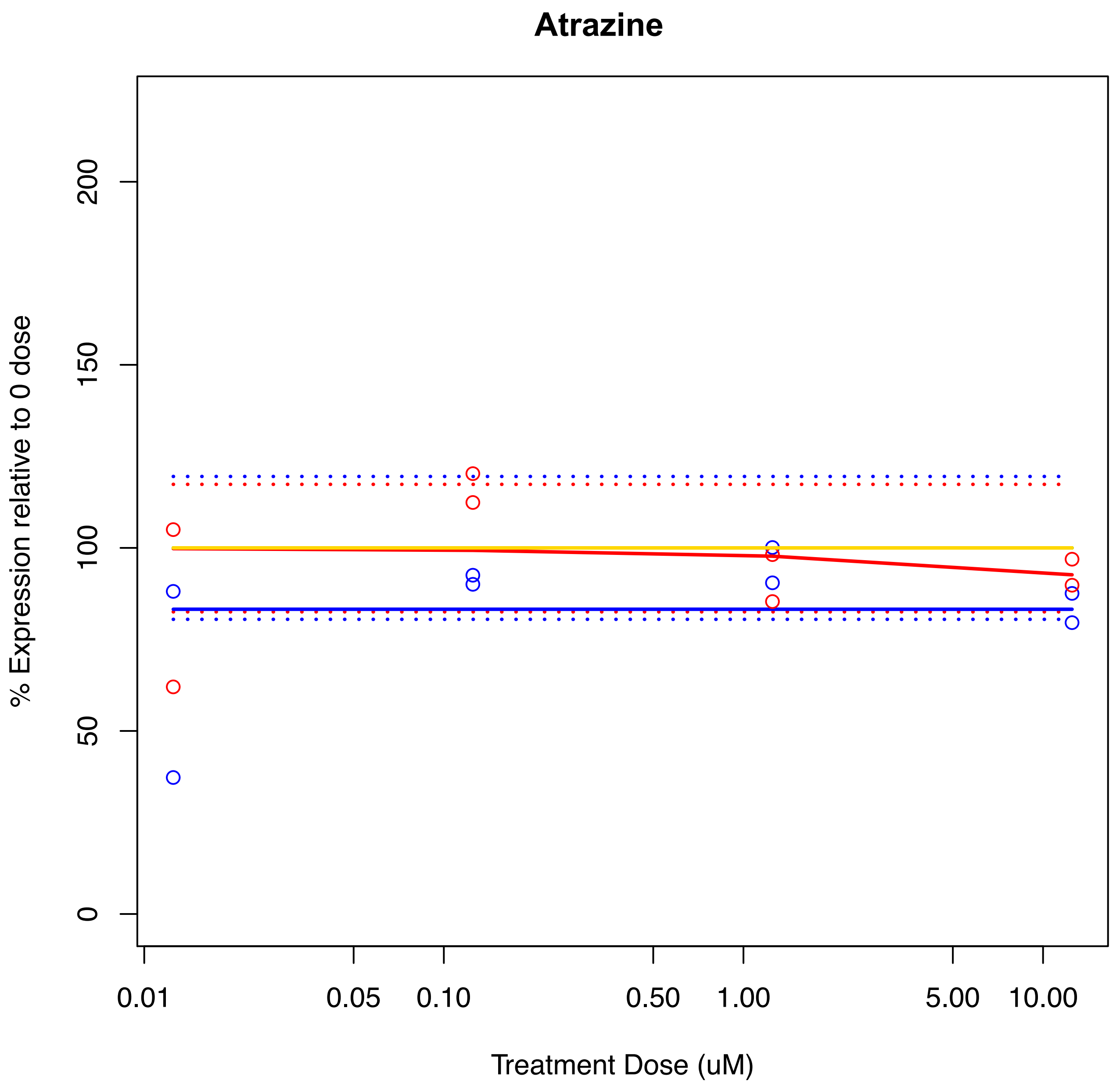

Atrazine
% Expression relative to 0 dose
0
50
100
150
200
0.01
0.05
0.10
0.50
1.00
5.00
10.00
Treatment Dose (uM)

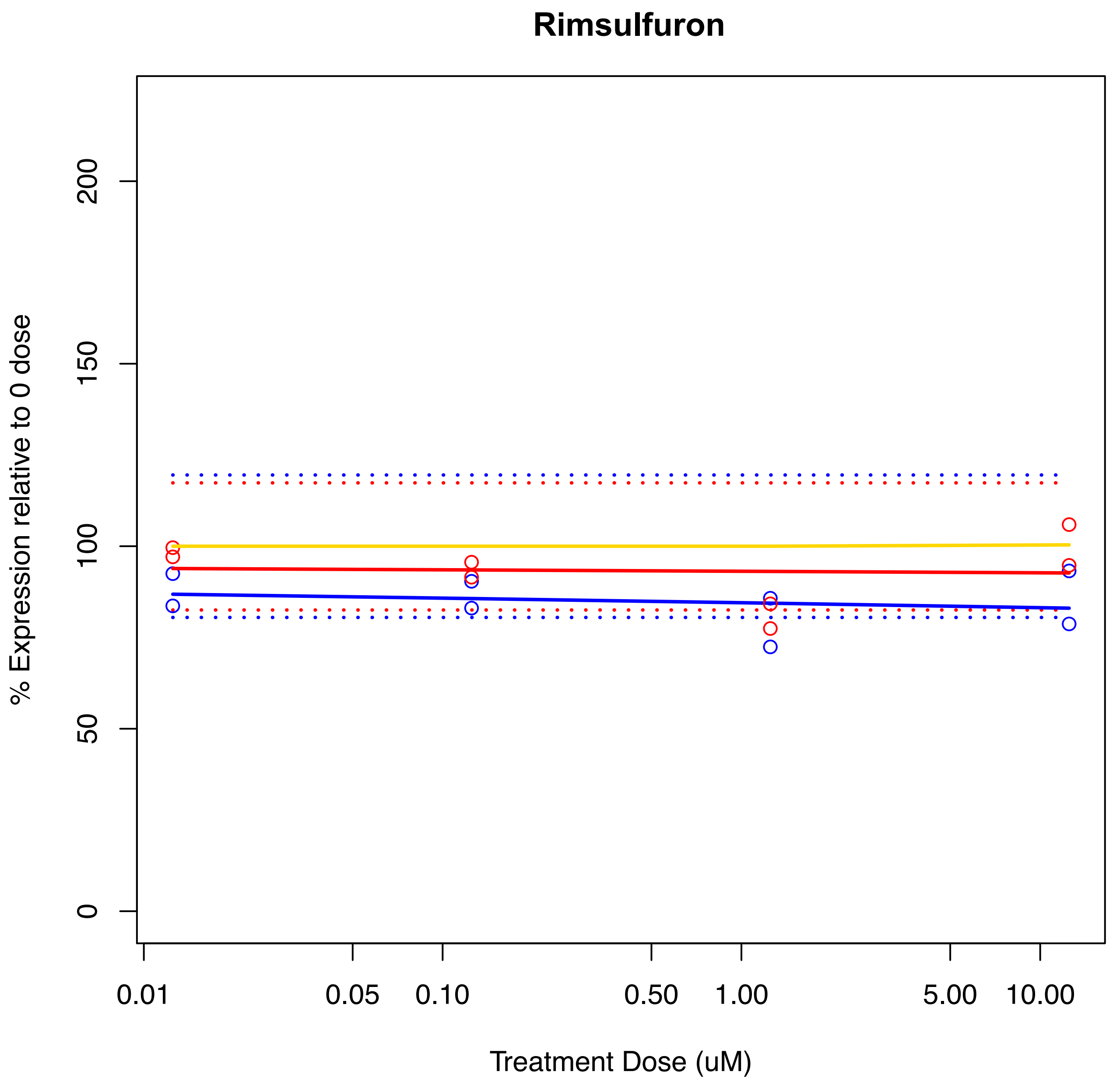

Rimsulfuron
% Expression relative to 0 dose
0
50
100
150
200
0.01
0.05
0.10
0.50
1.00
5.00
10.00
Treatment Dose (uM)

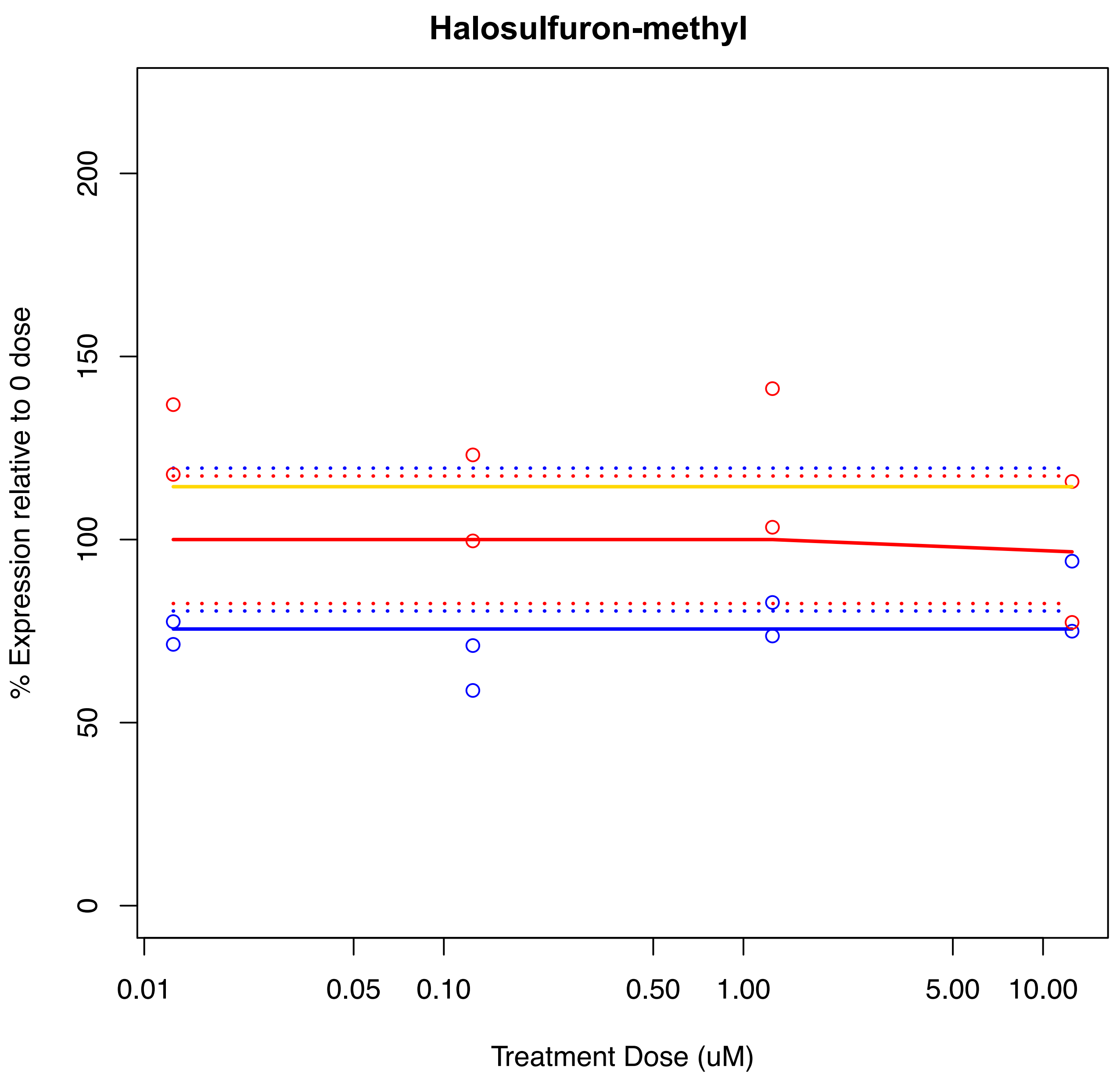
Halosulfuron-methyl
% Expression relative to 0 dose
200
150
100
50
0
0.01
0.05
0.10
0.50
1.00
5.00
10.00
Treatment Dose (uM)

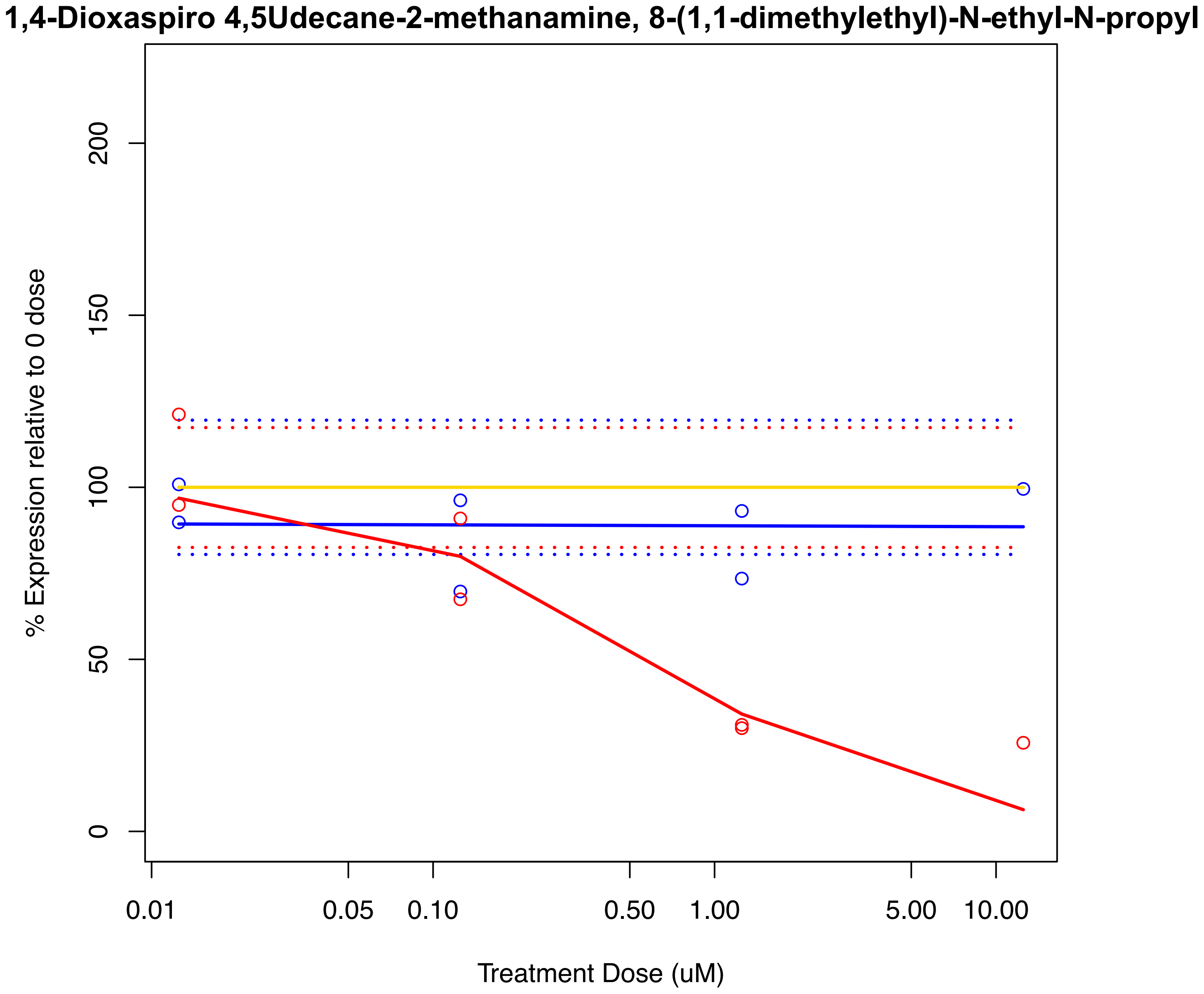

1,4-Dioxaspiro 4,5Udecane-2-methanamine, 8-(1,1-dimethylethyl)-N-ethyl-N-propyl
% Expression relative to 0 dose
0
50
100
150
200
0.01
0.05
0.10
0.50
1.00
5.00
10.00
Treatment Dose (uM)

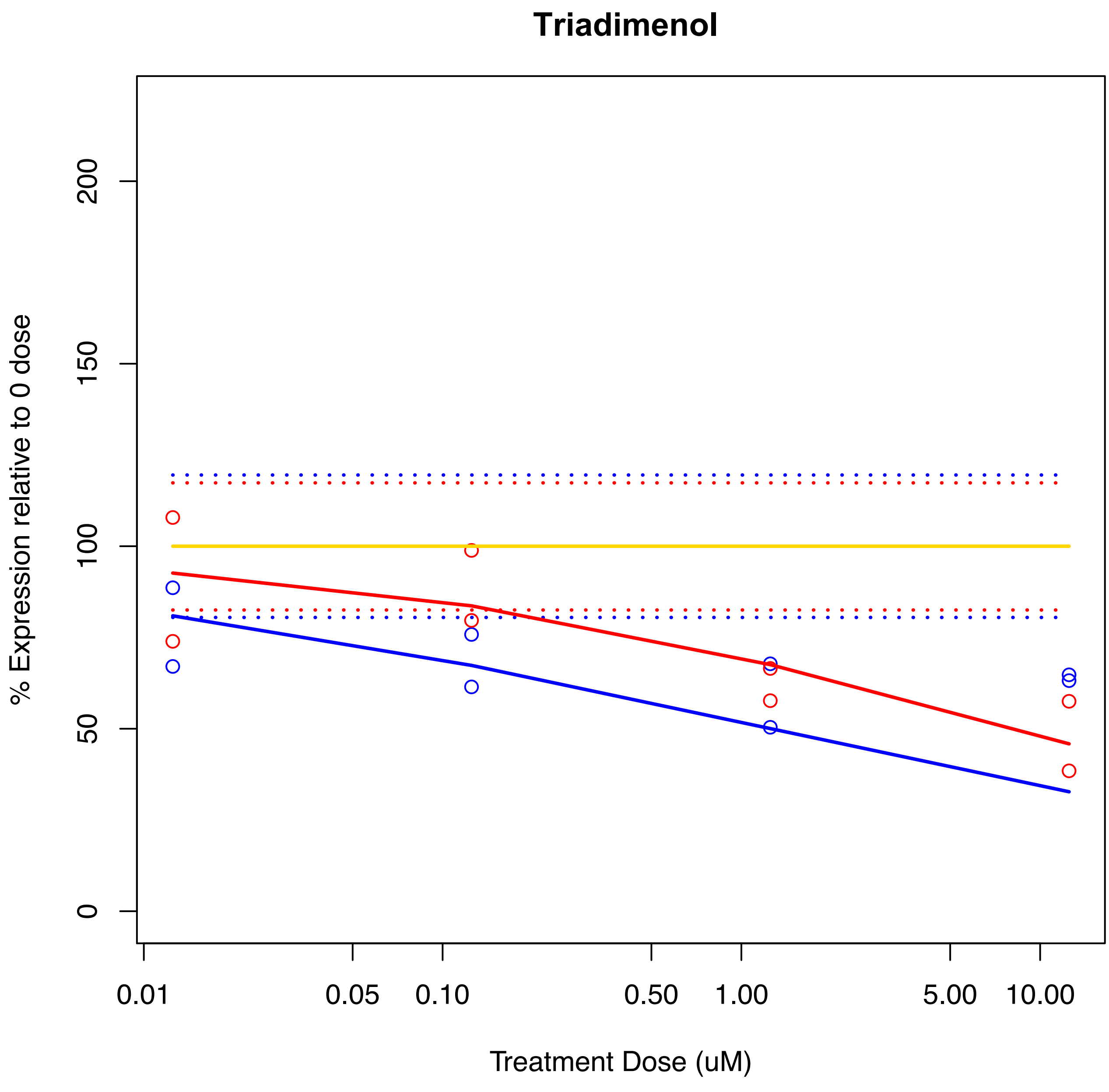
Triadimenol
% Expression relative to 0 dose
0
50
100
150
200
0.01
0.05
0.10
0.50
1.00
5.00
10.00
Treatment Dose (uM)

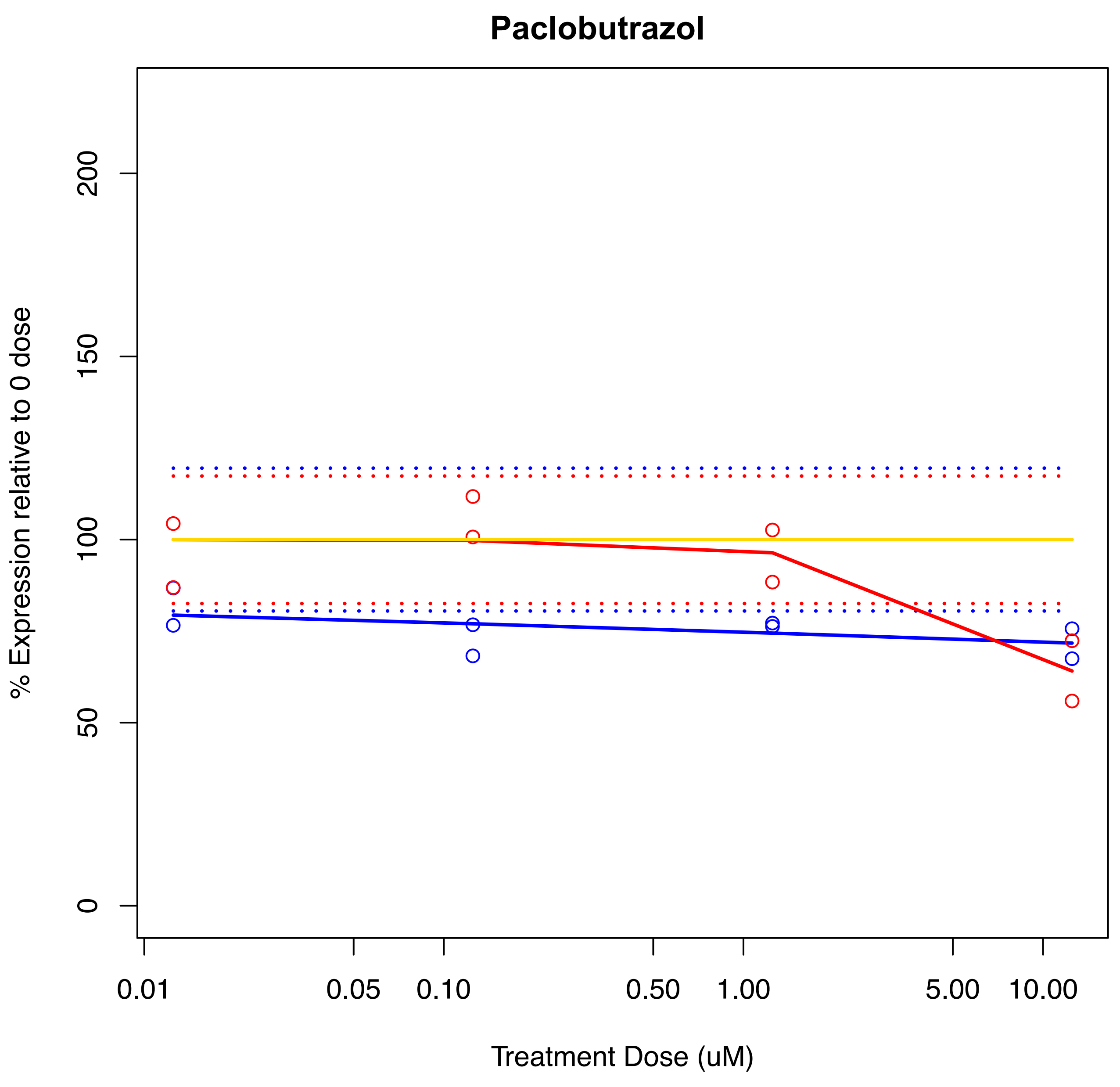
Paclobutrazol
% Expression relative to 0 dose
0
50
100
150
200
0.01
0.05
0.10
0.50
1.00
5.00
10.00
Treatment Dose (uM)

Bifenazate

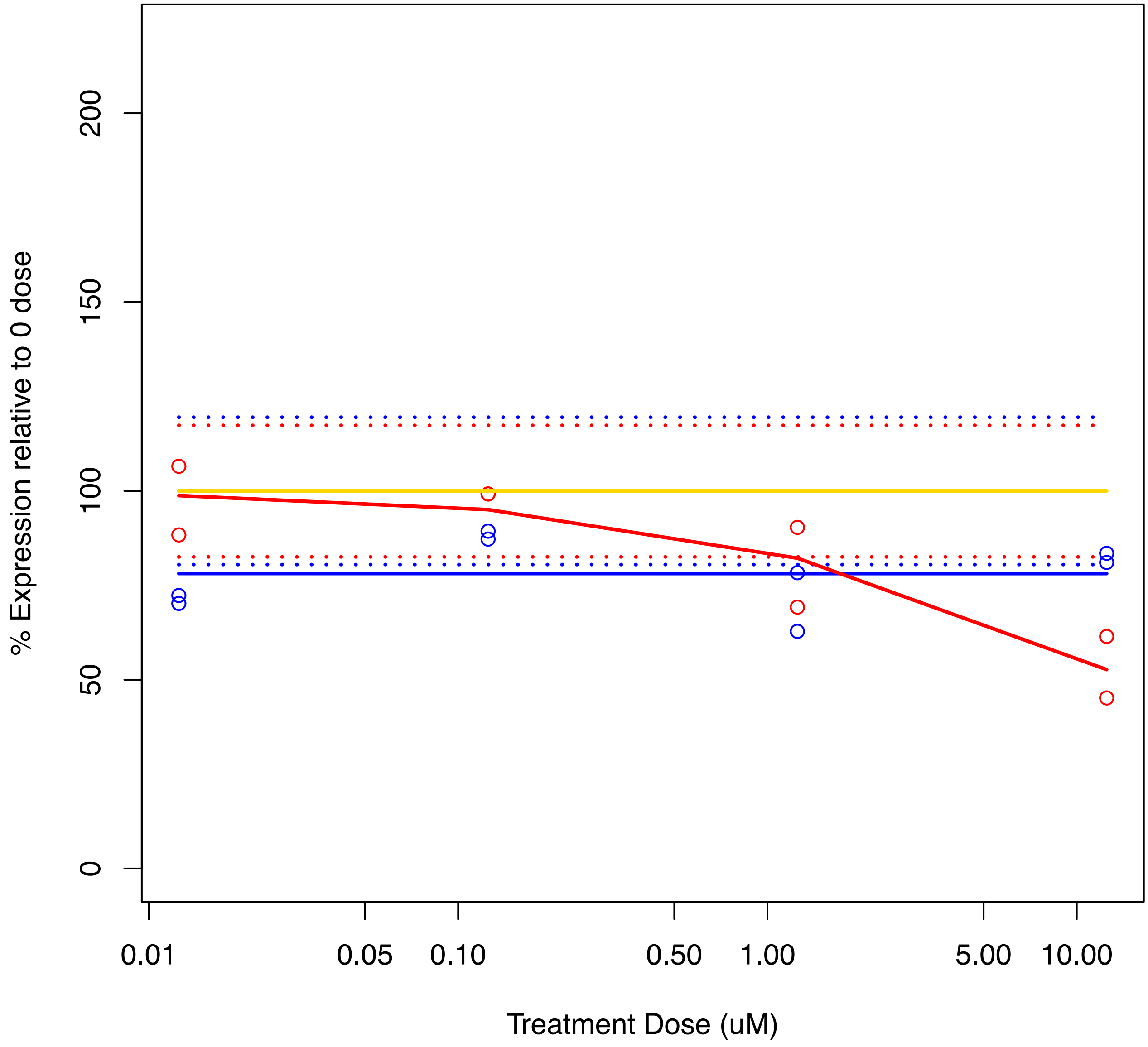

% Expression relative to 0 dose
0
50
100
150
200
0.01
0.05
0.10
0.50
1.00
5.00
10.00
Treatment Dose (uM)

1-Piperidinecarboxylic acid, 2-(2-hydroxyethyl)-, 1-methylpropyl ester

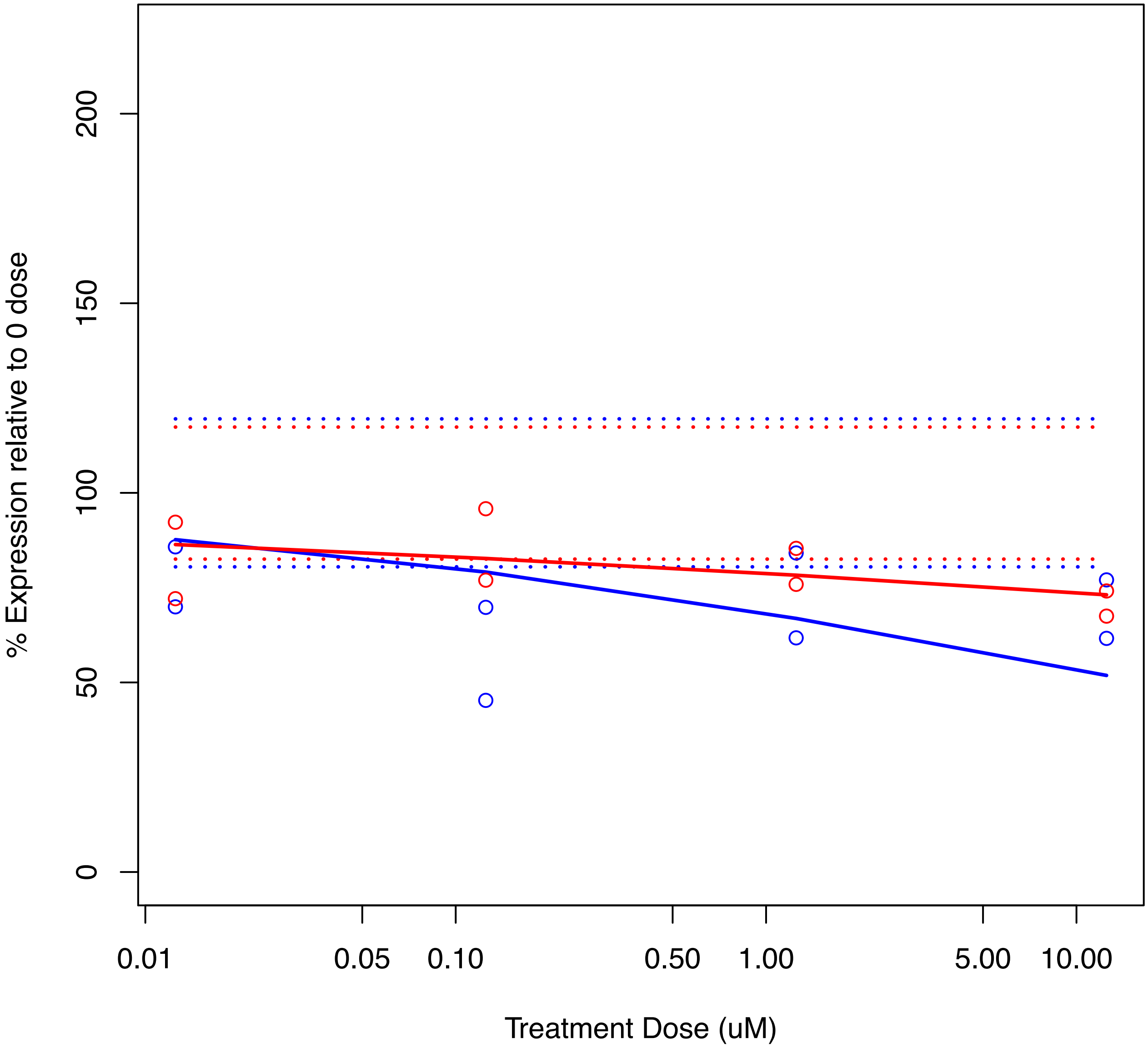

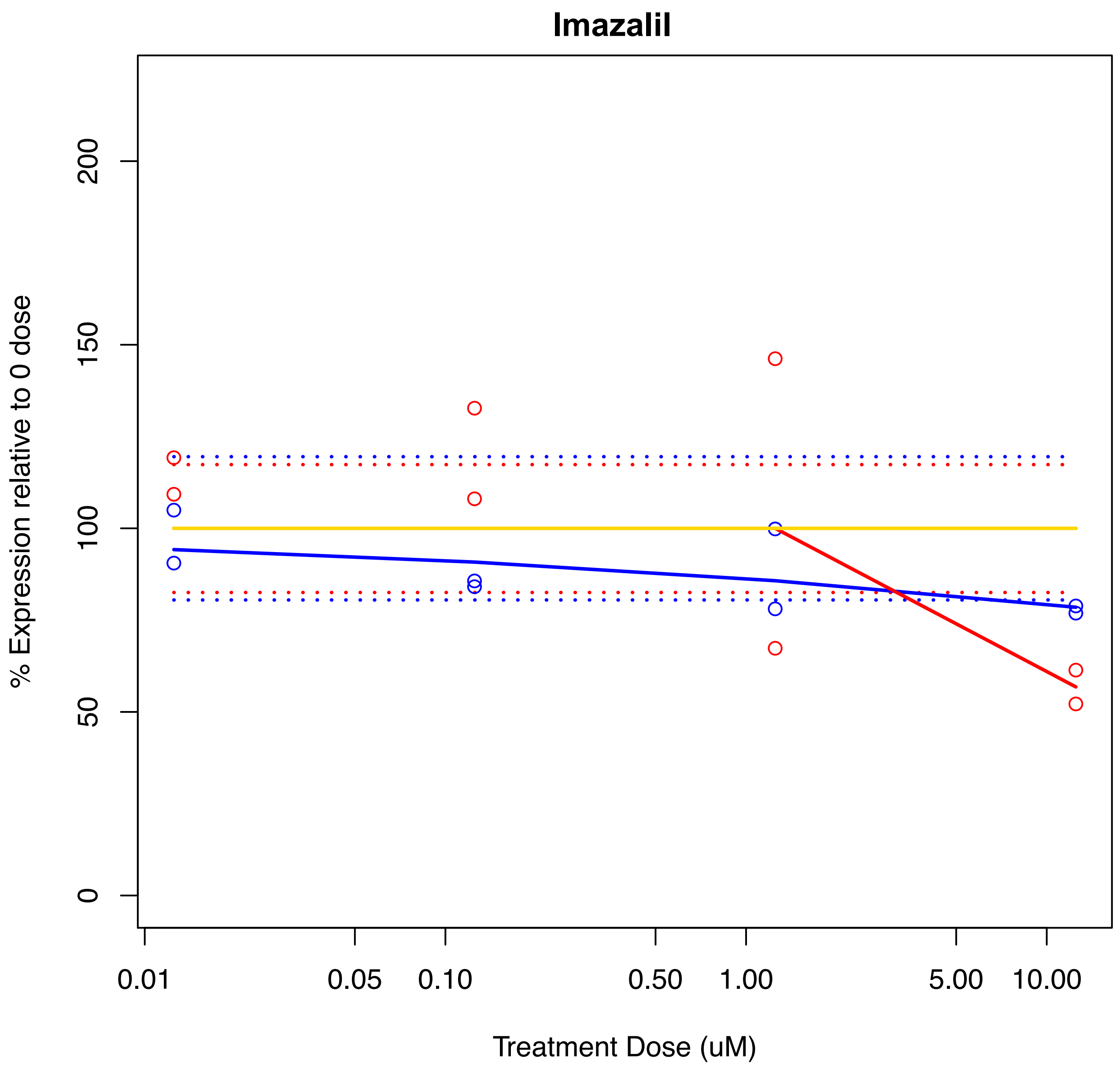
Imazalil
% Expression relative to 0 dose
0
50
100
150
200
0.01
0.05
0.10
0.50
1.00
5.00
10.00
Treatment Dose (uM)

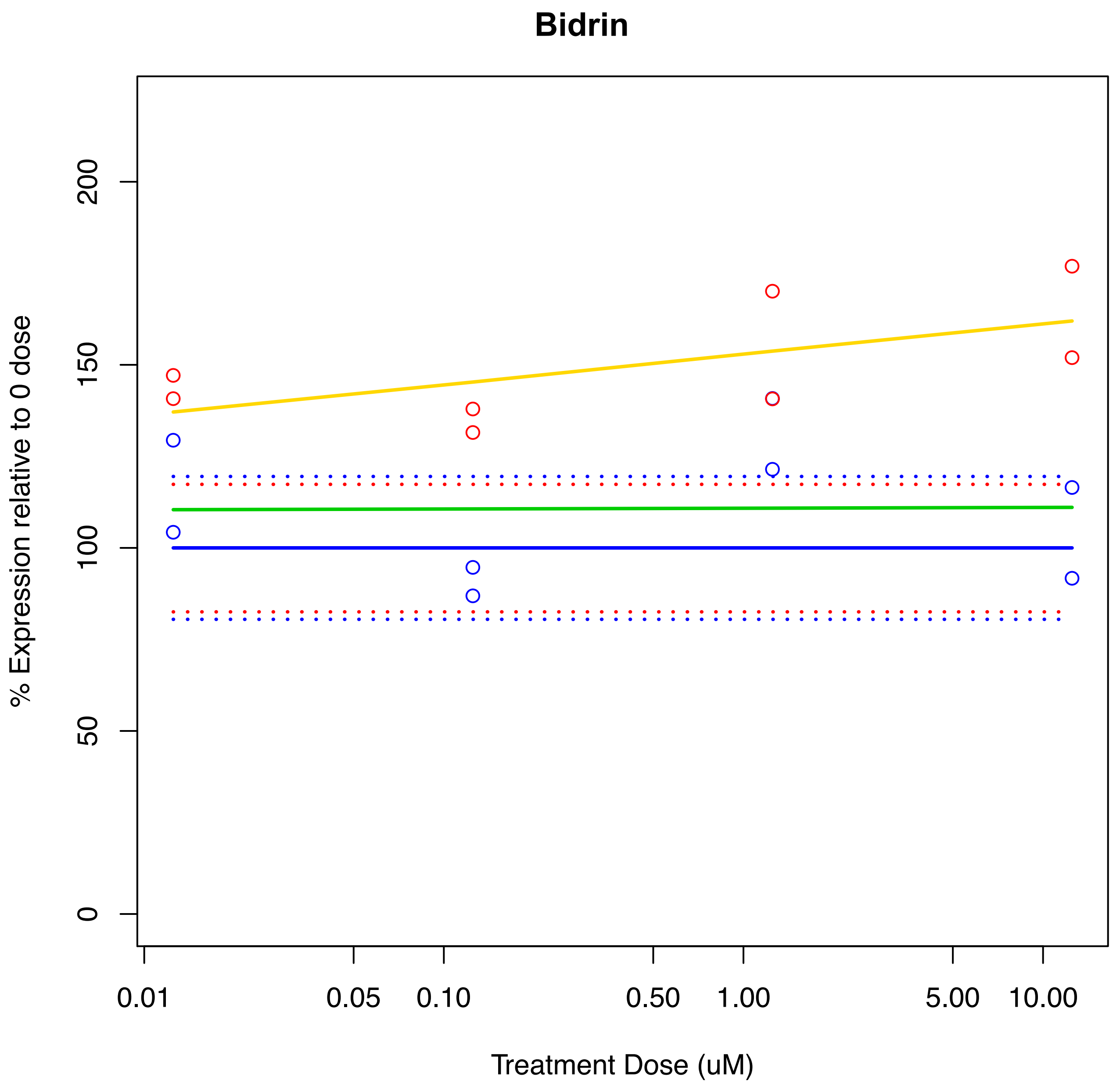
Bidrin
% Expression relative to 0 dose
0
50
100
150
200
0.01
0.05
0.10
0.50
1.00
5.00
10.00
Treatment Dose (uM)

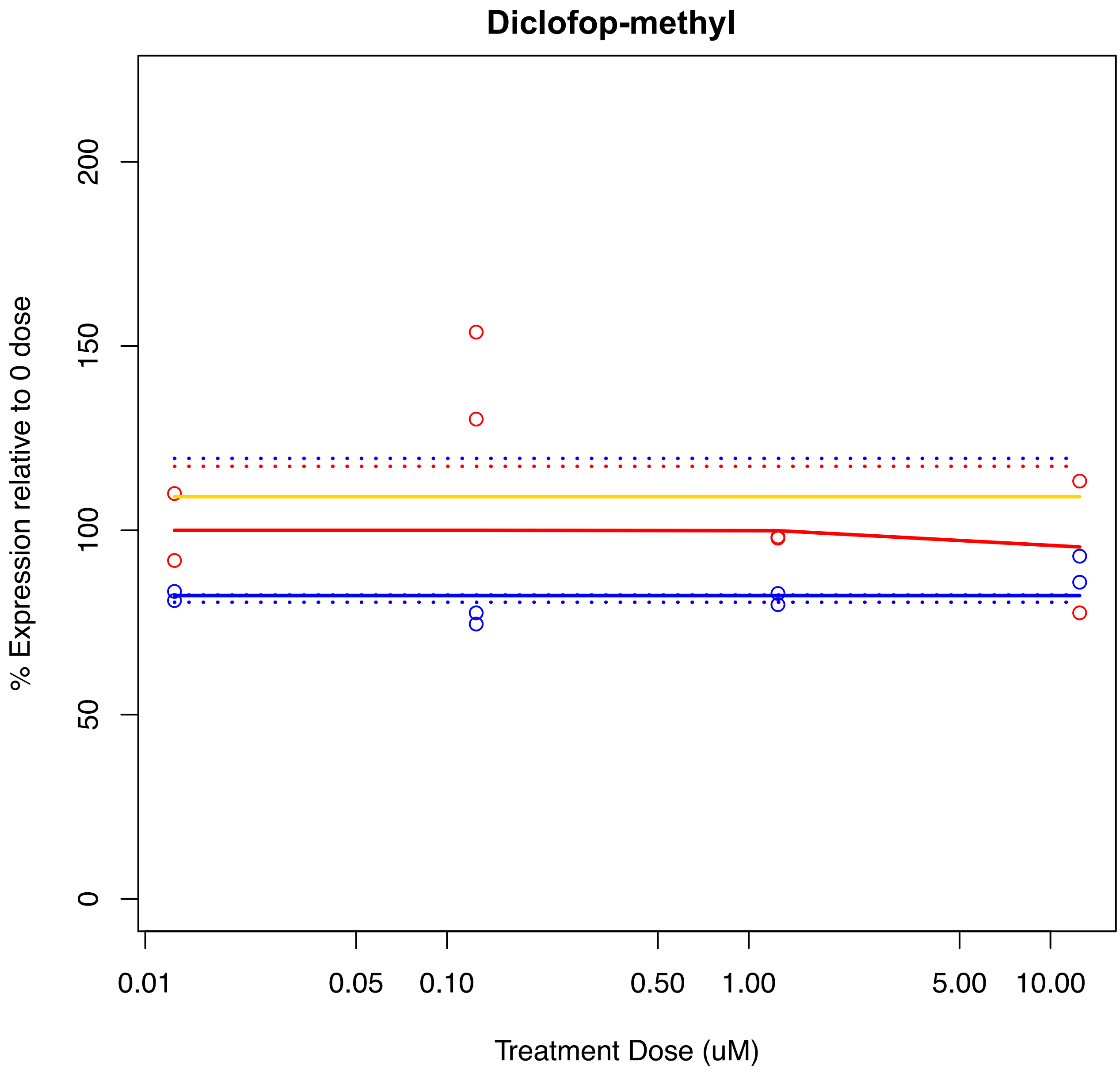

Diclofop-methyl
% Expression relative to 0 dose
0
50
100
150
200
0.01
0.05
0.10
0.50
1.00
5.00
10.00
Treatment Dose (uM)

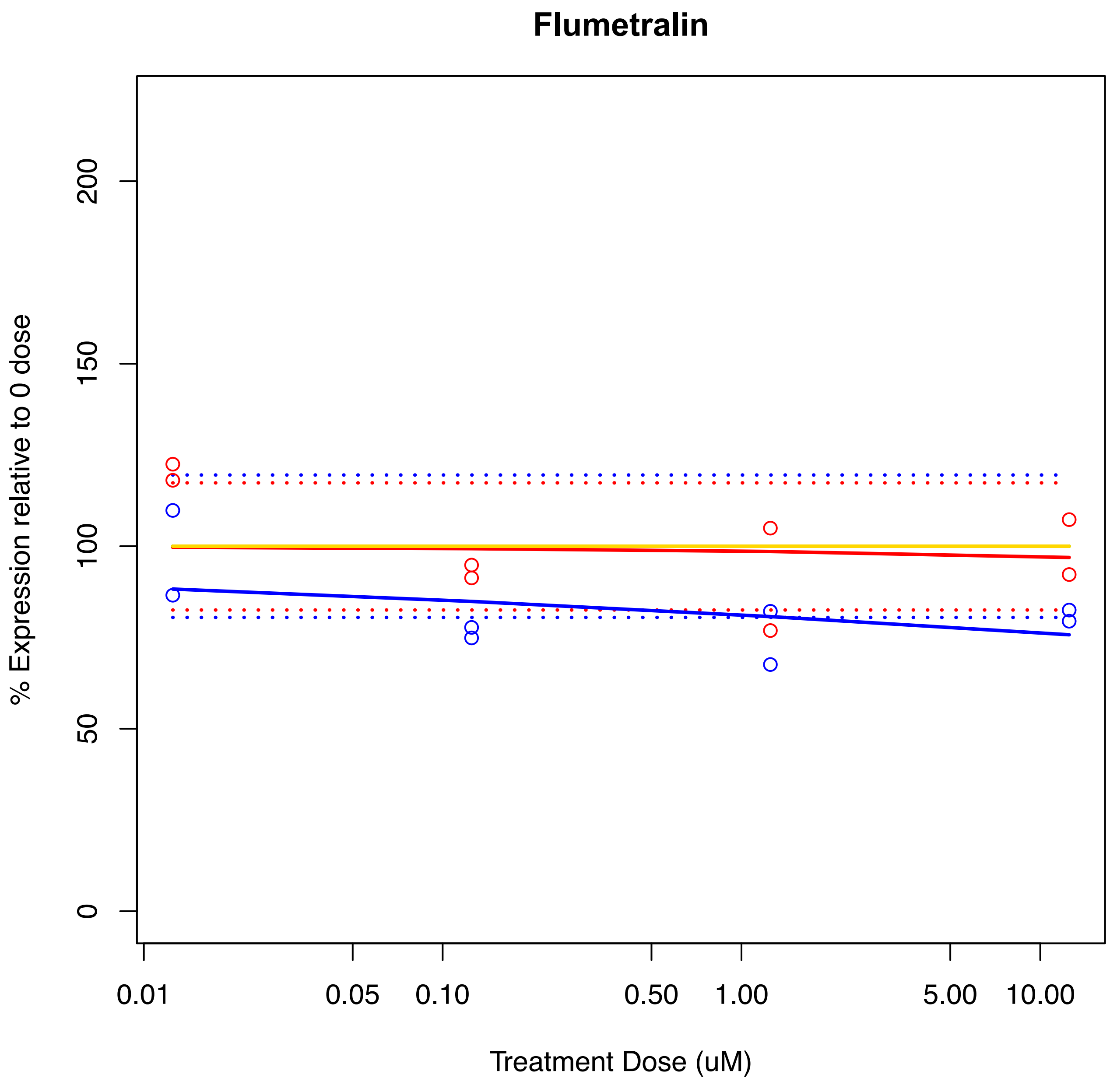
Flumetralin
% Expression relative to 0 dose
0
50
100
150
200
0.01
0.05
0.10
0.50
1.00
5.00
10.00
Treatment Dose (uM)

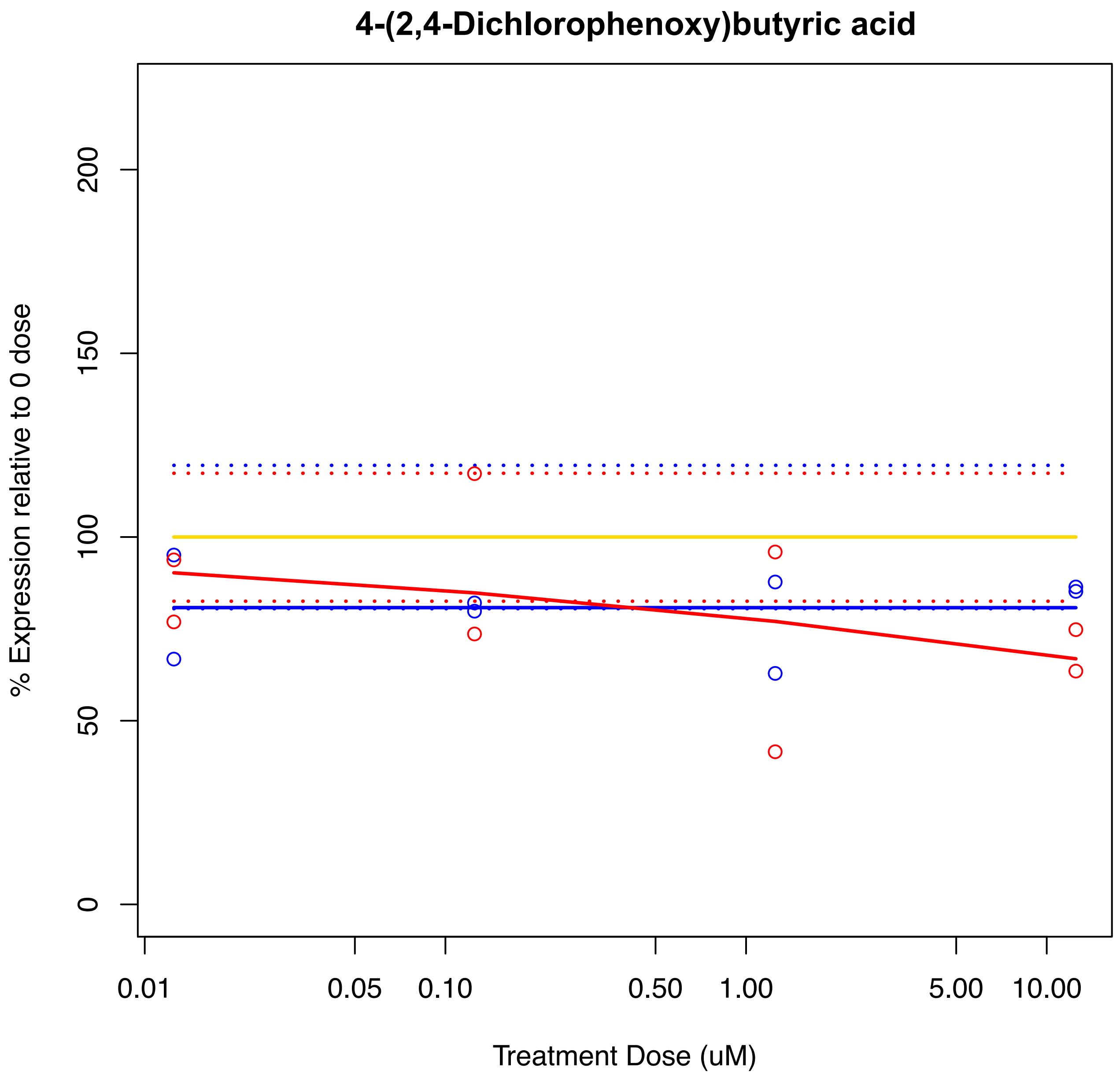
4-(2,4-Dichlorophenoxy)butyric acid
% Expression relative to 0 dose
0
50
100
150
200
0.01
0.05
0.10
0.50
1.00
5.00
10.00
Treatment Dose (uM)

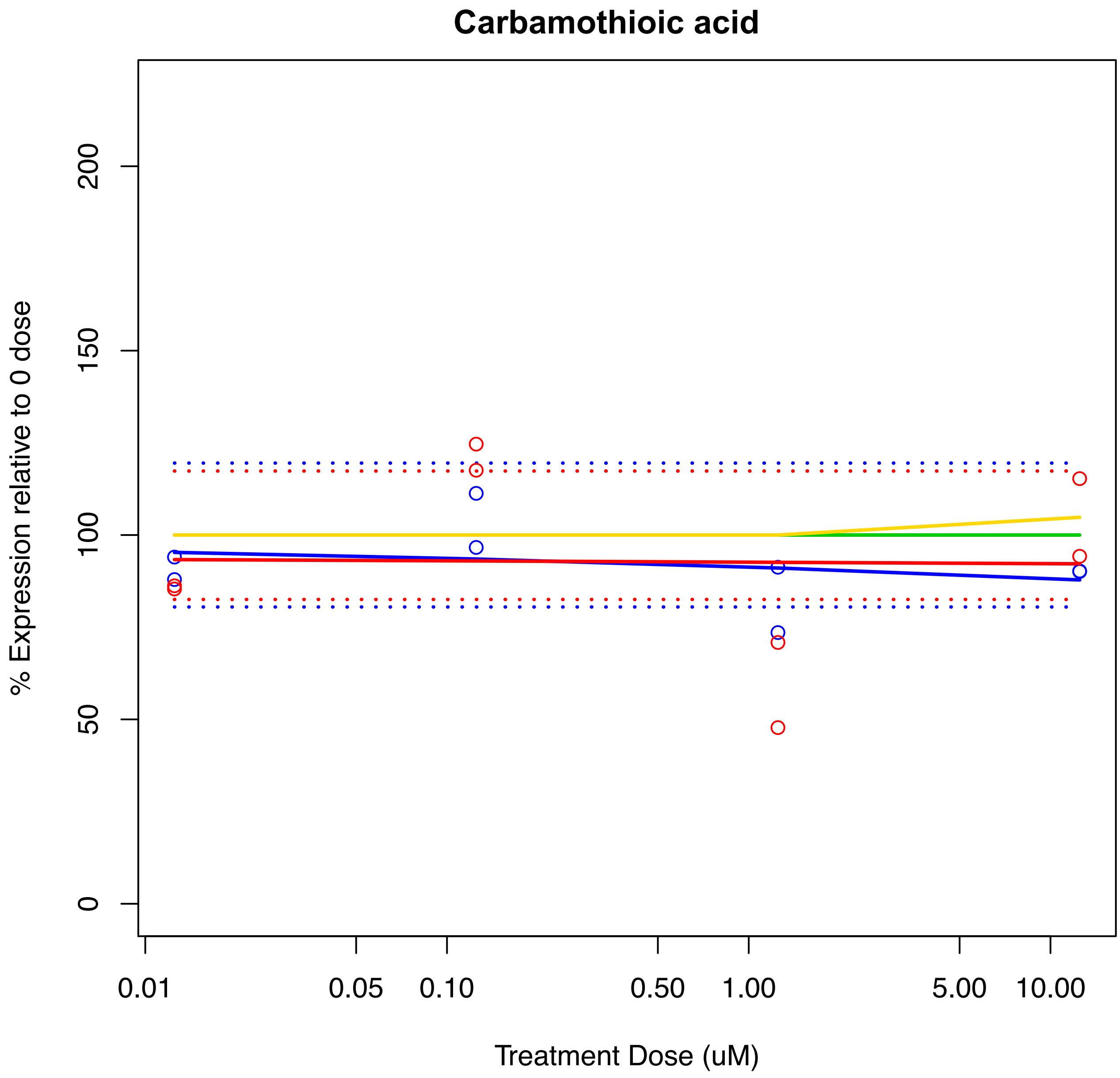

Carbamothioic acid
% Expression relative to 0 dose
0
50
100
150
200
0.01
0.05
0.10
0.50
1.00
5.00
10.00
Treatment Dose (uM)

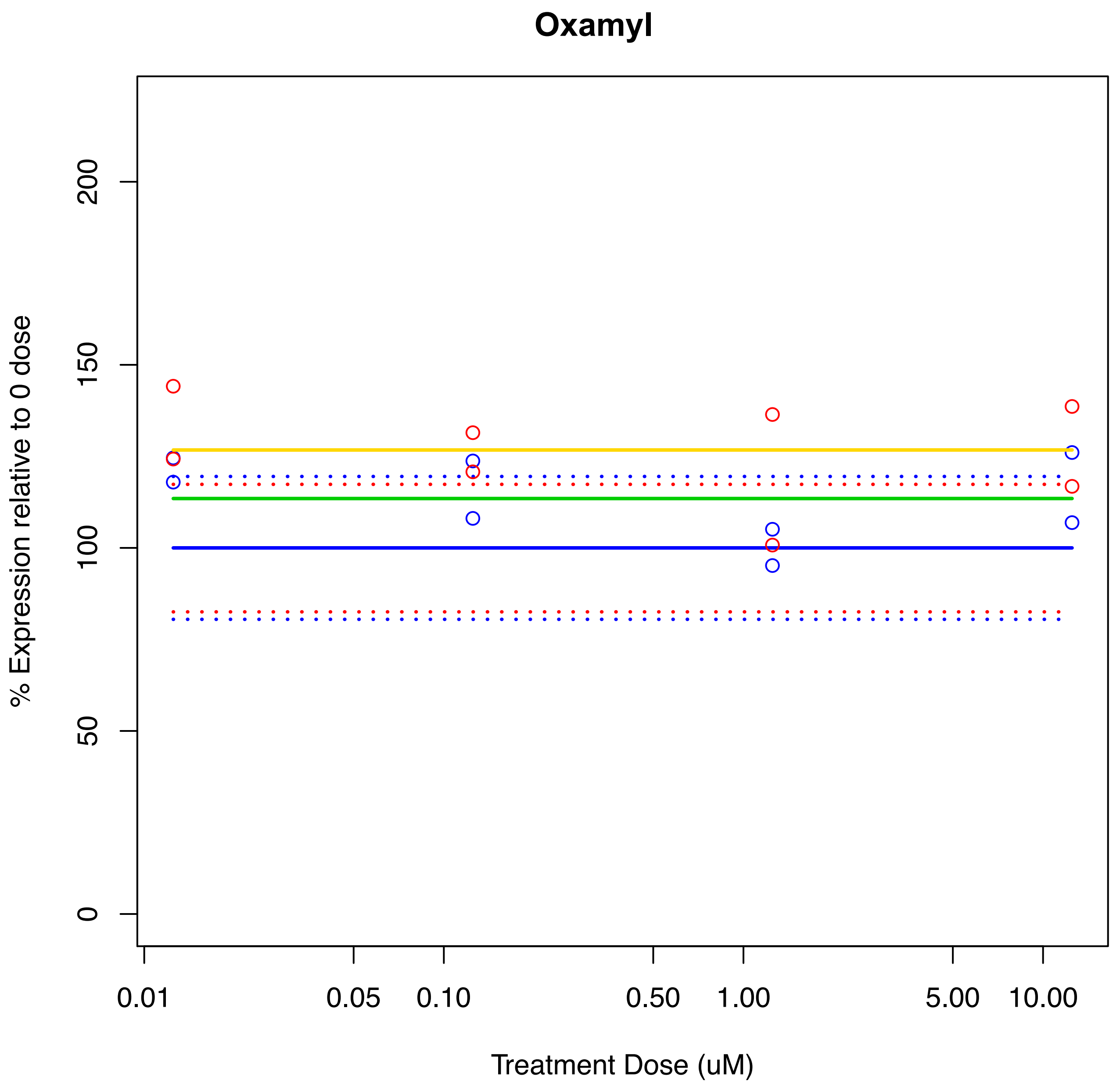
Oxamyl
% Expression relative to 0 dose
0
50
100
150
200
0.01
0.05
0.10
0.50
1.00
5.00
10.00
Treatment Dose (uM)

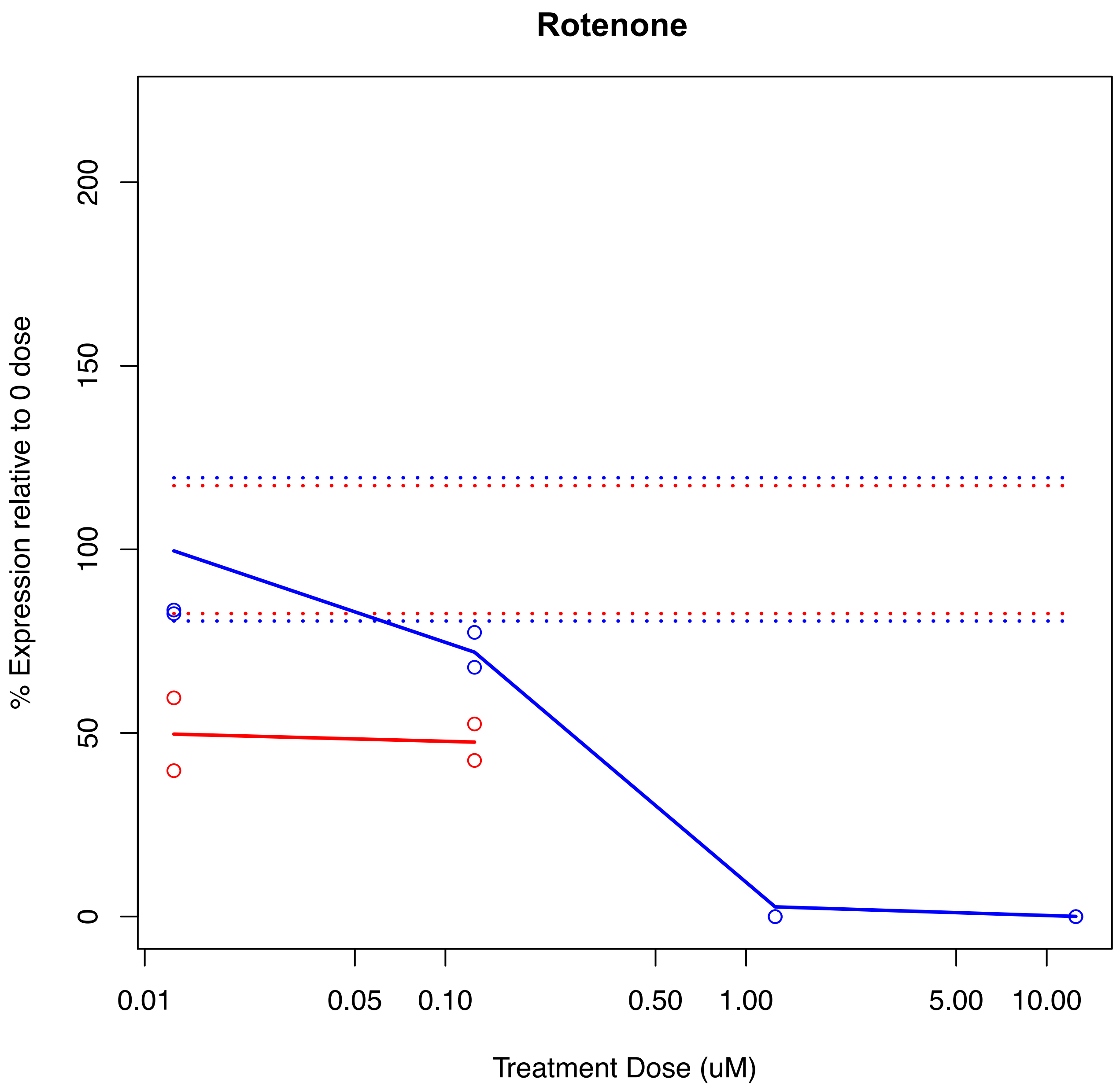
Rotenone
% Expression relative to 0 dose
0
50
100
150
200
0.01
0.05
0.10
0.50
1.00
5.00
10.00
Treatment Dose (uM)

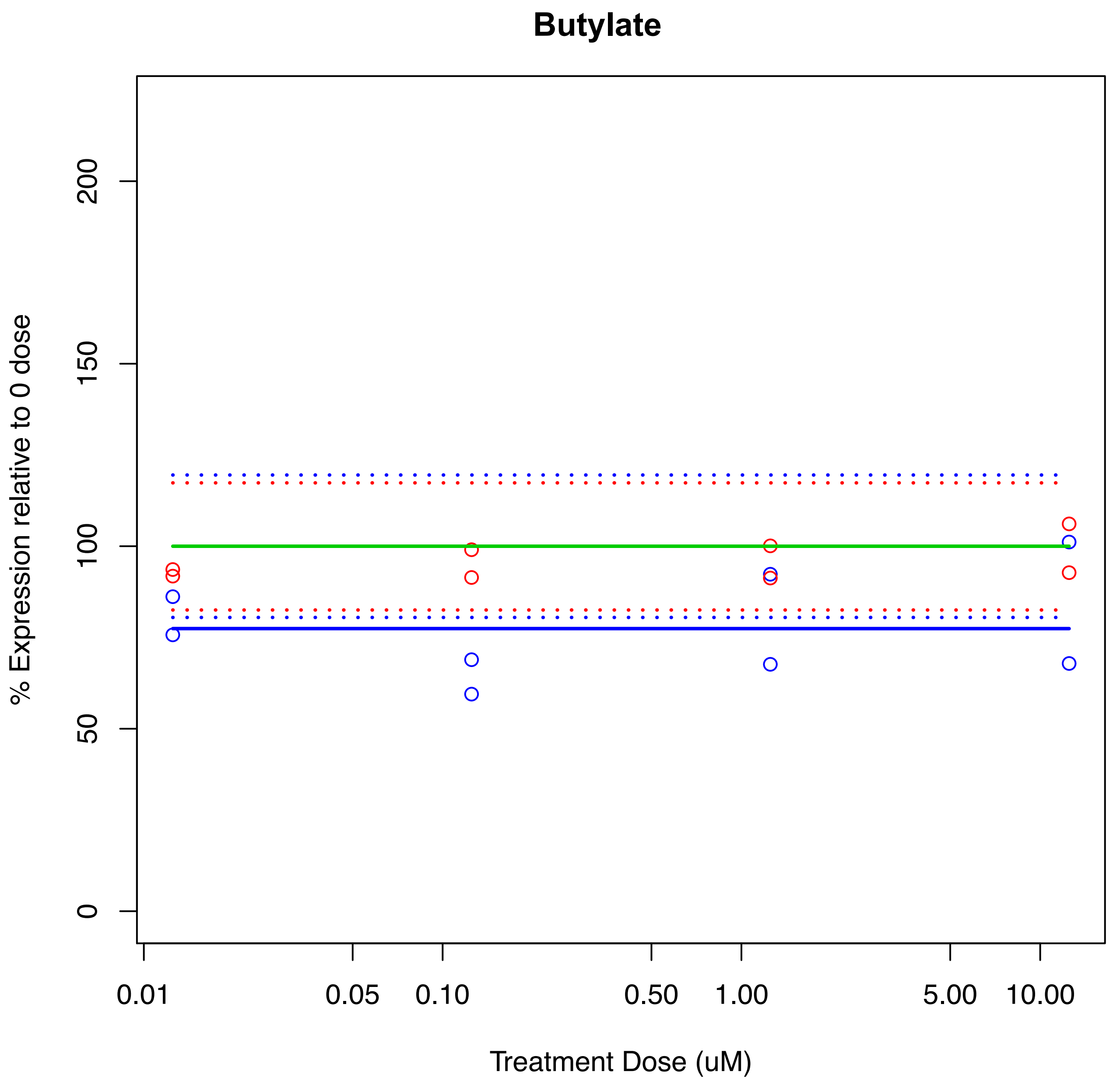

Butylate
% Expression relative to 0 dose
0
50
100
150
200
0.01
0.05
0.10
0.50
1.00
5.00
10.00
Treatment Dose (uM)

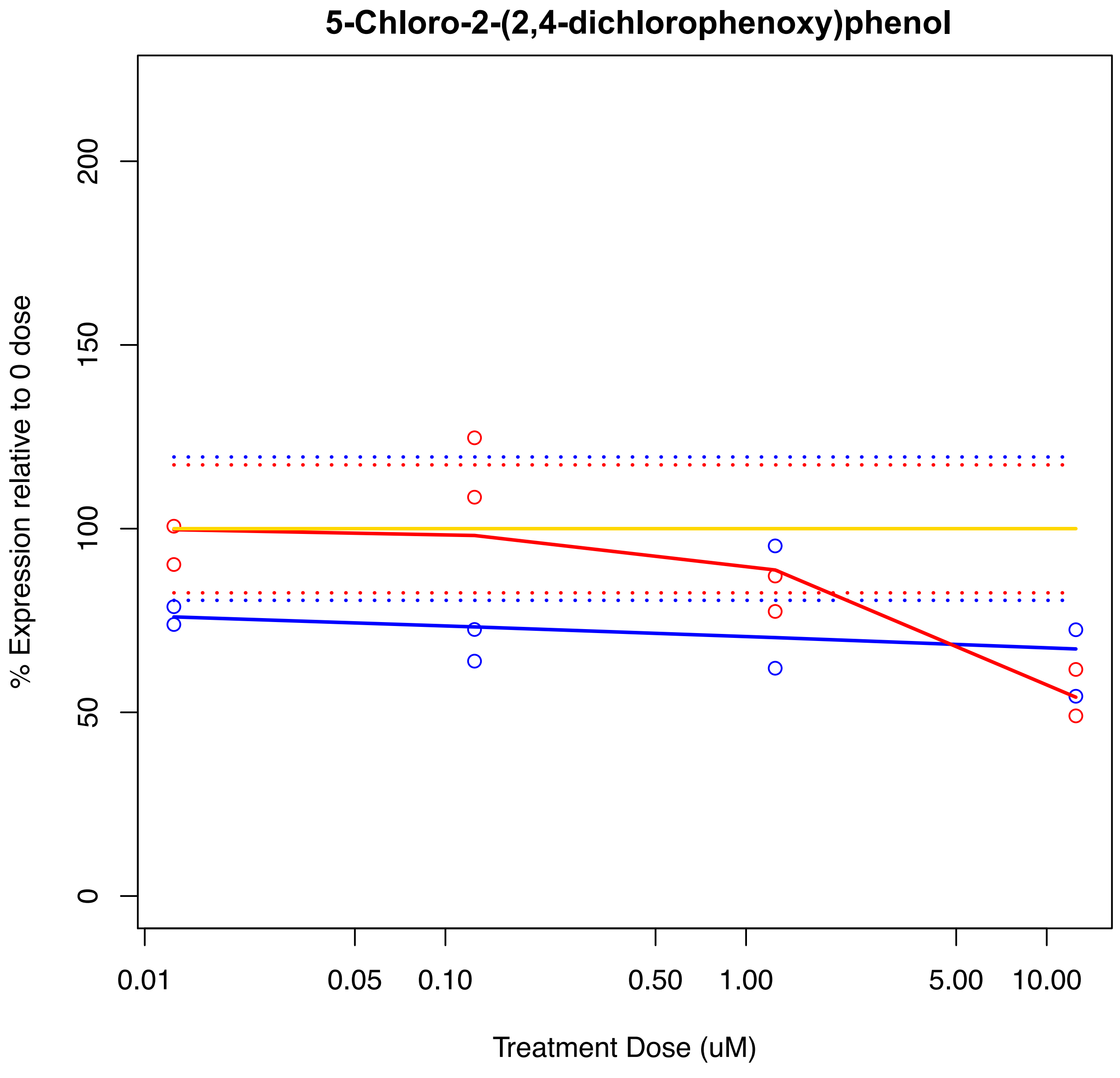
5-Chloro-2-(2,4-dichlorophenoxy)phenol
% Expression relative to 0 dose
0
50
100
150
200
0.01
0.05
0.10
0.50
1.00
5.00
10.00
Treatment Dose (uM)

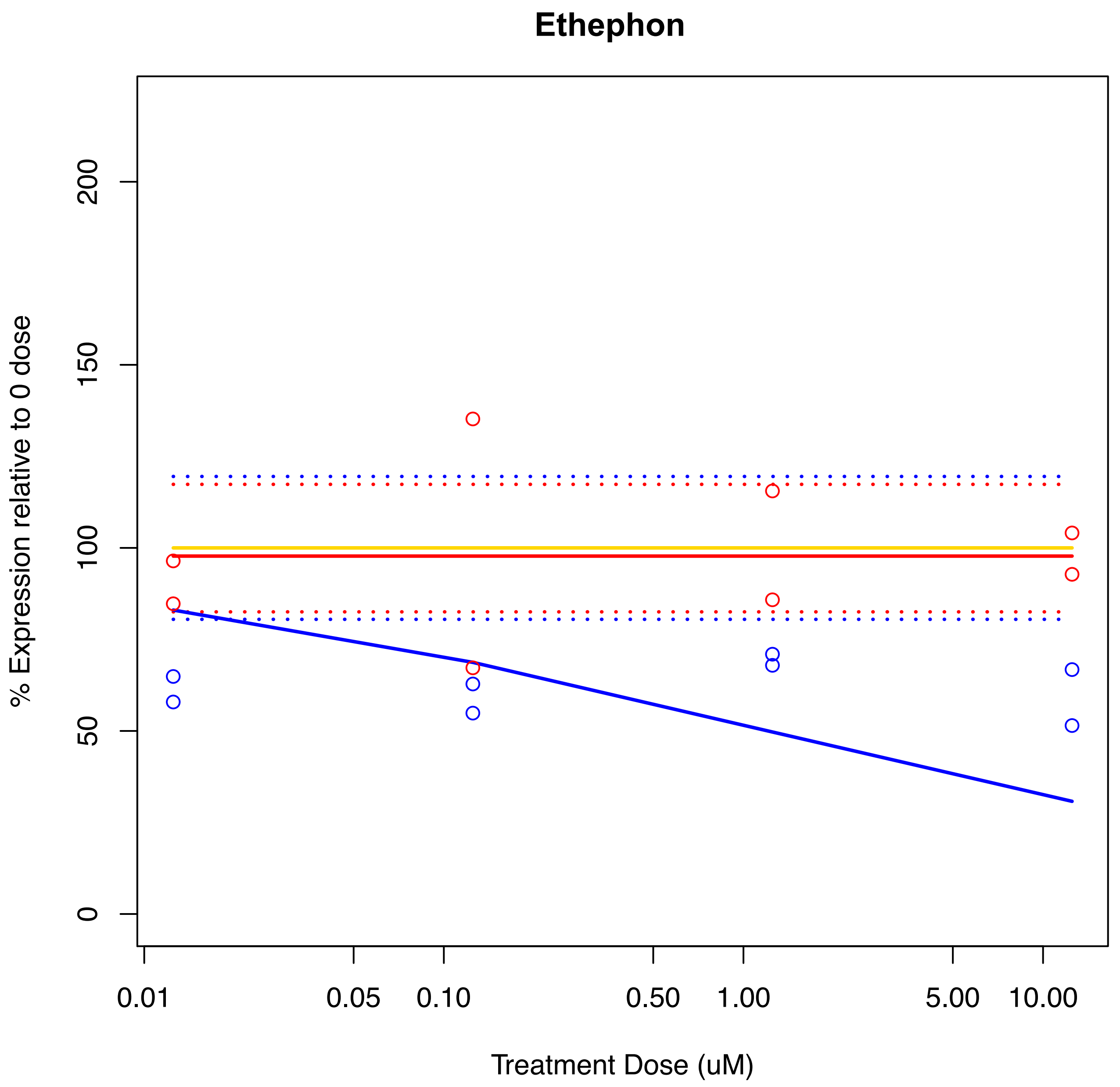
Ethephon
% Expression relative to 0 dose
0
50
100
150
200
0.01
0.05
0.10
0.50
1.00
5.00
10.00
Treatment Dose (uM)

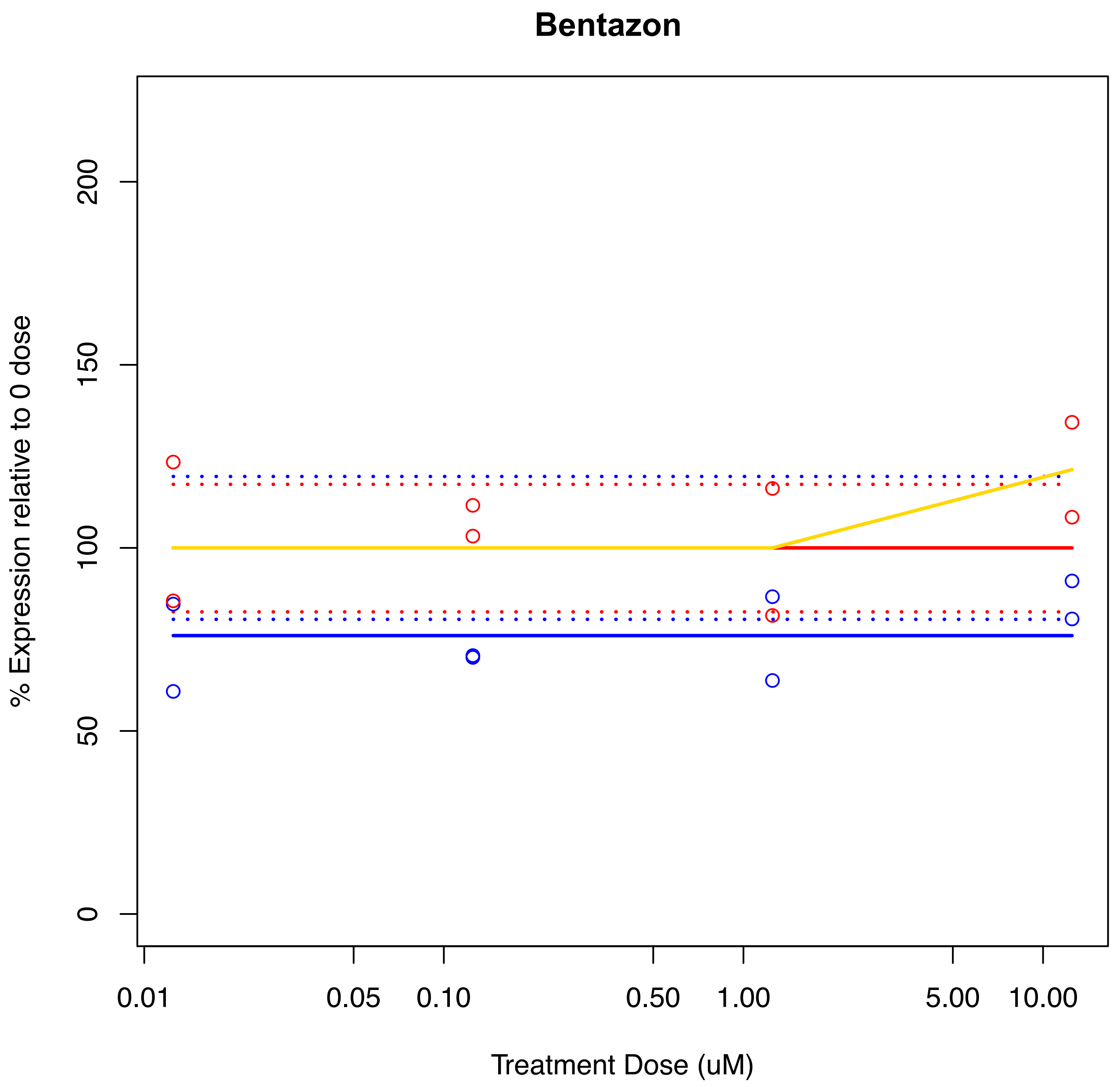
Bentazon
% Expression relative to 0 dose
0
50
100
150
200
0.01
0.05
0.10
0.50
1.00
5.00
10.00
Treatment Dose (uM)

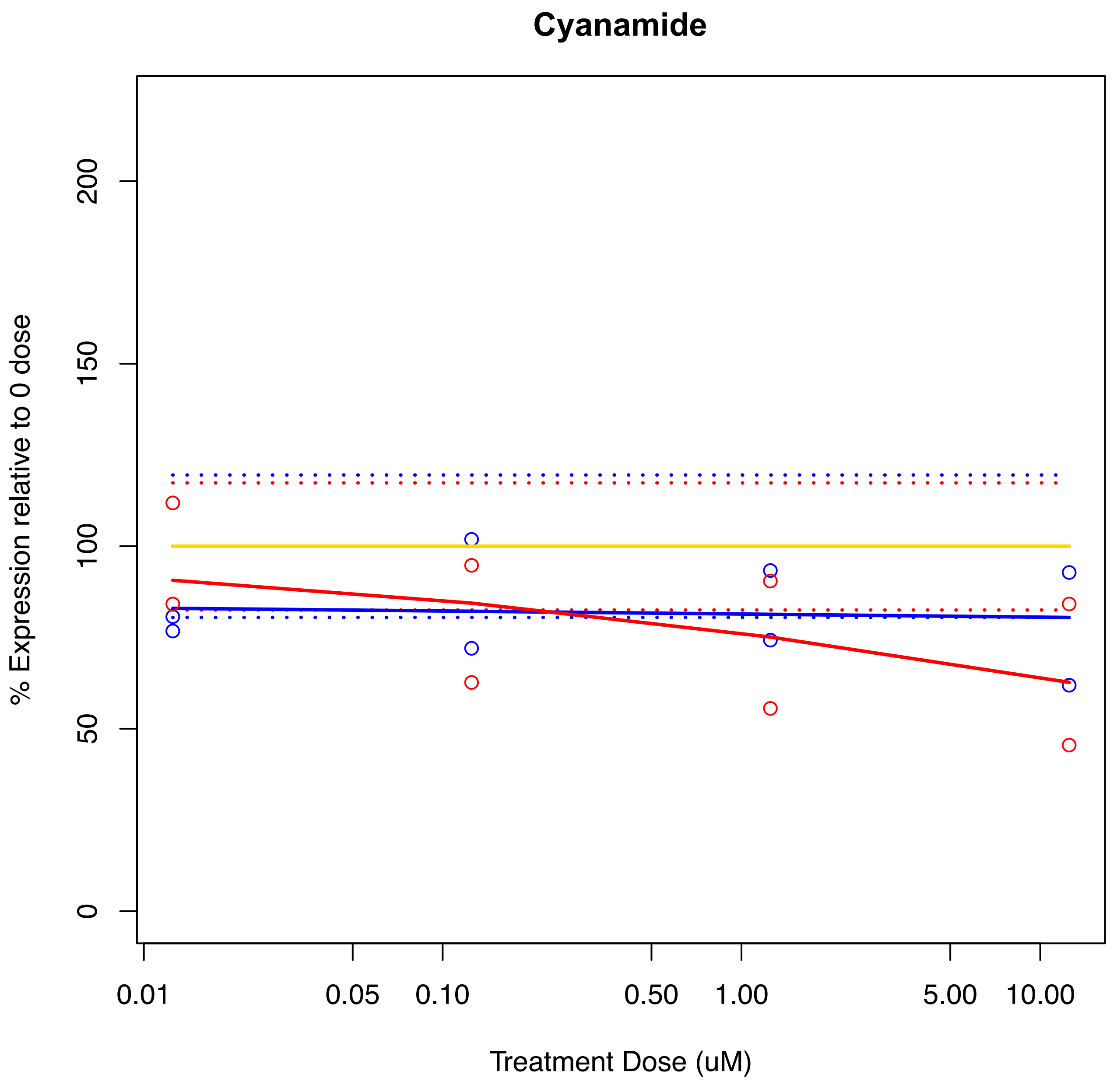

Cyanamide
% Expression relative to 0 dose
0
50
100
150
200
0.01
0.05
0.10
0.50
1.00
5.00
10.00
Treatment Dose (uM)

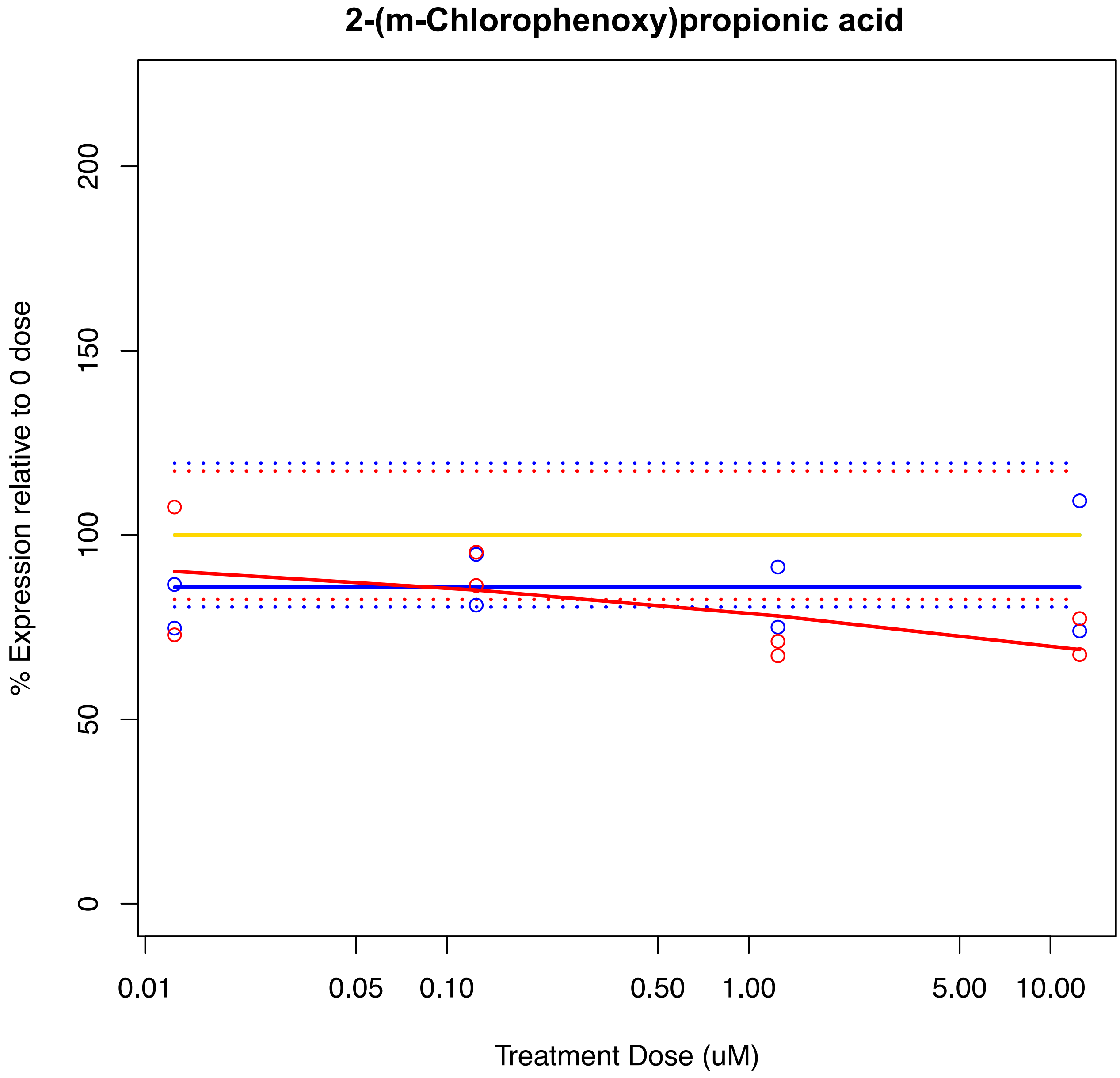

2-(m-Chlorophenoxy)propionic acid
% Expression relative to 0 dose
0
50
100
150
200
0.01
0.05
0.10
0.50
1.00
5.00
10.00
Treatment Dose (uM)

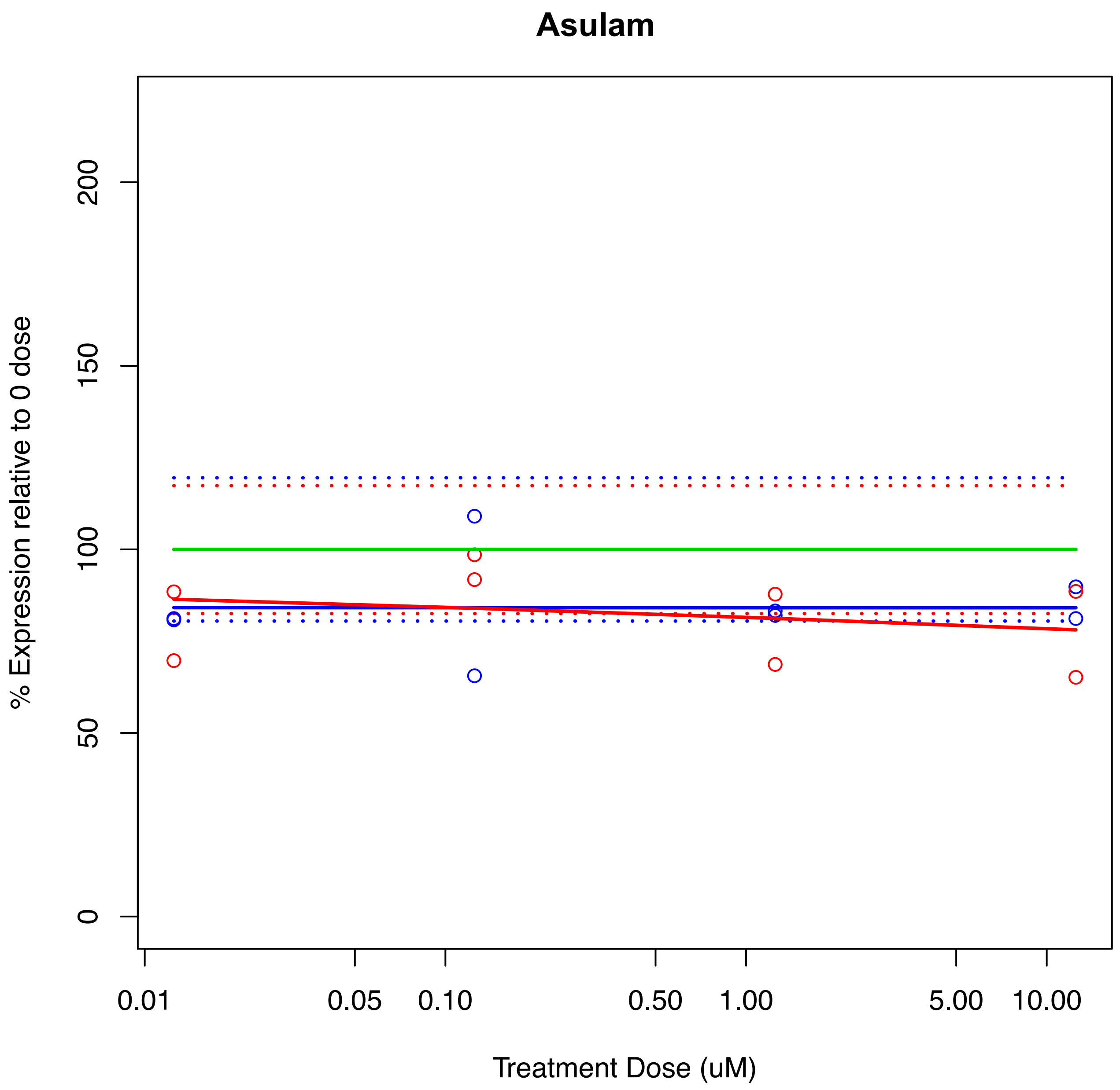
Asulam
% Expression relative to 0 dose
0
50
100
150
200
0.01
0.05
0.10
0.50
1.00
5.00
10.00
Treatment Dose (uM)

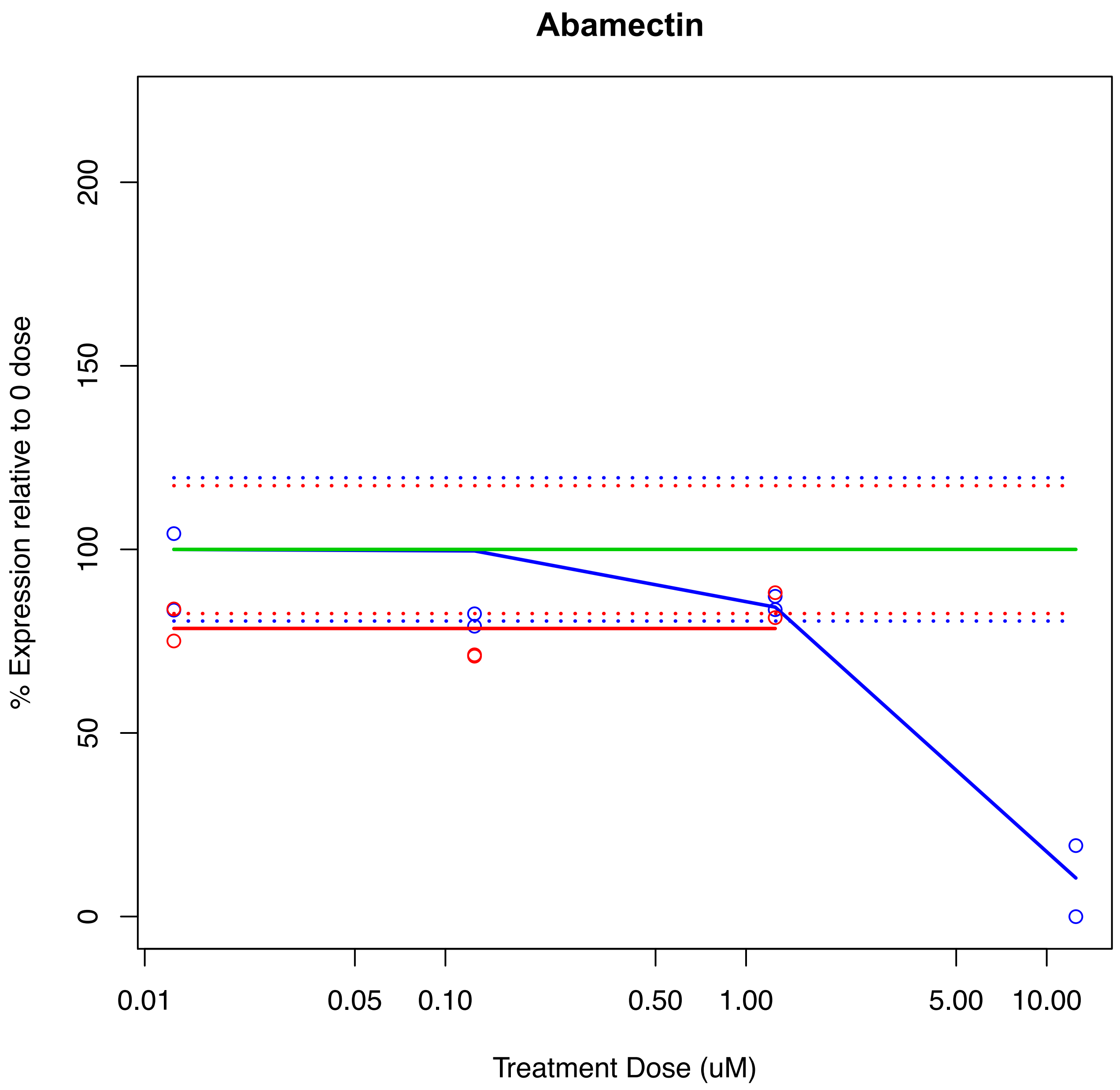
Abamectin
% Expression relative to 0 dose
0
50
100
150
200
0.01
0.05
0.10
0.50
1.00
5.00
10.00
Treatment Dose (uM)

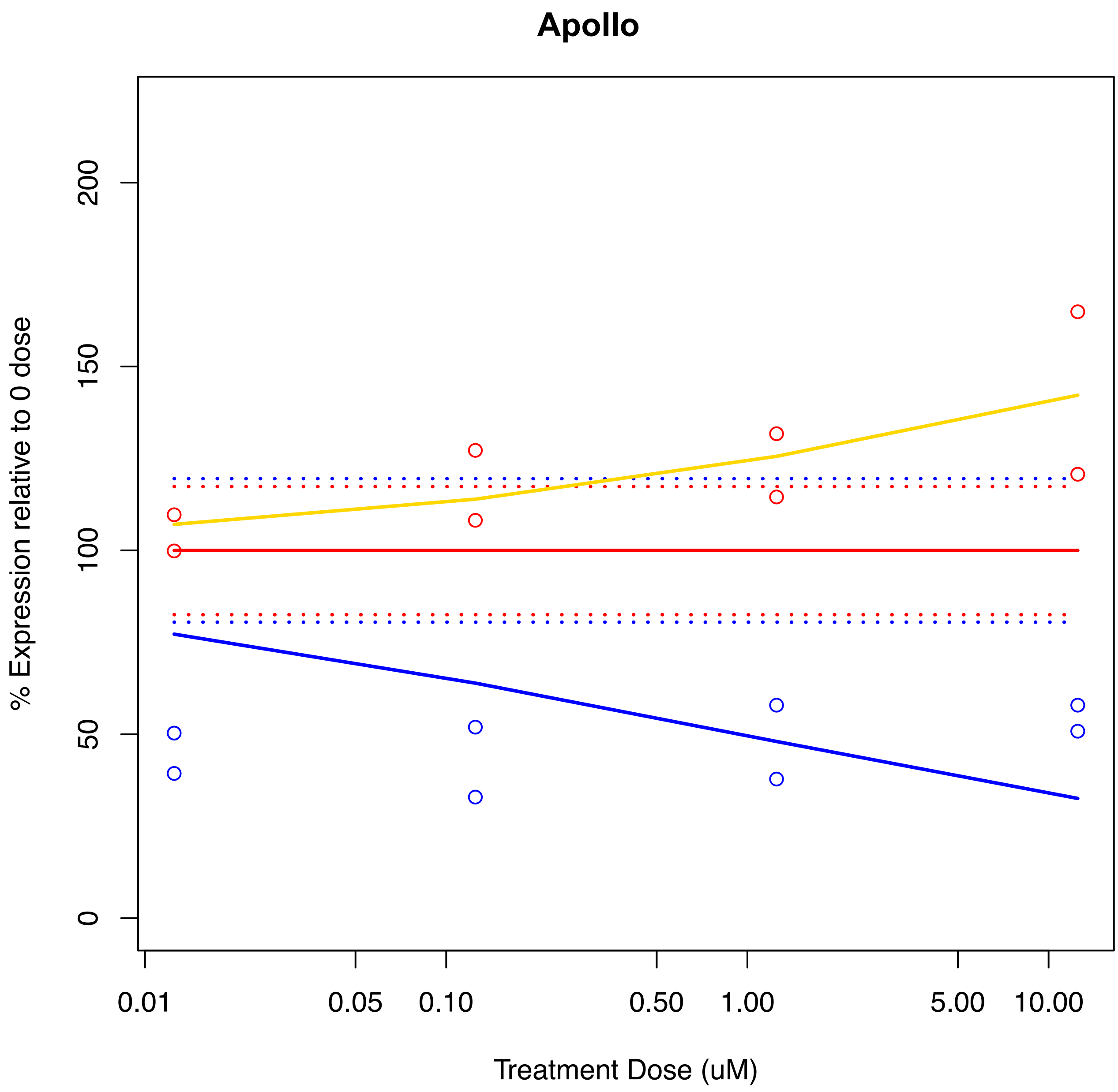

Apollo
200
150
100
50
0
% Expression relative to 0 dose
0.01
0.05
0.10
0.50
1.00
5.00
10.00
Treatment Dose (uM)

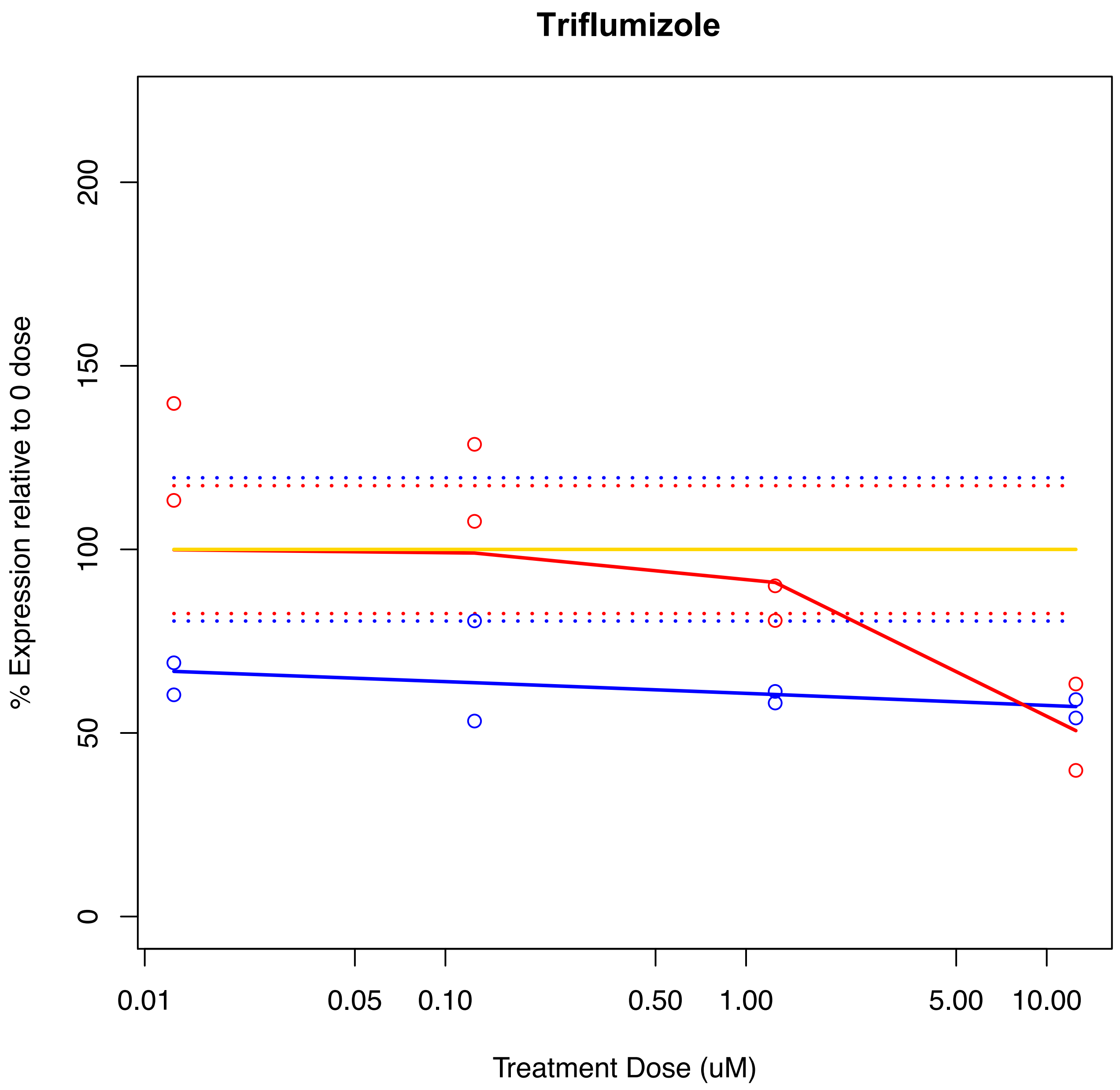

Triflumizole
% Expression relative to 0 dose
0
50
100
150
200
0.01
0.05
0.10
0.50
1.00
5.00
10.00
Treatment Dose (uM)

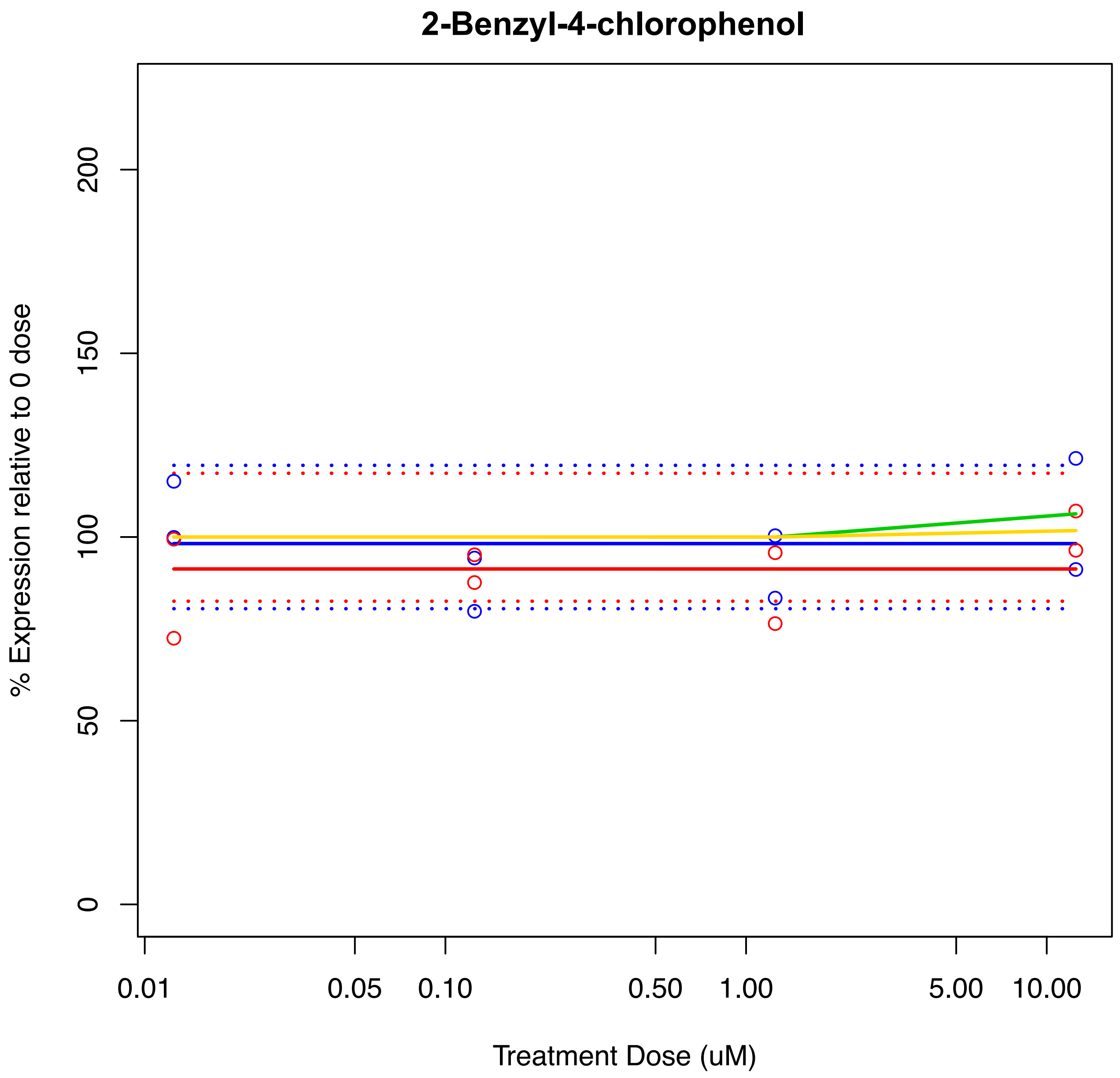
2-Benzyl-4-chlorophenol
% Expression relative to 0 dose
0
50
100
150
200
0.01
0.05
0.10
0.50
1.00
5.00
10.00
Treatment Dose (uM)

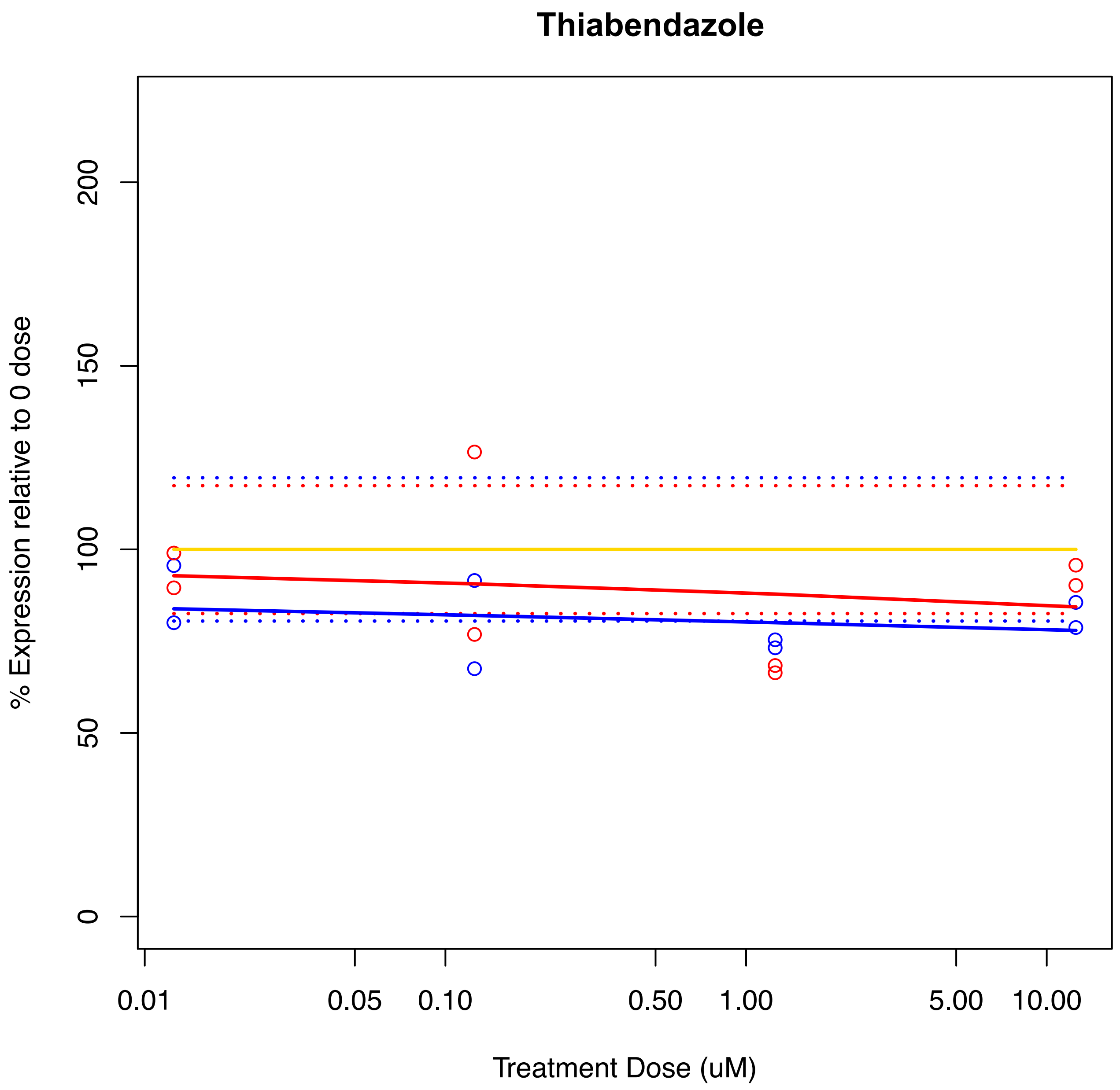
Thiabendazole
% Expression relative to 0 dose
0
50
100
150
200
0.01
0.05
0.10
0.50
1.00
5.00
10.00
Treatment Dose (uM)

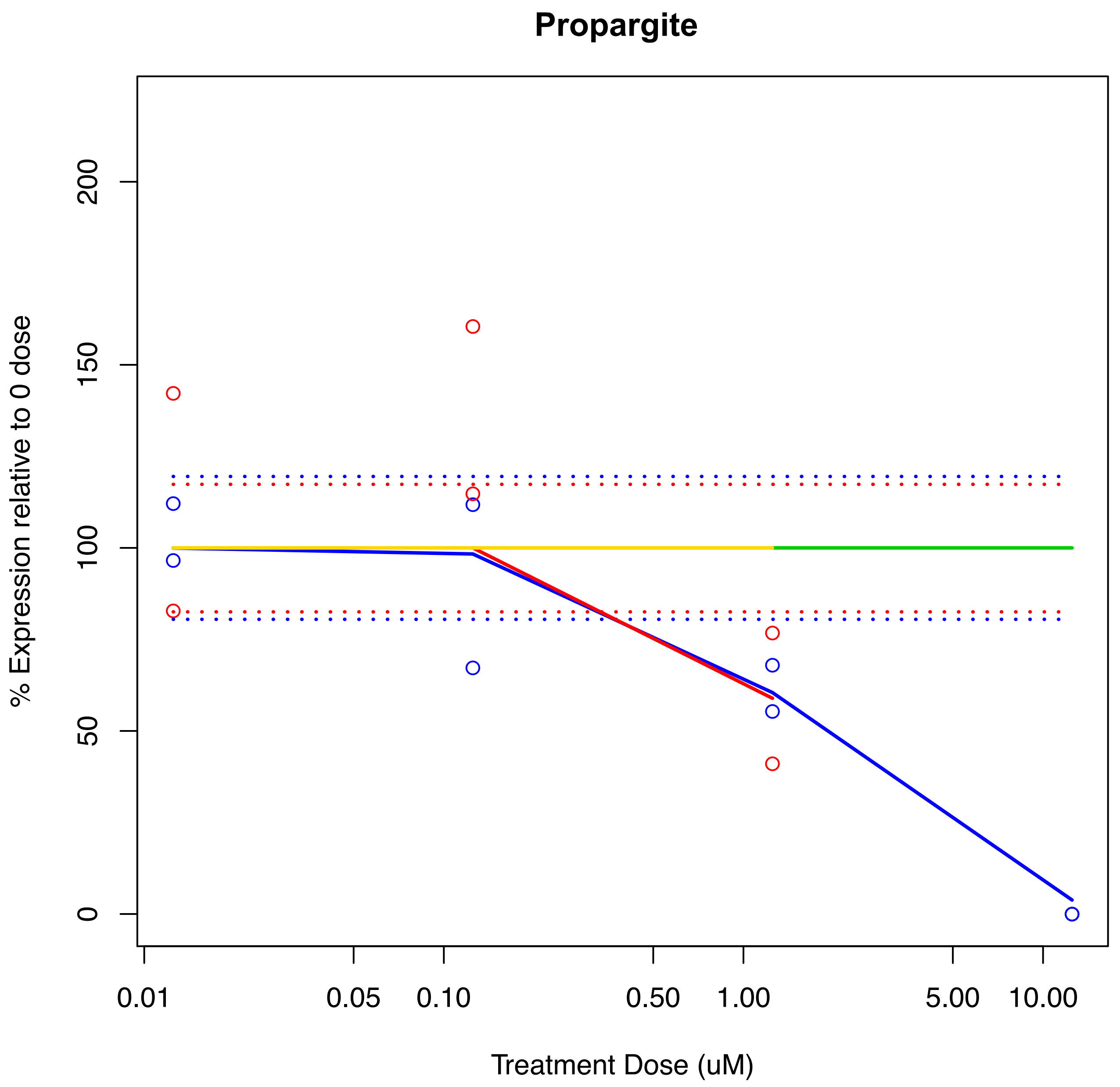
Propargite
% Expression relative to 0 dose
0
50
100
150
200
0.01
0.05
0.10
0.50
1.00
5.00
10.00
Treatment Dose (uM)

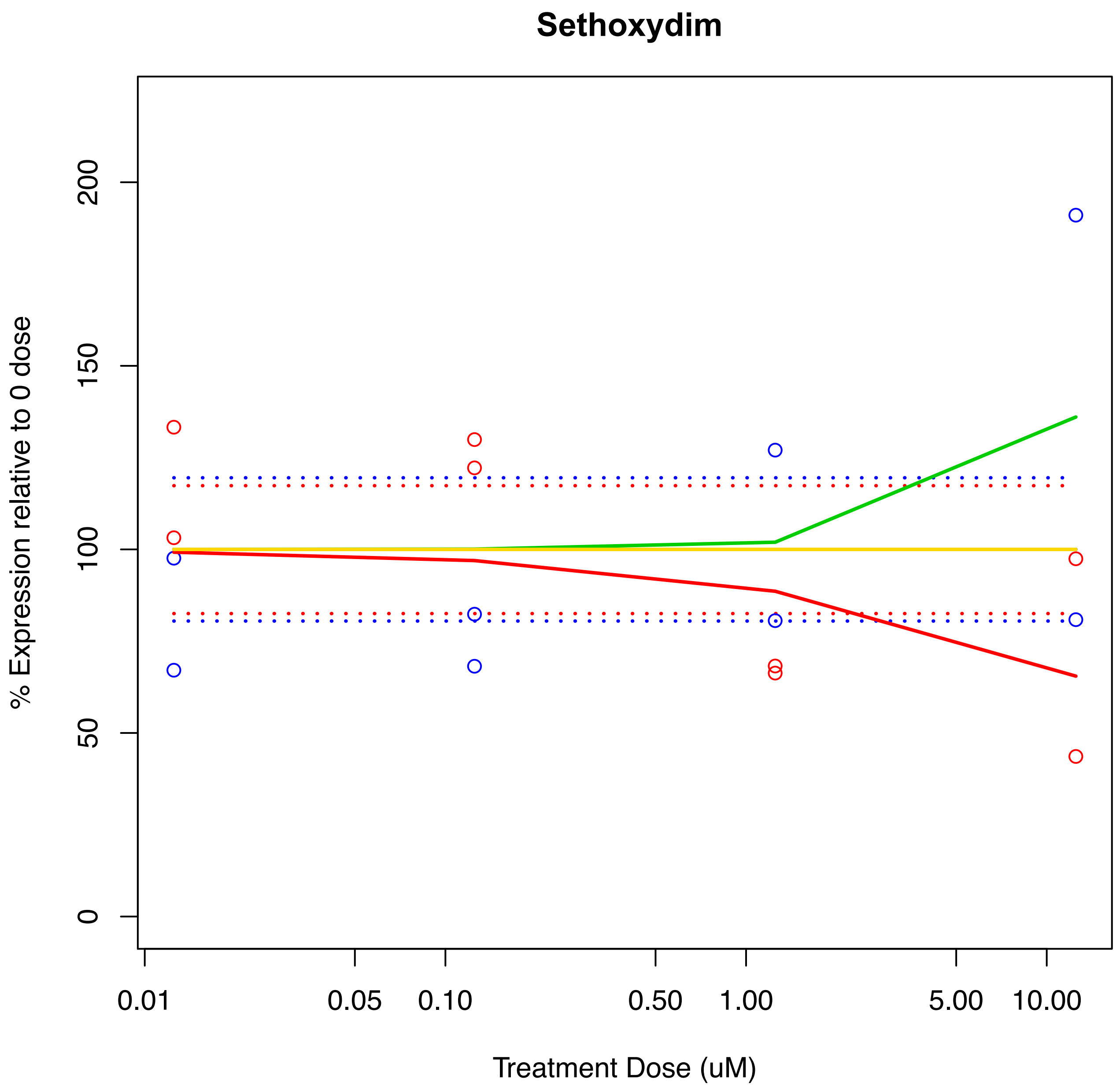

Sethoxydim
% Expression relative to 0 dose
0
50
100
150
200
0.01
0.05
0.10
0.50
1.00
5.00
10.00
Treatment Dose (uM)

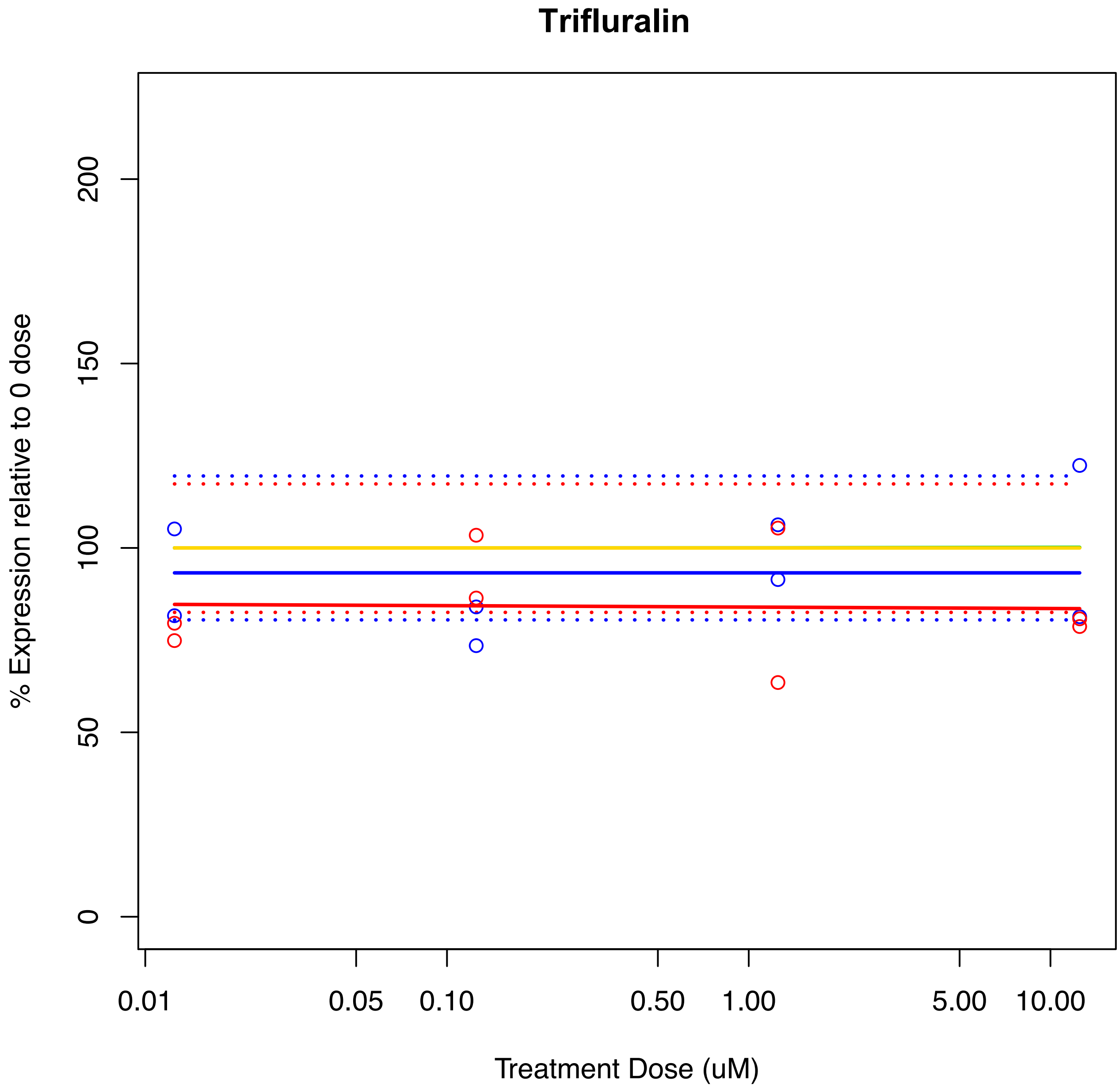
Trifluralin
% Expression relative to 0 dose
0
50
100
150
200
0.01
0.05
0.10
0.50
1.00
5.00
10.00
Treatment Dose (uM)

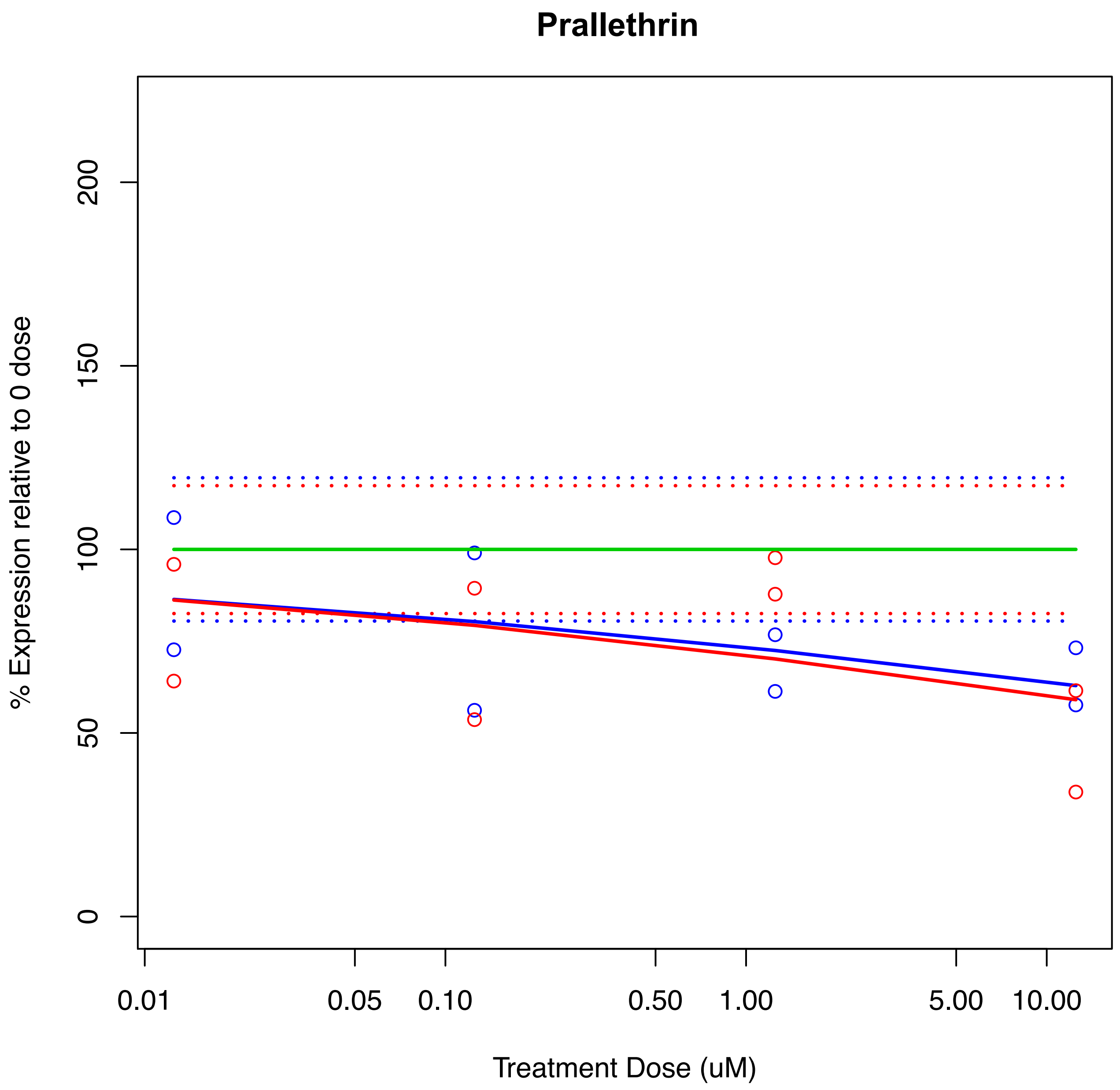
Prallethrin
% Expression relative to 0 dose
0
50
100
150
200
0.01
0.05
0.10
0.50
1.00
5.00
10.00
Treatment Dose (uM)

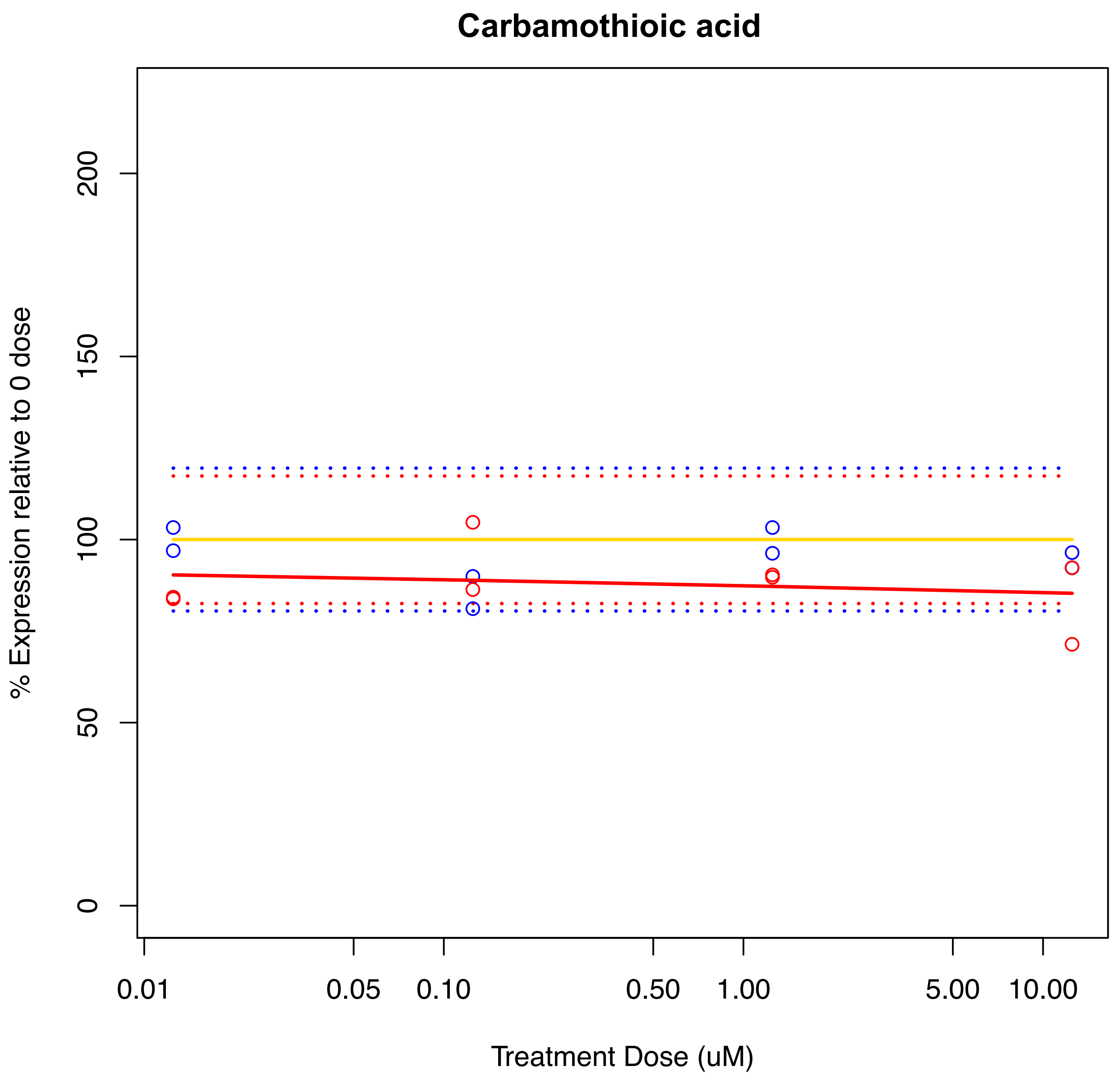

Carbamothioic acid
% Expression relative to 0 dose
0
50
100
150
200
0.01
0.05
0.10
0.50
1.00
5.00
10.00
Treatment Dose (uM)

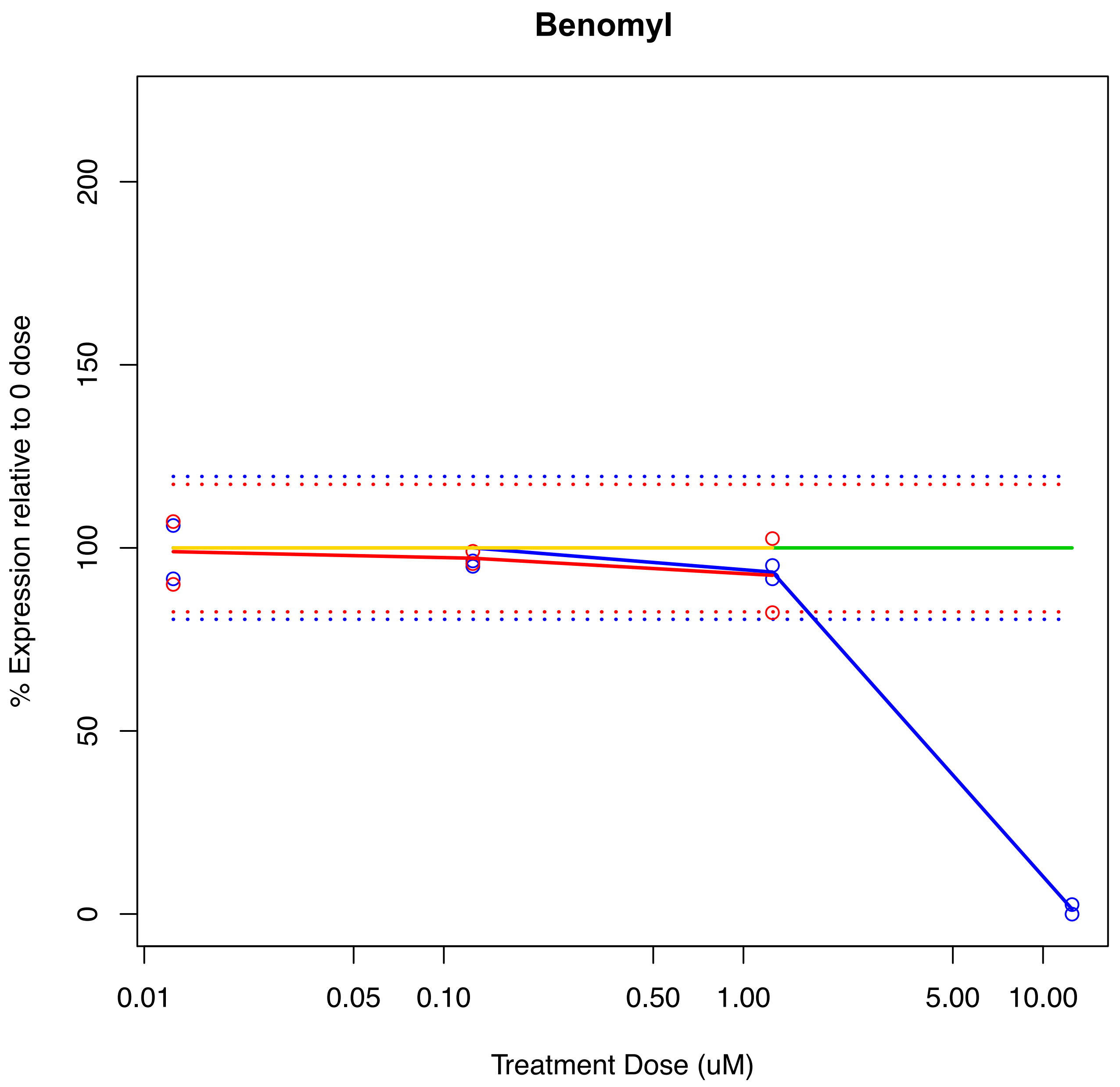
Benomyl
% Expression relative to 0 dose
0
50
100
150
200
0.01
0.05
0.10
0.50
1.00
5.00
10.00
Treatment Dose (uM)

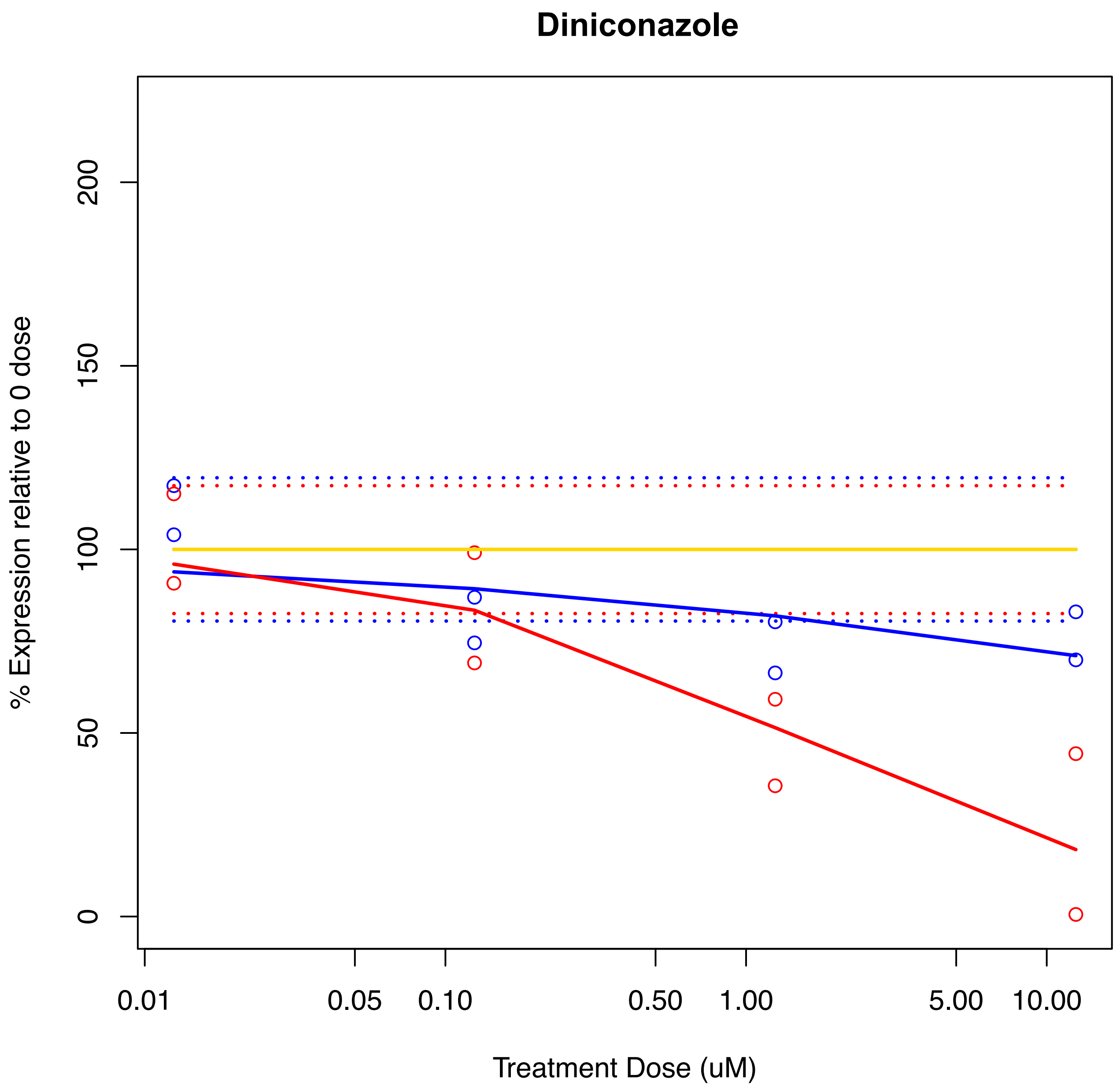

Diniconazole
% Expression relative to 0 dose
0
50
100
150
200
0.01
0.05
0.10
0.50
1.00
5.00
10.00
Treatment Dose (uM)

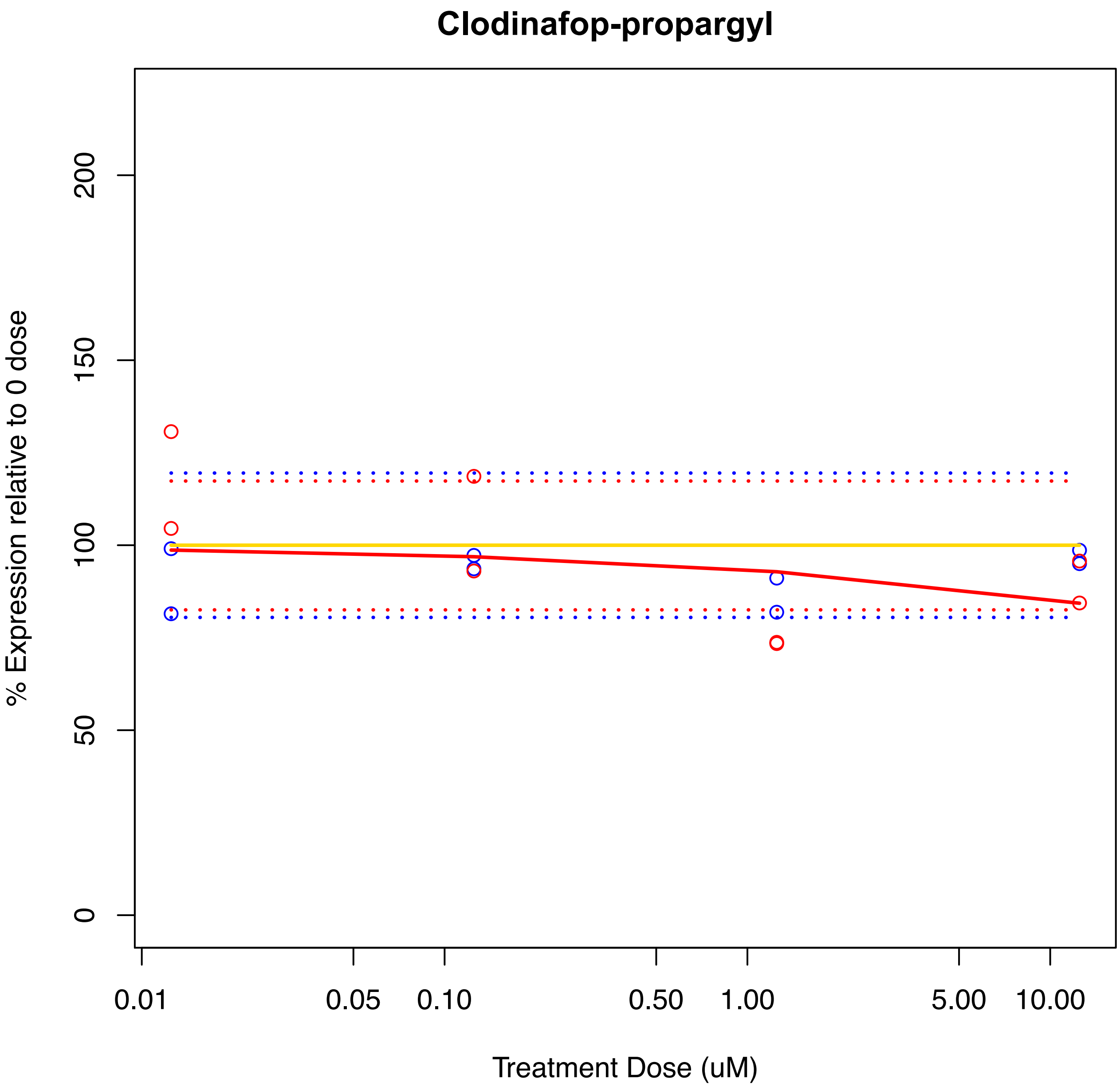
Clodinafop-propargyl
% Expression relative to 0 dose
0
50
100
150
200
0.01
0.05
0.10
0.50
1.00
5.00
10.00
Treatment Dose (uM)

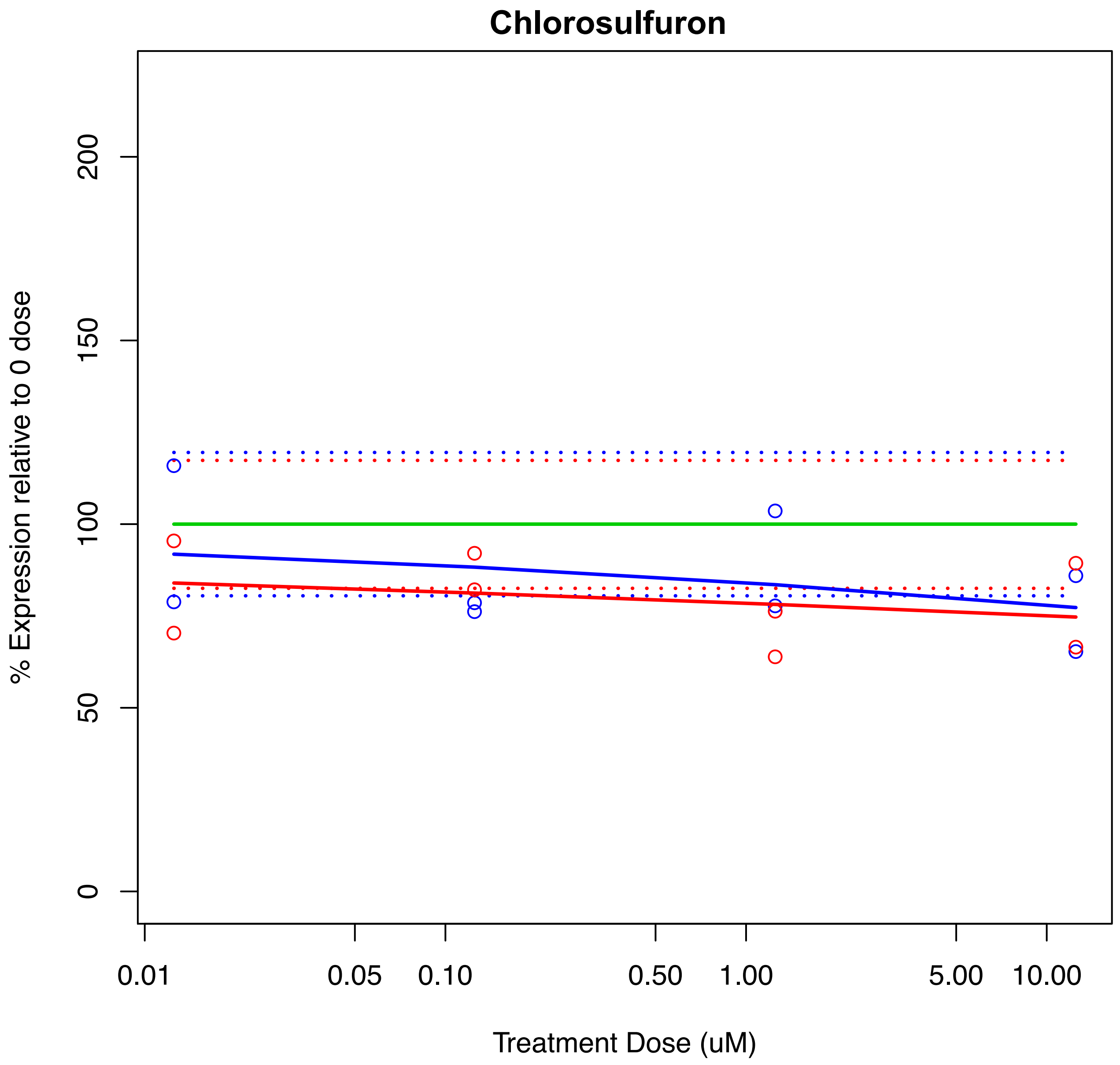
Chlorosulfuron
% Expression relative to 0 dose
0
50
100
150
200
0.01
0.05
0.10
0.50
1.00
5.00
10.00
Treatment Dose (uM)

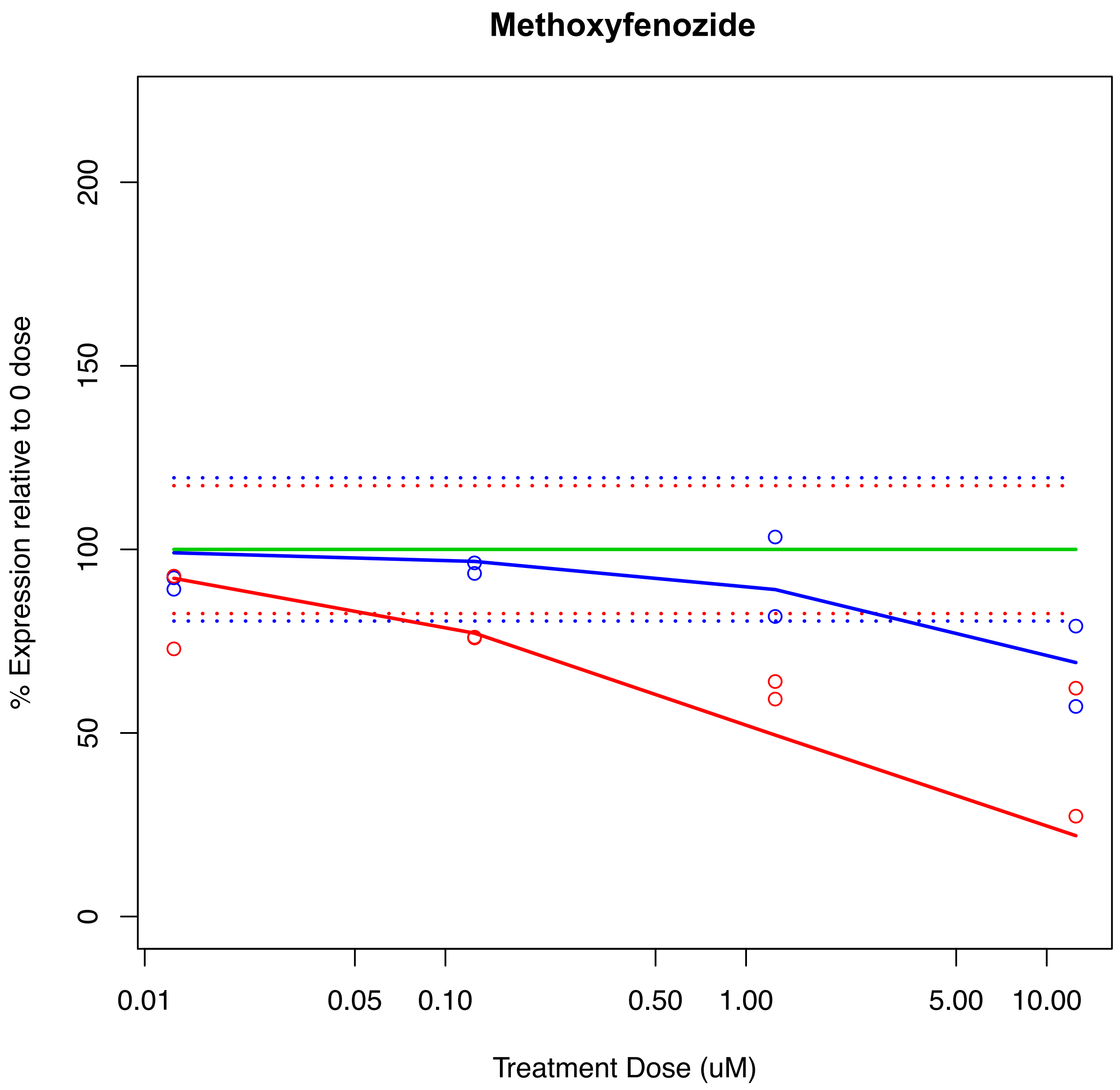
Methoxyfenozide
% Expression relative to 0 dose
0
50
100
150
200
0.01
0.05
0.10
0.50
1.00
5.00
10.00
Treatment Dose (uM)

N,N-Dimethyl-2-3-(4,6-dimethoxypyrimidin-2-yl)ureidosulfonylU-4-formylaminobenzamide

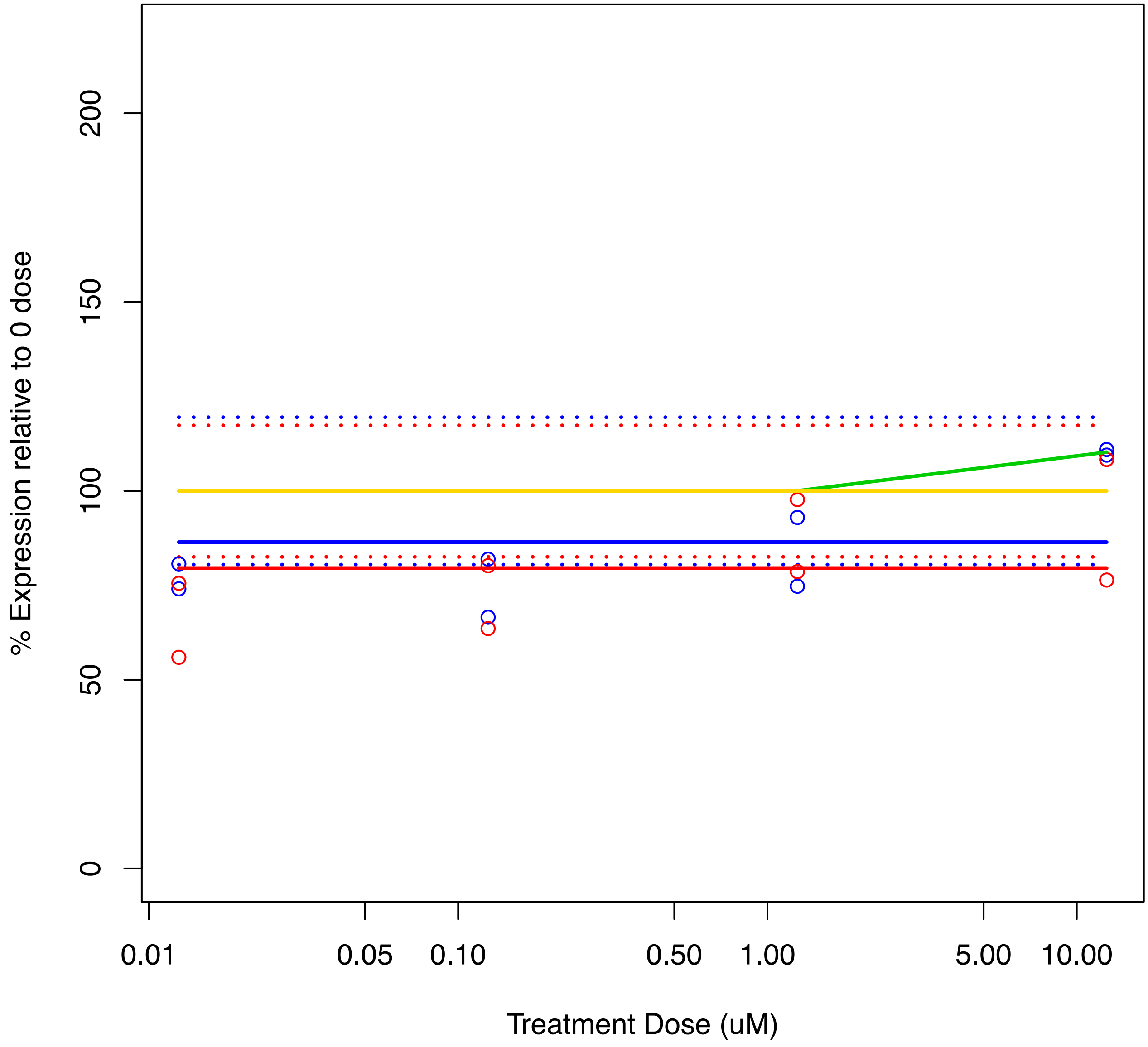

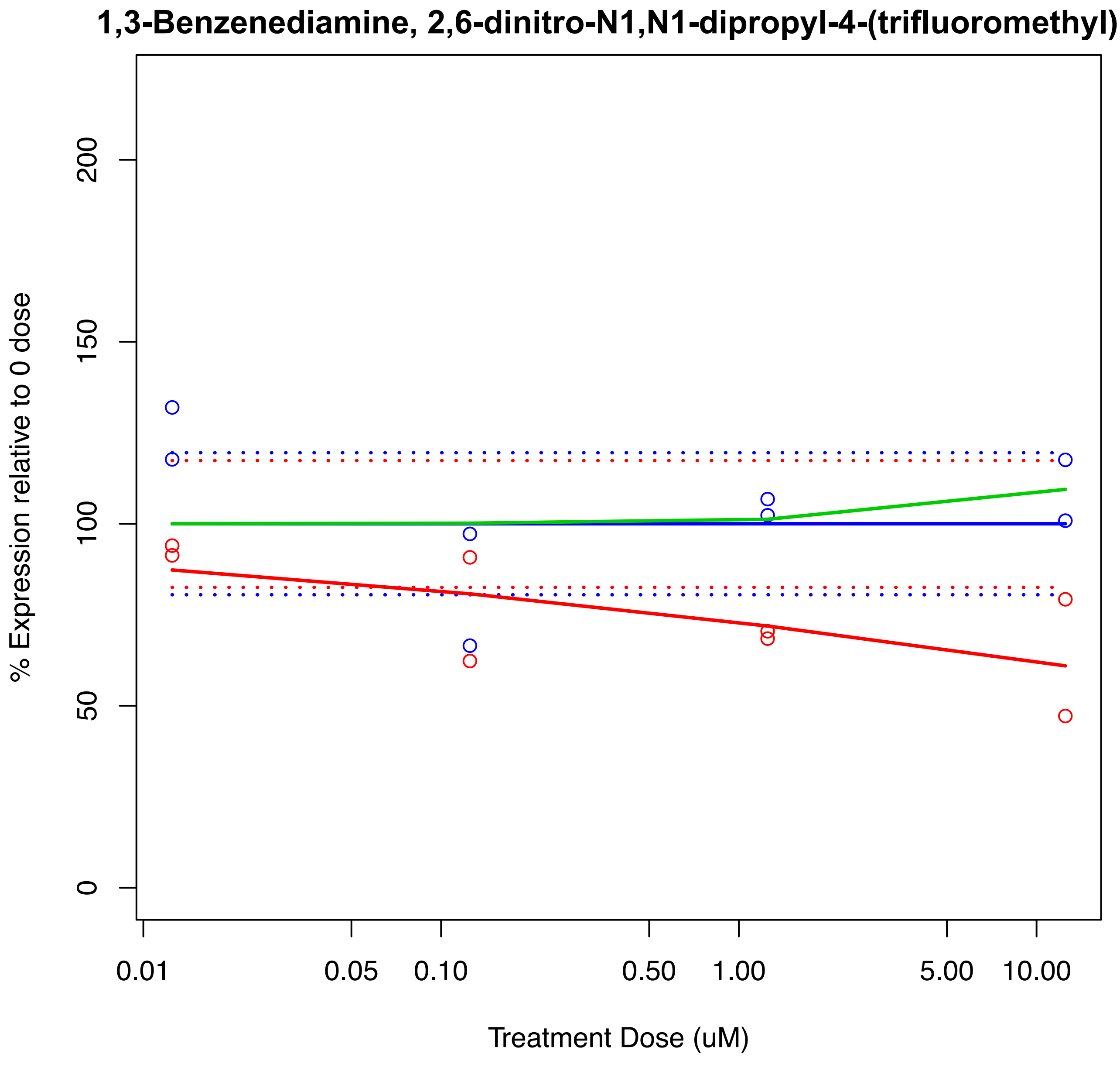

1,3-Benzenediamine, 2,6-dinitro-N1,N1-dipropyl-4-(trifluoromethyl)-
% Expression relative to 0 dose
0
50
100
150
200
0.01
0.05
0.10
0.50
1.00
5.00
10.00
Treatment Dose (uM)

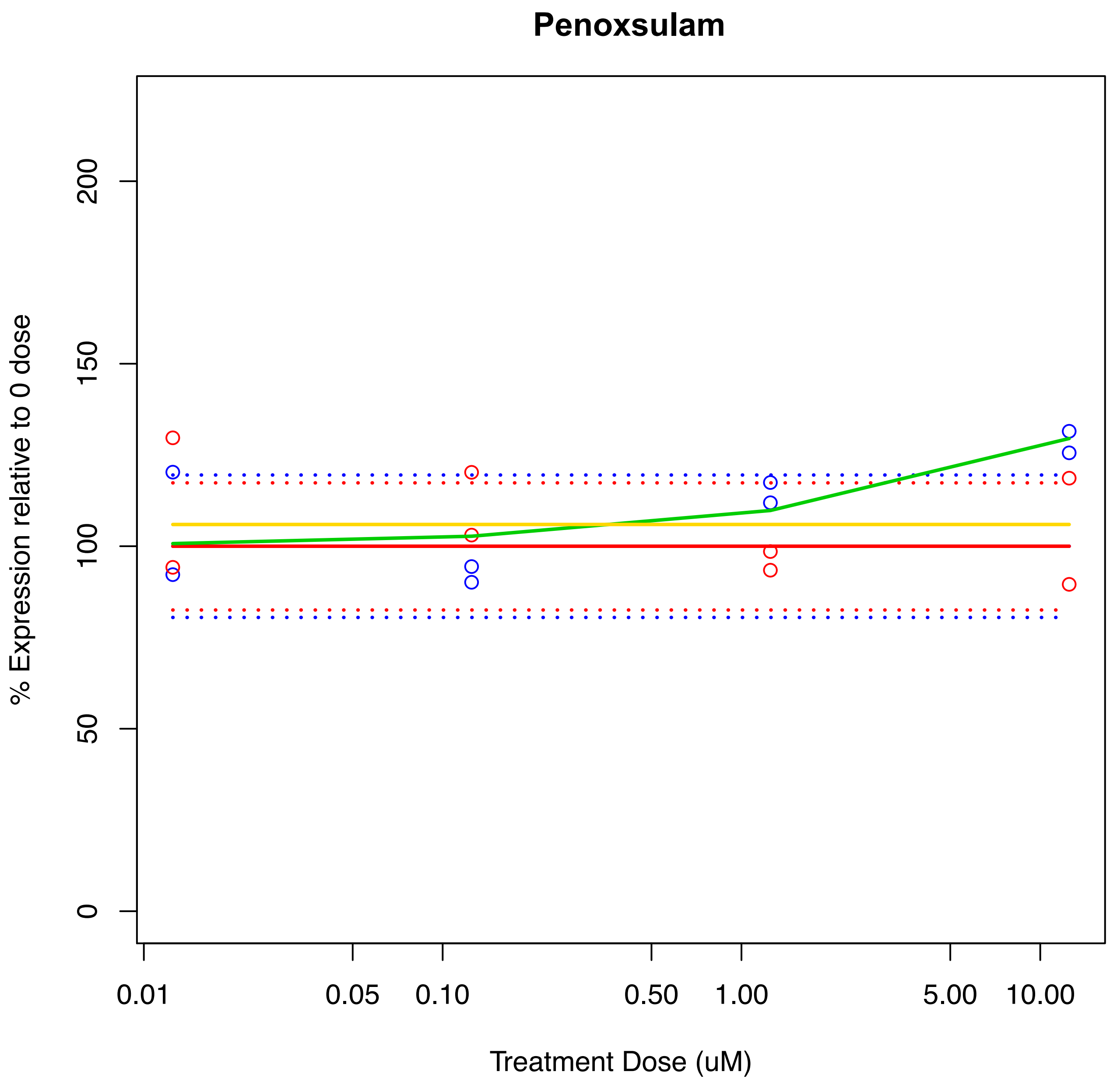

Penoxsulam
% Expression relative to 0 dose
0
50
100
150
200
0.01
0.05
0.10
0.50
1.00
5.00
10.00
Treatment Dose (uM)

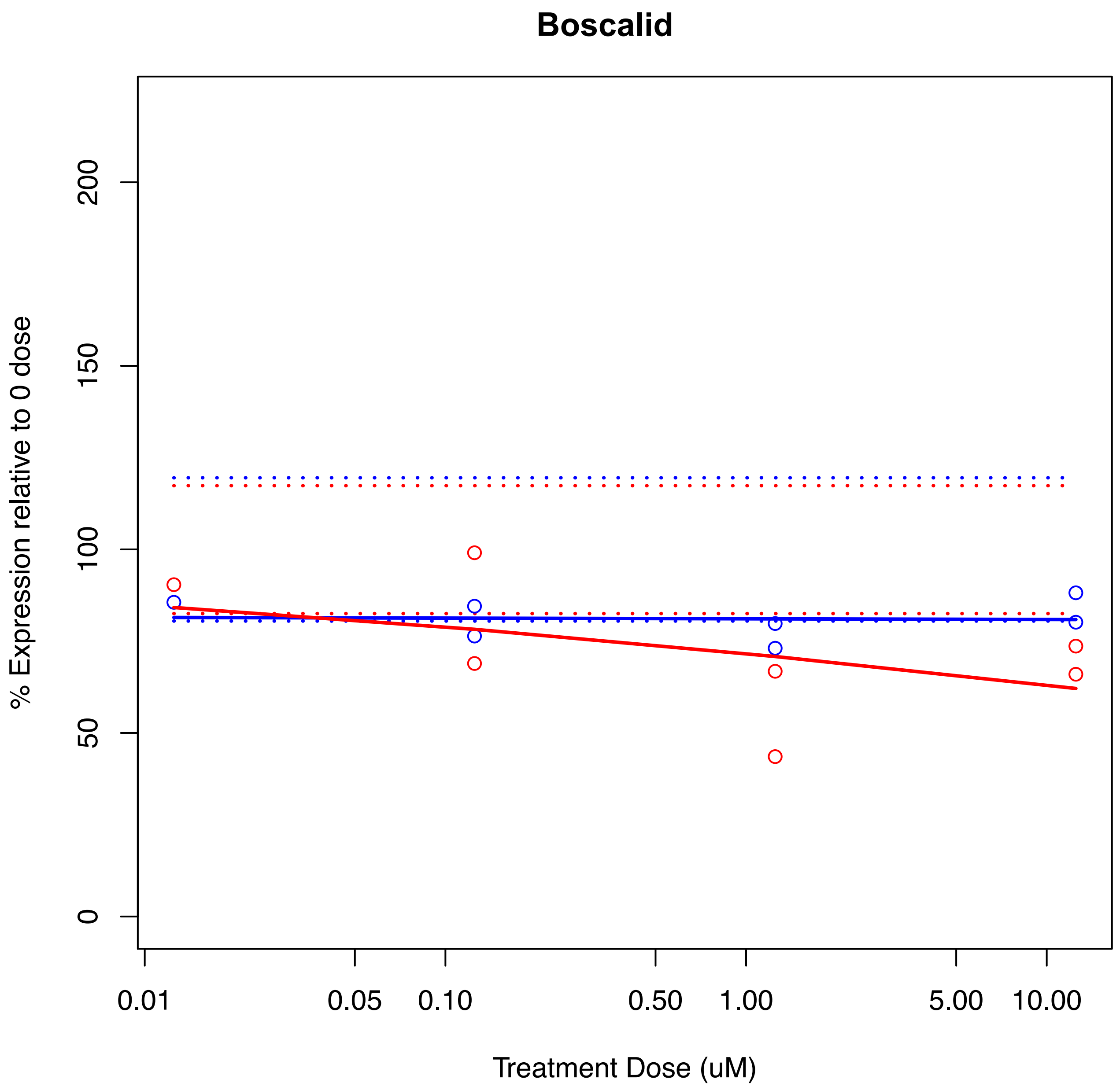
Boscalid
% Expression relative to 0 dose
0
50
100
150
200
0.01
0.05
0.10
0.50
1.00
5.00
10.00
Treatment Dose (uM)

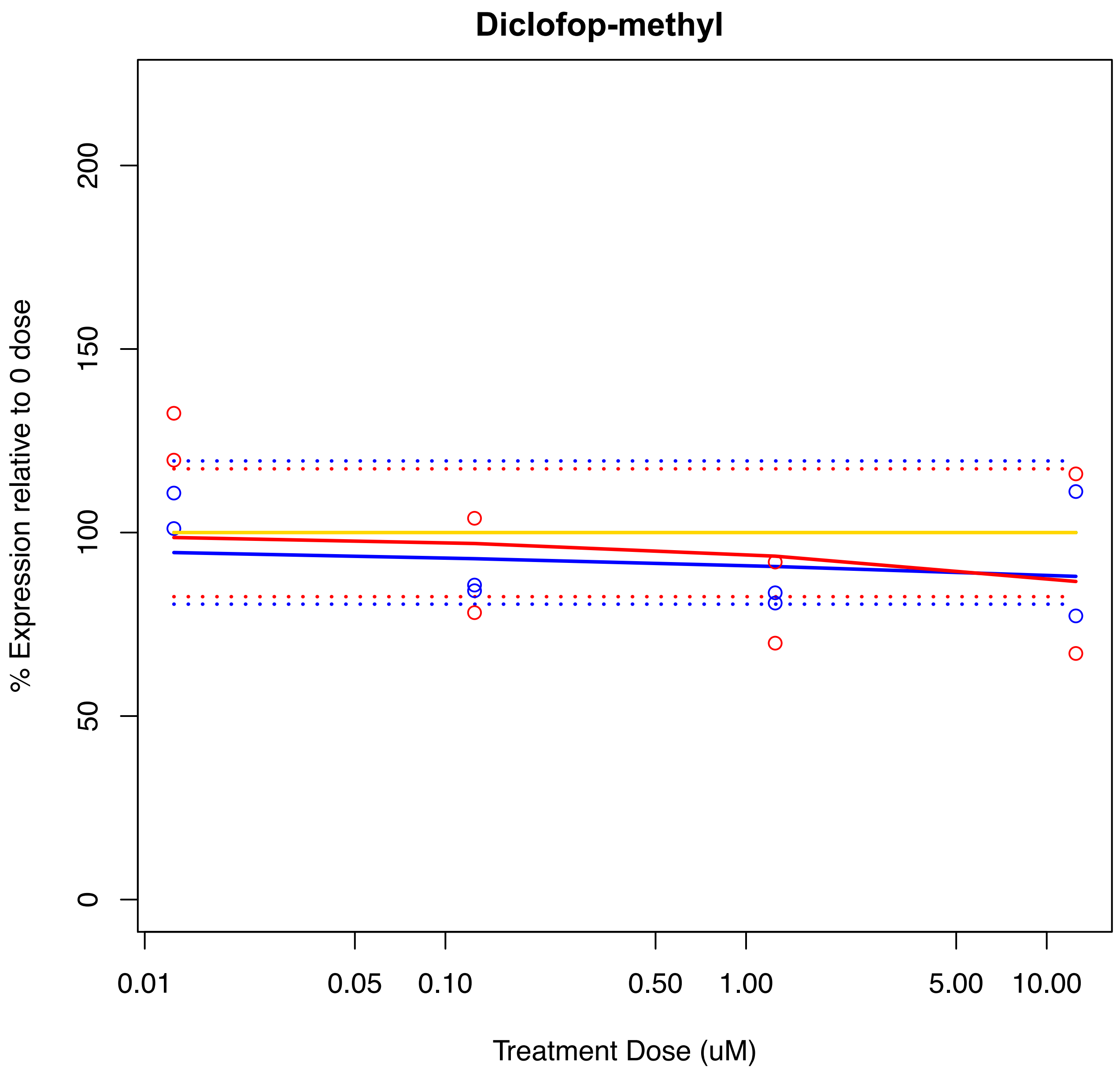
Diclofop-methyl
% Expression relative to 0 dose
0
50
100
150
200
0.01
0.05
0.10
0.50
1.00
5.00
10.00
Treatment Dose (uM)

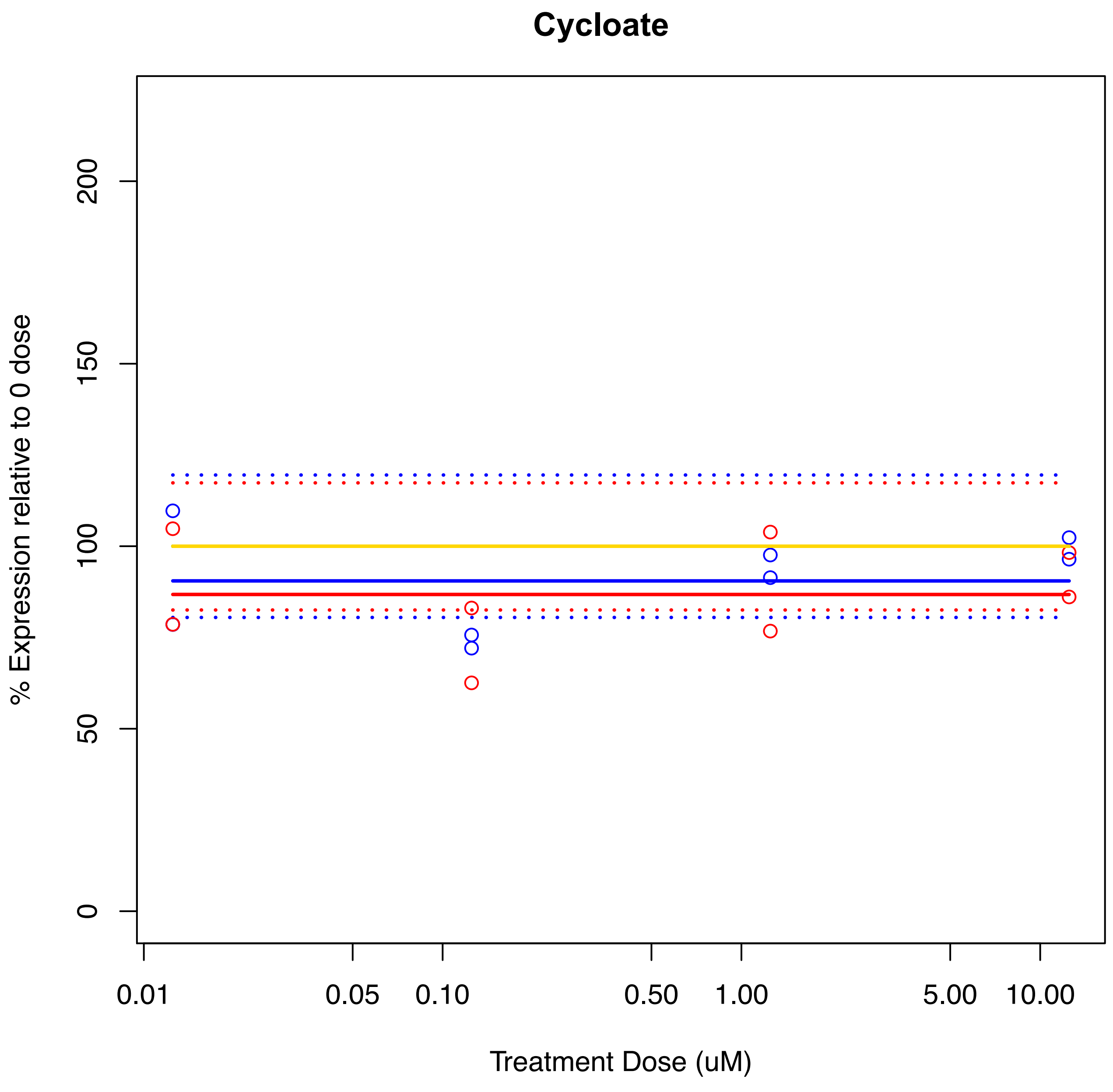
Cycloate
% Expression relative to 0 dose
0
50
100
150
200
0.01
0.05
0.10
0.50
1.00
5.00
10.00
Treatment Dose (uM)

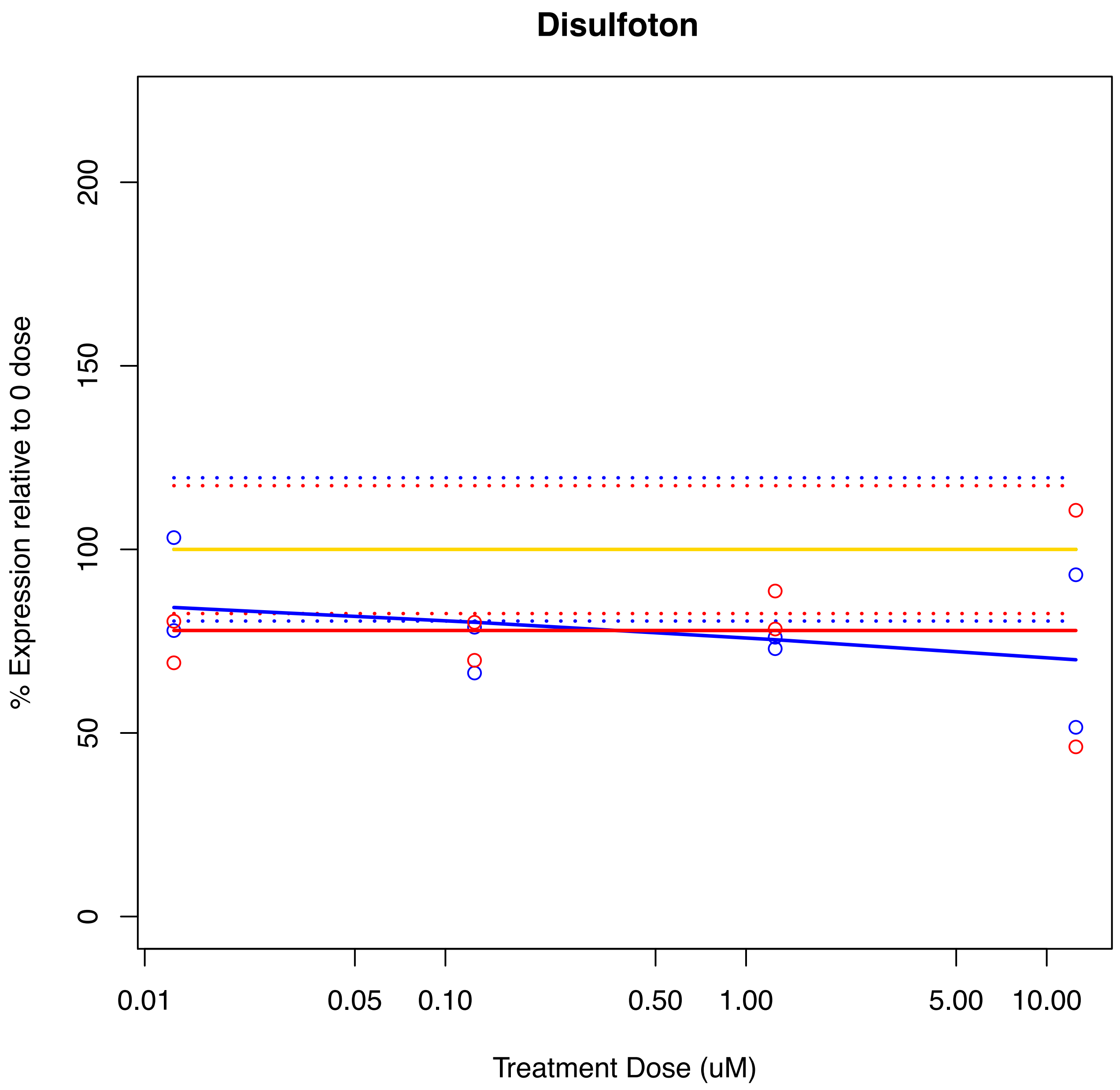

Disulfoton
% Expression relative to 0 dose
0
50
100
150
200
0.01
0.05
0.10
0.50
1.00
5.00
10.00
Treatment Dose (uM)

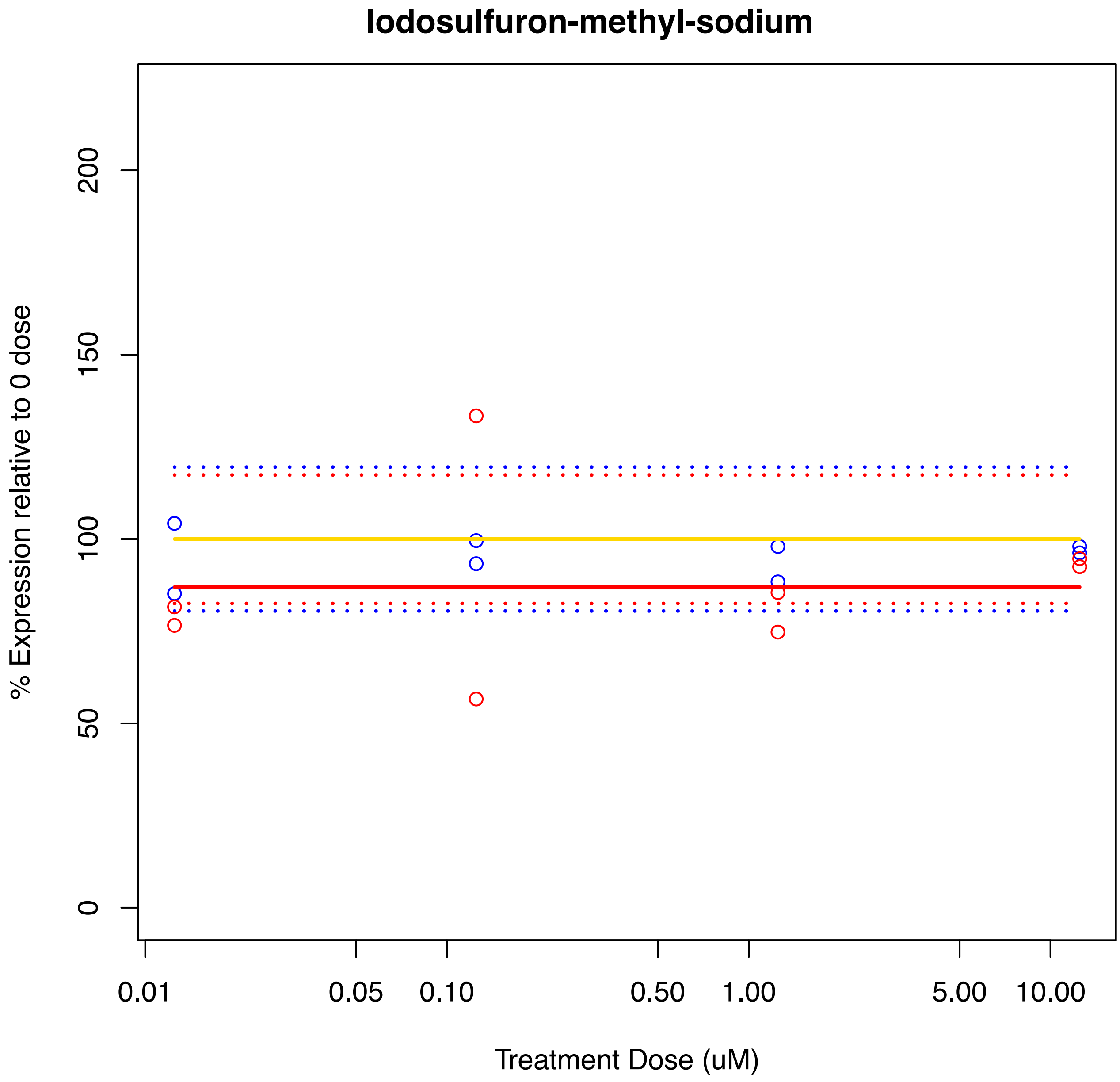

Iodosulfuron-methyl-sodium
% Expression relative to 0 dose
0
50
100
150
200
0.01
0.05
0.10
0.50
1.00
5.00
10.00
Treatment Dose (uM)

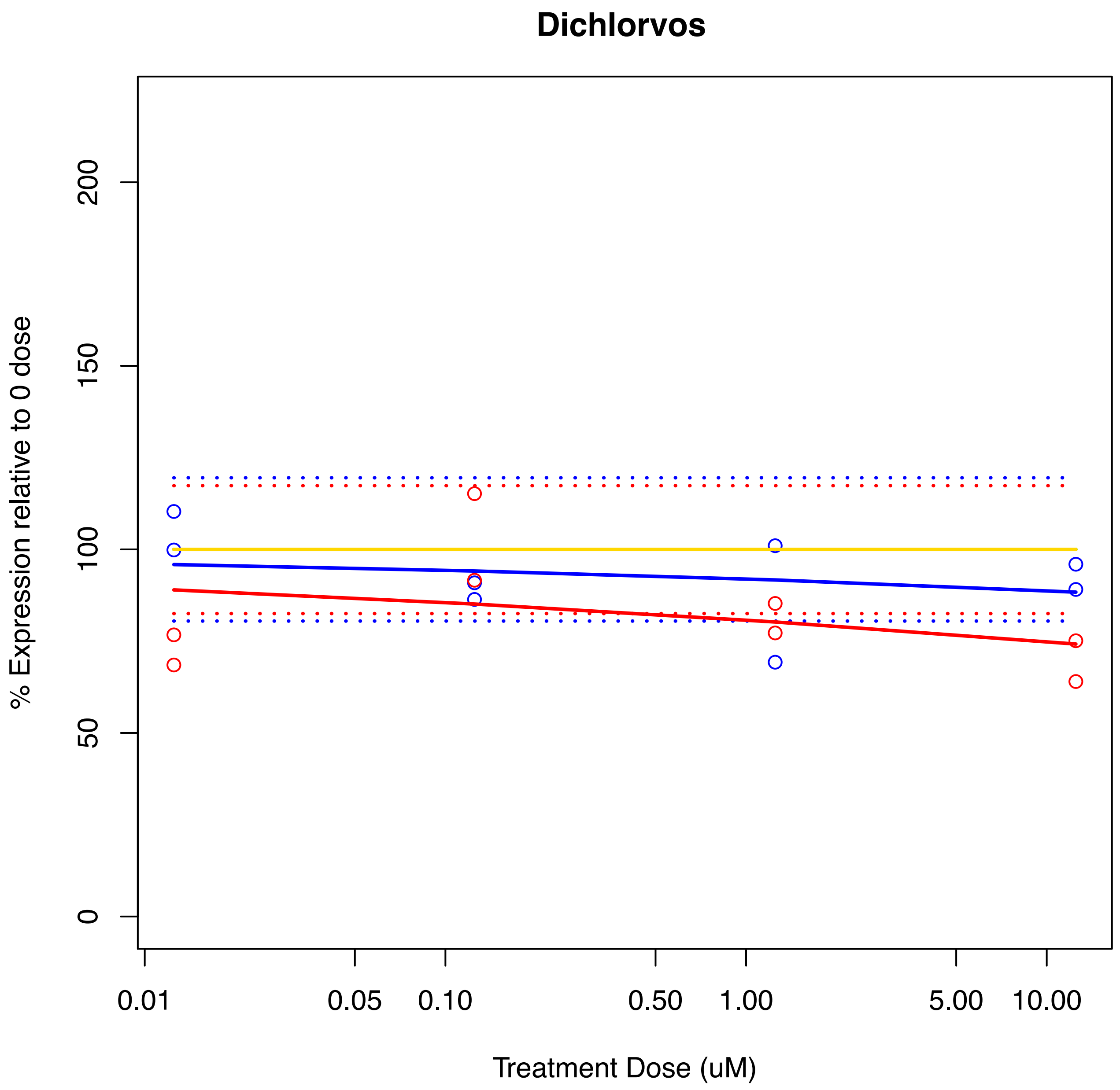

Dichlorvos
% Expression relative to 0 dose
0
50
100
150
200
0.01
0.05
0.10
0.50
1.00
5.00
10.00
Treatment Dose (uM)

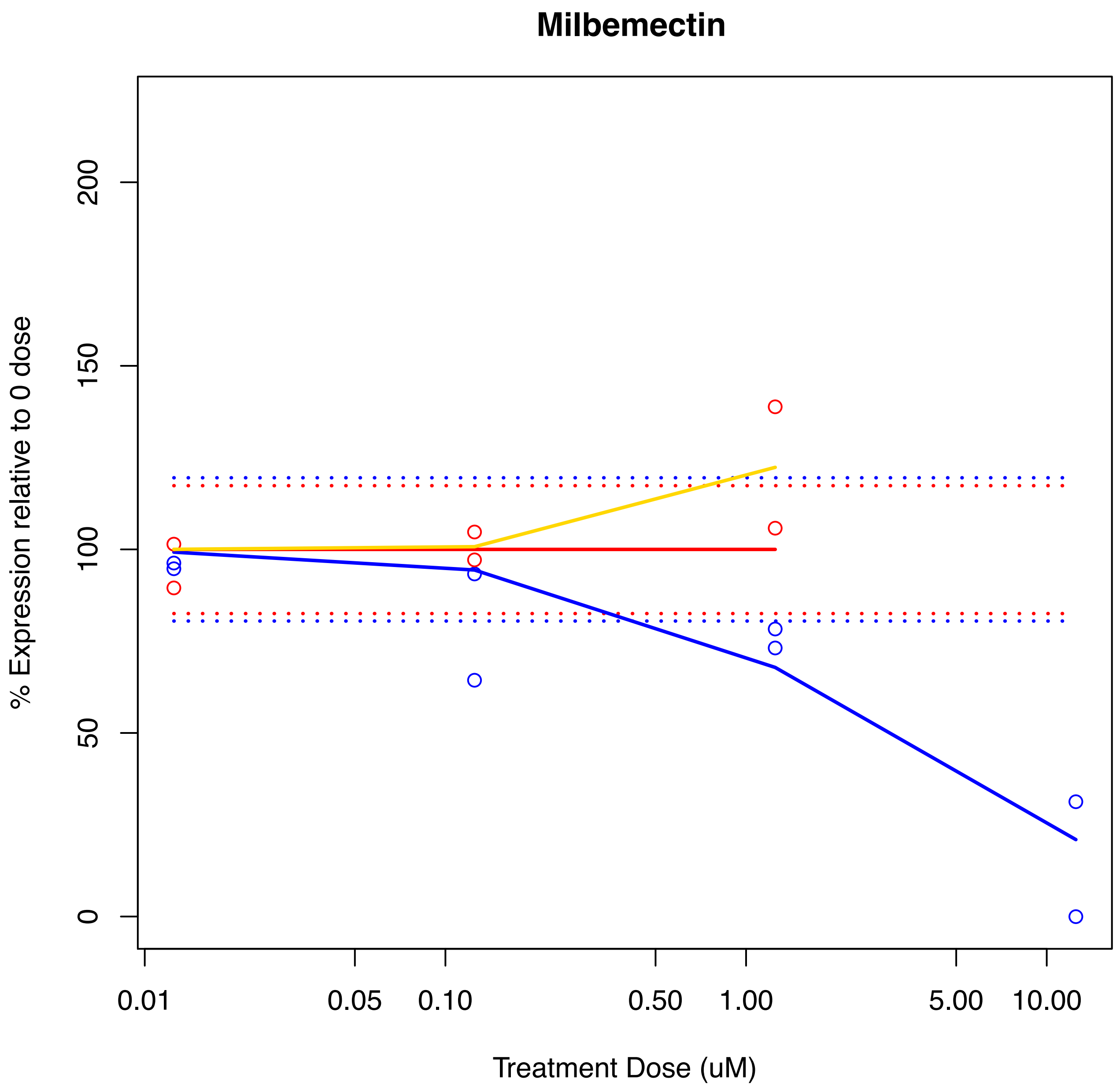
Milbemectin
% Expression relative to 0 dose
0
50
100
150
200
0.01
0.05
0.10
0.50
1.00
5.00
10.00
Treatment Dose (uM)

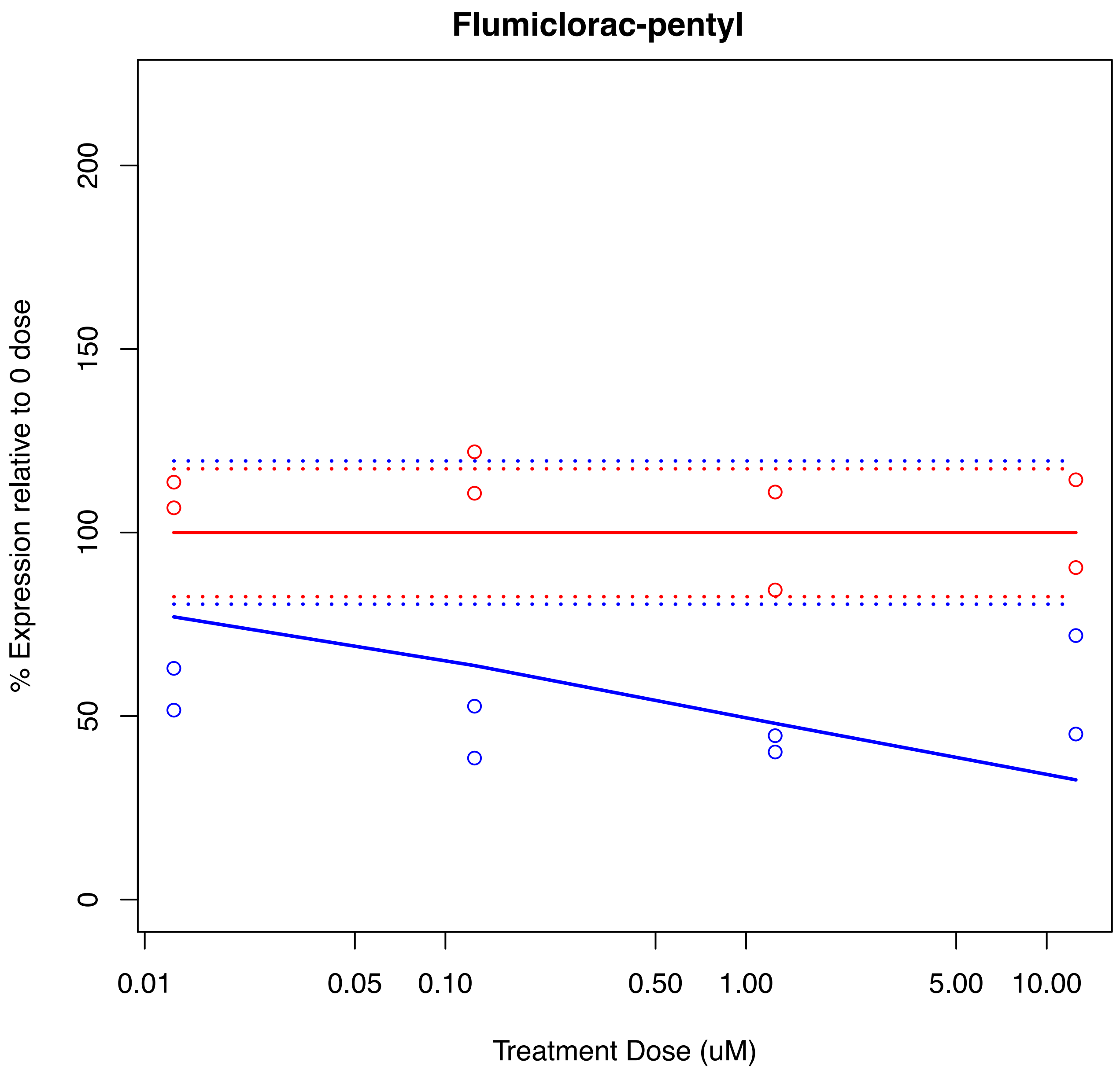
Flumiclorac-pentyl
% Expression relative to 0 dose
0
50
100
150
200
0.01
0.05
0.10
0.50
1.00
5.00
10.00
Treatment Dose (uM)

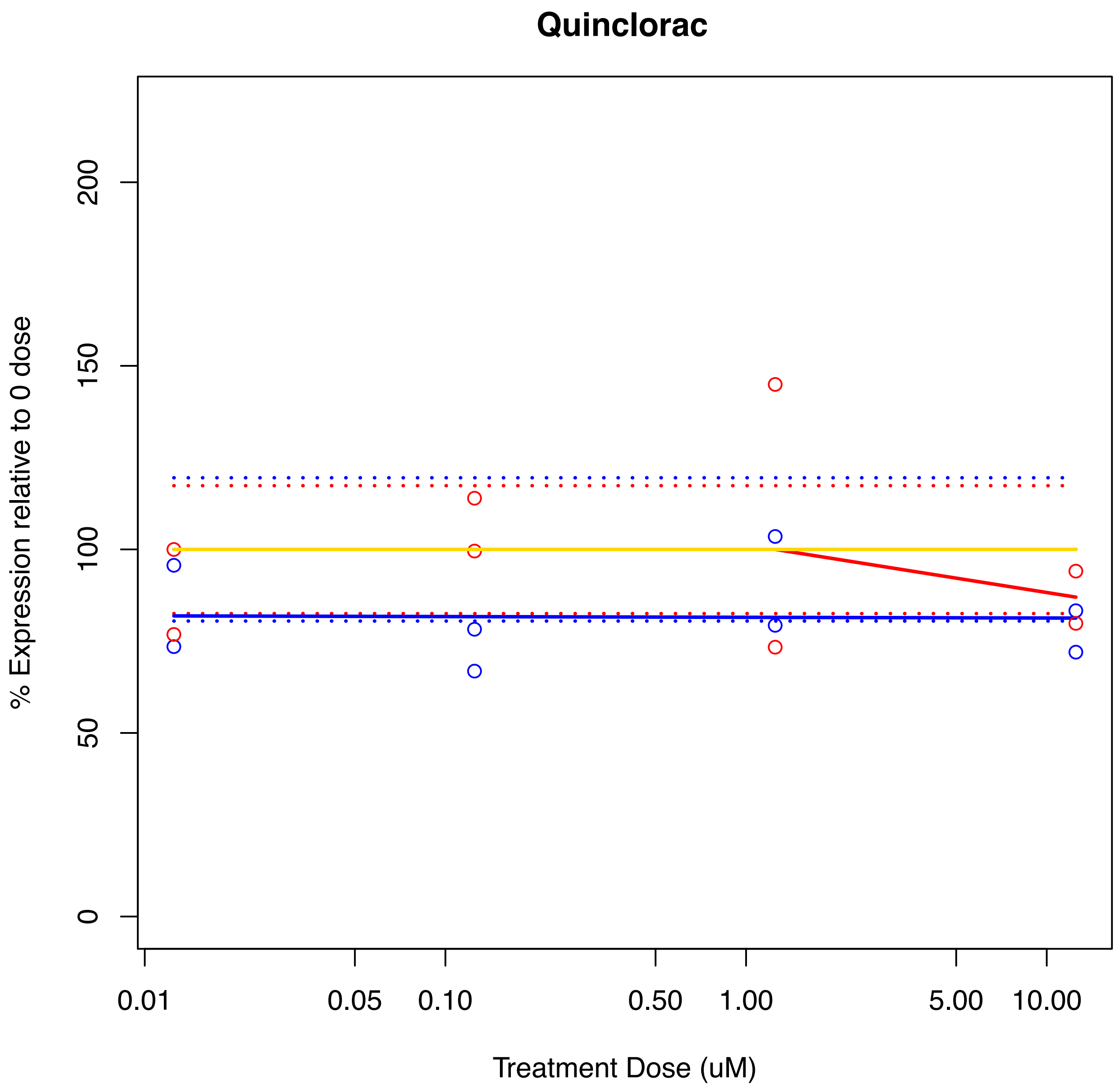

Quinclorac
% Expression relative to 0 dose
0
50
100
150
200
0.01
0.05
0.10
0.50
1.00
5.00
10.00
Treatment Dose (uM)

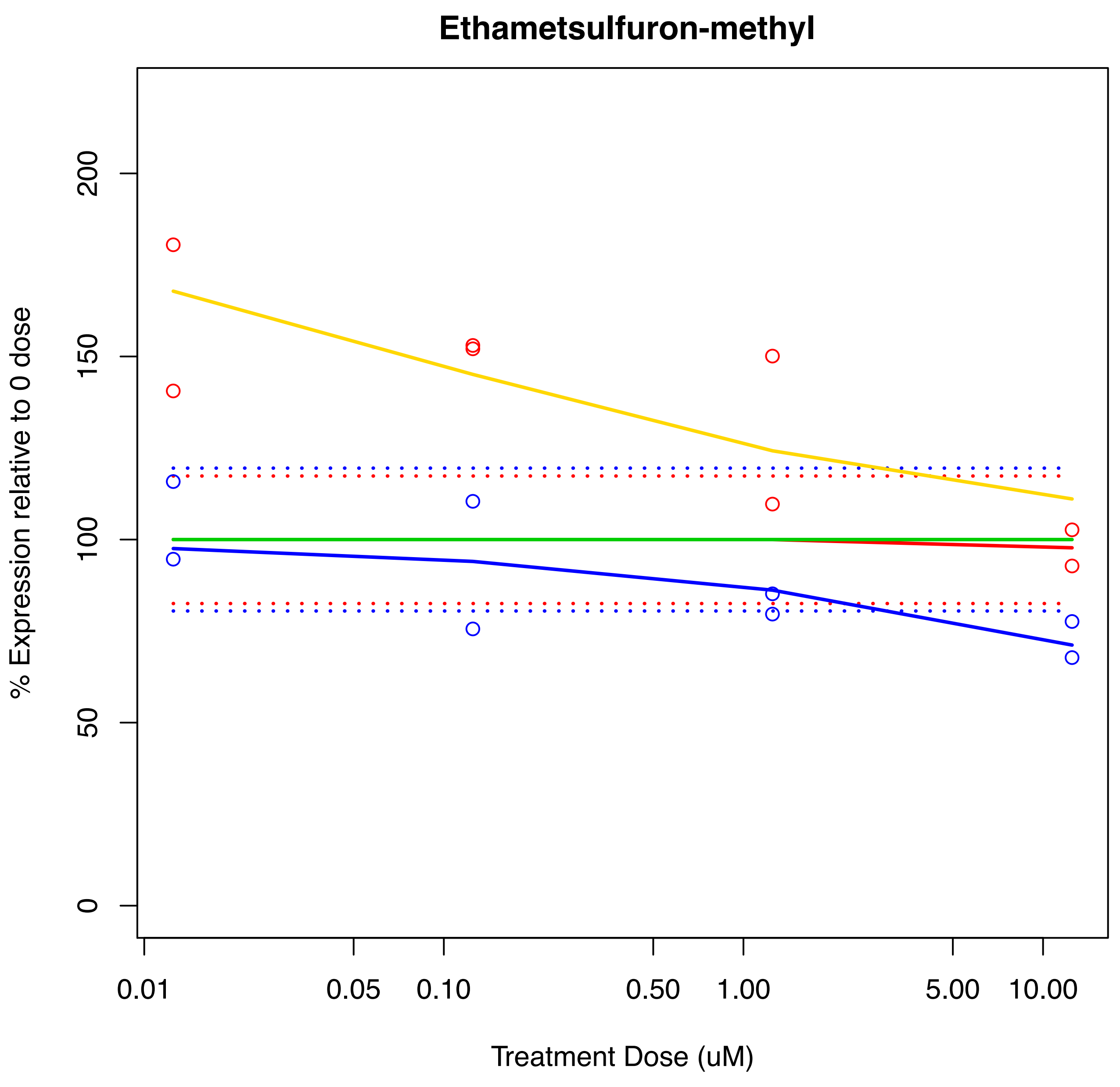

Ethametsulfuron-methyl
% Expression relative to 0 dose
0
50
100
150
200
0.01
0.05
0.10
0.50
1.00
5.00
10.00
Treatment Dose (uM)

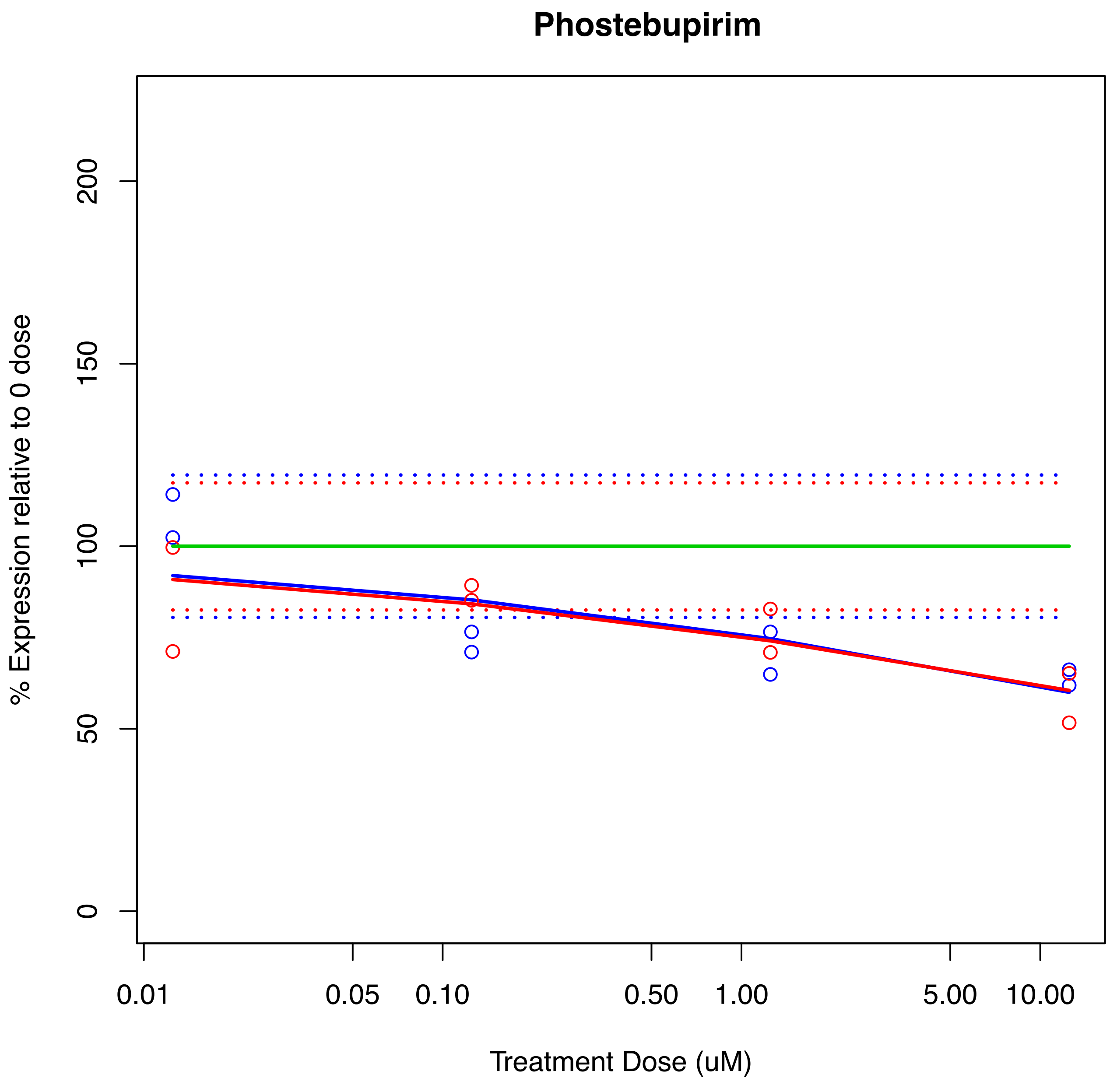

Phostebupirim
% Expression relative to 0 dose
0
50
100
150
200
0.01
0.05
0.10
0.50
1.00
5.00
10.00
Treatment Dose (uM)

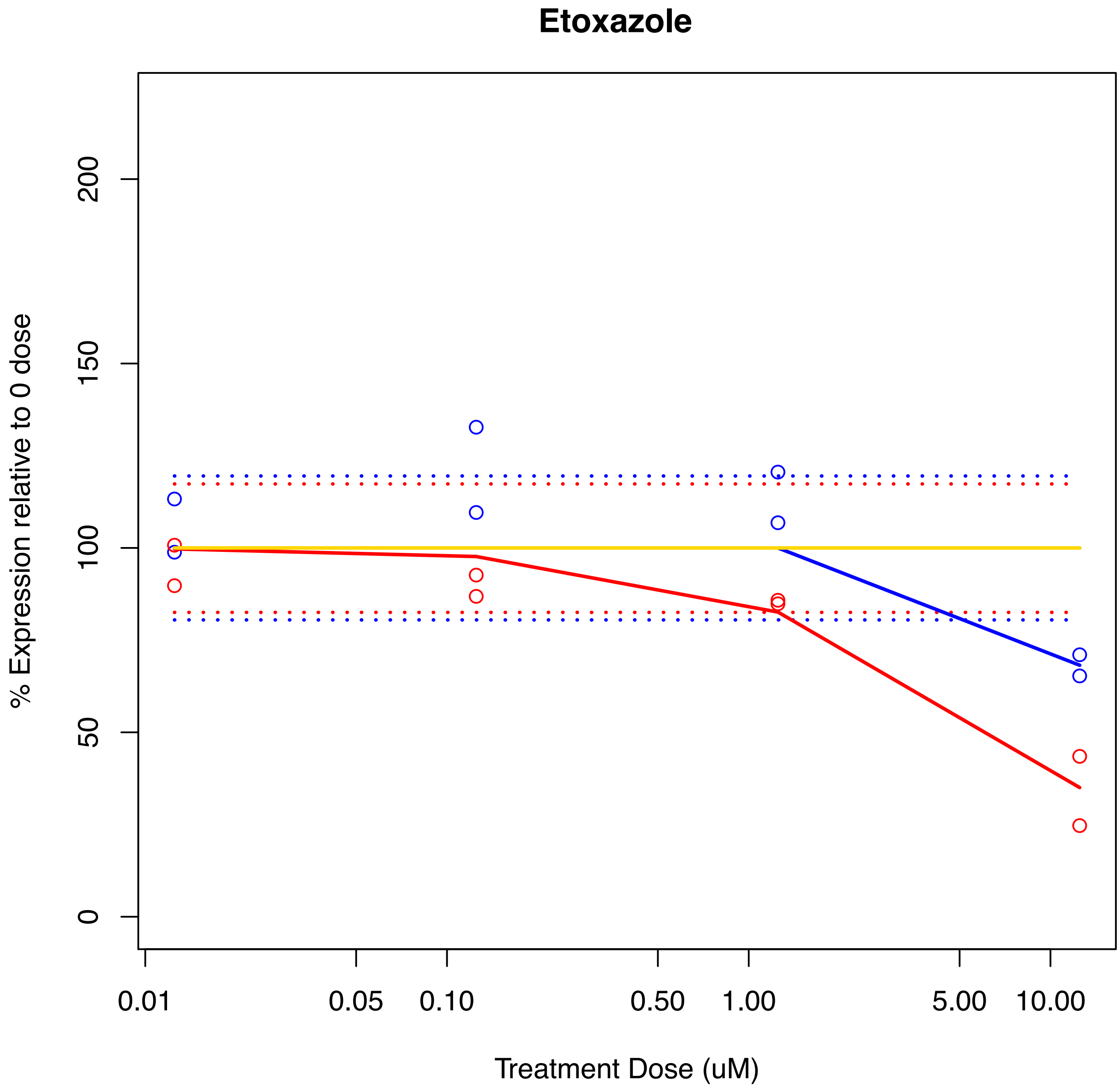

Etoxazole
% Expression relative to 0 dose
0
50
100
150
200
0.01
0.05
0.10
0.50
1.00
5.00
10.00
Treatment Dose (uM)

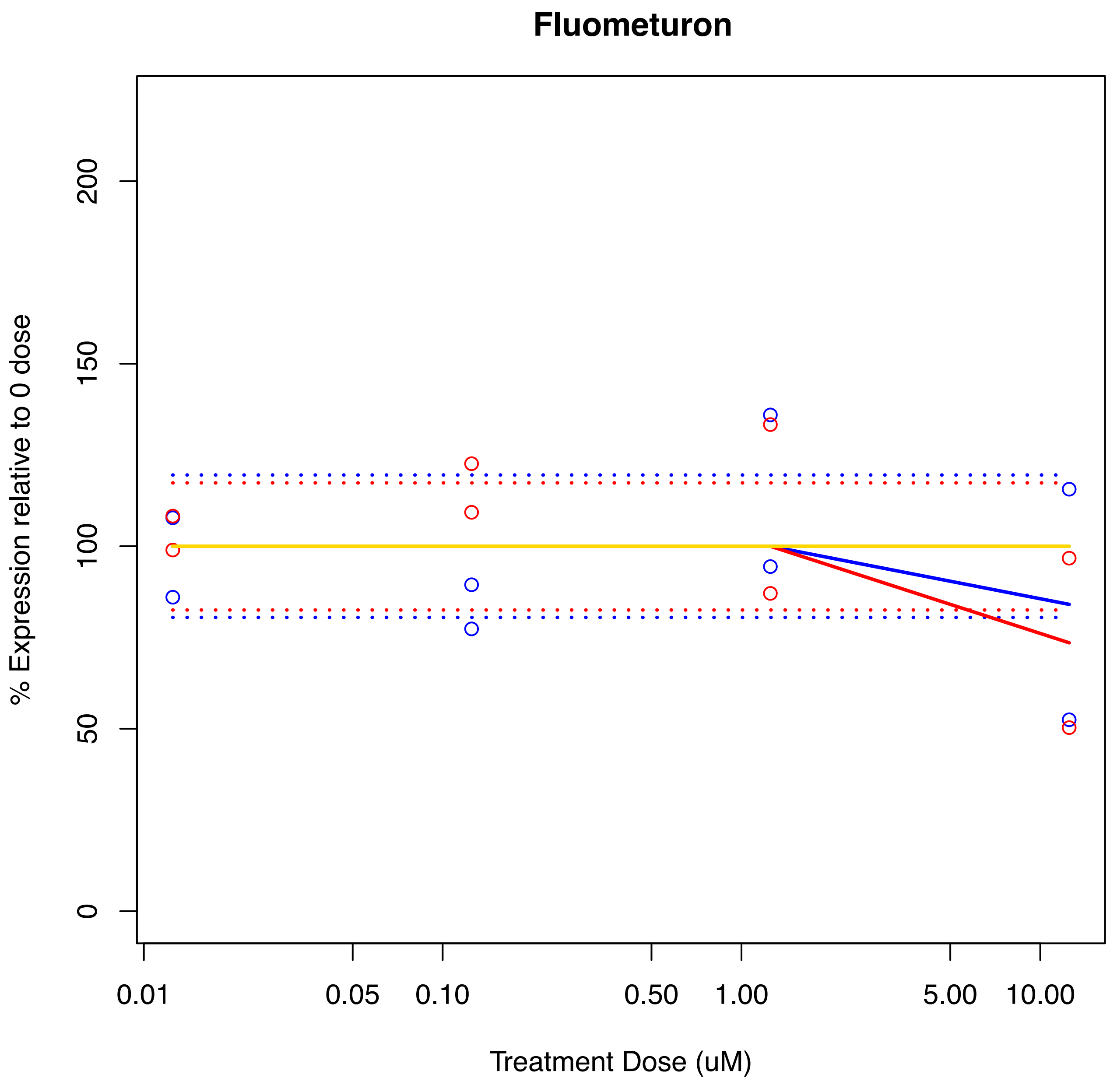

Fluometuron
% Expression relative to 0 dose
0
50
100
150
200
0.01
0.05
0.10
0.50
1.00
5.00
10.00
Treatment Dose (uM)

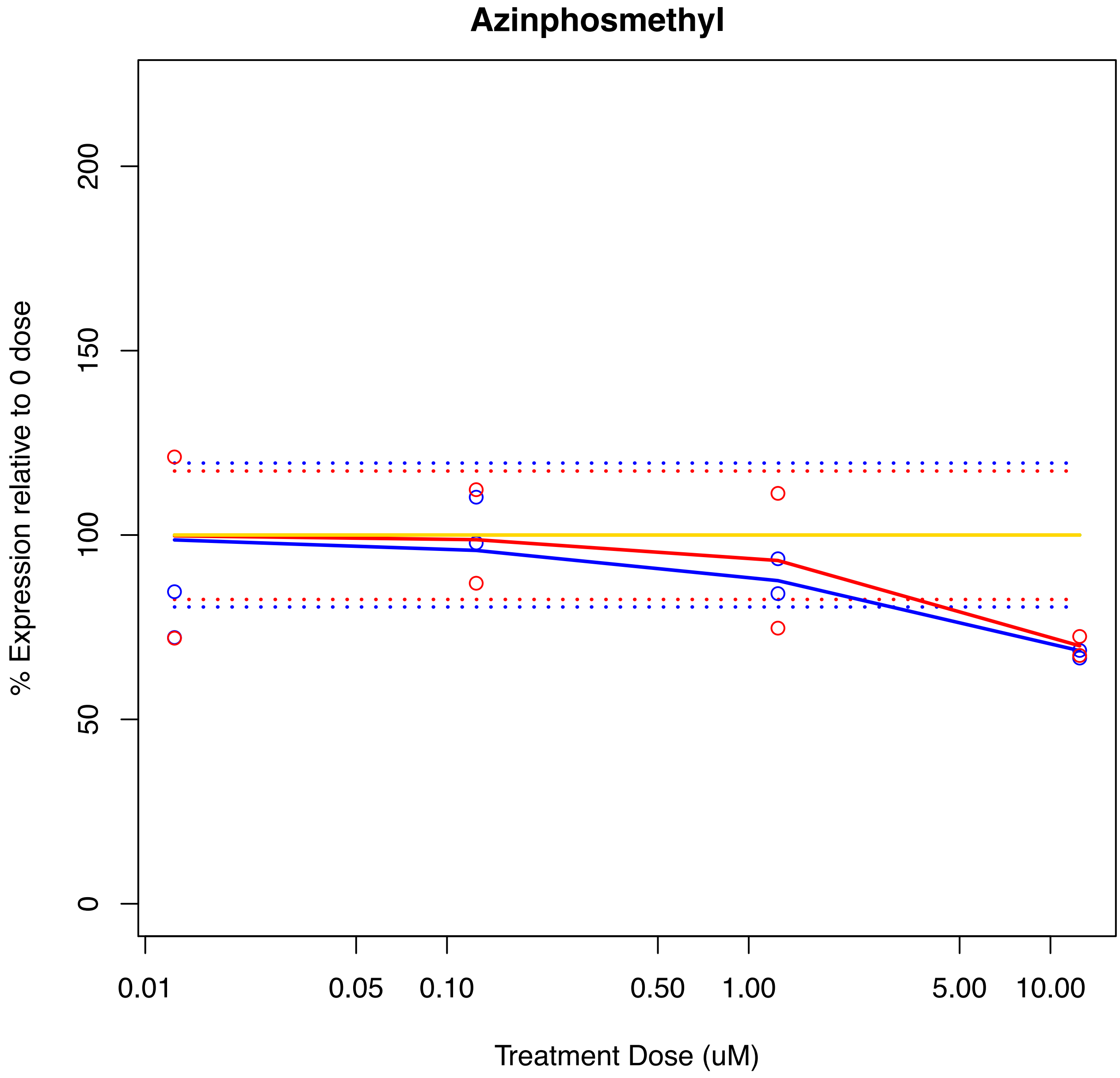

Azinphosmethyl
% Expression relative to 0 dose
0
50
100
150
200
0.01
0.05
0.10
0.50
1.00
5.00
10.00
Treatment Dose (uM)

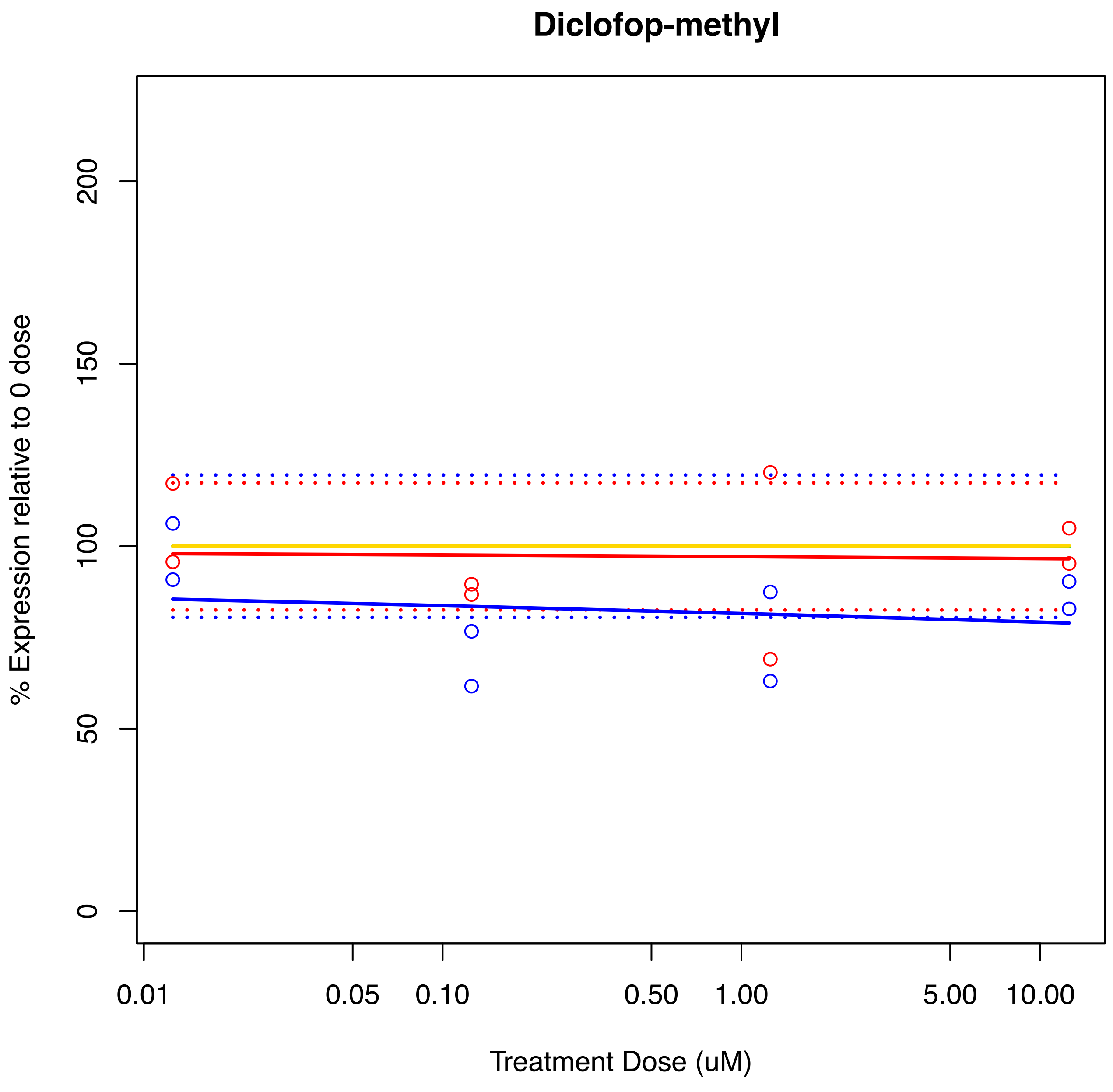
Diclofop-methyl
% Expression relative to 0 dose
Treatment Dose (uM)
0
50
100
150
200
0.01
0.05
0.10
0.50
1.00
5.00
10.00

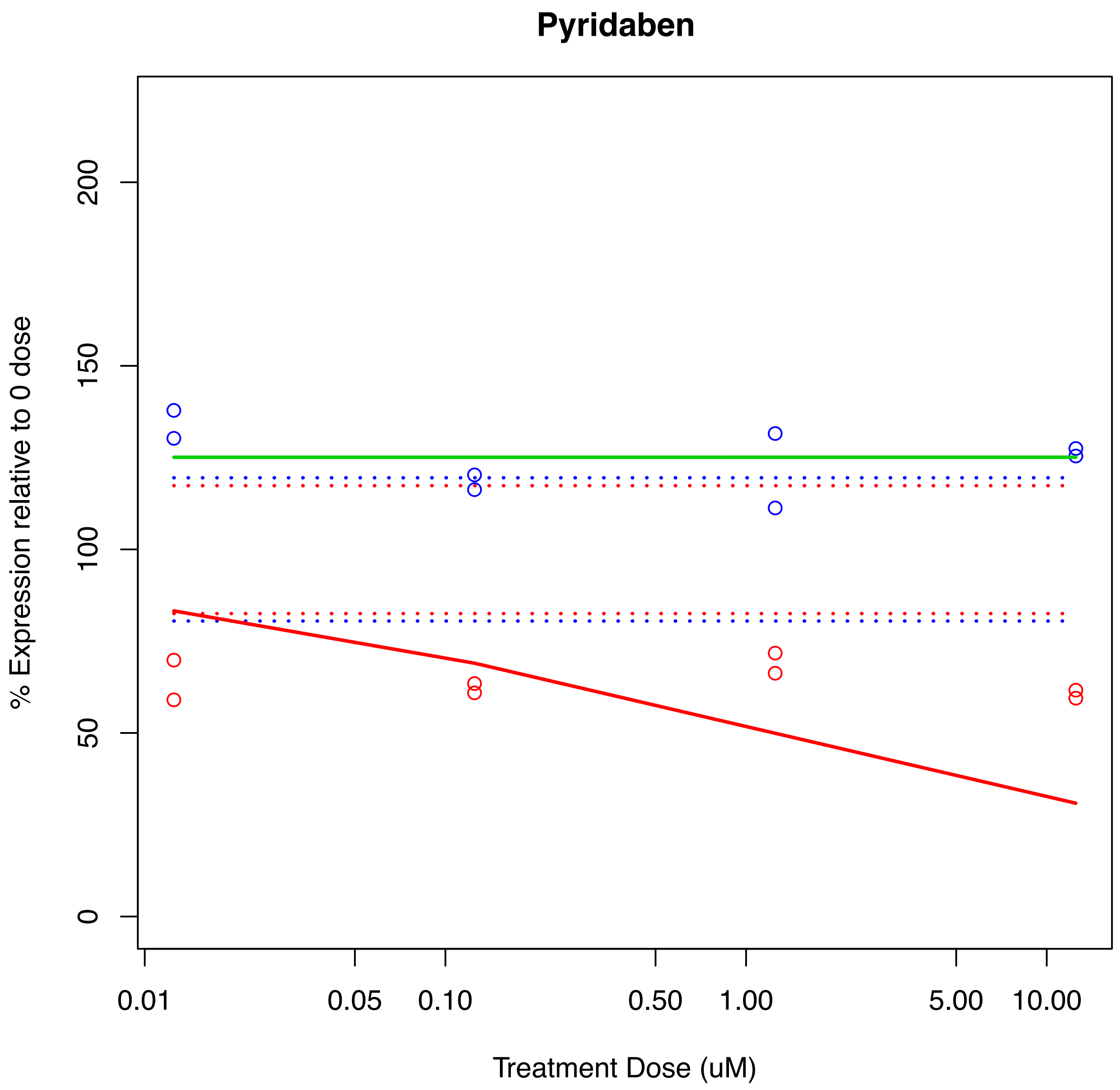
Pyridaben
% Expression relative to 0 dose
0
50
100
150
200
0.01
0.05
0.10
0.50
1.00
5.00
10.00
Treatment Dose (uM)

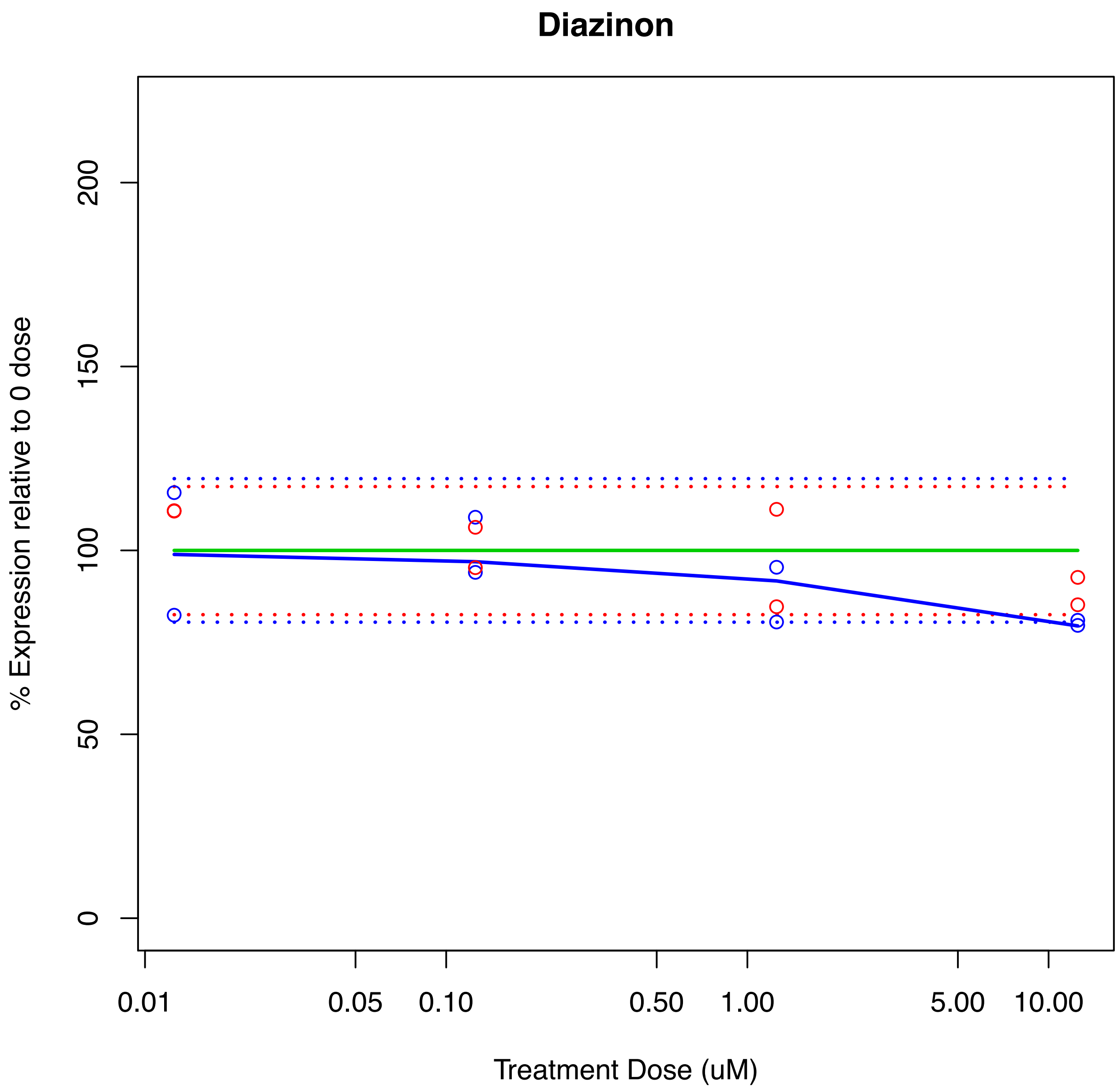

Diazinon
% Expression relative to 0 dose
0
50
100
150
200
0.01
0.05
0.10
0.50
1.00
5.00
10.00
Treatment Dose (uM)

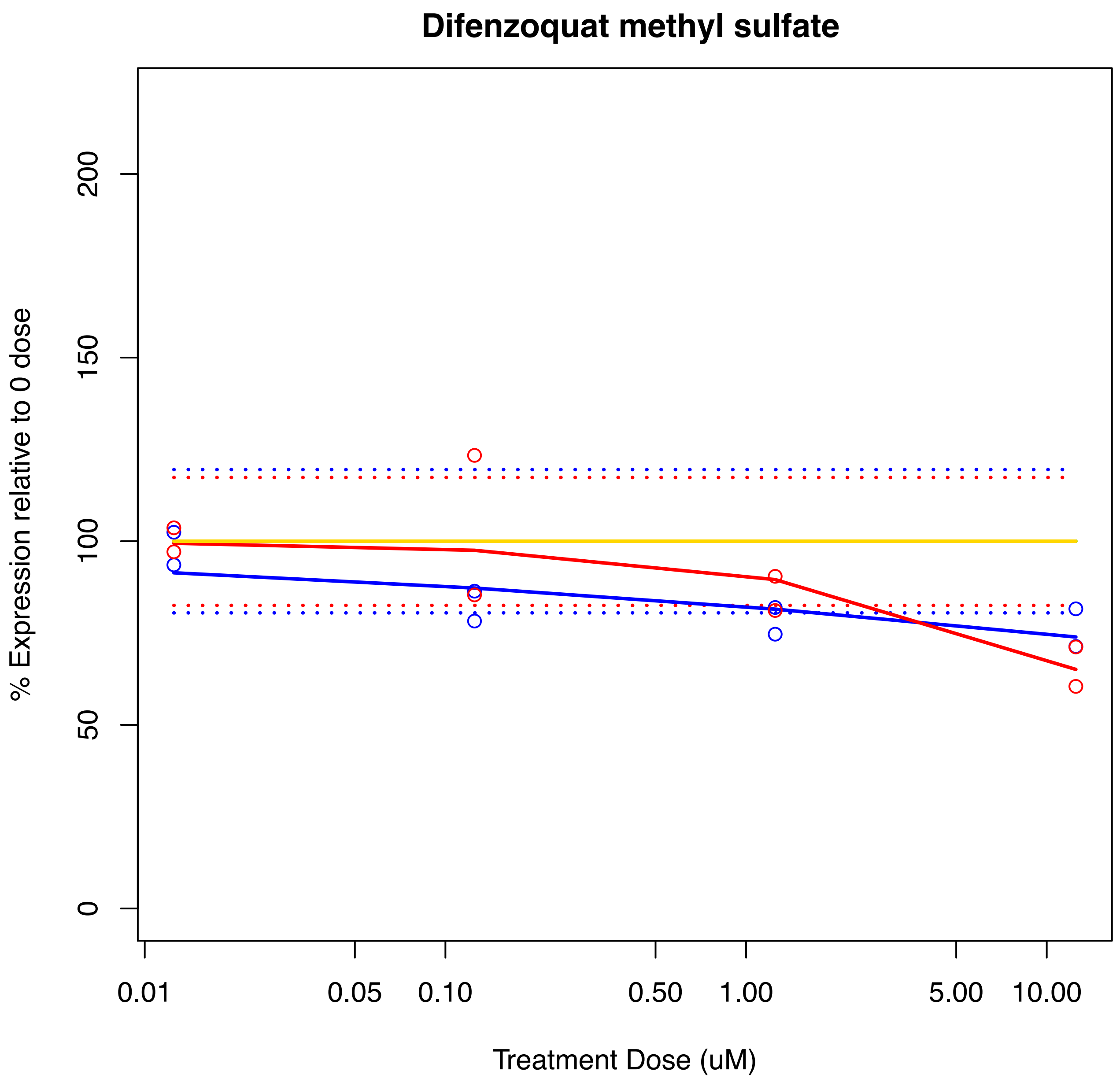
Difenzoquat methyl sulfate
% Expression relative to 0 dose
0
50
100
150
200
0.01
0.05
0.10
0.50
1.00
5.00
10.00
Treatment Dose (uM)

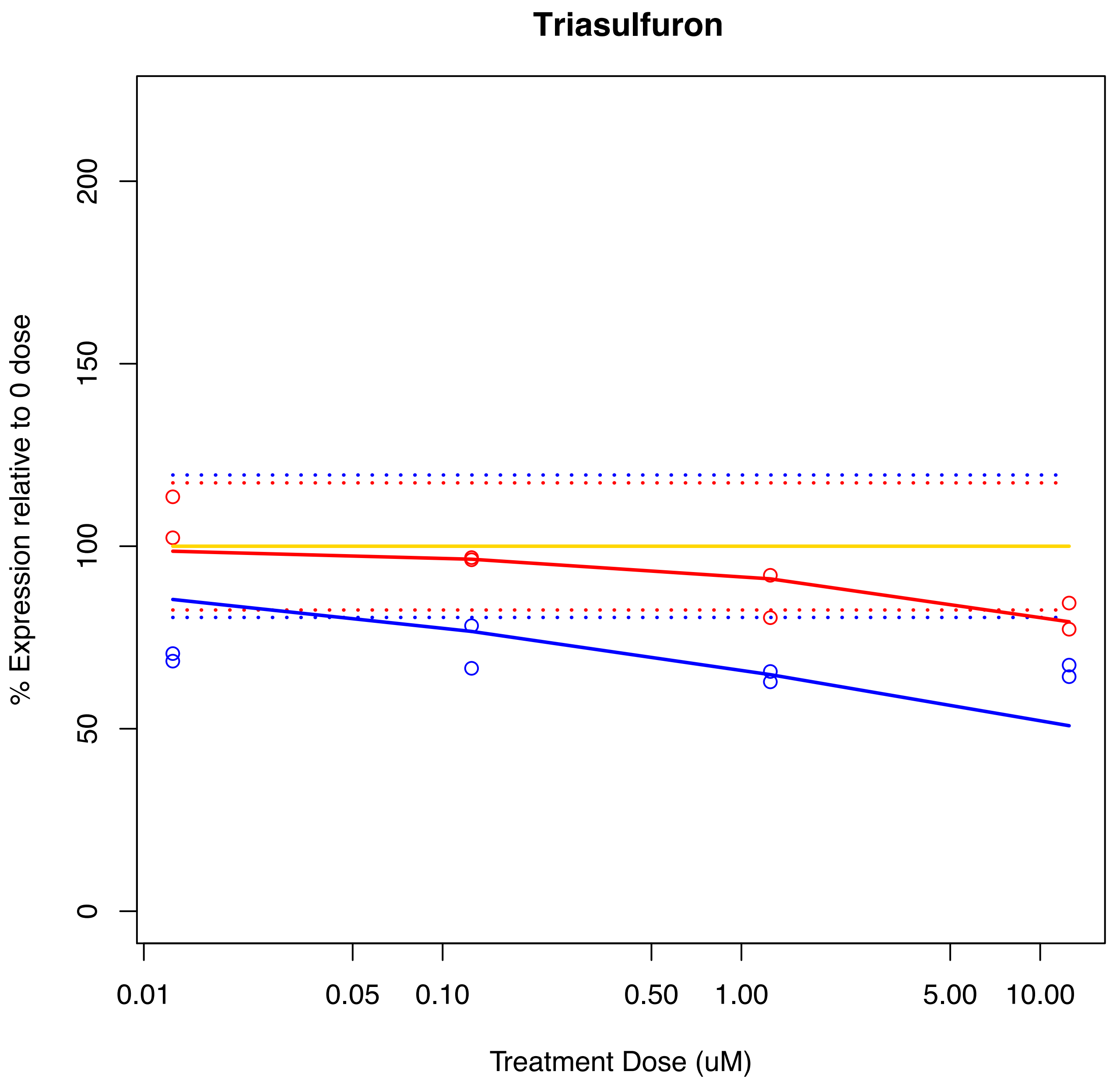

Triasulfuron
% Expression relative to 0 dose
0
50
100
150
200
0.01
0.05
0.10
0.50
1.00
5.00
10.00
Treatment Dose (uM)

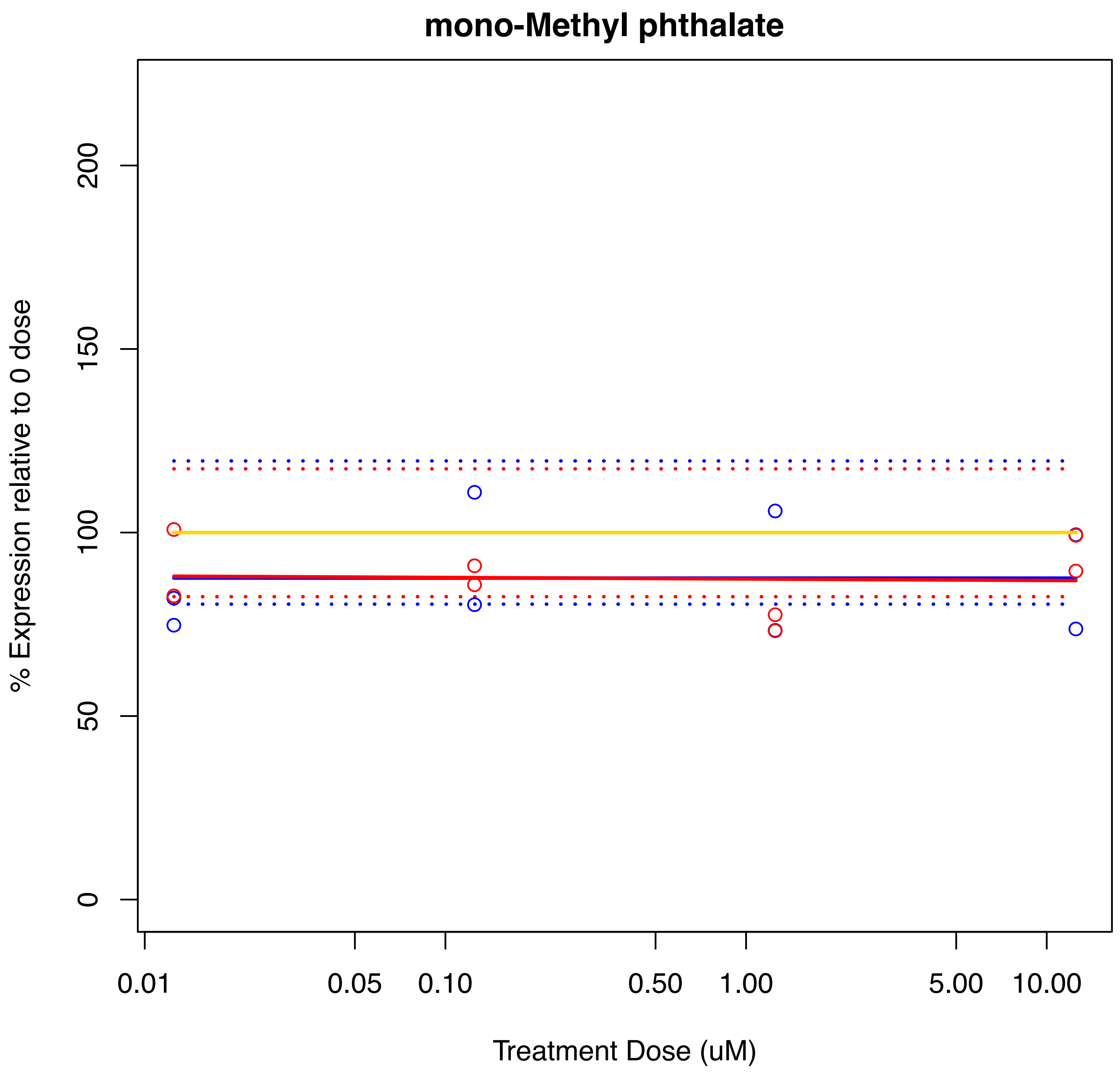

mono-Methyl phthalate
% Expression relative to 0 dose
0
50
100
150
200
0.01
0.05
0.10
0.50
1.00
5.00
10.00
Treatment Dose (uM)

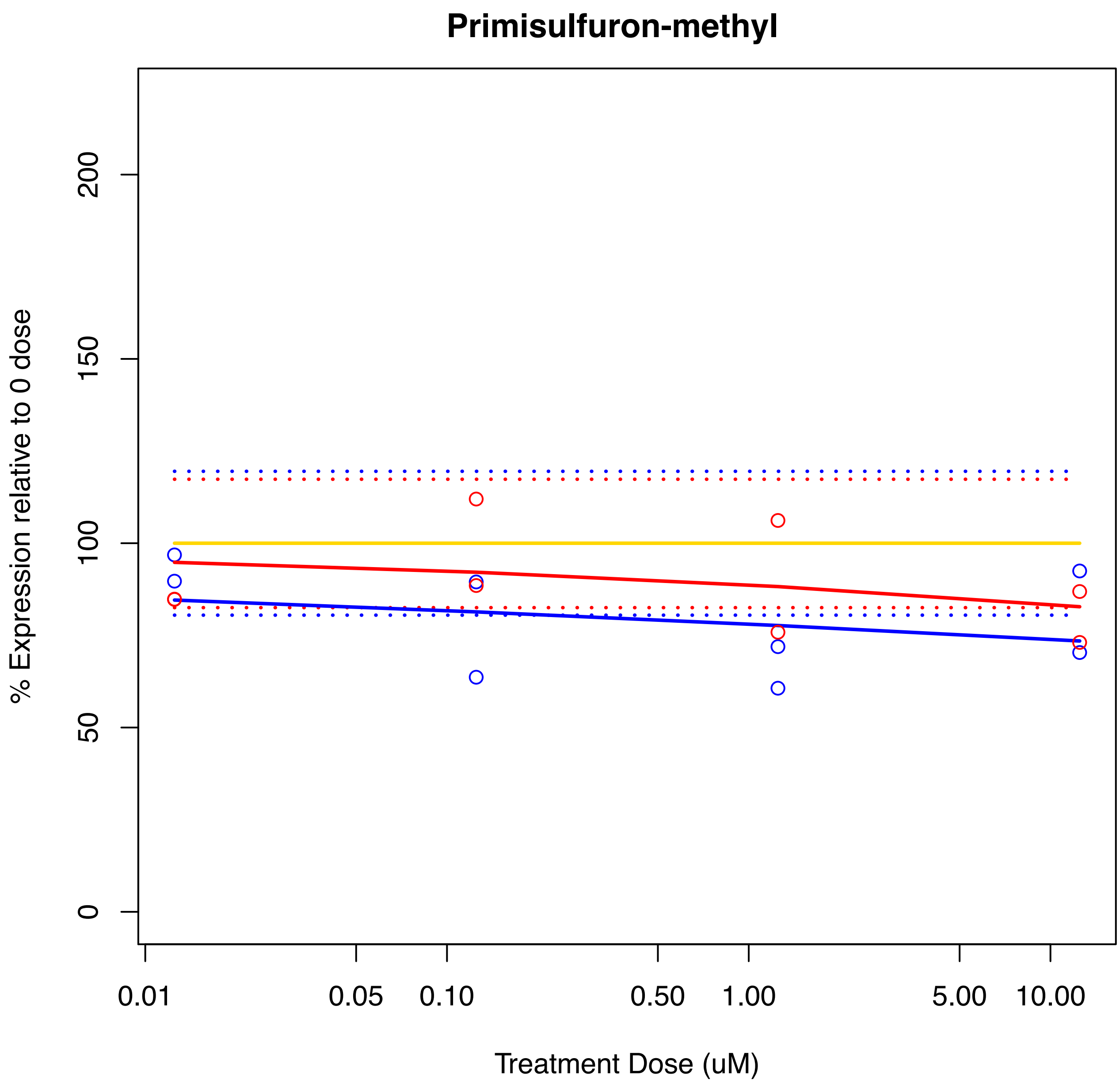

Primisulfuron-methyl
% Expression relative to 0 dose
0
50
100
150
200
0.01
0.05
0.10
0.50
1.00
5.00
10.00
Treatment Dose (uM)

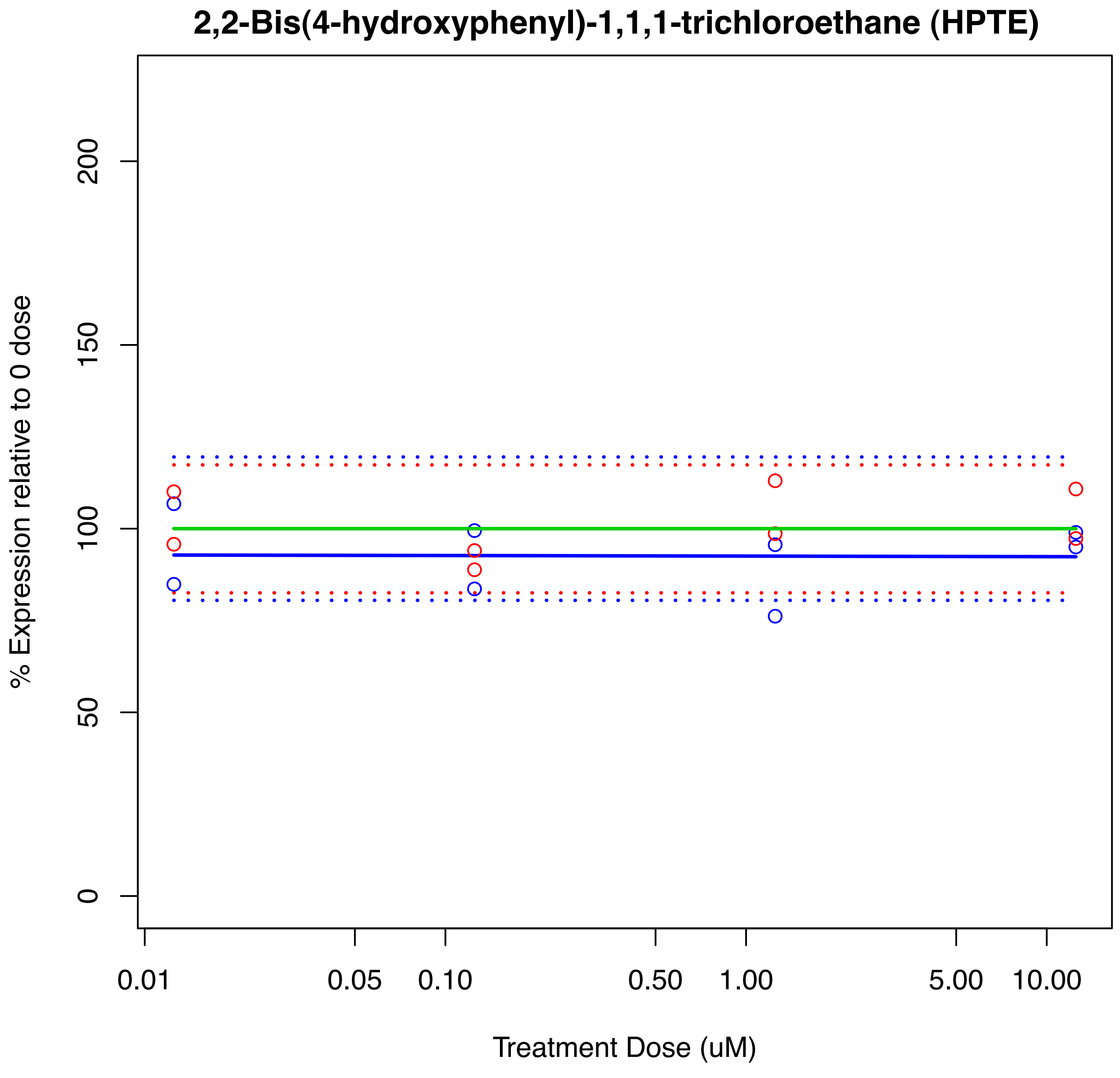
2,2-Bis(4-hydroxyphenyl)-1,1,1-trichloroethane (HPTE)
% Expression relative to 0 dose
0
50
100
150
200
0.01
0.05
0.10
0.50
1.00
5.00
10.00
Treatment Dose (uM)

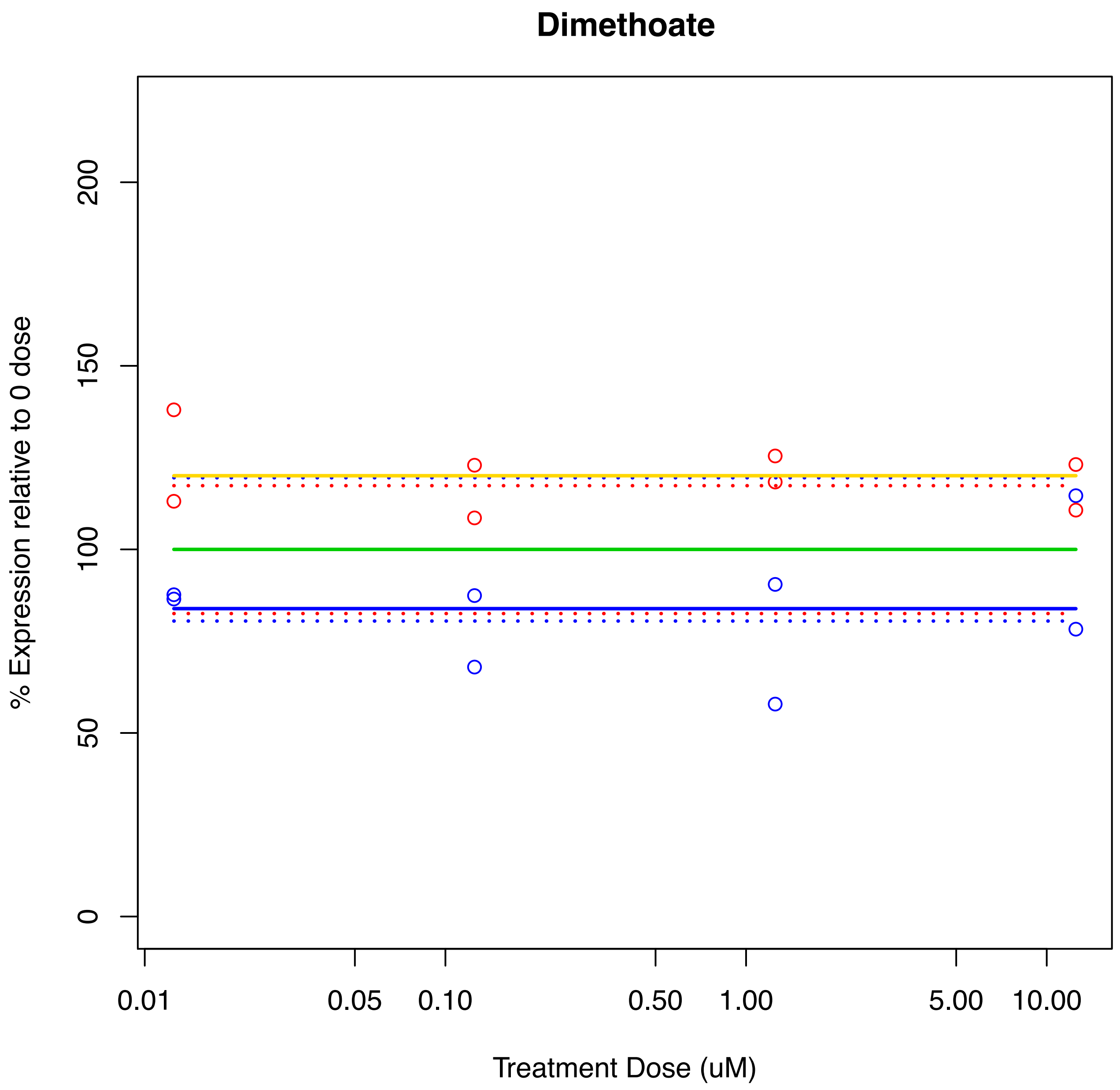

Dimethoate
% Expression relative to 0 dose
0
50
100
150
200
0.01
0.05
0.10
0.50
1.00
5.00
10.00
Treatment Dose (uM)

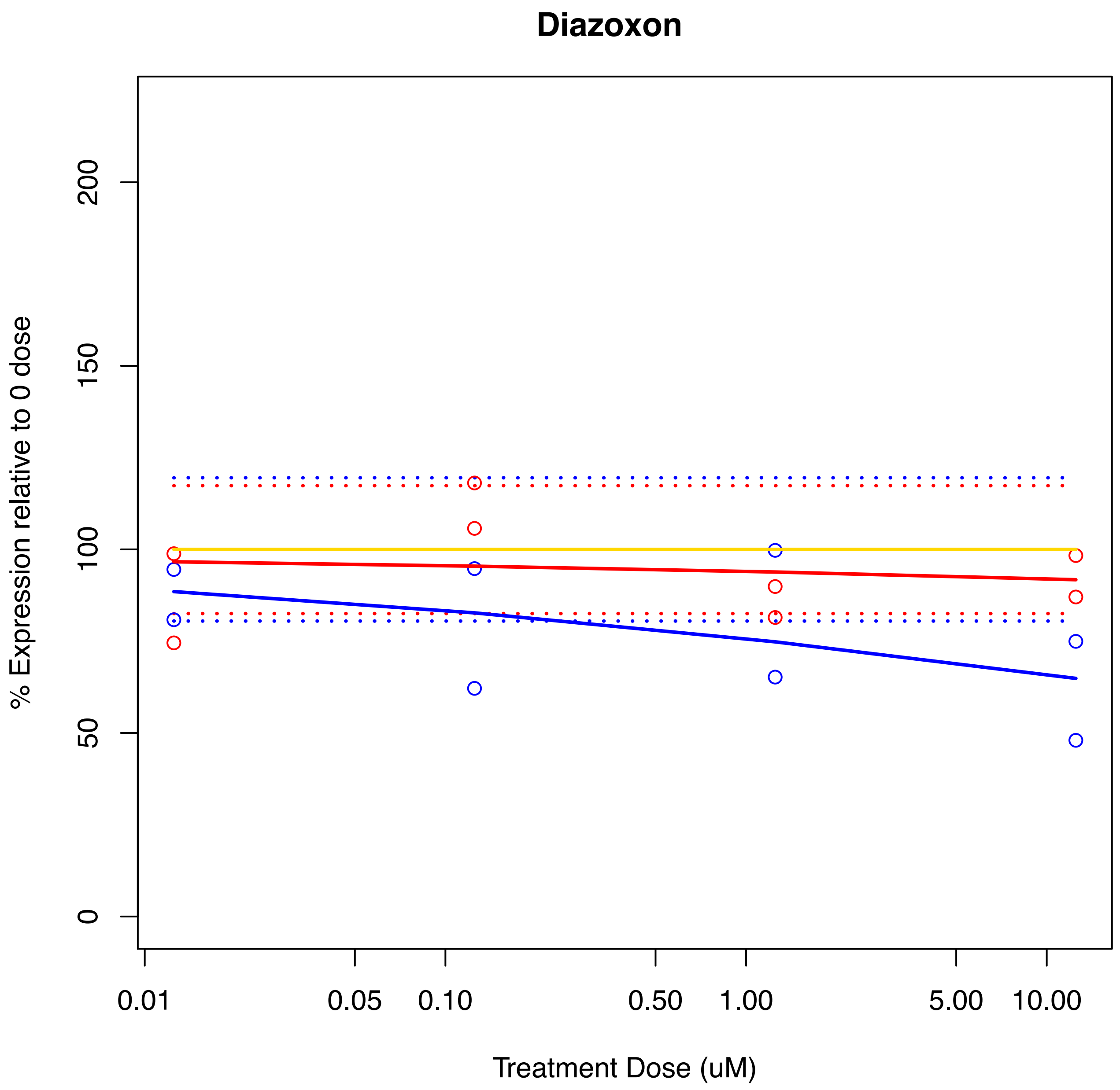

Diazoxon
% Expression relative to 0 dose
0
50
100
150
200
0.01
0.05
0.10
0.50
1.00
5.00
10.00
Treatment Dose (uM)

S-sec-Butyl O-ethyl (2-oxo-3-thiazolidinyl)phosphonothioate

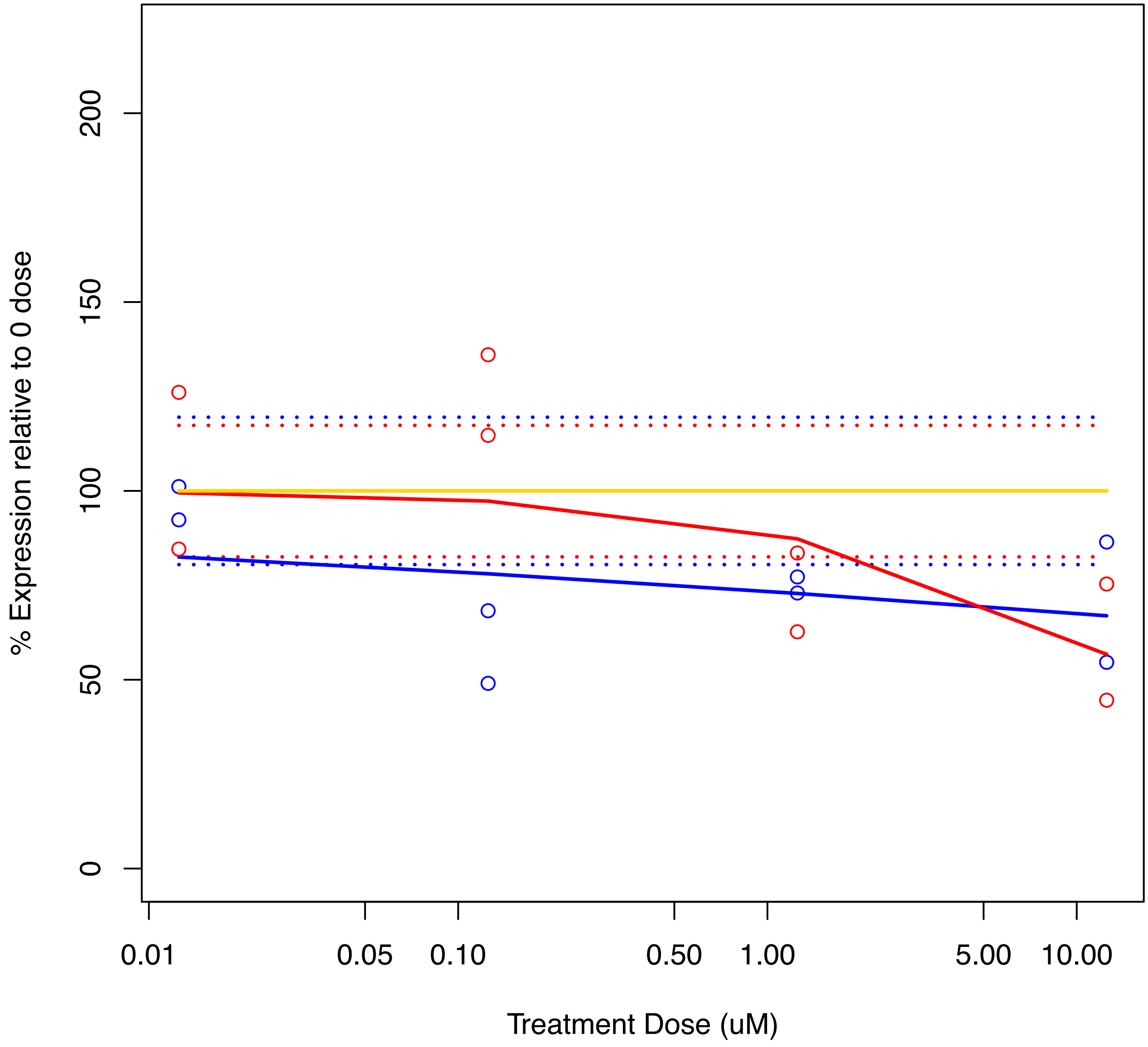

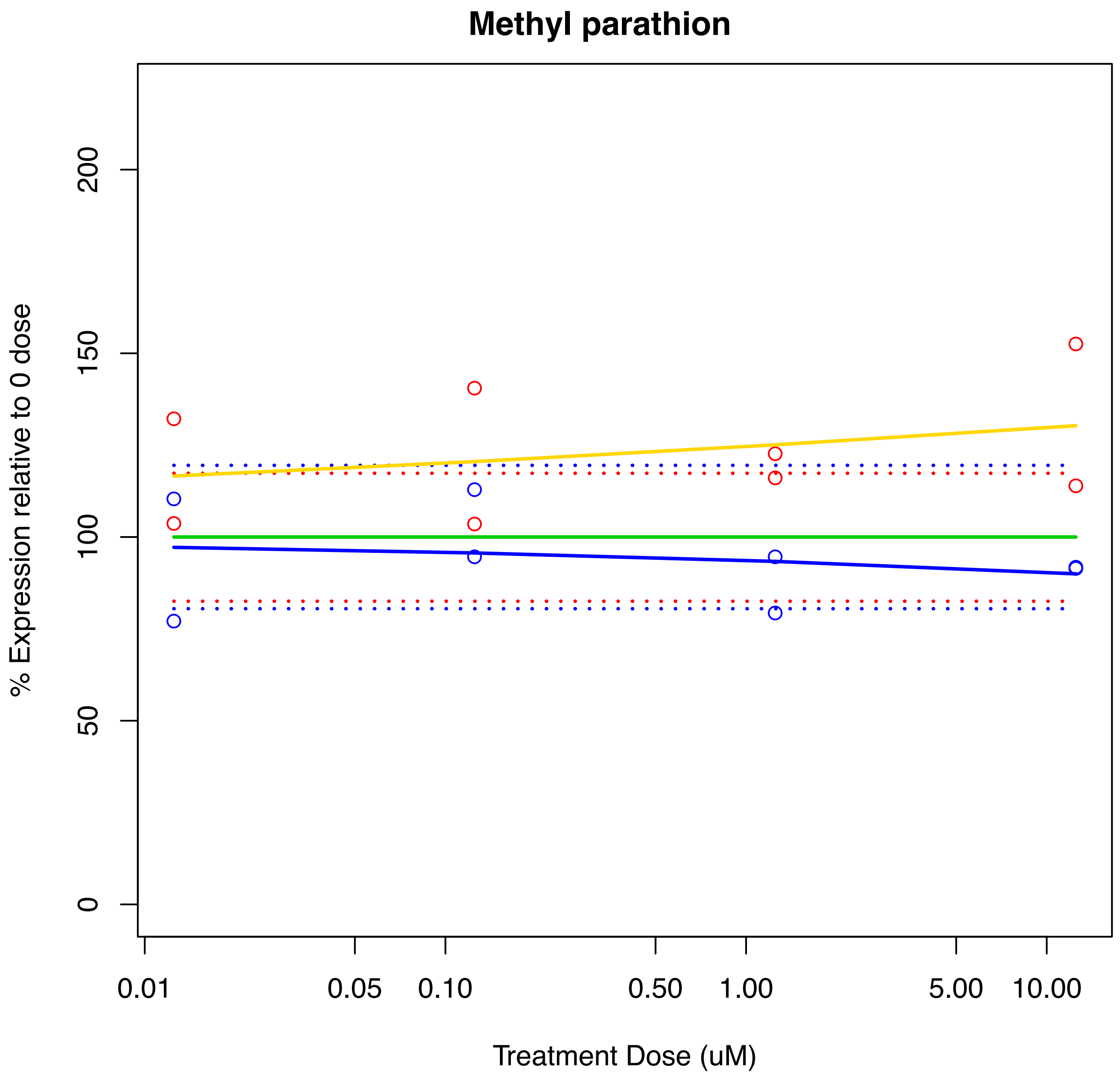

Methyl parathion
% Expression relative to 0 dose
0
50
100
150
200
0.01
0.05
0.10
0.50
1.00
5.00
10.00
Treatment Dose (uM)

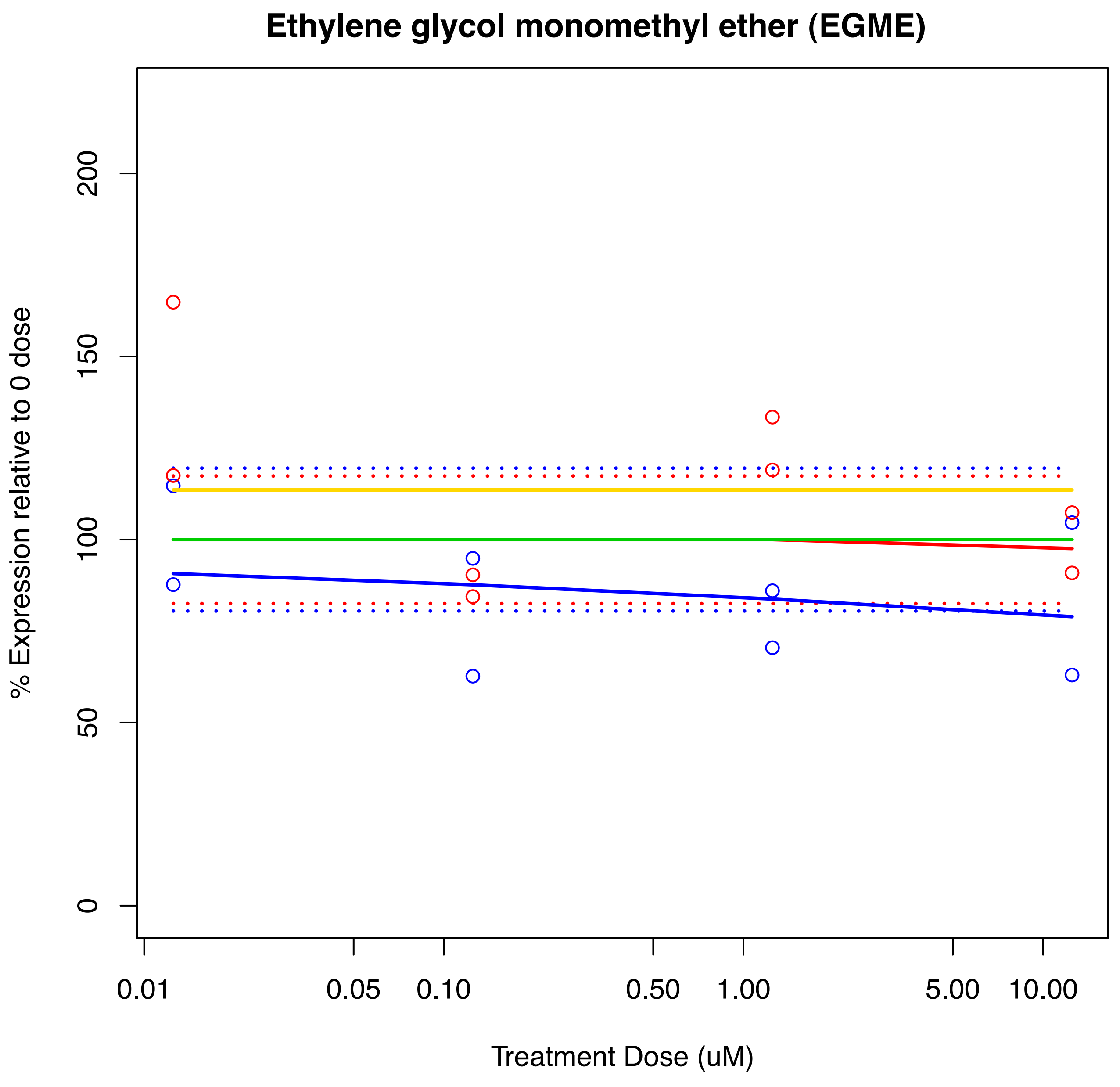

Ethylene glycol monomethyl ether (EGME)
% Expression relative to 0 dose
0
50
100
150
200
0.01
0.05
0.10
0.50
1.00
5.00
10.00
Treatment Dose (uM)

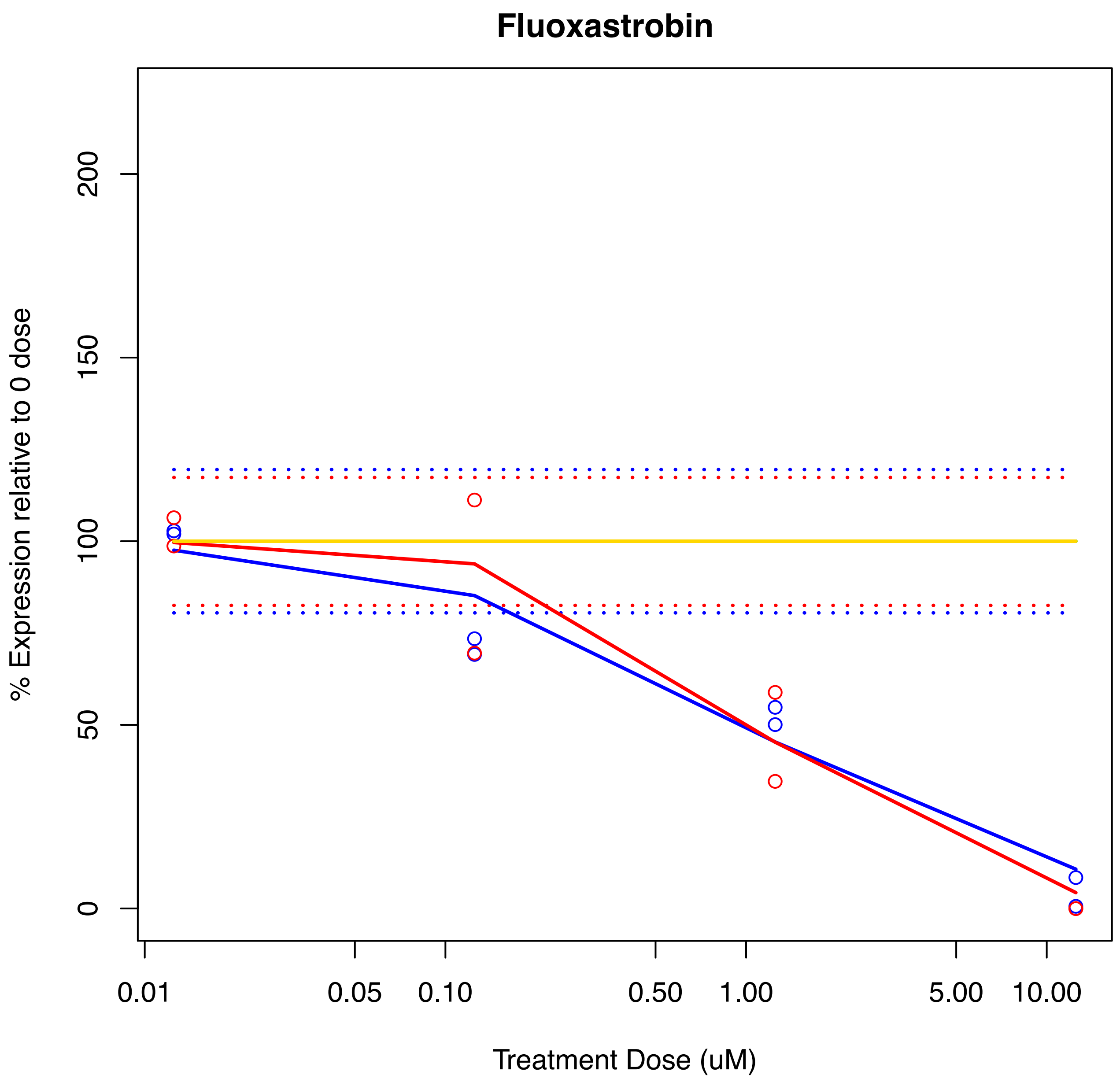
Fluoxastrobin
% Expression relative to 0 dose
0
50
100
150
200
0.01
0.05
0.10
0.50
1.00
5.00
10.00
Treatment Dose (uM)

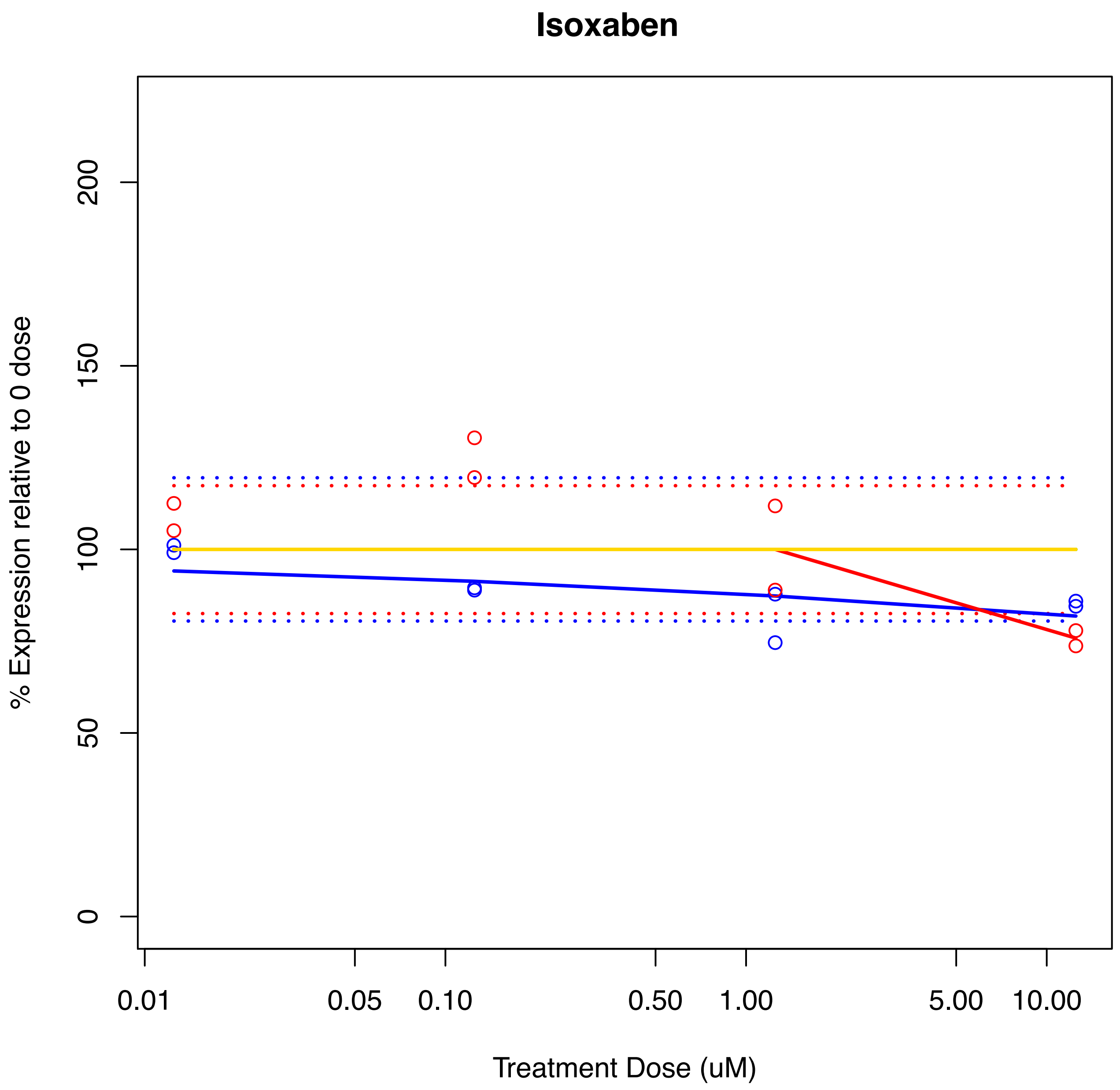

Isoxaben
% Expression relative to 0 dose
0
50
100
150
200
0.01
0.05
0.10
0.50
1.00
5.00
10.00
Treatment Dose (uM)

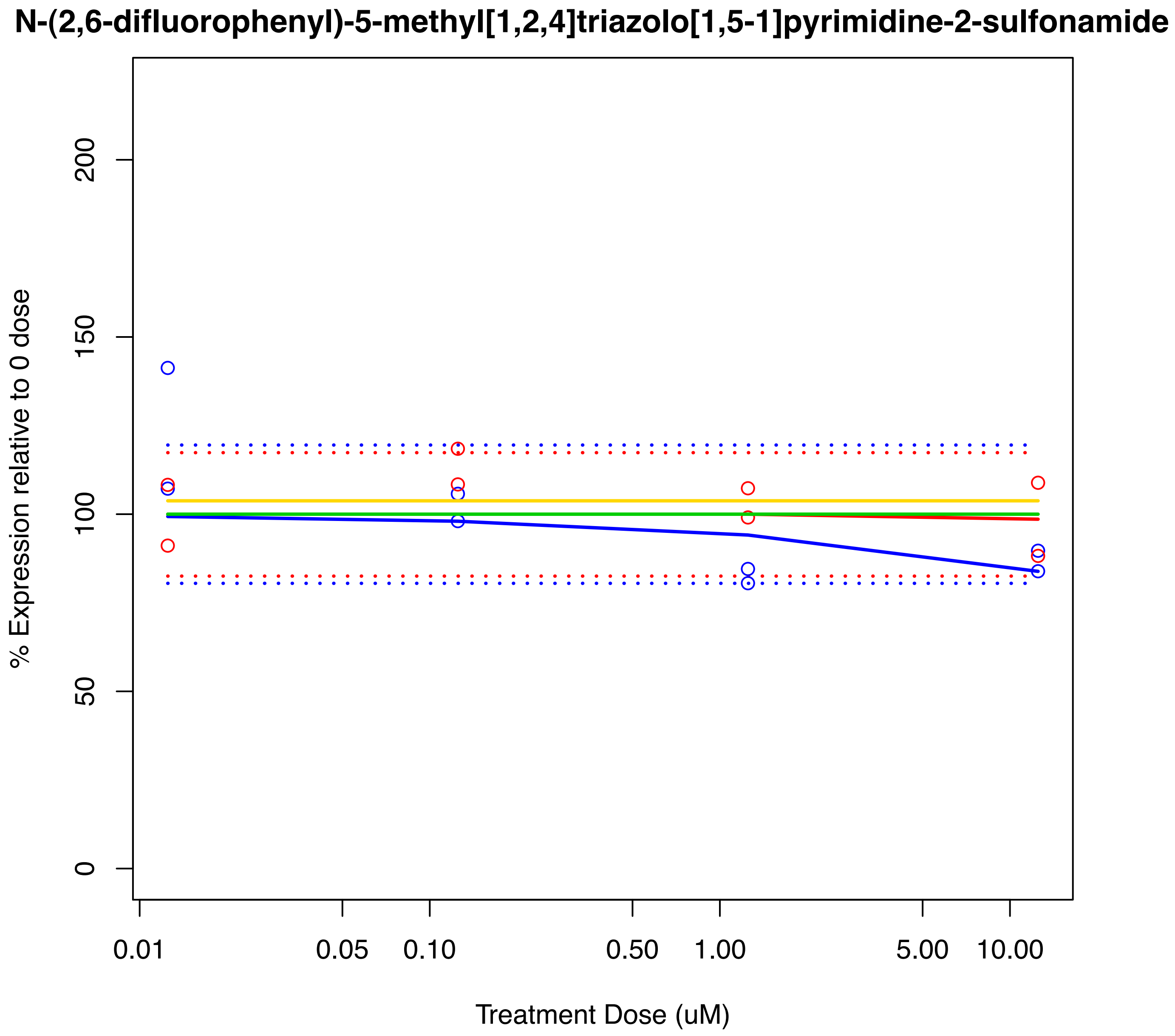
N-(2,6-difluorophenyl)-5-methyl[1,2,4]triazolo[1,5-1]pyrimidine-2-sulfonamide
% Expression relative to 0 dose
0
50
100
150
200
0.01
0.05
0.10
0.50
1.00
5.00
10.00
Treatment Dose (uM)

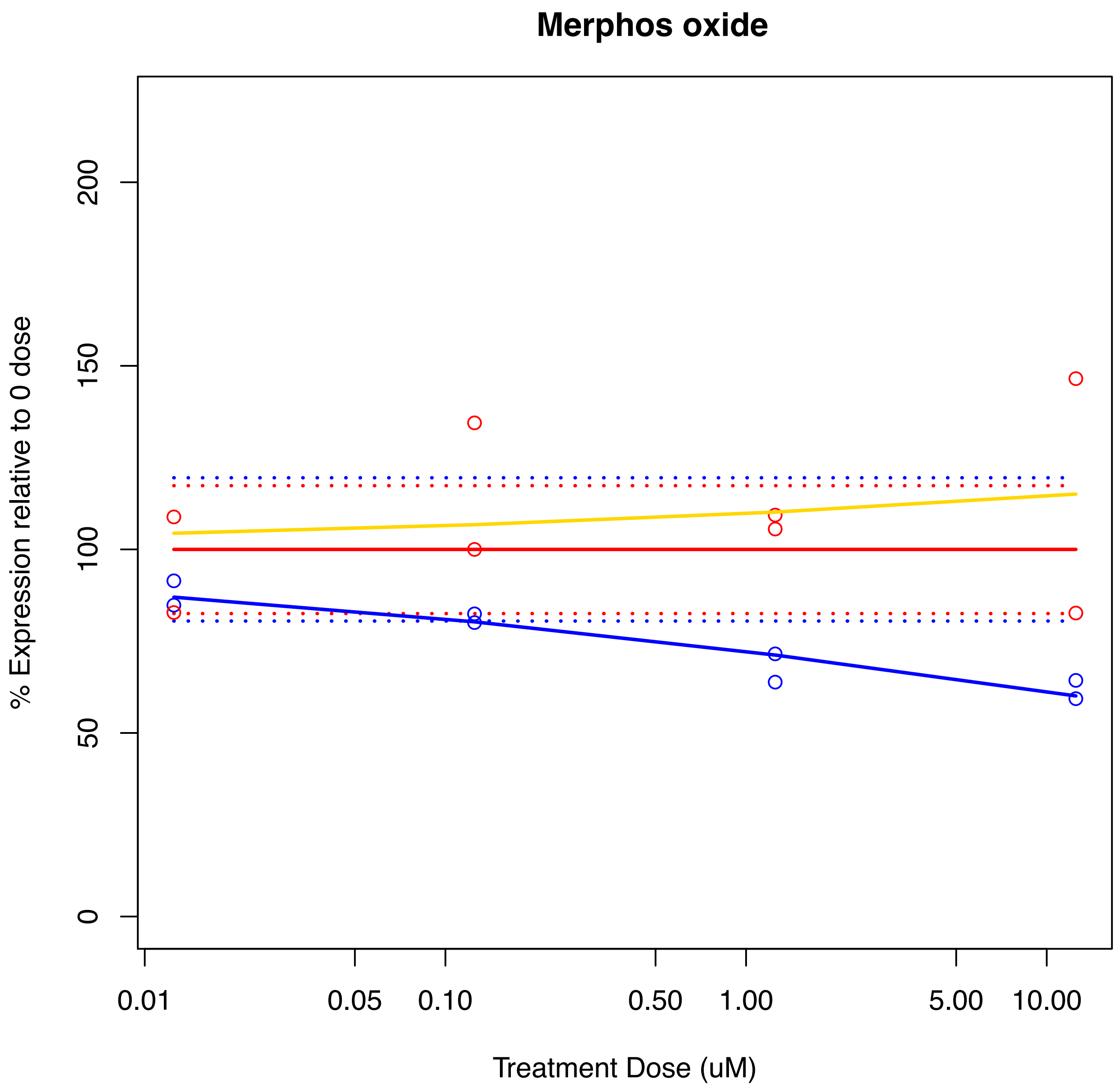
Merphos oxide
% Expression relative to 0 dose
0
50
100
150
200
0.01
0.05
0.10
0.50
1.00
5.00
10.00
Treatment Dose (uM)

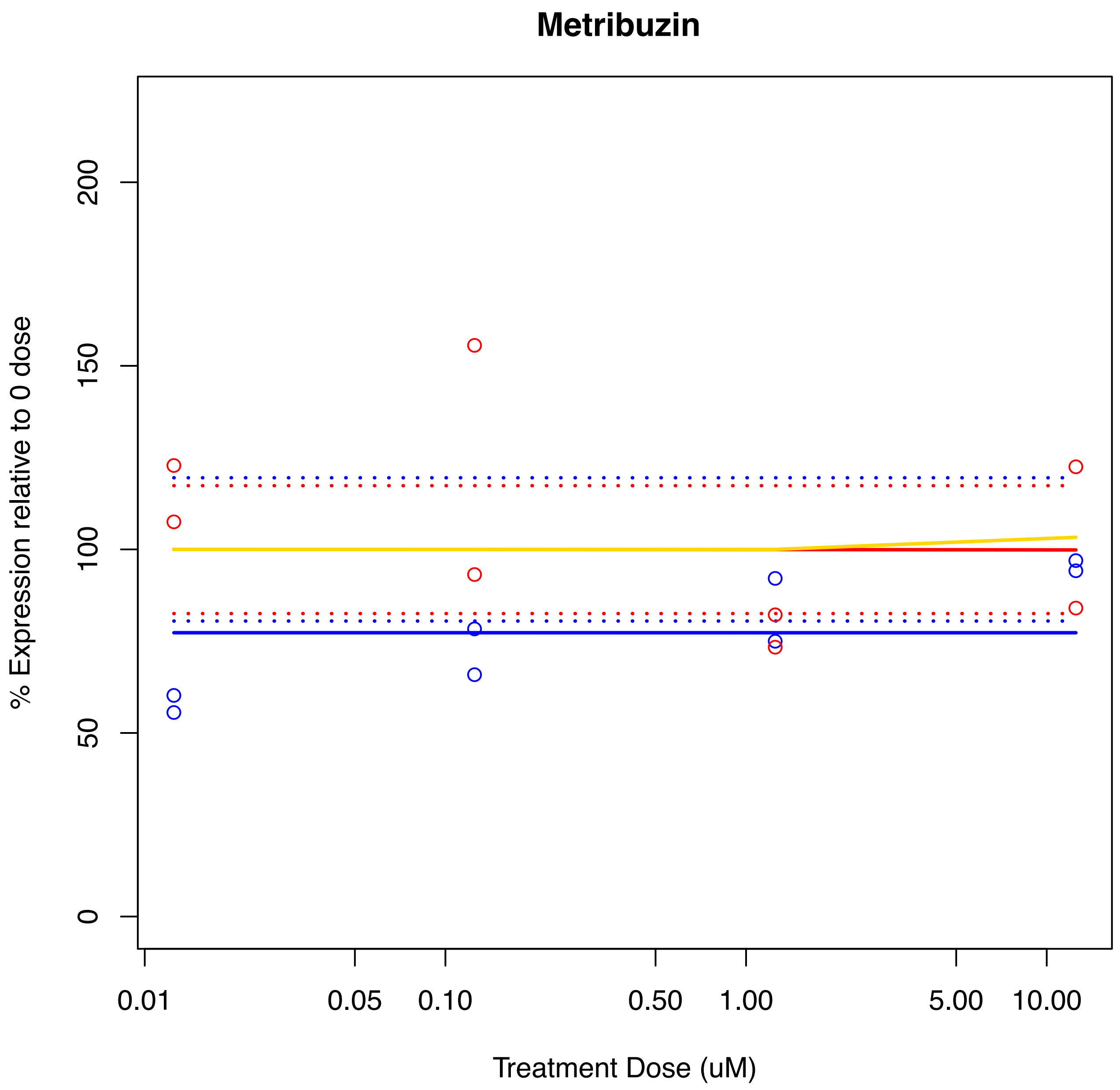

Metribuzin
% Expression relative to 0 dose
0
50
100
150
200
0.01
0.05
0.10
0.50
1.00
5.00
10.00
Treatment Dose (uM)

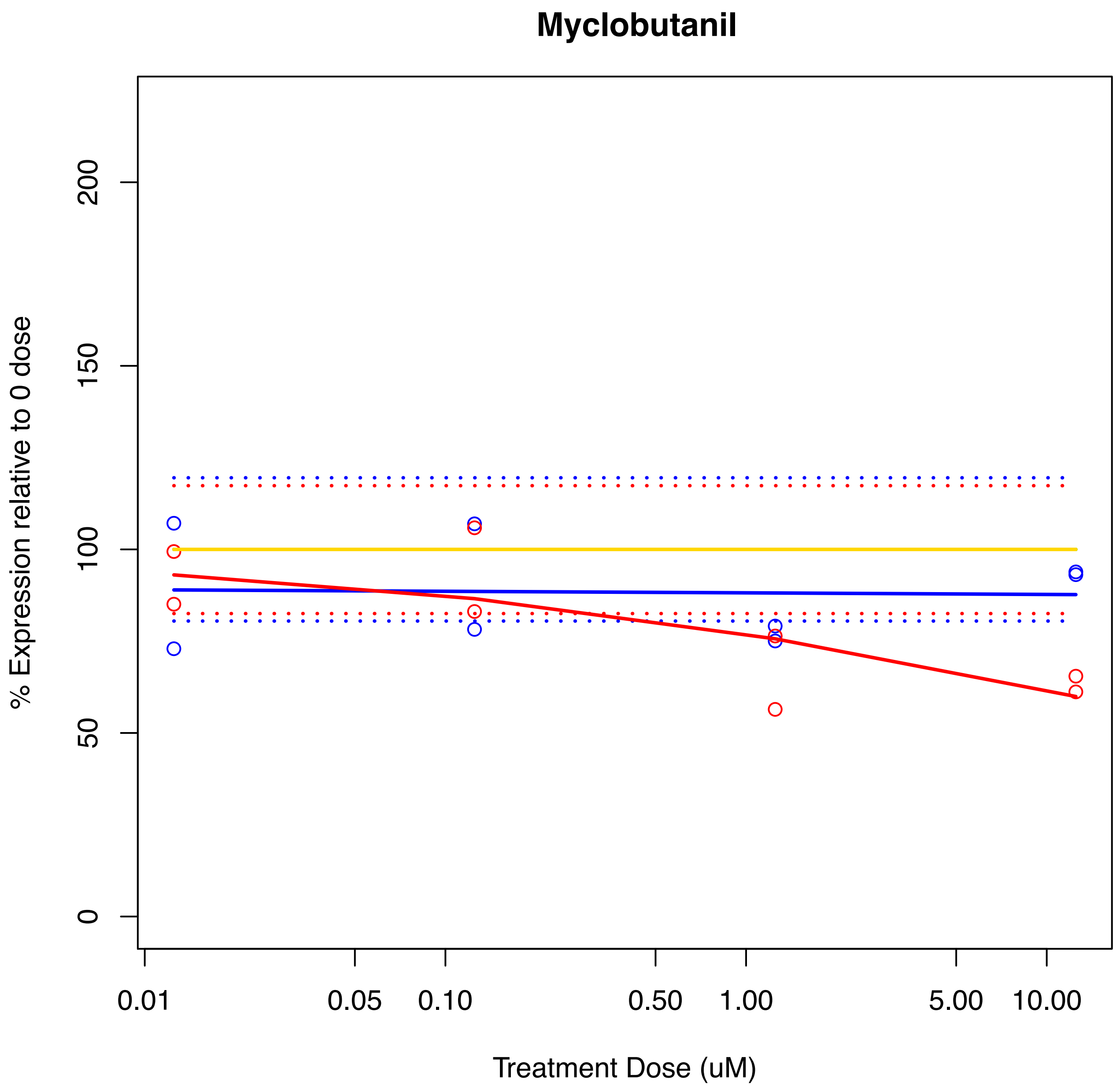

Myclobutanil
% Expression relative to 0 dose
200
150
100
50
0
0.01
0.05
0.10
0.50
1.00
5.00
10.00
Treatment Dose (uM)

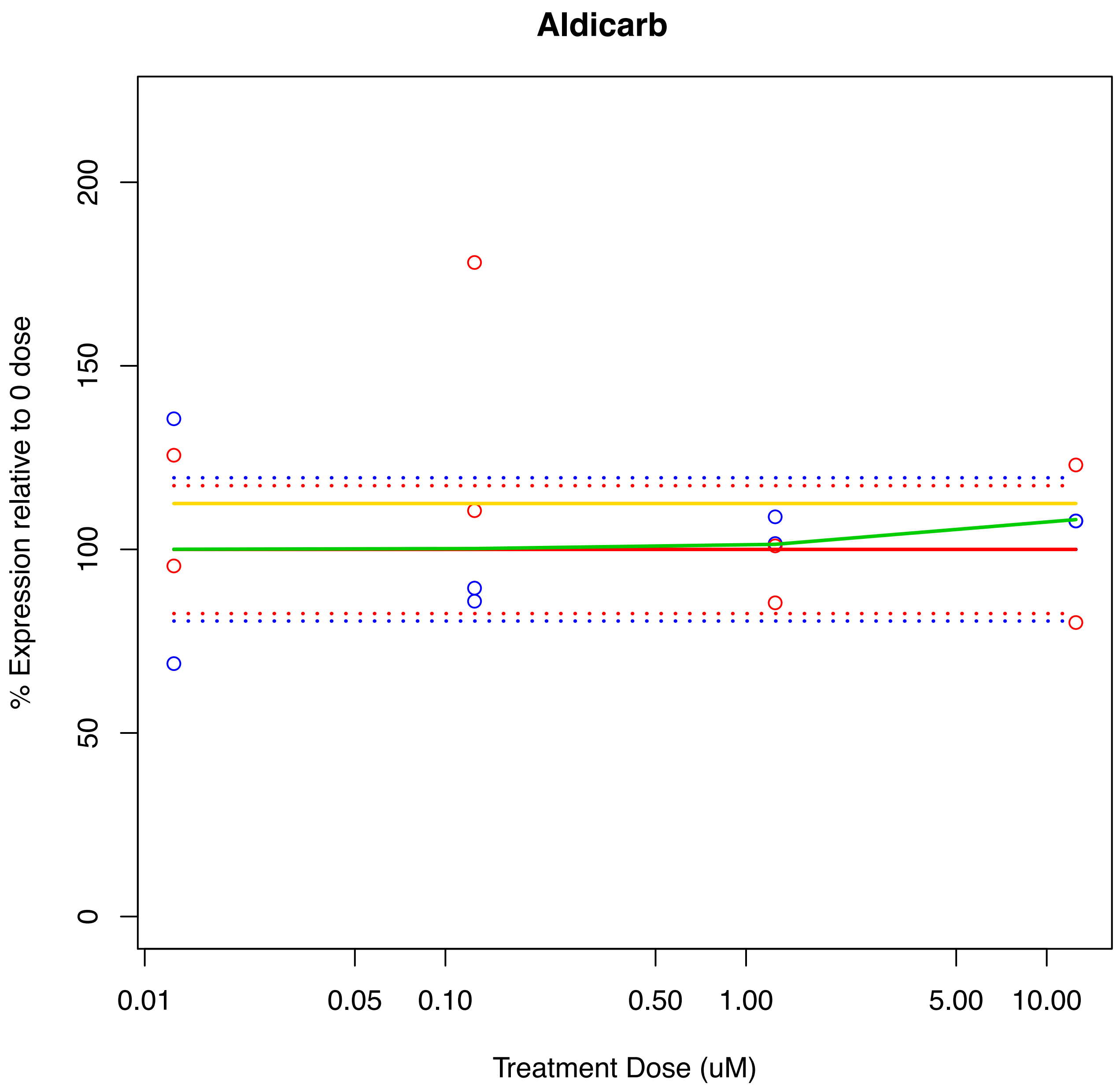
Aldicarb
% Expression relative to 0 dose
0
50
100
150
200
0.01
0.05
0.10
0.50
1.00
5.00
10.00
Treatment Dose (uM)

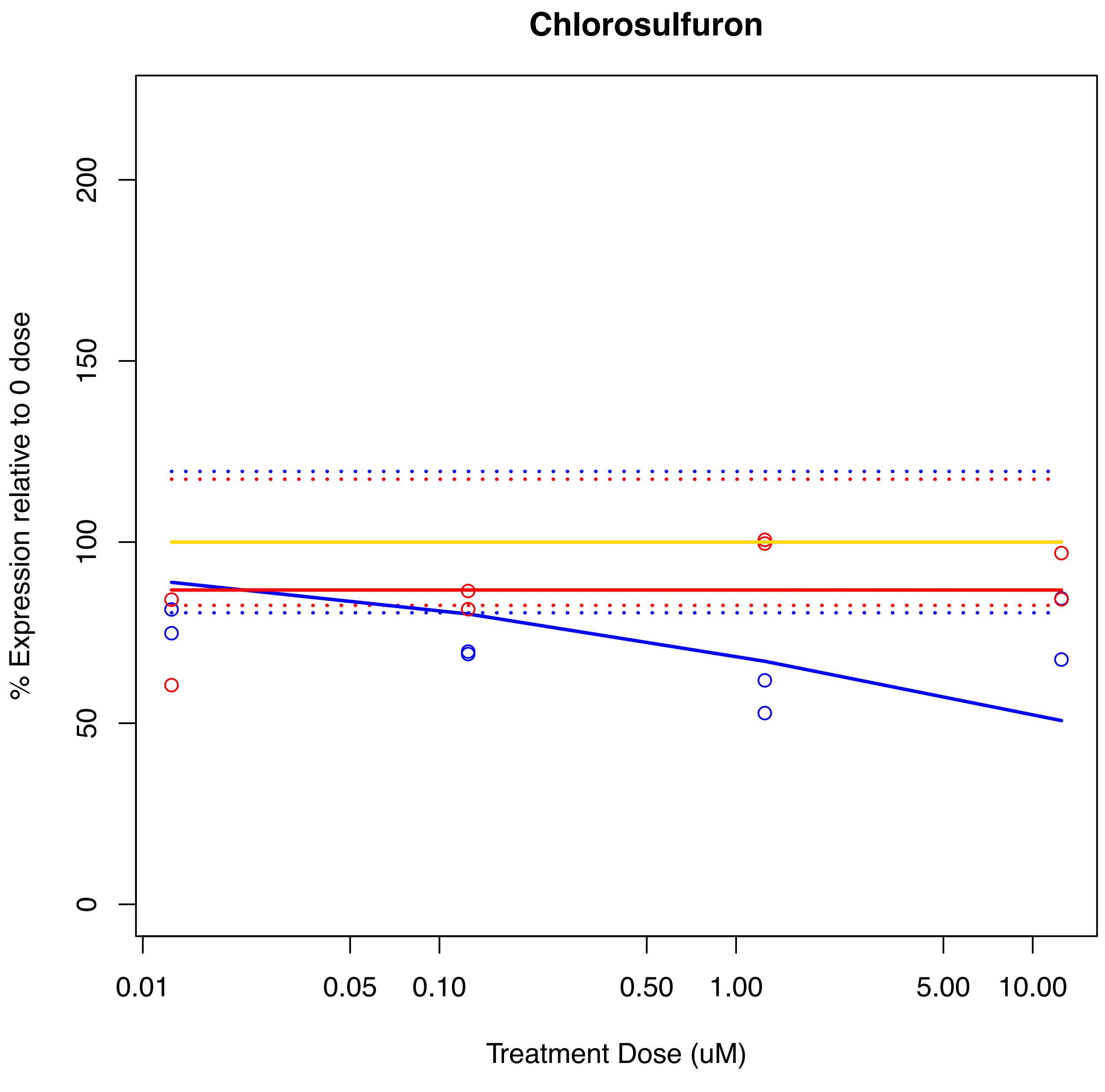
Chlorosulfuron
% Expression relative to 0 dose
0
50
100
150
200
0.01
0.05
0.10
0.50
1.00
5.00
10.00
Treatment Dose (uM)

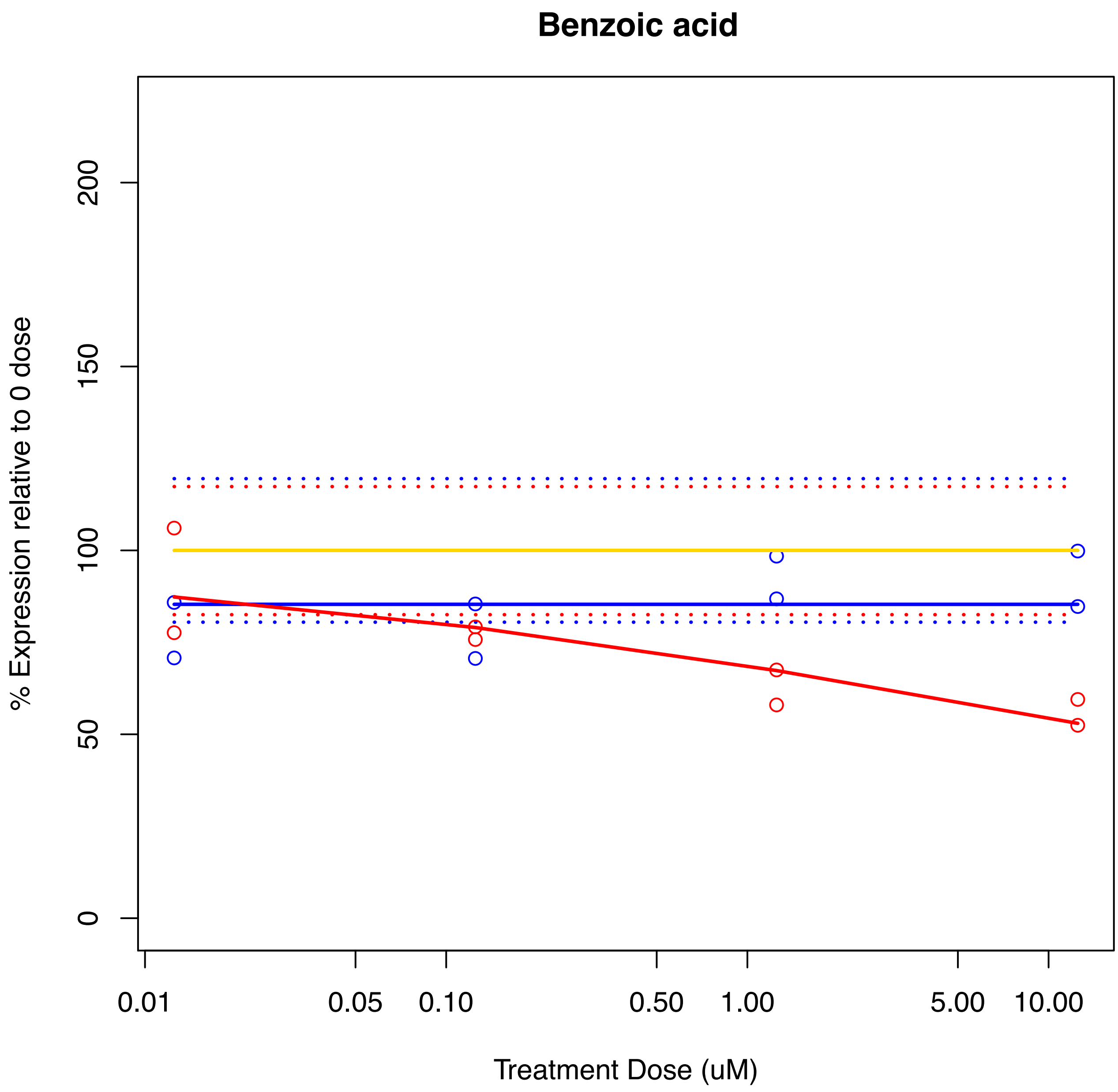

Benzoic acid
% Expression relative to 0 dose
0
50
100
150
200
0.01
0.05
0.10
0.50
1.00
5.00
10.00
Treatment Dose (uM)

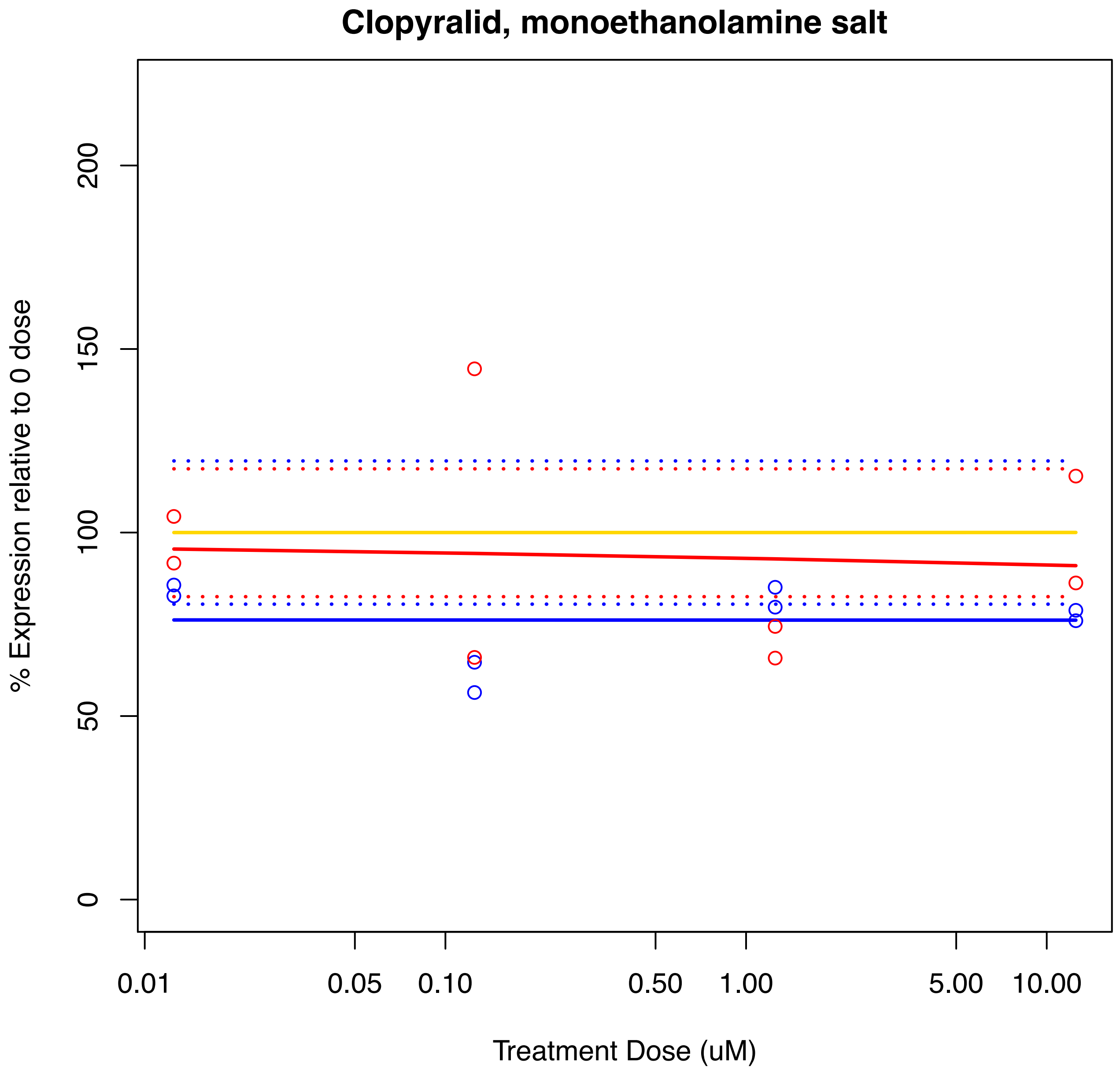
Clopyralid, monoethanolamine salt
% Expression relative to 0 dose
0
50
100
150
200
0.01
0.05
0.10
0.50
1.00
5.00
10.00
Treatment Dose (uM)

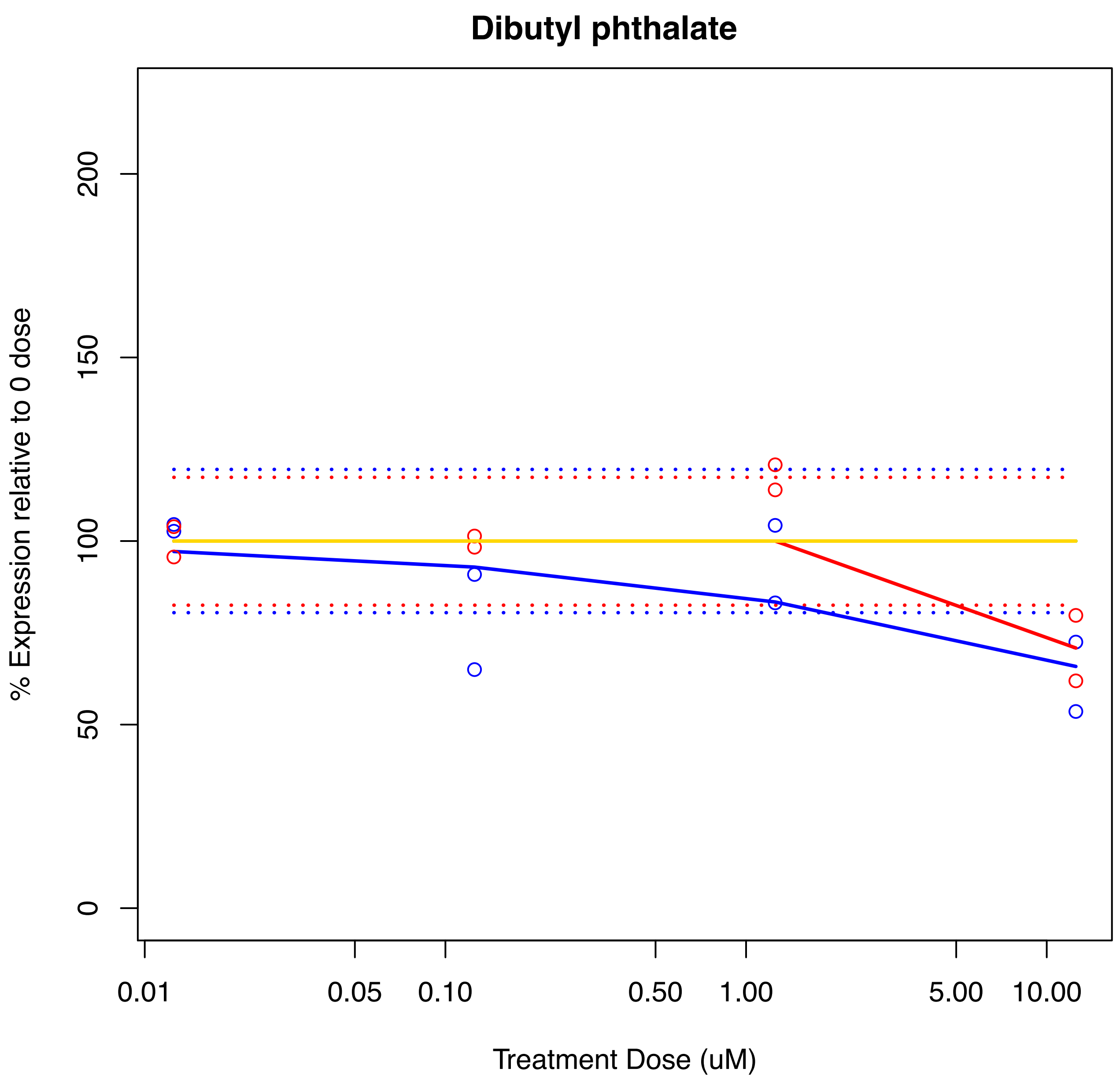

Dibutyl phthalate
% Expression relative to 0 dose
0
50
100
150
200
0.01
0.05
0.10
0.50
1.00
5.00
10.00
Treatment Dose (uM)

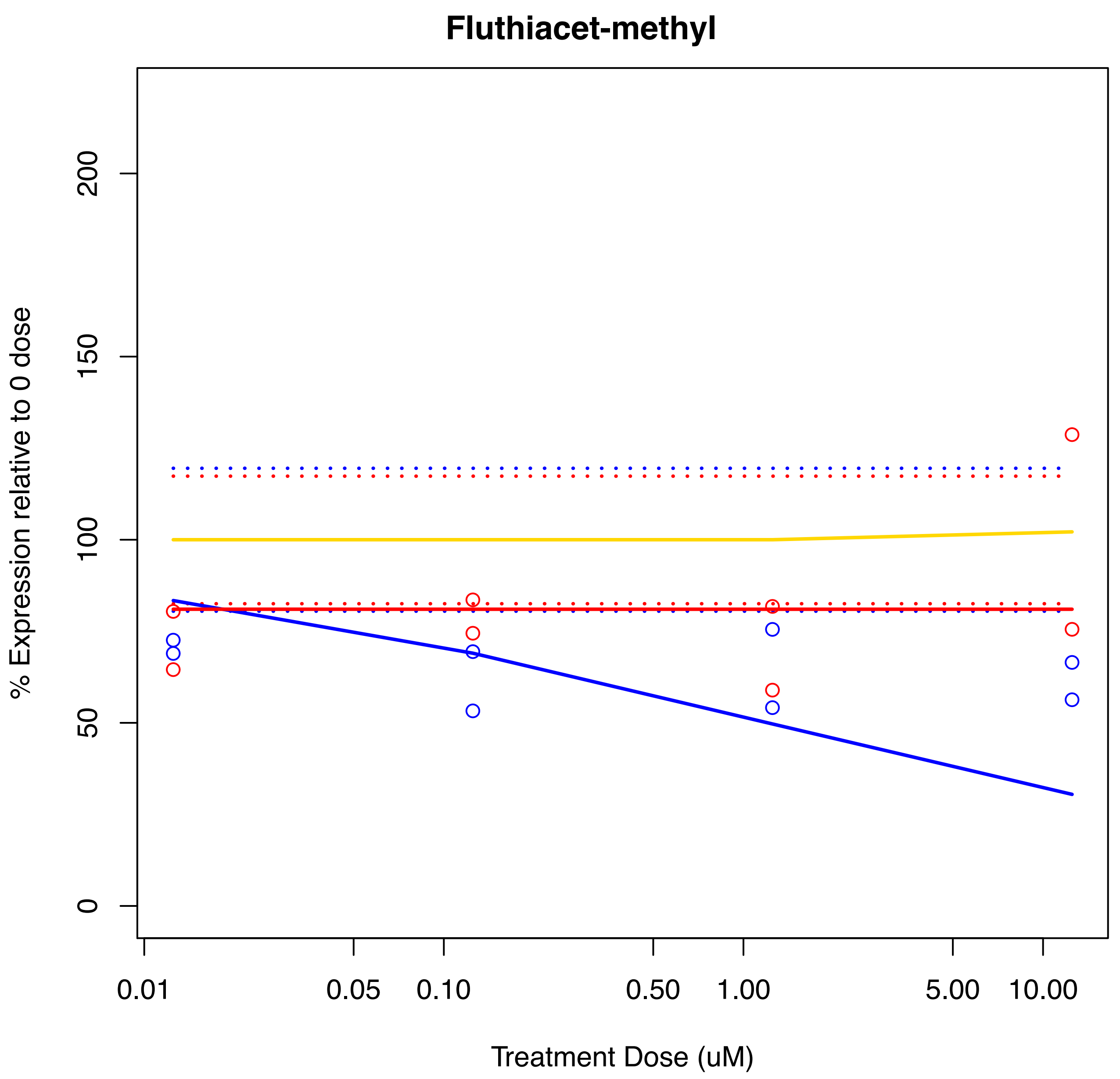

Fluthiacet-methyl
% Expression relative to 0 dose
0
50
100
150
200
0.01
0.05
0.10
0.50
1.00
5.00
10.00
Treatment Dose (uM)

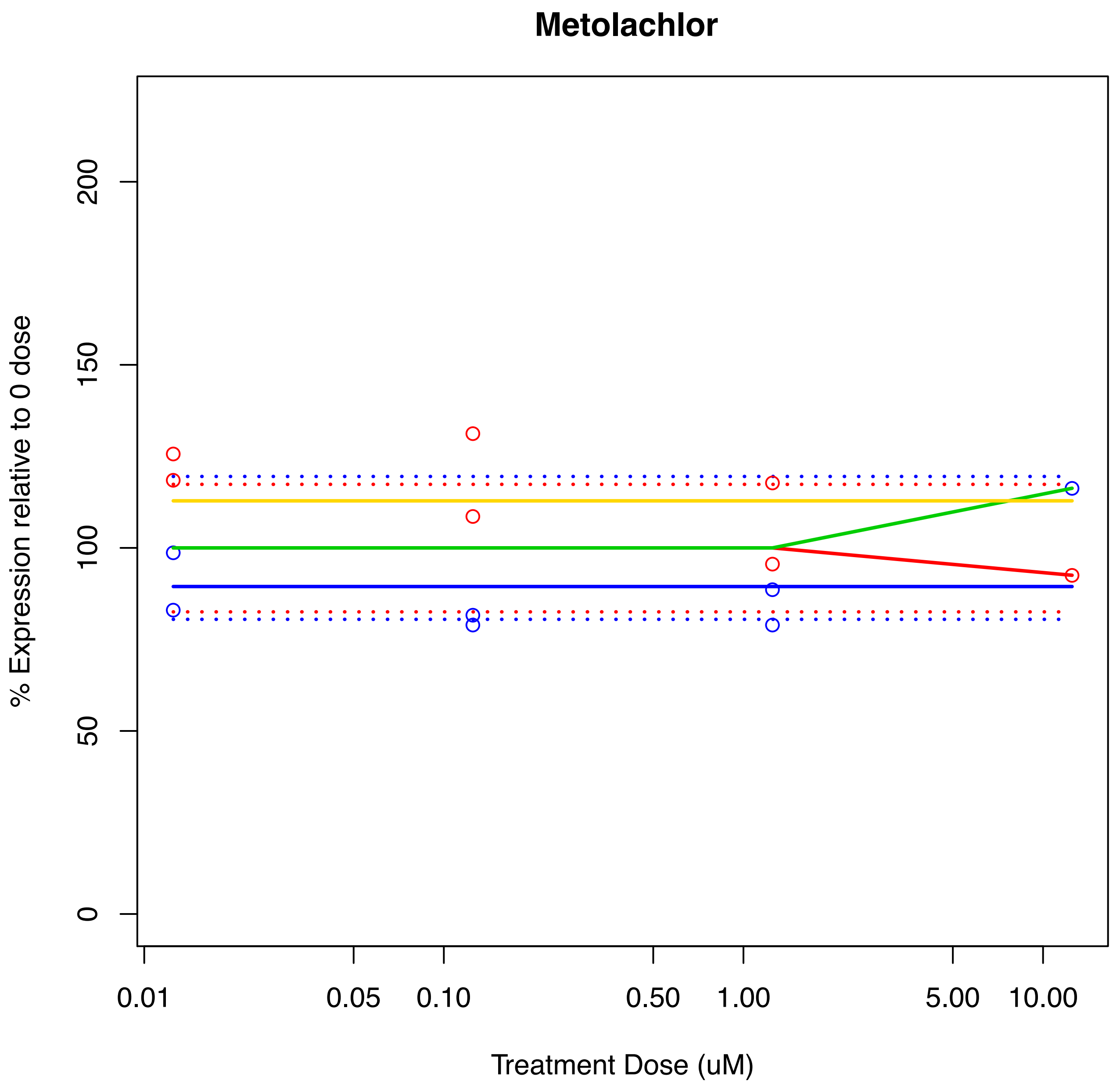

Metolachlor
% Expression relative to 0 dose
0
50
100
150
200
0.01
0.05
0.10
0.50
1.00
5.00
10.00
Treatment Dose (uM)

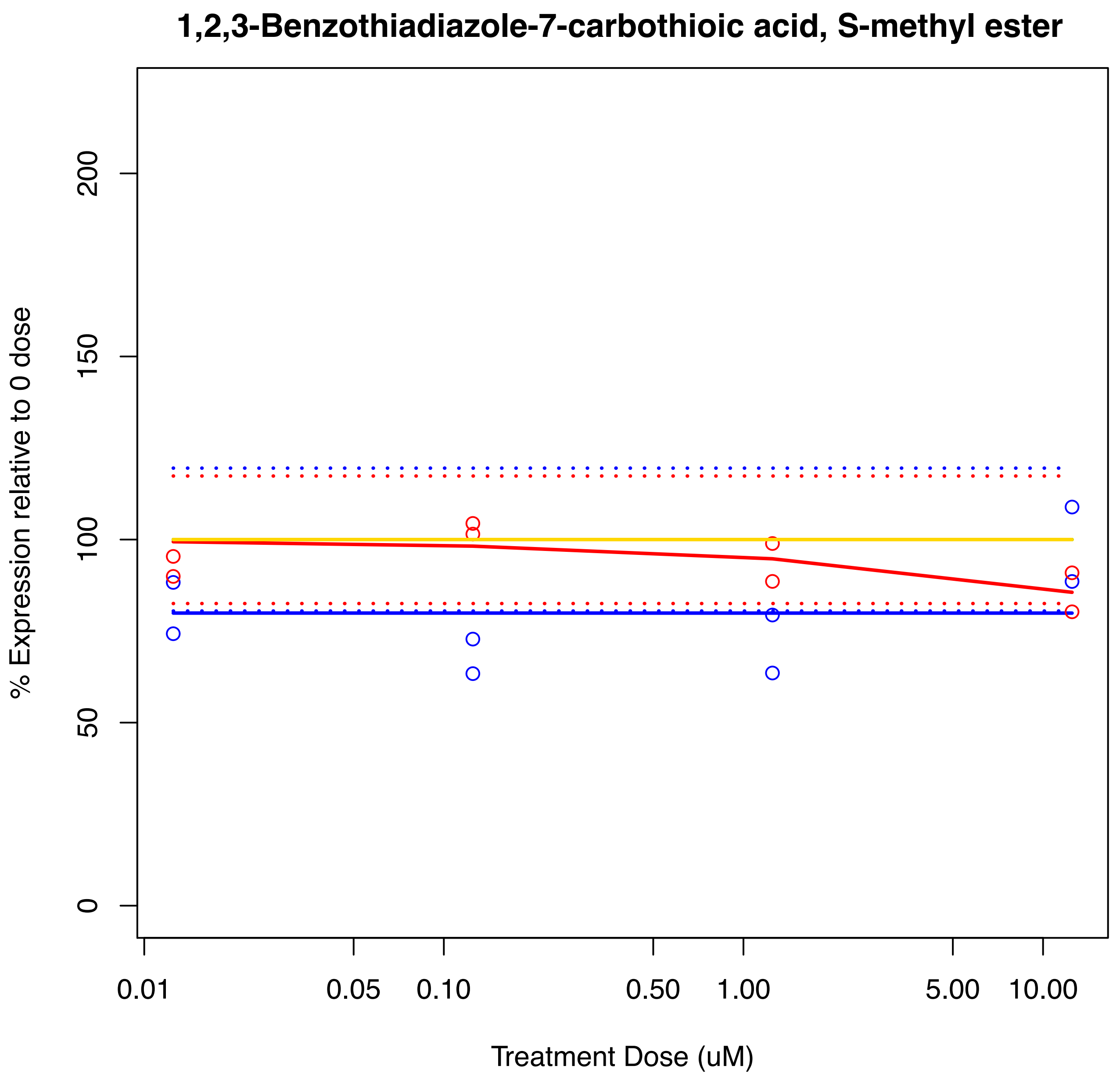
1,2,3-Benzothiadiazole-7-carbothioic acid, S-methyl ester
% Expression relative to 0 dose
0
50
100
150
200
0.01
0.05
0.10
0.50
1.00
5.00
10.00
Treatment Dose (uM)

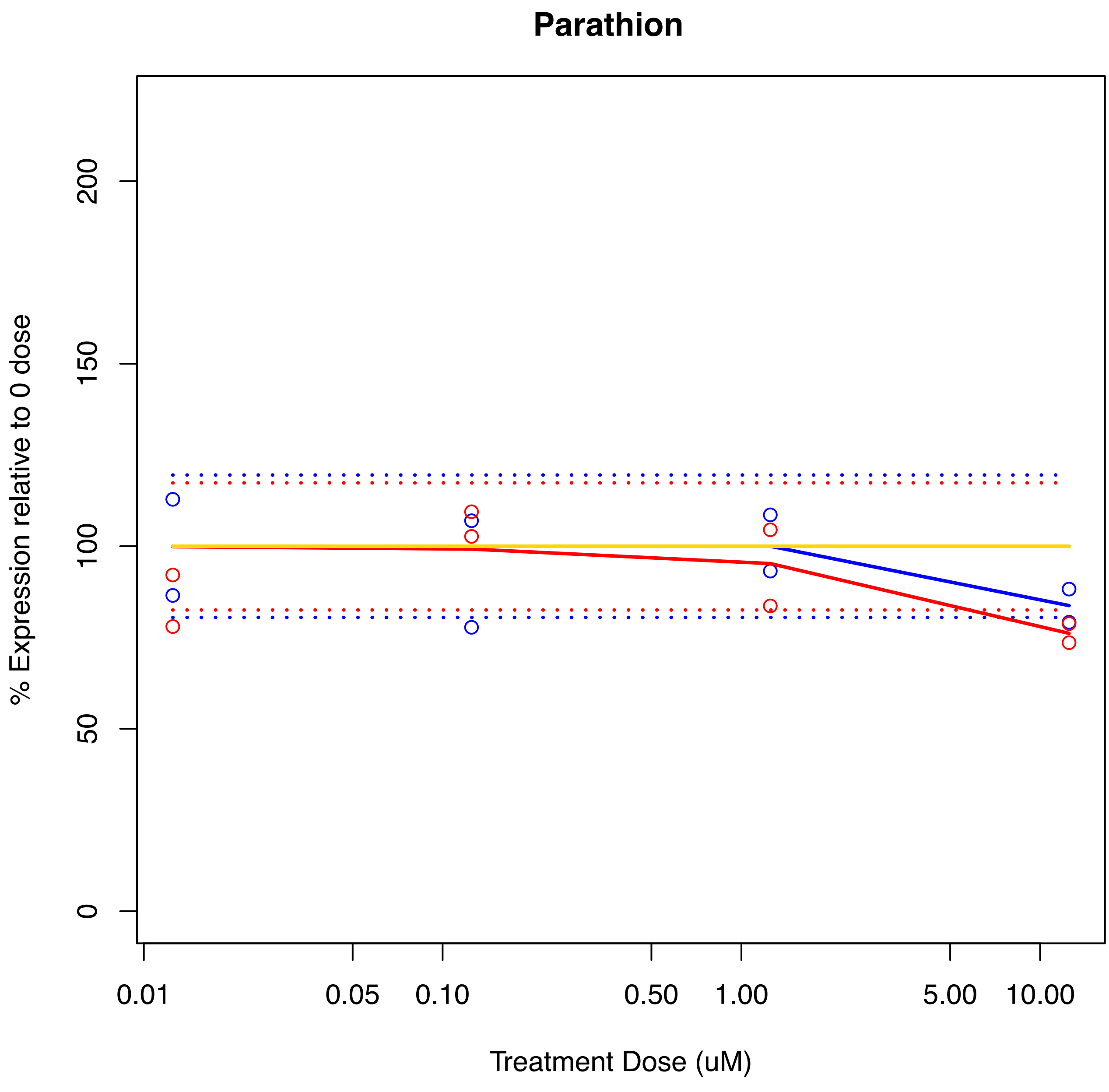

Parathion
% Expression relative to 0 dose
0
50
100
150
200
0.01
0.05
0.10
0.50
1.00
5.00
10.00
Treatment Dose (uM)

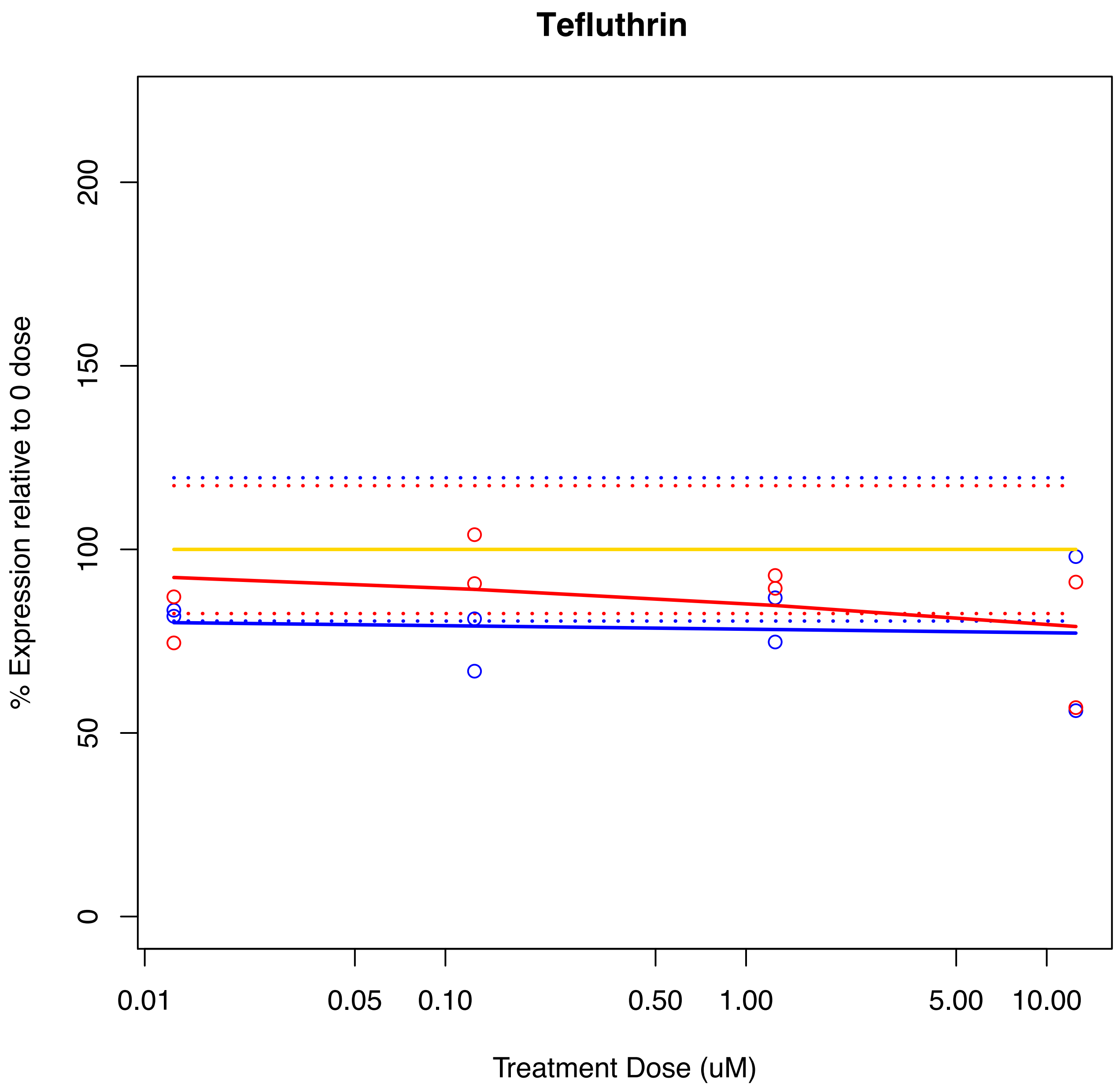
Tefluthrin
% Expression relative to 0 dose
0
50
100
150
200
0.01
0.05
0.10
0.50
1.00
5.00
10.00
Treatment Dose (uM)

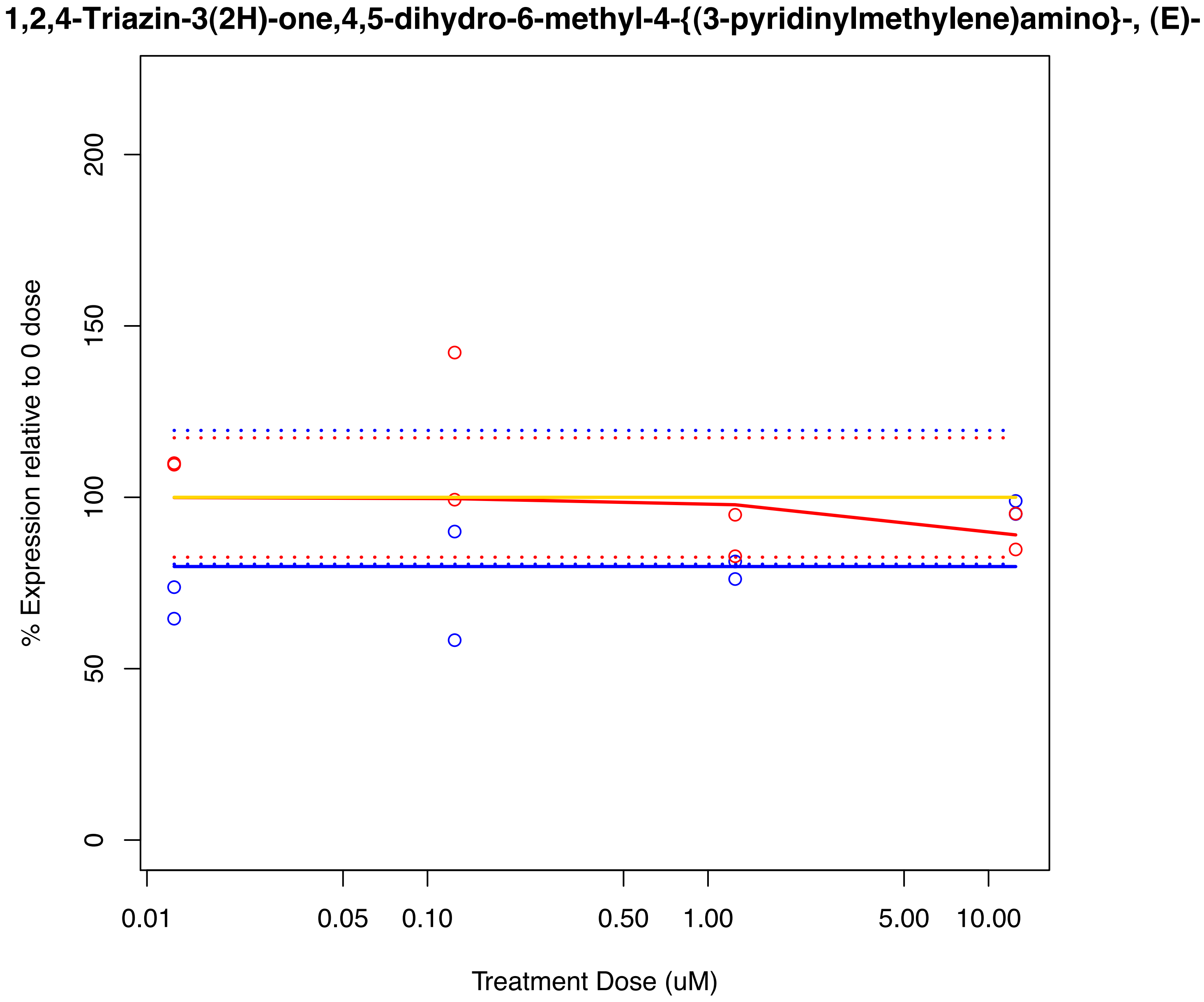

1,2,4-Triazin-3(2H)-one,4,5-dihydro-6-methyl-4-{(3-pyridinylmethylene)amino}-, (E)-
% Expression relative to 0 dose
0
50
100
150
200
0.01
0.05
0.10
0.50
1.00
5.00
10.00
Treatment Dose (uM)

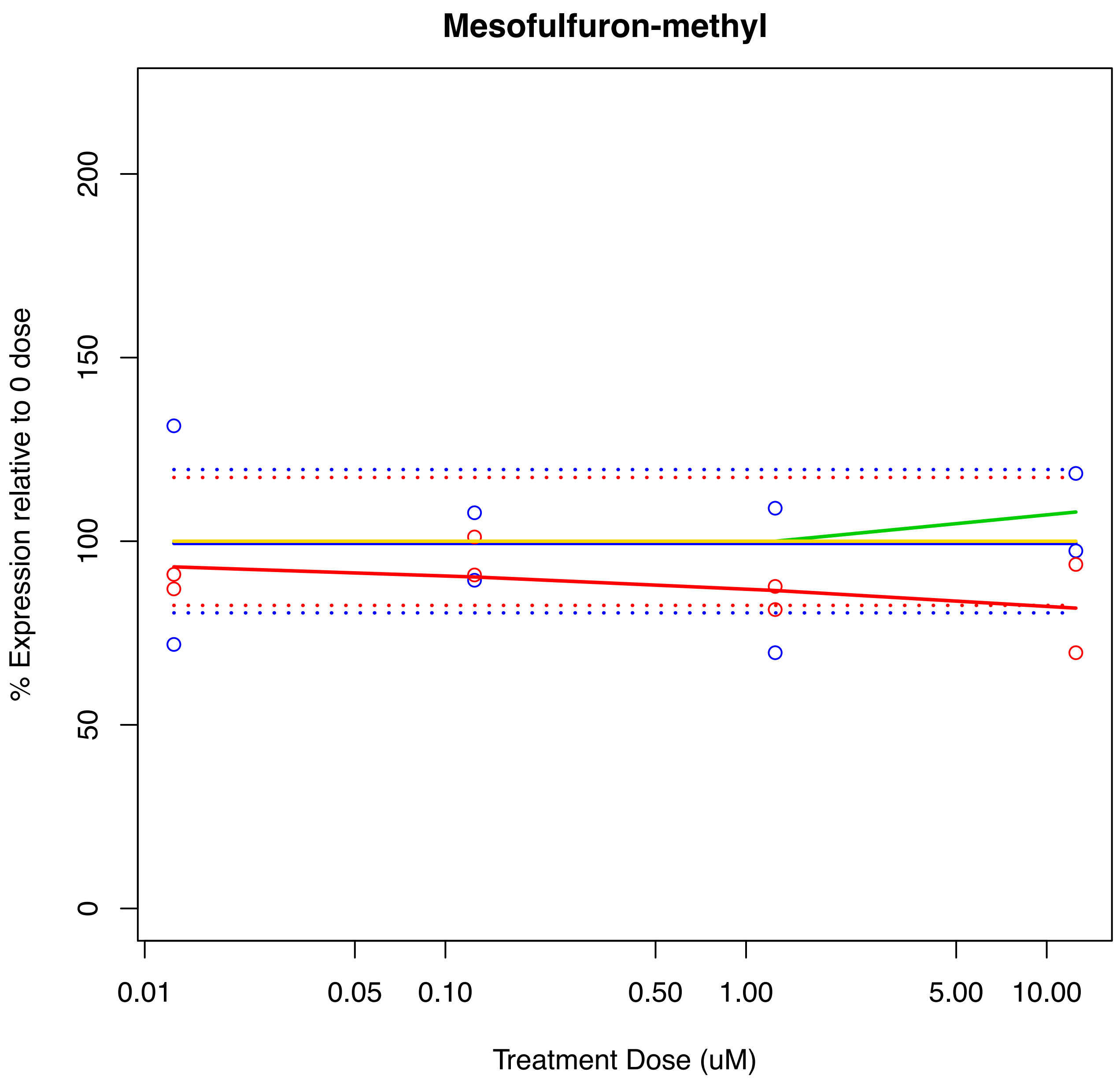

Mesofulfuron-methyl
% Expression relative to 0 dose
0
50
100
150
200
0.01
0.05
0.10
0.50
1.00
5.00
10.00
Treatment Dose (uM)

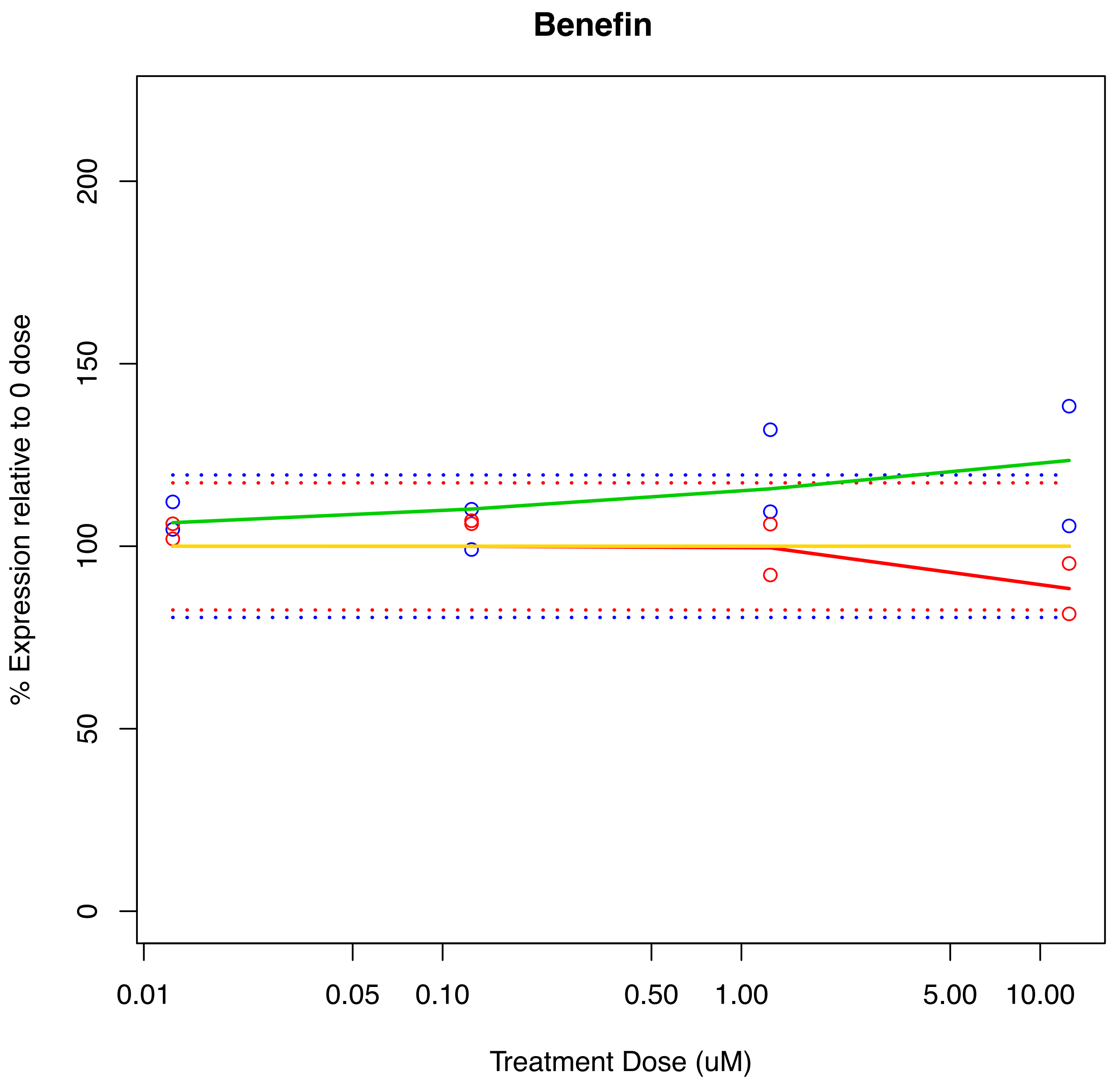
Benefin
% Expression relative to 0 dose
0
50
100
150
200
0.01
0.05
0.10
0.50
1.00
5.00
10.00
Treatment Dose (uM)

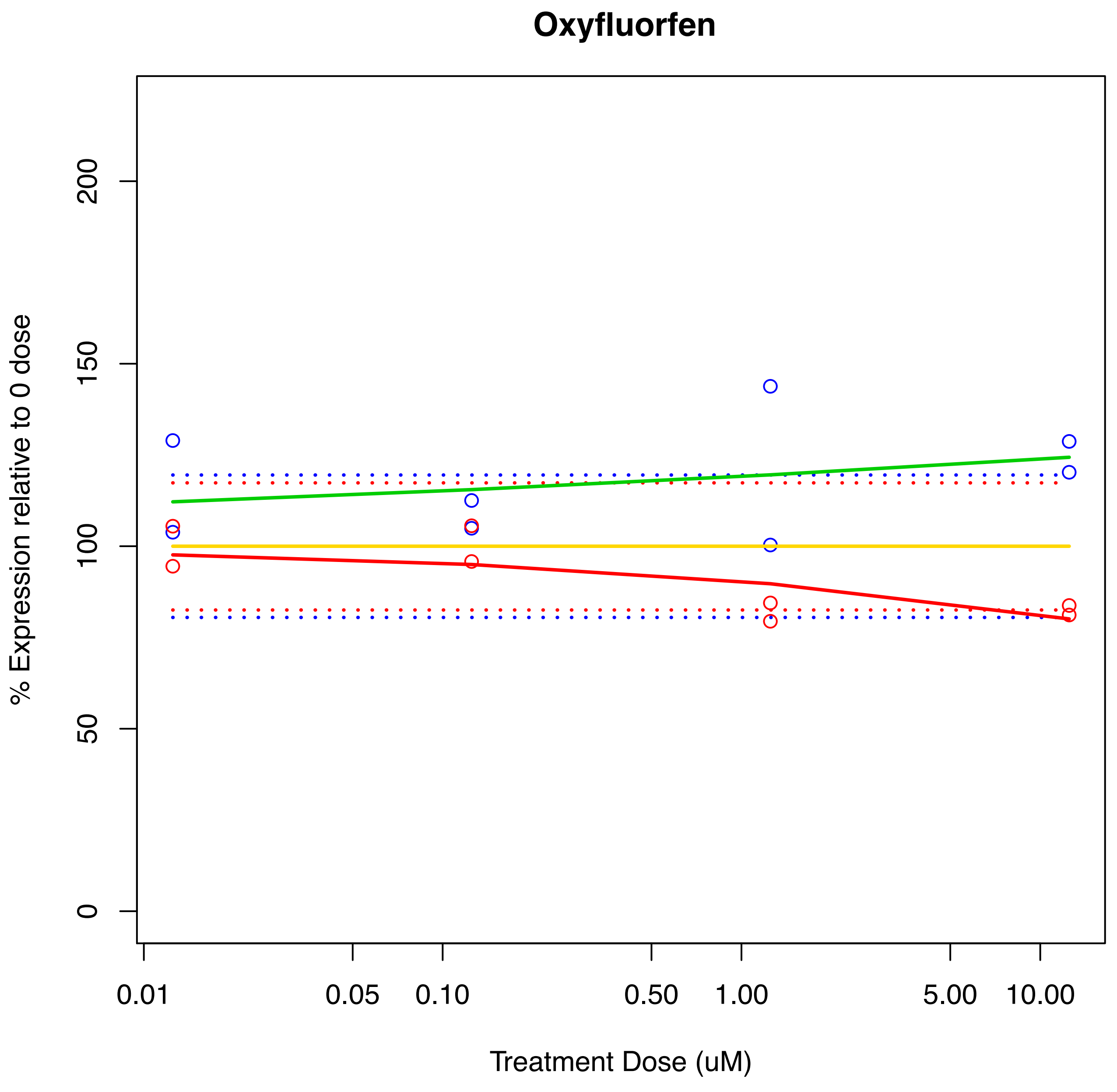

Oxyfluorfen
% Expression relative to 0 dose
0
50
100
150
200
0.01
0.05
0.10
0.50
1.00
5.00
10.00
Treatment Dose (uM)

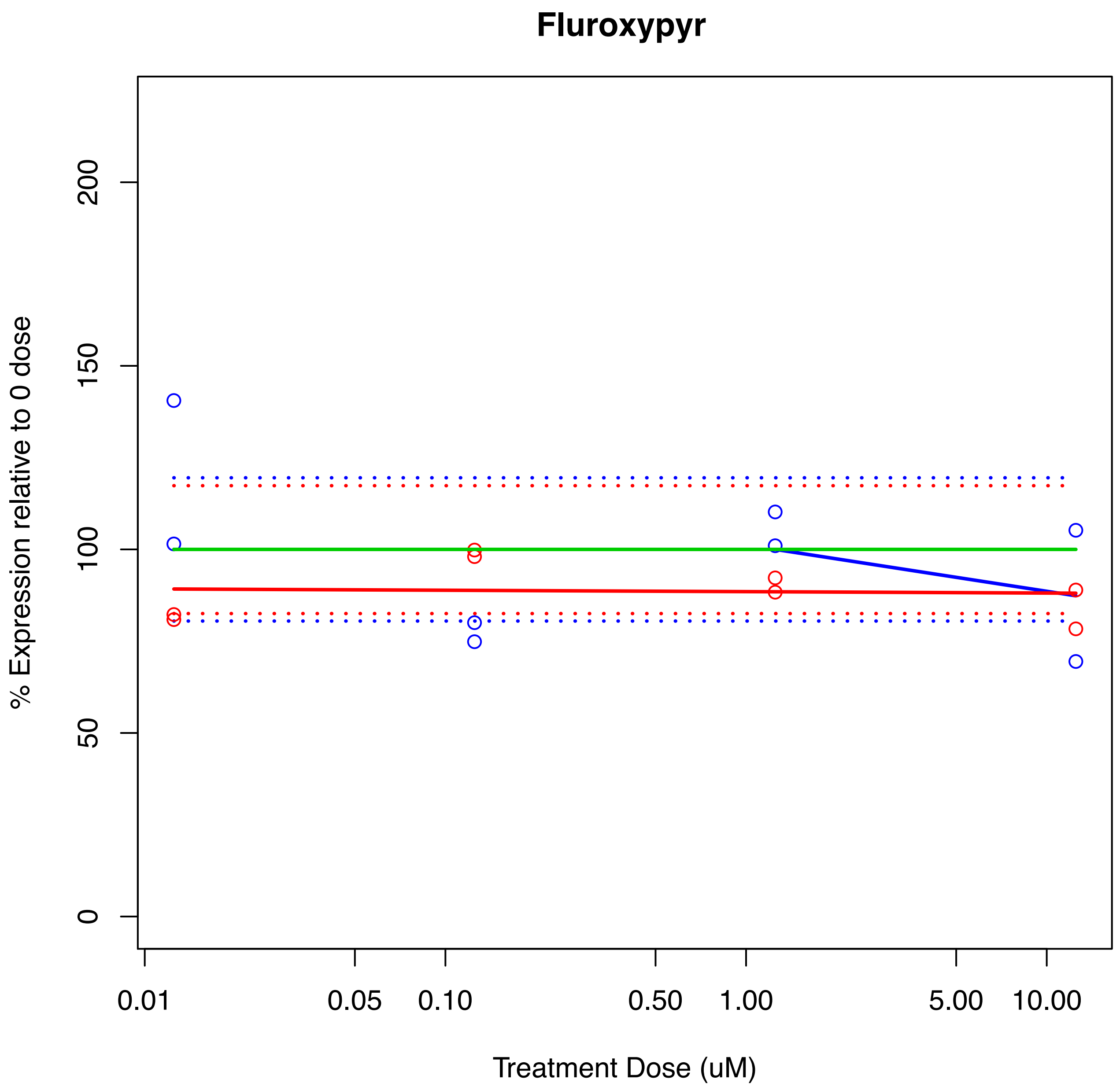

Fluroxypyr
% Expression relative to 0 dose
0
50
100
150
200
0.01
0.05
0.10
0.50
1.00
5.00
10.00
Treatment Dose (uM)

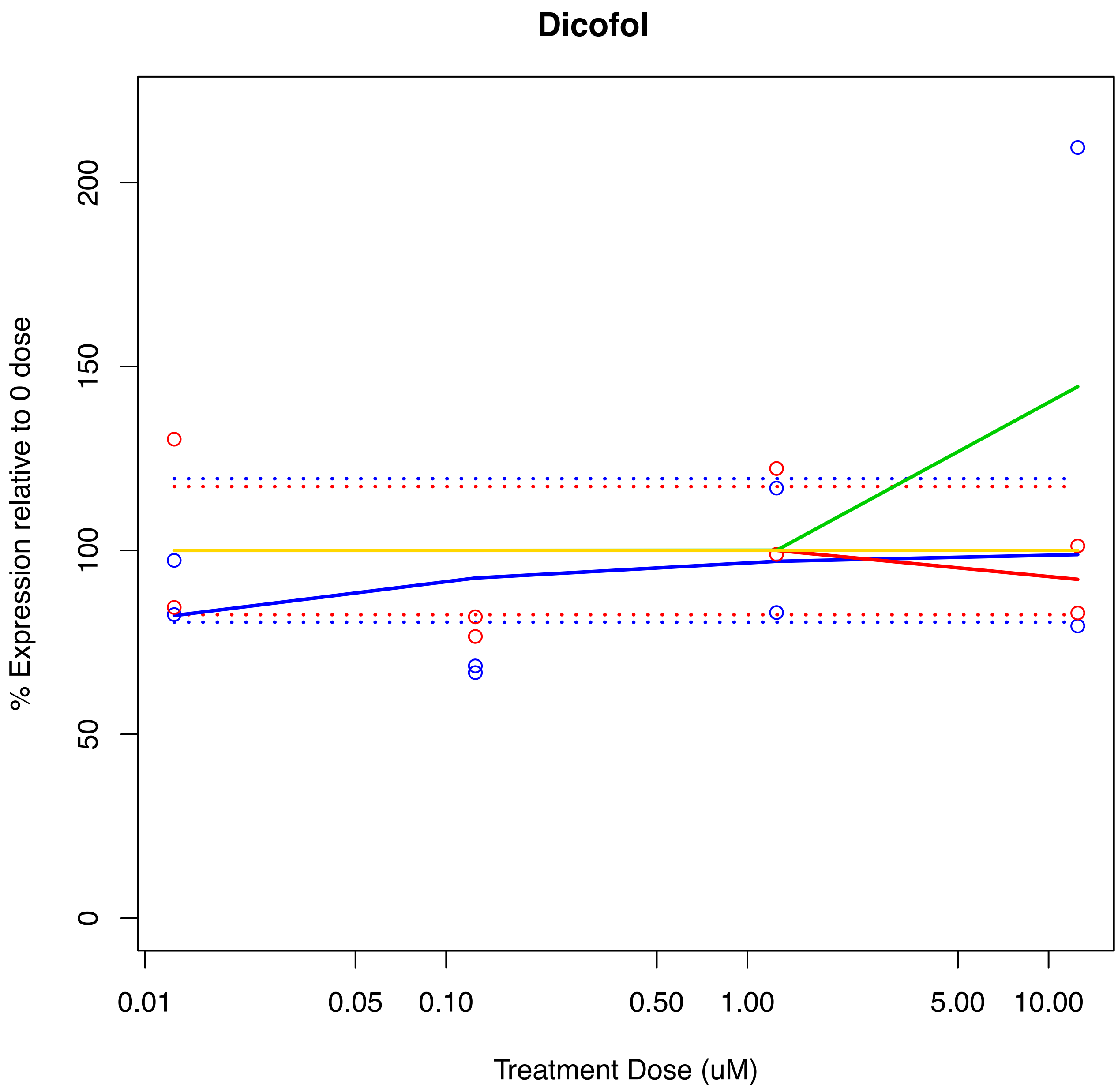
Dicofol
% Expression relative to 0 dose
0
50
100
150
200
0.01
0.05
0.10
0.50
1.00
5.00
10.00
Treatment Dose (uM)

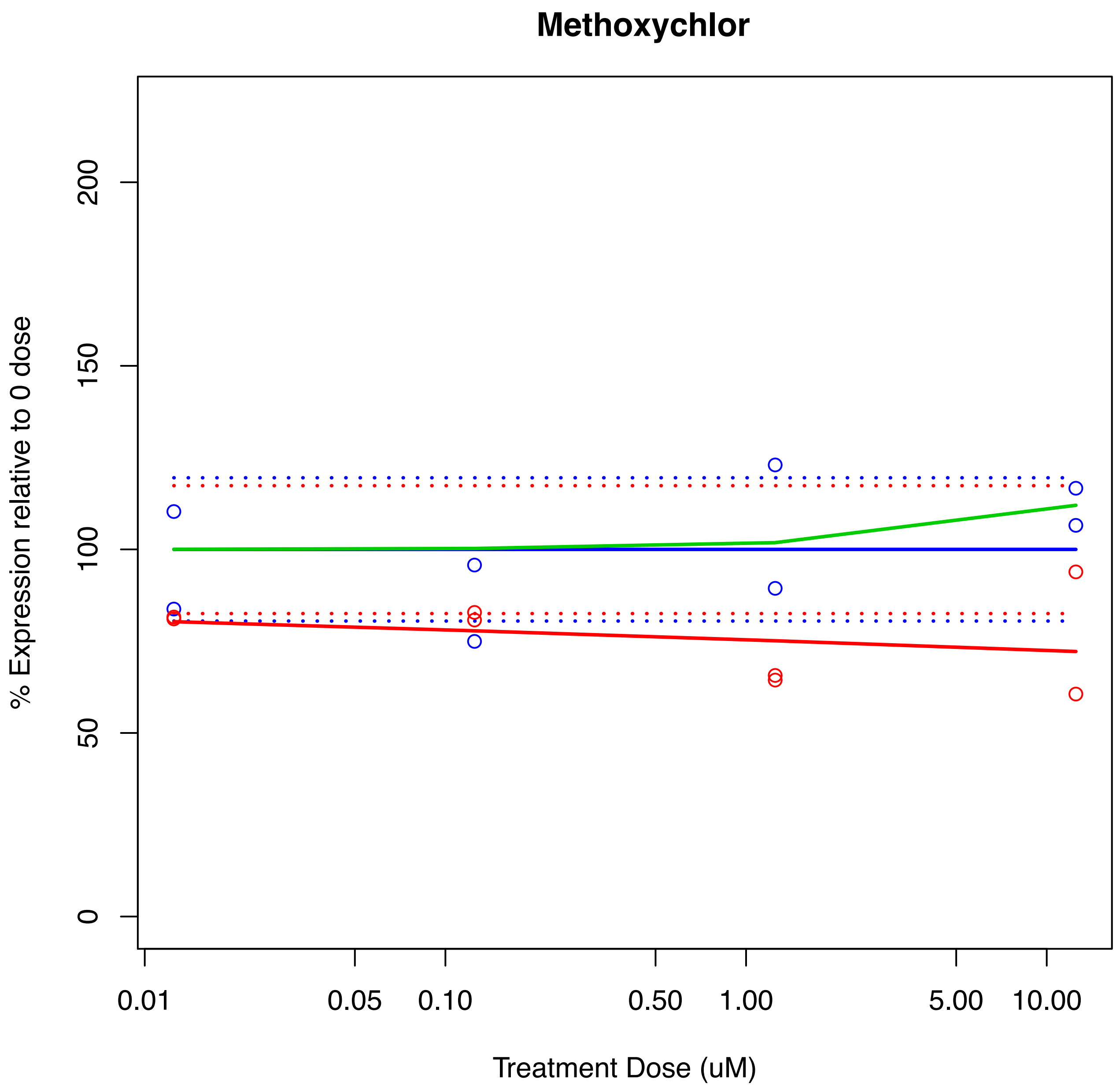

Methoxychlor
% Expression relative to 0 dose
0
50
100
150
200
0.01
0.05
0.10
0.50
1.00
5.00
10.00
Treatment Dose (uM)

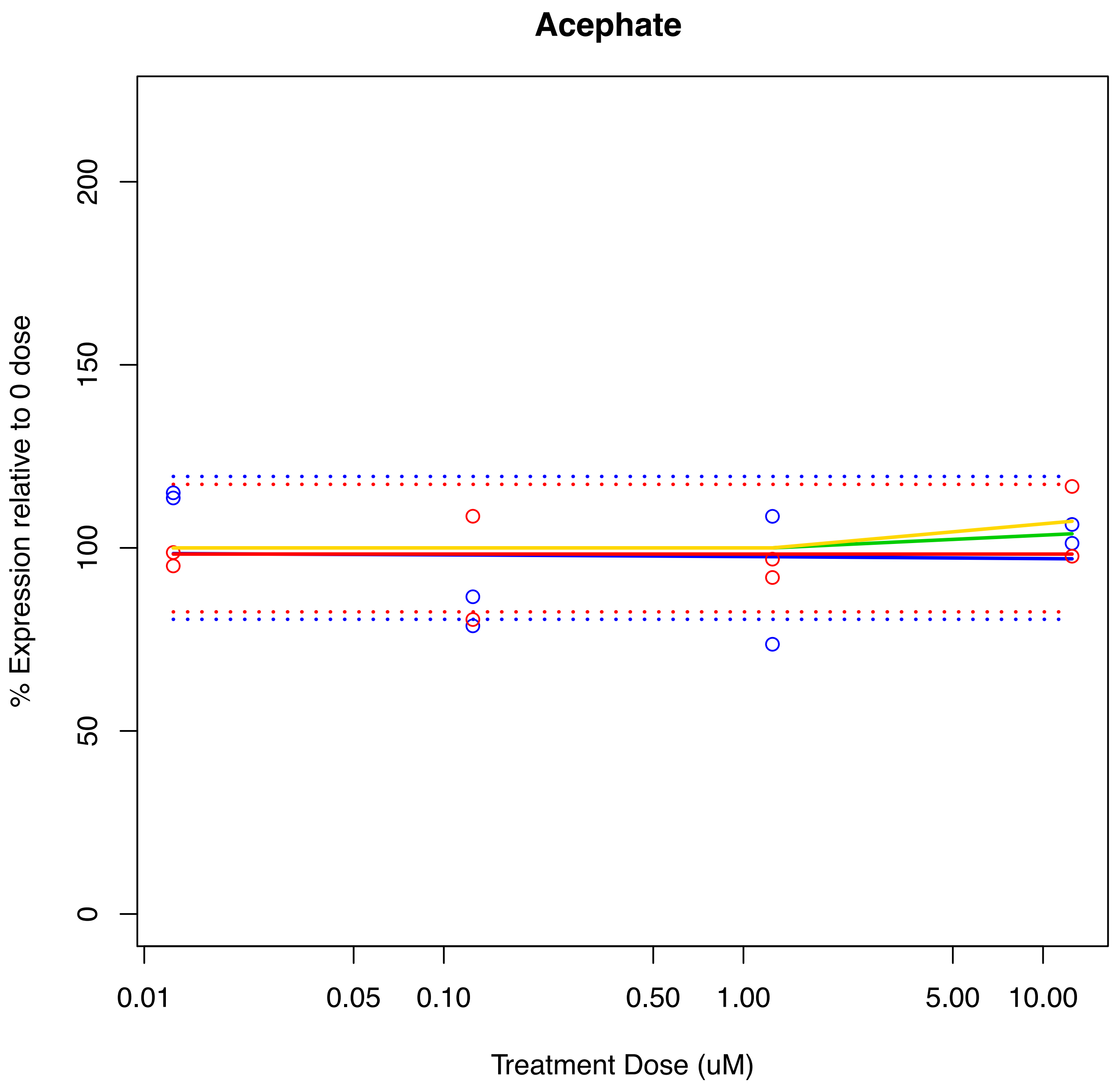
Acephate
% Expression relative to 0 dose
0
50
100
150
200
0.01
0.05
0.10
0.50
1.00
5.00
10.00
Treatment Dose (uM)

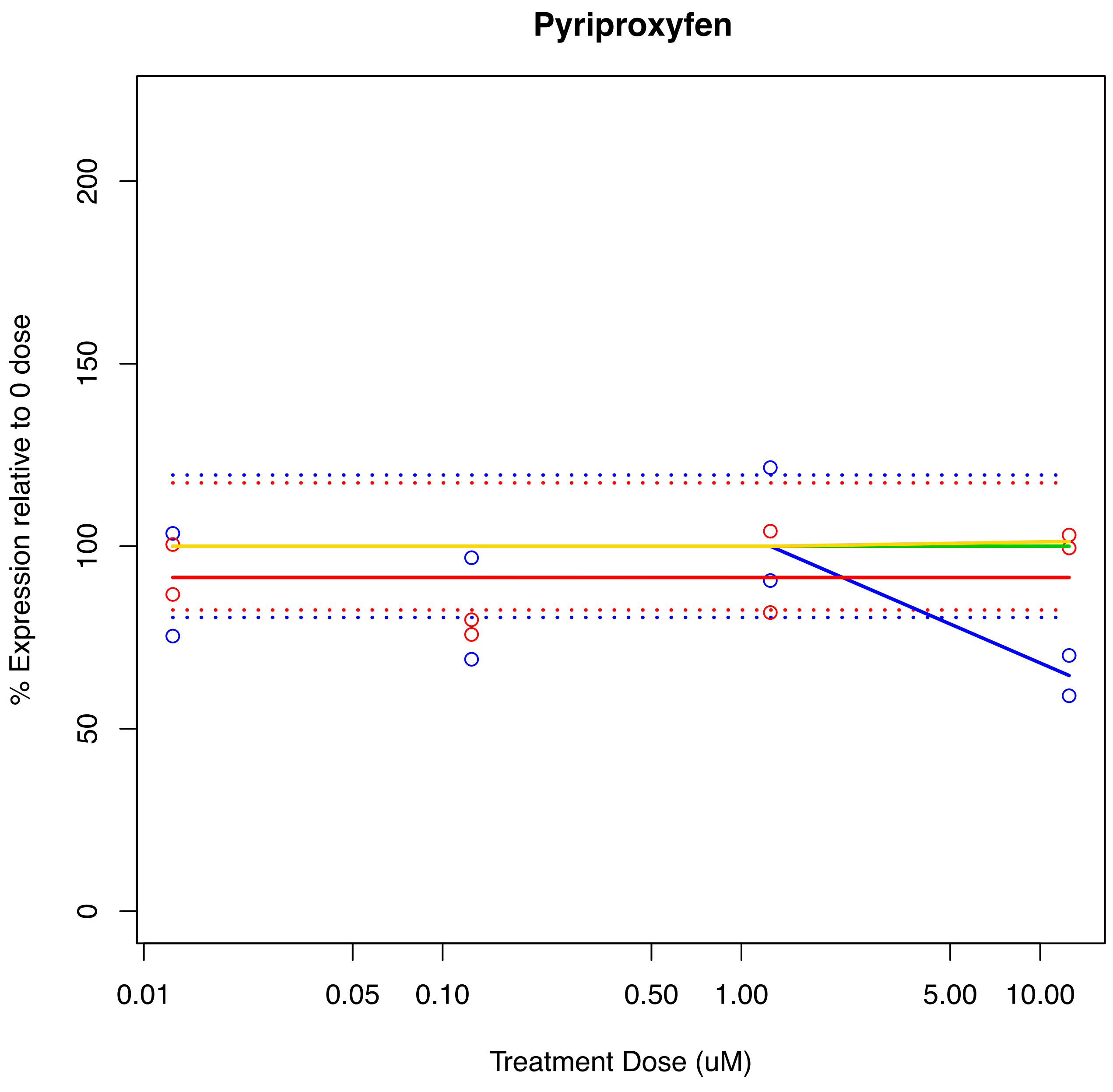
Pyriproxyfen
% Expression relative to 0 dose
0
50
100
150
200
0.01
0.05
0.10
0.50
1.00
5.00
10.00
Treatment Dose (uM)

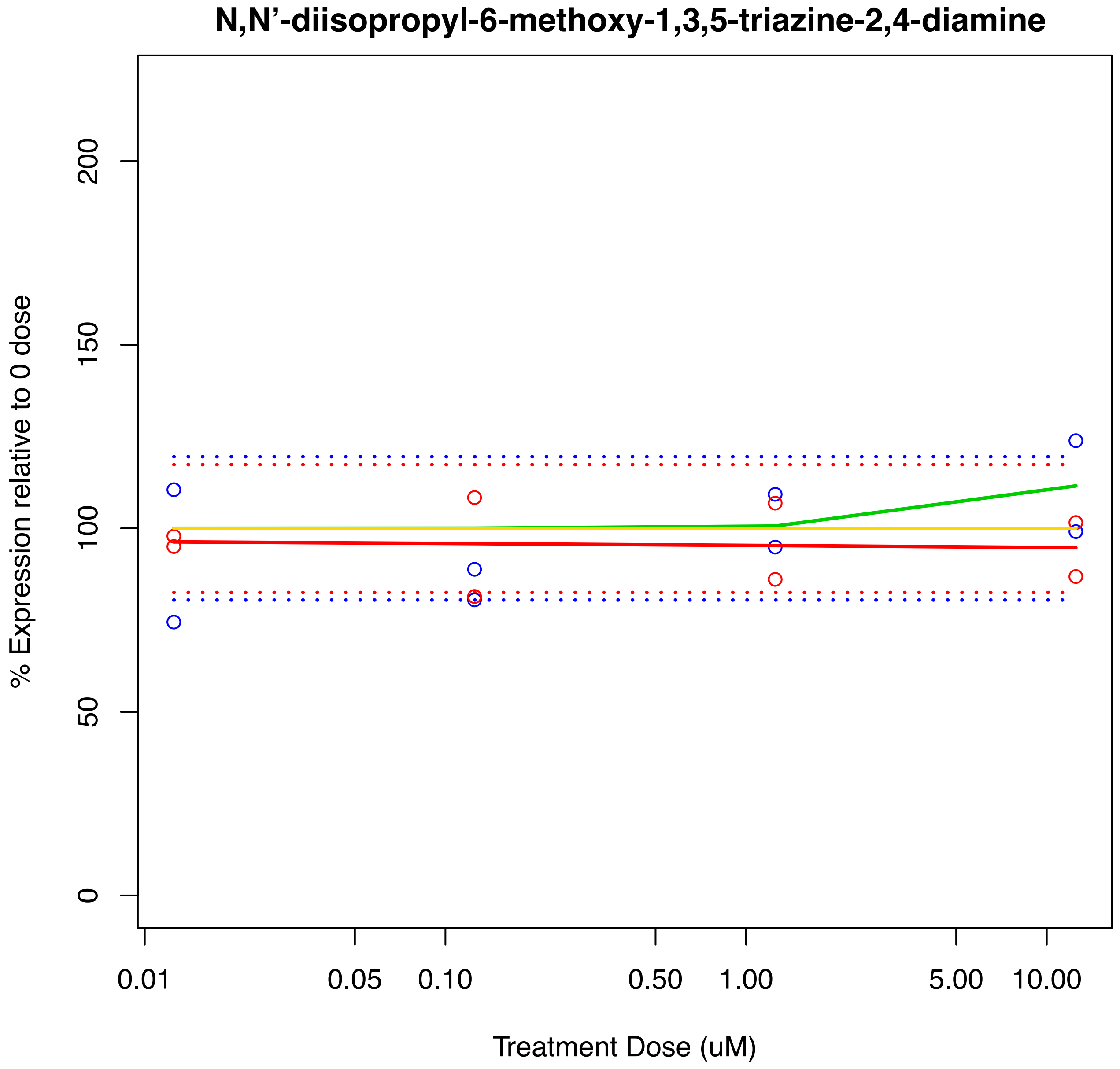
N,N’-diisopropyl-6-methoxy-1,3,5-triazine-2,4-diamine
% Expression relative to 0 dose
0
50
100
150
200
0.01
0.05
0.10
0.50
1.00
5.00
10.00
Treatment Dose (uM)

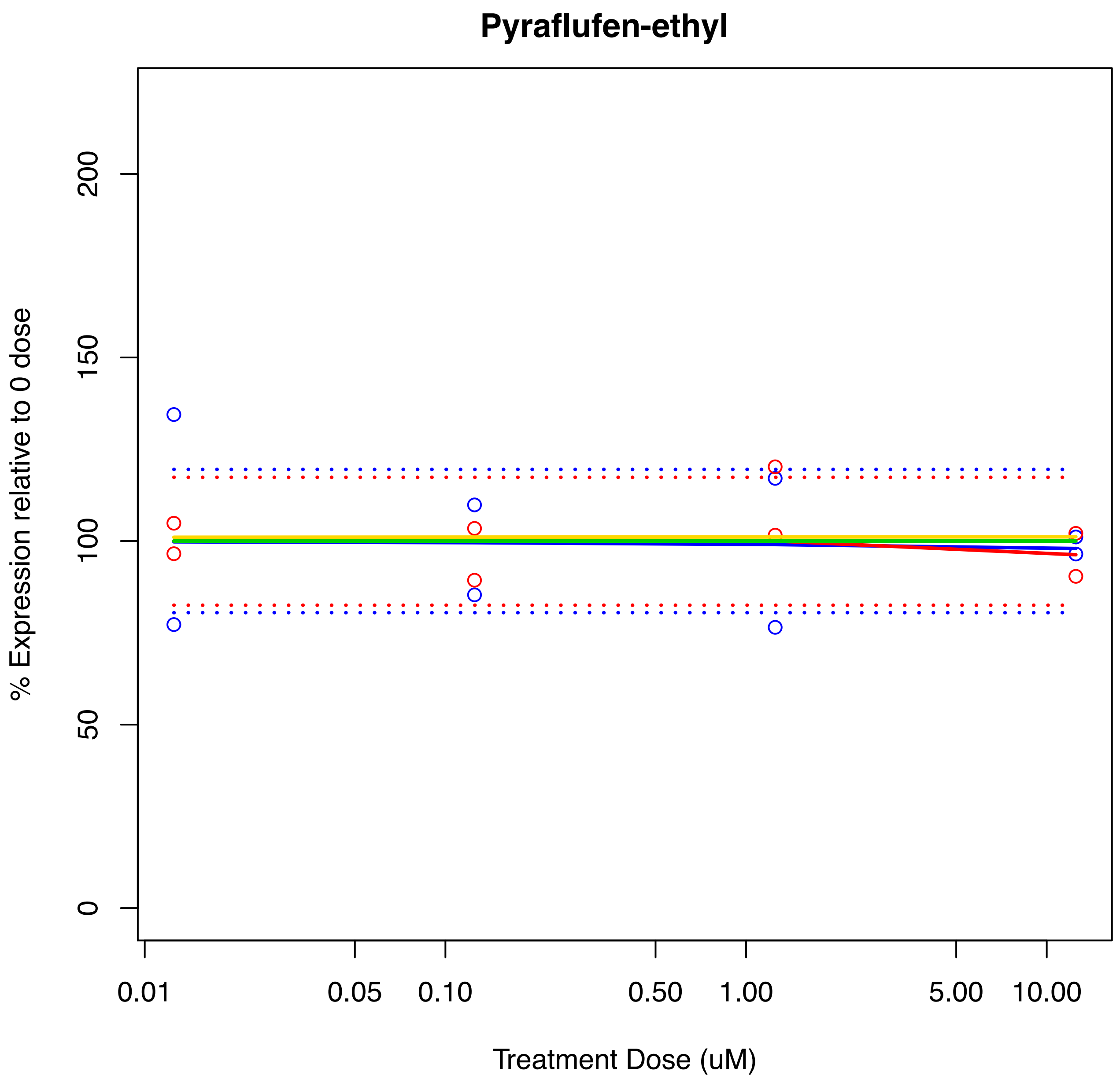
Pyraflufen-ethyl
% Expression relative to 0 dose
0
50
100
150
200
0.01
0.05
0.10
0.50
1.00
5.00
10.00
Treatment Dose (uM)

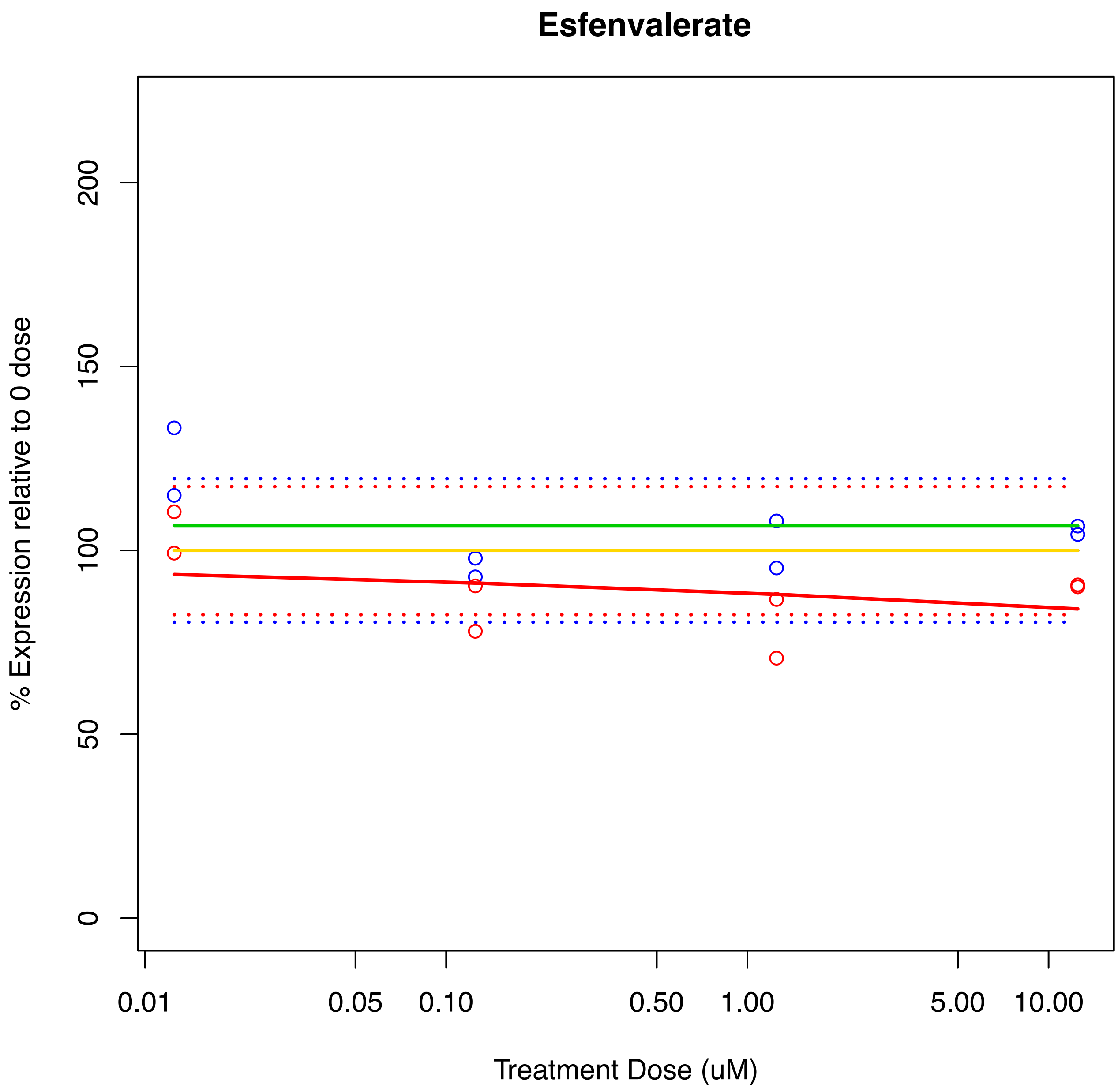

Esfenvalerate
% Expression relative to 0 dose
0
50
100
150
200
0.01
0.05
0.10
0.50
1.00
5.00
10.00
Treatment Dose (uM)

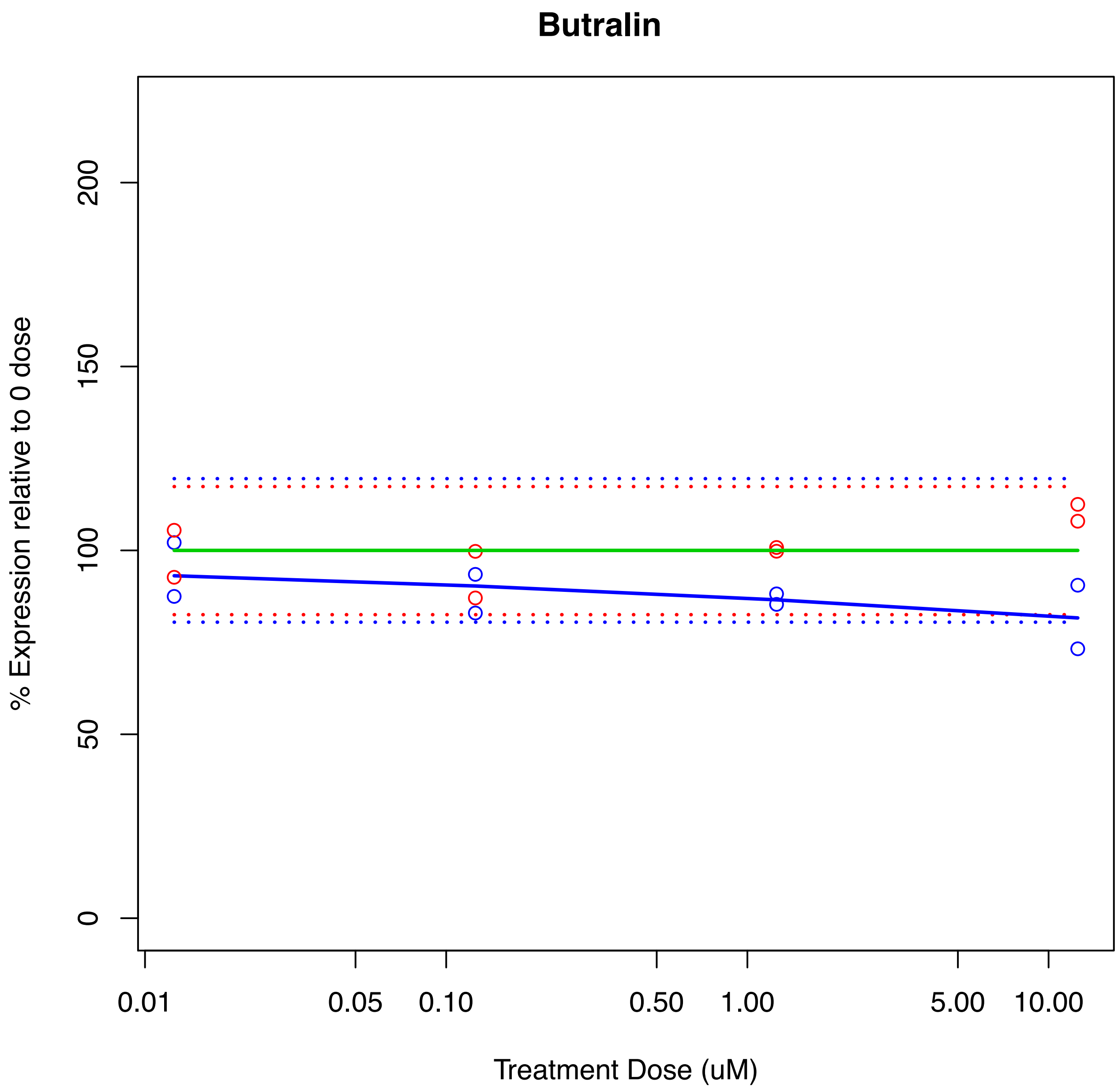

Butralin
% Expression relative to 0 dose
0
50
100
150
200
0.01
0.05
0.10
0.50
1.00
5.00
10.00
Treatment Dose (uM)

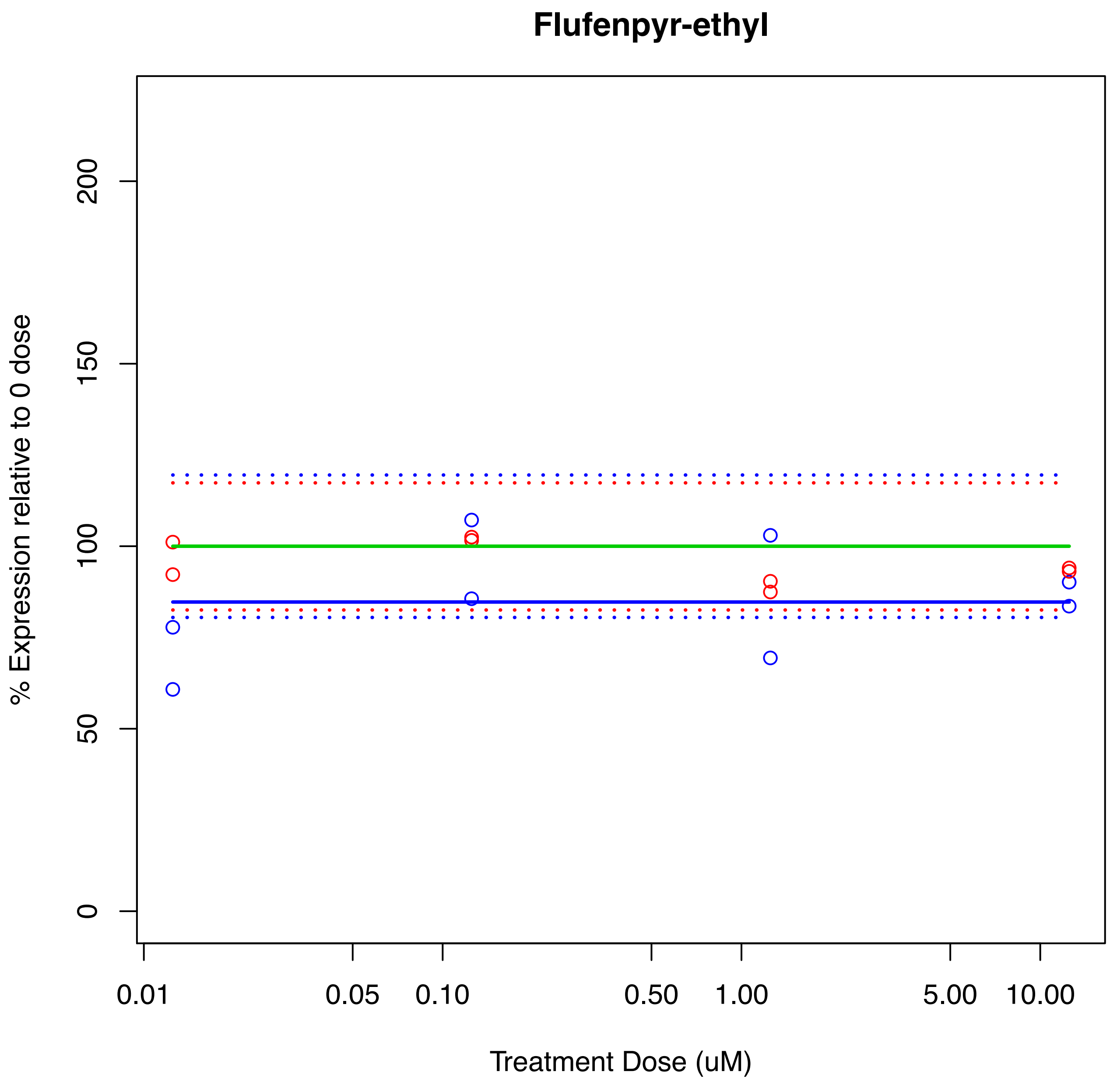

Flufenpyr-ethyl
% Expression relative to 0 dose
0
50
100
150
200
0.01
0.05
0.10
0.50
1.00
5.00
10.00
Treatment Dose (uM)

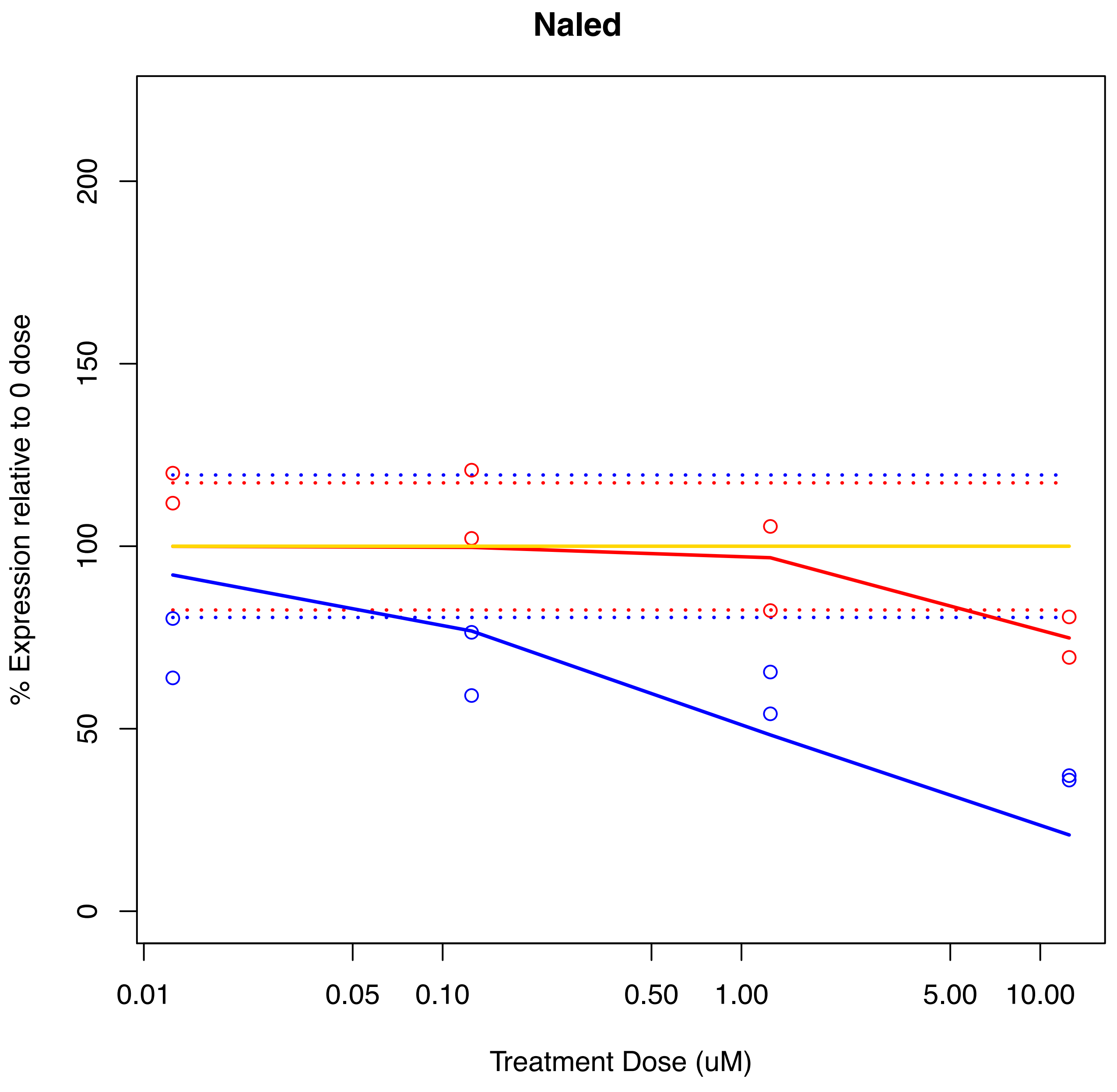
Naled
% Expression relative to 0 dose
0
50
100
150
200
0.01
0.05
0.10
0.50
1.00
5.00
10.00
Treatment Dose (uM)

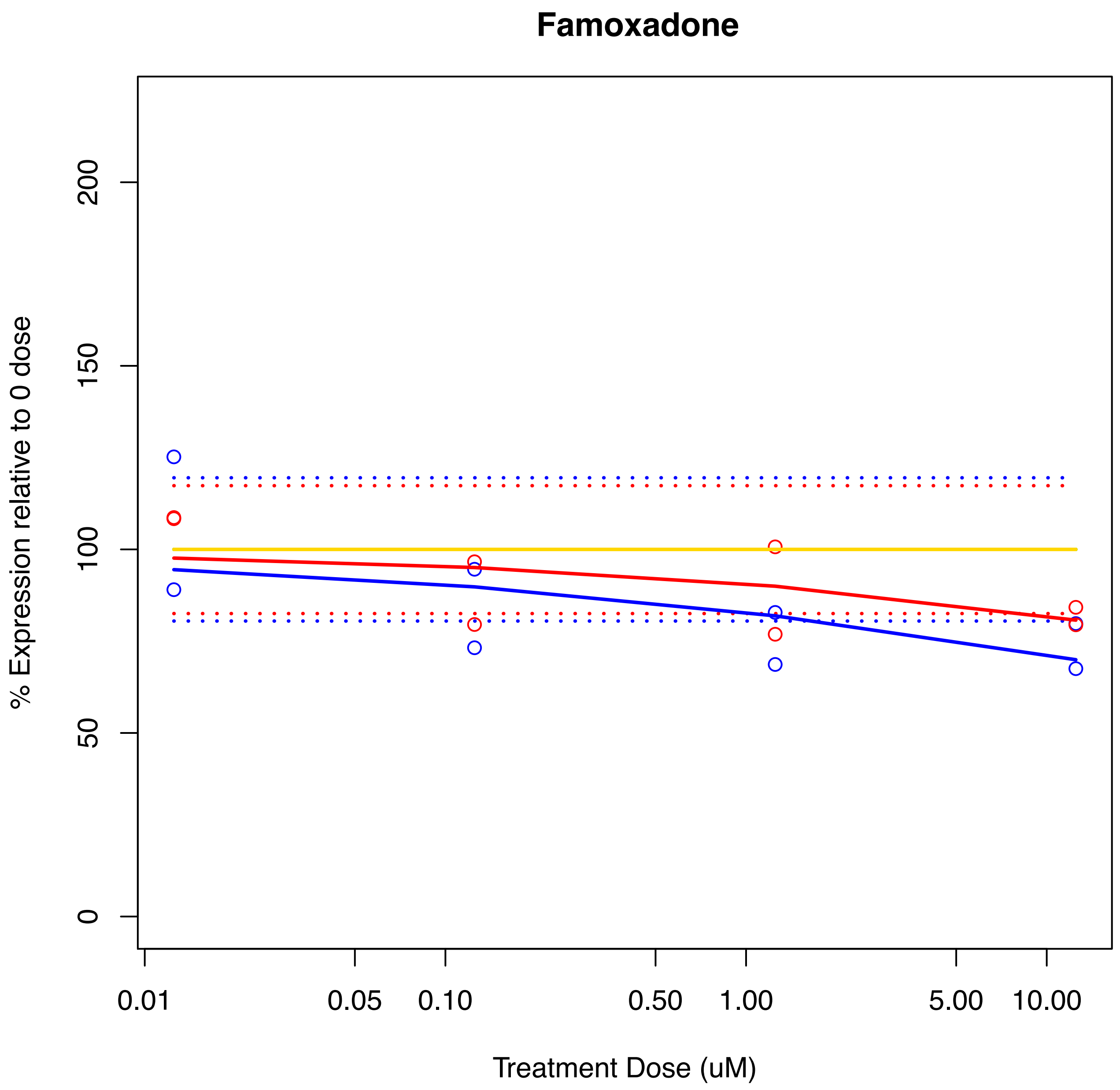

Famoxadone
% Expression relative to 0 dose
0
50
100
150
200
0.01
0.05
0.10
0.50
1.00
5.00
10.00
Treatment Dose (uM)

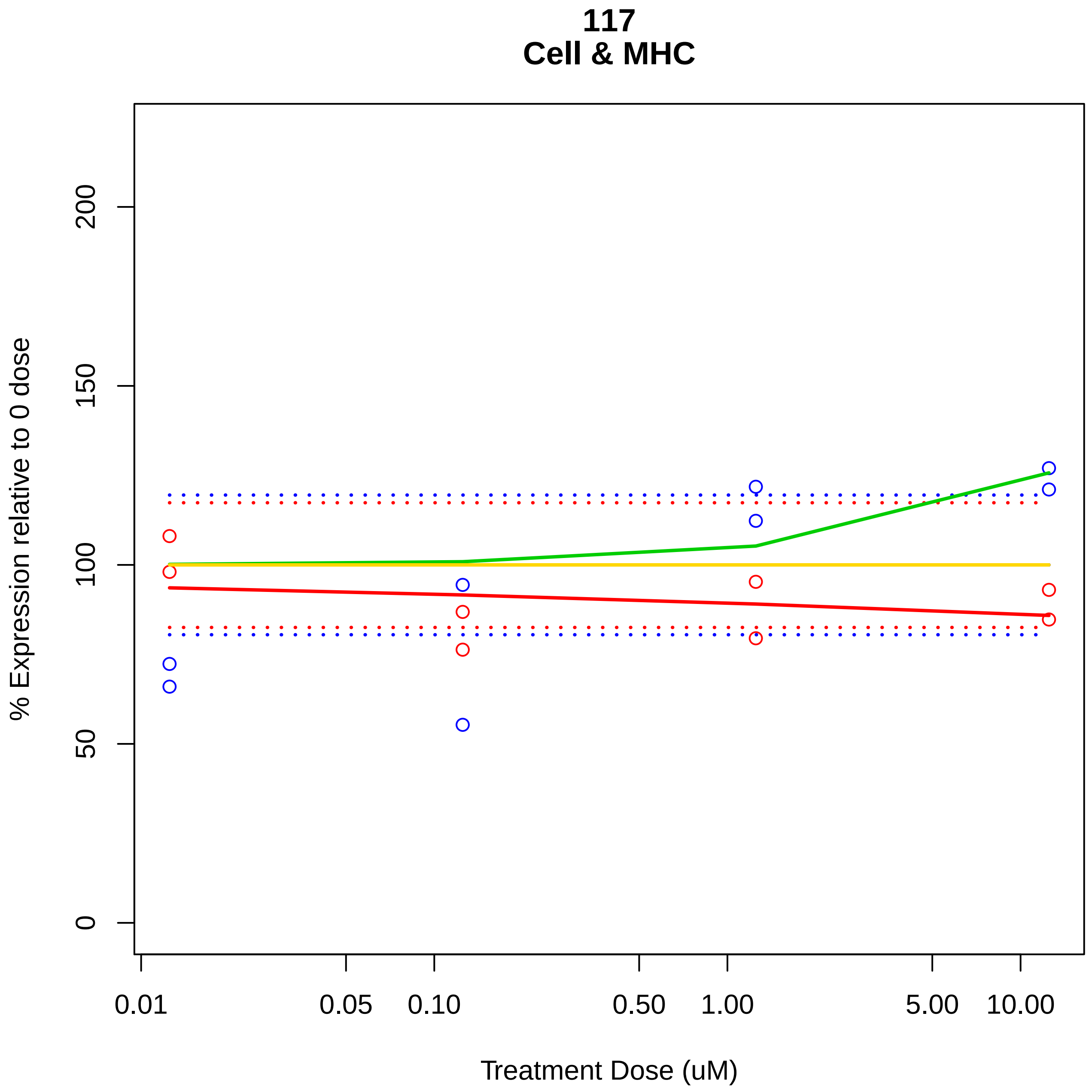

117
Cell & MHC
% Expression relative to 0 dose
0
50
100
150
200
0.01
0.05
0.10
0.50
1.00
5.00
10.00
Treatment Dose (uM)

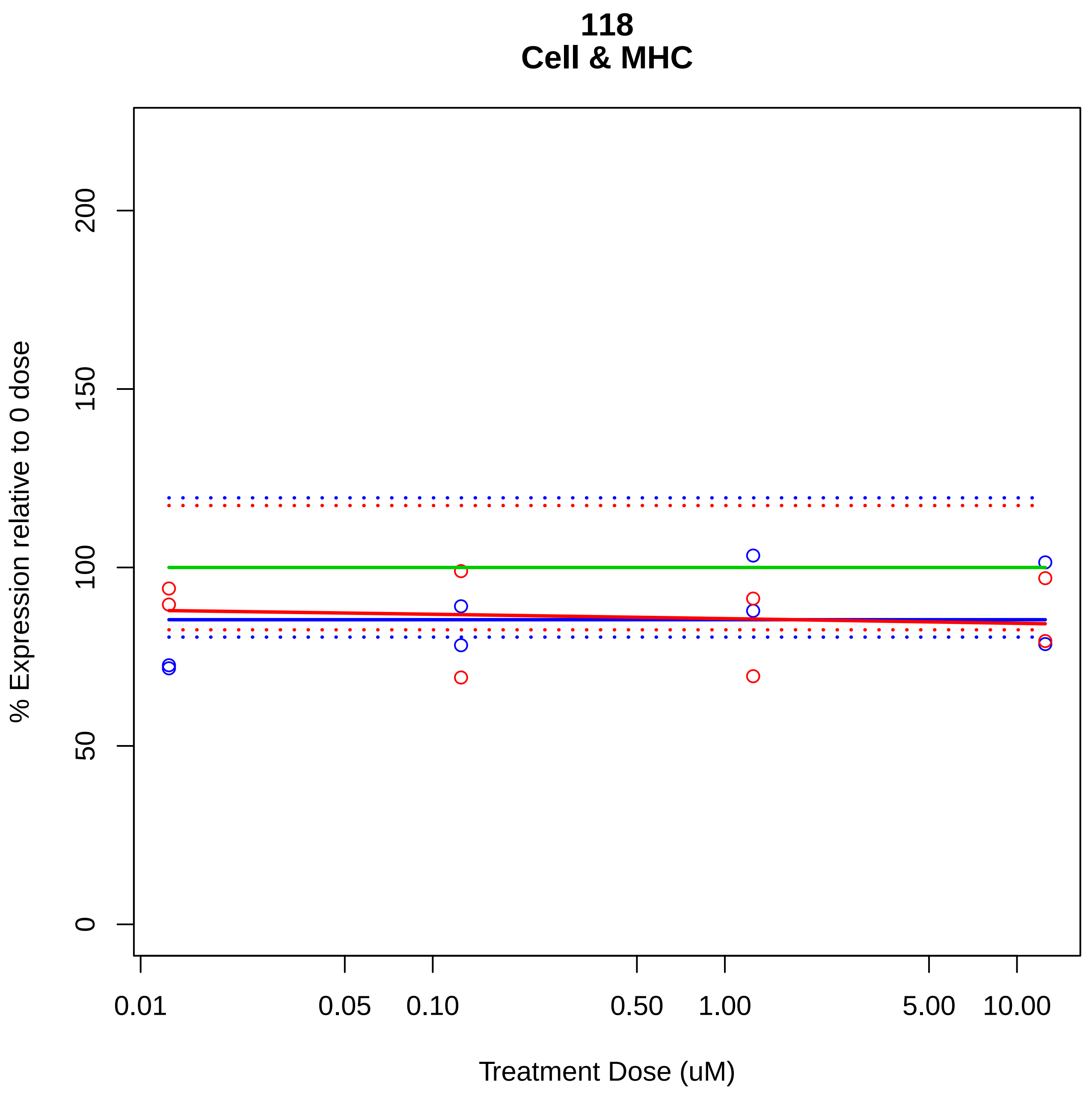
118
Cell & MHC
% Expression relative to 0 dose
0
50
100
150
200
0.01
0.05
0.10
0.50
1.00
5.00
10.00
Treatment Dose (uM)

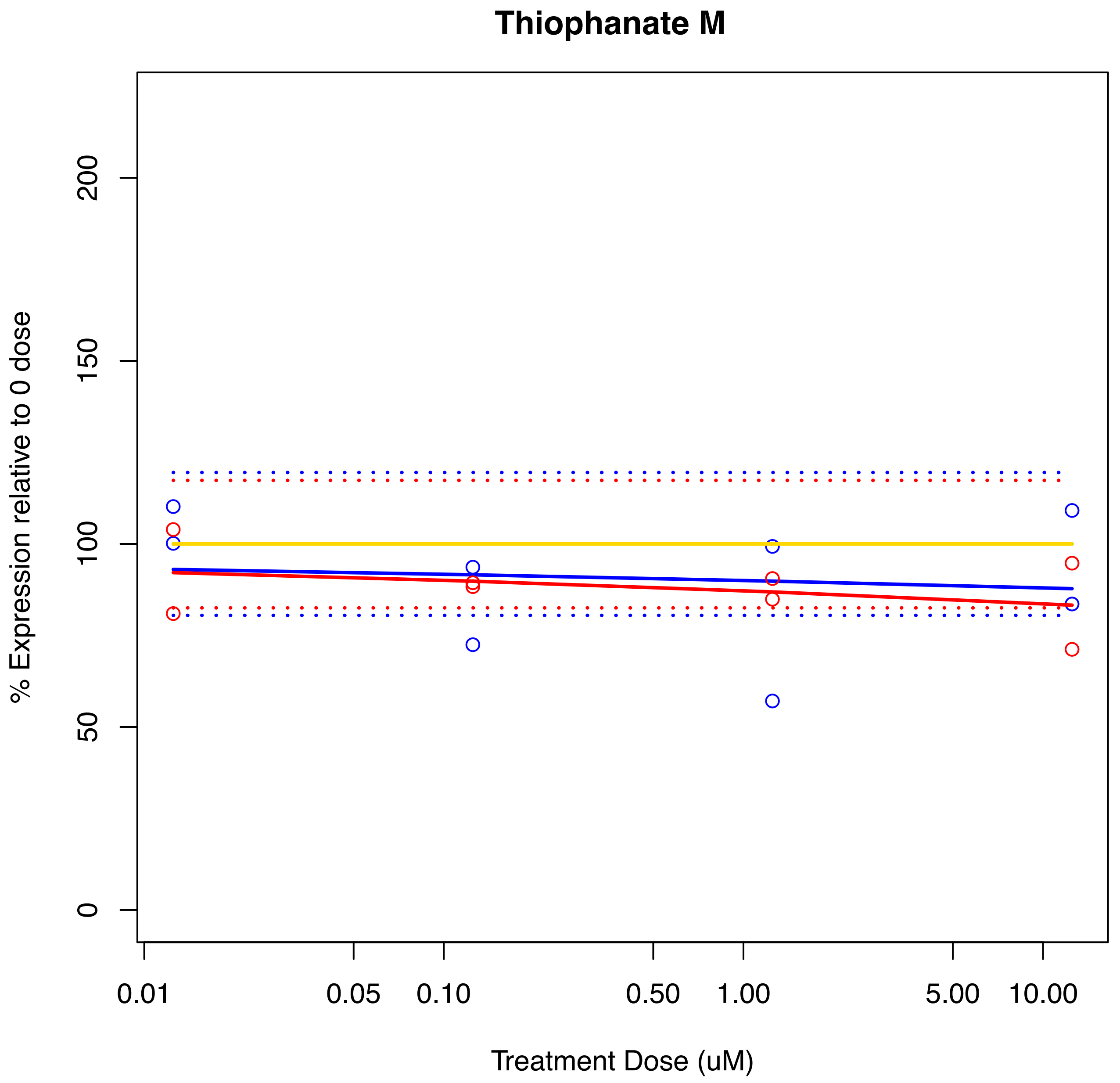
Thiophanate M
% Expression relative to 0 dose
0
50
100
150
200
0.01
0.05
0.10
0.50
1.00
5.00
10.00
Treatment Dose (uM)

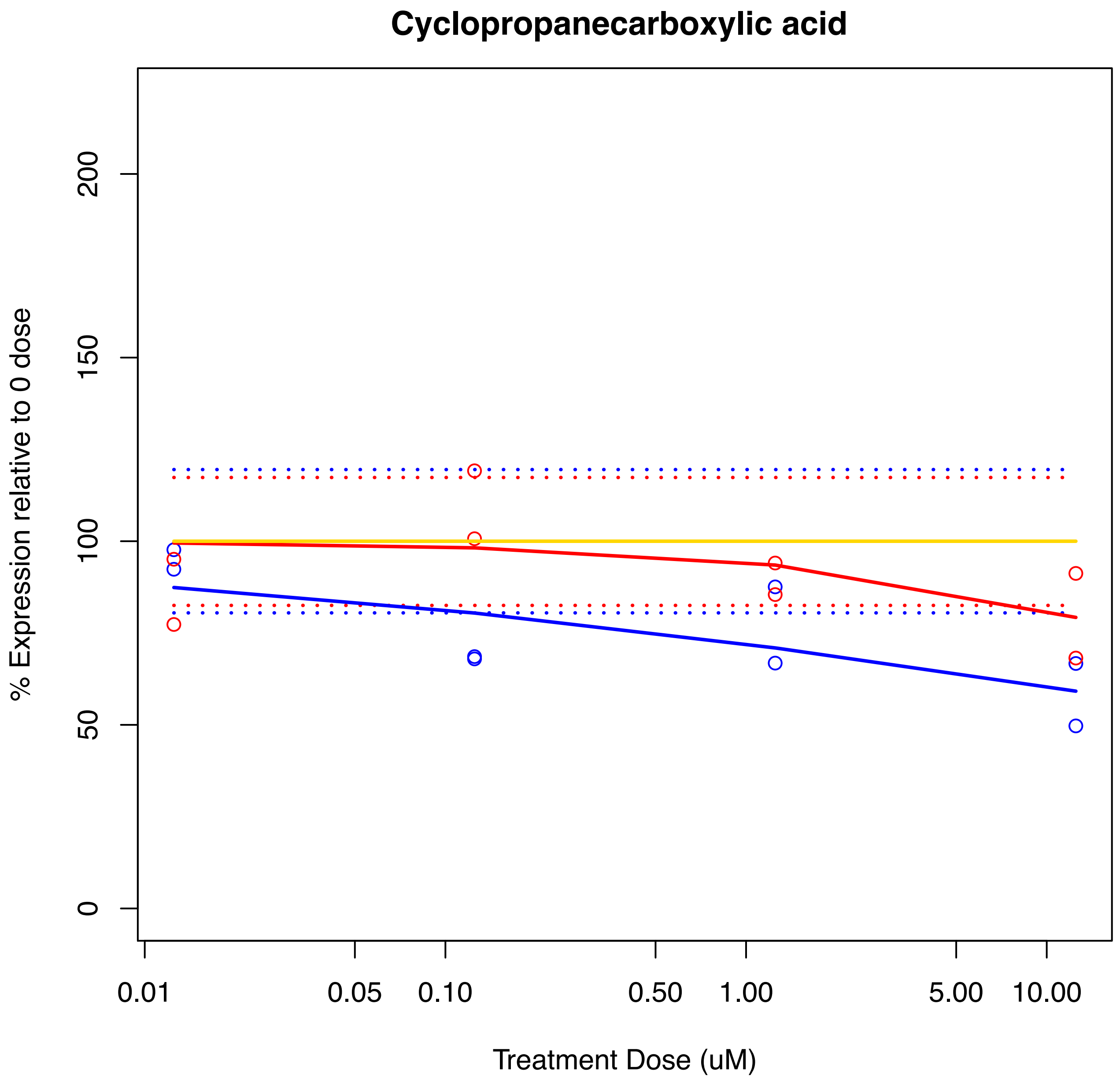

Cyclopropanecarboxylic acid
% Expression relative to 0 dose
0
50
100
150
200
0.01
0.05
0.10
0.50
1.00
5.00
10.00
Treatment Dose (uM)

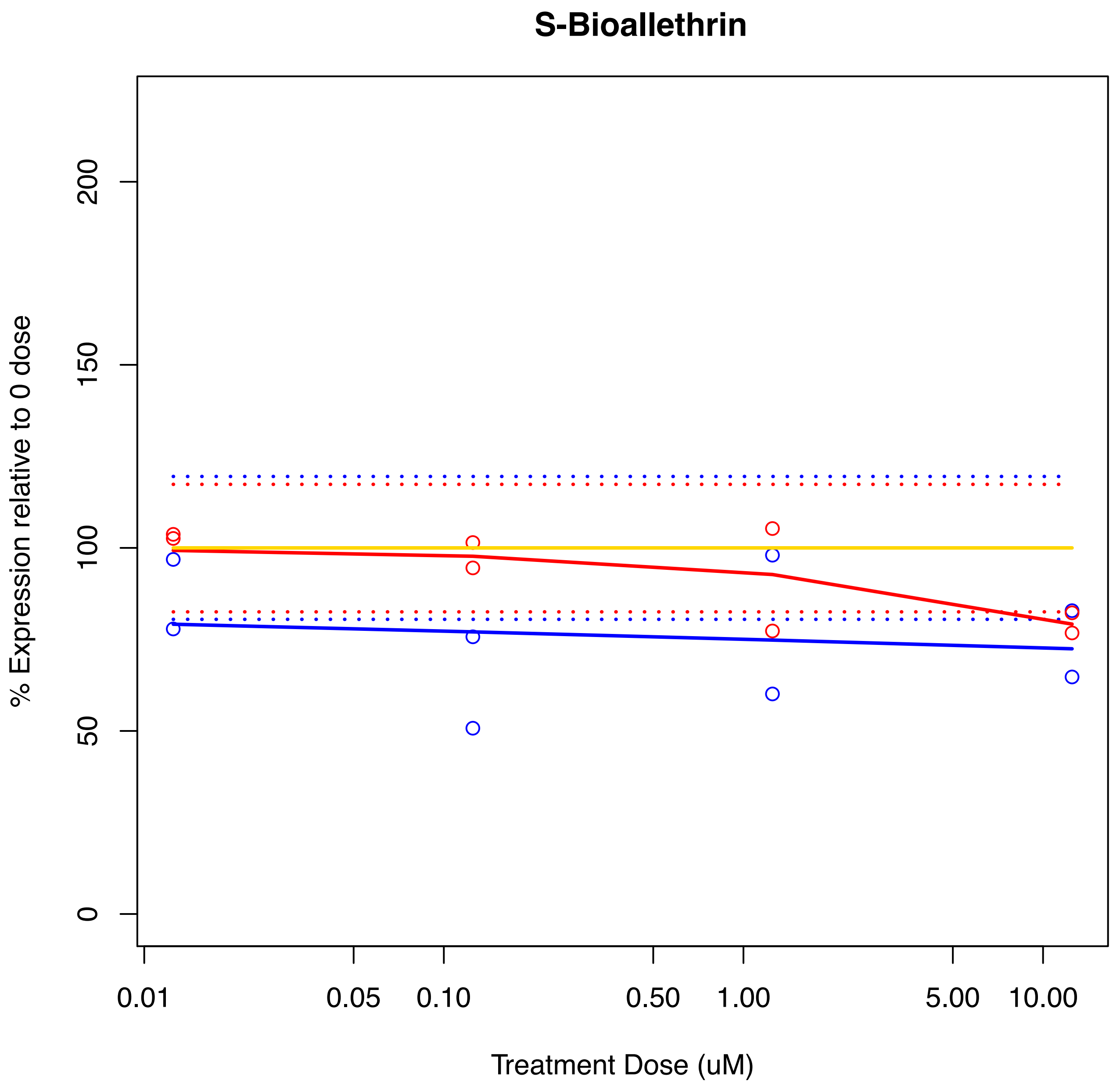

S-Bioallethrin
% Expression relative to 0 dose
0
50
100
150
200
0.01
0.05
0.10
0.50
1.00
5.00
10.00
Treatment Dose (uM)

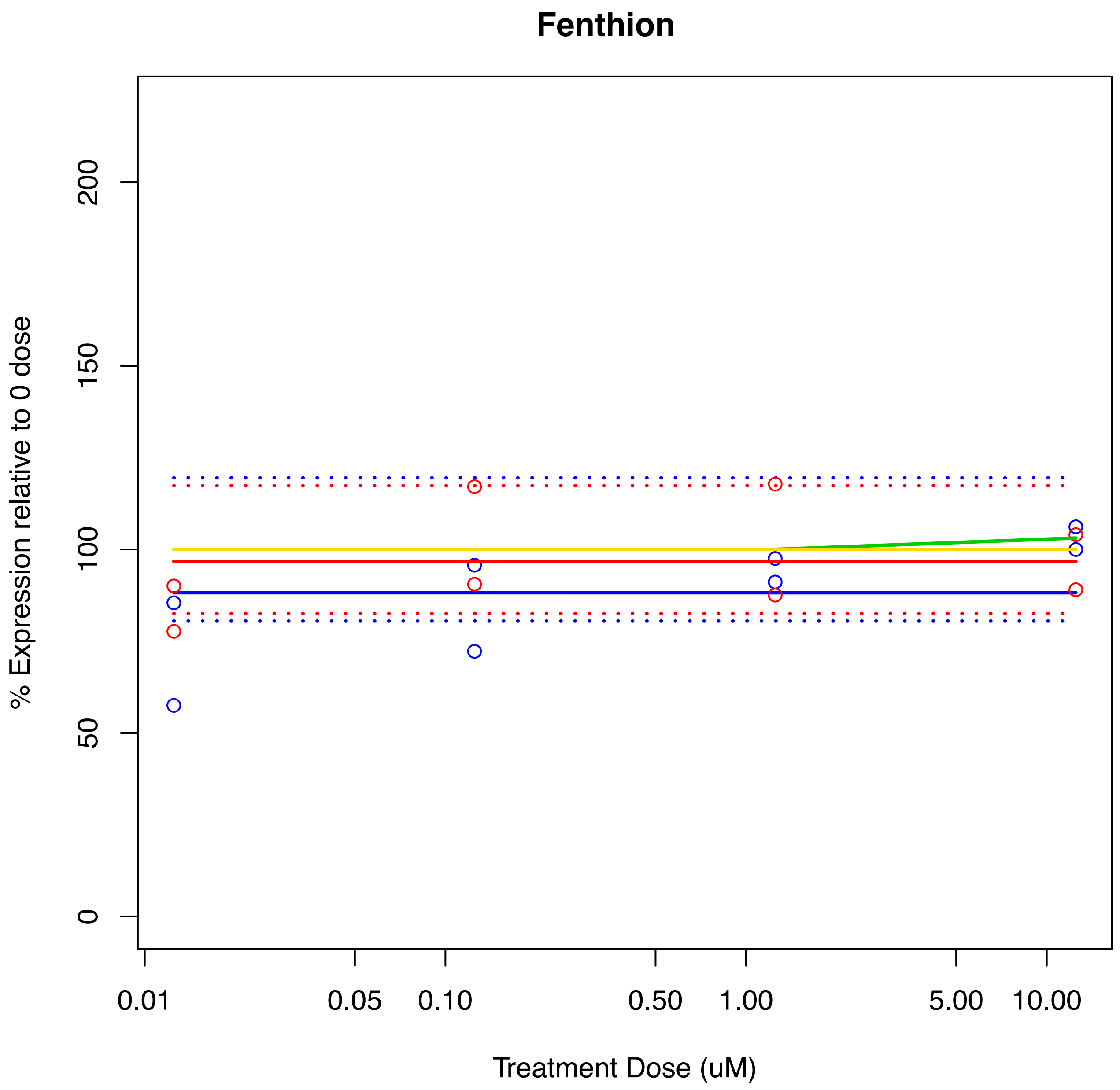

Fenthion
% Expression relative to 0 dose
0
50
100
150
200
0.01
0.05
0.10
0.50
1.00
5.00
10.00
Treatment Dose (uM)

2-Cyclohexen-1-one, 2-{1-{{(3-chloro-2-propenyl)oxy}imino}propyl}-3-hydroxy-5-(tetrahydro-2H-pyran-4-yl)-,(E,?)-

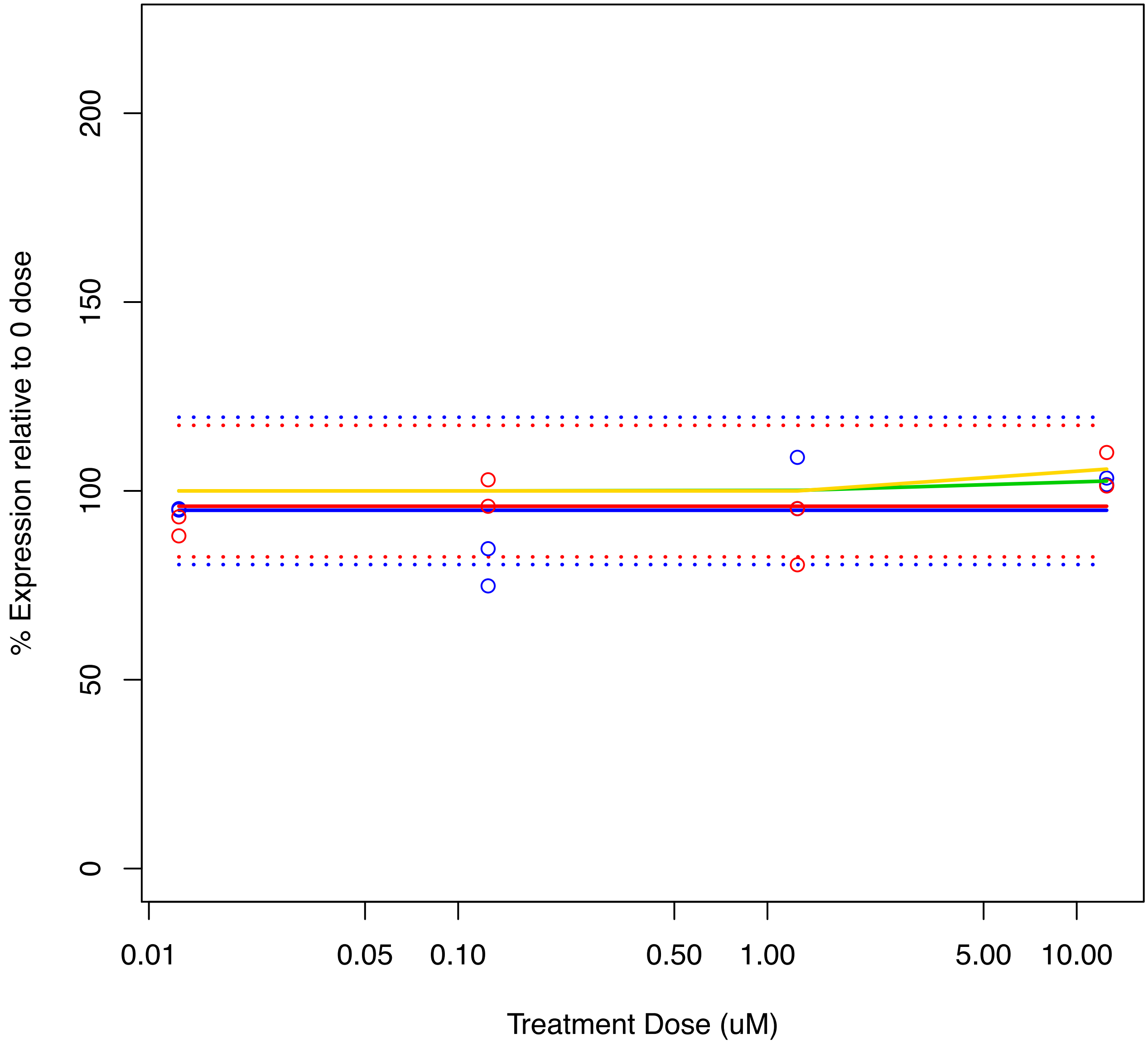

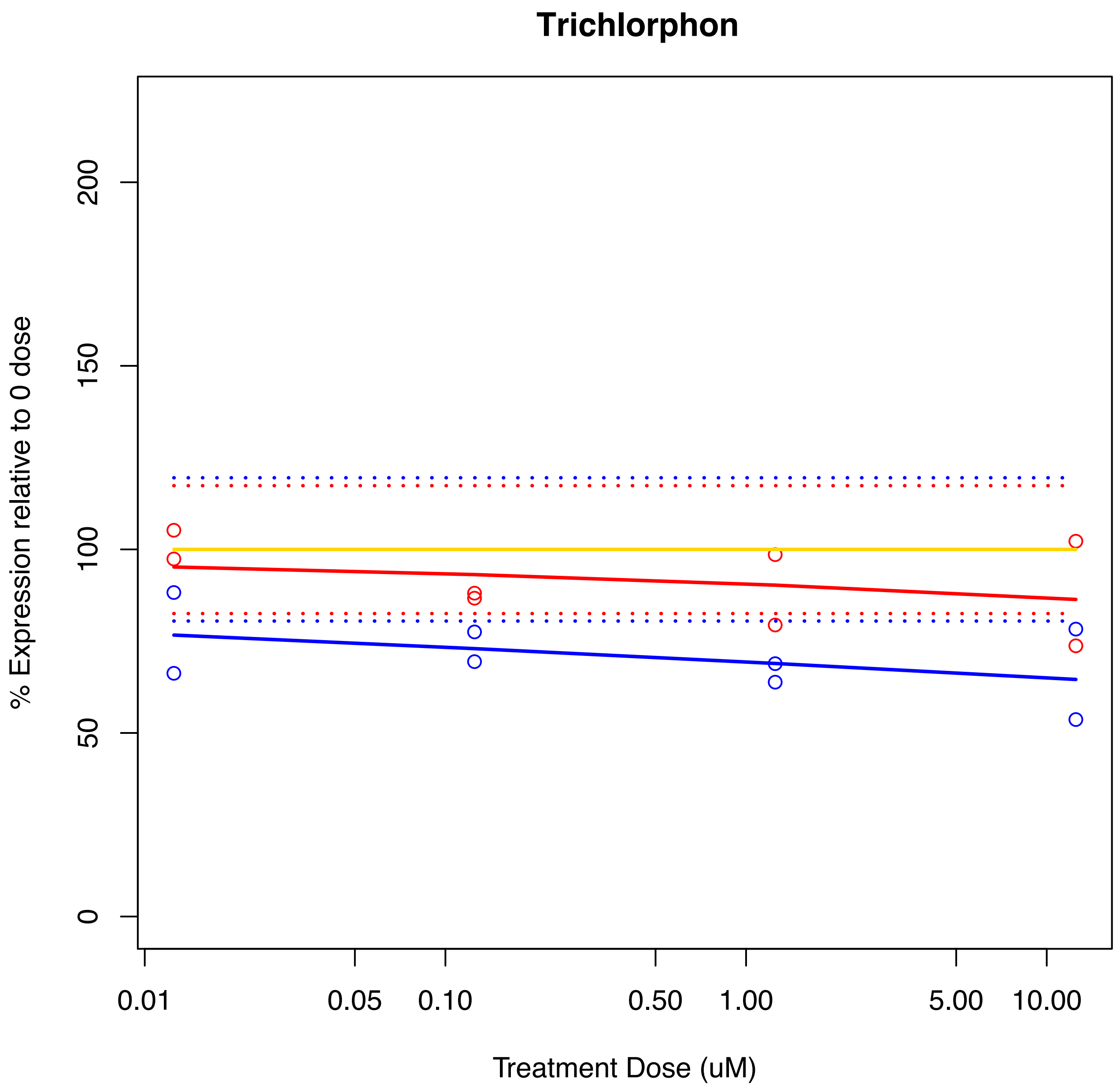
Trichlorphon
% Expression relative to 0 dose
0
50
100
150
200
0.01
0.05
0.10
0.50
1.00
5.00
10.00
Treatment Dose (uM)

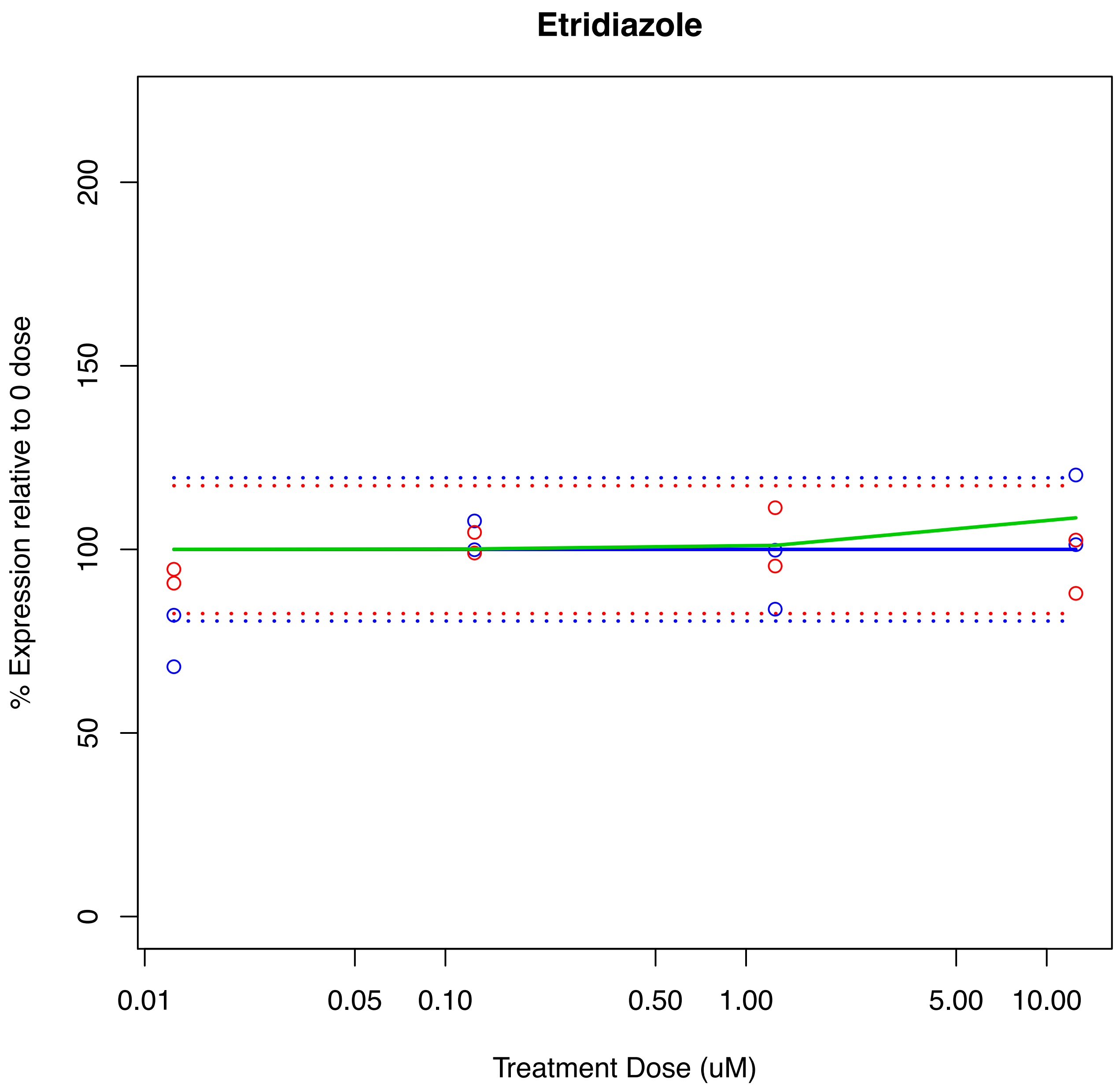
Etridiazole
% Expression relative to 0 dose
0
50
100
150
200
0.01
0.05
0.10
0.50
1.00
5.00
10.00
Treatment Dose (uM)

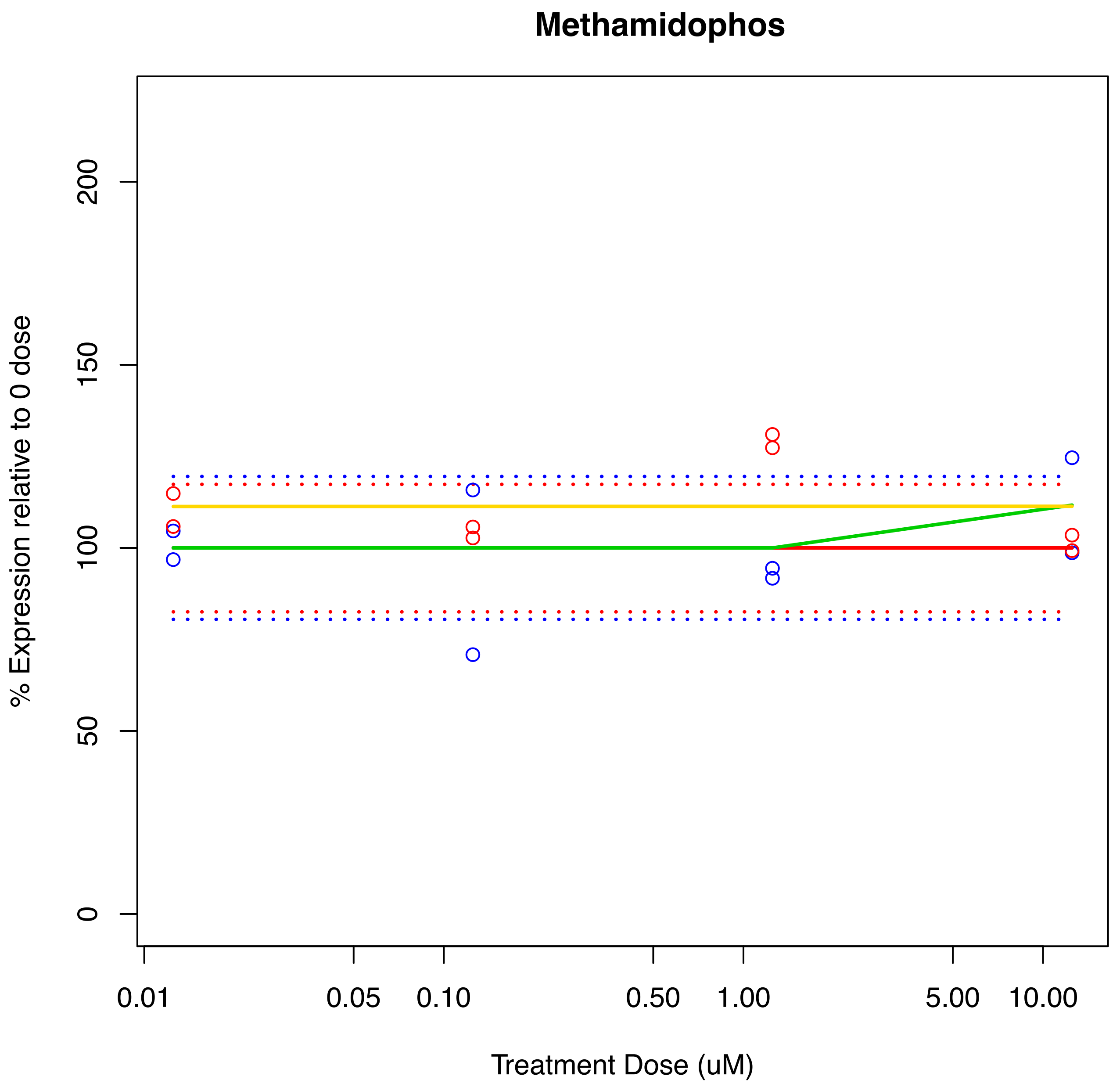
Methamidophos
% Expression relative to 0 dose
0
50
100
150
200
0.01
0.05
0.10
0.50
1.00
5.00
10.00
Treatment Dose (uM)

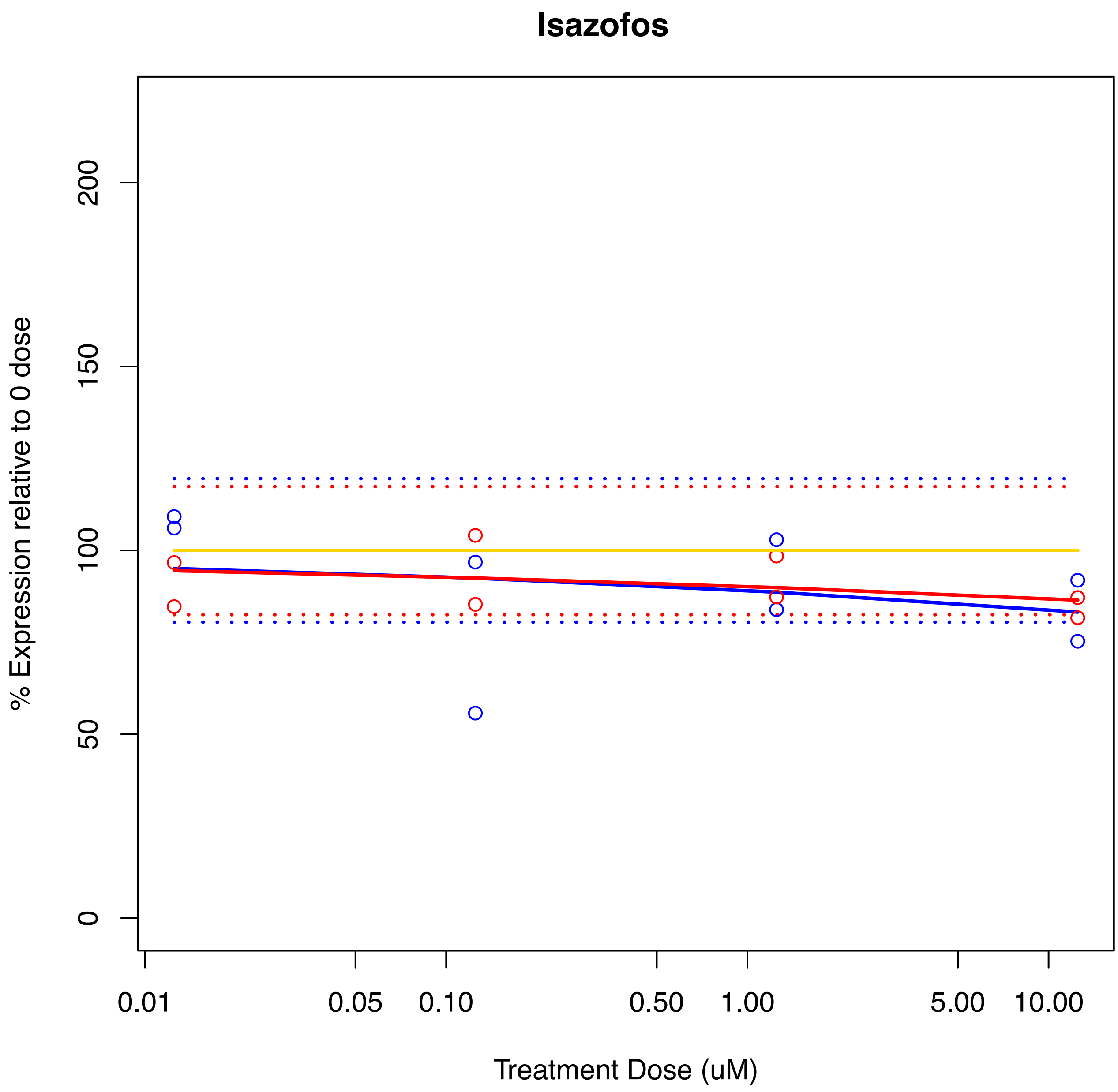
Isazofos
% Expression relative to 0 dose
0
50
100
150
200
0.01
0.05
0.10
0.50
1.00
5.00
10.00
Treatment Dose (uM)

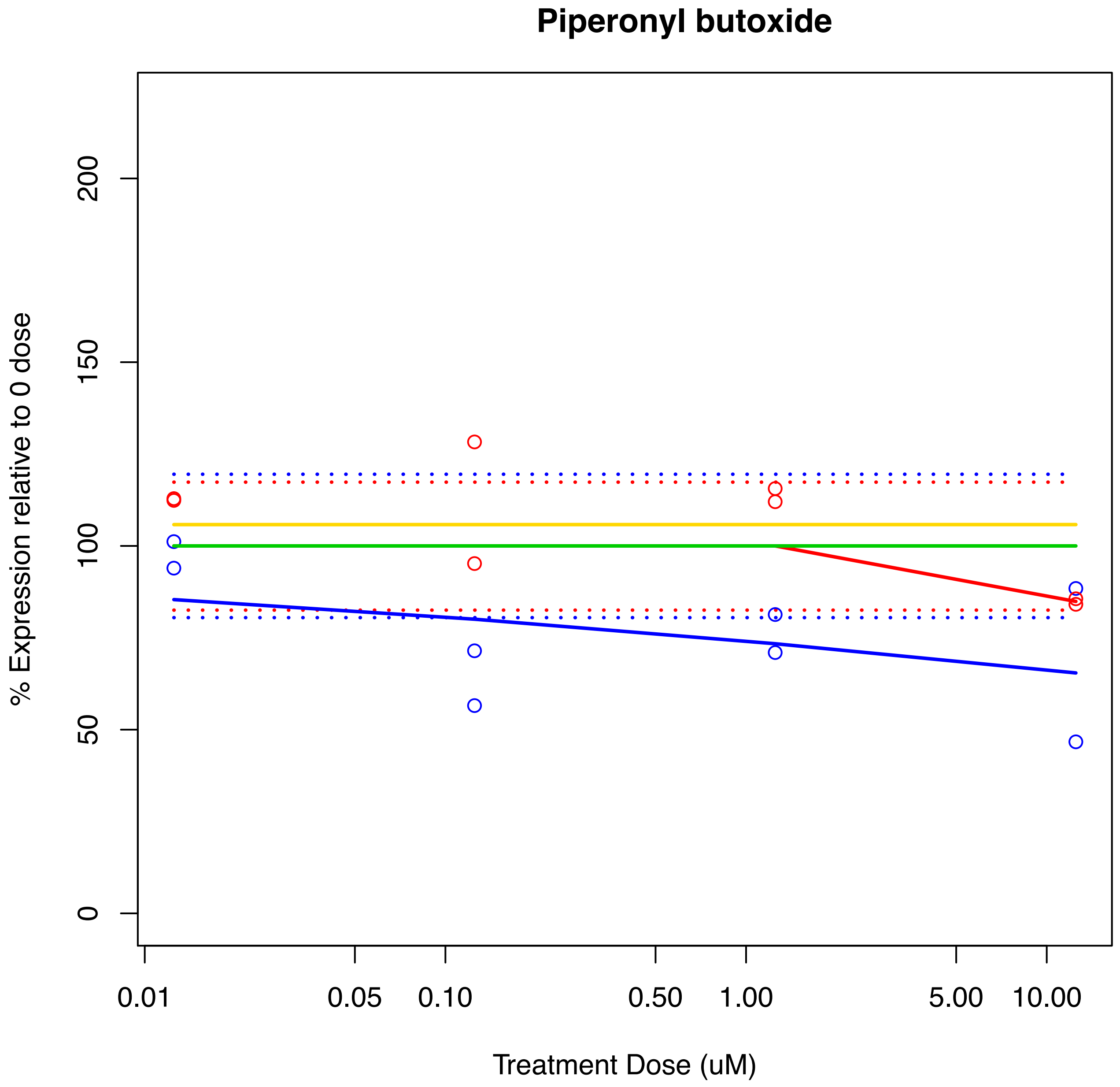

Piperonyl butoxide
% Expression relative to 0 dose
0
50
100
150
200
0.01
0.05
0.10
0.50
1.00
5.00
10.00
Treatment Dose (uM)

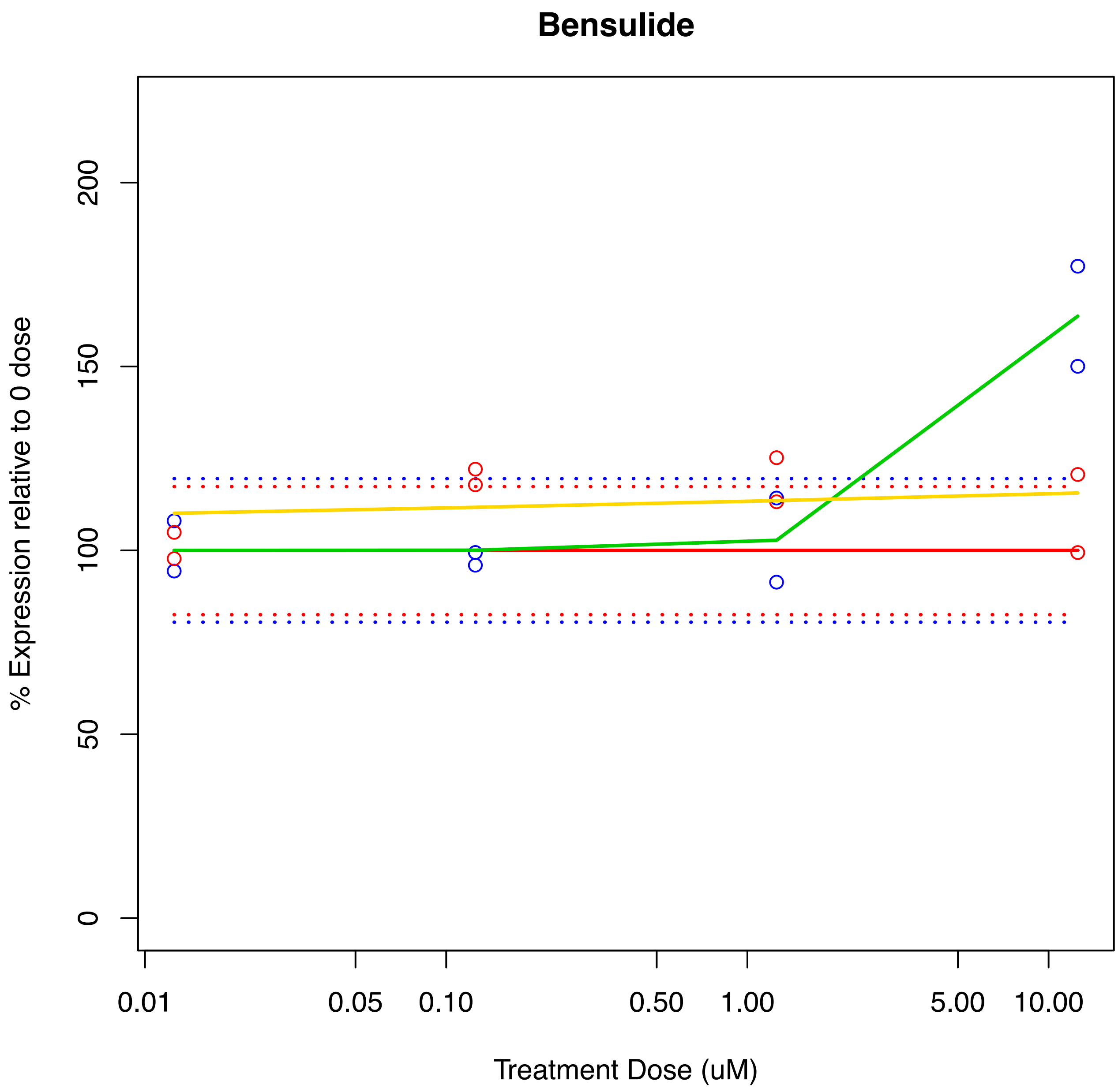

Bensulide
% Expression relative to 0 dose
0
50
100
150
200
0.01
0.05
0.10
0.50
1.00
5.00
10.00
Treatment Dose (uM)

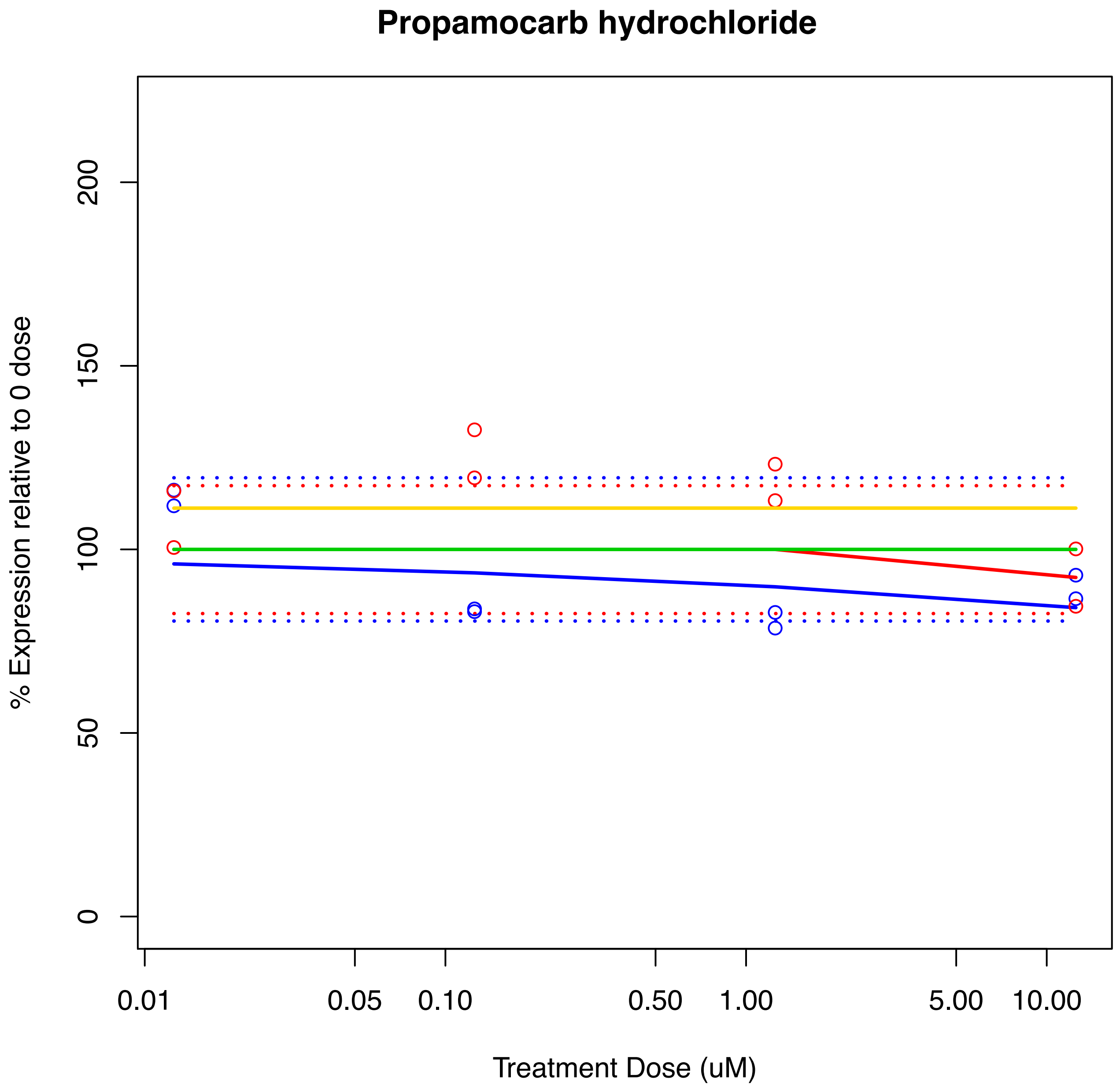
Propamocarb hydrochloride
% Expression relative to 0 dose
0
50
100
150
200
0.01
0.05
0.10
0.50
1.00
5.00
10.00
Treatment Dose (uM)

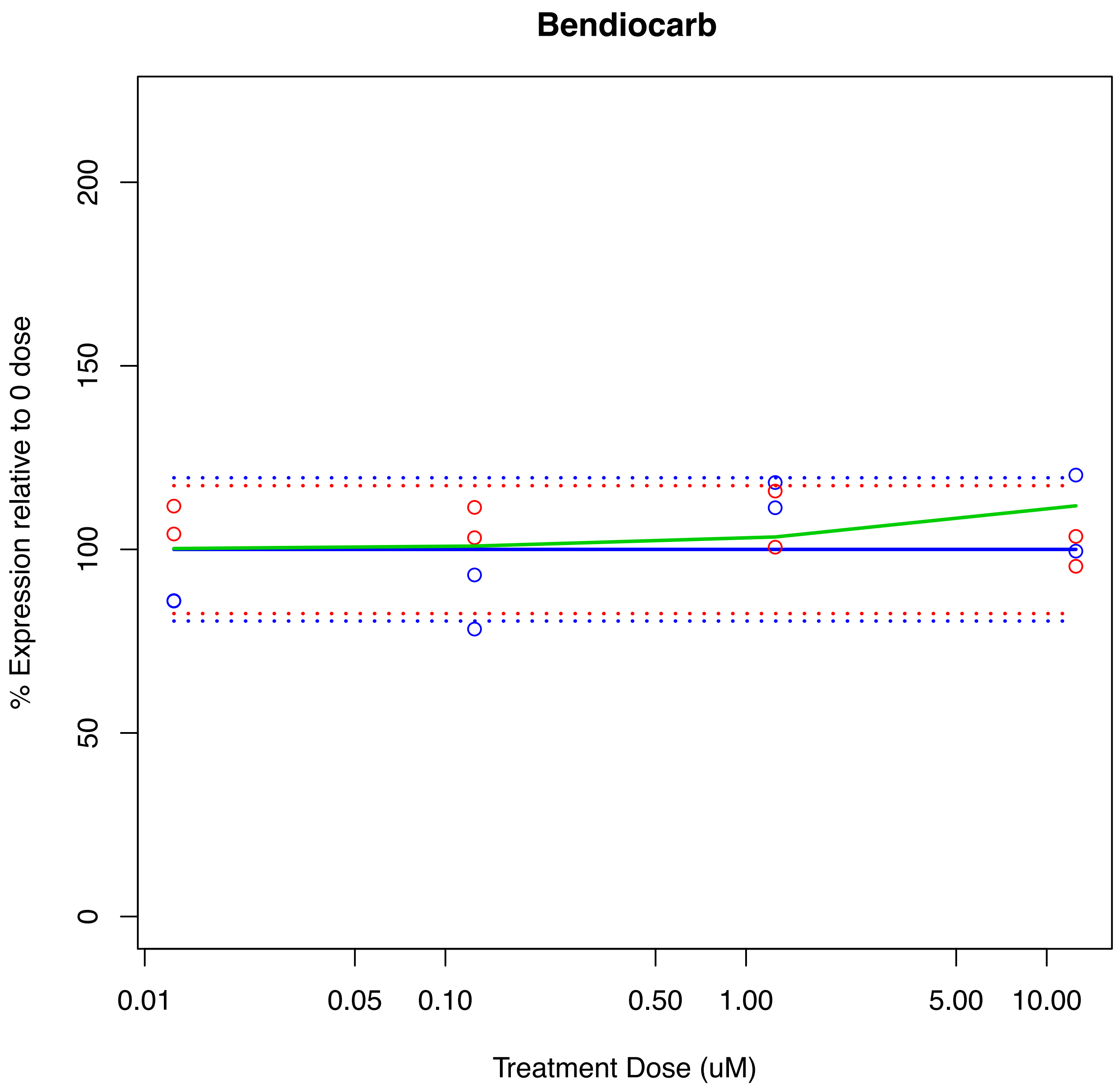

Bendiocarb
% Expression relative to 0 dose
0
50
100
150
200
0.01
0.05
0.10
0.50
1.00
5.00
10.00
Treatment Dose (uM)

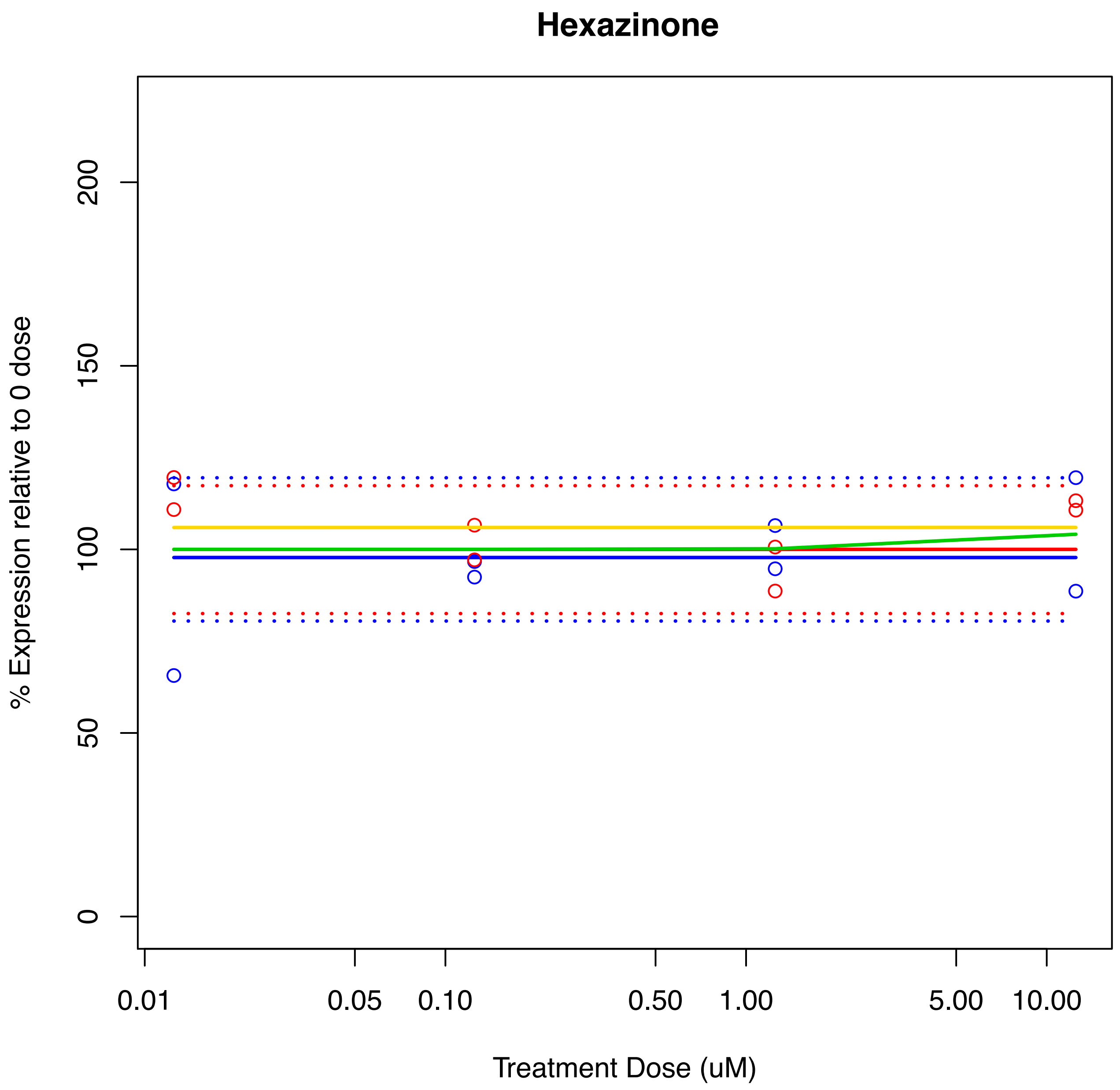
Hexazinone
% Expression relative to 0 dose
0
50
100
150
200
0.01
0.05
0.10
0.50
1.00
5.00
10.00
Treatment Dose (uM)

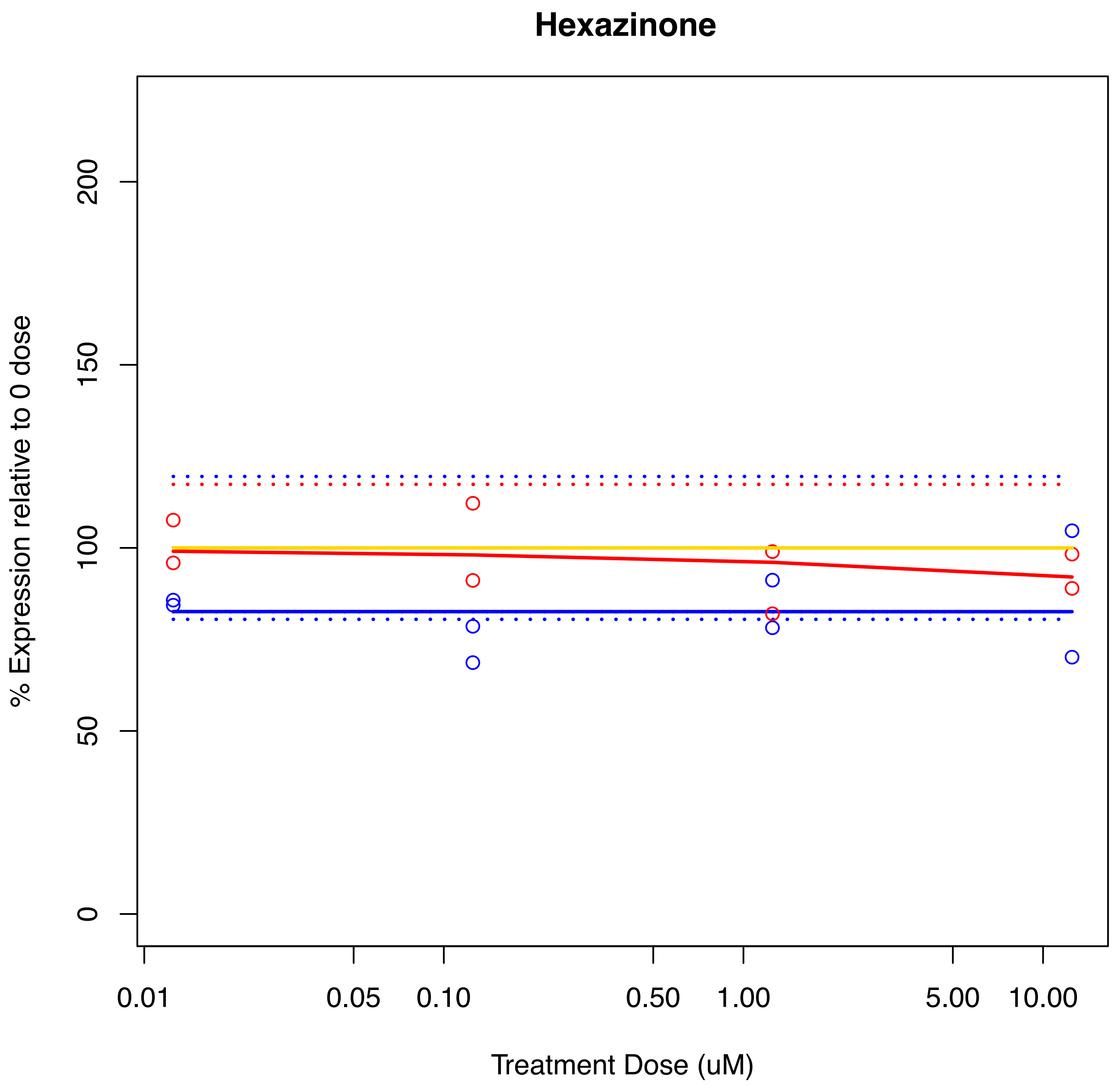
Hexazinone
% Expression relative to 0 dose
0
50
100
150
200
0.01
0.05
0.10
0.50
1.00
5.00
10.00
Treatment Dose (uM)

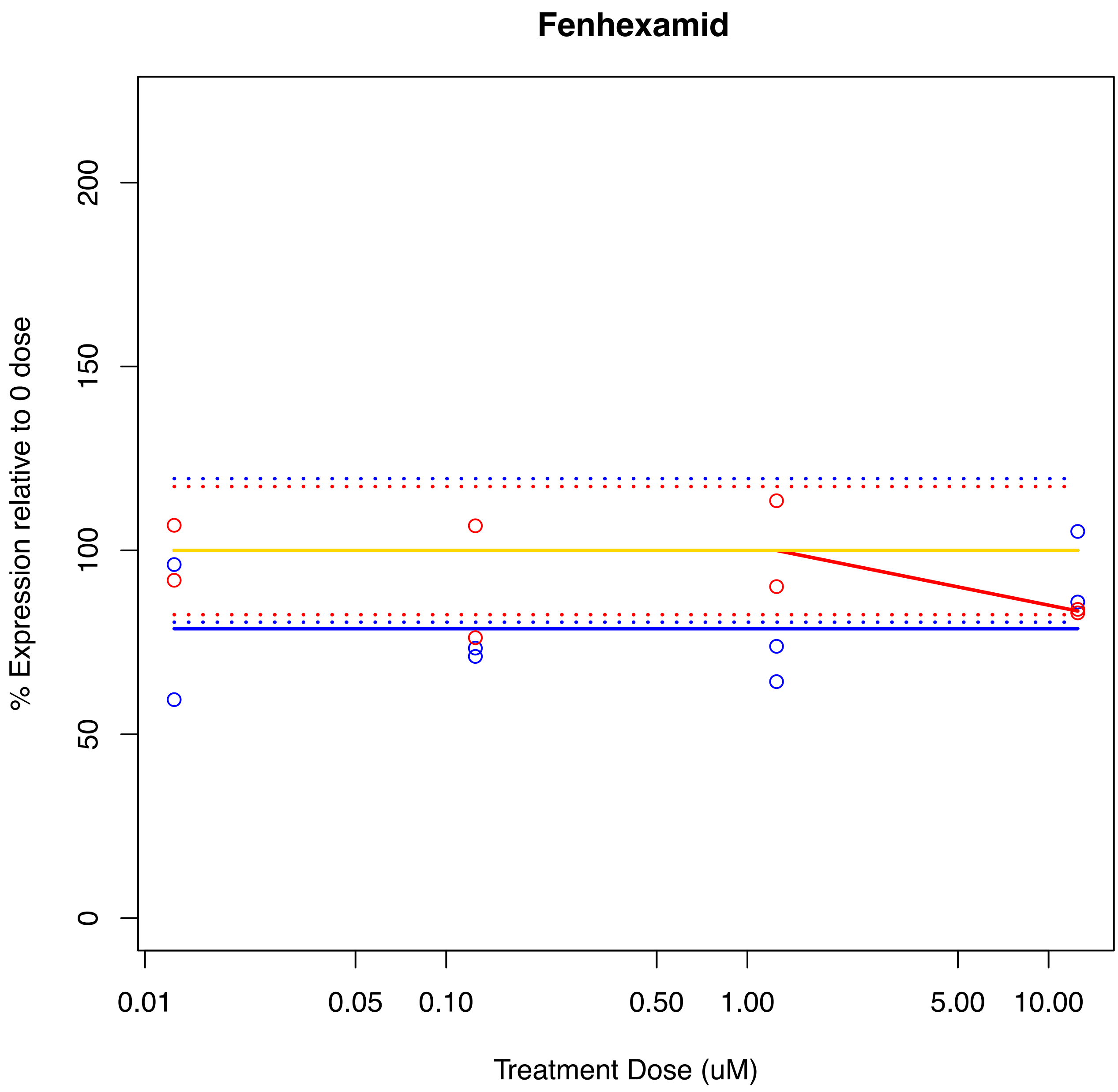

Fenhexamid
% Expression relative to 0 dose
0
50
100
150
200
0.01
0.05
0.10
0.50
1.00
5.00
10.00
Treatment Dose (uM)

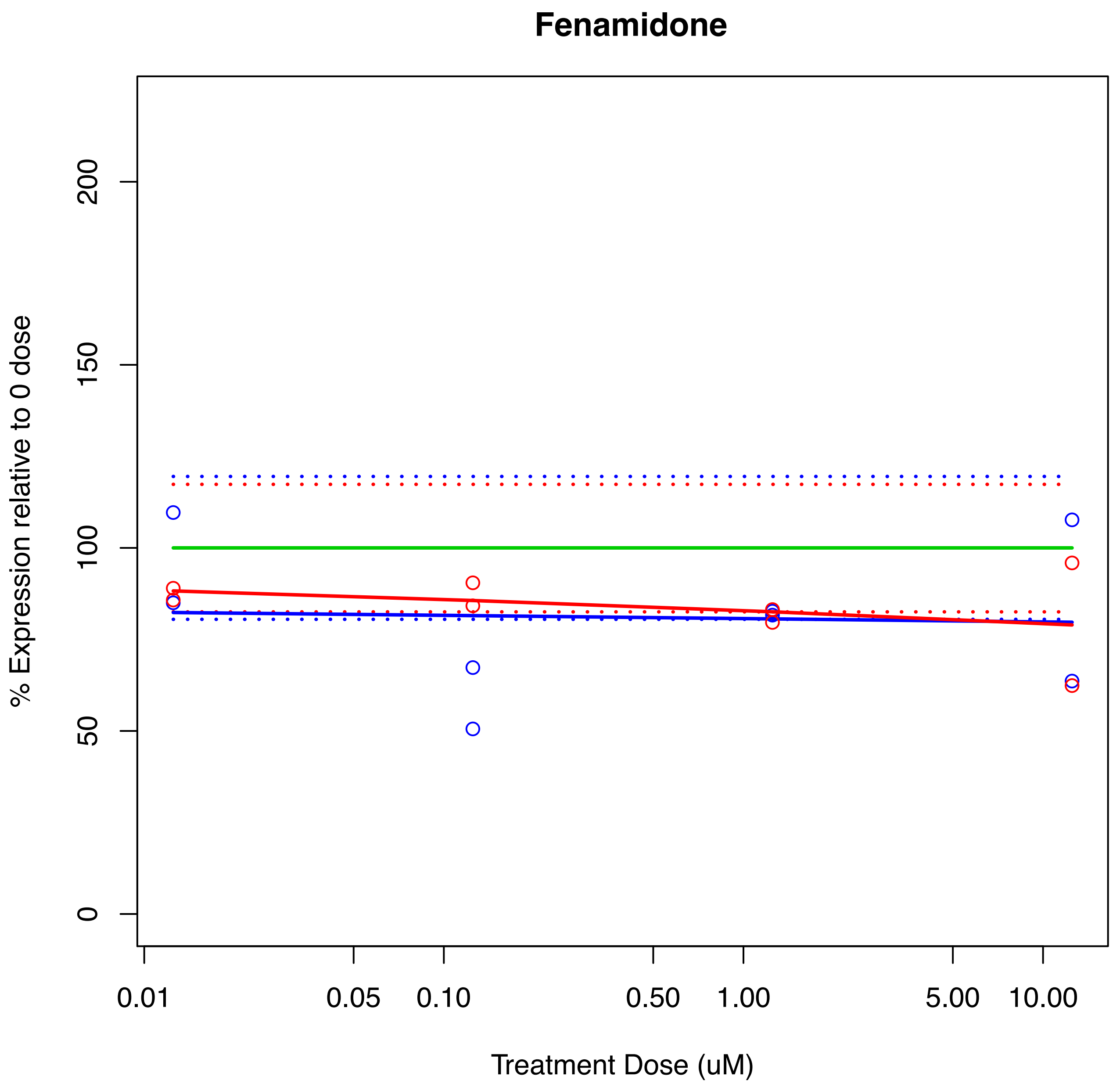
Fenamidone
% Expression relative to 0 dose
0
50
100
150
200
0.01
0.05
0.10
0.50
1.00
5.00
10.00
Treatment Dose (uM)

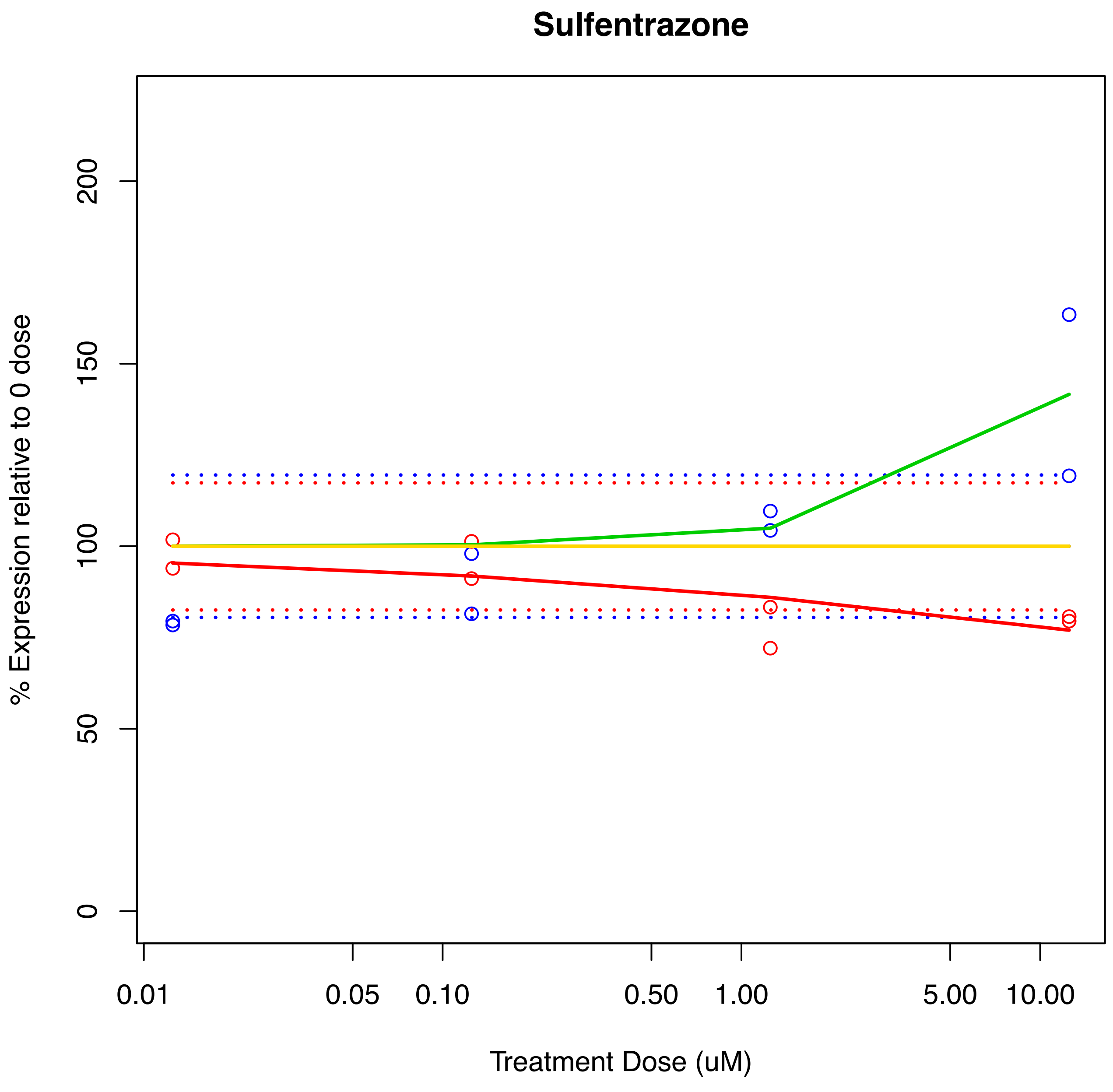

Sulfentrazone
% Expression relative to 0 dose
0
50
100
150
200
0.01
0.05
0.10
0.50
1.00
5.00
10.00
Treatment Dose (uM)

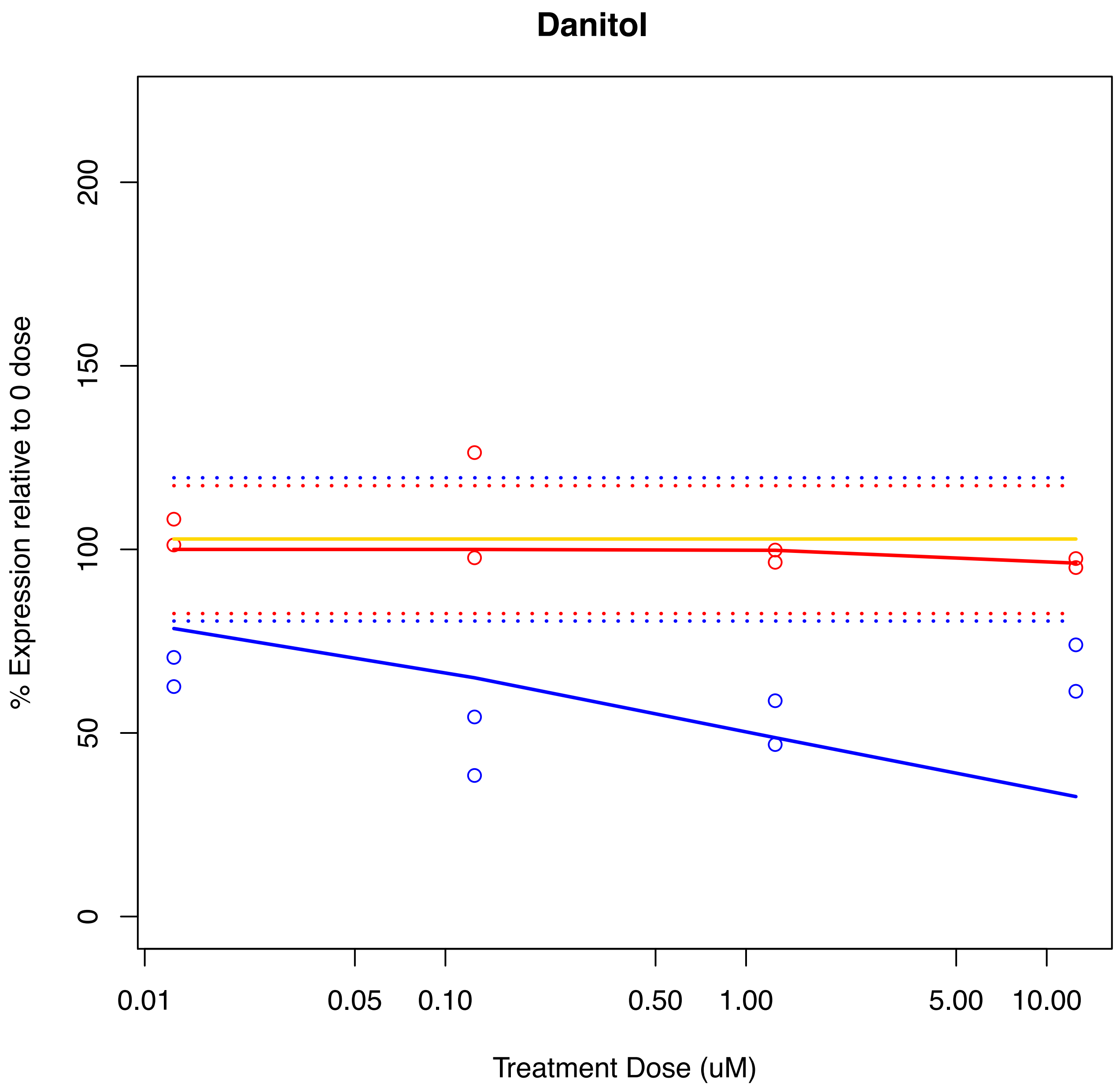

Danitol
% Expression relative to 0 dose
0
50
100
150
200
0.01
0.05
0.10
0.50
1.00
5.00
10.00
Treatment Dose (uM)

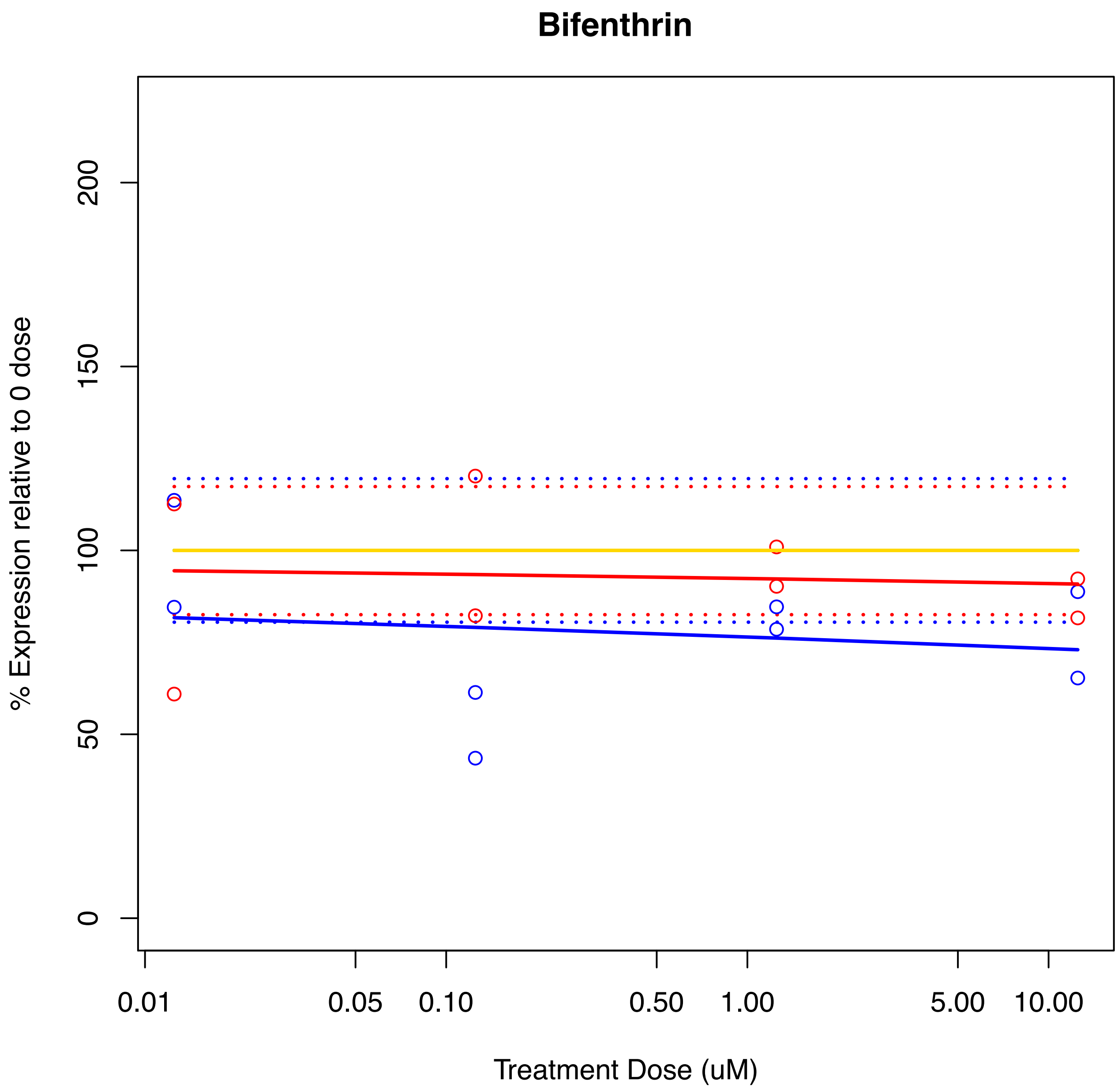

Bifenthrin
% Expression relative to 0 dose
0
50
100
150
200
0.01
0.05
0.10
0.50
1.00
5.00
10.00
Treatment Dose (uM)

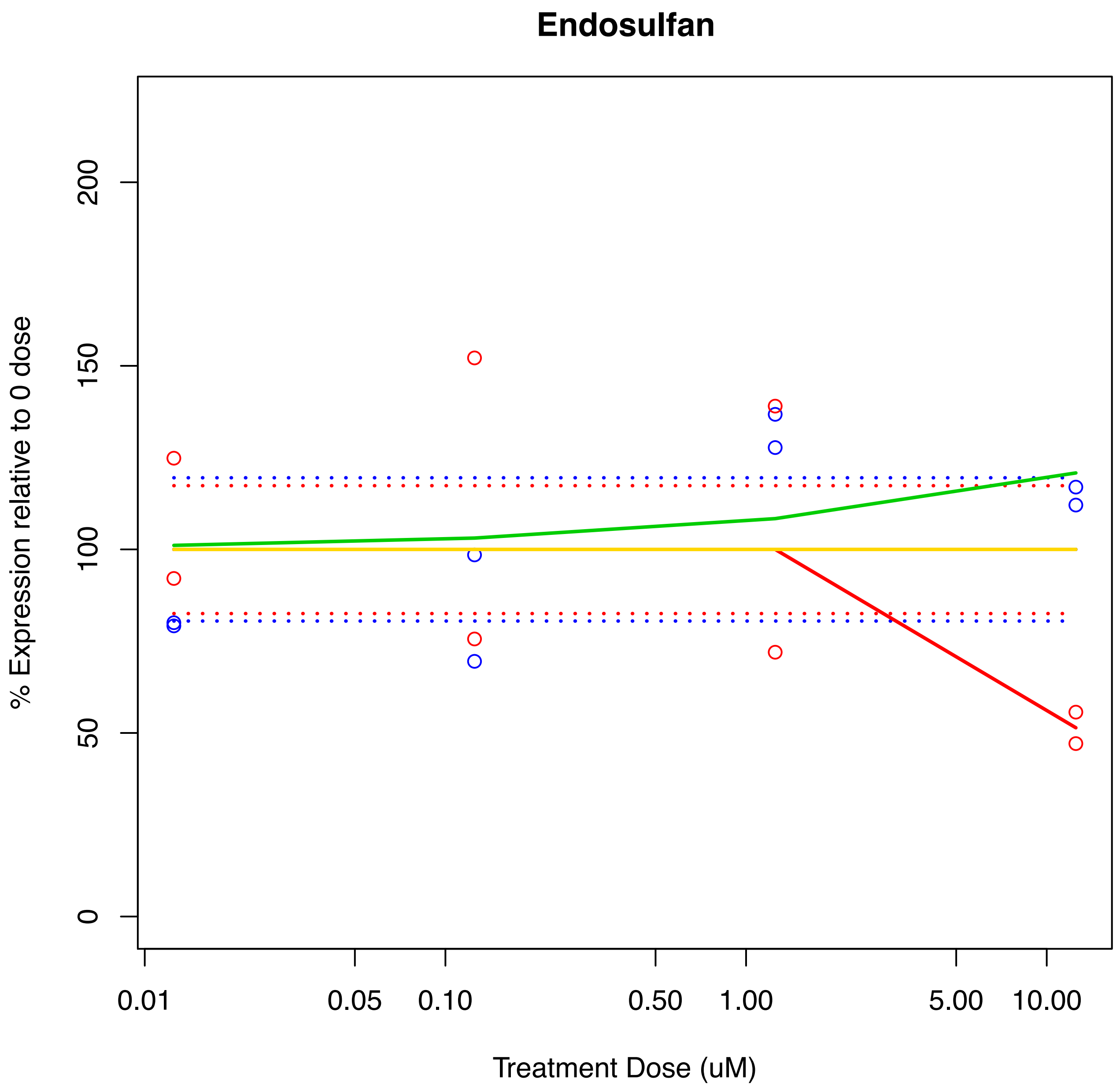
Endosulfan
% Expression relative to 0 dose
0
50
100
150
200
0.01
0.05
0.10
0.50
1.00
5.00
10.00
Treatment Dose (uM)

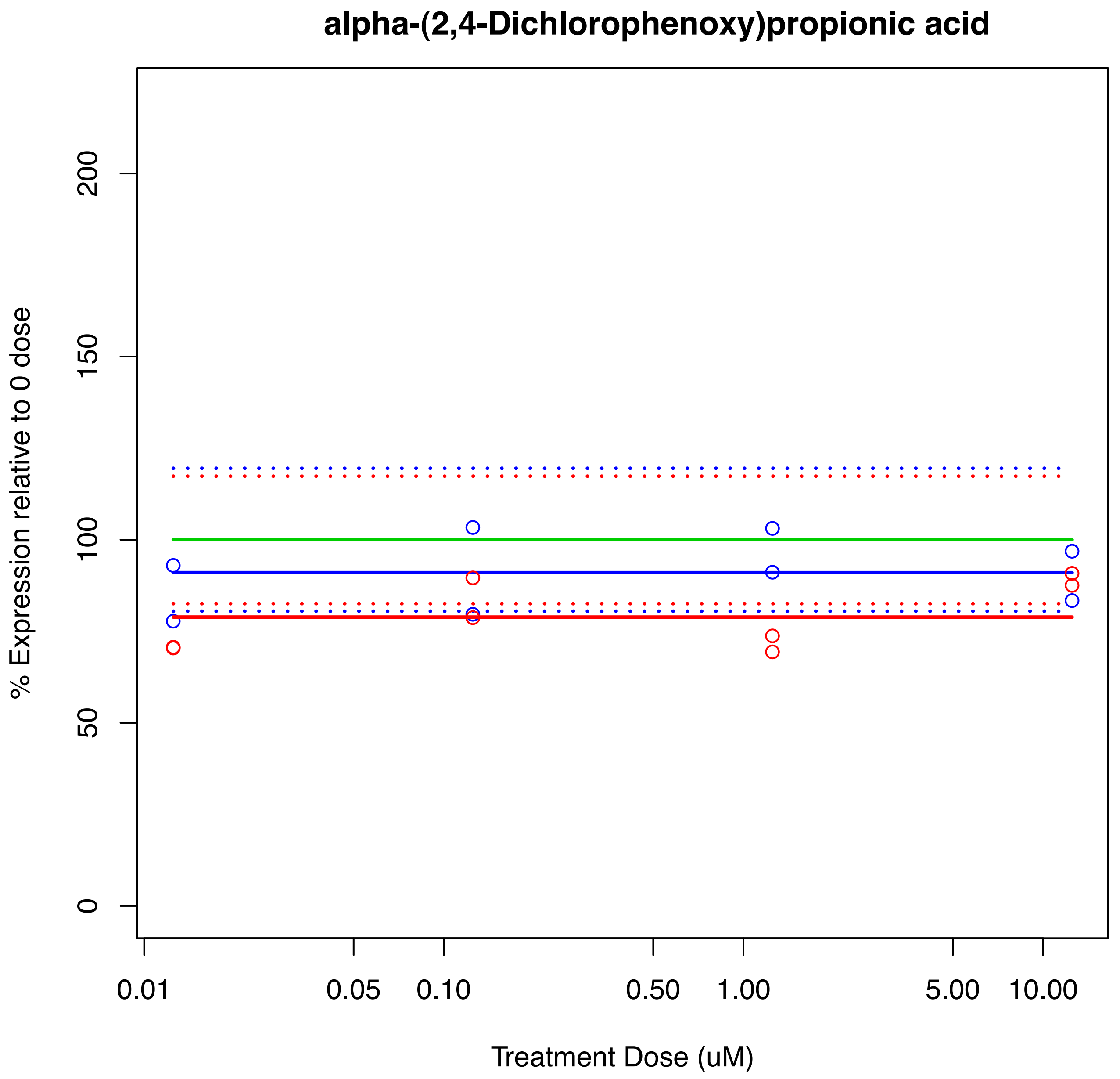
alpha-(2,4-Dichlorophenoxy)propionic acid
% Expression relative to 0 dose
0
50
100
150
200
0.01
0.05
0.10
0.50
1.00
5.00
10.00
Treatment Dose (uM)

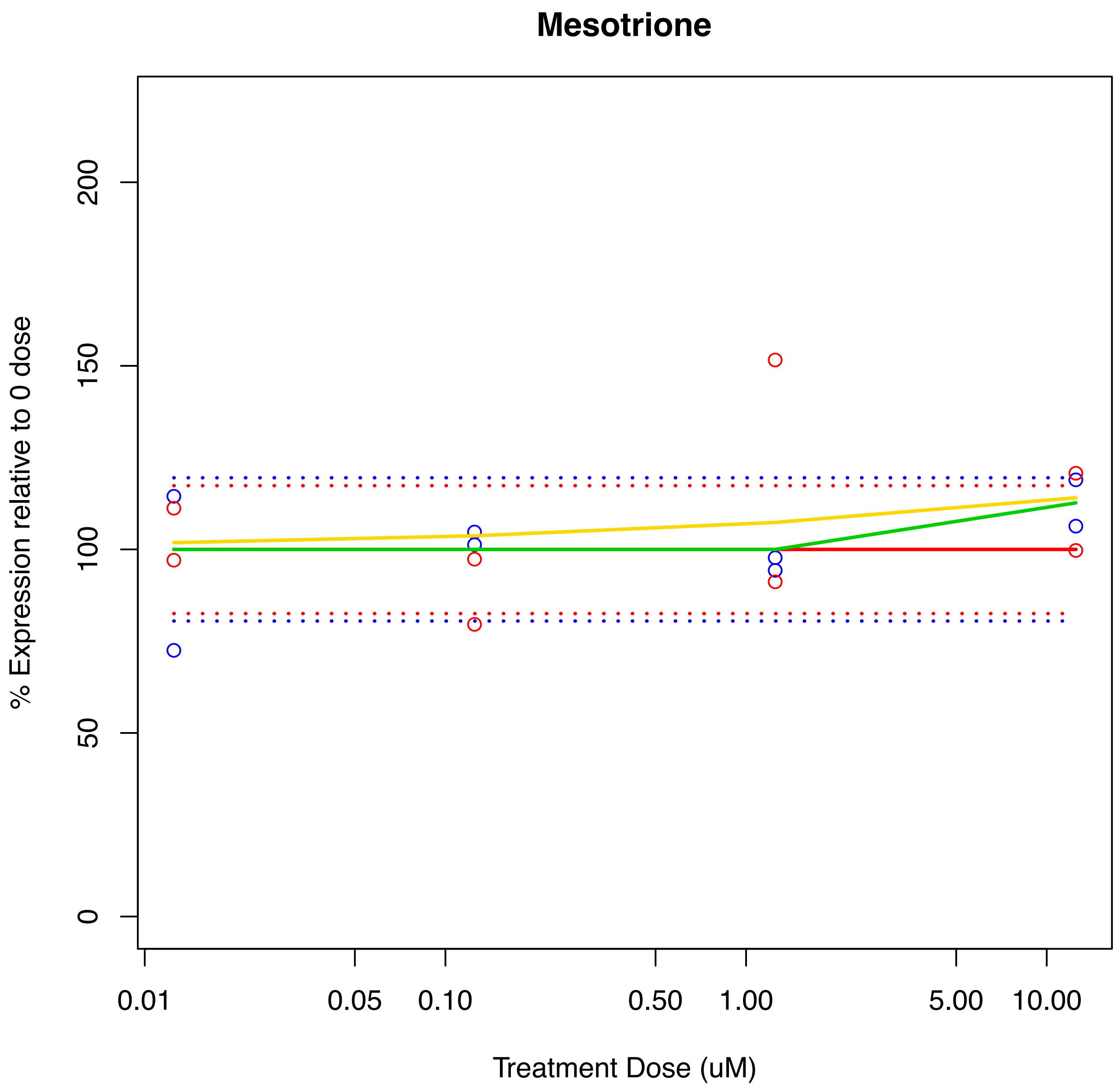

Mesotrione
% Expression relative to 0 dose
0
50
100
150
200
0.01
0.05
0.10
0.50
1.00
5.00
10.00
Treatment Dose (uM)

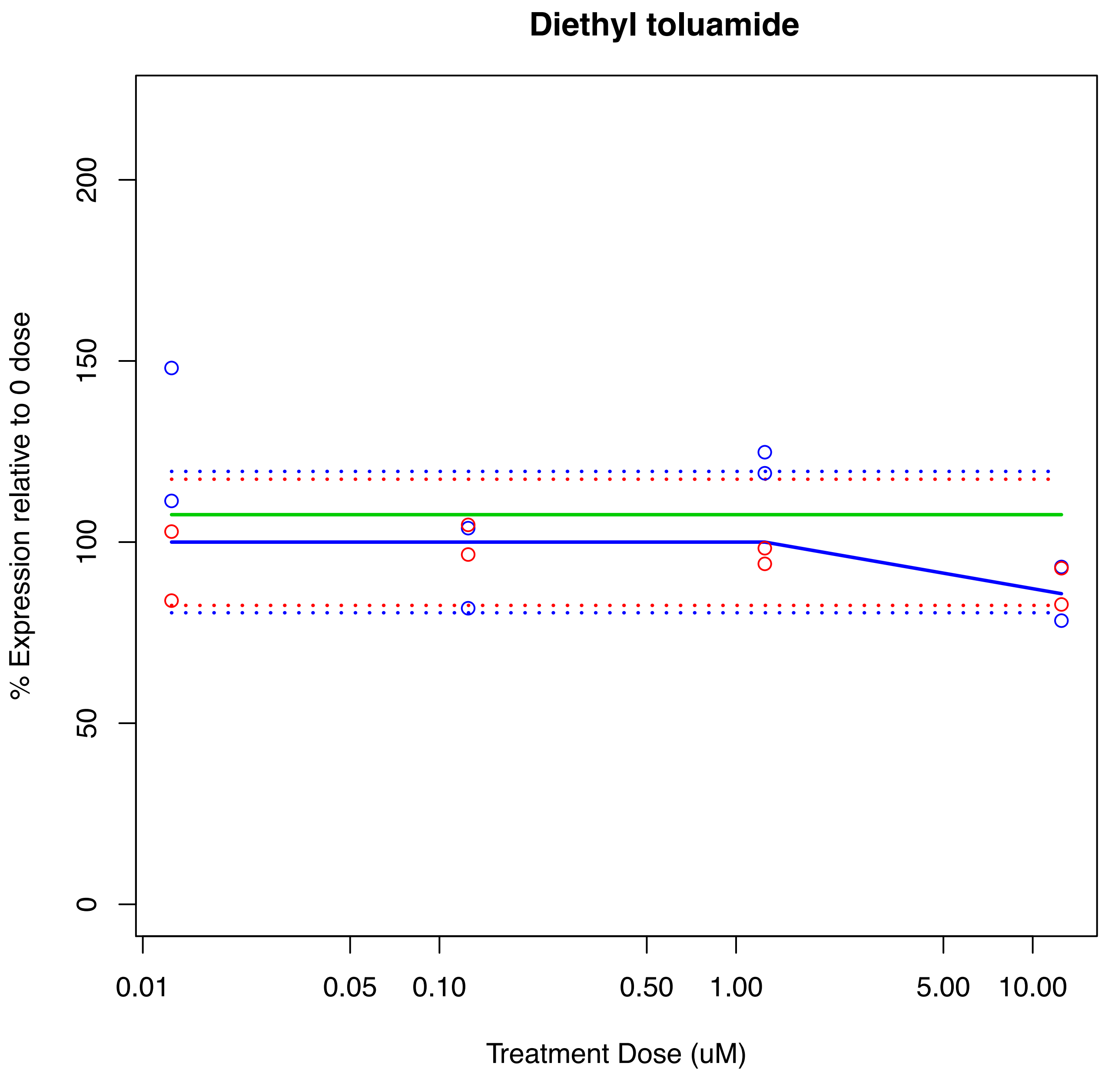

Diethyl toluamide
% Expression relative to 0 dose
0
50
100
150
200
0.01
0.05
0.10
0.50
1.00
5.00
10.00
Treatment Dose (uM)

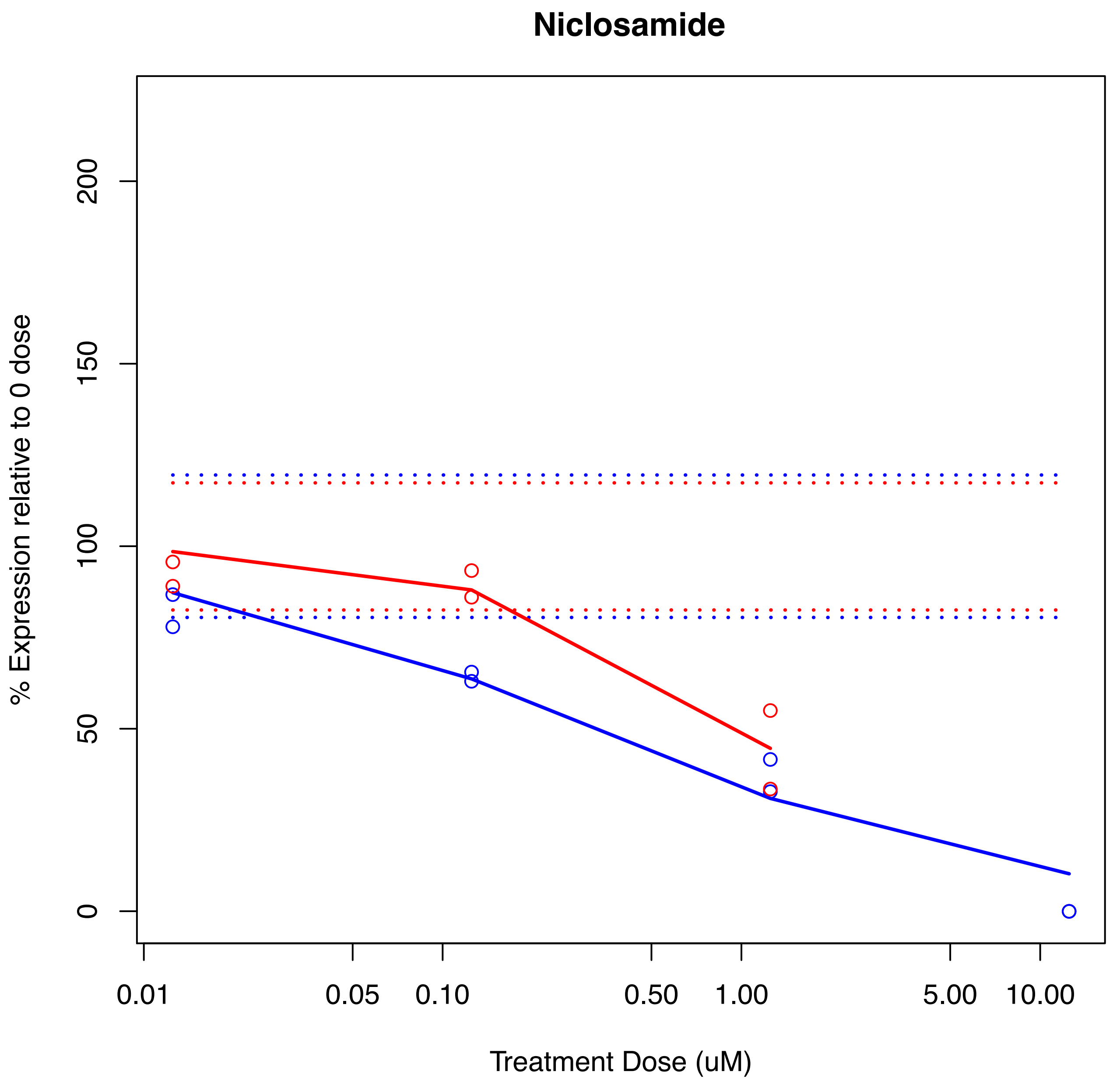

Niclosamide
% Expression relative to 0 dose
0
50
100
150
200
0.01
0.05
0.10
0.50
1.00
5.00
10.00
Treatment Dose (uM)

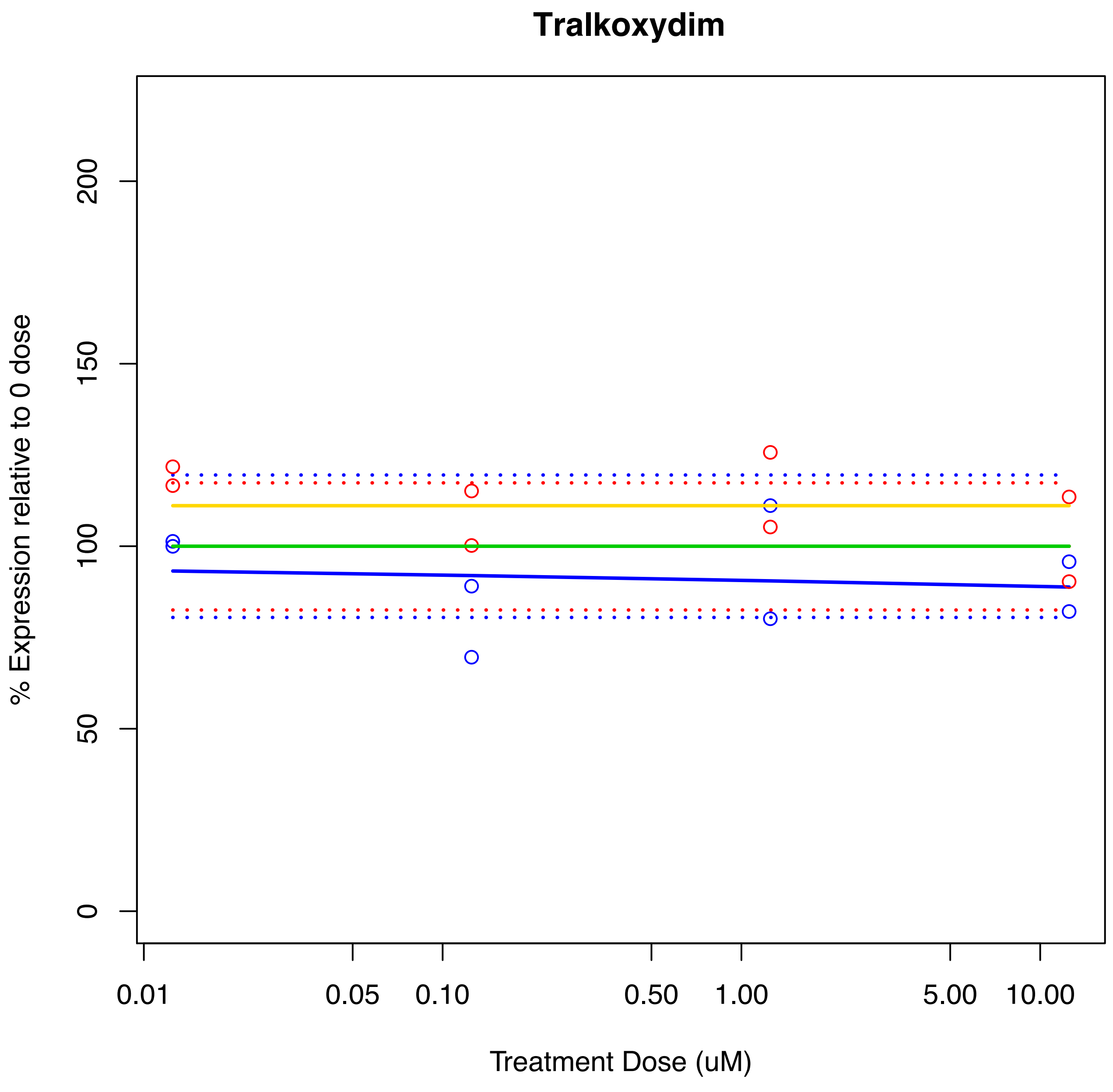

Tralkoxydim
% Expression relative to 0 dose
0
50
100
150
200
0.01
0.05
0.10
0.50
1.00
5.00
10.00
Treatment Dose (uM)

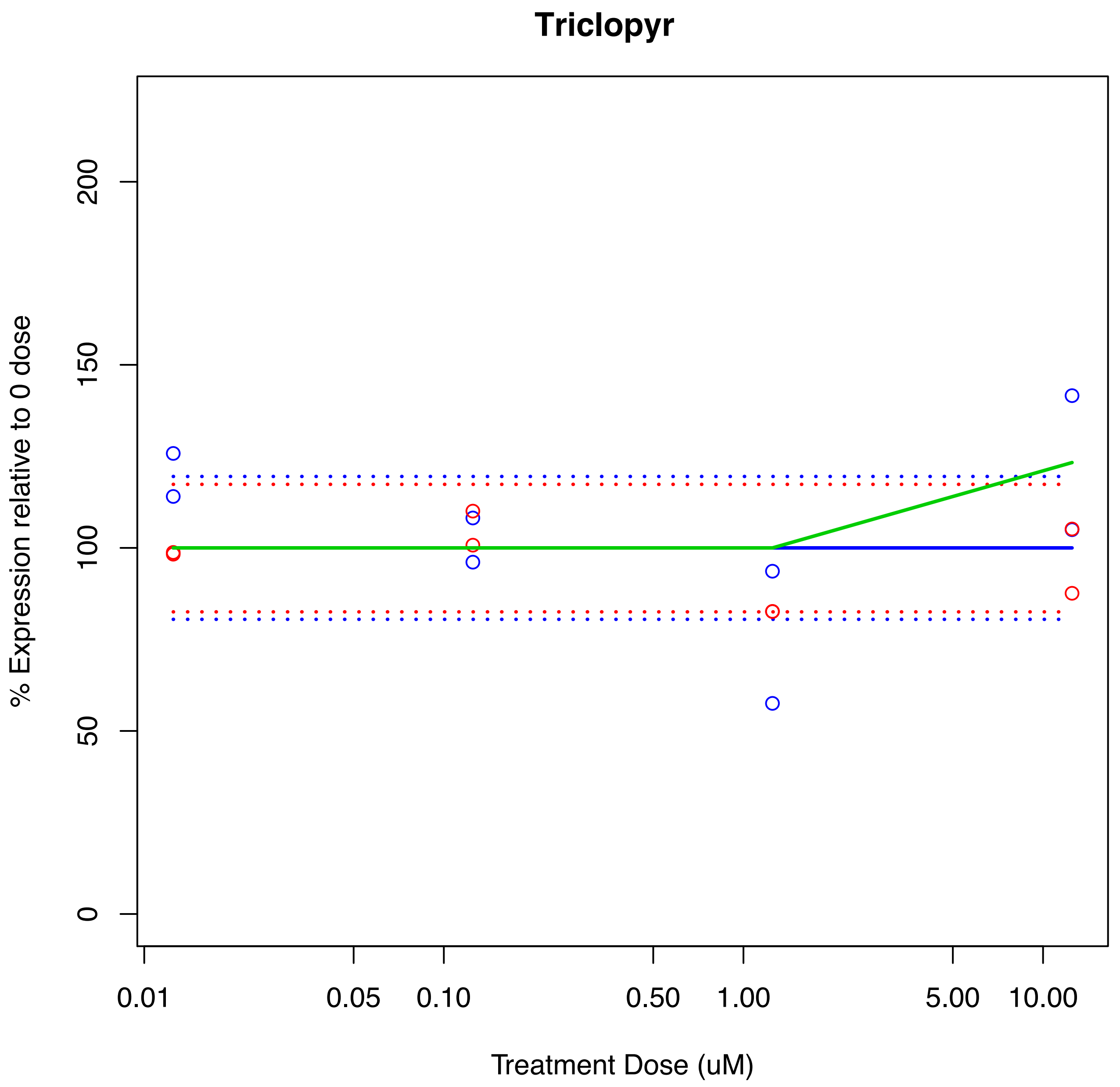
Triclopyr
% Expression relative to 0 dose
0
50
100
150
200
0.01
0.05
0.10
0.50
1.00
5.00
10.00
Treatment Dose (uM)

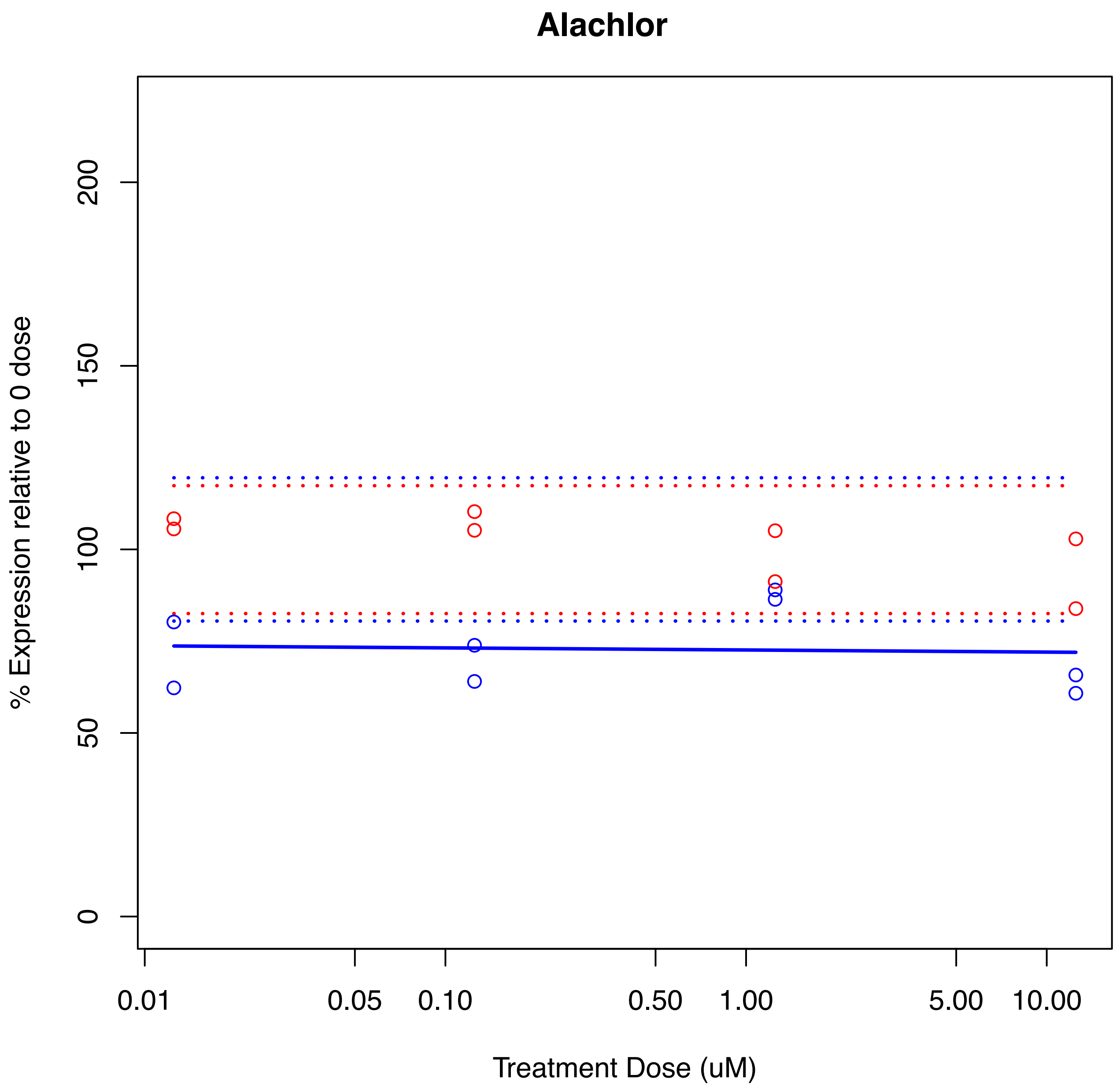
Alachlor
% Expression relative to 0 dose
0
50
100
150
200
0.01
0.05
0.10
0.50
1.00
5.00
10.00
Treatment Dose (uM)

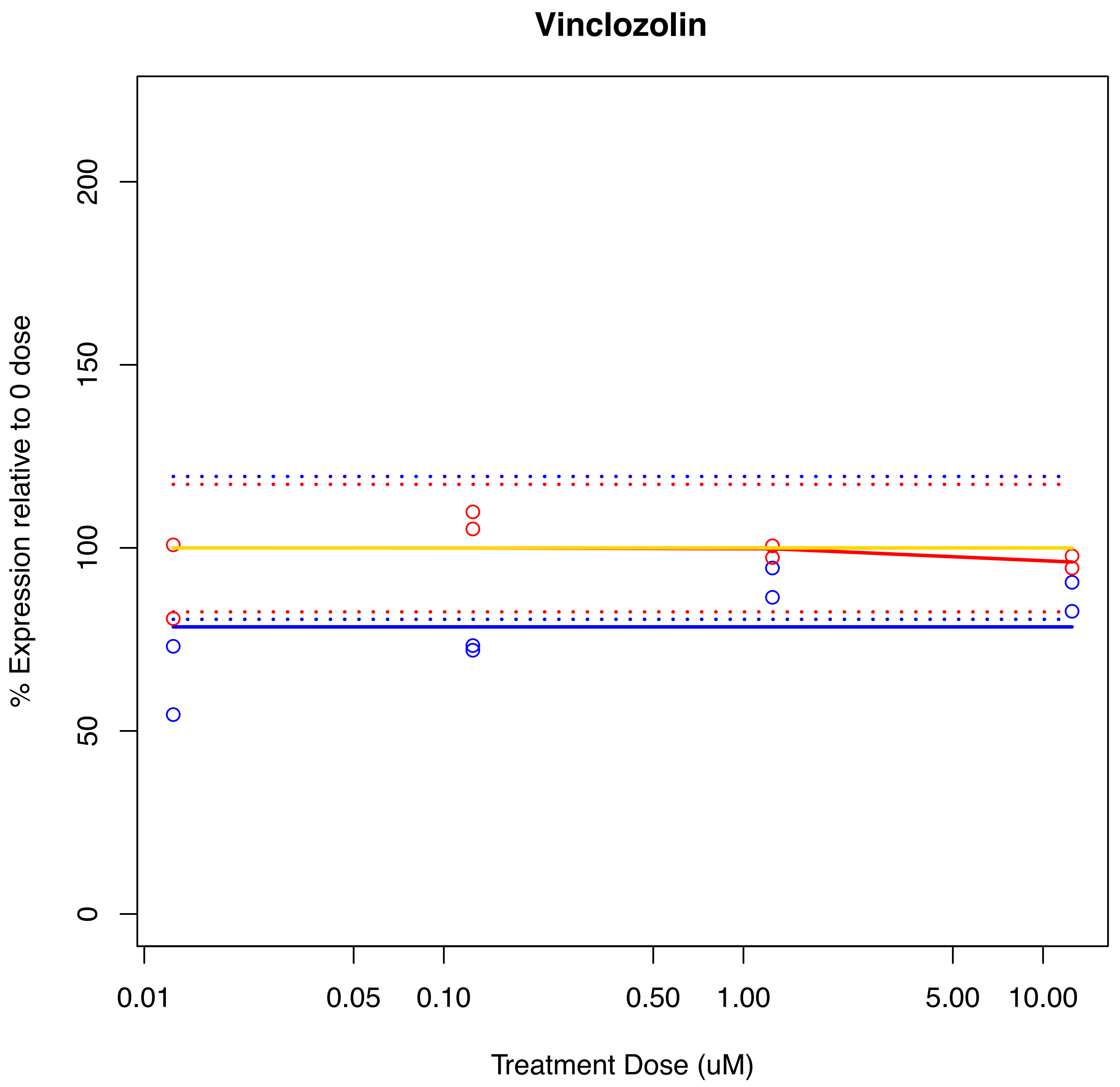

Vinclozolin
% Expression relative to 0 dose
0
50
100
150
200
0.01
0.05
0.10
0.50
1.00
5.00
10.00
Treatment Dose (uM)

Bensulide

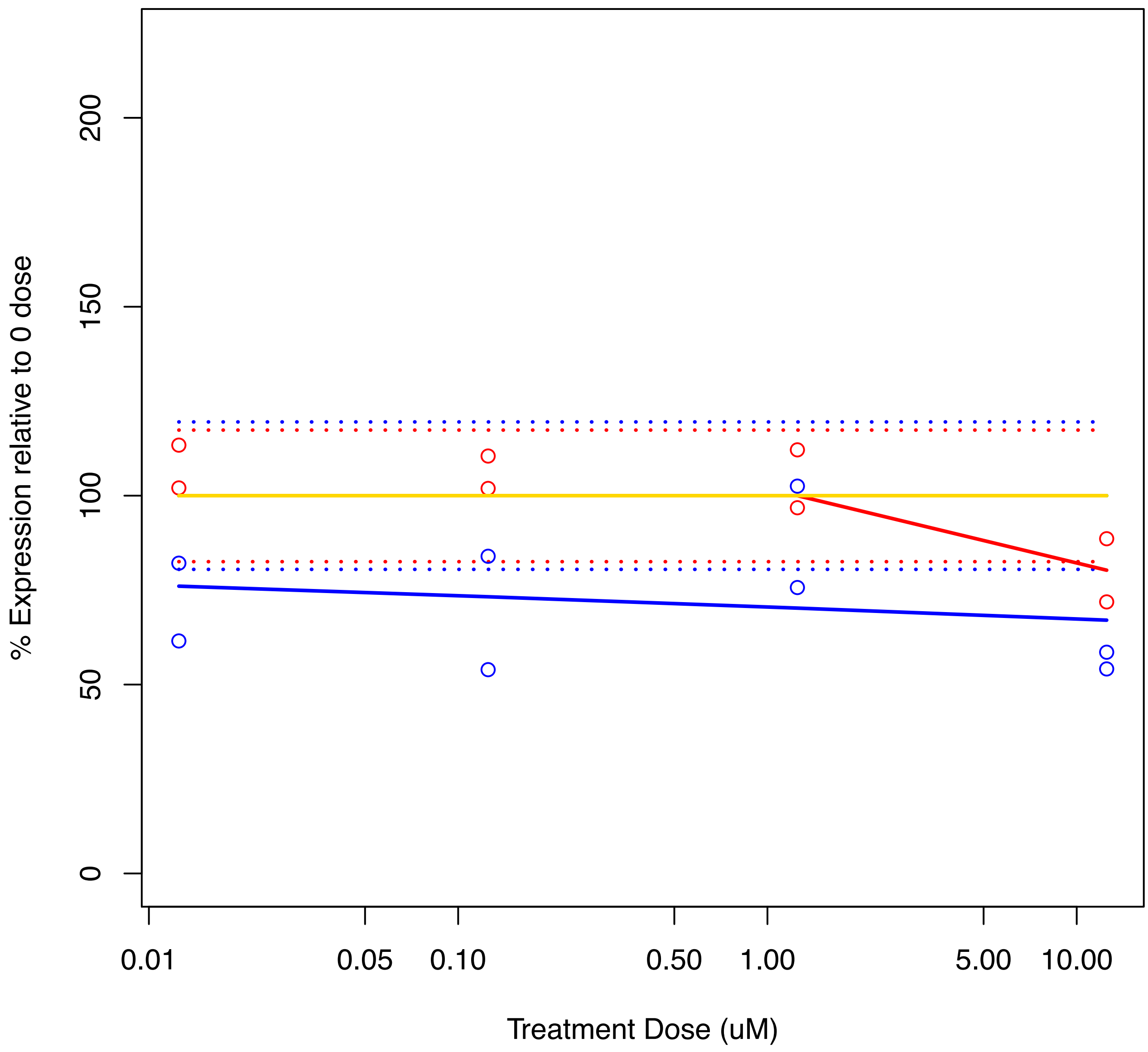

% Expression relative to 0 dose
0
50
100
150
200
0.01
0.05
0.10
0.50
1.00
5.00
10.00
Treatment Dose (uM)

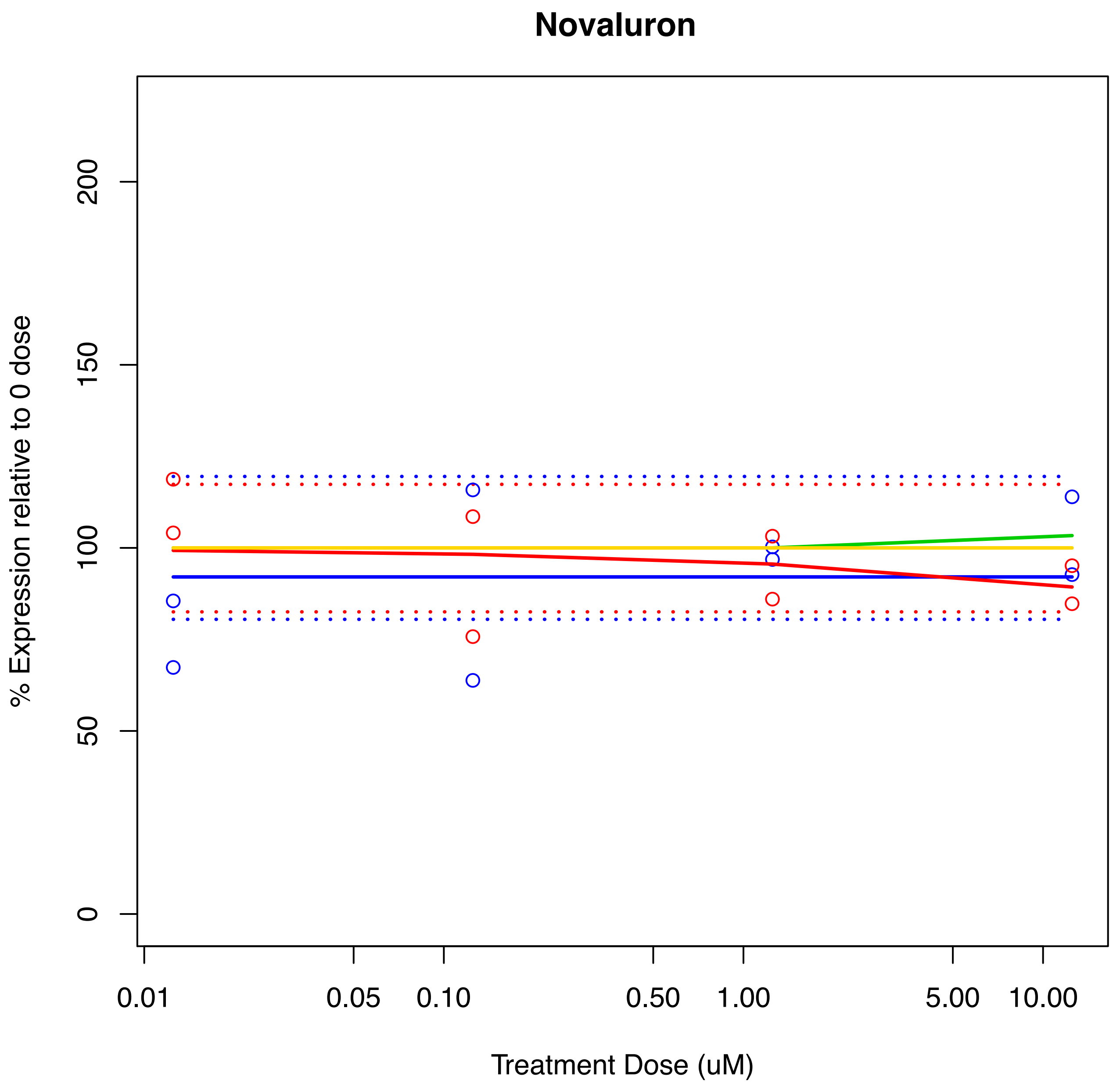

Novaluron
% Expression relative to 0 dose
0
50
100
150
200
0.01
0.05
0.10
0.50
1.00
5.00
10.00
Treatment Dose (uM)

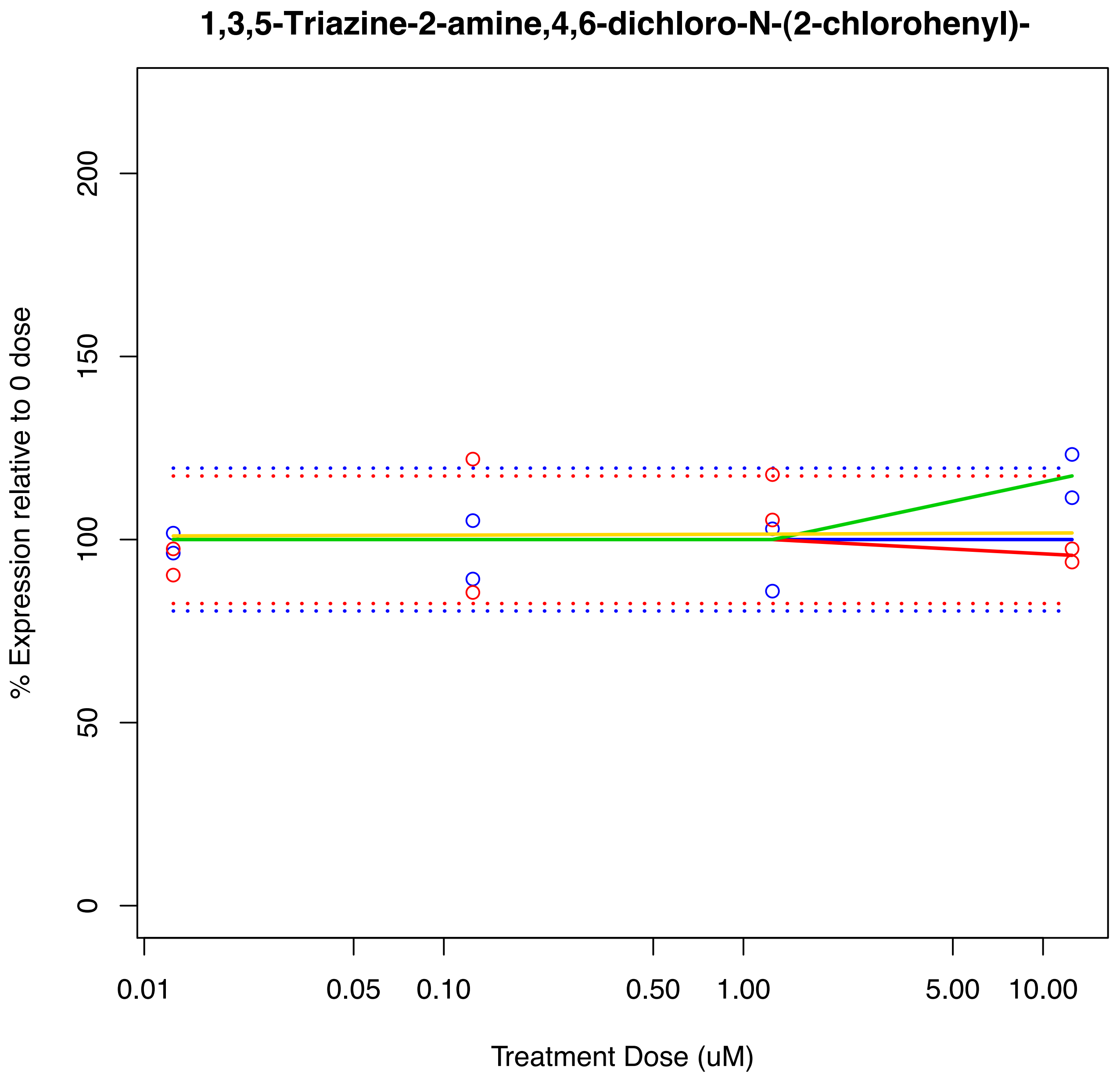

1,3,5-Triazine-2-amine,4,6-dichloro-N-(2-chlorohenyl)-
% Expression relative to 0 dose
0
50
100
150
200
0.01
0.05
0.10
0.50
1.00
5.00
10.00
Treatment Dose (uM)

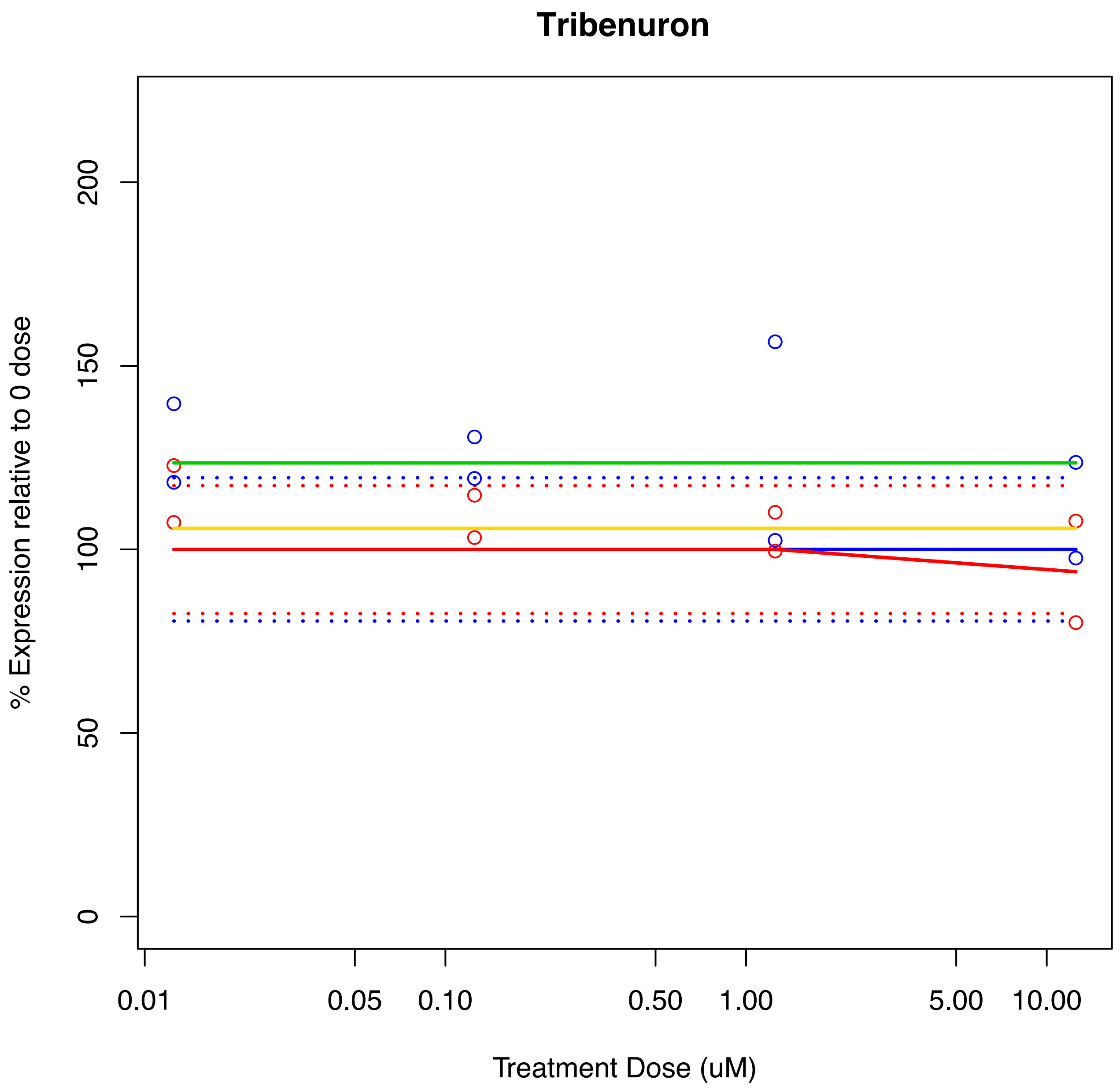
Tribenuron
% Expression relative to 0 dose
0
50
100
150
200
0.01
0.05
0.10
0.50
1.00
5.00
10.00
Treatment Dose (uM)

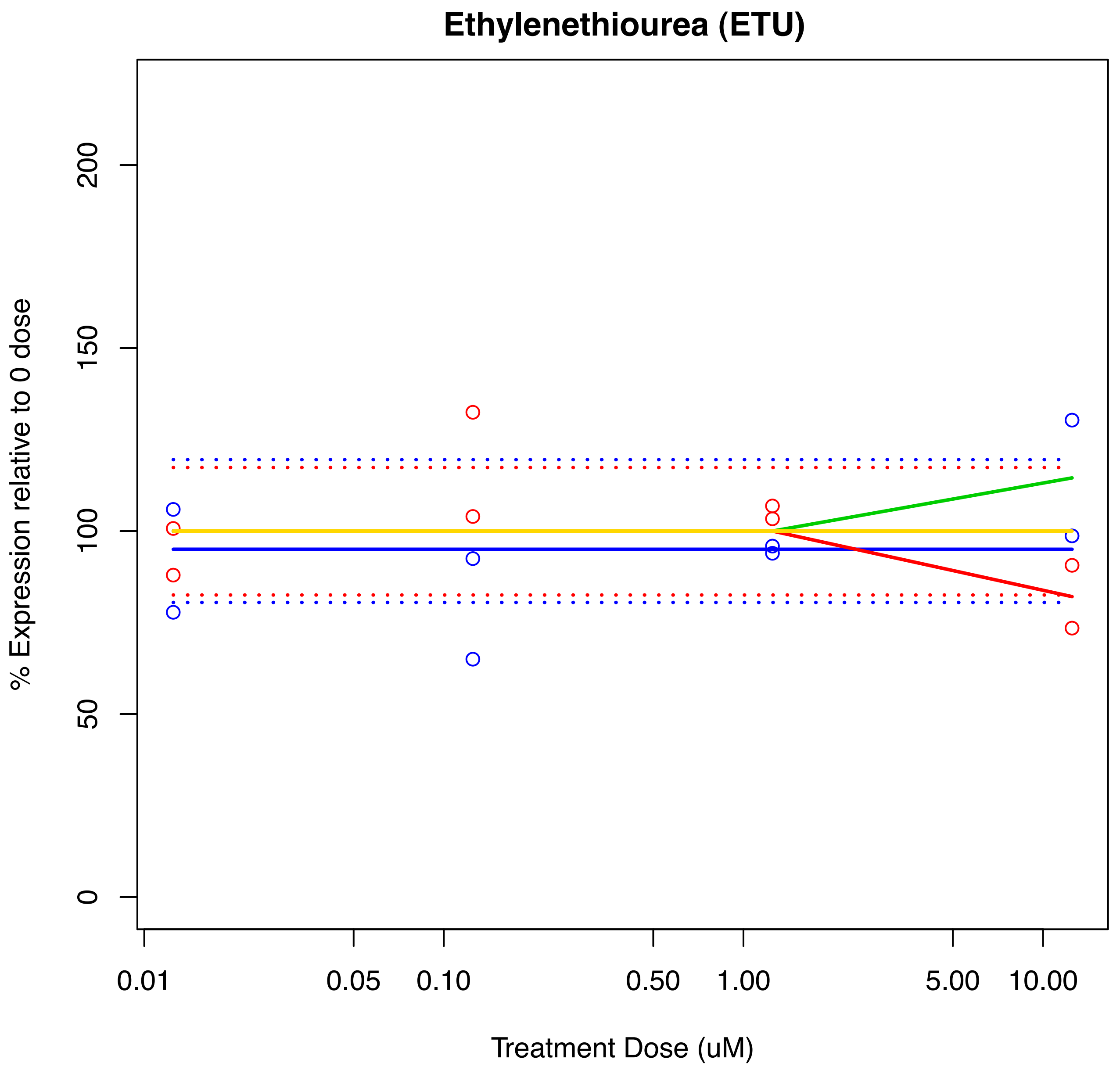

Ethylenethiourea (ETU)
% Expression relative to 0 dose
0
50
100
150
200
0.01
0.05
0.10
0.50
1.00
5.00
10.00
Treatment Dose (uM)

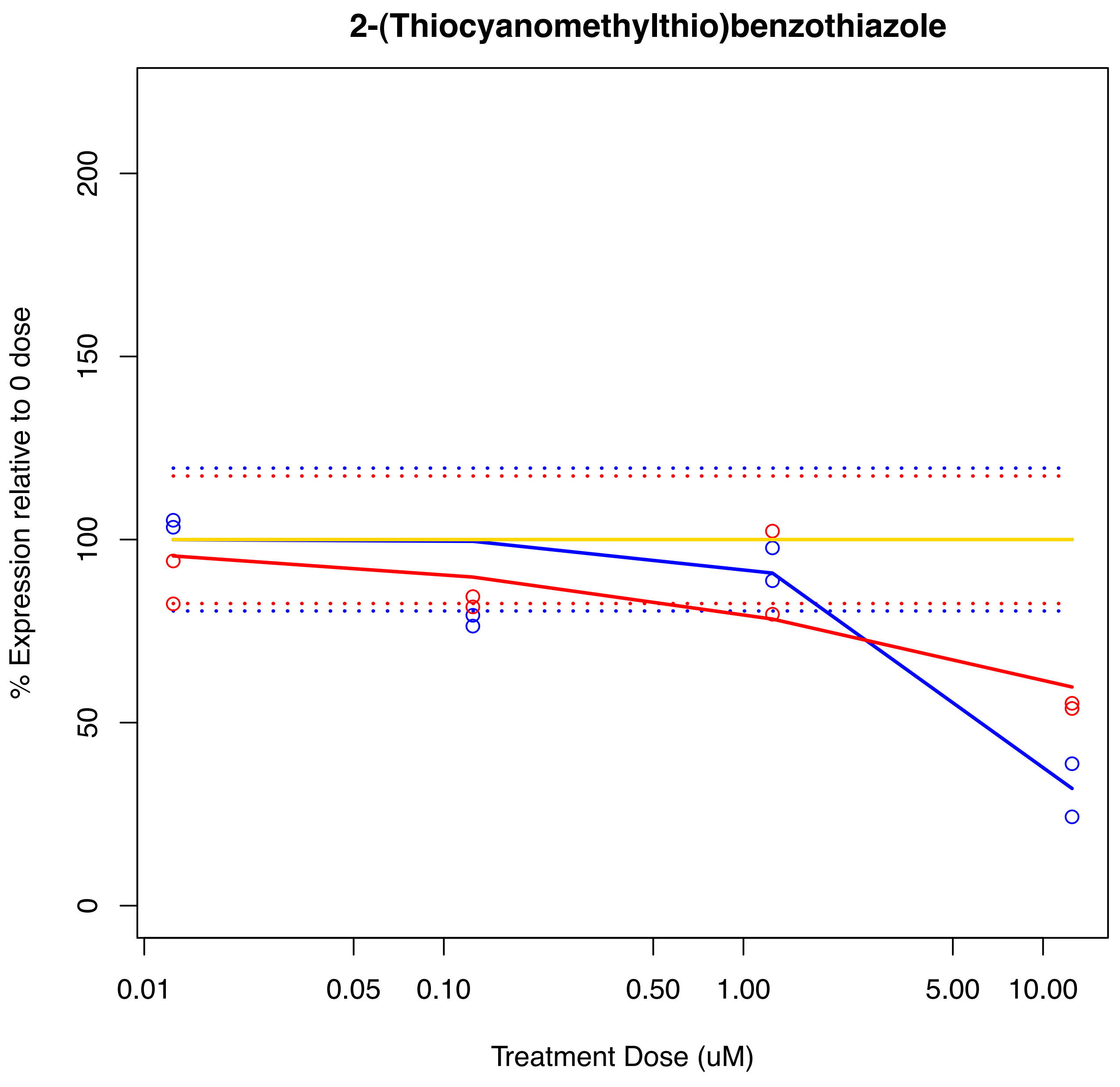
2-(Thiocyanomethylthio)benzothiazole
% Expression relative to 0 dose
0
50
100
150
200
0.01
0.05
0.10
0.50
1.00
5.00
10.00
Treatment Dose (uM)

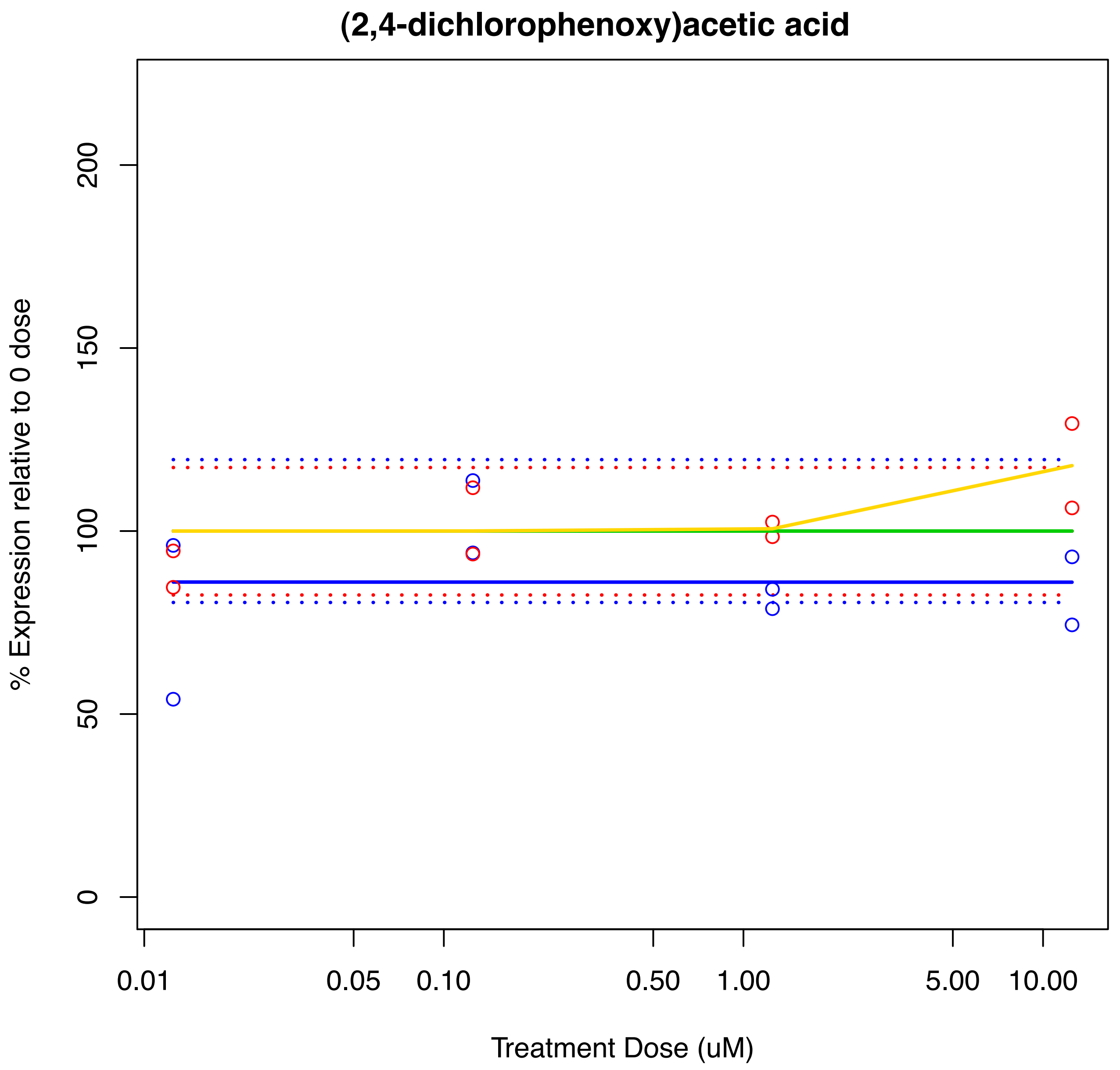

(2,4-dichlorophenoxy)acetic acid
% Expression relative to 0 dose
0
50
100
150
200
0.01
0.05
0.10
0.50
1.00
5.00
10.00
Treatment Dose (uM)

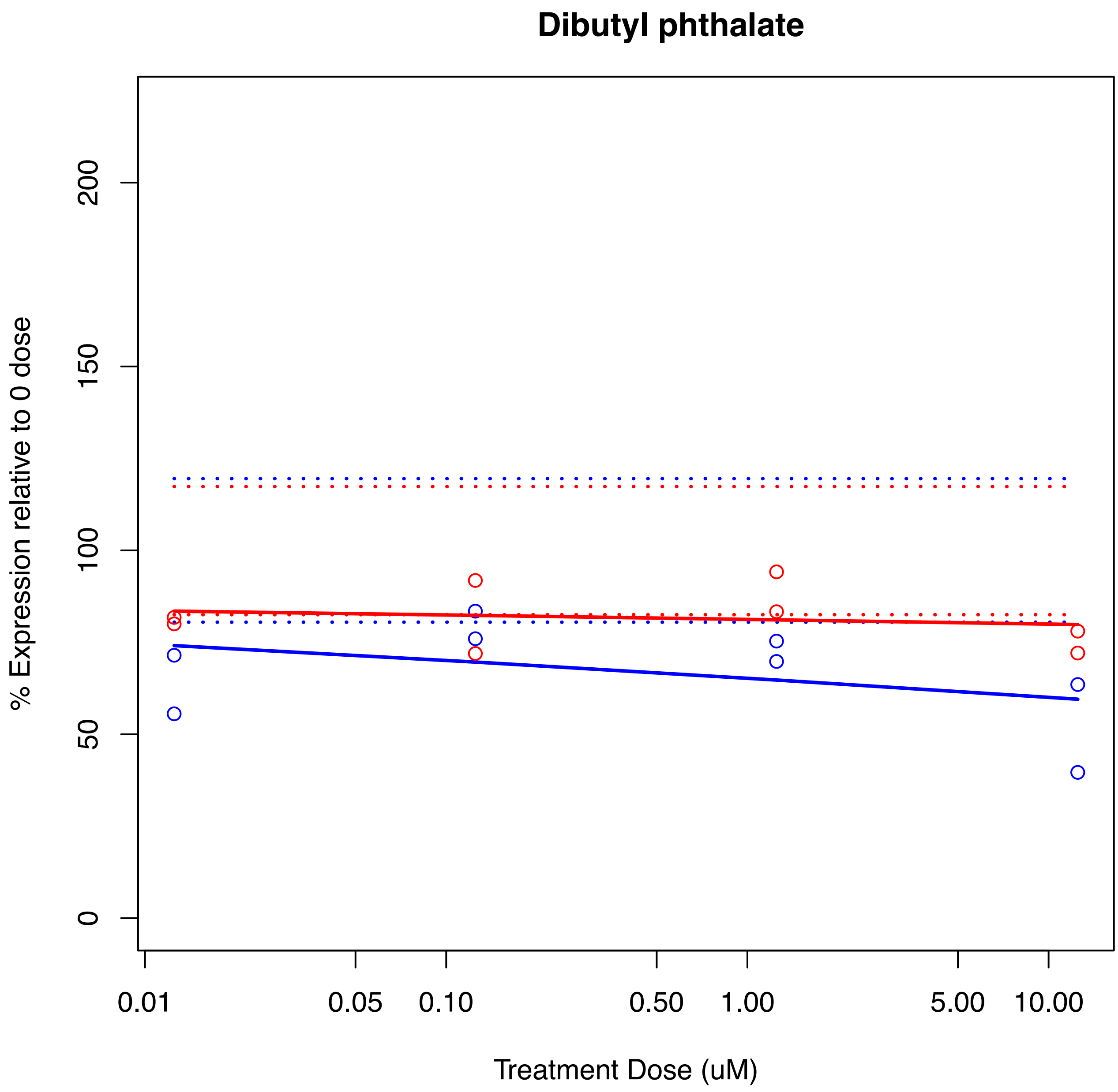

Dibutyl phthalate
% Expression relative to 0 dose
0
50
100
150
200
0.01
0.05
0.10
0.50
1.00
5.00
10.00
Treatment Dose (uM)

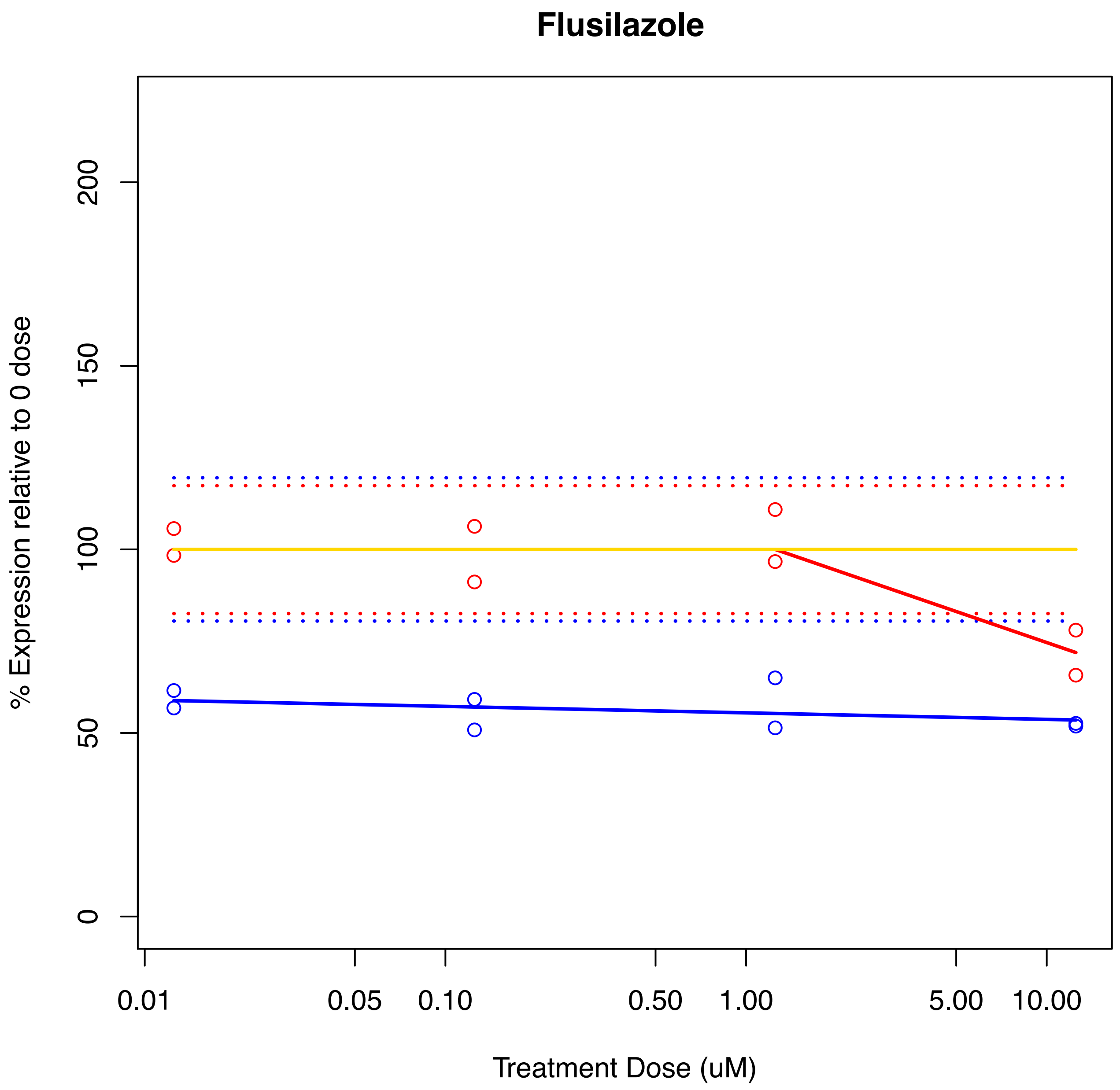

Flusilazole
% Expression relative to 0 dose
0
50
100
150
200
0.01
0.05
0.10
0.50
1.00
5.00
10.00
Treatment Dose (uM)

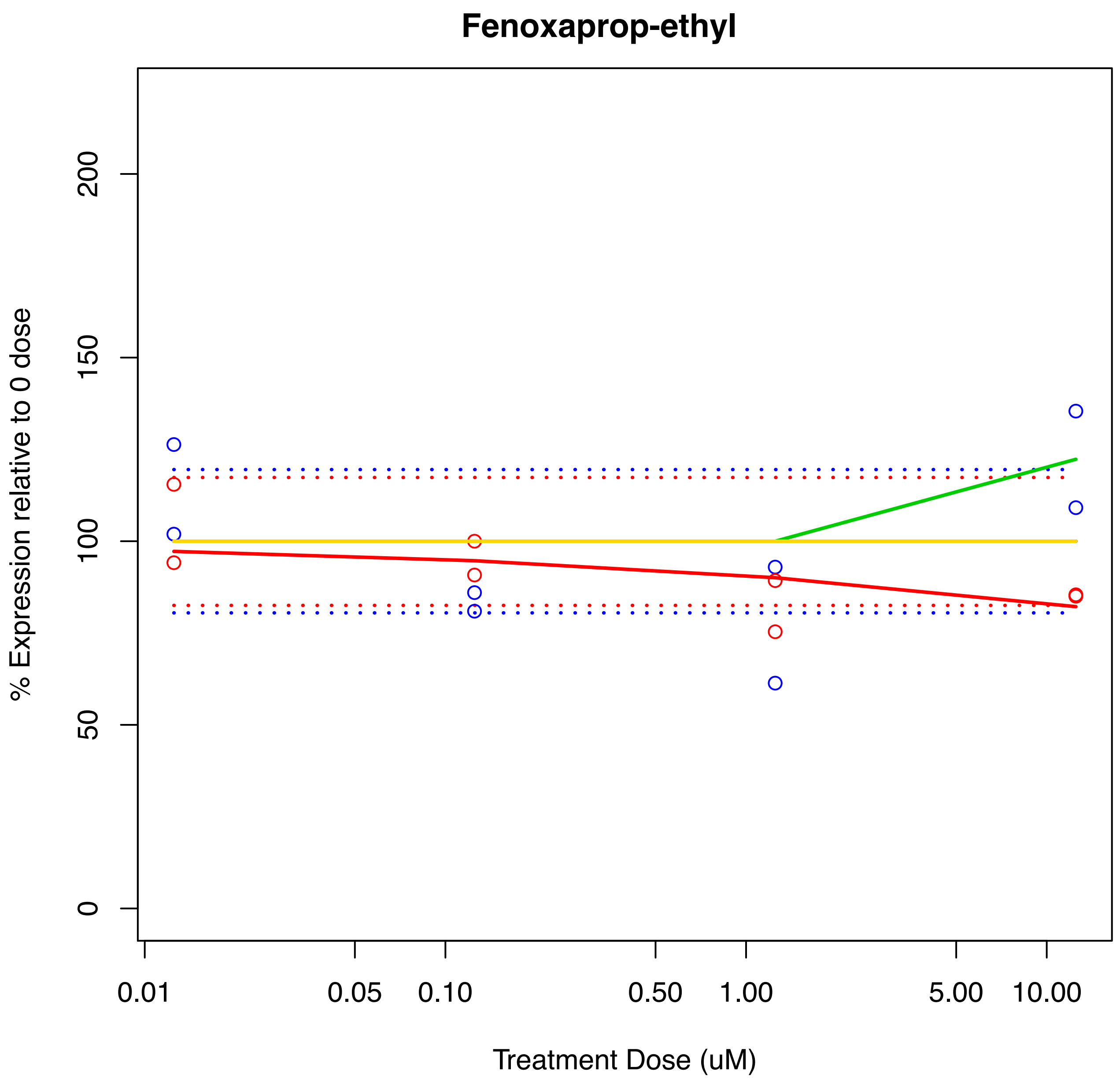
Fenoxaprop-ethyl
% Expression relative to 0 dose
0
50
100
150
200
0.01
0.05
0.10
0.50
1.00
5.00
10.00
Treatment Dose (uM)

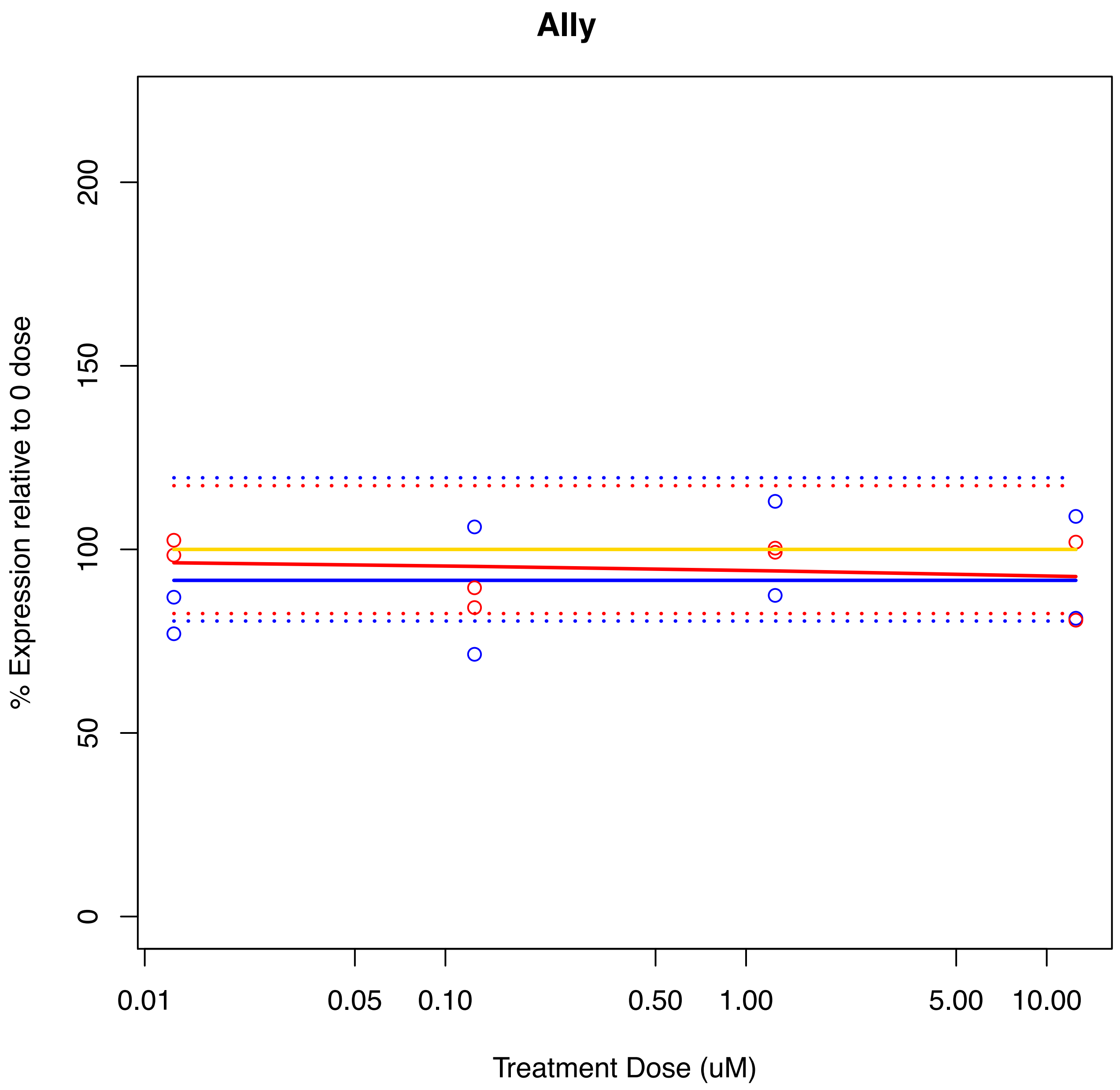

Ally
% Expression relative to 0 dose
0
50
100
150
200
0.01
0.05
0.10
0.50
1.00
5.00
10.00
Treatment Dose (uM)

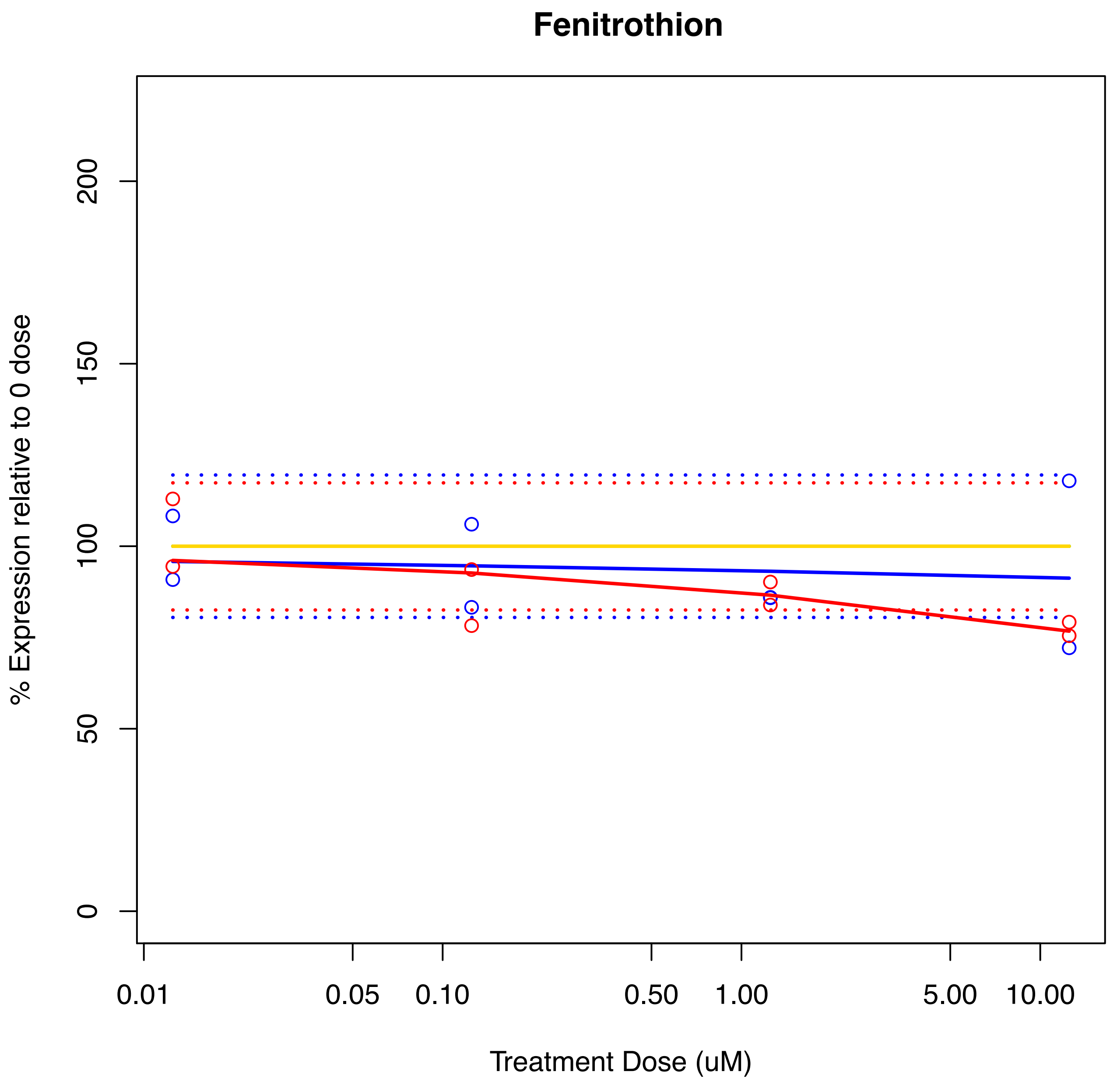

Fenitrothion
% Expression relative to 0 dose
0
50
100
150
200
0.01
0.05
0.10
0.50
1.00
5.00
10.00
Treatment Dose (uM)

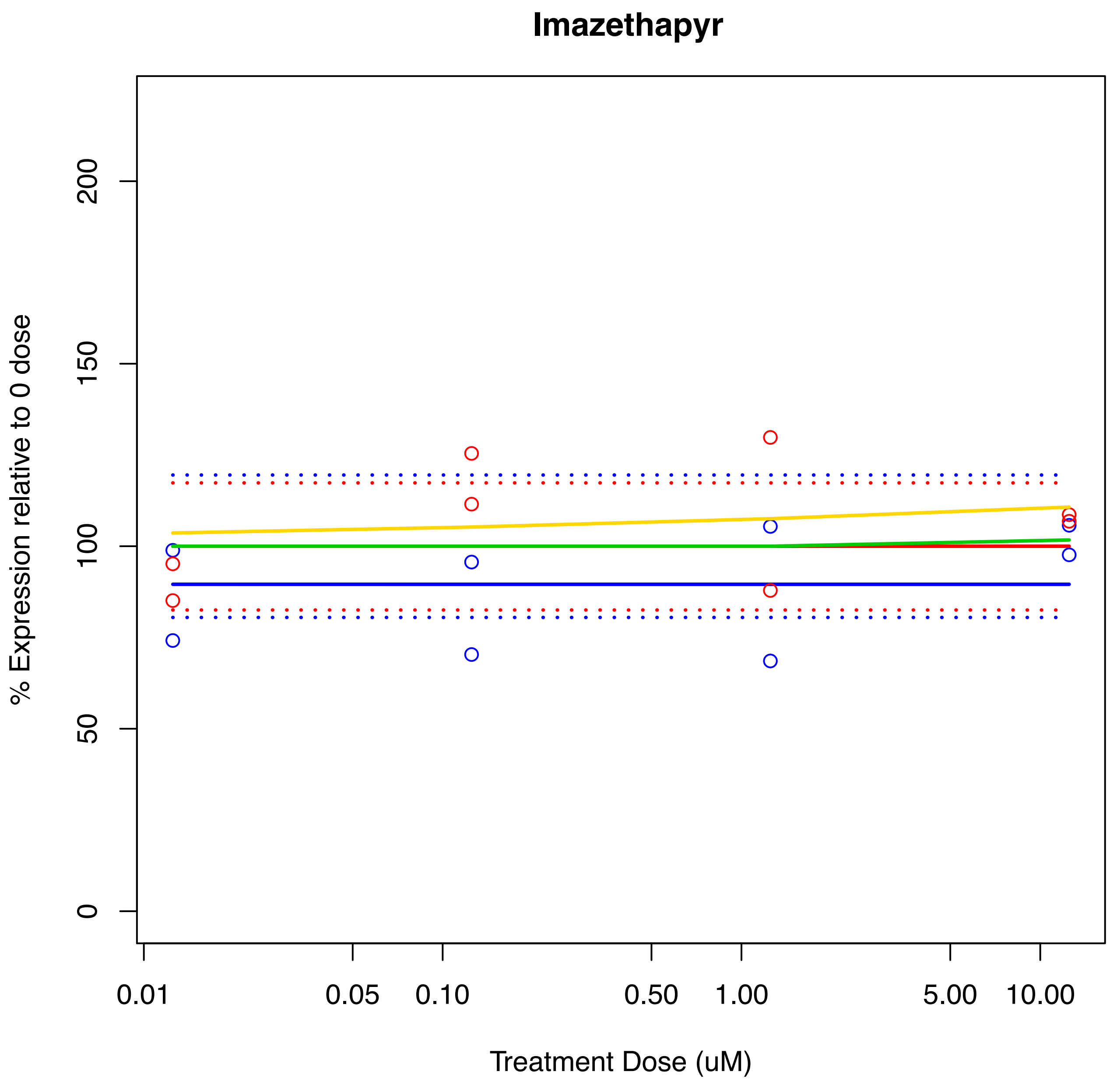

Imazethapyr
% Expression relative to 0 dose
0
50
100
150
200
0.01
0.05
0.10
0.50
1.00
5.00
10.00
Treatment Dose (uM)

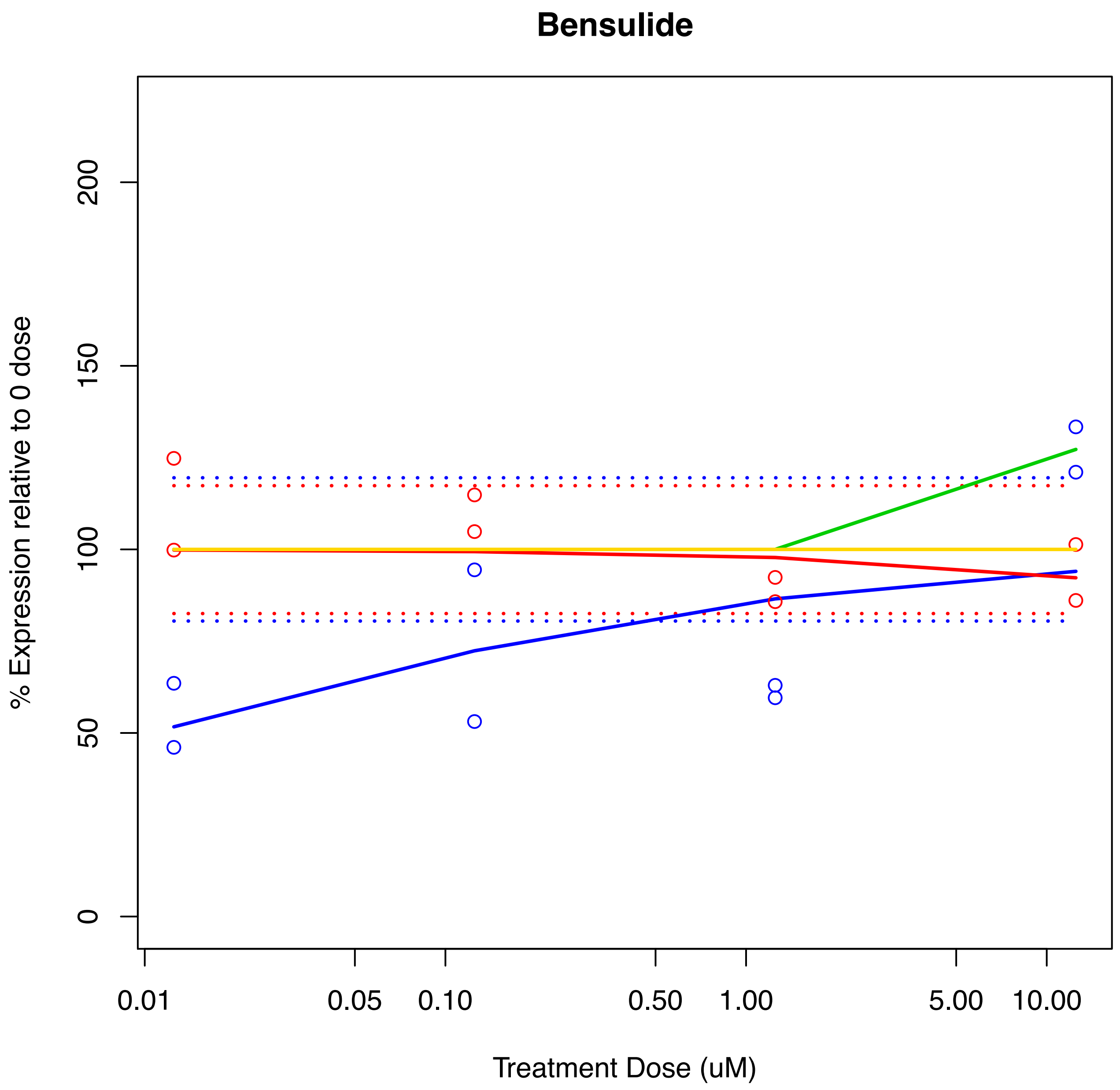

Bensulide
% Expression relative to 0 dose
0
50
100
150
200
0.01
0.05
0.10
0.50
1.00
5.00
10.00
Treatment Dose (uM)

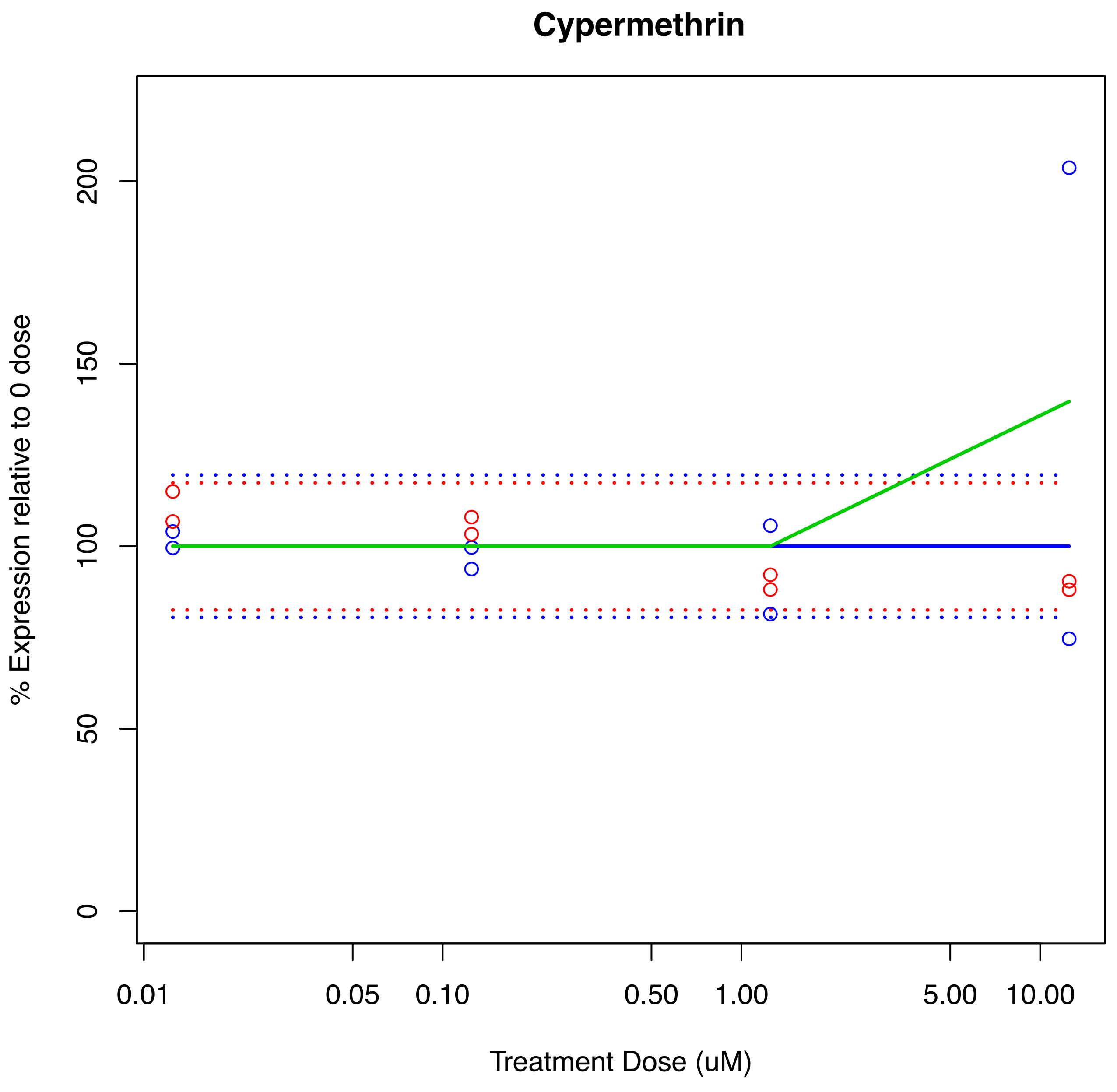

Cypermethrin
% Expression relative to 0 dose
0
50
100
150
200
0.01
0.05
0.10
0.50
1.00
5.00
10.00
Treatment Dose (uM)

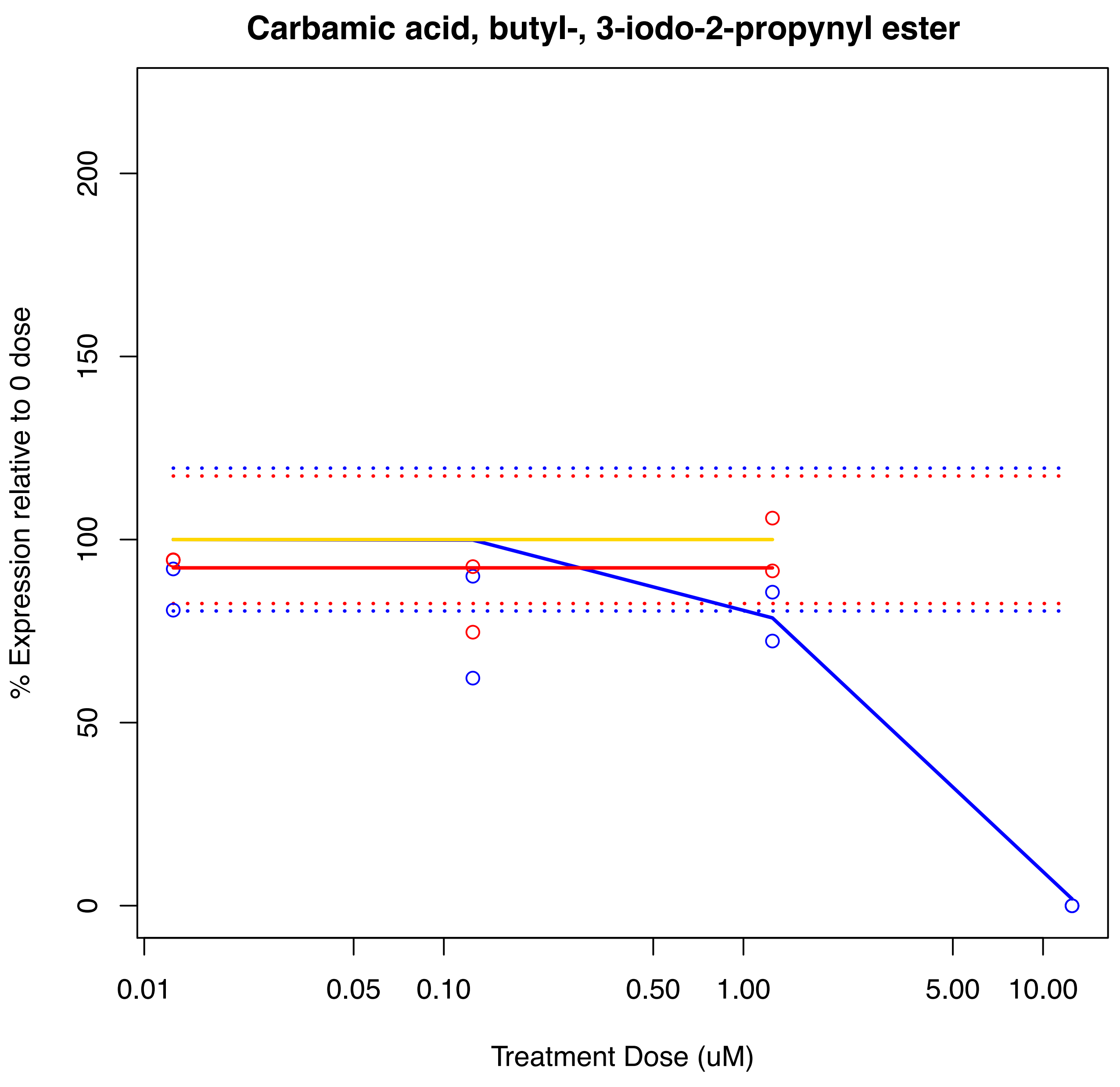
Carbamic acid, butyl-, 3-iodo-2-propynyl ester
% Expression relative to 0 dose
0
50
100
150
200
0.01
0.05
0.10
0.50
1.00
5.00
10.00
Treatment Dose (uM)

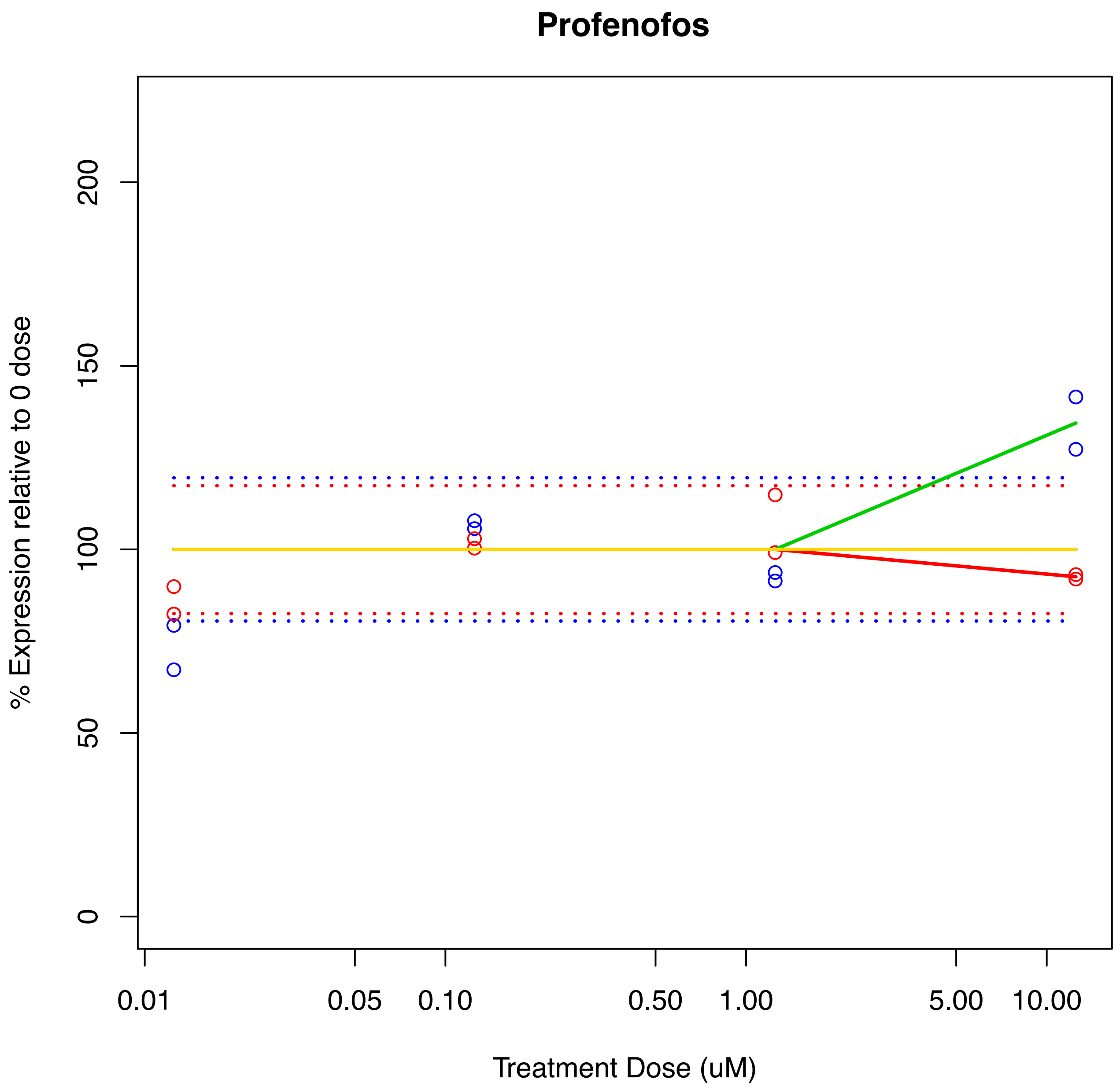

Profenofos
% Expression relative to 0 dose
0
50
100
150
200
0.01
0.05
0.10
0.50
1.00
5.00
10.00
Treatment Dose (uM)

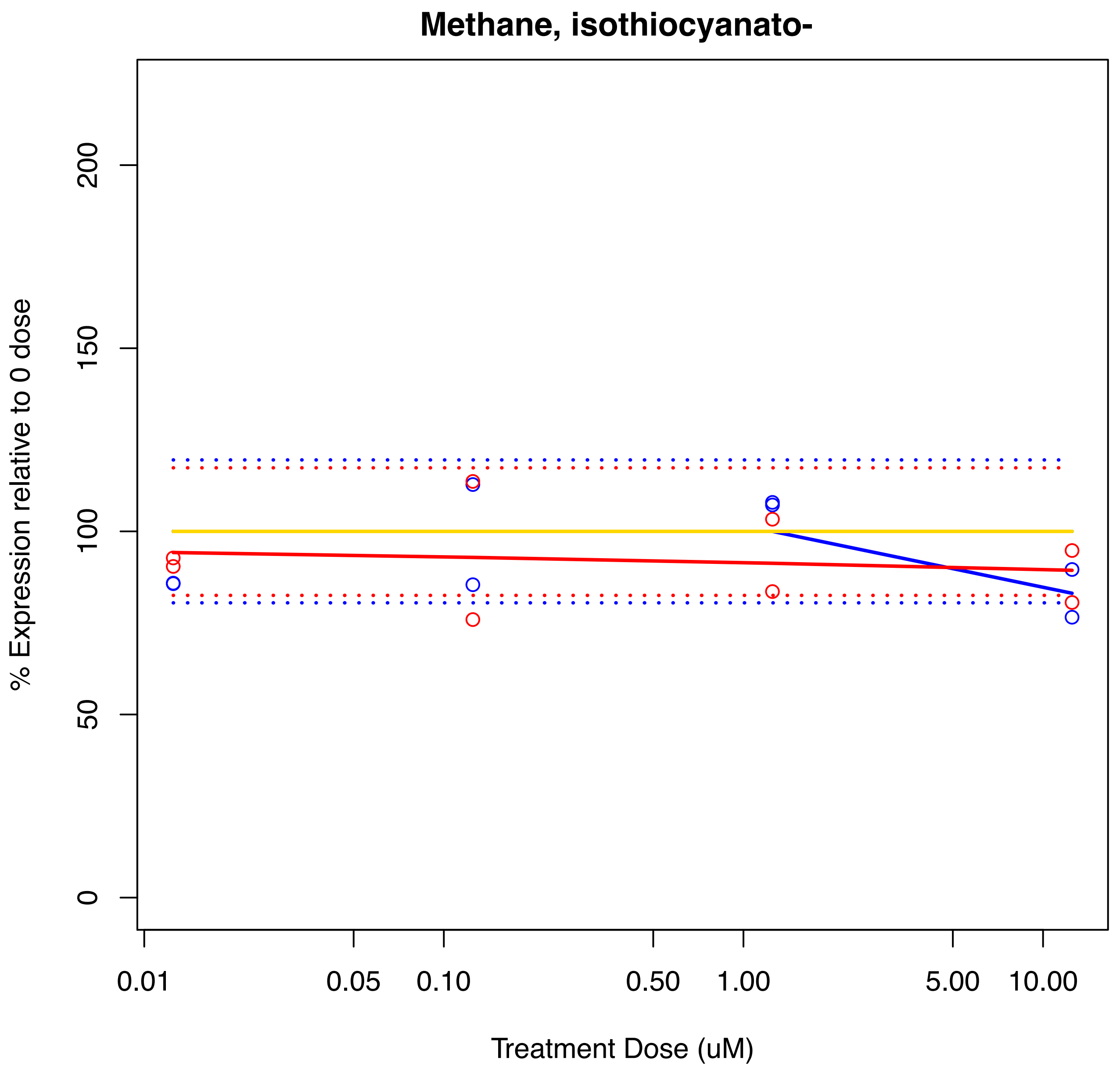
Methane, isothiocyanato-
% Expression relative to 0 dose
0
50
100
150
200
0.01
0.05
0.10
0.50
1.00
5.00
10.00
Treatment Dose (uM)

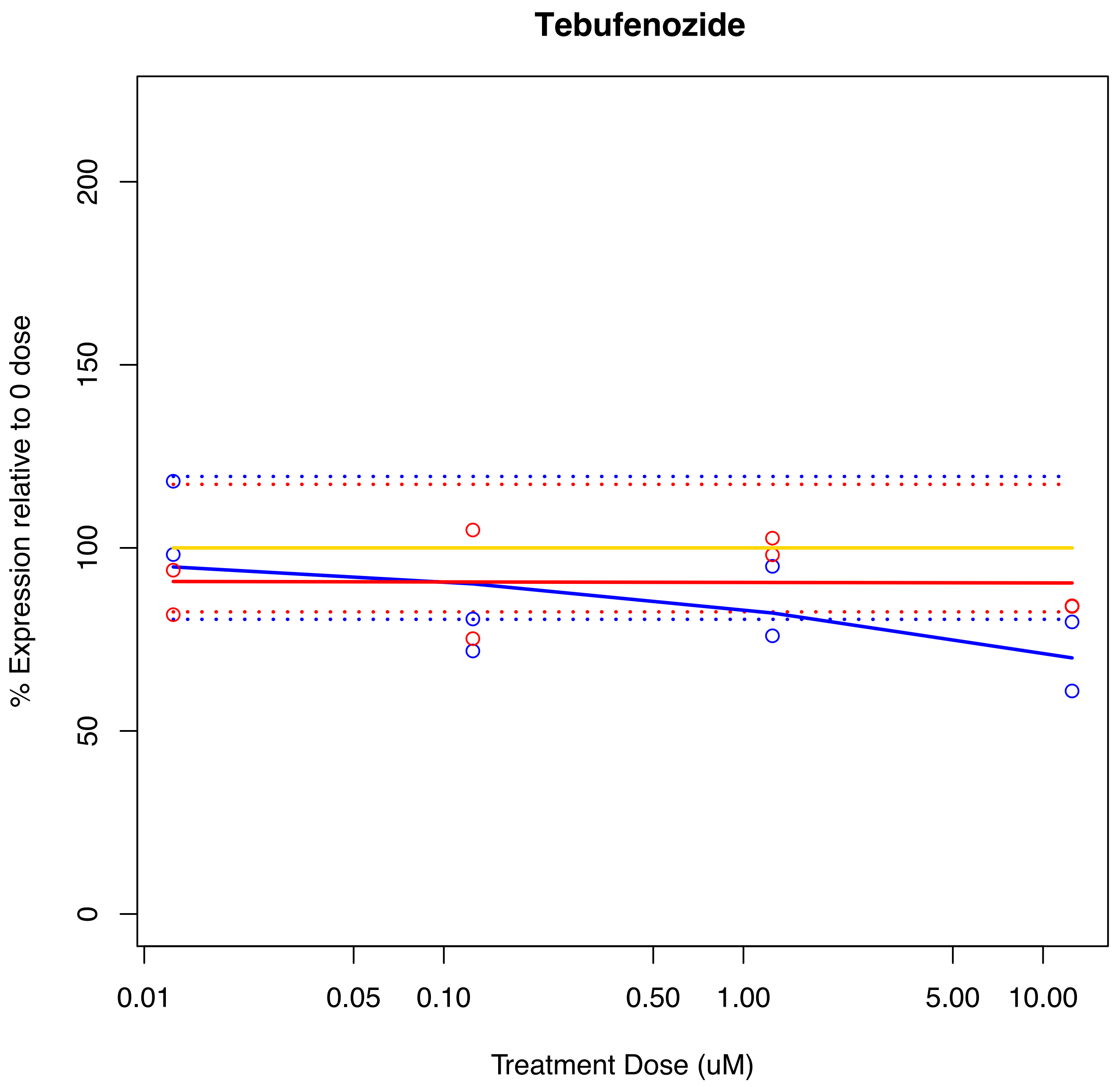

Tebufenozide
% Expression relative to 0 dose
0
50
100
150
200
0.01
0.05
0.10
0.50
1.00
5.00
10.00
Treatment Dose (uM)

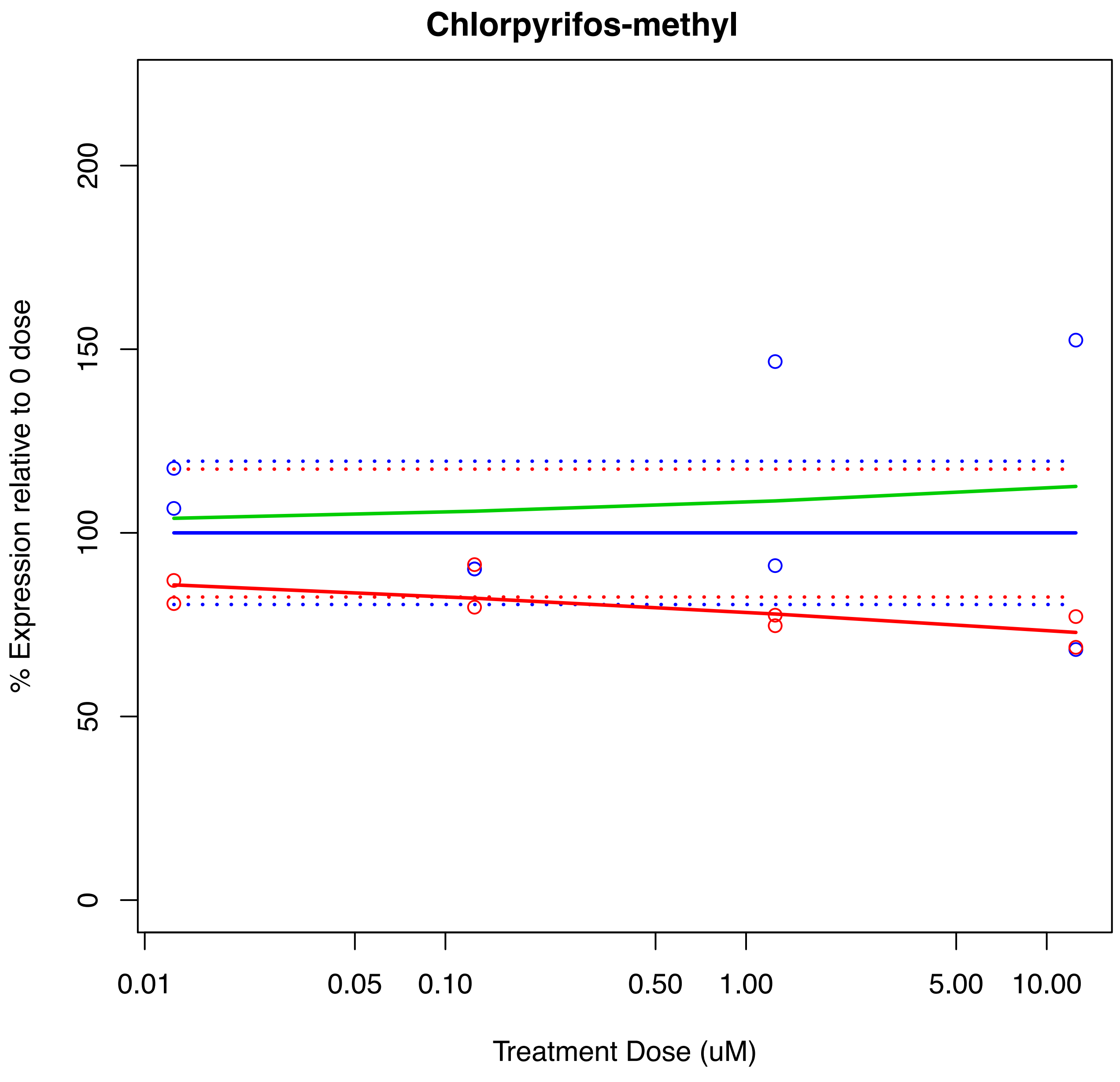
Chlorpyrifos-methyl
% Expression relative to 0 dose
0
50
100
150
200
0.01
0.05
0.10
0.50
1.00
5.00
10.00
Treatment Dose (uM)

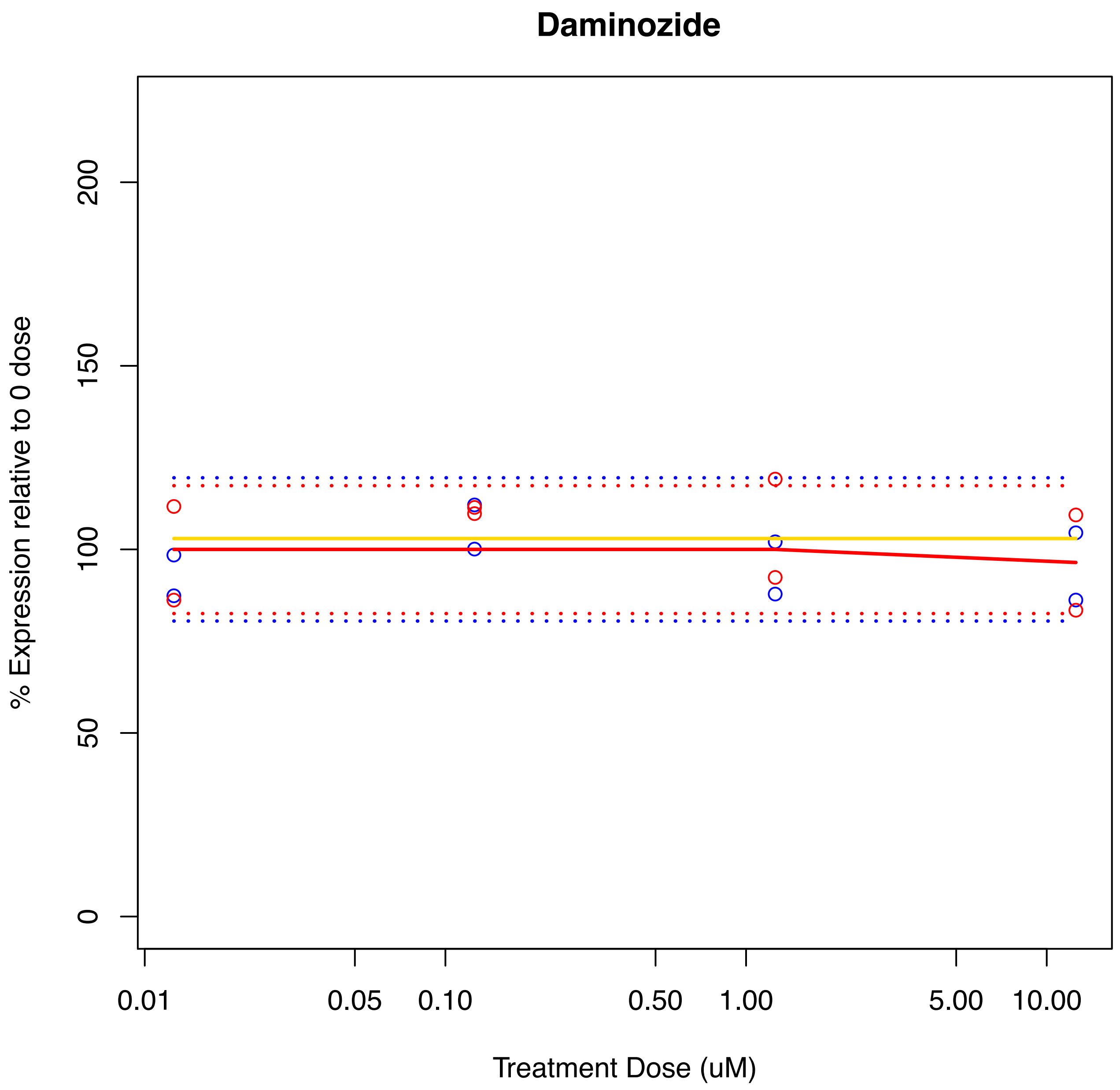
Daminozide
% Expression relative to 0 dose
0
50
100
150
200
0.01
0.05
0.10
0.50
1.00
5.00
10.00
Treatment Dose (uM)

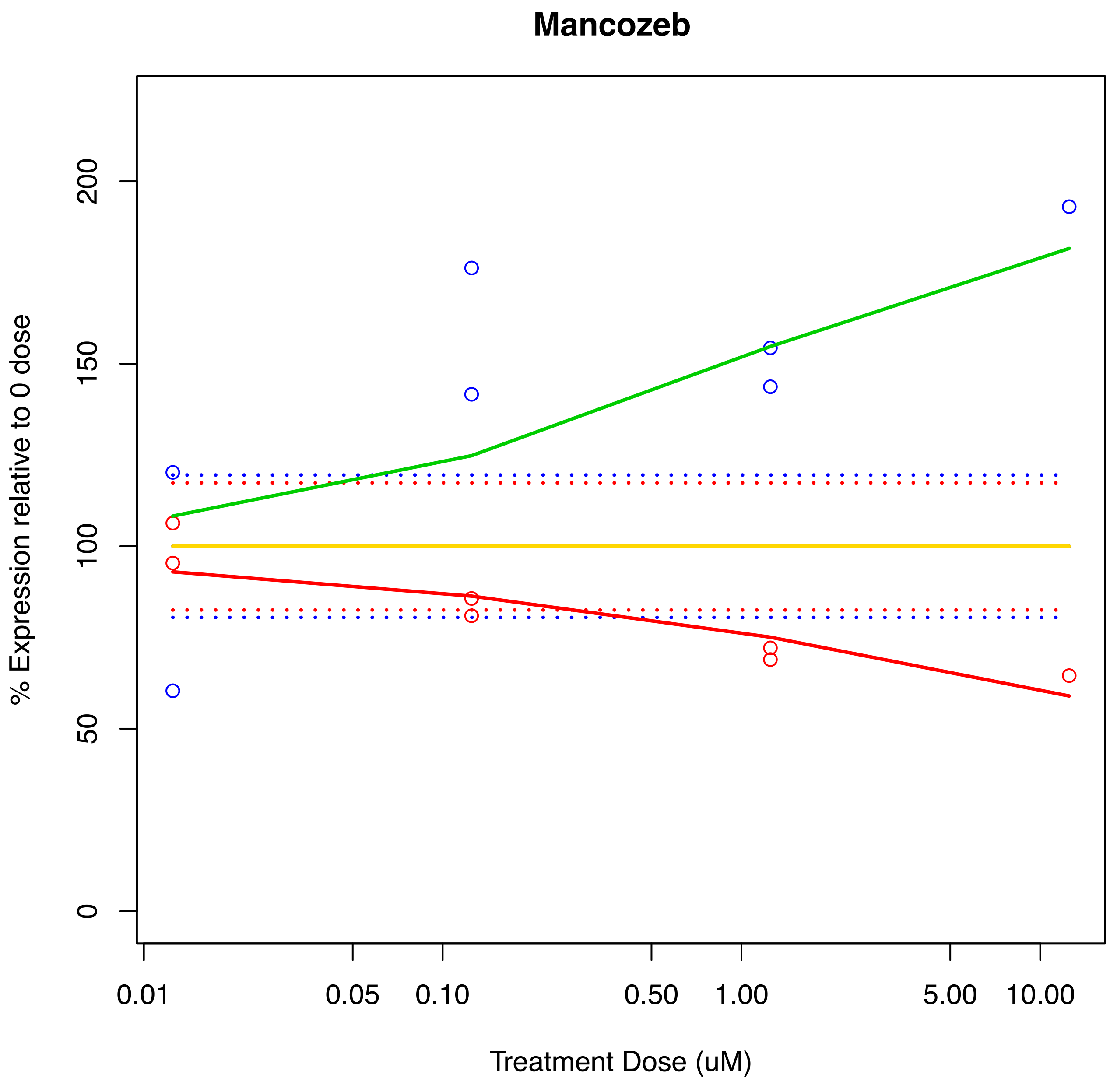
Mancozeb
% Expression relative to 0 dose
0
50
100
150
200
0.01
0.05
0.10
0.50
1.00
5.00
10.00
Treatment Dose (uM)

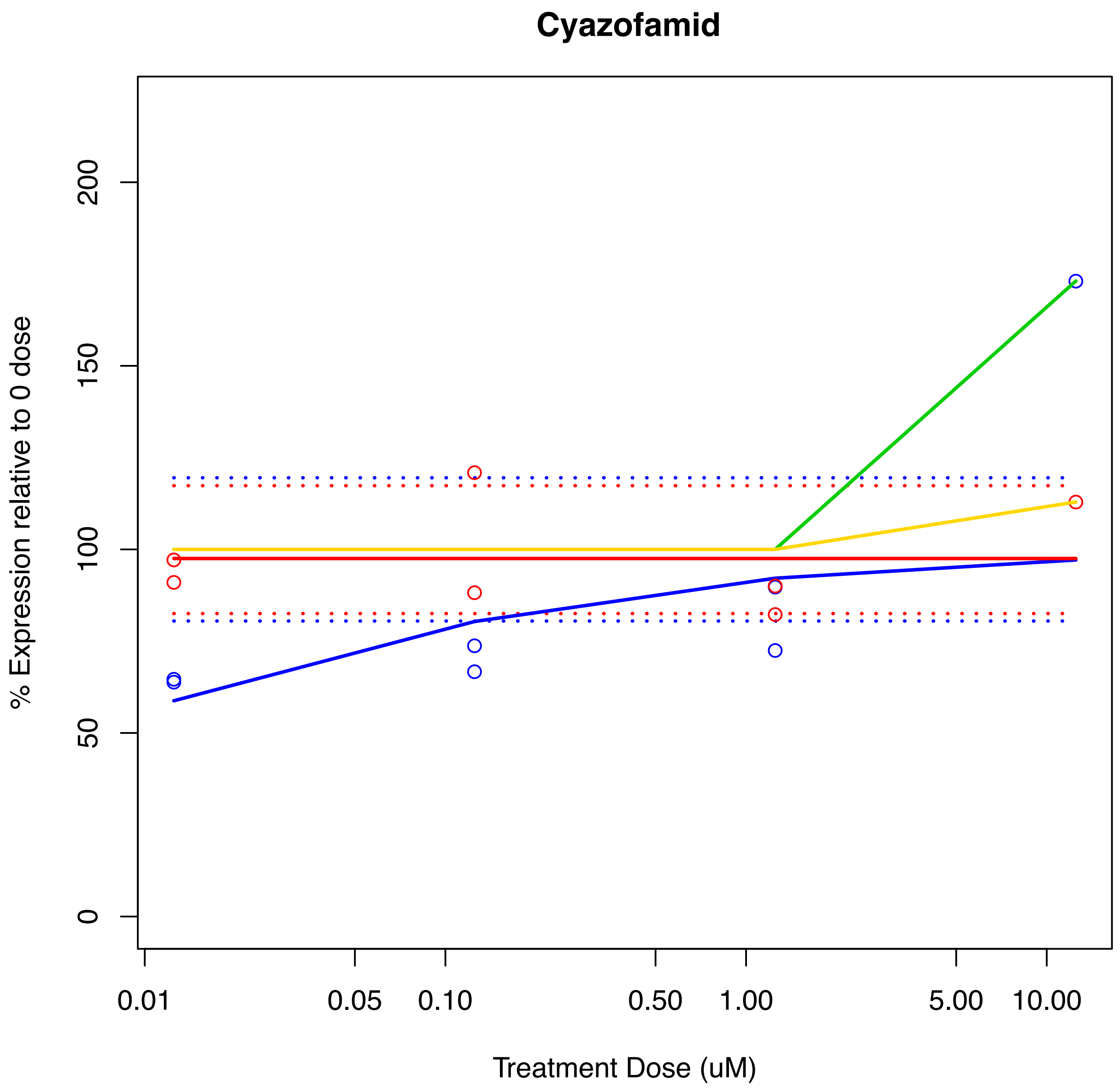

Cyazofamid
% Expression relative to 0 dose
0
50
100
150
200
0.01
0.05
0.10
0.50
1.00
5.00
10.00
Treatment Dose (uM)

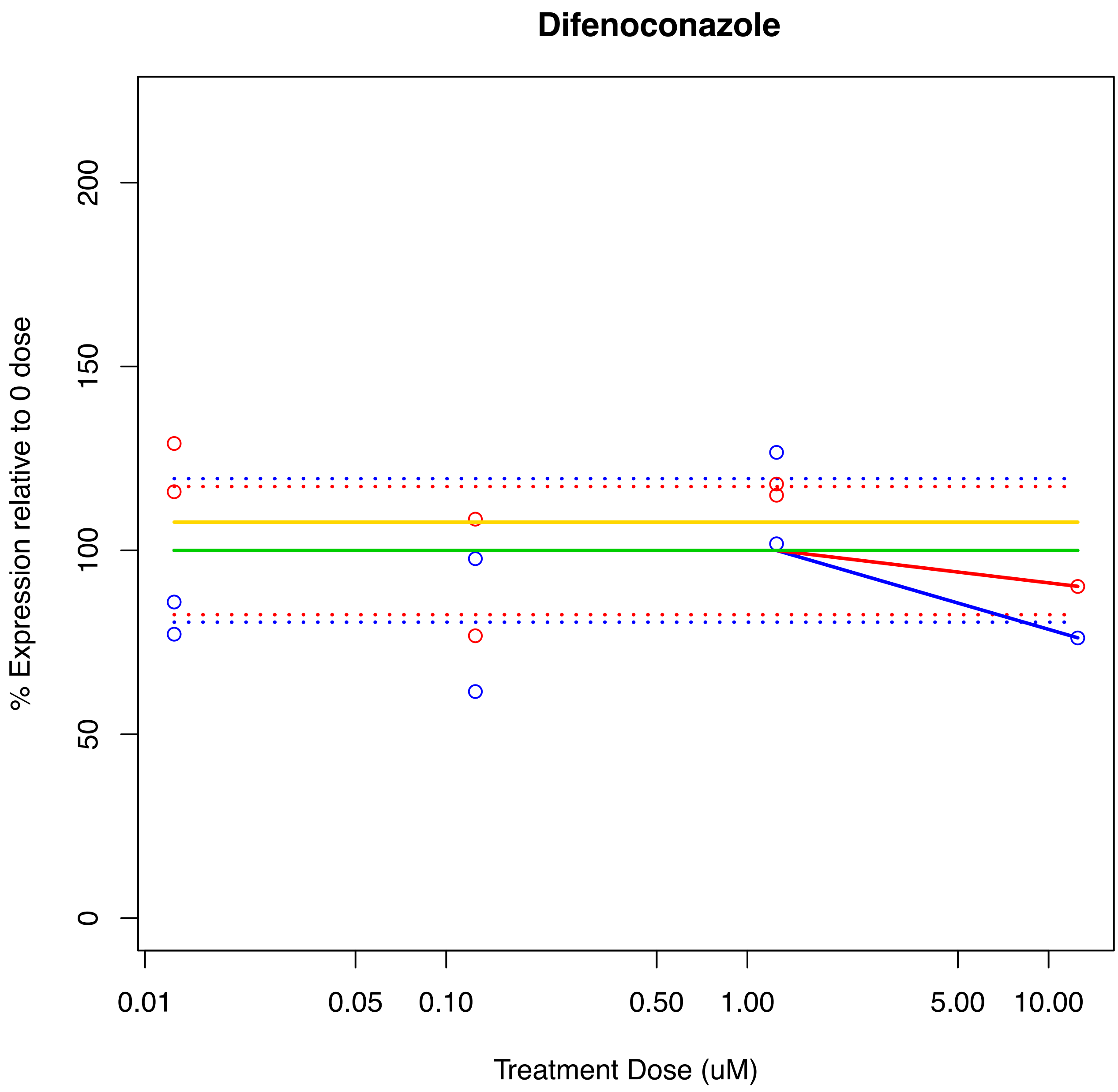

Difenoconazole
% Expression relative to 0 dose
0
50
100
150
200
0.01
0.05
0.10
0.50
1.00
5.00
10.00
Treatment Dose (uM)

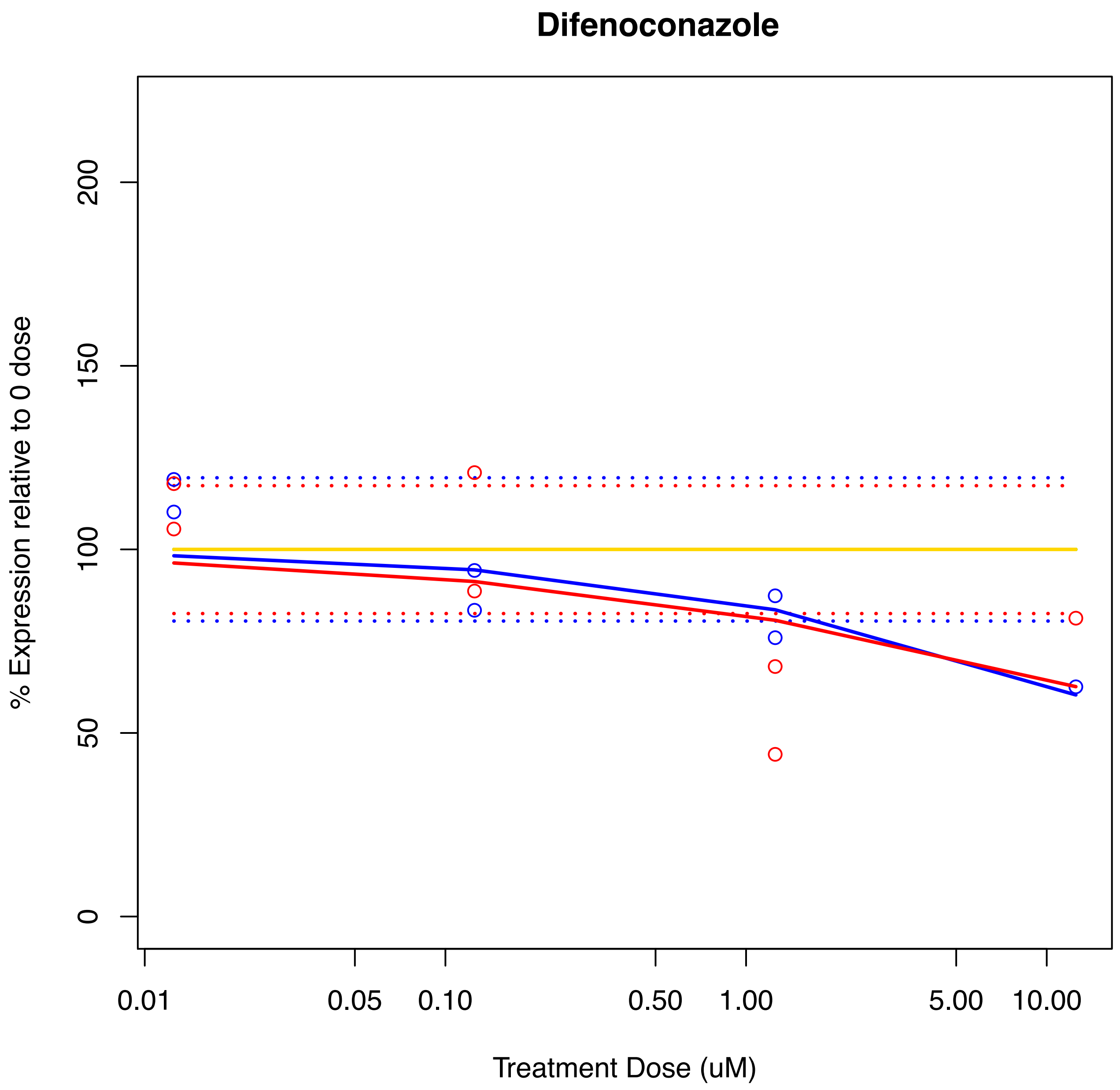

Difenoconazole
% Expression relative to 0 dose
0
50
100
150
200
0.01
0.05
0.10
0.50
1.00
5.00
10.00
Treatment Dose (uM)

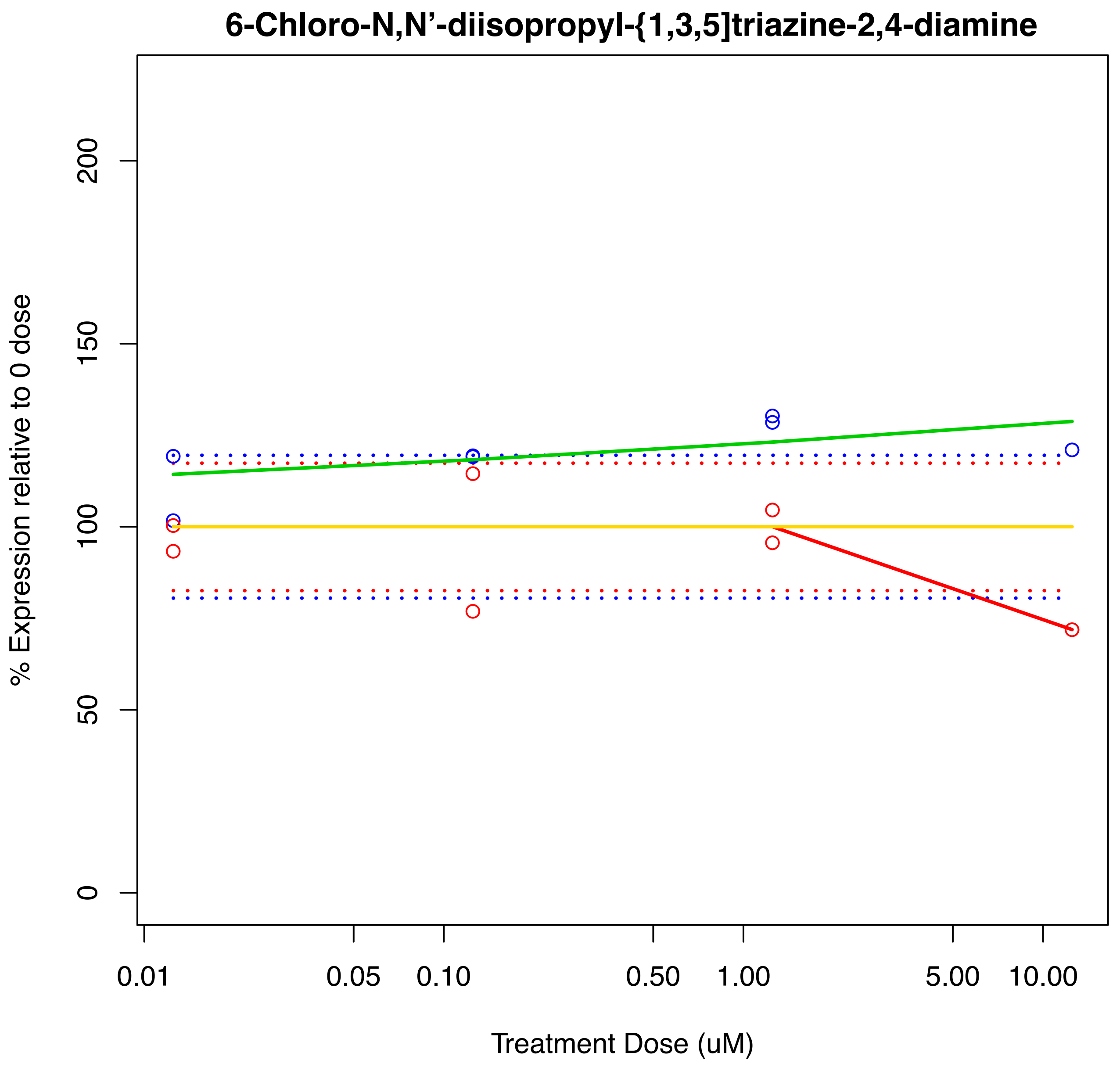
6-Chloro-N,N'-diisopropyl-{1,3,5]triazine-2,4-diamine
% Expression relative to 0 dose
0
50
100
150
200
0.01
0.05
0.10
0.50
1.00
5.00
10.00
Treatment Dose (uM)

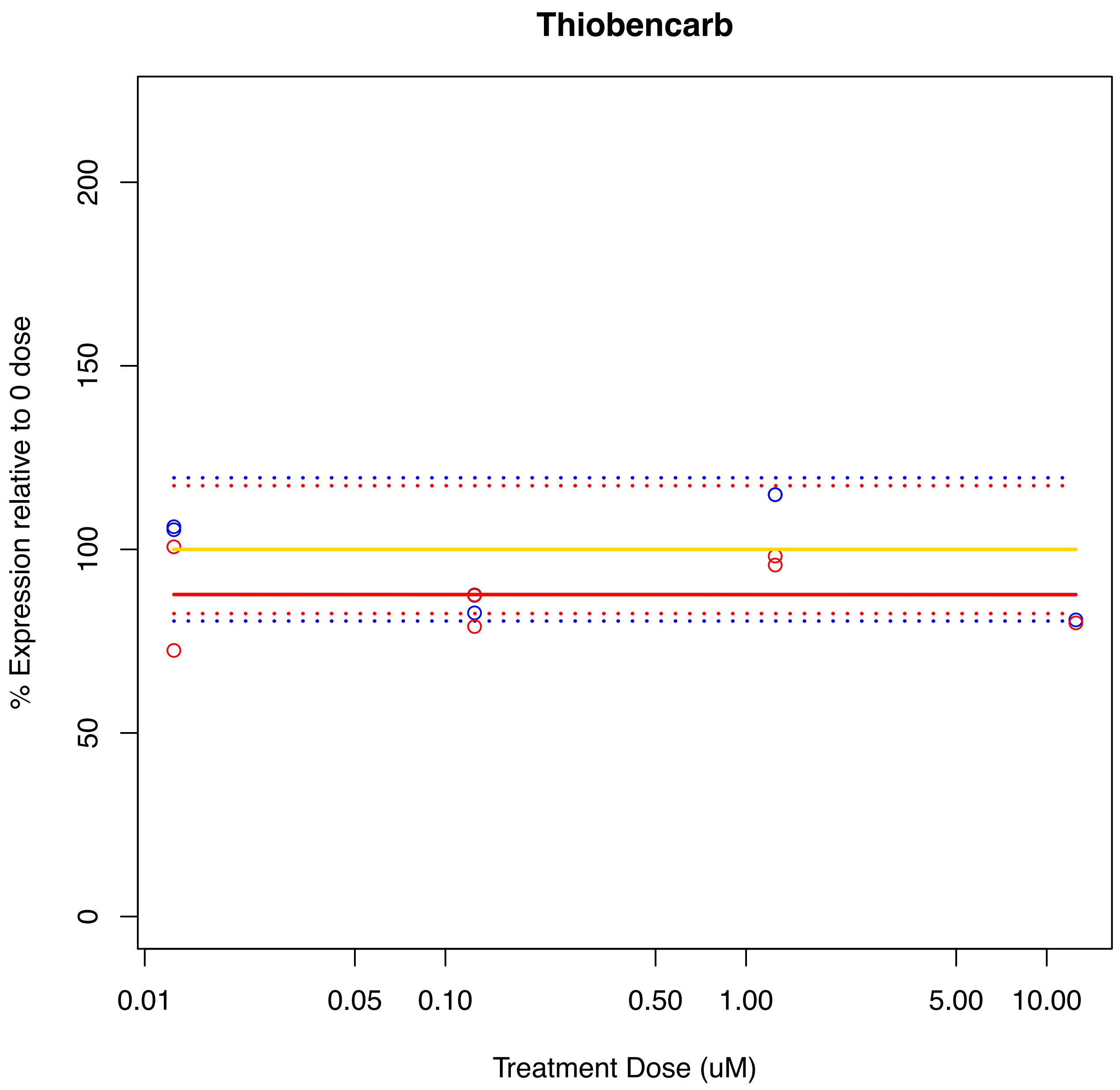

Thiobencarb
% Expression relative to 0 dose
0
50
100
150
200
0.01
0.05
0.10
0.50
1.00
5.00
10.00
Treatment Dose (uM)

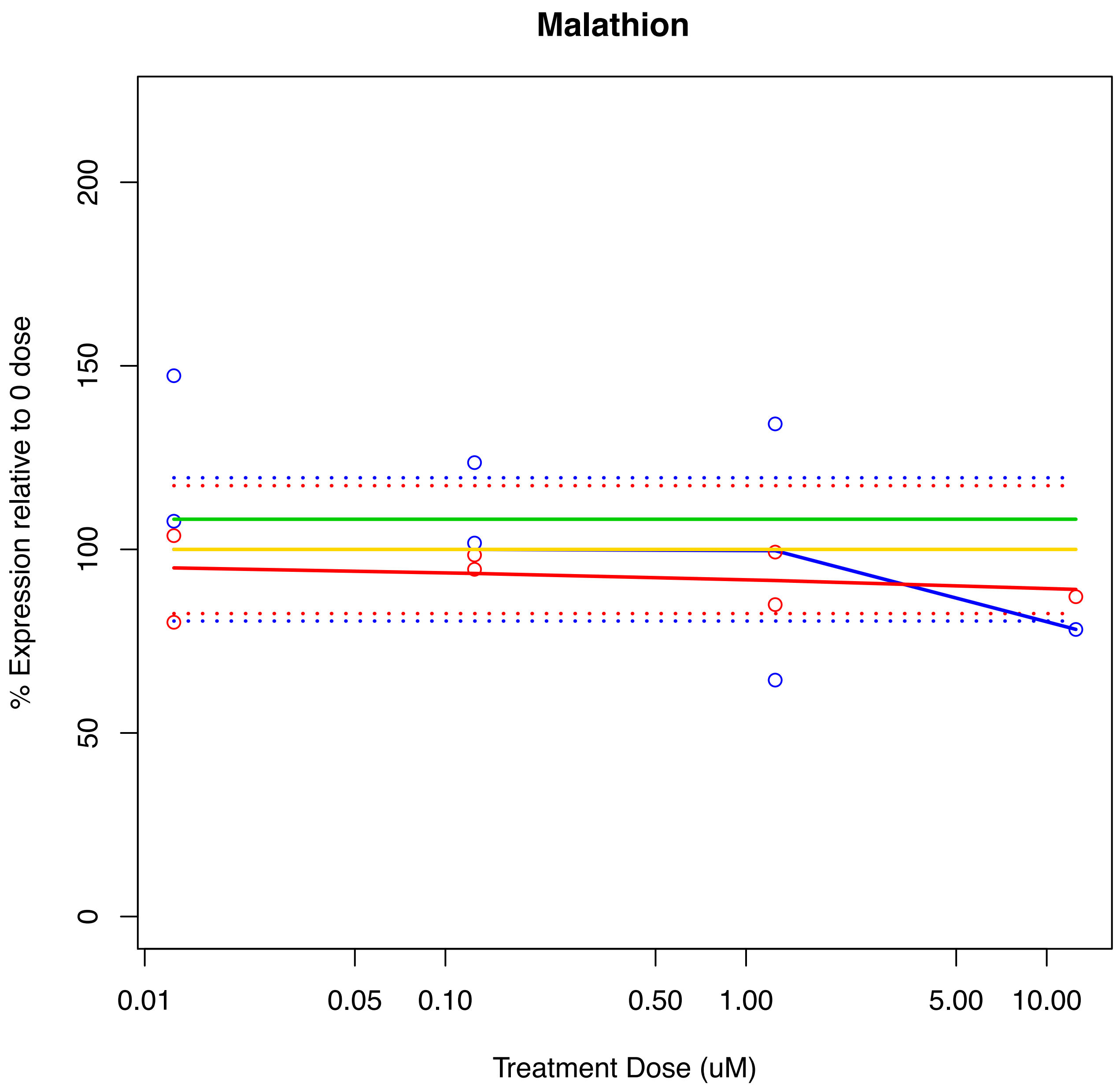
Malathion
% Expression relative to 0 dose
0
50
100
150
200
0.01
0.05
0.10
0.50
1.00
5.00
10.00
Treatment Dose (uM)

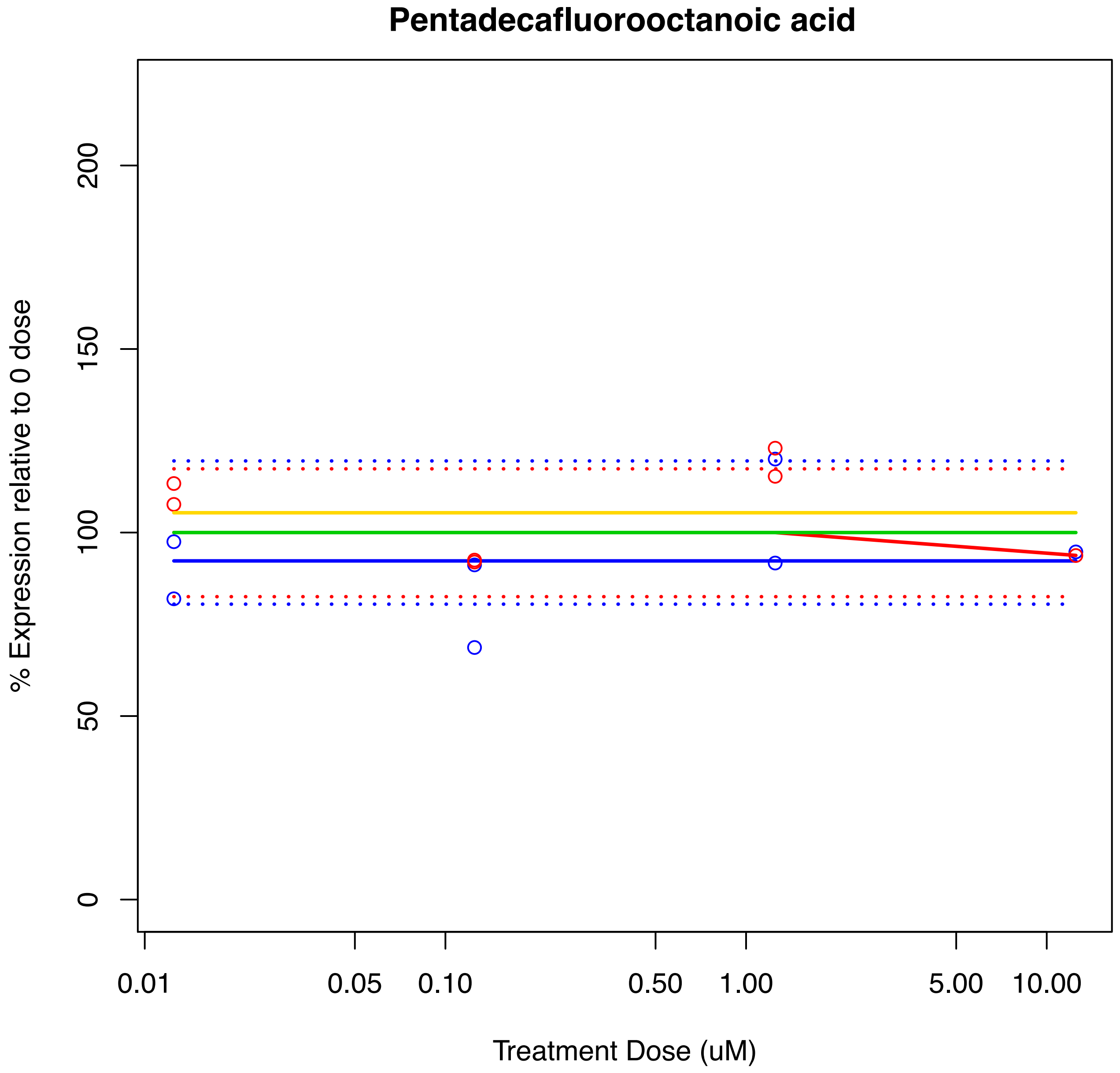

Pentadecafluorooctanoic acid
% Expression relative to 0 dose
0
50
100
150
200
0.01
0.05
0.10
0.50
1.00
5.00
10.00
Treatment Dose (uM)

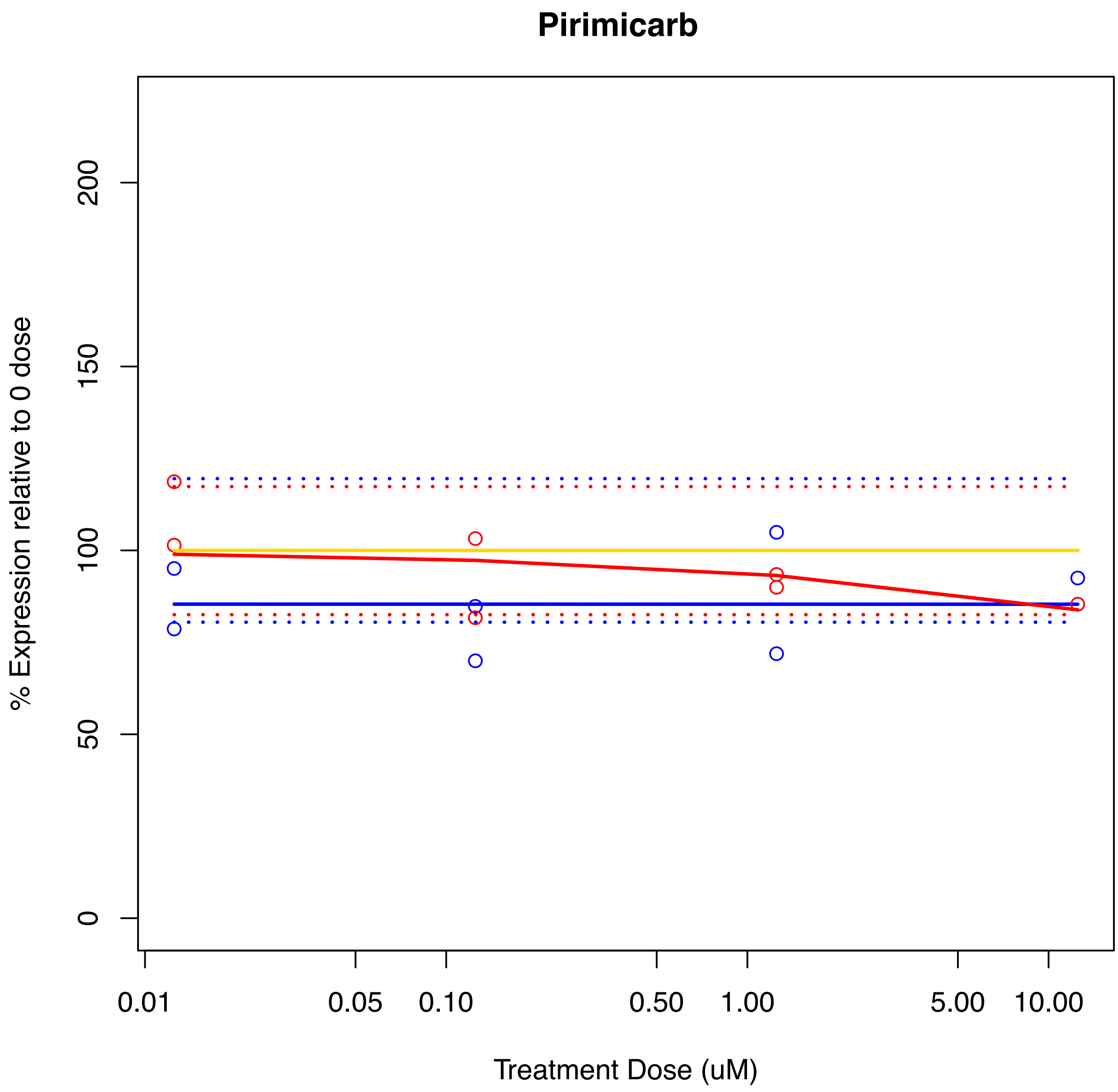

Pirimicarb
% Expression relative to 0 dose
0
50
100
150
200
0.01
0.05
0.10
0.50
1.00
5.00
10.00
Treatment Dose (uM)

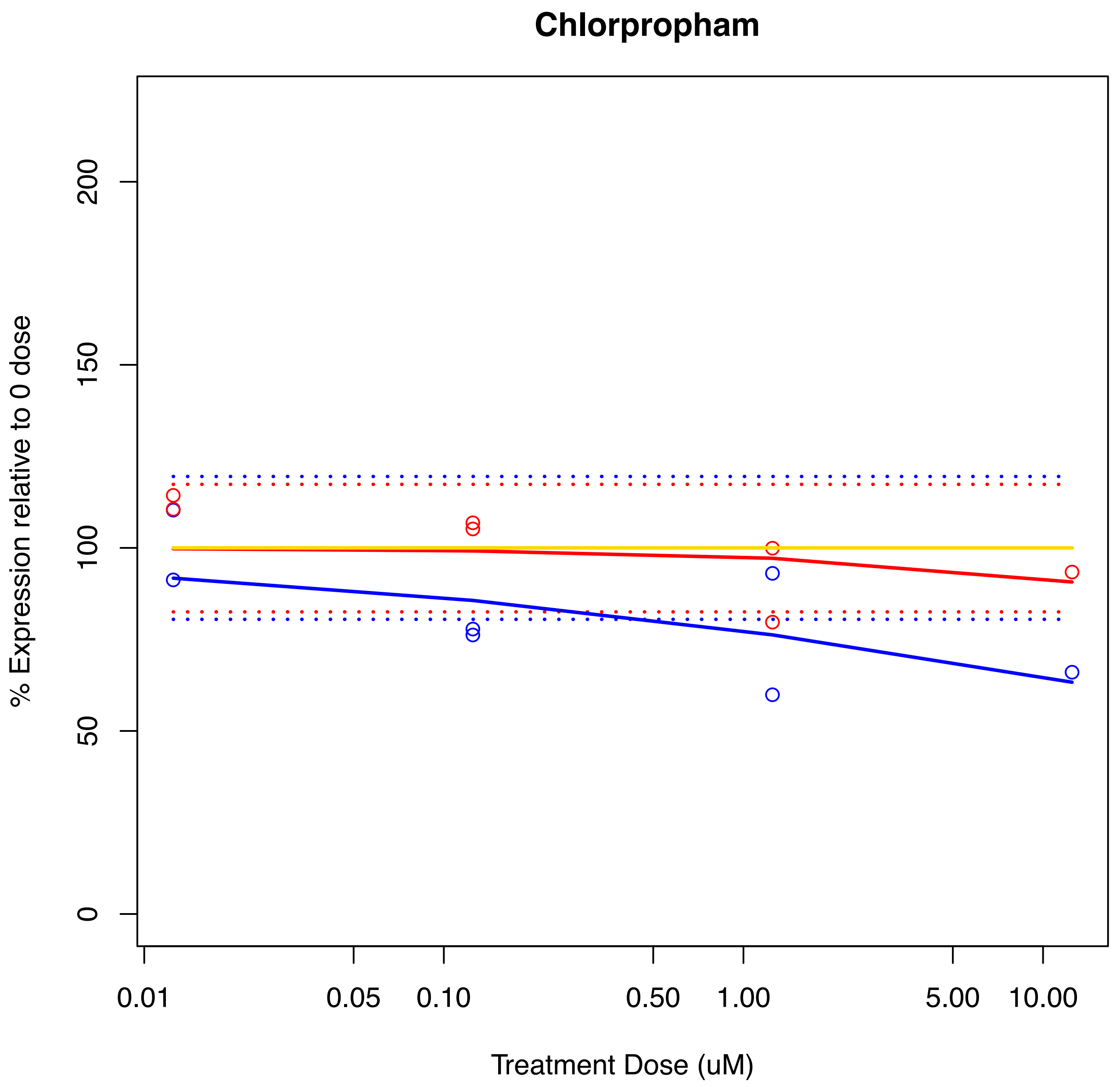

Chlorpropham
% Expression relative to 0 dose
0
50
100
150
200
0.01
0.05
0.10
0.50
1.00
5.00
10.00
Treatment Dose (uM)

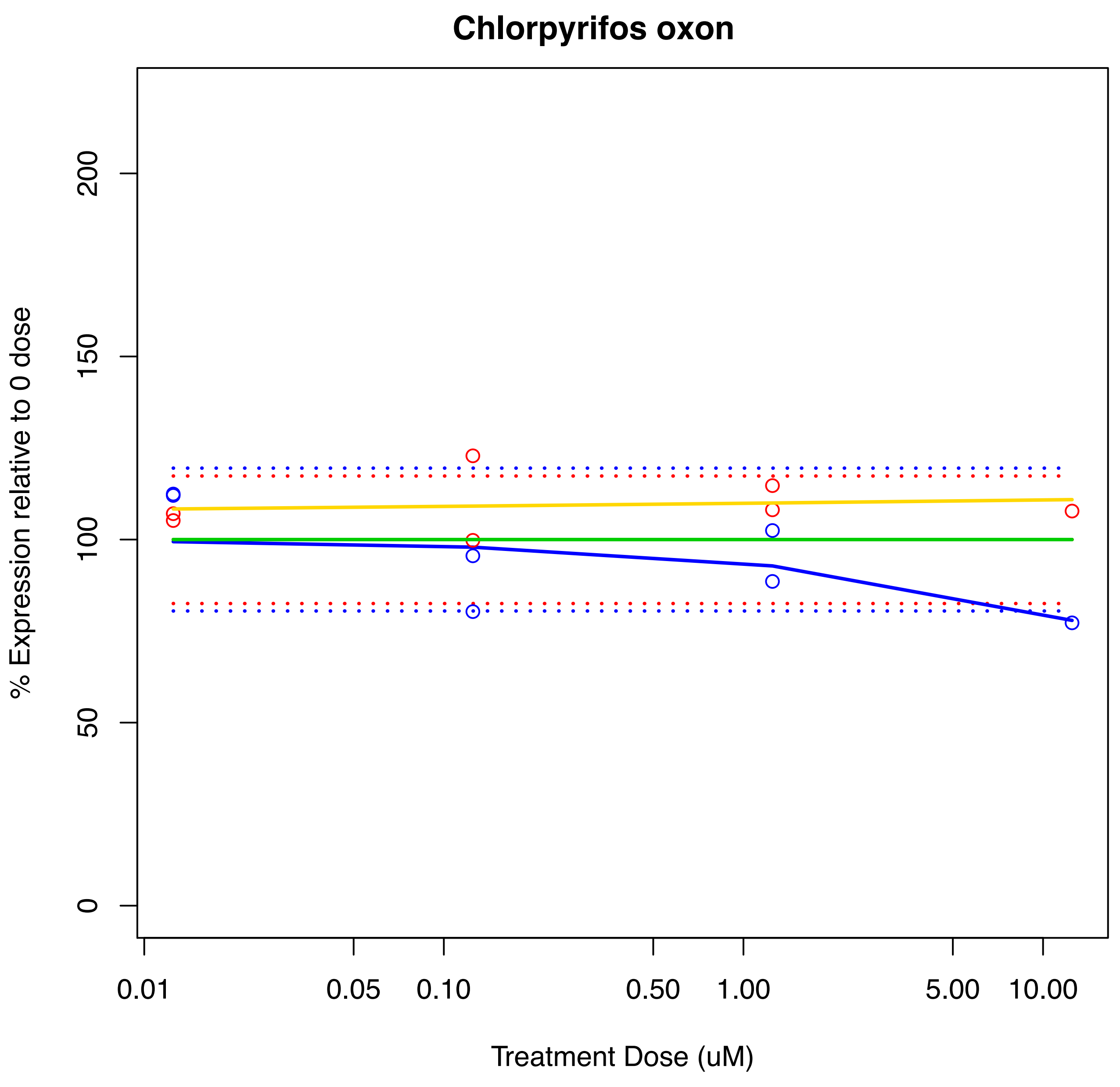
Chlorpyrifos oxon
% Expression relative to 0 dose
0
50
100
150
200
0.01
0.05
0.10
0.50
1.00
5.00
10.00
Treatment Dose (uM)

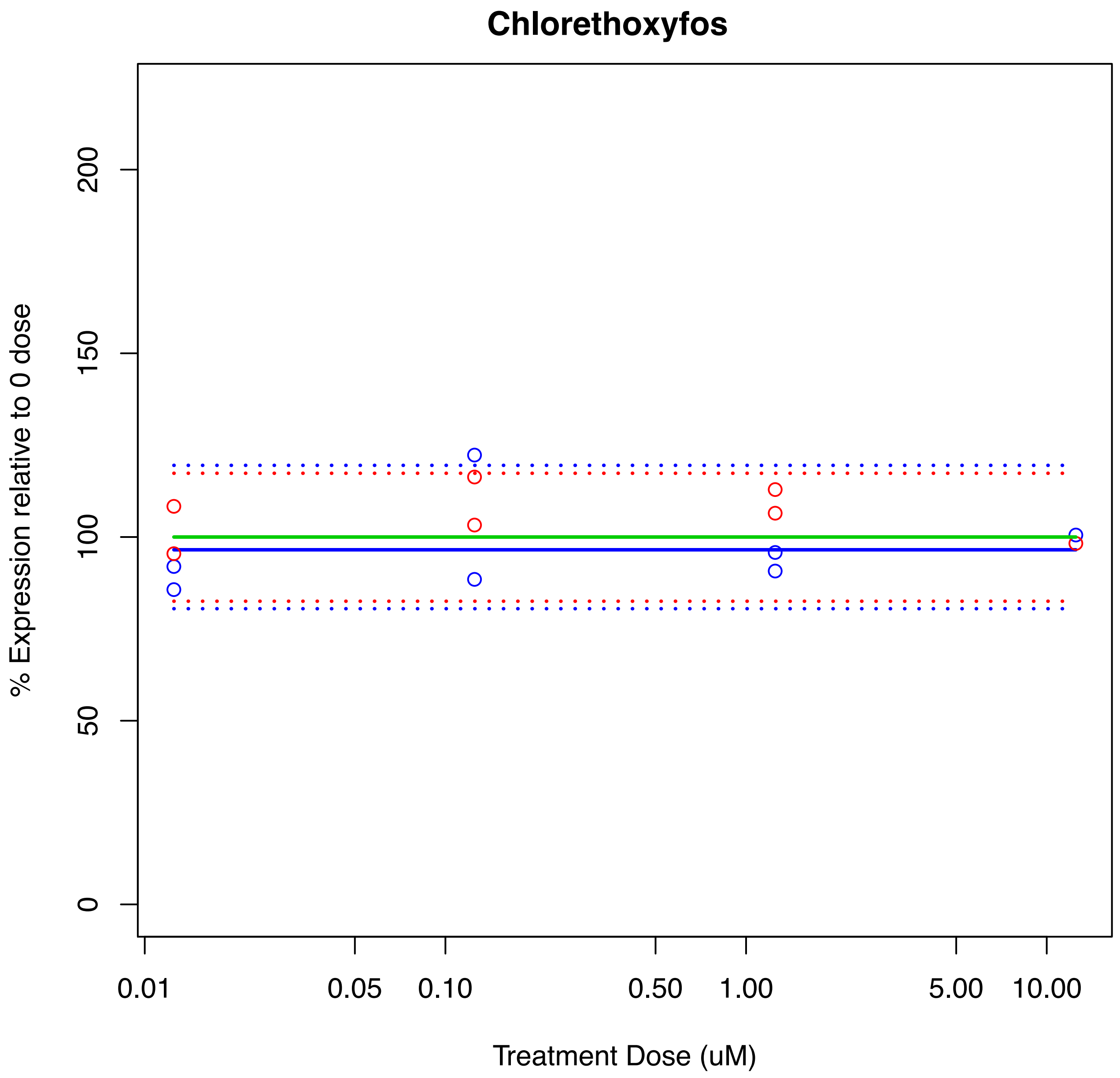

Chlorethoxyfos
% Expression relative to 0 dose
0
50
100
150
200
0.01
0.05
0.10
0.50
1.00
5.00
10.00
Treatment Dose (uM)

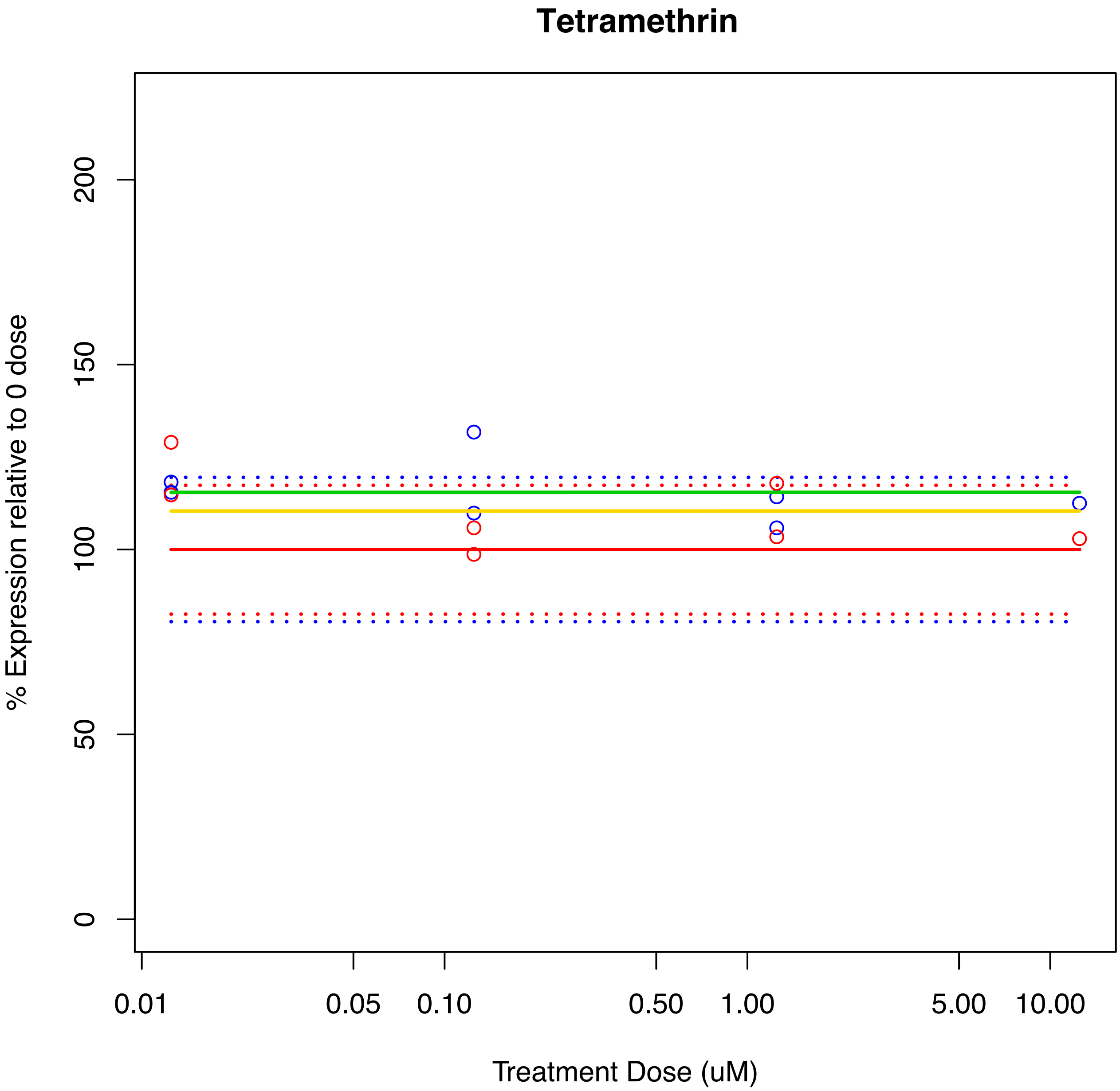

Tetramethrin
% Expression relative to 0 dose
0
50
100
150
200
0.01
0.05
0.10
0.50
1.00
5.00
10.00
Treatment Dose (uM)

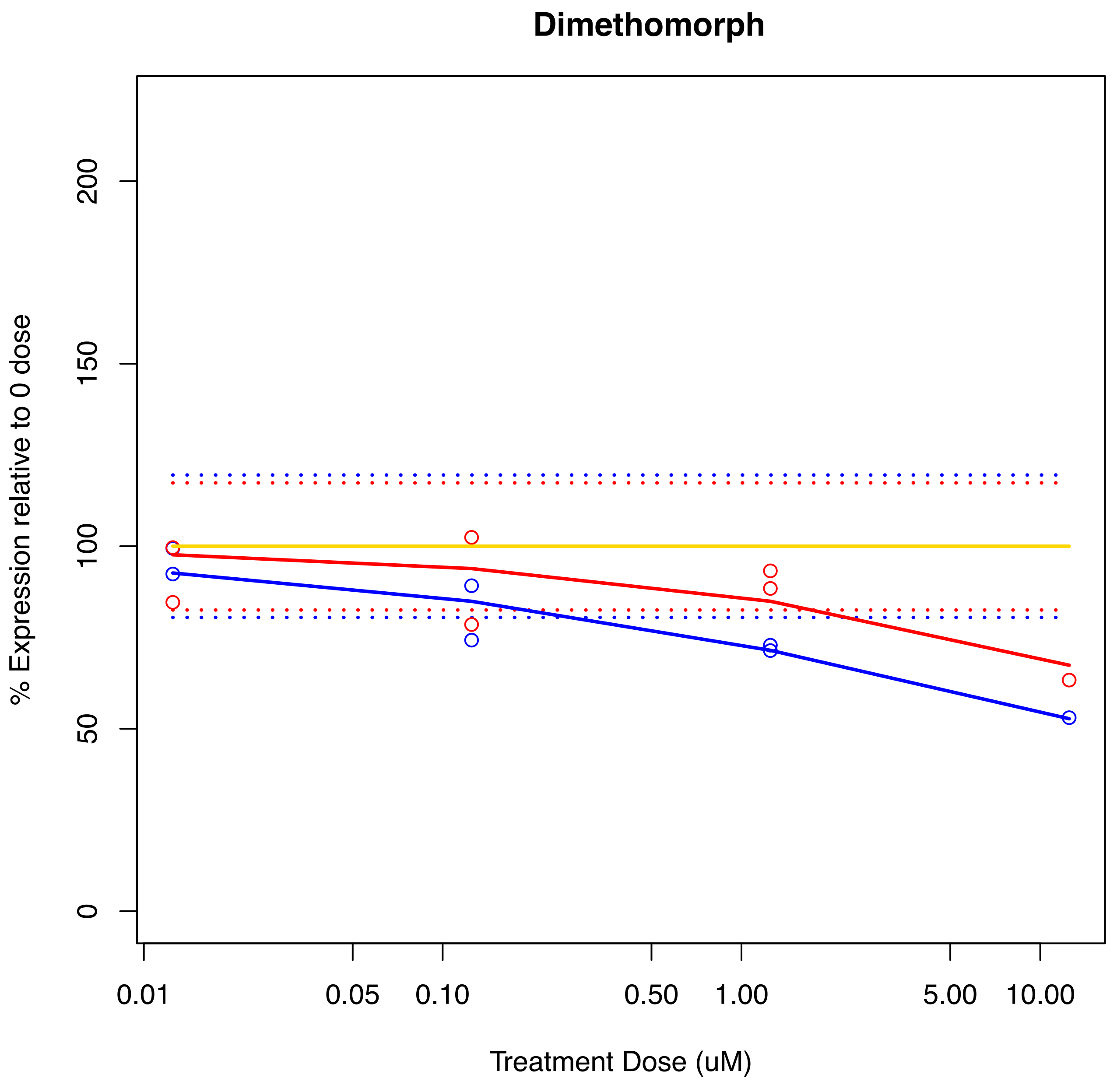

Dimethomorph
% Expression relative to 0 dose
0
50
100
150
200
0.01
0.05
0.10
0.50
1.00
5.00
10.00
Treatment Dose (uM)

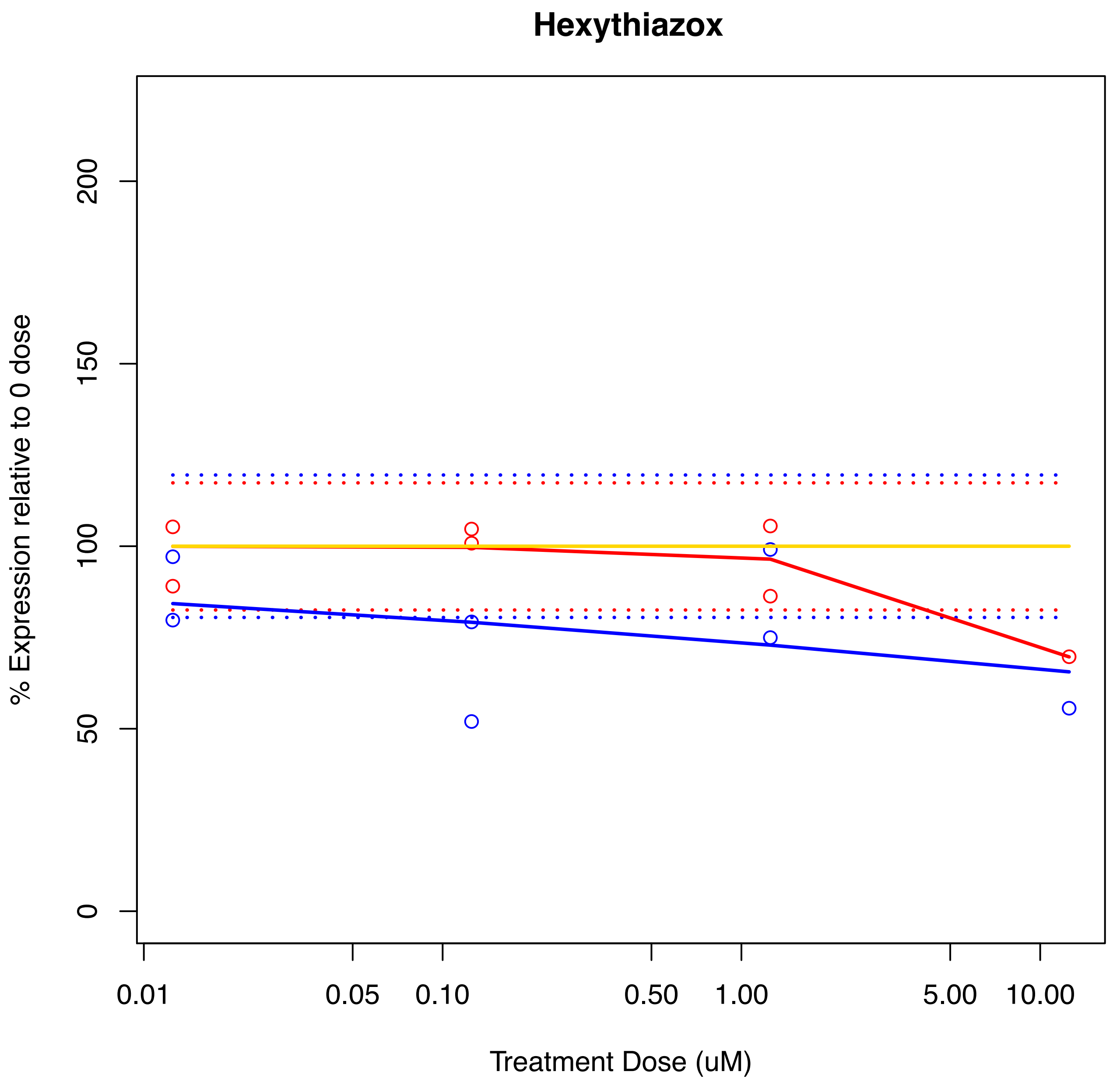
Hexythiazox
% Expression relative to 0 dose
0
50
100
150
200
0.01
0.05
0.10
0.50
1.00
5.00
10.00
Treatment Dose (uM)

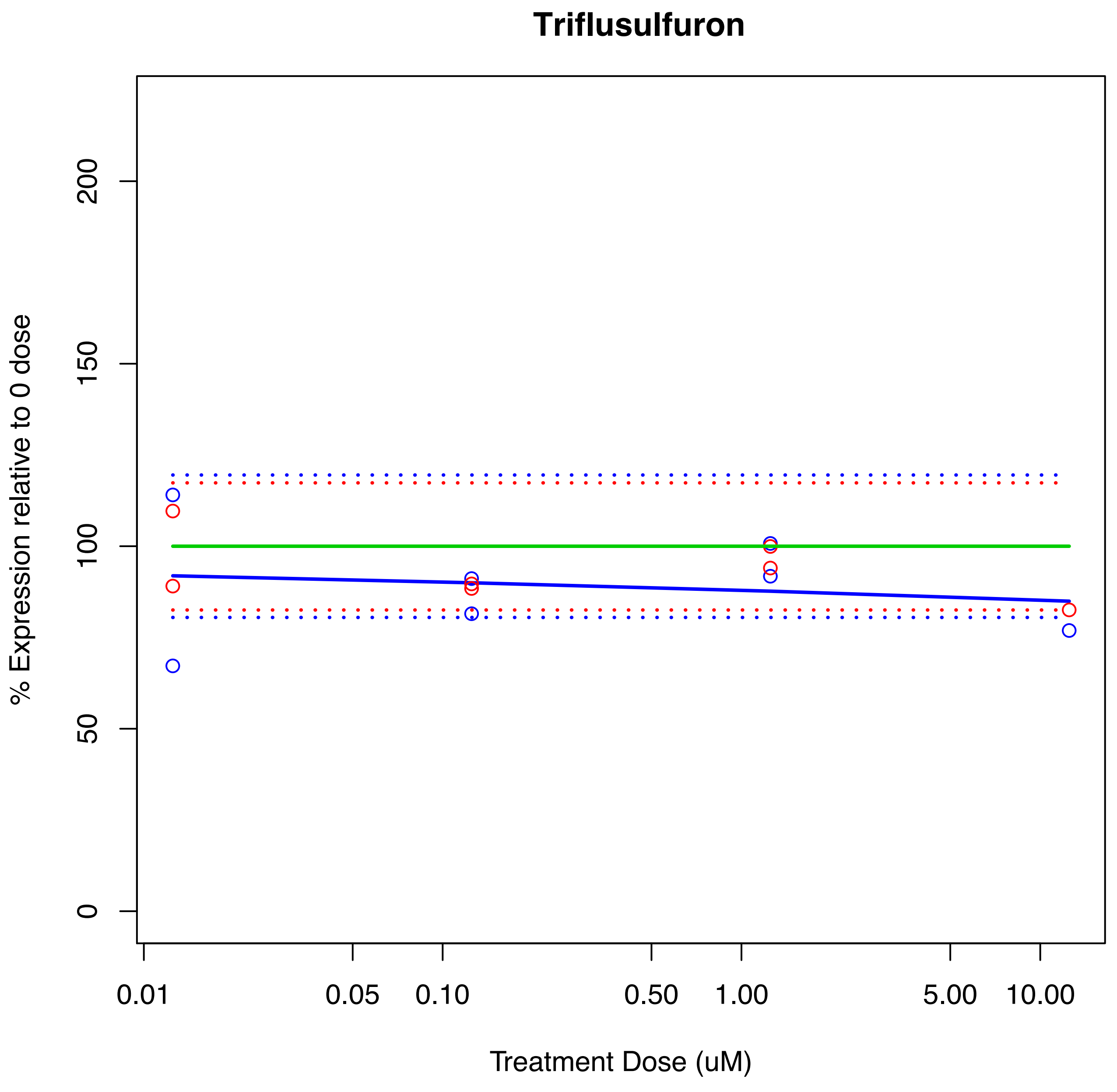
Triflusulfuron
% Expression relative to 0 dose
0
50
100
150
200
0.01
0.05
0.10
0.50
1.00
5.00
10.00
Treatment Dose (uM)

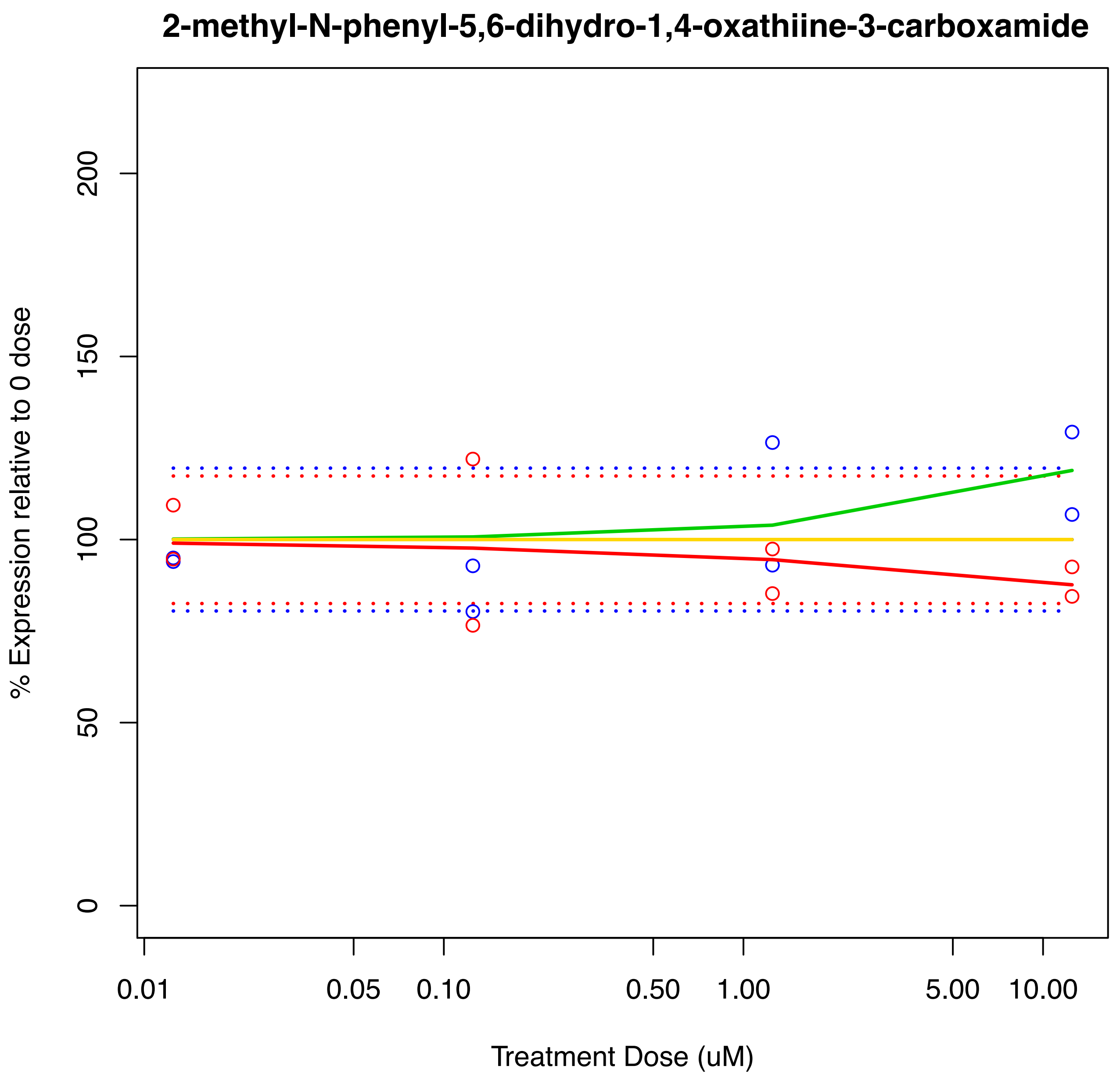

2-methyl-N-phenyl-5,6-dihydro-1,4-oxathiine-3-carboxamide
% Expression relative to 0 dose
0
50
100
150
200
0.01
0.05
0.10
0.50
1.00
5.00
10.00
Treatment Dose (uM)

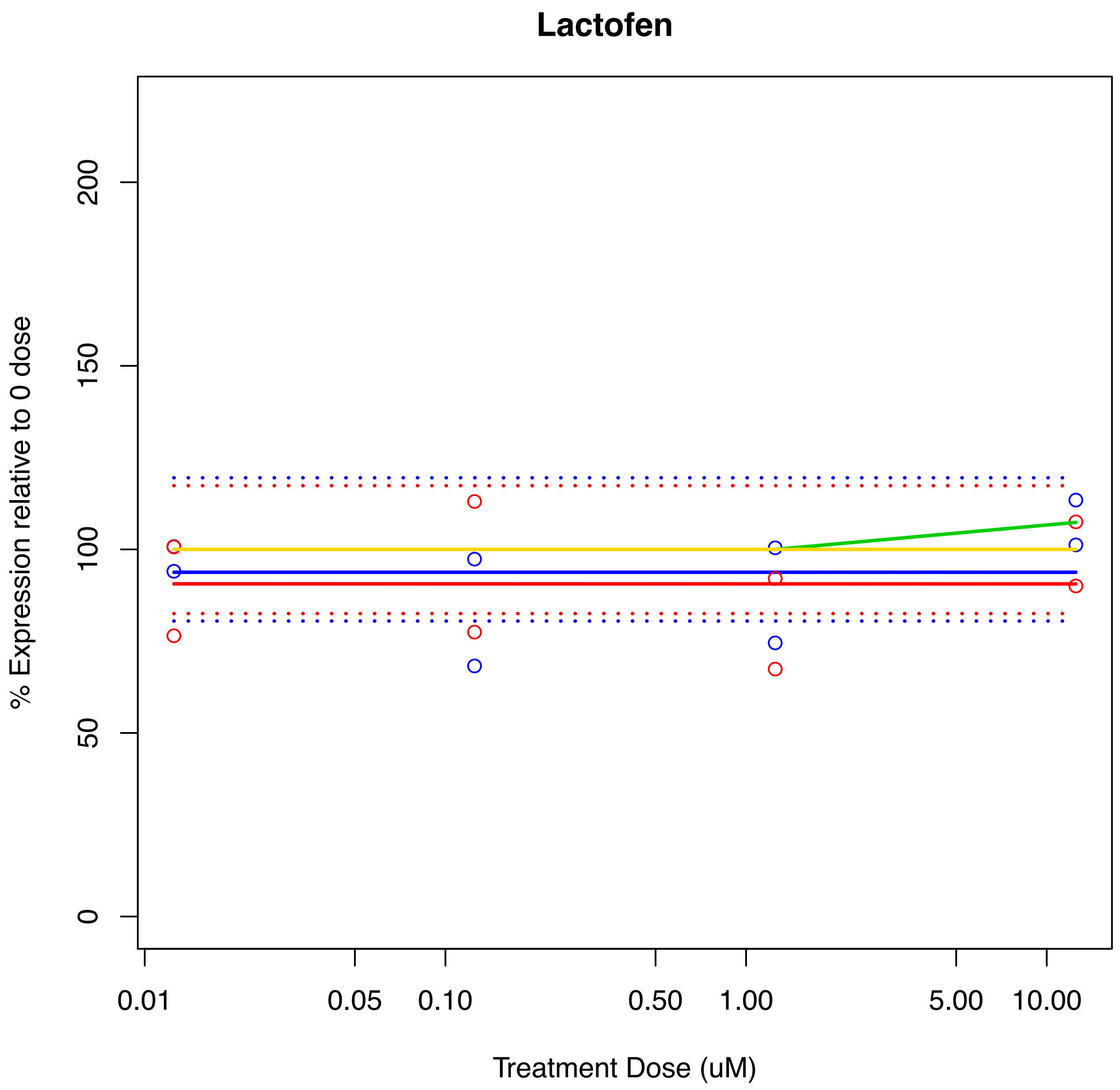
Lactofen
% Expression relative to 0 dose
0
50
100
150
200
0.01
0.05
0.10
0.50
1.00
5.00
10.00
Treatment Dose (uM)

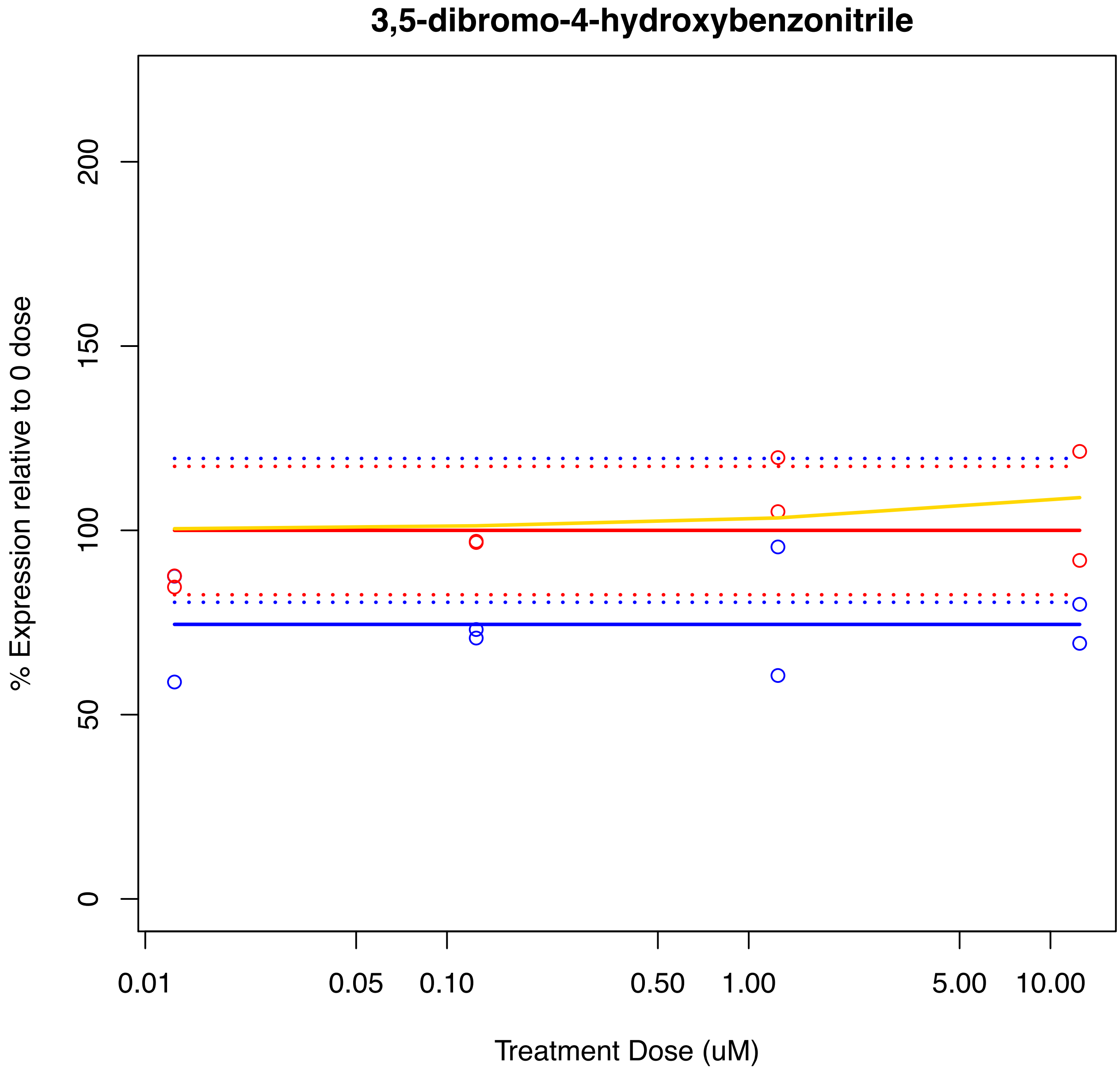
3,5-dibromo-4-hydroxybenzonitrile
% Expression relative to 0 dose
0
50
100
150
200
0.01
0.05
0.10
0.50
1.00
5.00
10.00
Treatment Dose (uM)

Folpet

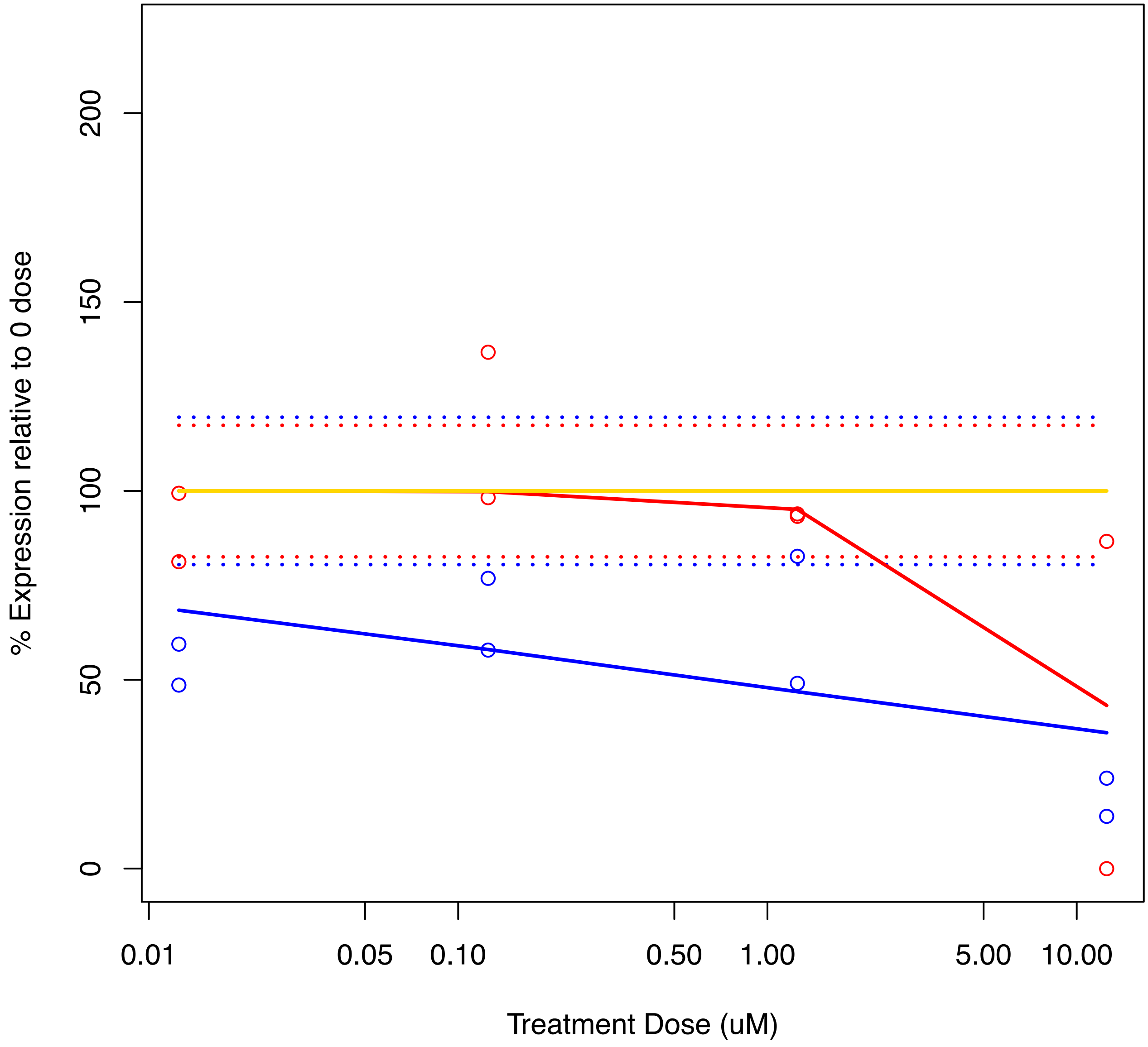

% Expression relative to 0 dose
0
50
100
150
200
0.01
0.05
0.10
0.50
1.00
5.00
10.00
Treatment Dose (uM)

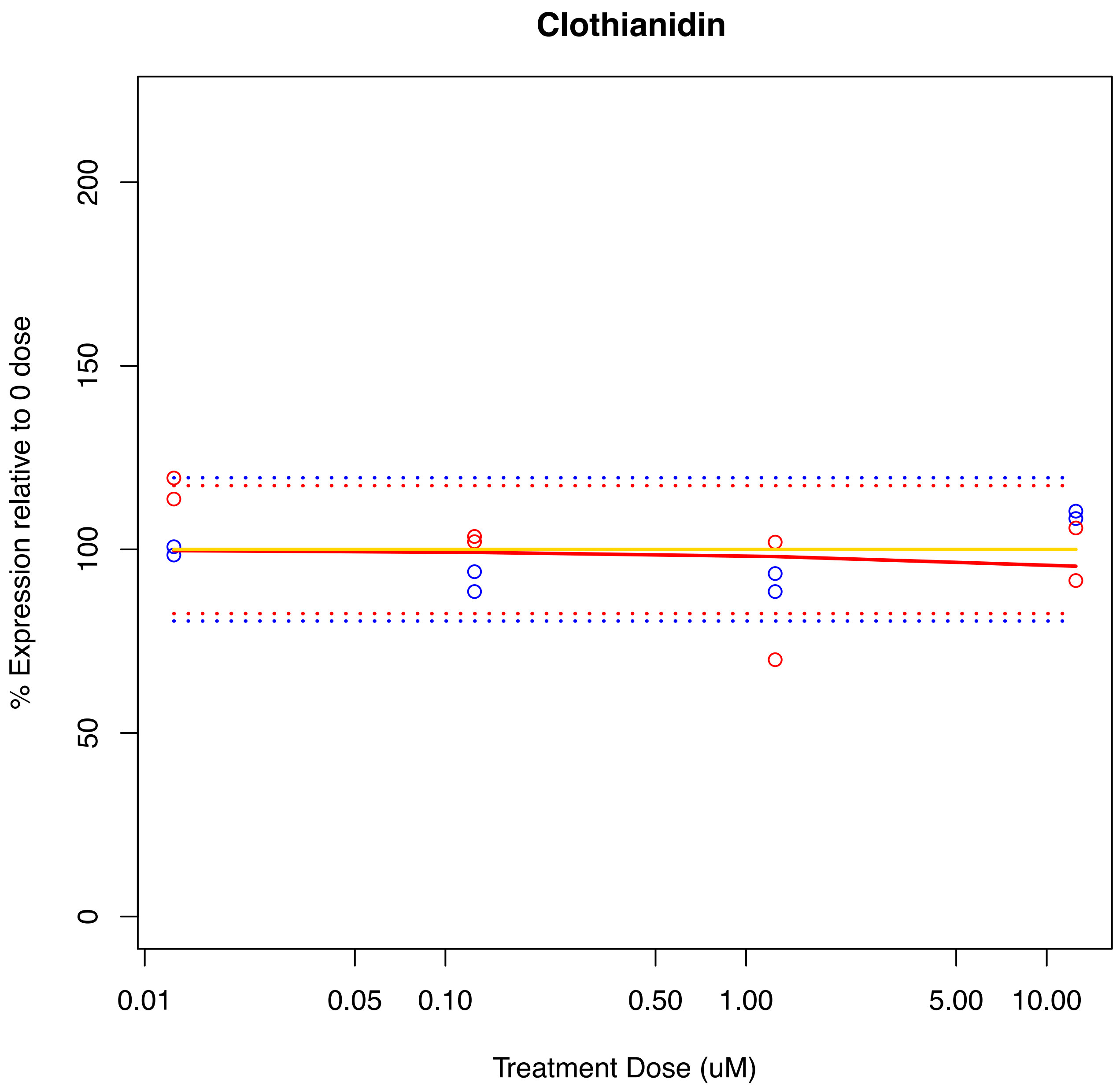
Clothianidin
% Expression relative to 0 dose
0
50
100
150
200
0.01
0.05
0.10
0.50
1.00
5.00
10.00
Treatment Dose (uM)

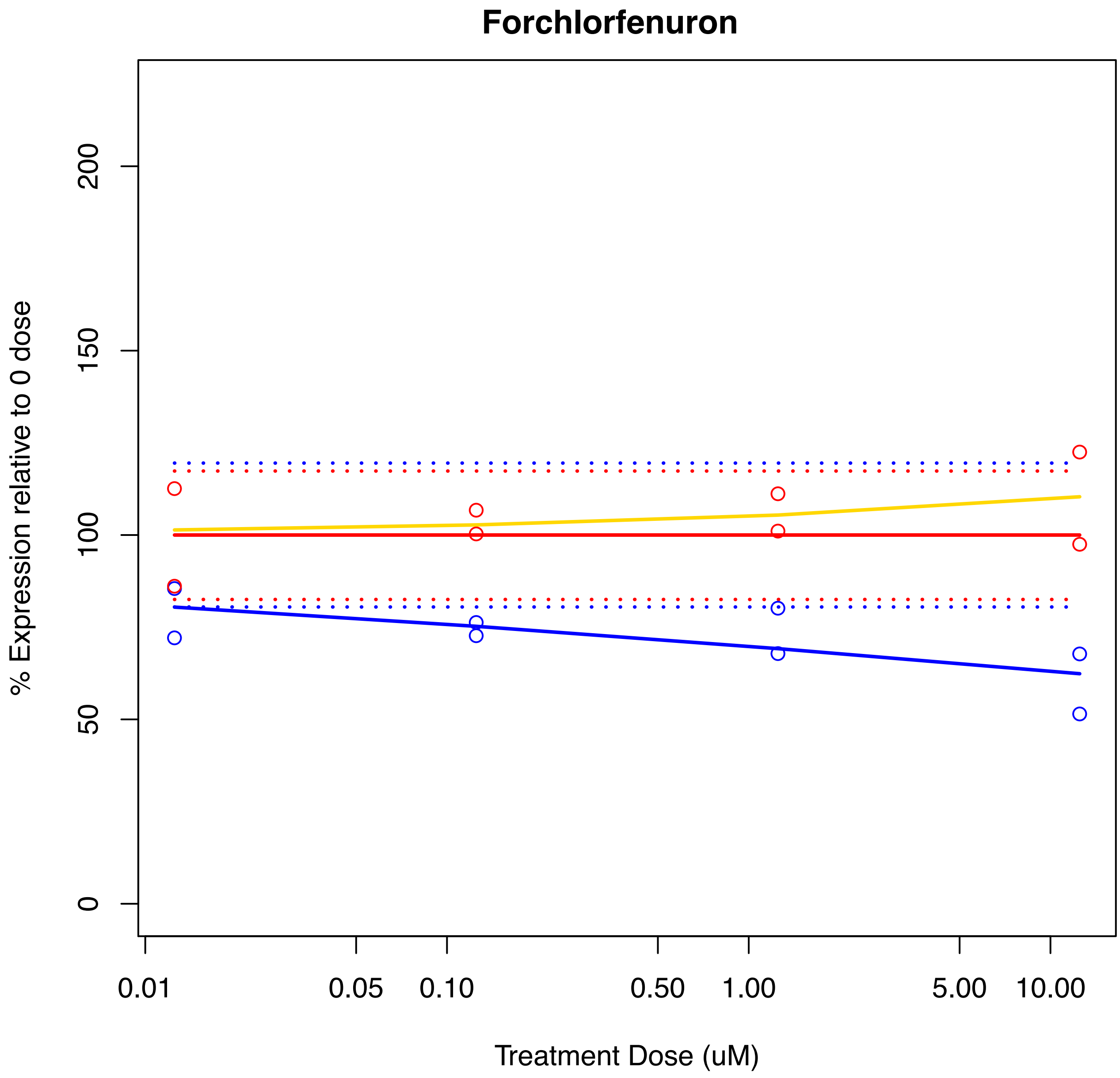

Forchlorfenuron
% Expression relative to 0 dose
0
50
100
150
200
0.01
0.05
0.10
0.50
1.00
5.00
10.00
Treatment Dose (uM)

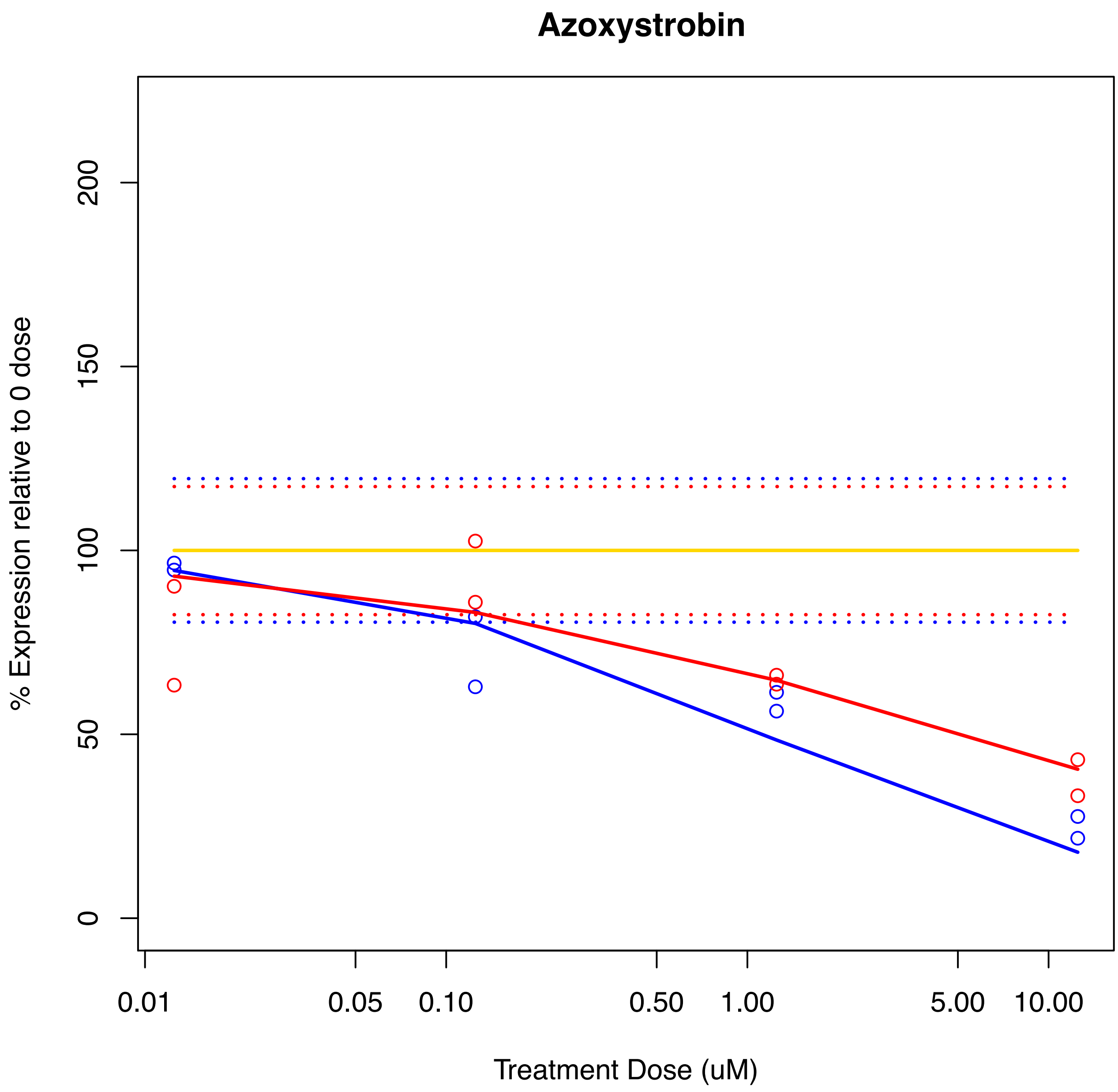
Azoxystrobin
% Expression relative to 0 dose
0
50
100
150
200
0.01
0.05
0.10
0.50
1.00
5.00
10.00
Treatment Dose (uM)

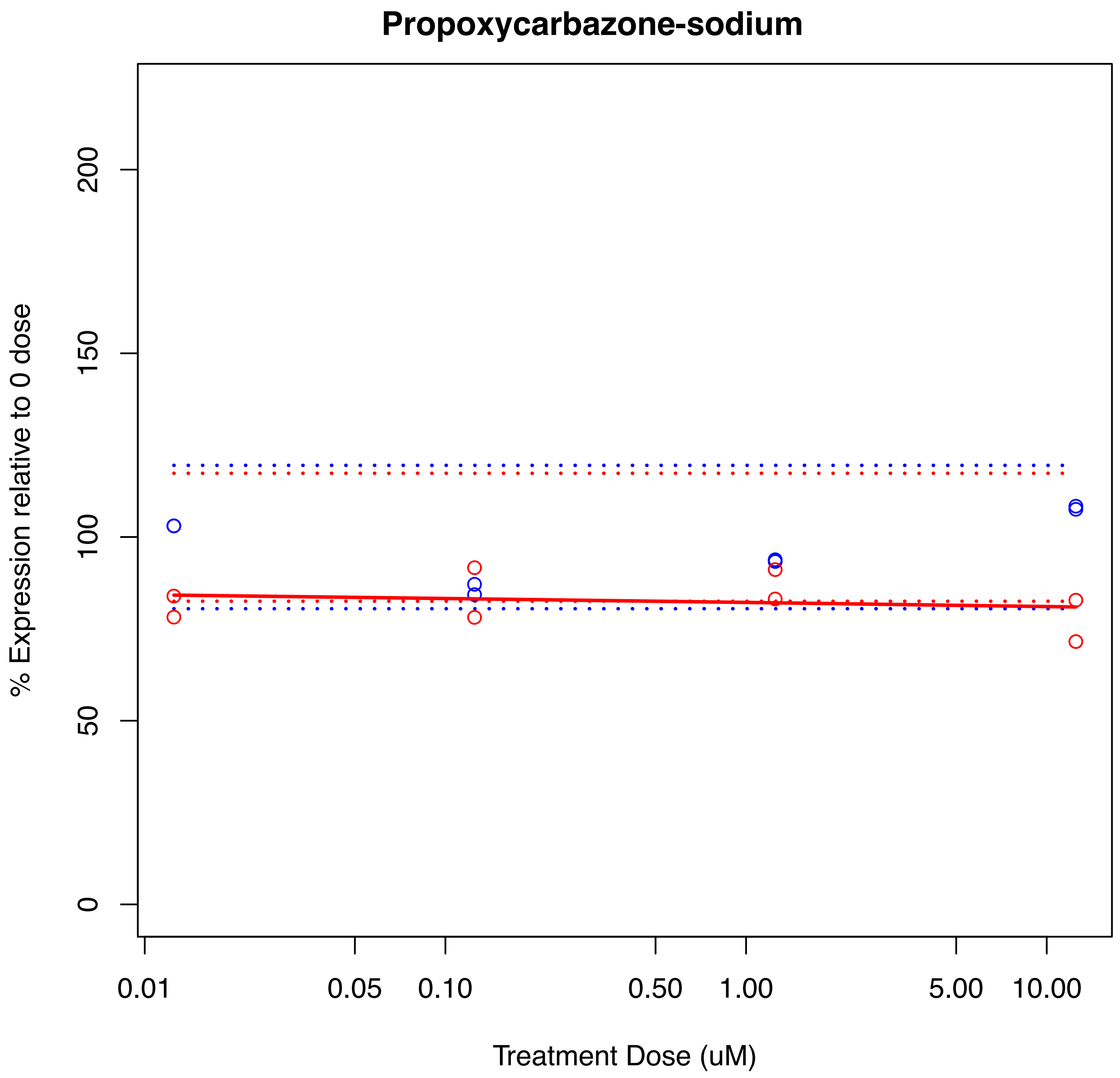

Propoxycarbazone-sodium
% Expression relative to 0 dose
0
50
100
150
200
0.01
0.05
0.10
0.50
1.00
5.00
10.00
Treatment Dose (uM)

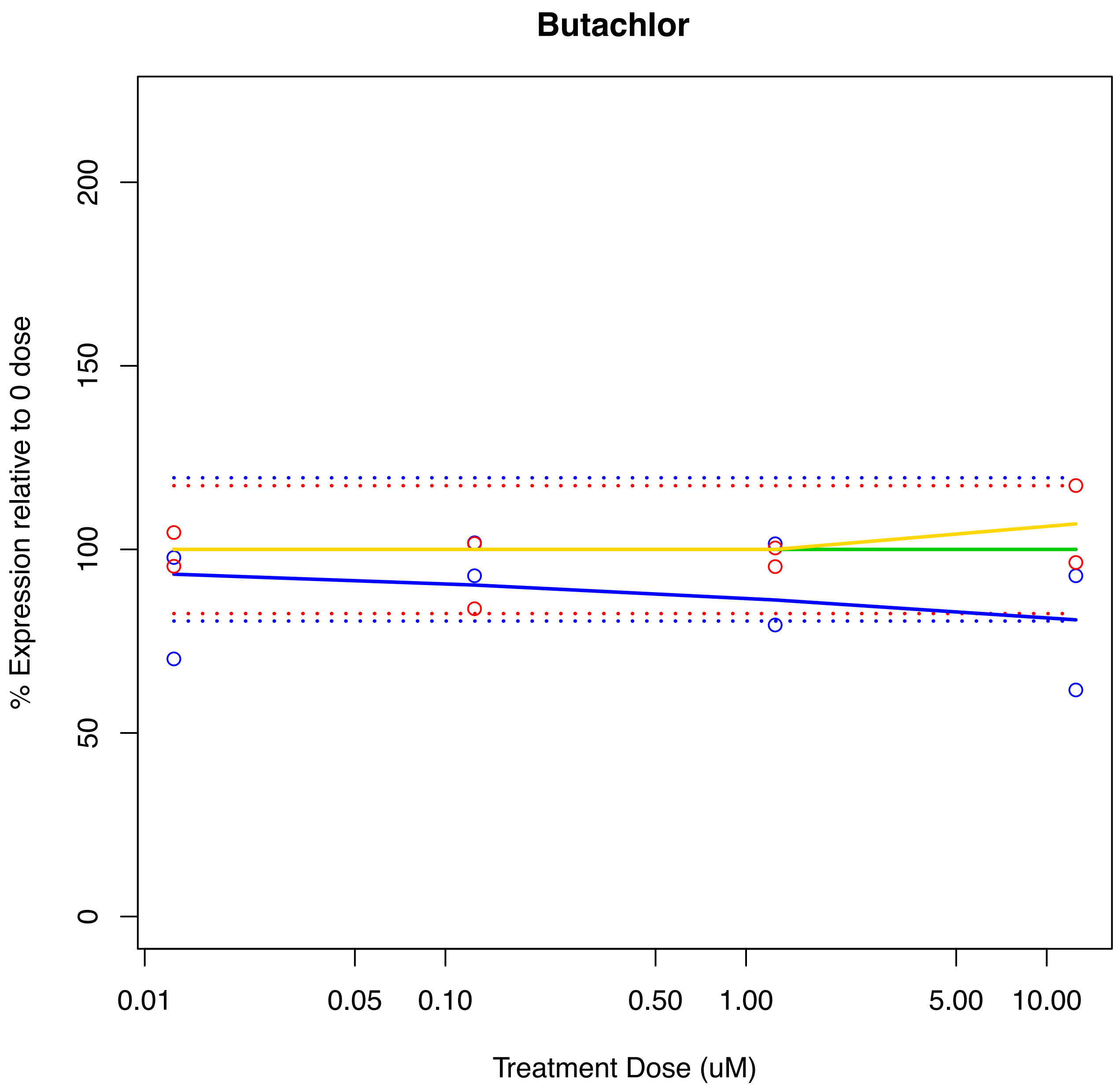
Butachlor
% Expression relative to 0 dose
0
50
100
150
200
0.01
0.05
0.10
0.50
1.00
5.00
10.00
Treatment Dose (uM)

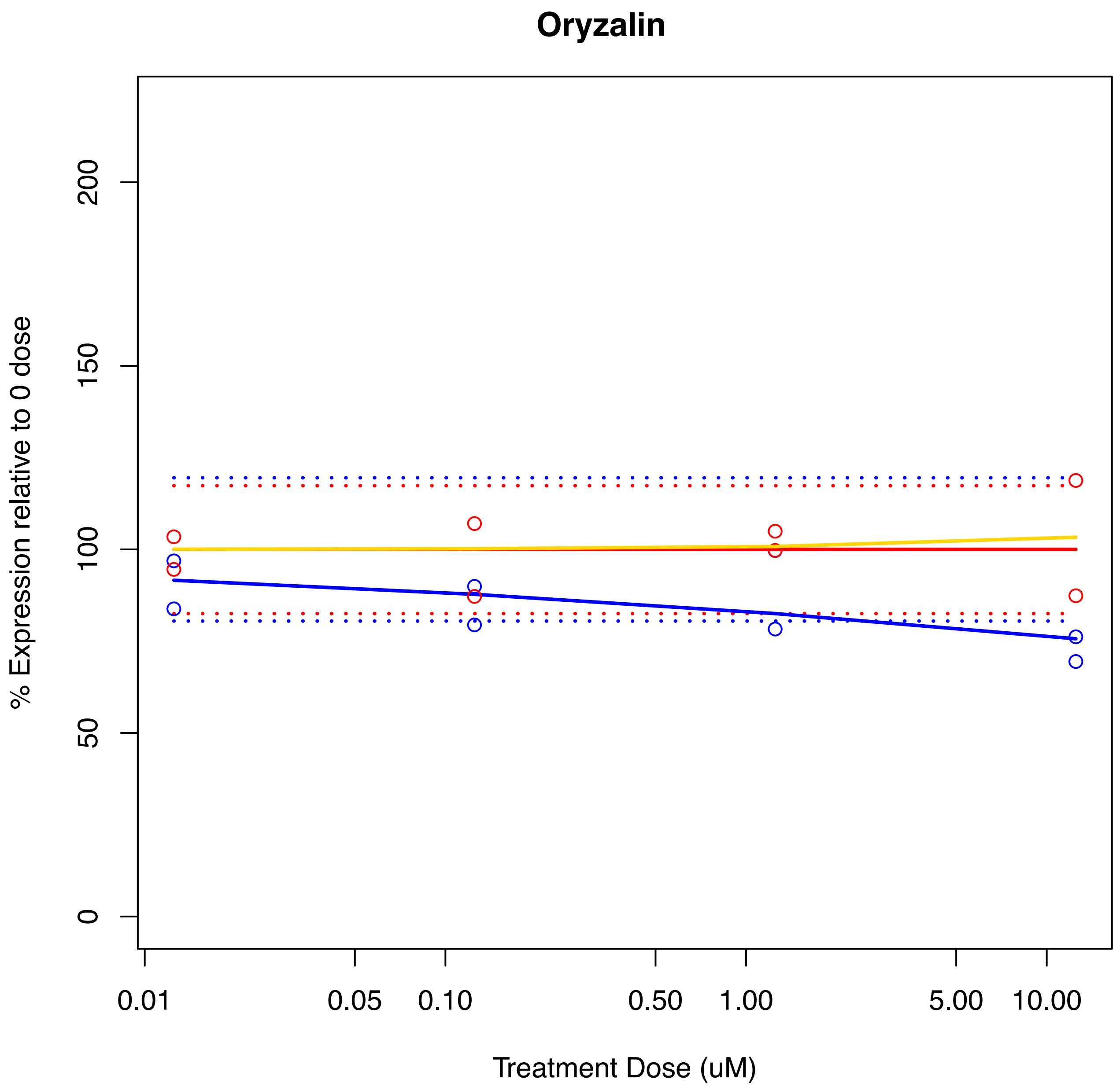

Oryzalin
% Expression relative to 0 dose
0
50
100
150
200
0.01
0.05
0.10
0.50
1.00
5.00
10.00
Treatment Dose (uM)

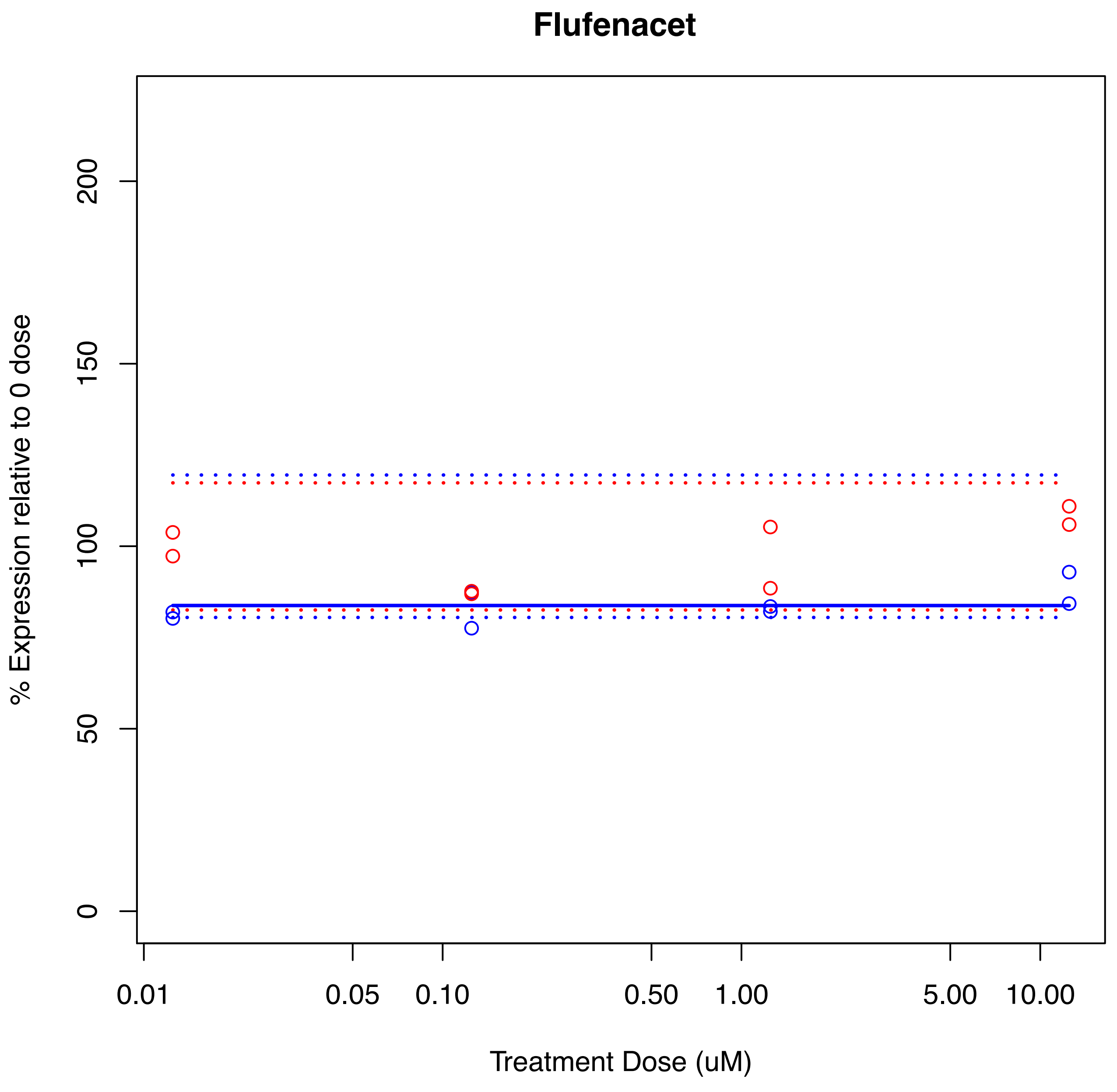

Flufenacet
% Expression relative to 0 dose
0
50
100
150
200
0.01
0.05
0.10
0.50
1.00
5.00
10.00
Treatment Dose (uM)

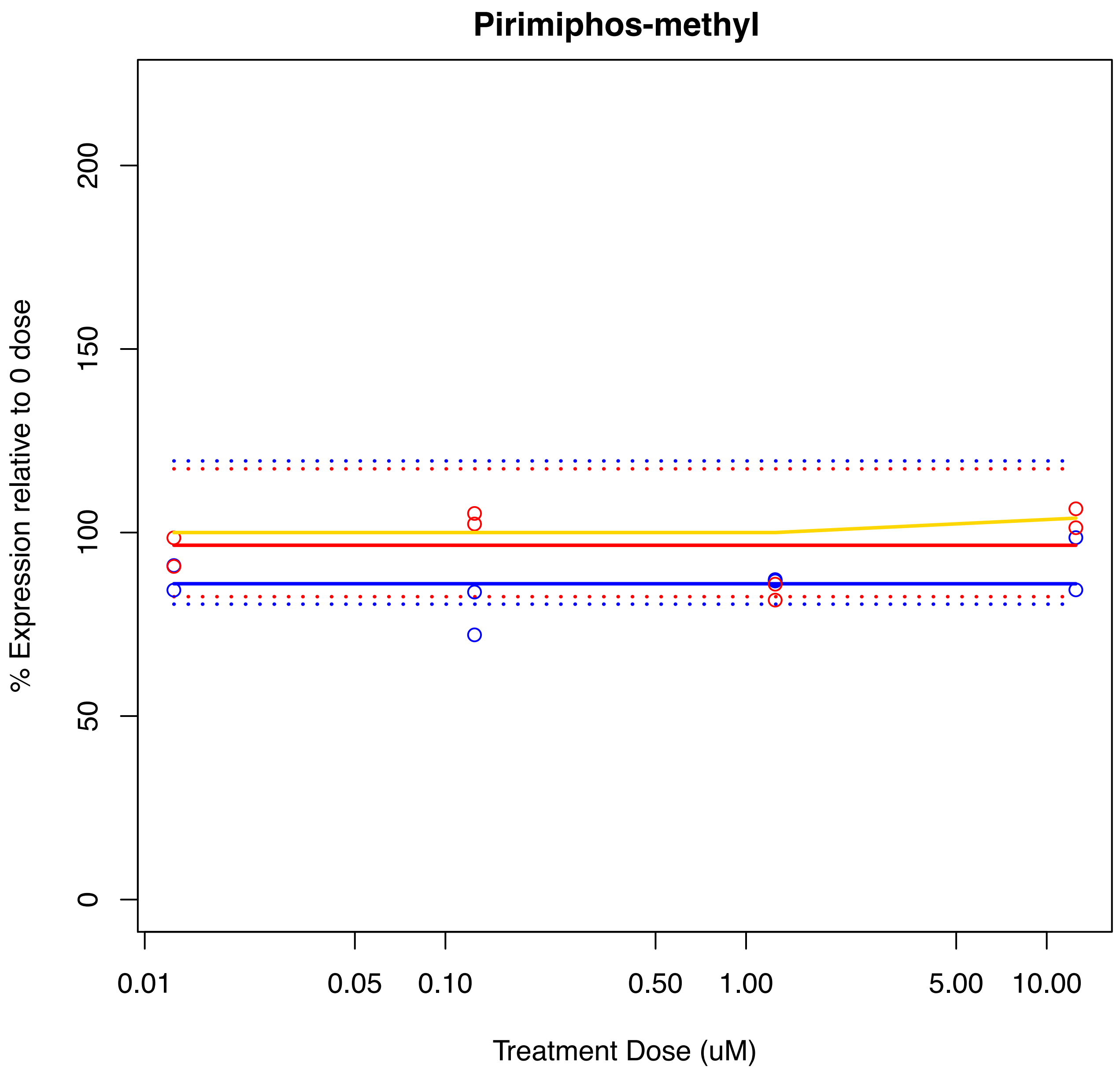

Pirimiphos-methyl
% Expression relative to 0 dose
0
50
100
150
200
0.01
0.05
0.10
0.50
1.00
5.00
10.00
Treatment Dose (uM)

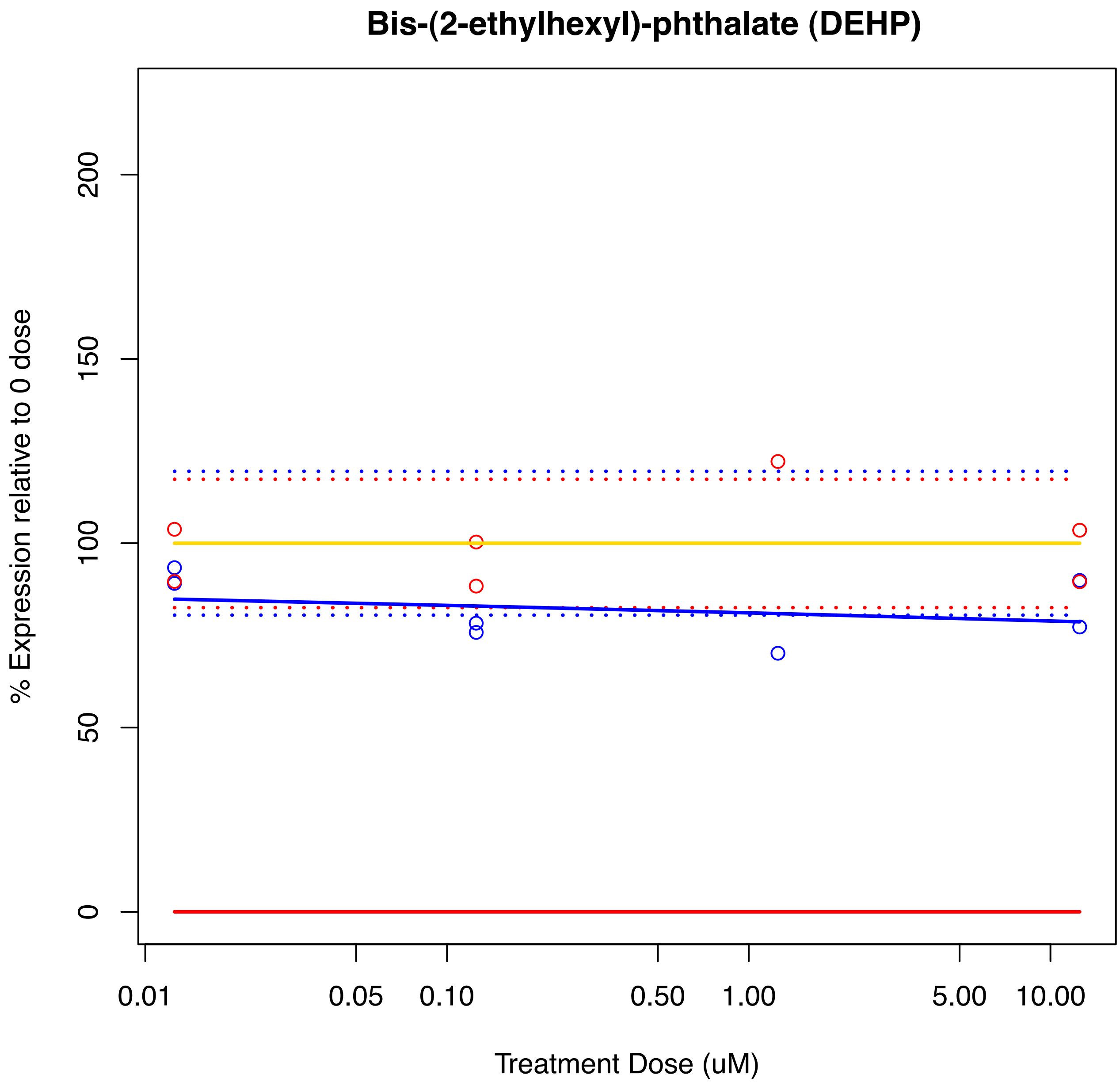
Bis-(2-ethylhexyl)-phthalate (DEHP)
% Expression relative to 0 dose
0
50
100
150
200
0.01
0.05
0.10
0.50
1.00
5.00
10.00
Treatment Dose (uM)

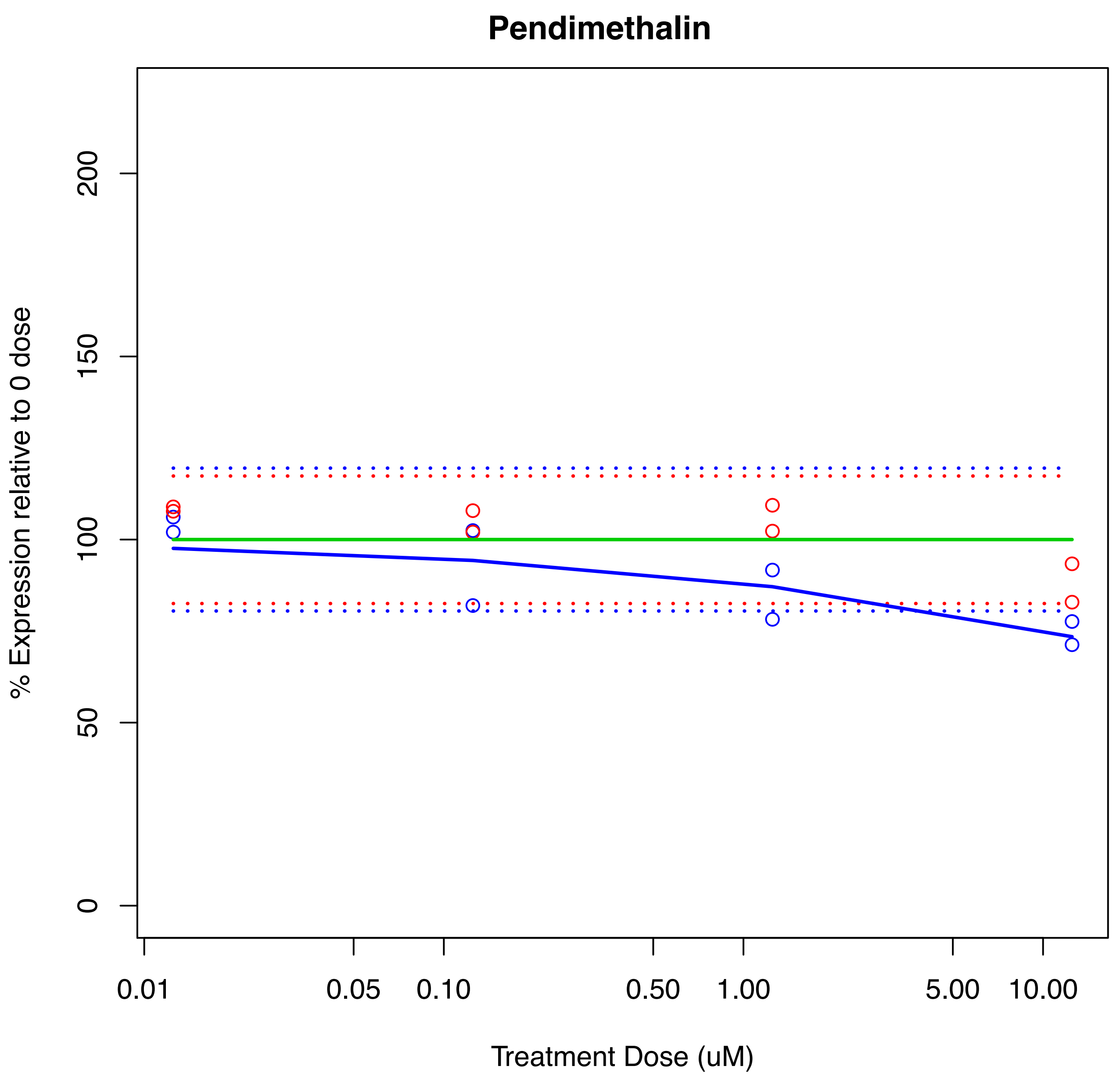
Pendimethalin
% Expression relative to 0 dose
0
50
100
150
200
0.01
0.05
0.10
0.50
1.00
5.00
10.00
Treatment Dose (uM)

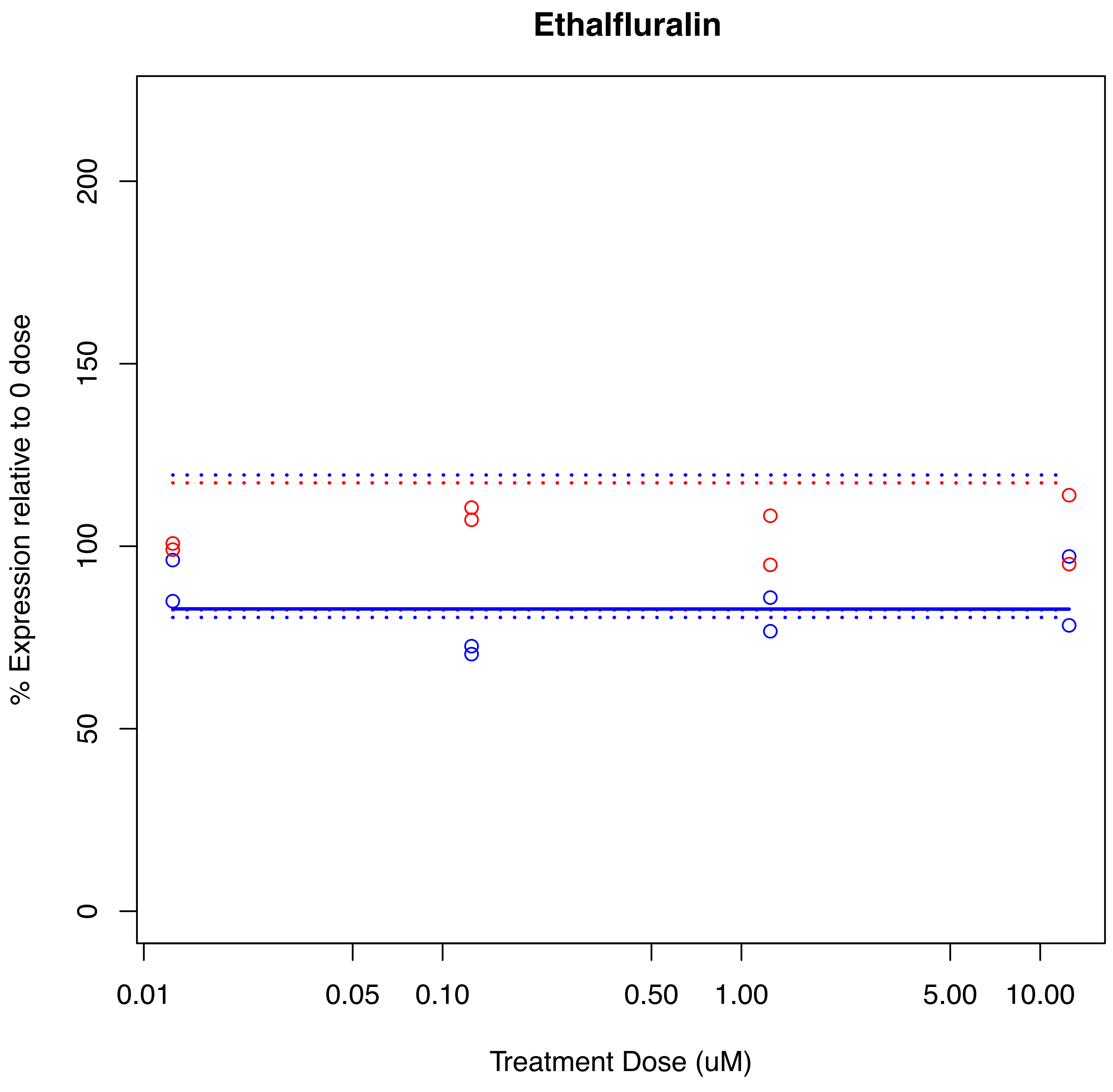

Ethalfluralin
% Expression relative to 0 dose
0
50
100
150
200
0.01
0.05
0.10
0.50
1.00
5.00
10.00
Treatment Dose (uM)

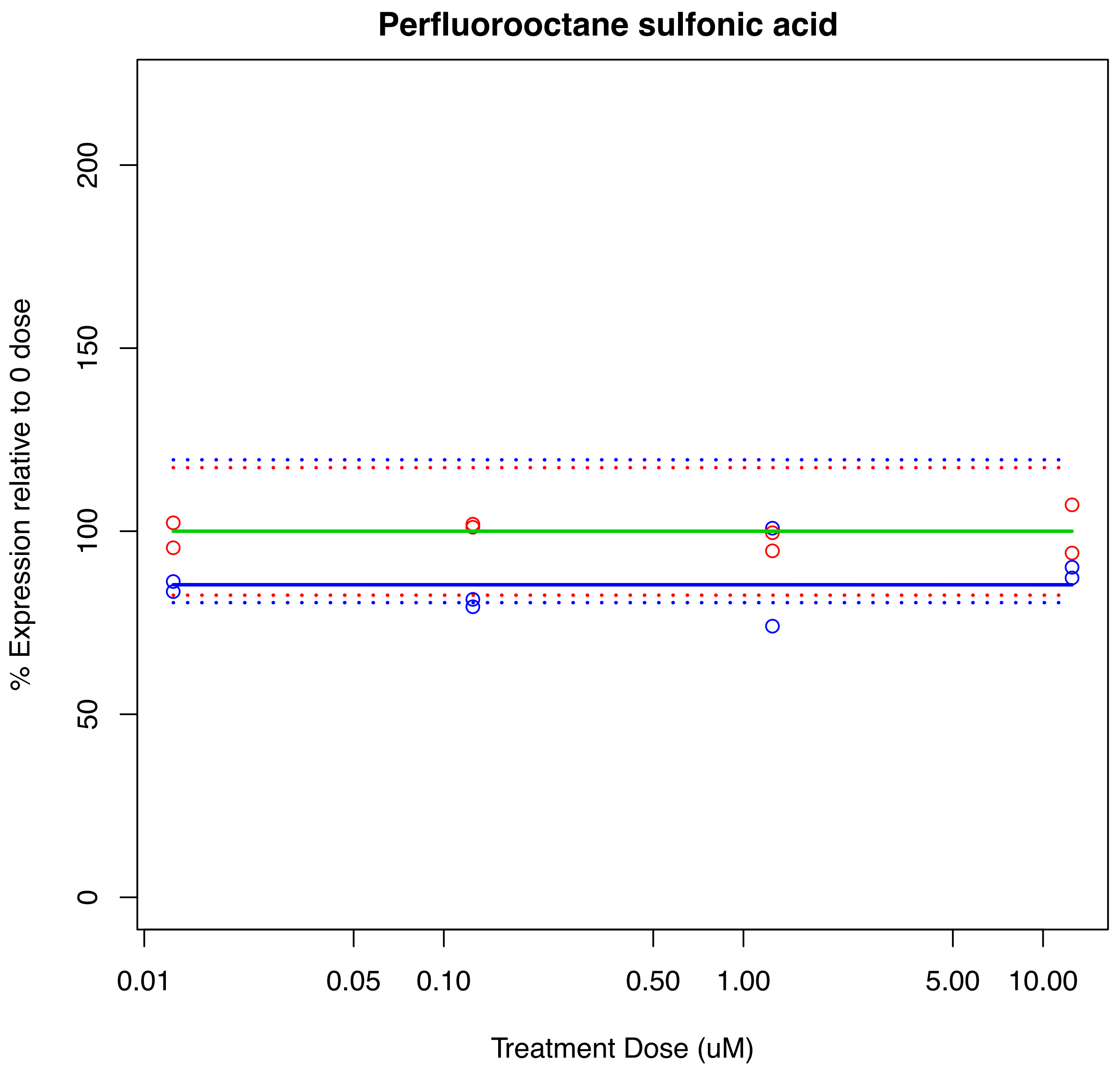

Perfluorooctane sulfonic acid
% Expression relative to 0 dose
0
50
100
150
200
0.01
0.05
0.10
0.50
1.00
5.00
10.00
Treatment Dose (uM)

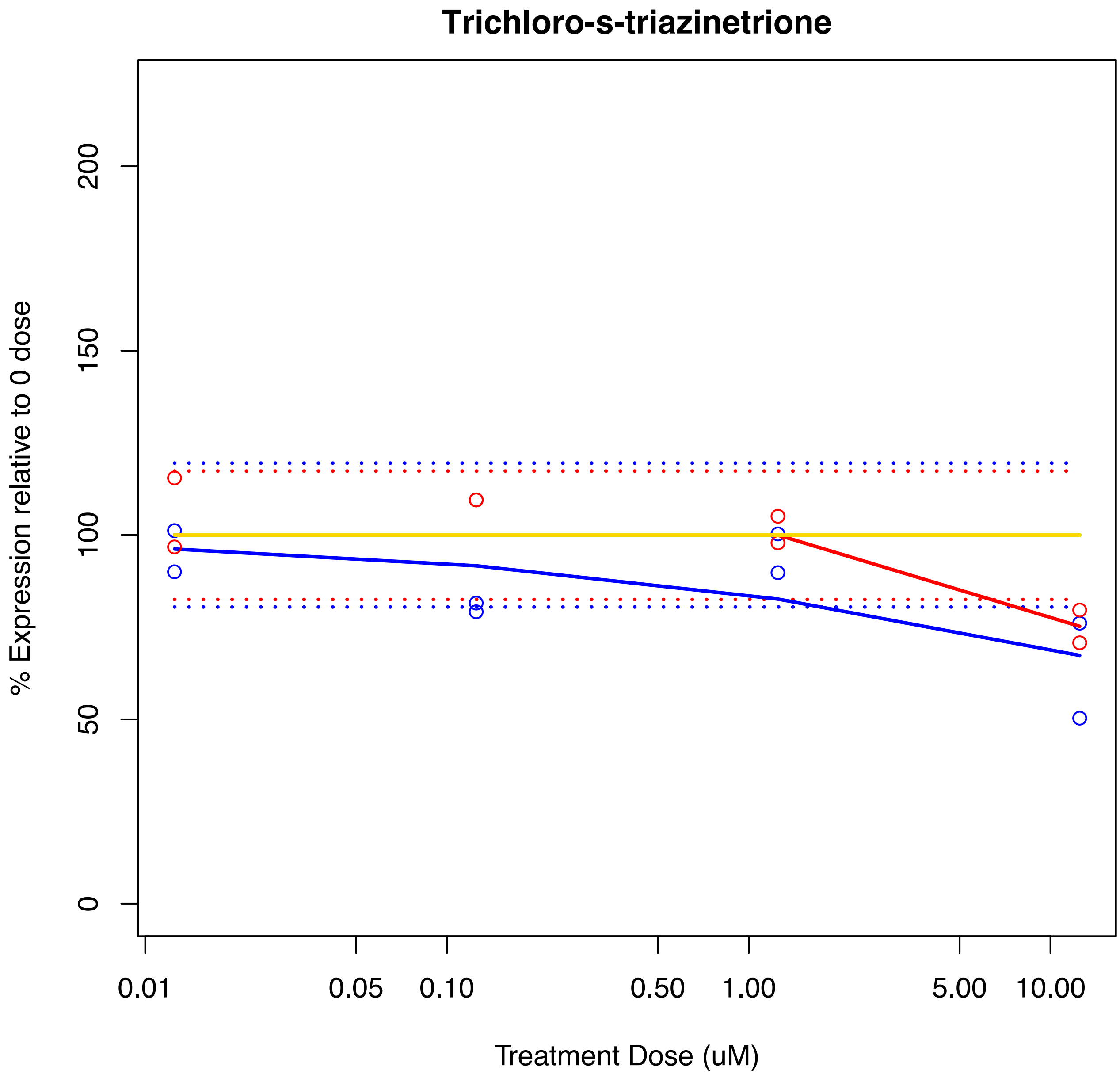
Trichloro-s-triazinetrione
% Expression relative to 0 dose
0
50
100
150
200
0.01
0.05
0.10
0.50
1.00
5.00
10.00
Treatment Dose (uM)

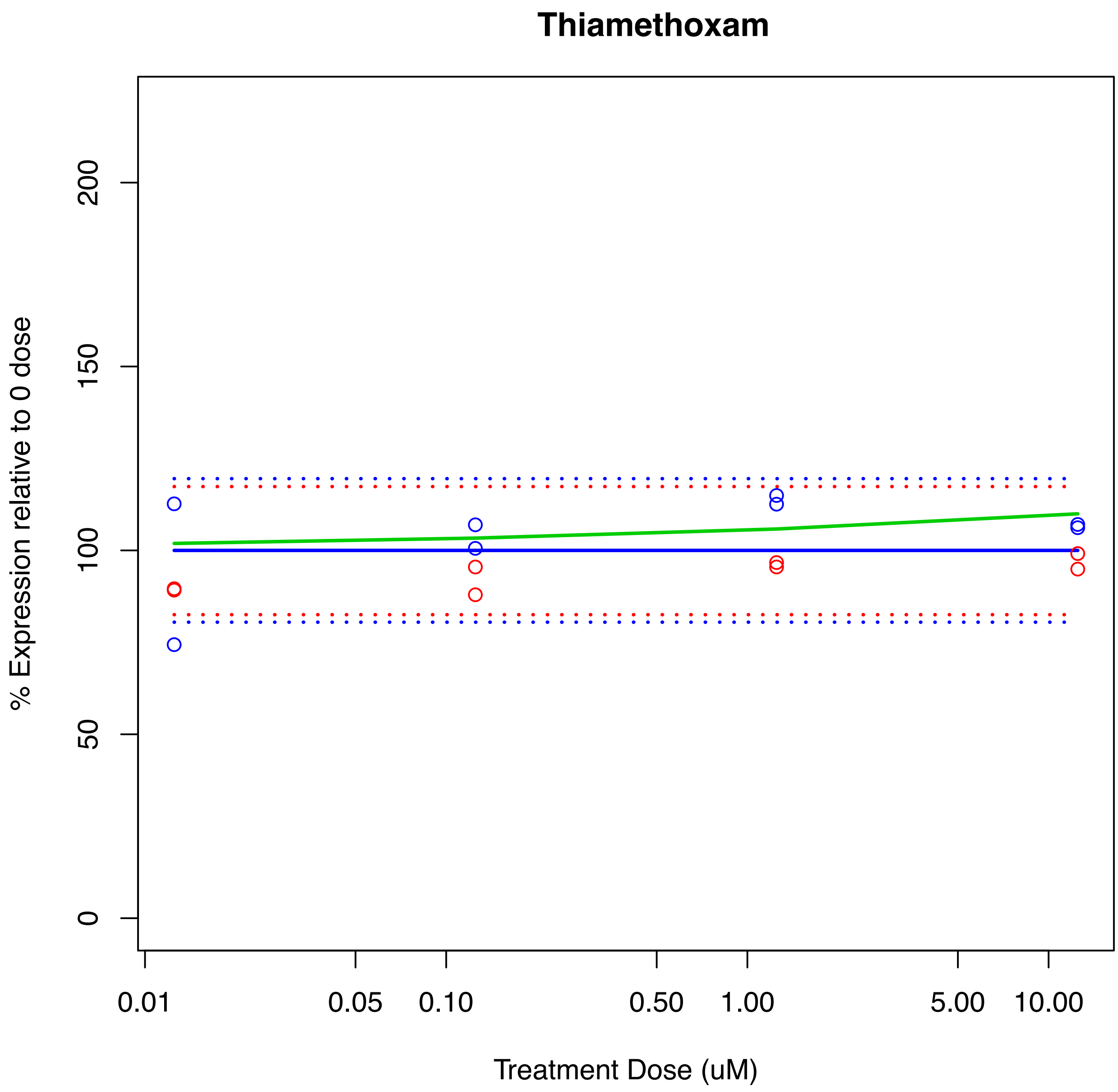
Thiamethoxam
% Expression relative to 0 dose
0
50
100
150
200
0.01
0.05
0.10
0.50
1.00
5.00
10.00
Treatment Dose (uM)

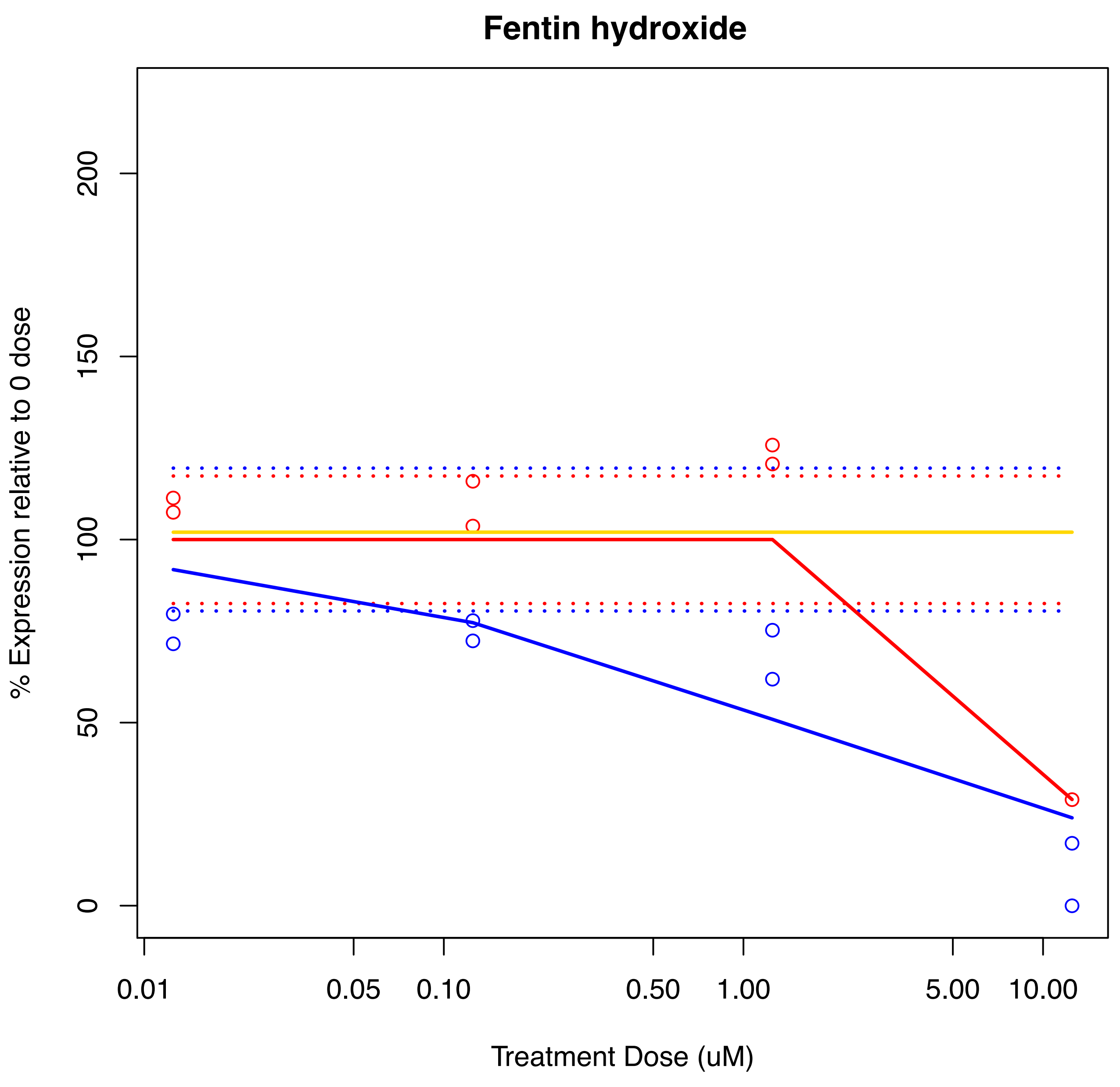

Fentin hydroxide
% Expression relative to 0 dose
Treatment Dose (uM)
0
50
100
150
200
0.01
0.05
0.10
0.50
1.00
5.00
10.00

2-Pyridinesulfonamide, N-[[(4,6-dimethoxy-2-pyrimidinyl)amino]carbonyl]-3-(2,2,2-trifluoroethoxy)-, monosodium salt, monohydrate

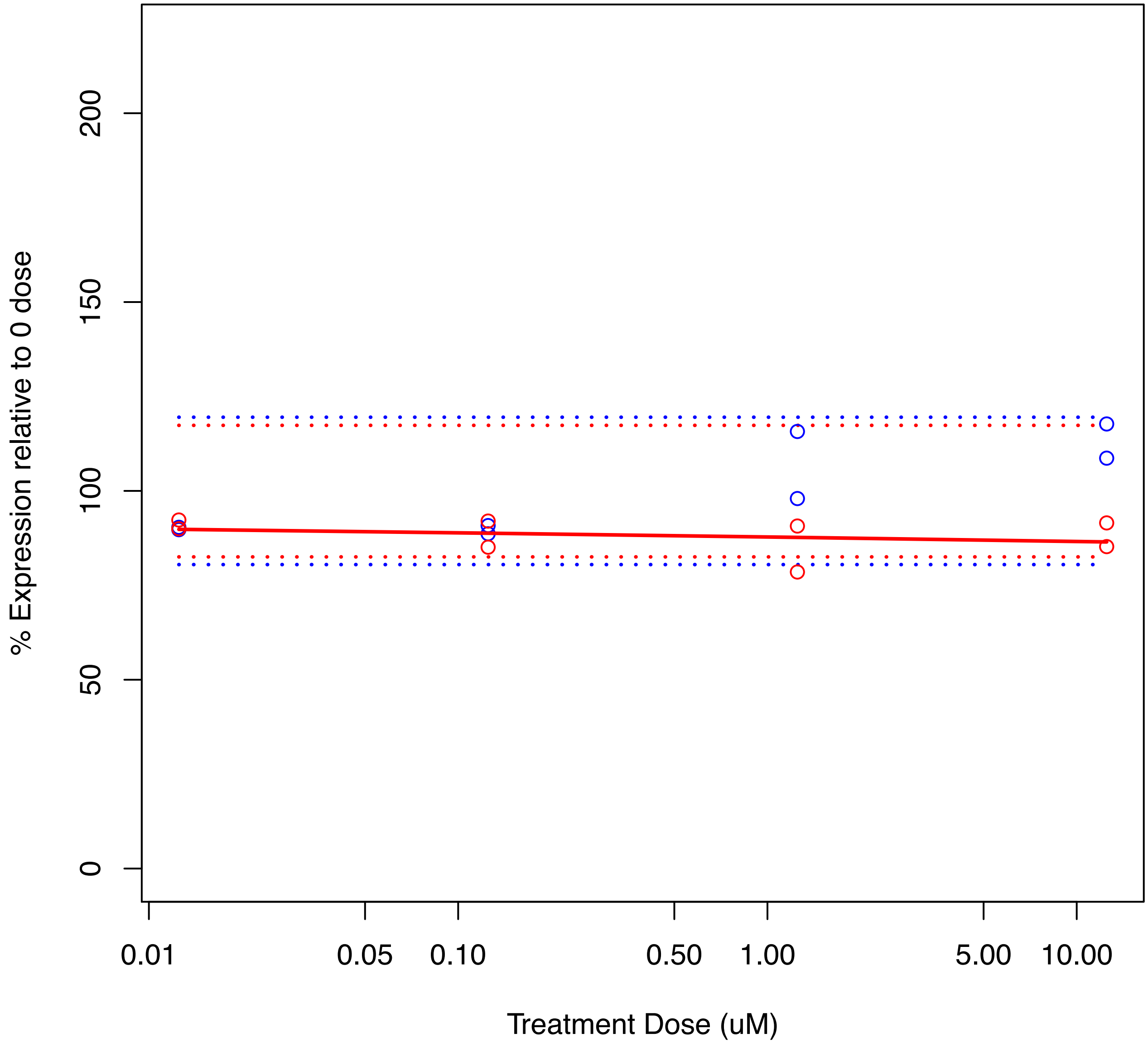

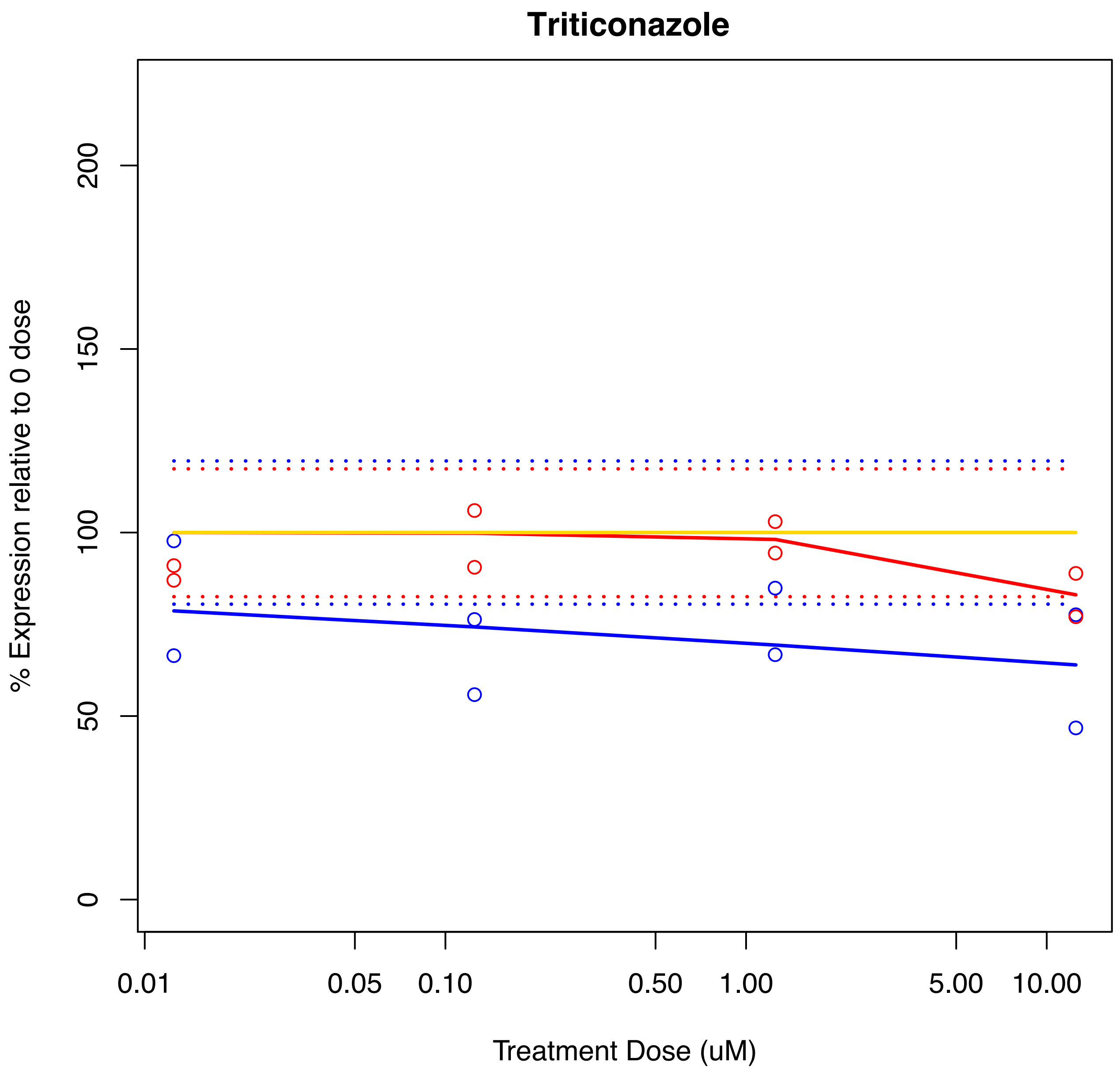
Triticonazole
% Expression relative to 0 dose
0
50
100
150
200
0.01
0.05
0.10
0.50
1.00
5.00
10.00
Treatment Dose (uM)

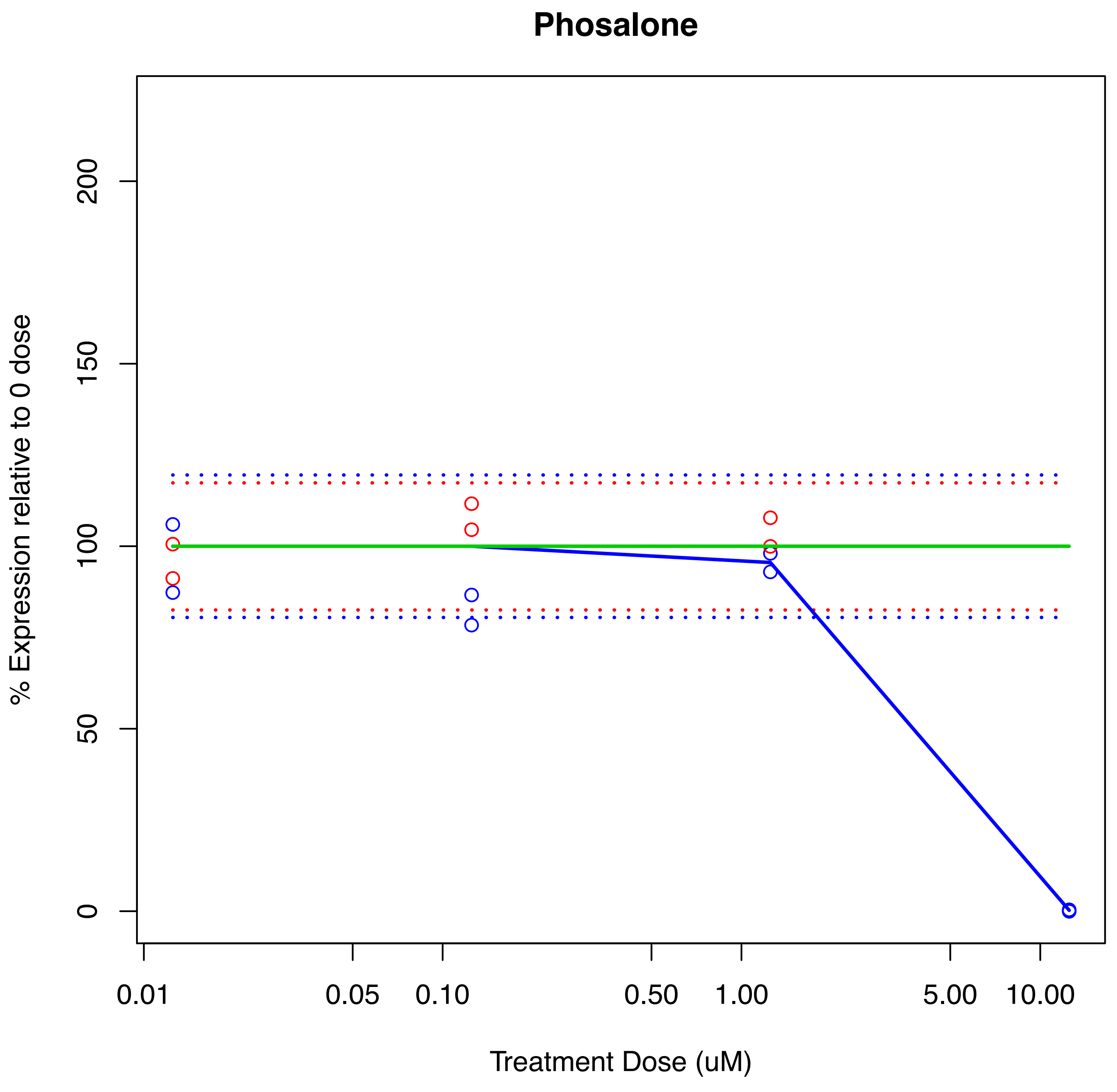
Phosalone
% Expression relative to 0 dose
0
50
100
150
200
0.01
0.05
0.10
0.50
1.00
5.00
10.00
Treatment Dose (uM)

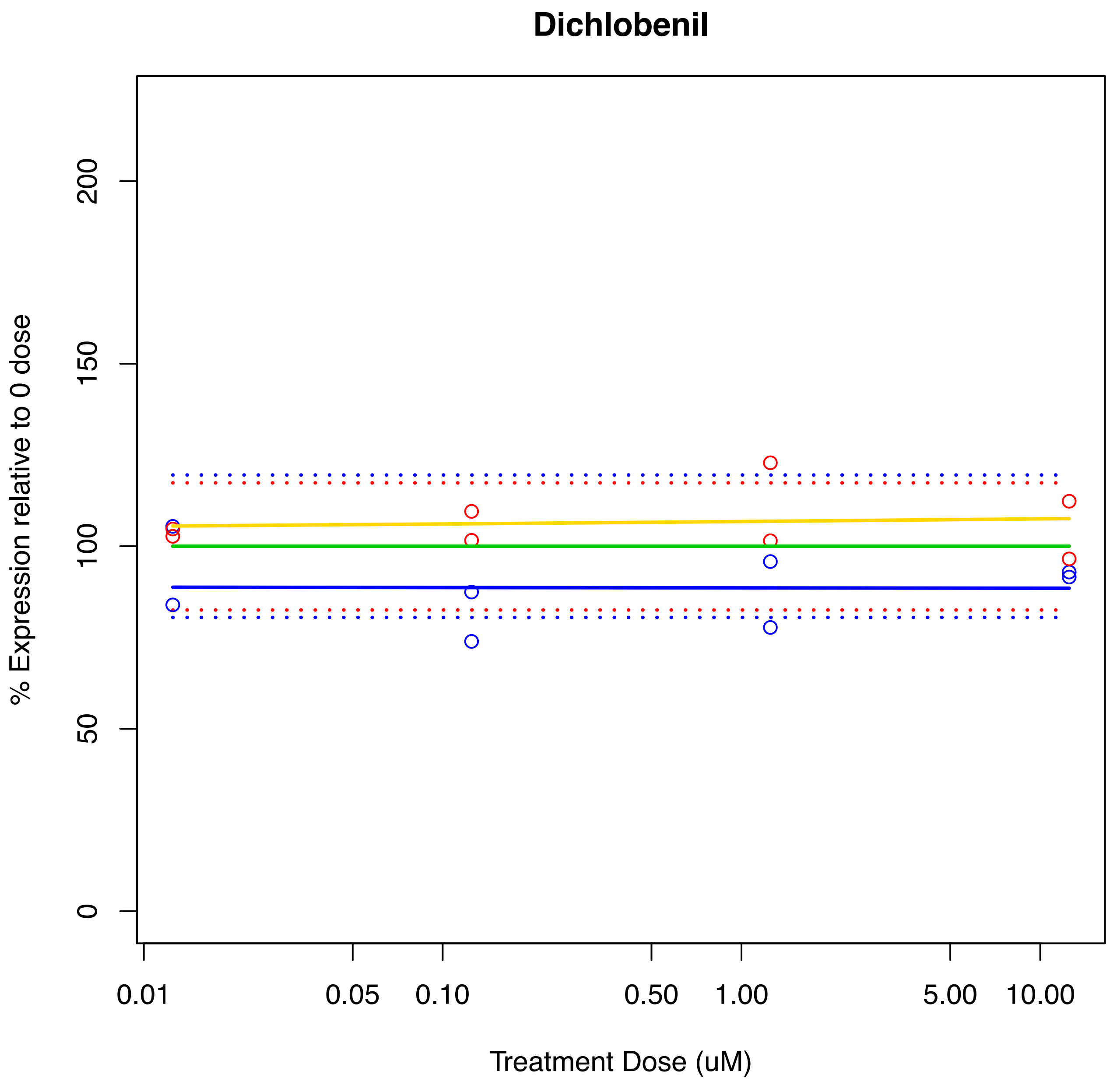

Dichlobenil
% Expression relative to 0 dose
0
50
100
150
200
0.01
0.05
0.10
0.50
1.00
5.00
10.00
Treatment Dose (uM)

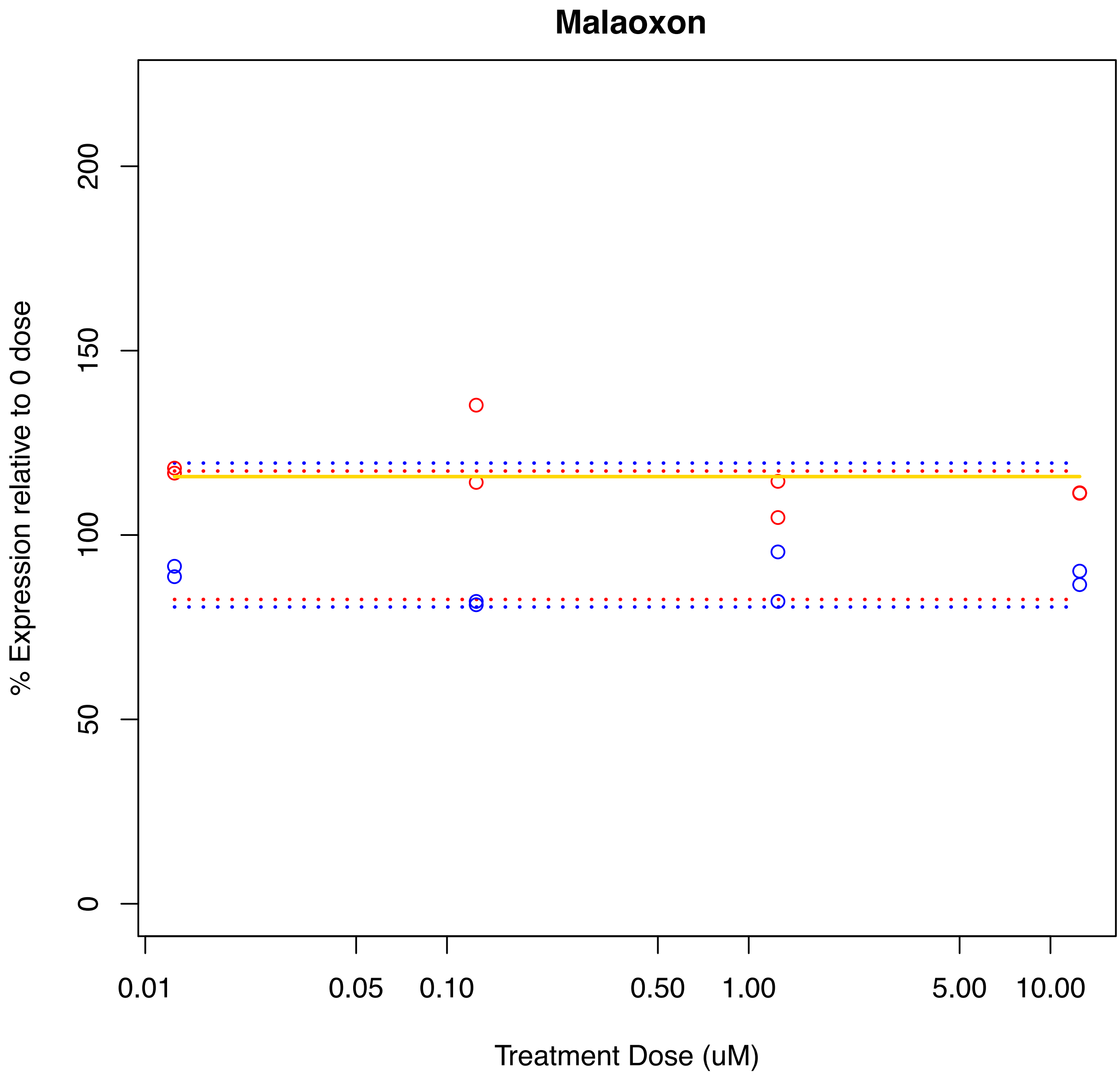

Malaoxon
% Expression relative to 0 dose
0
50
100
150
200
0.01
0.05
0.10
0.50
1.00
5.00
10.00
Treatment Dose (uM)

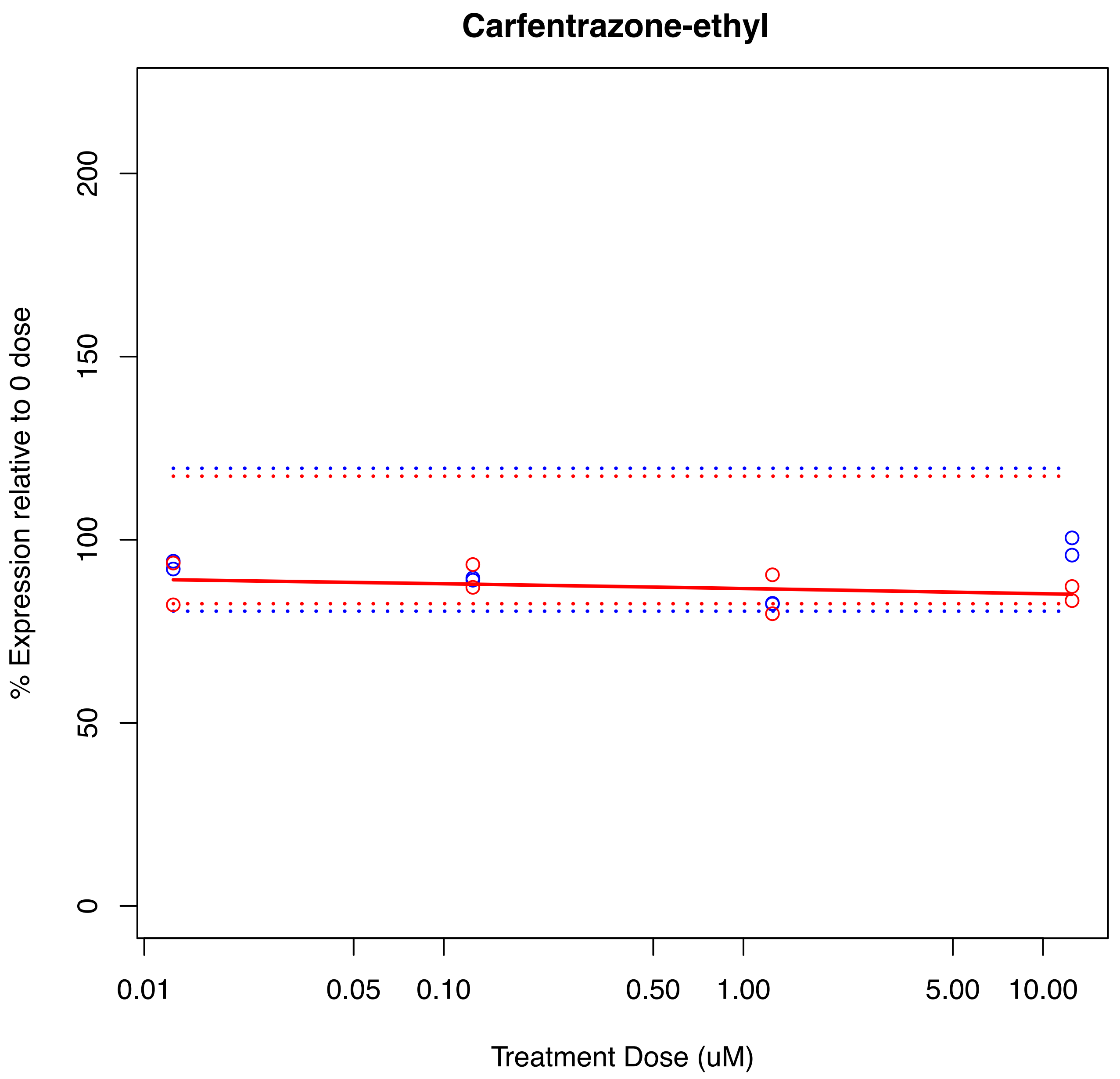
Carfentrazone-ethyl
% Expression relative to 0 dose
0
50
100
150
200
0.01
0.05
0.10
0.50
1.00
5.00
10.00
Treatment Dose (uM)

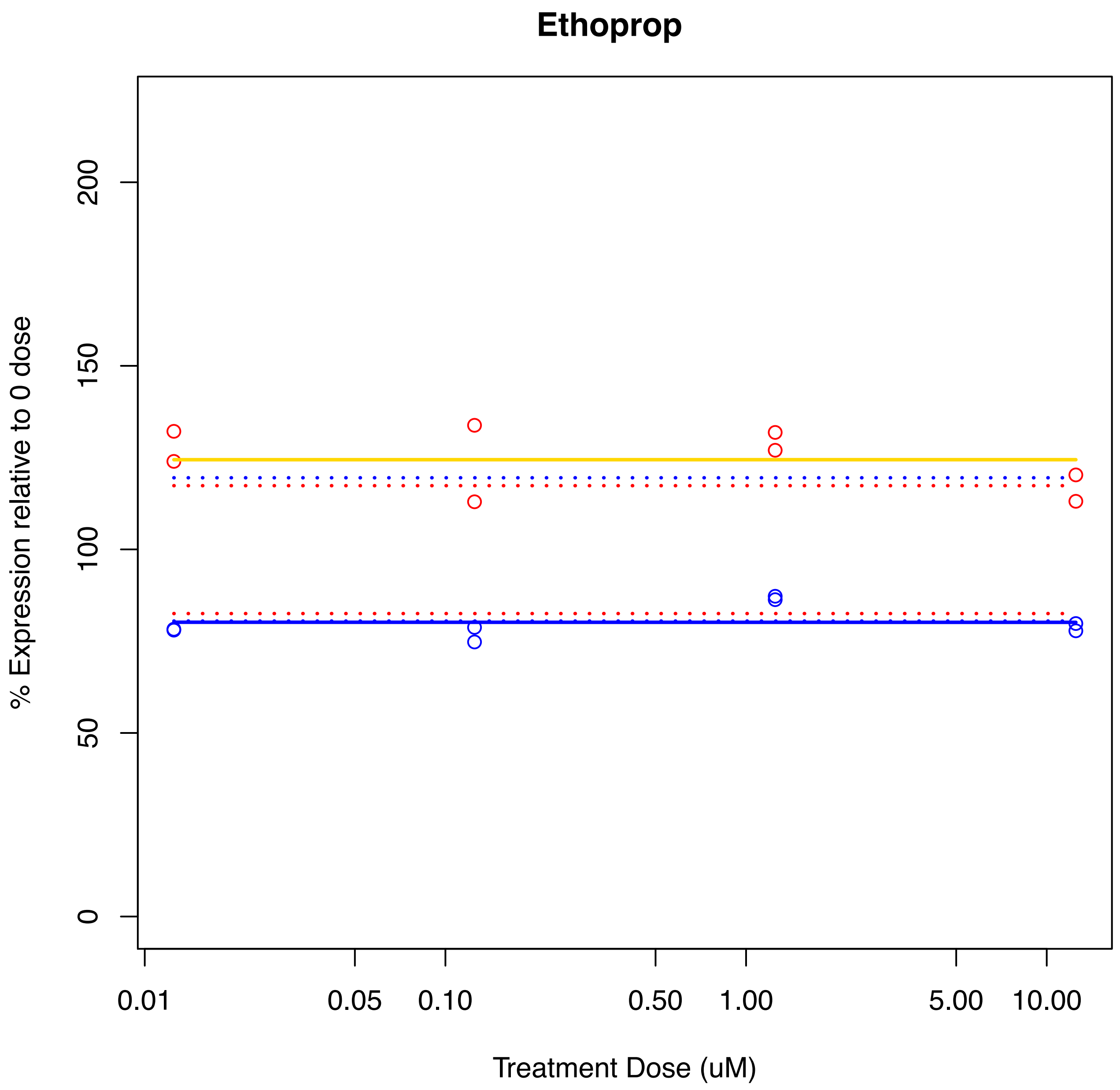

Ethoprop
% Expression relative to 0 dose
0
50
100
150
200
0.01
0.05
0.10
0.50
1.00
5.00
10.00
Treatment Dose (uM)

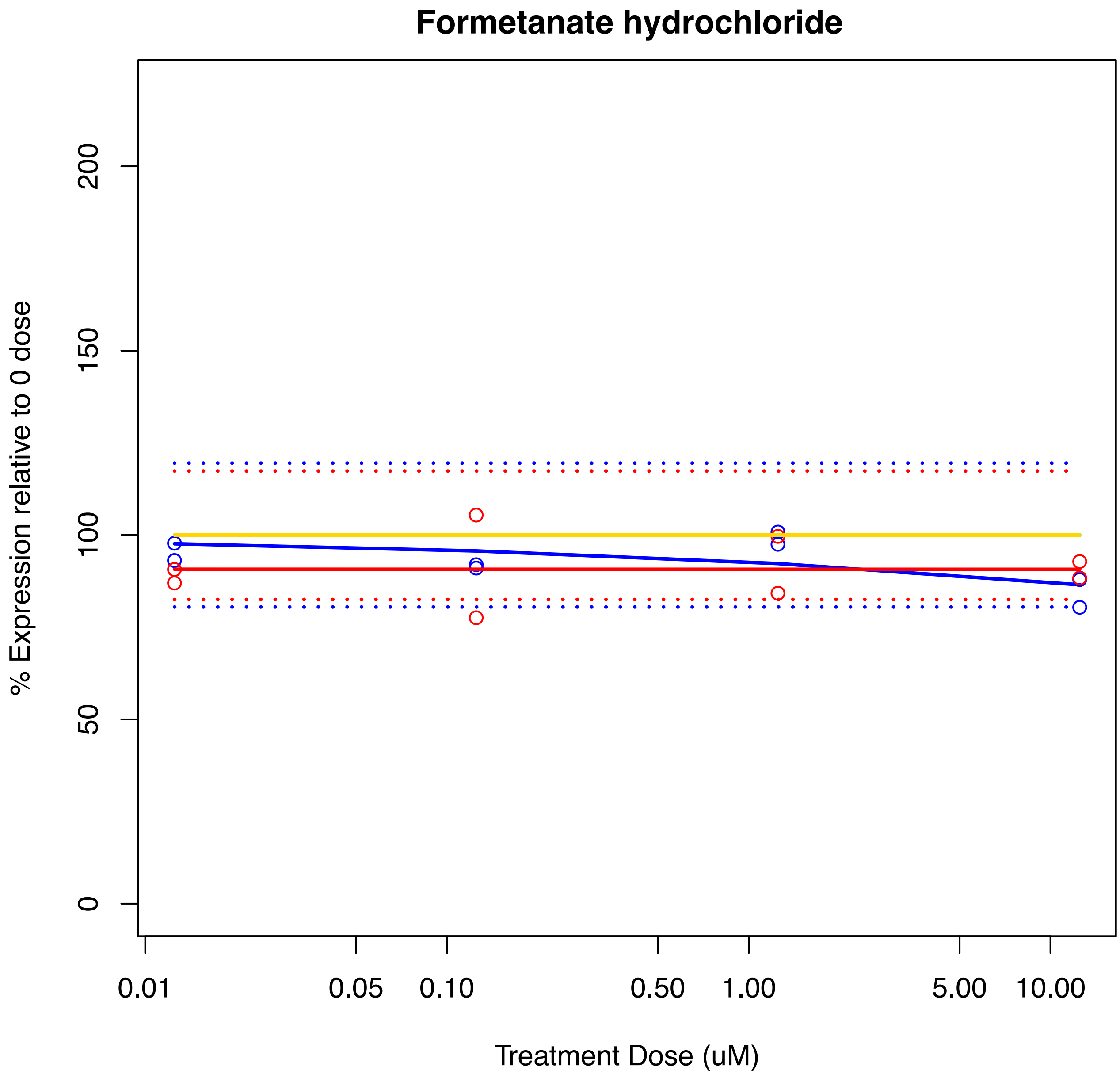

Formetanate hydrochloride
% Expression relative to 0 dose
0
50
100
150
200
0.01
0.05
0.10
0.50
1.00
5.00
10.00
Treatment Dose (uM)

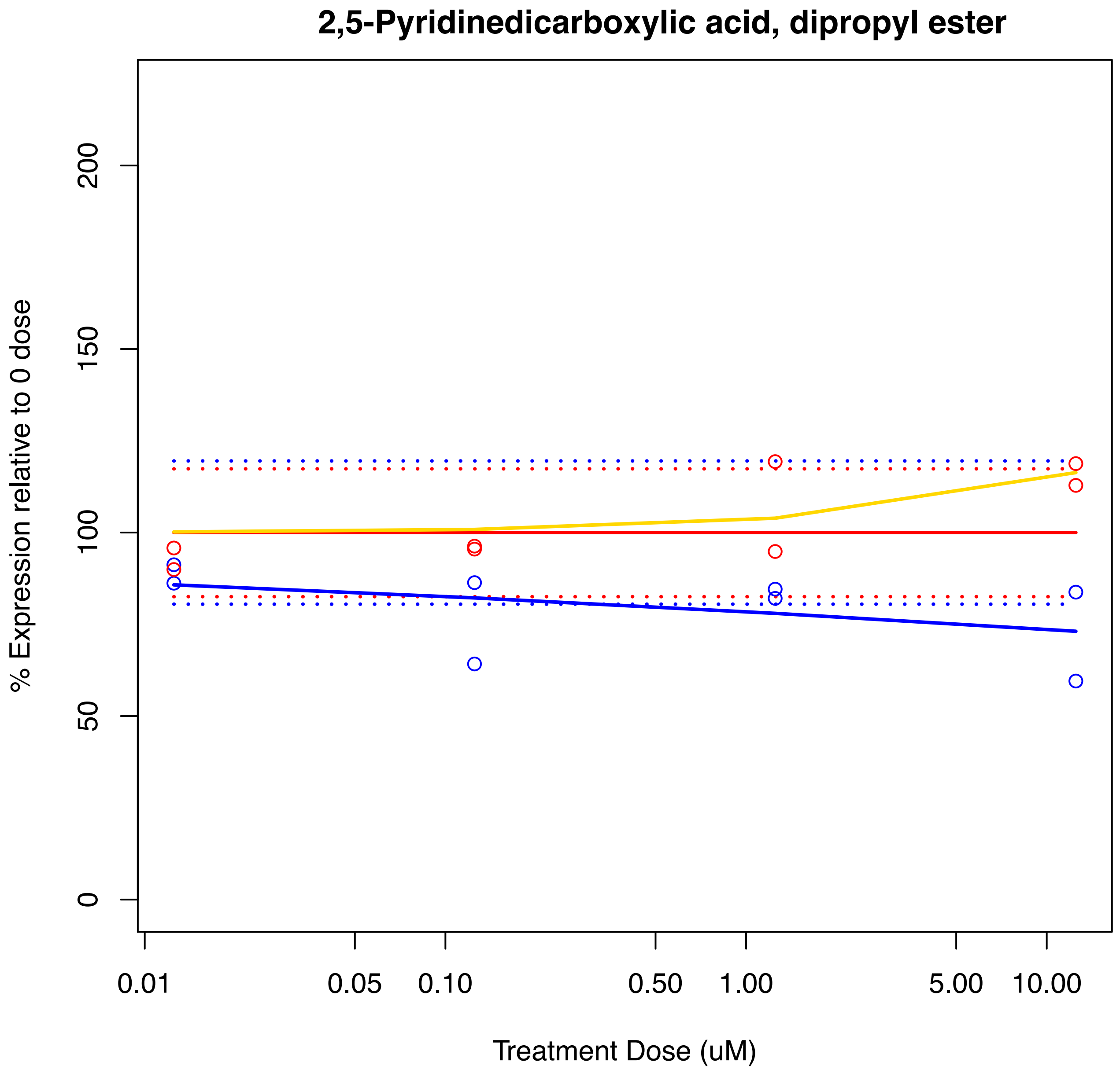

2,5-Pyridinedicarboxylic acid, dipropyl ester
% Expression relative to 0 dose
0
50
100
150
200
0.01
0.05
0.10
0.50
1.00
5.00
10.00
Treatment Dose (uM)

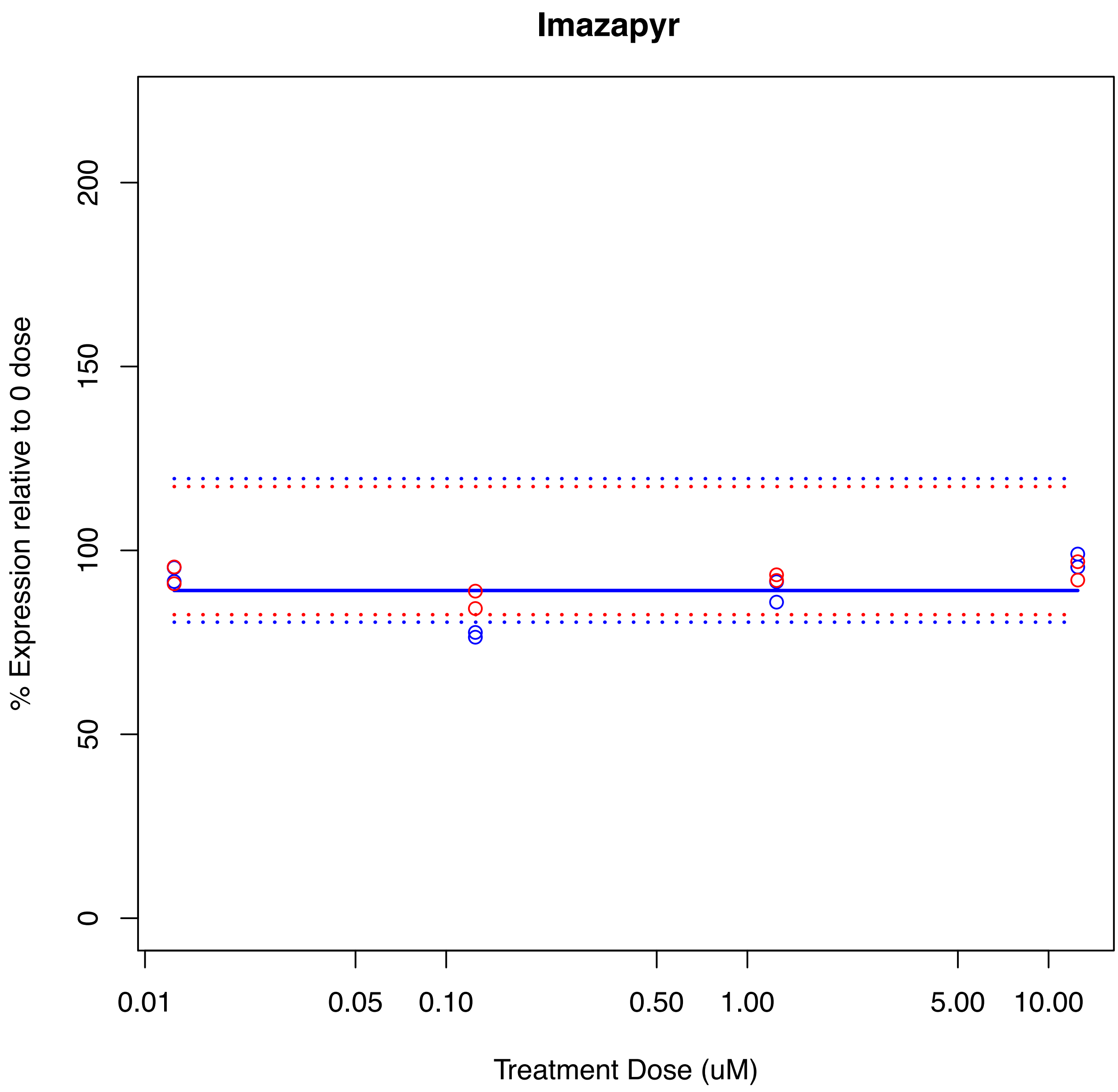
Imazapyr
% Expression relative to 0 dose
0
50
100
150
200
0.01
0.05
0.10
0.50
1.00
5.00
10.00
Treatment Dose (uM)

2-Naphthacenecarboxamide, 4-(dimethylamino)-1,4,4a,5,5a,6,11, 12a-hexahydroxy-6-methyl-1,11-dioxo-, (4S-

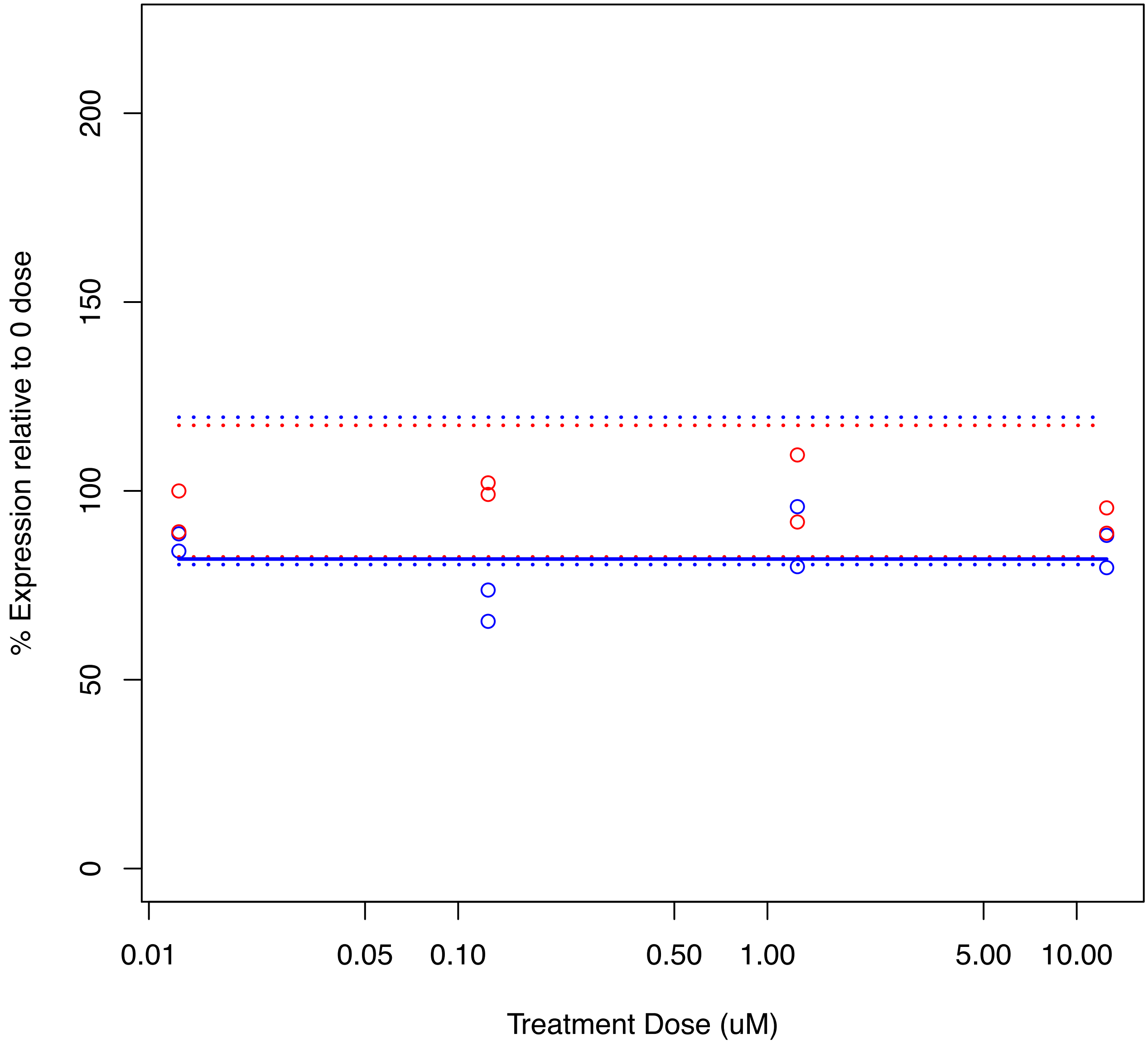

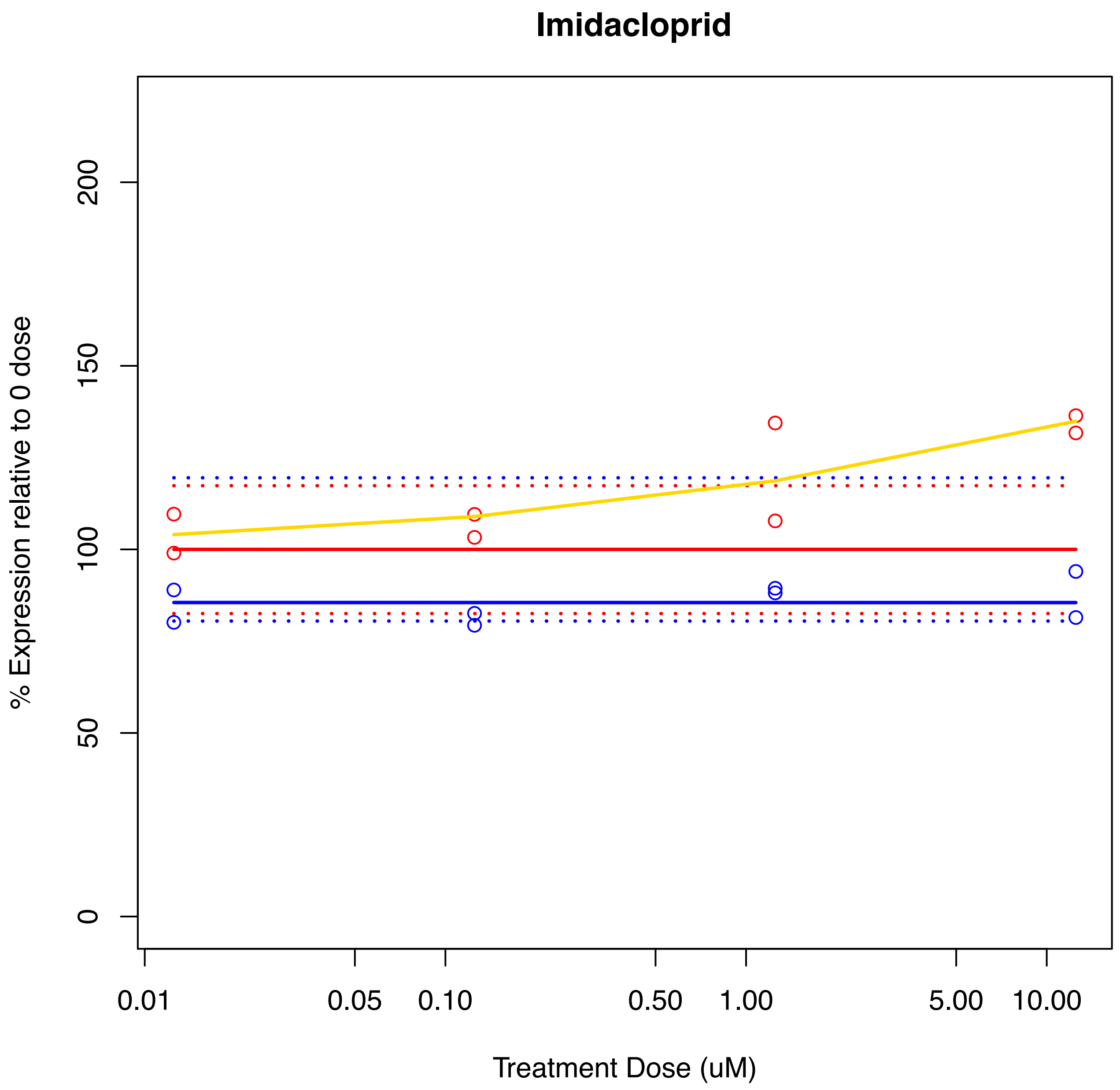

Imidacloprid
% Expression relative to 0 dose
0
50
100
150
200
0.01
0.05
0.10
0.50
1.00
5.00
10.00
Treatment Dose (uM)

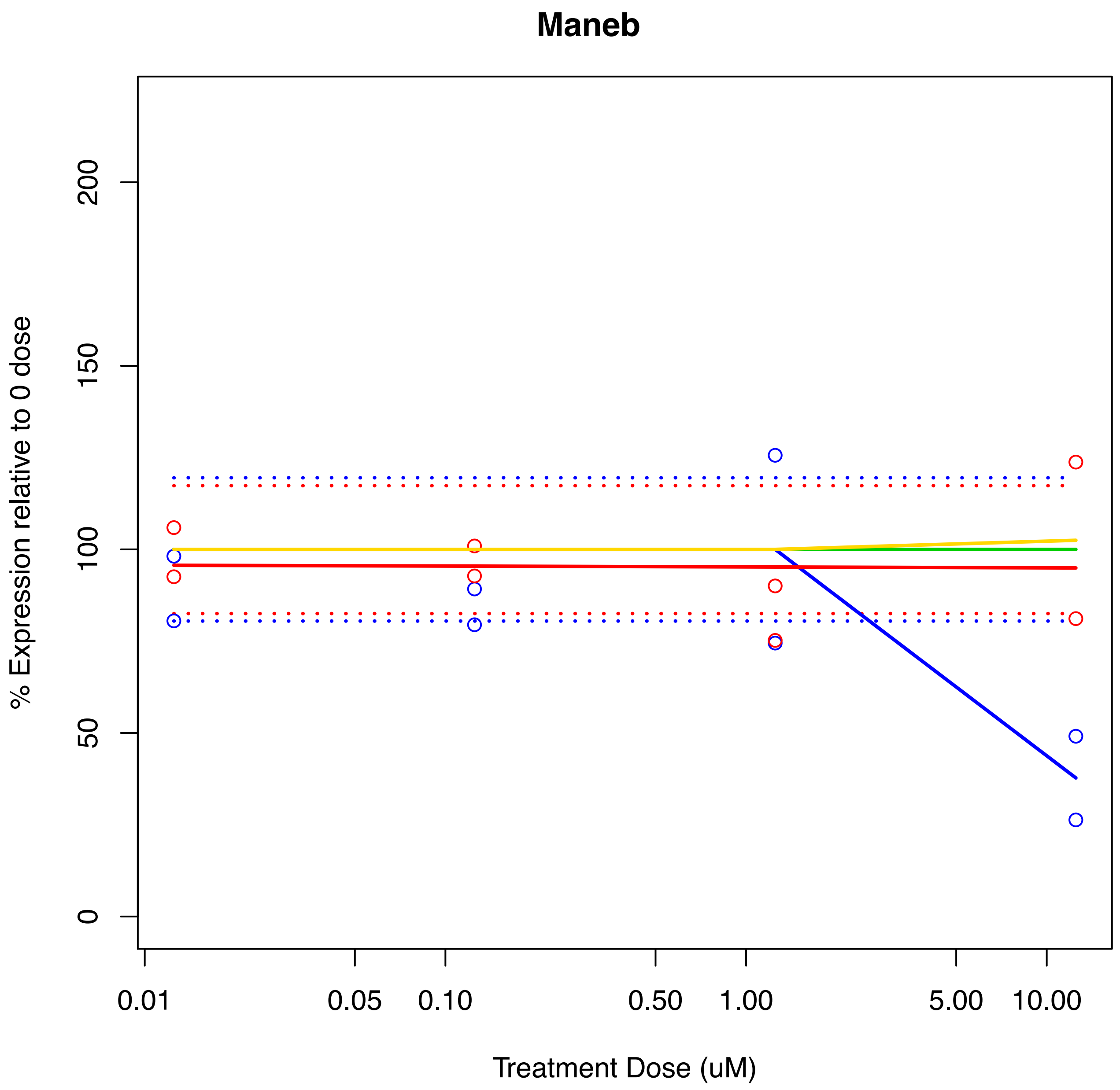

Maneb
% Expression relative to 0 dose
0
50
100
150
200
0.01
0.05
0.10
0.50
1.00
5.00
10.00
Treatment Dose (uM)

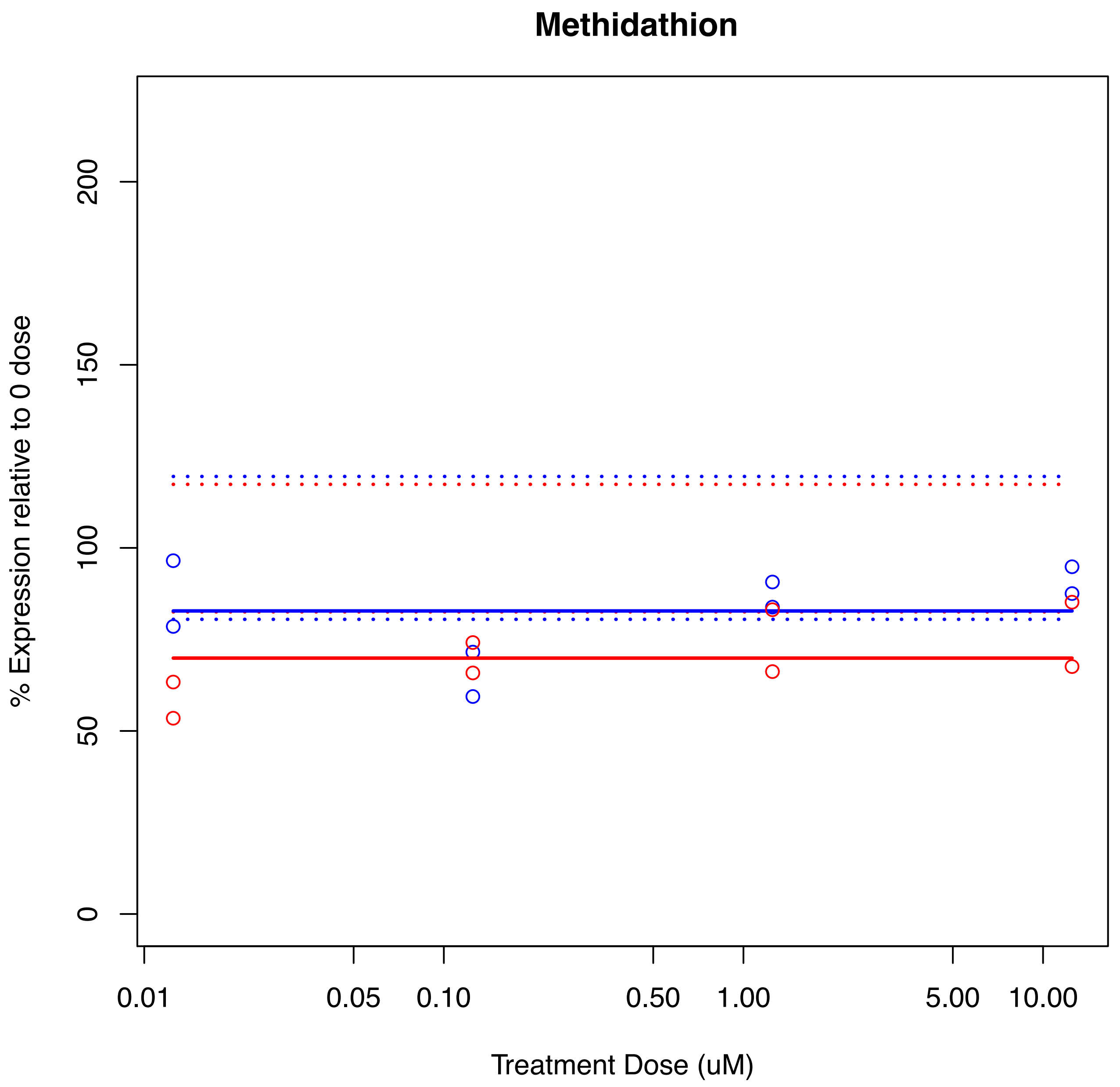
Methidathion
% Expression relative to 0 dose
0
50
100
150
200
0.01
0.05
0.10
0.50
1.00
5.00
10.00
Treatment Dose (uM)

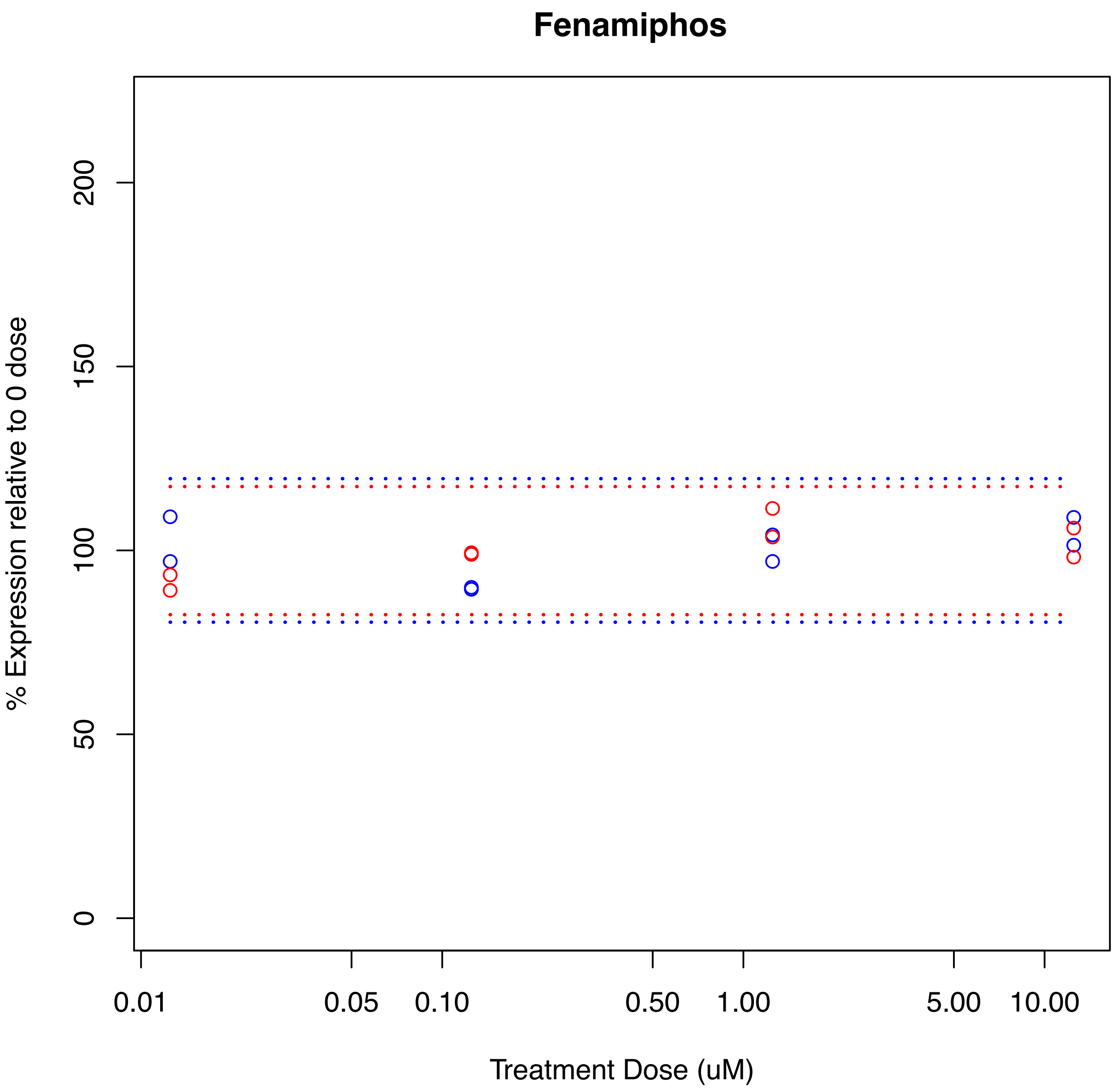
Fenamiphos
% Expression relative to 0 dose
0
50
100
150
200
0.01
0.05
0.10
0.50
1.00
5.00
10.00
Treatment Dose (uM)

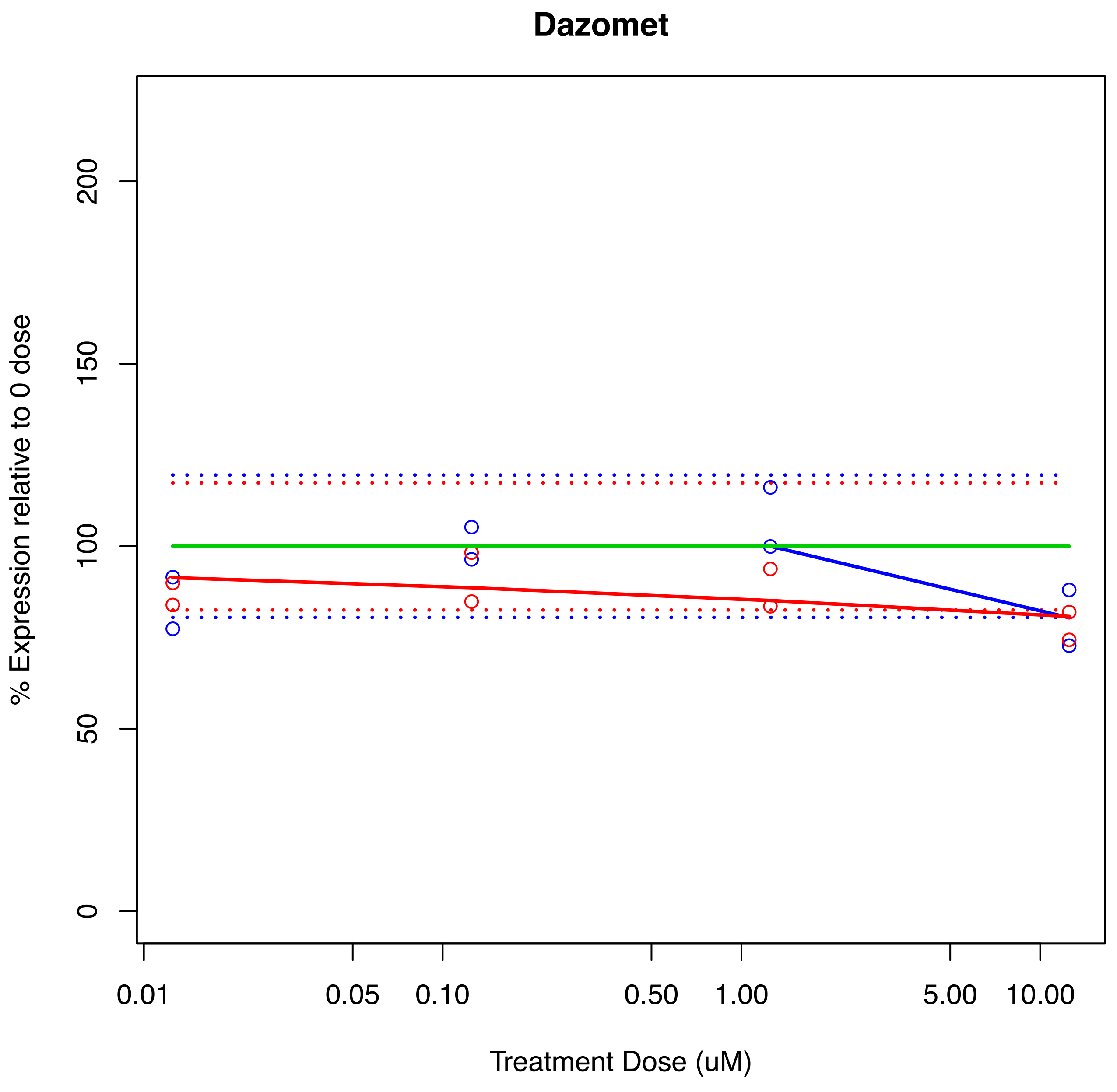
Dazomet
% Expression relative to 0 dose
0
50
100
150
200
0.01
0.05
0.10
0.50
1.00
5.00
10.00
Treatment Dose (uM)

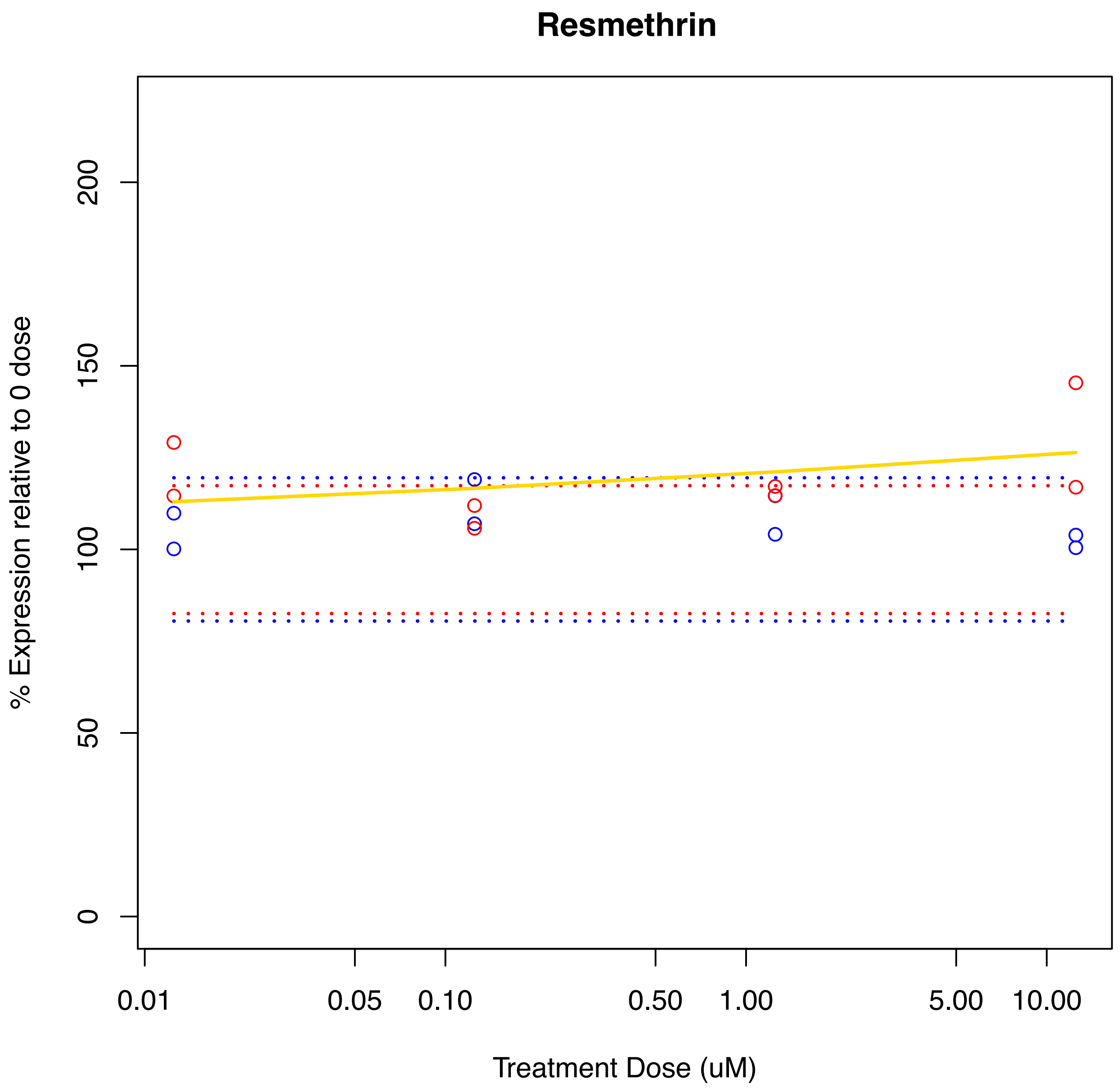
Resmethrin
% Expression relative to 0 dose
0
50
100
150
200
0.01
0.05
0.10
0.50
1.00
5.00
10.00
Treatment Dose (uM)

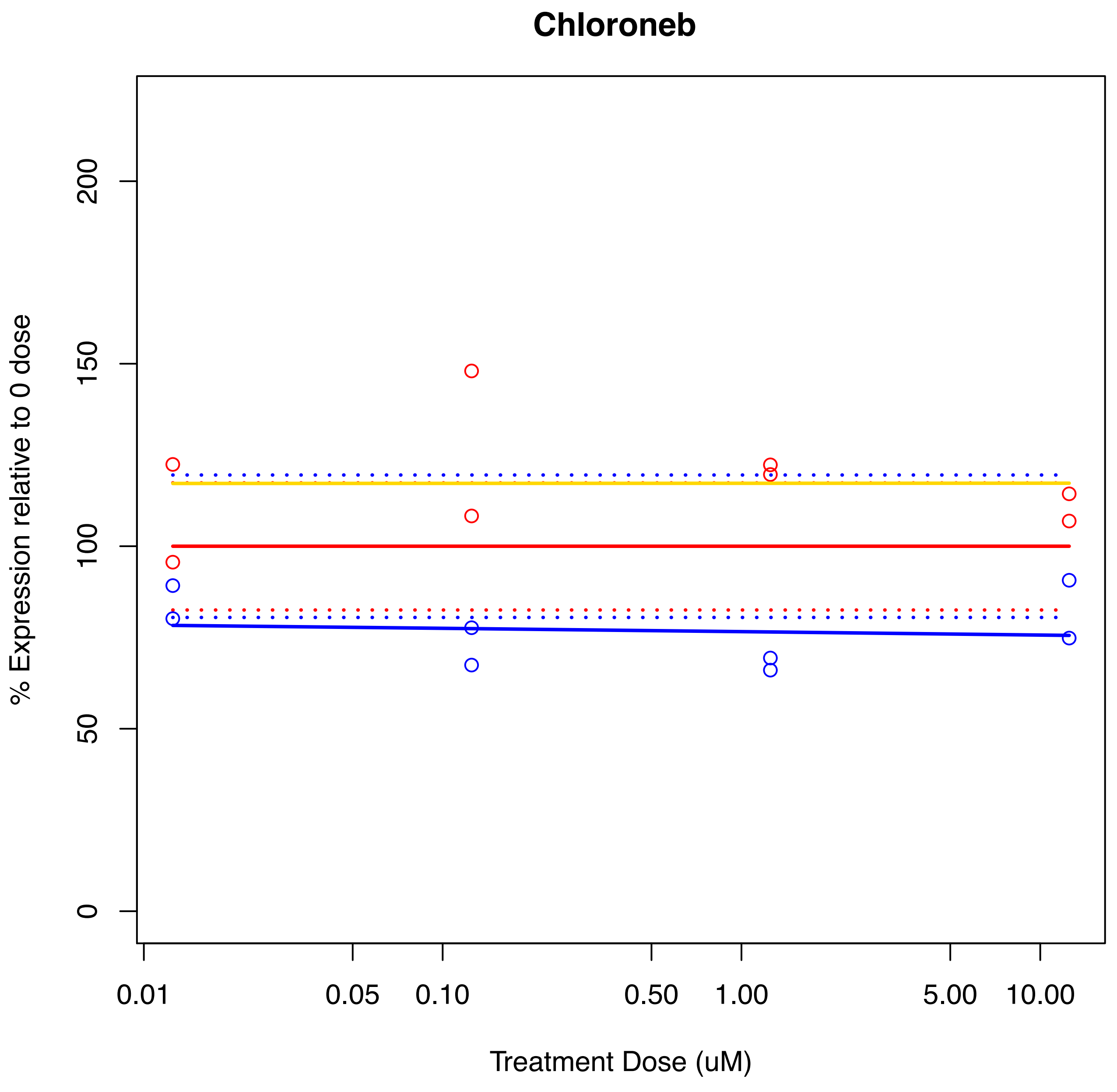

Chloroneb
% Expression relative to 0 dose
0
50
100
150
200
0.01
0.05
0.10
0.50
1.00
5.00
10.00
Treatment Dose (uM)

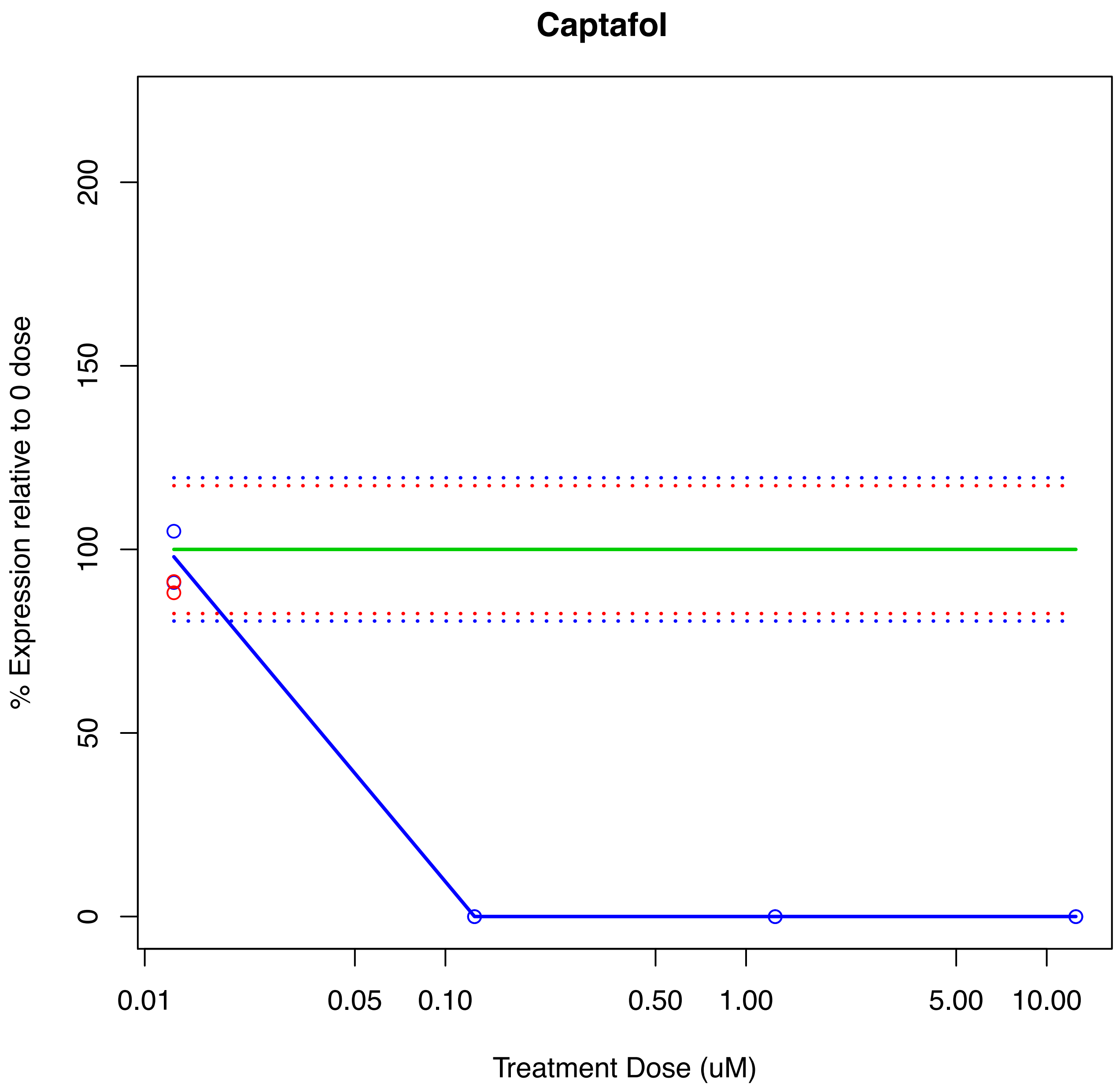

Captafol
% Expression relative to 0 dose
0
50
100
150
200
0.01
0.05
0.10
0.50
1.00
5.00
10.00
Treatment Dose (uM)

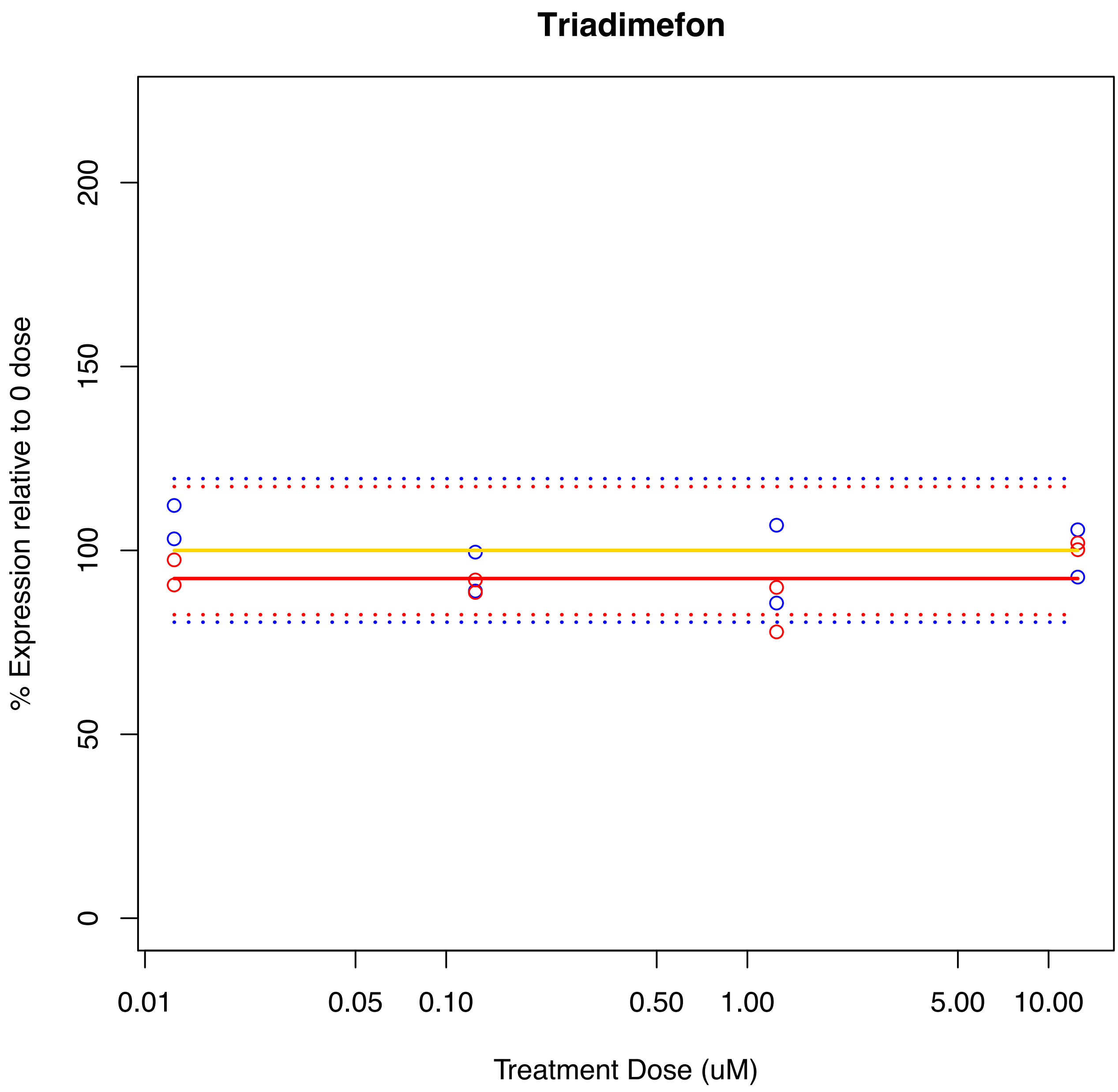
Triadimefon
% Expression relative to 0 dose
0
50
100
150
200
0.01
0.05
0.10
0.50
1.00
5.00
10.00
Treatment Dose (uM)

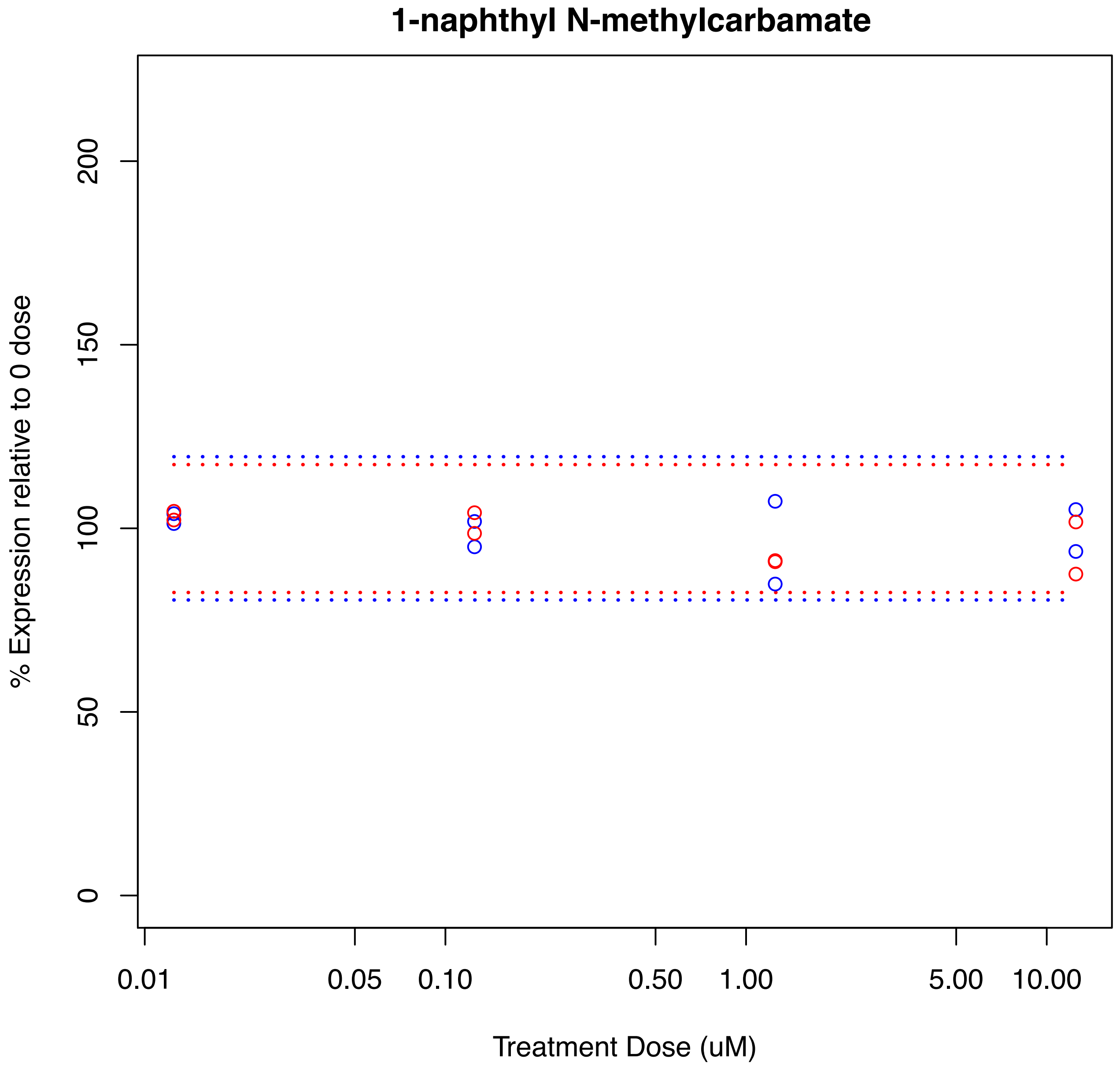
1-naphthyl N-methylcarbamate
% Expression relative to 0 dose
0
50
100
150
200
0.01
0.05
0.10
0.50
1.00
5.00
10.00
Treatment Dose (uM)

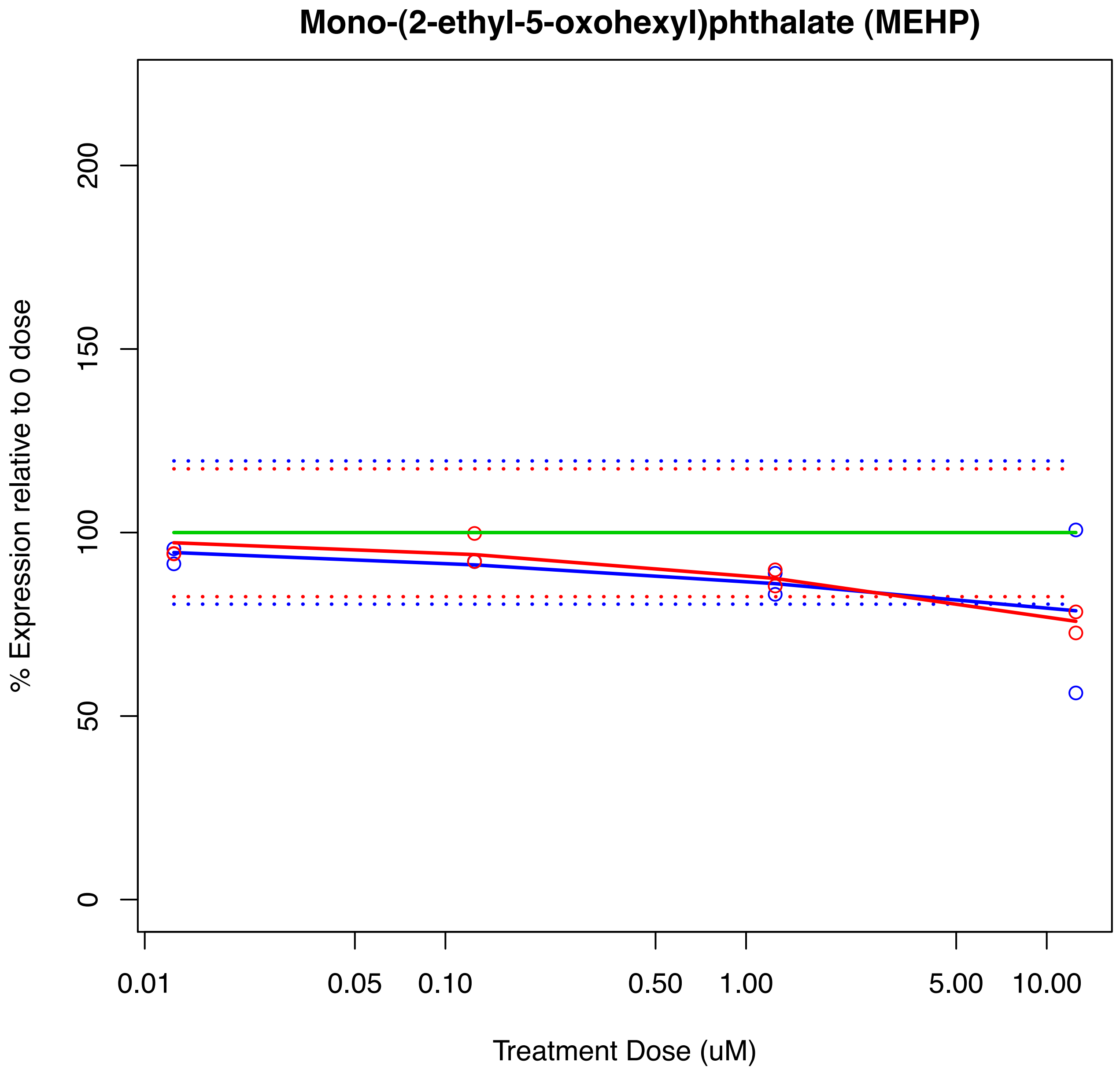

Mono-(2-ethyl-5-oxohexyl)phthalate (MEHP)
% Expression relative to 0 dose
0
50
100
150
200
0.01
0.05
0.10
0.50
1.00
5.00
10.00
Treatment Dose (uM)

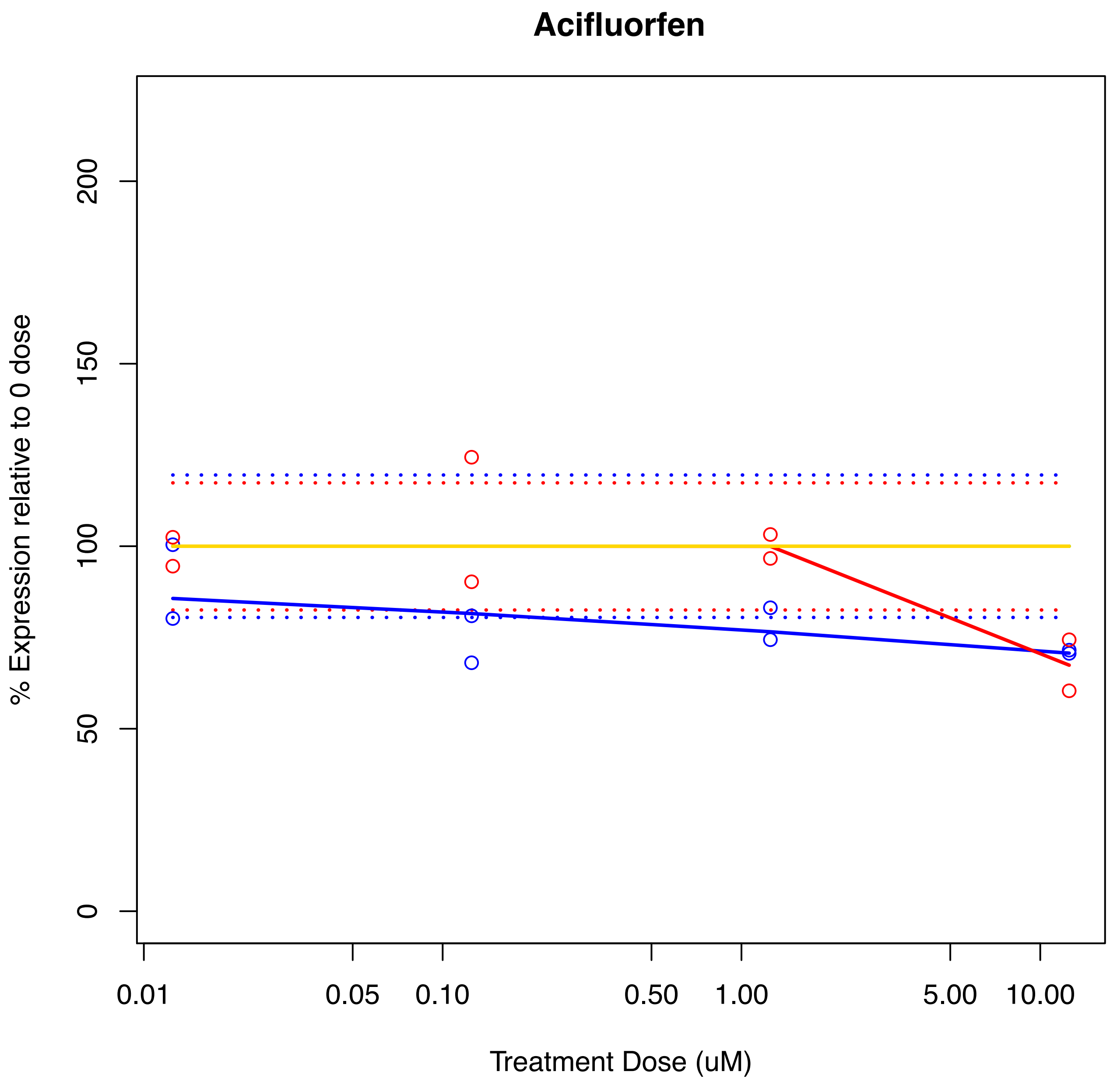

Acifluorfen
% Expression relative to 0 dose
200
150
100
50
0
0.01
0.05
0.10
0.50
1.00
5.00
10.00
Treatment Dose (uM)

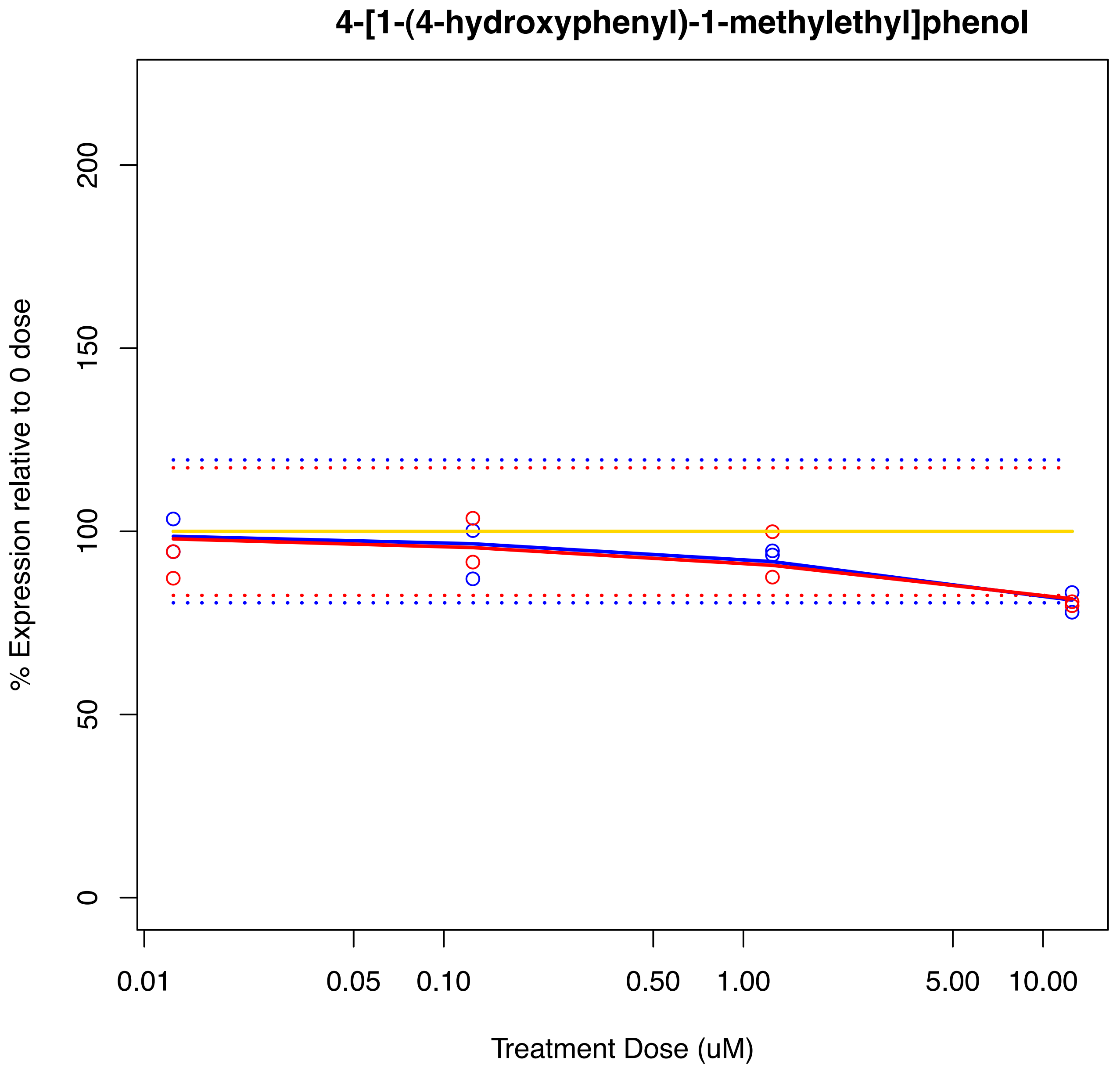
4-[1-(4-hydroxyphenyl)-1-methylethyl]phenol
% Expression relative to 0 dose
0
50
100
150
200
0.01
0.05
0.10
0.50
1.00
5.00
10.00
Treatment Dose (uM)

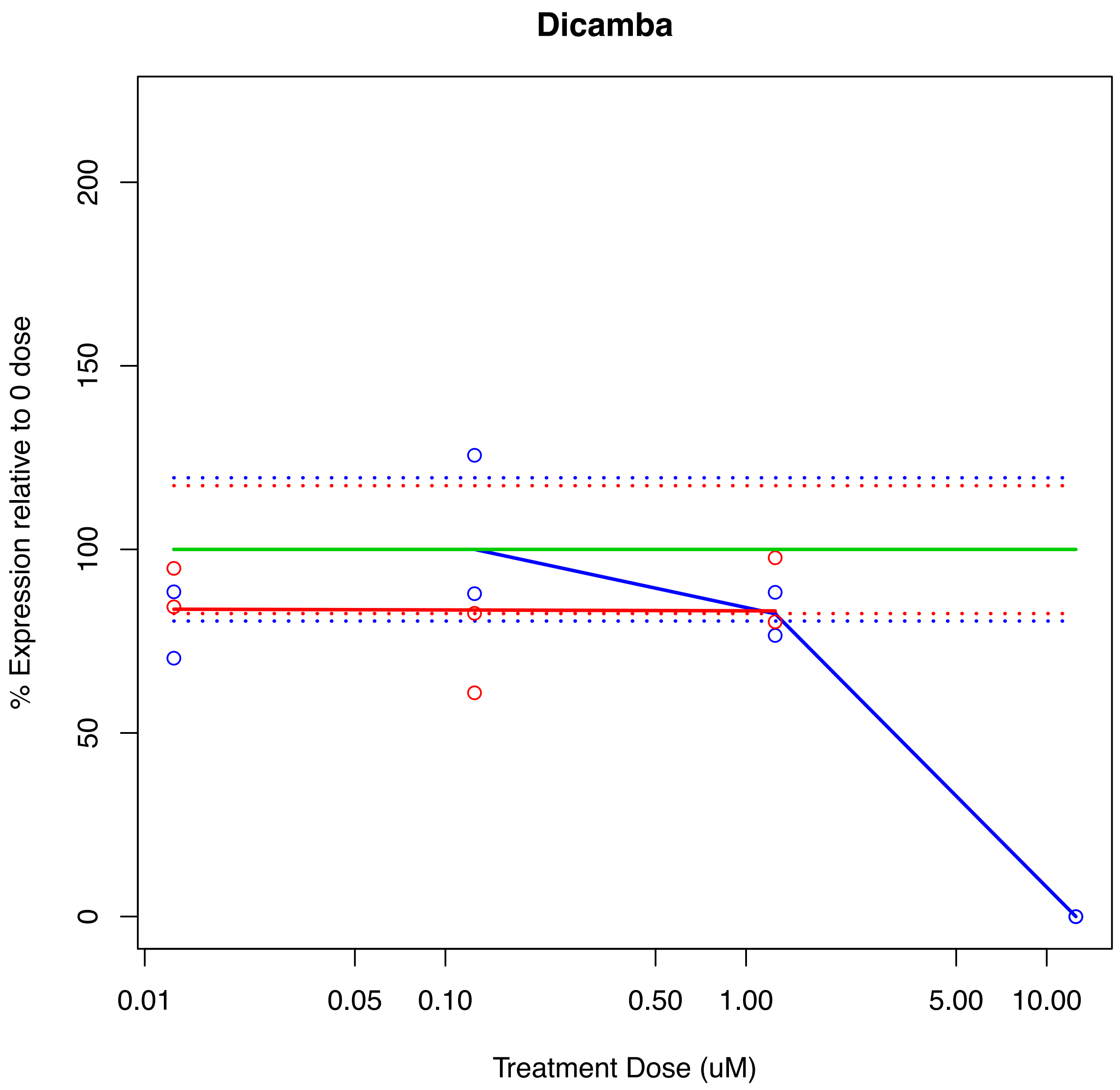

Dicamba
% Expression relative to 0 dose
0
50
100
150
200
0.01
0.05
0.10
0.50
1.00
5.00
10.00
Treatment Dose (uM)

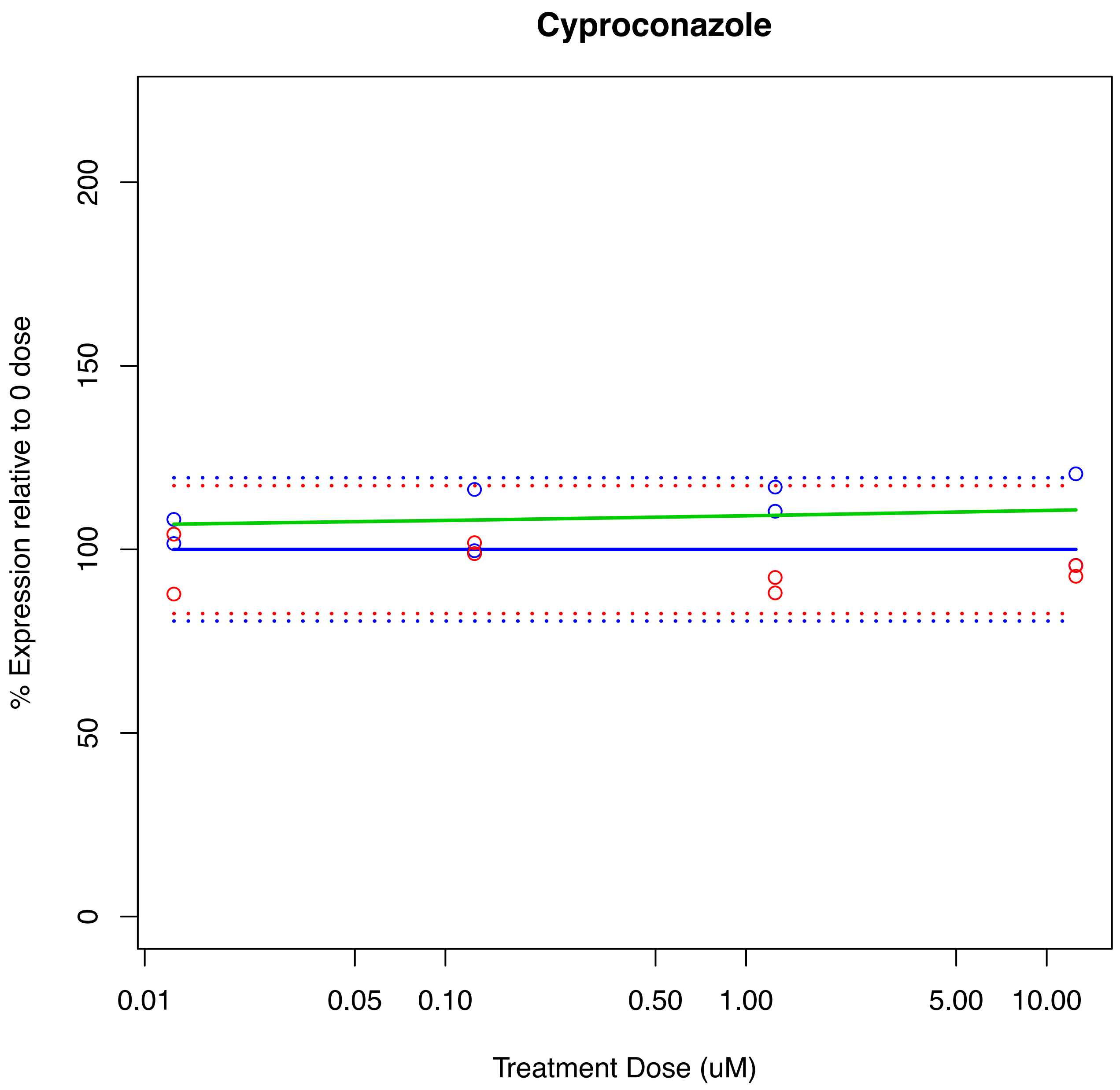
Cyproconazole
% Expression relative to 0 dose
0
50
100
150
200
0.01
0.05
0.10
0.50
1.00
5.00
10.00
Treatment Dose (uM)

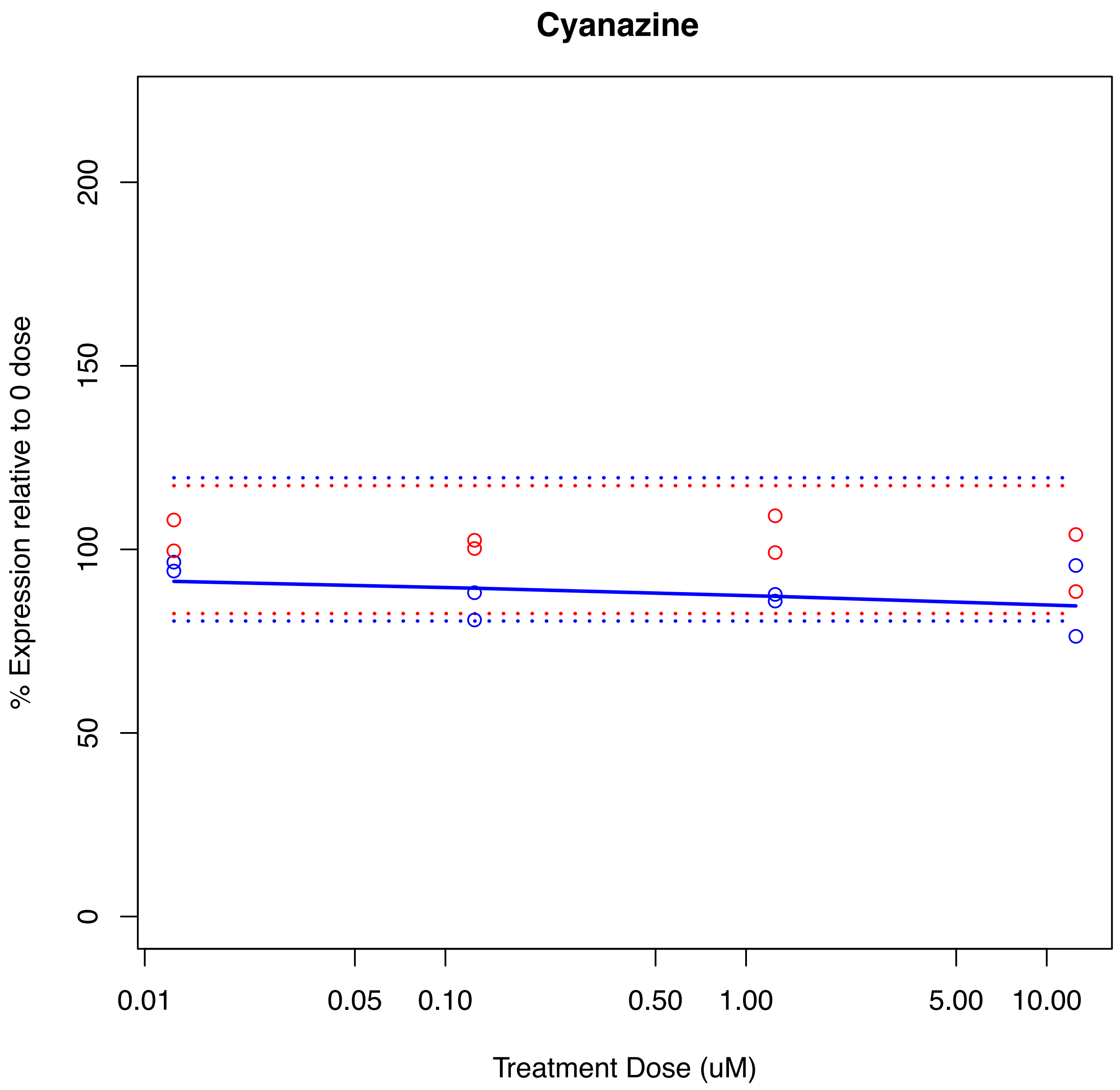

Cyanazine
% Expression relative to 0 dose
0
50
100
150
200
0.01
0.05
0.10
0.50
1.00
5.00
10.00
Treatment Dose (uM)

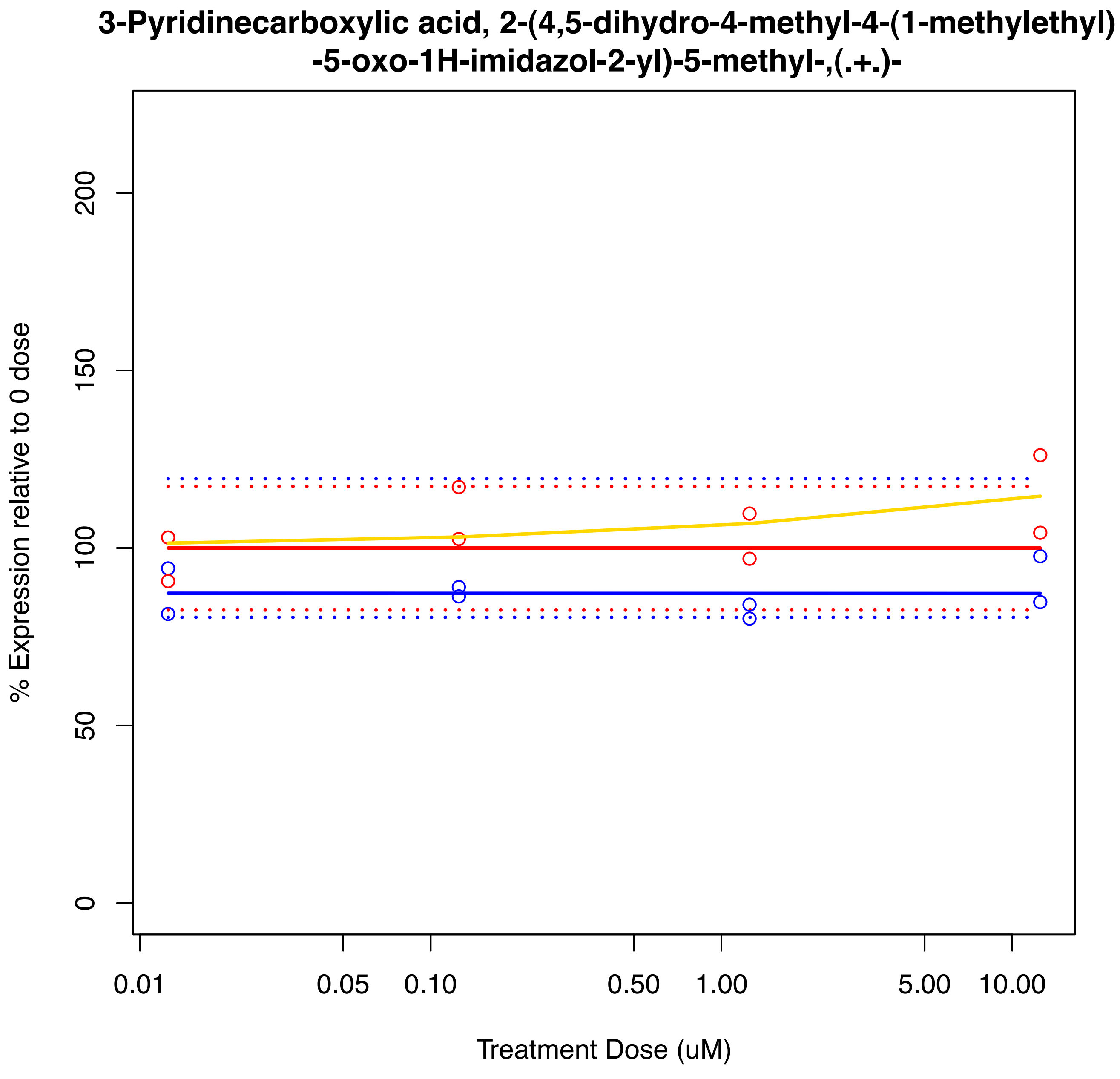
3-Pyridinecarboxylic acid, 2-(4,5-dihydro-4-methyl-4-(1-methylethyl)
-5-oxo-1H-imidazol-2-yl)-5-methyl-,(.+.)-
% Expression relative to 0 dose
0
50
100
150
200
0.01
0.05
0.10
0.50
1.00
5.00
10.00
Treatment Dose (uM)

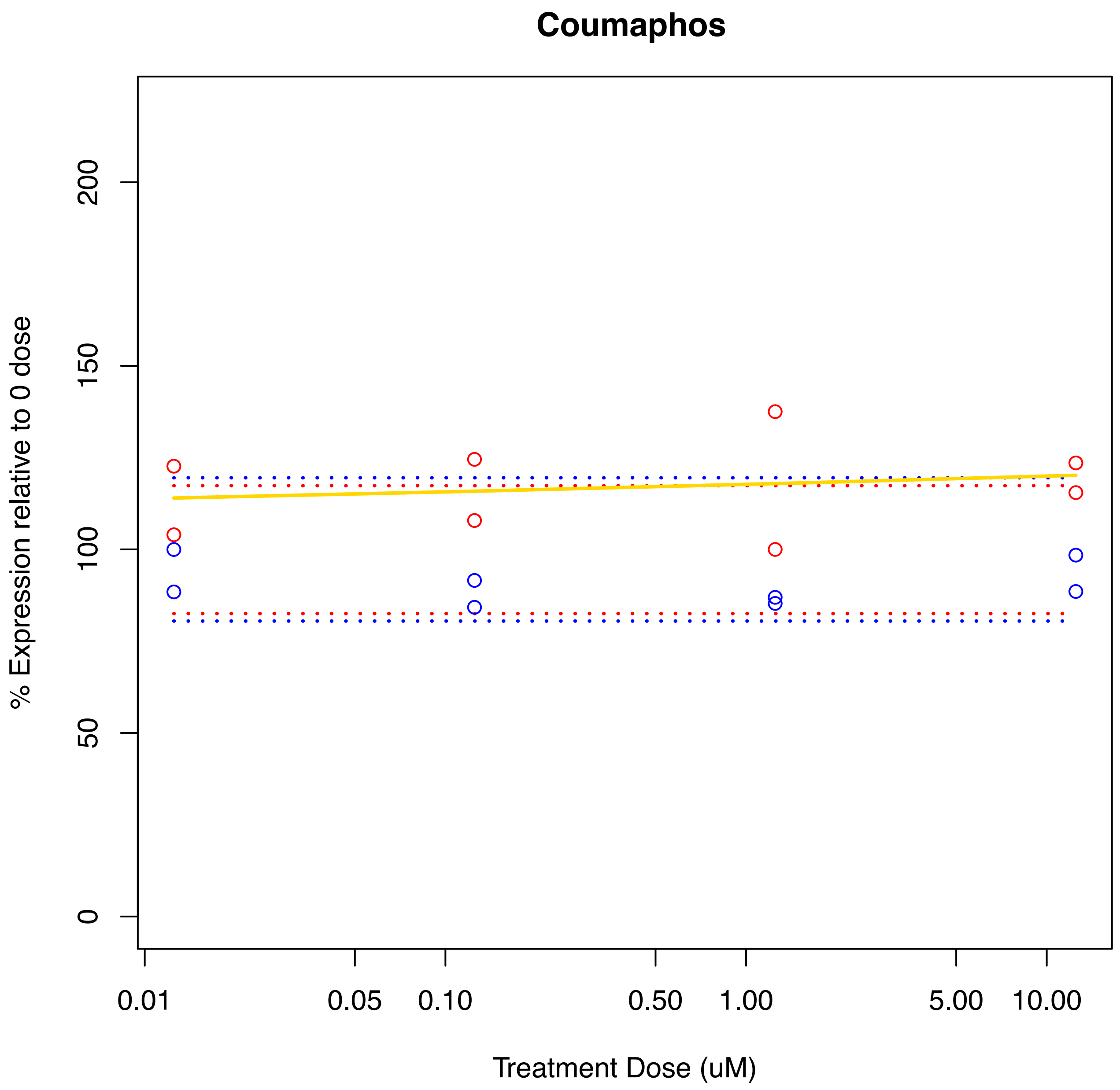
Coumaphos
% Expression relative to 0 dose
0
50
100
150
200
0.01
0.05
0.10
0.50
1.00
5.00
10.00
Treatment Dose (uM)

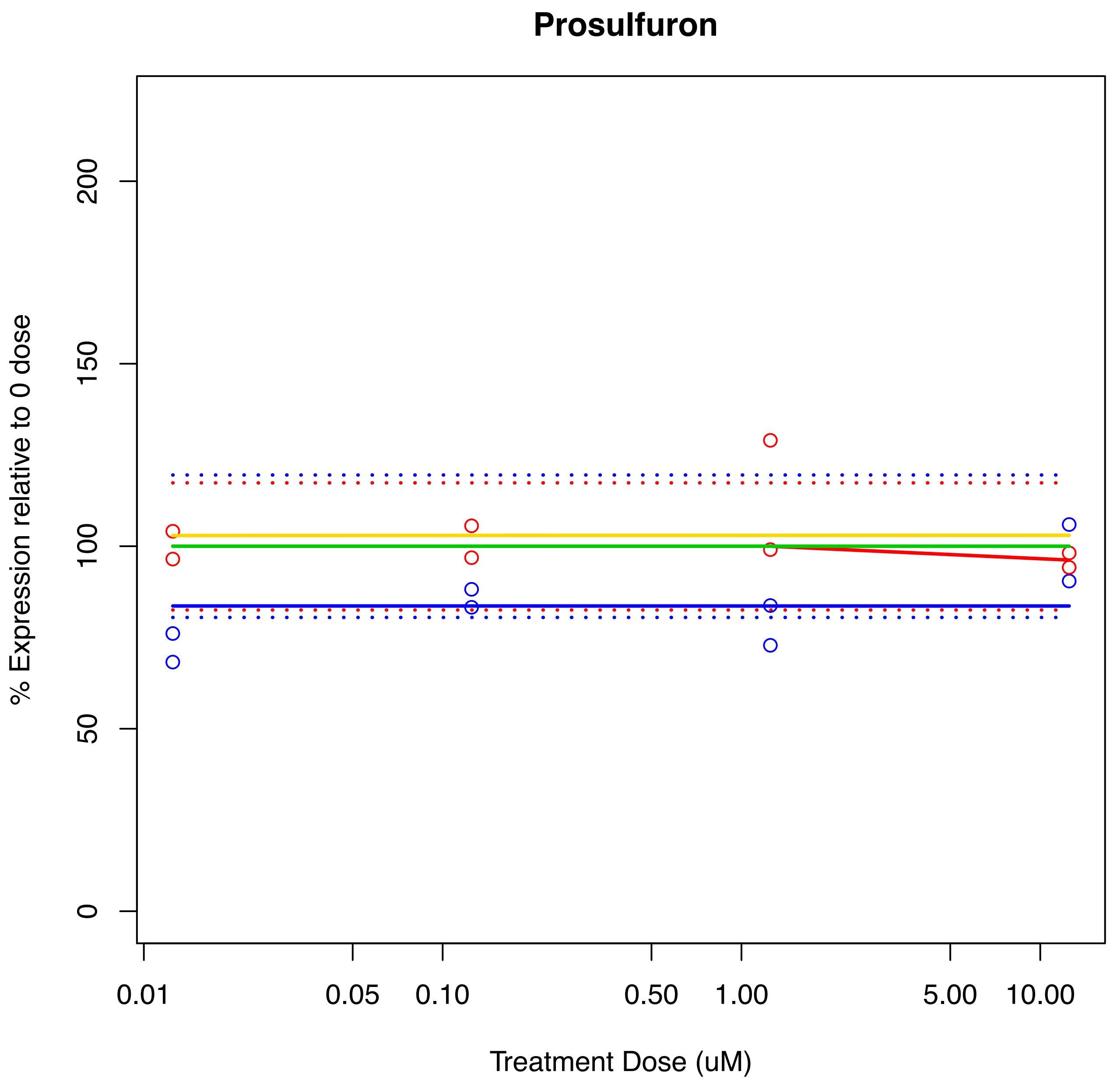

Prosulfuron
% Expression relative to 0 dose
0
50
100
150
200
0.01
0.05
0.10
0.50
1.00
5.00
10.00
Treatment Dose (uM)

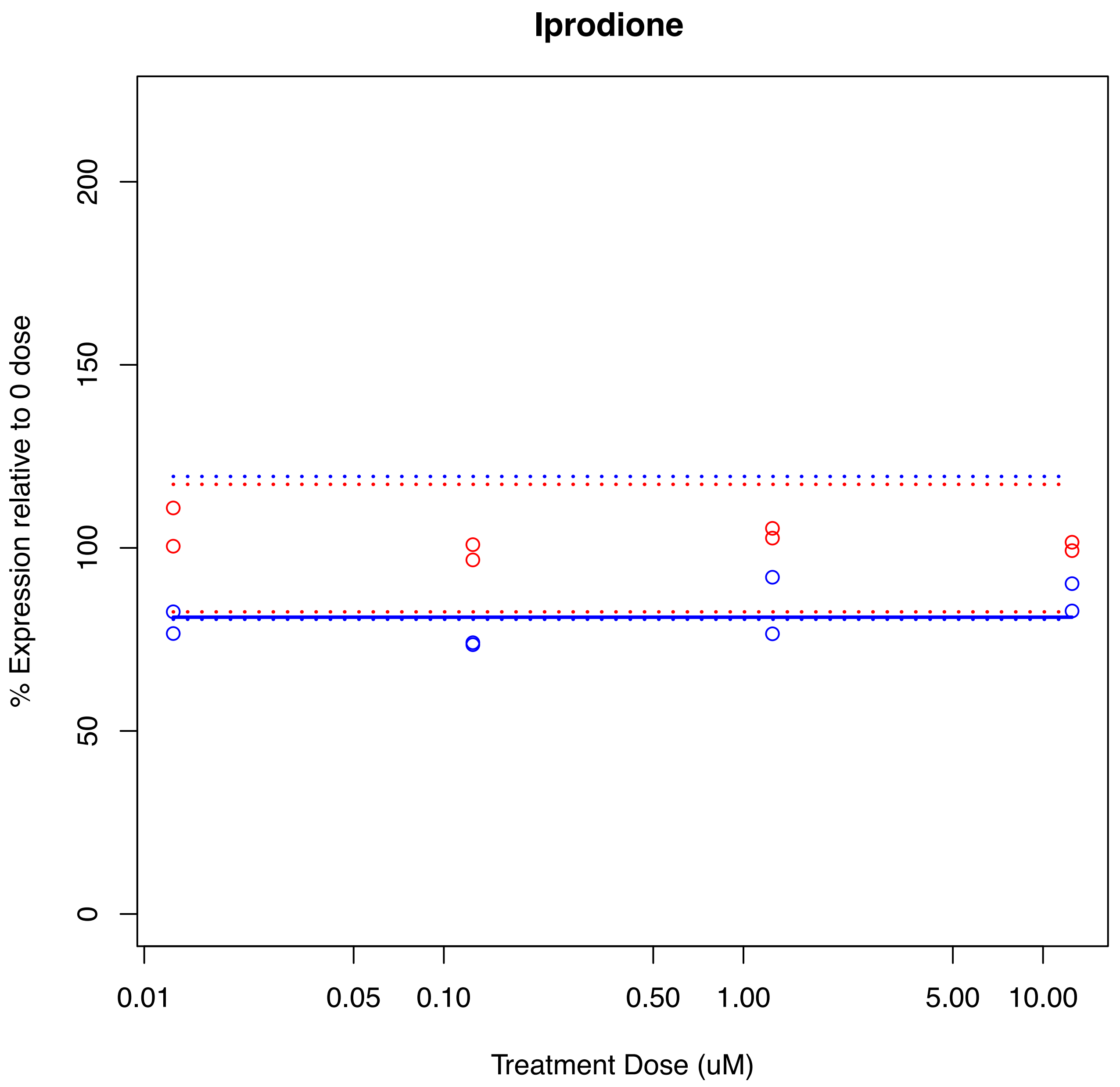

Iprodione
% Expression relative to 0 dose
0
50
100
150
200
0.01
0.05
0.10
0.50
1.00
5.00
10.00
Treatment Dose (uM)

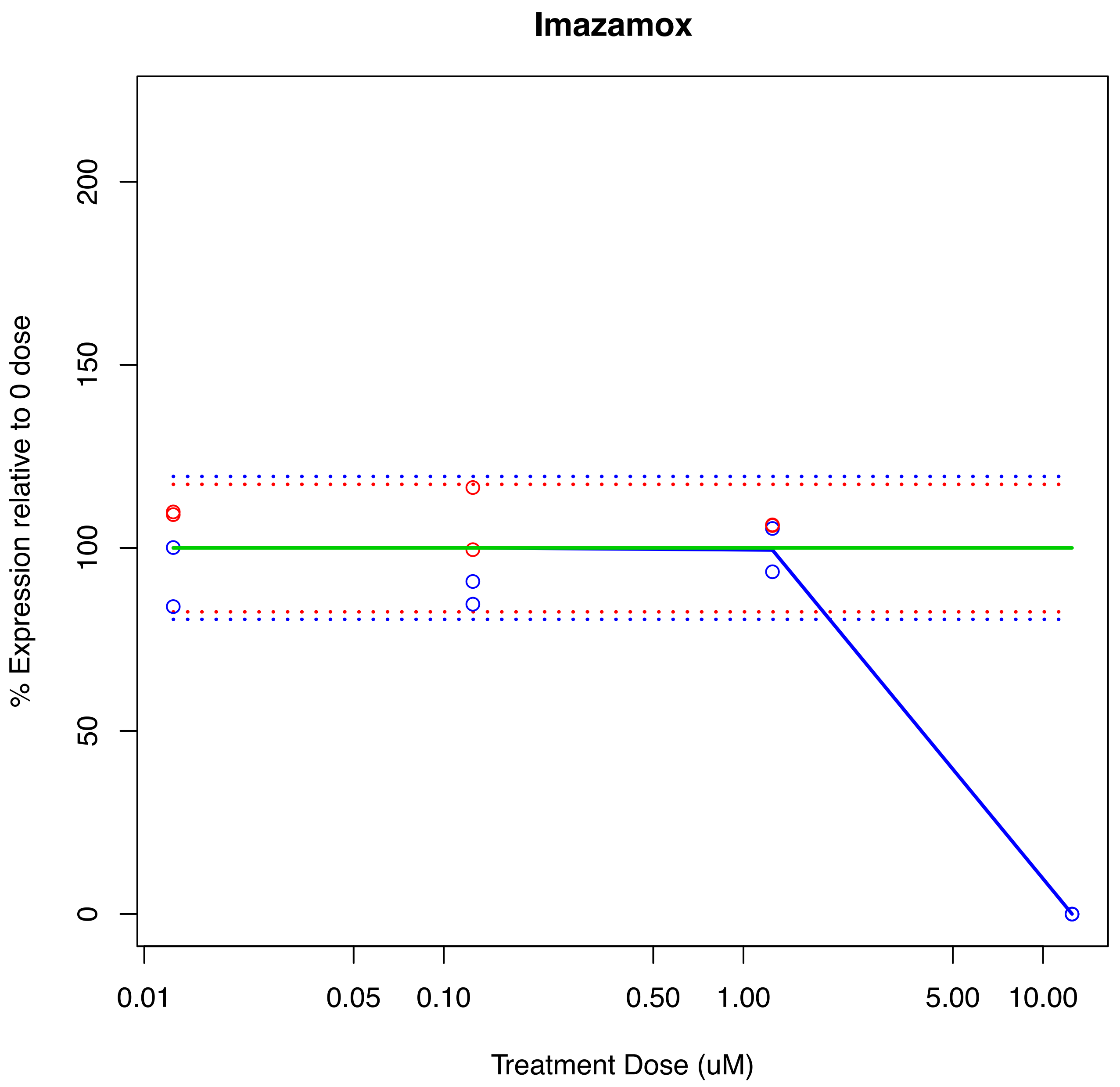
Imazamox
% Expression relative to 0 dose
0
50
100
150
200
0.01
0.05
0.10
0.50
1.00
5.00
10.00
Treatment Dose (uM)

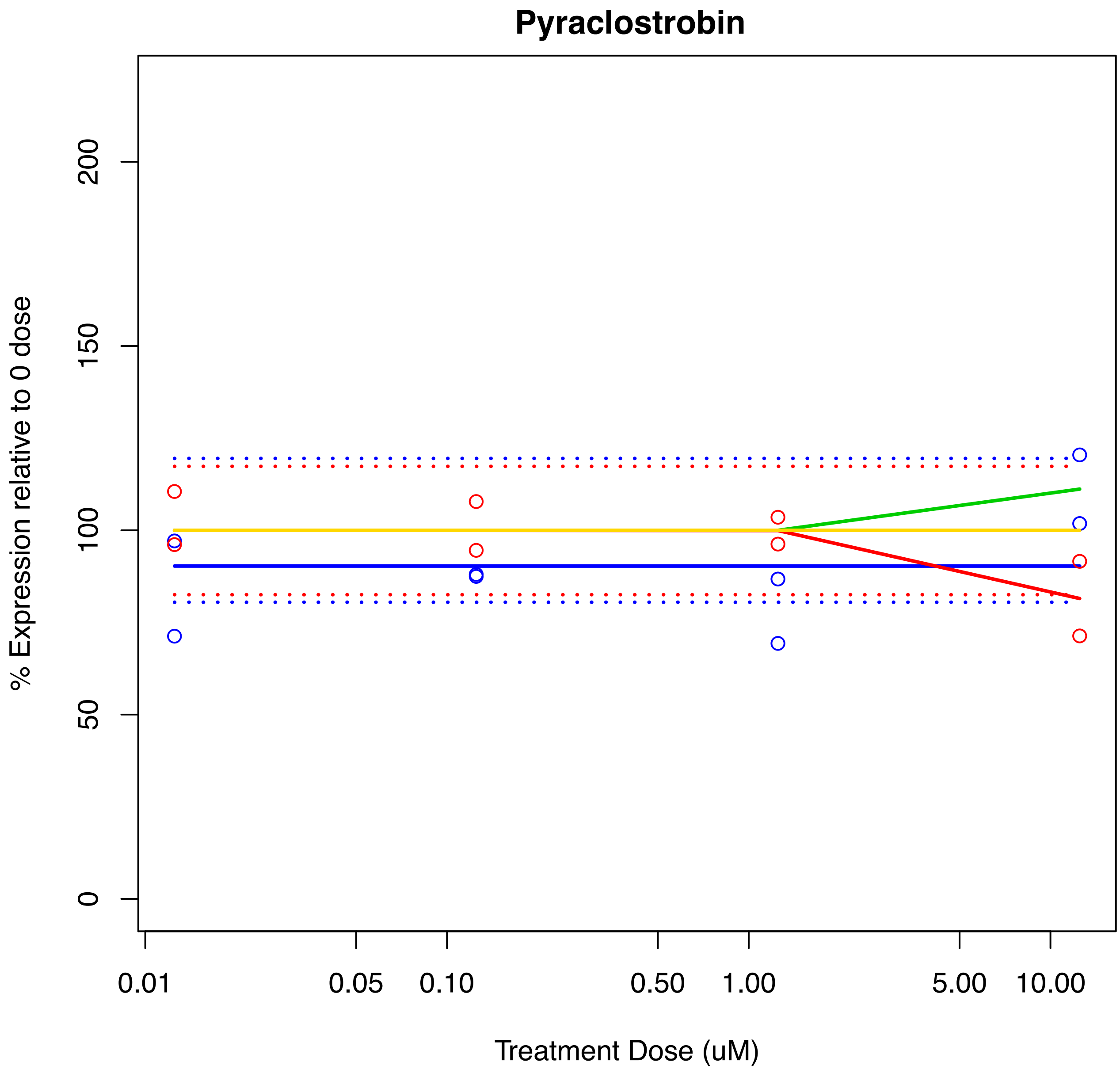

Pyraclostrobin
% Expression relative to 0 dose
0
50
100
150
200
0.01
0.05
0.10
0.50
1.00
5.00
10.00
Treatment Dose (uM)

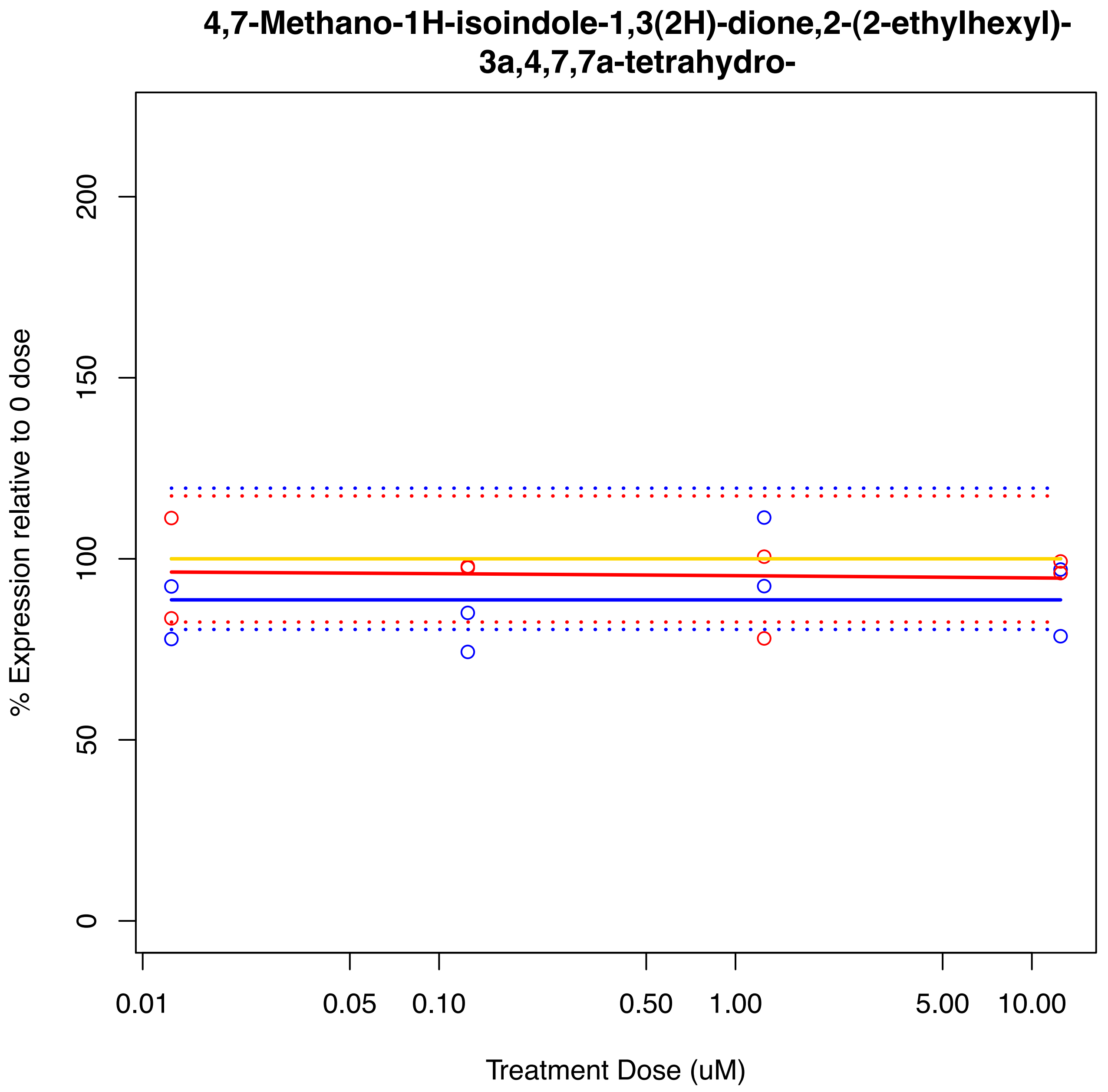
4,7-Methano-1H-isoindole-1,3(2H)-dione,2-(2-ethylhexyl)-
3a,4,7,7a-tetrahydro-
% Expression relative to 0 dose
0
50
100
150
200
0.01
0.05
0.10
0.50
1.00
5.00
10.00
Treatment Dose (uM)

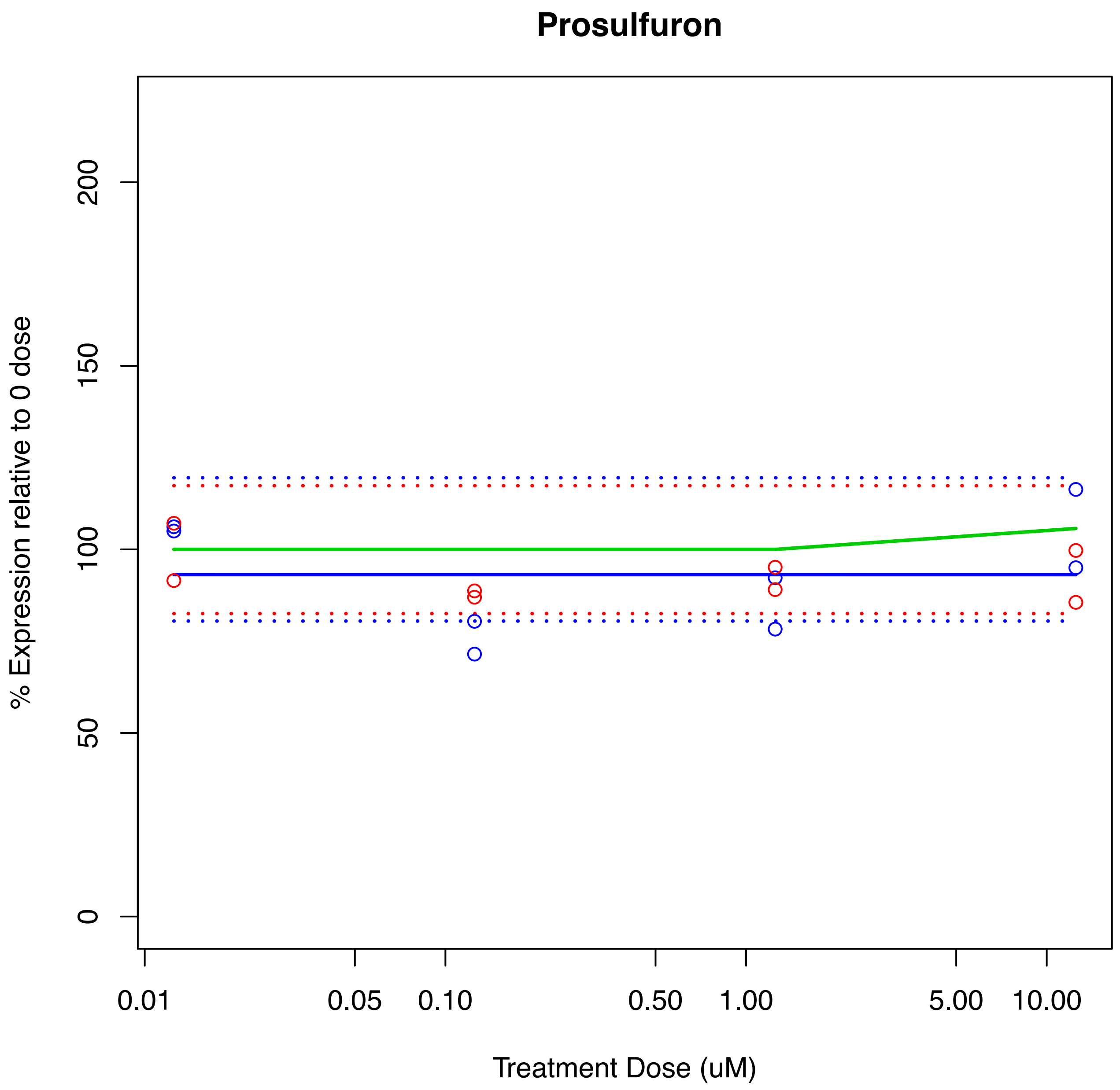

Prosulfuron
% Expression relative to 0 dose
0
50
100
150
200
0.01
0.05
0.10
0.50
1.00
5.00
10.00
Treatment Dose (uM)

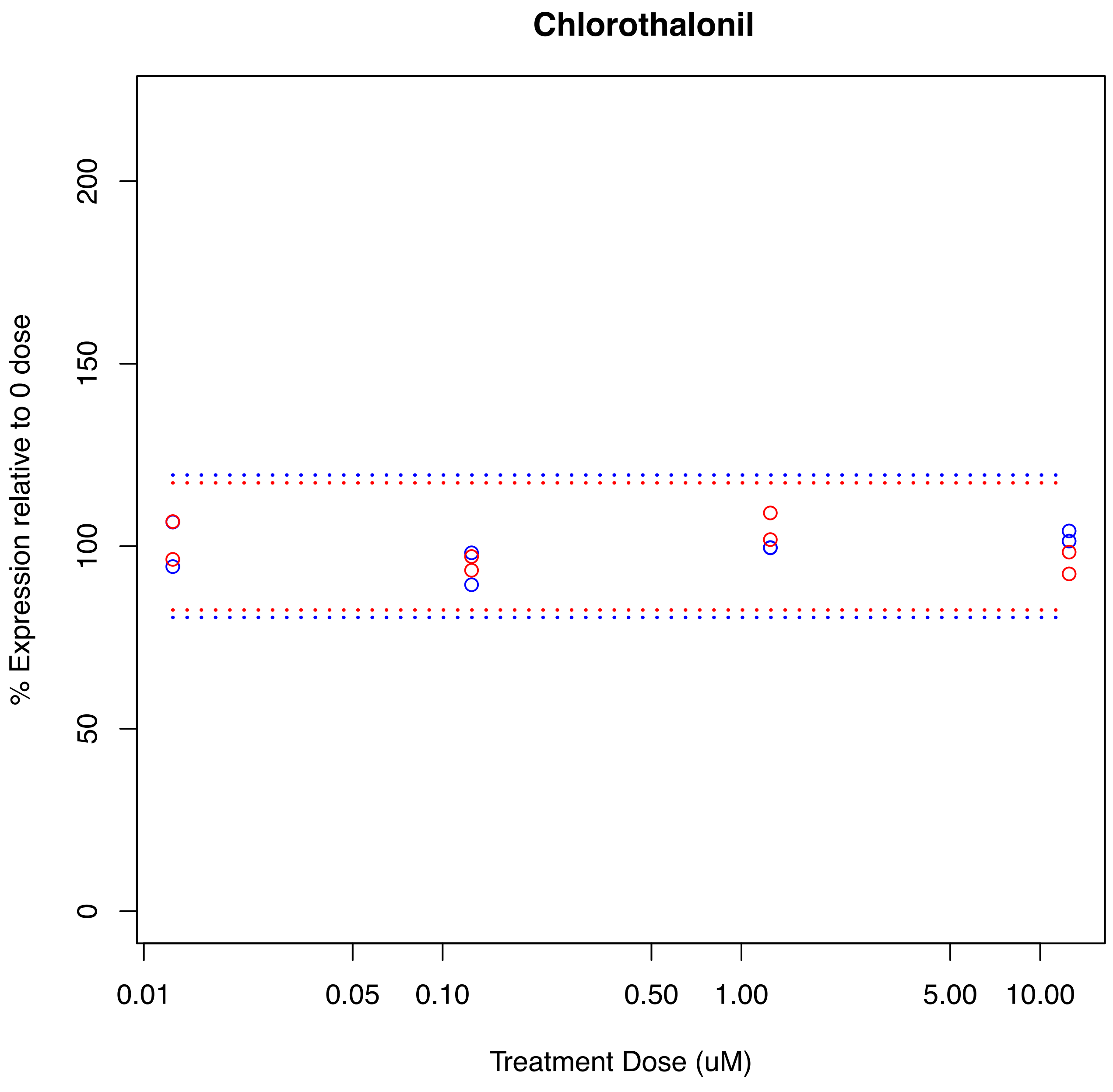
Chlorothalonil
% Expression relative to 0 dose
0
50
100
150
200
0.01
0.05
0.10
0.50
1.00
5.00
10.00
Treatment Dose (uM)

Thiodicarb

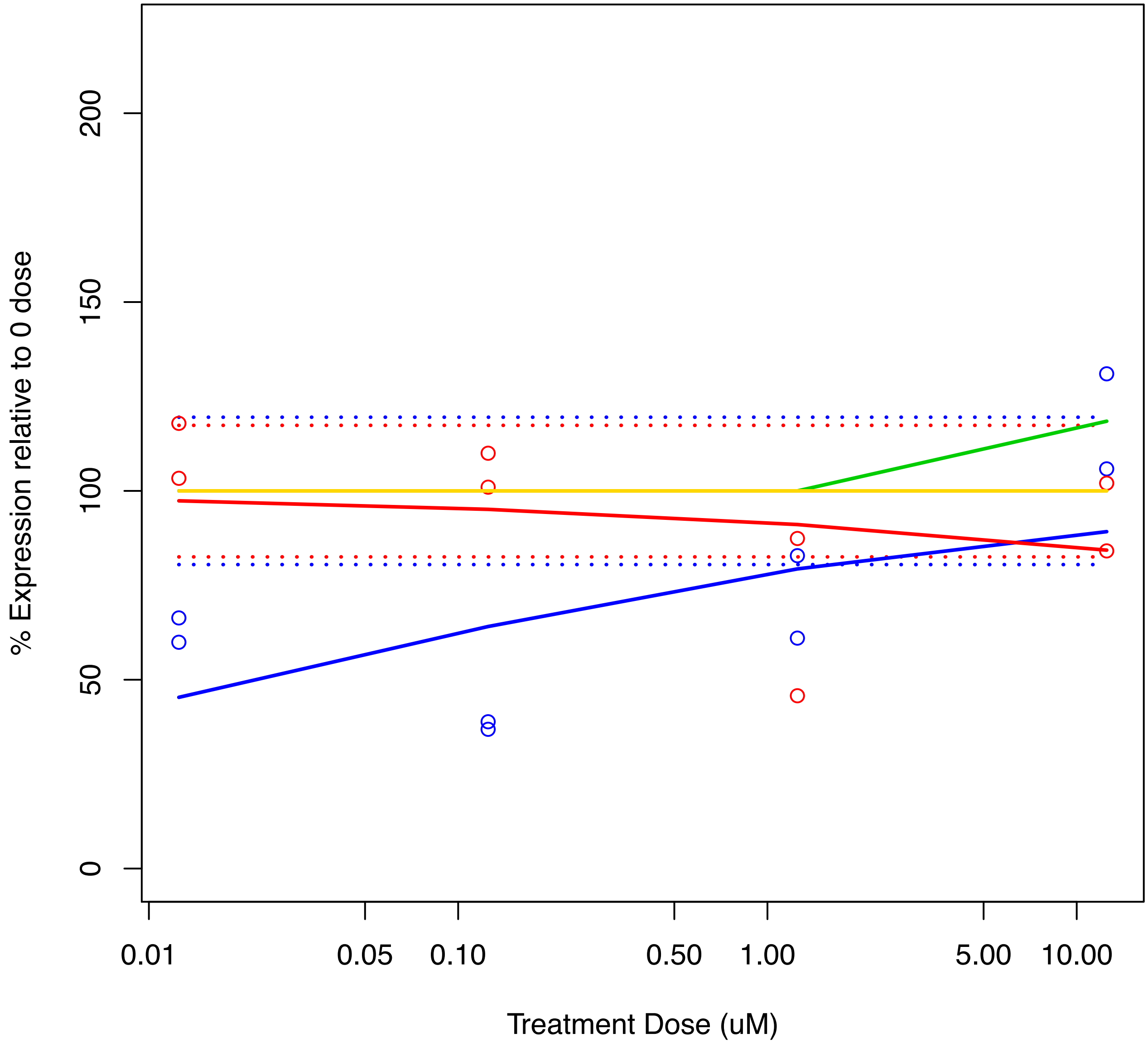

% Expression relative to 0 dose
0
50
100
150
200
0.01
0.05
0.10
0.50
1.00
5.00
10.00
Treatment Dose (uM)

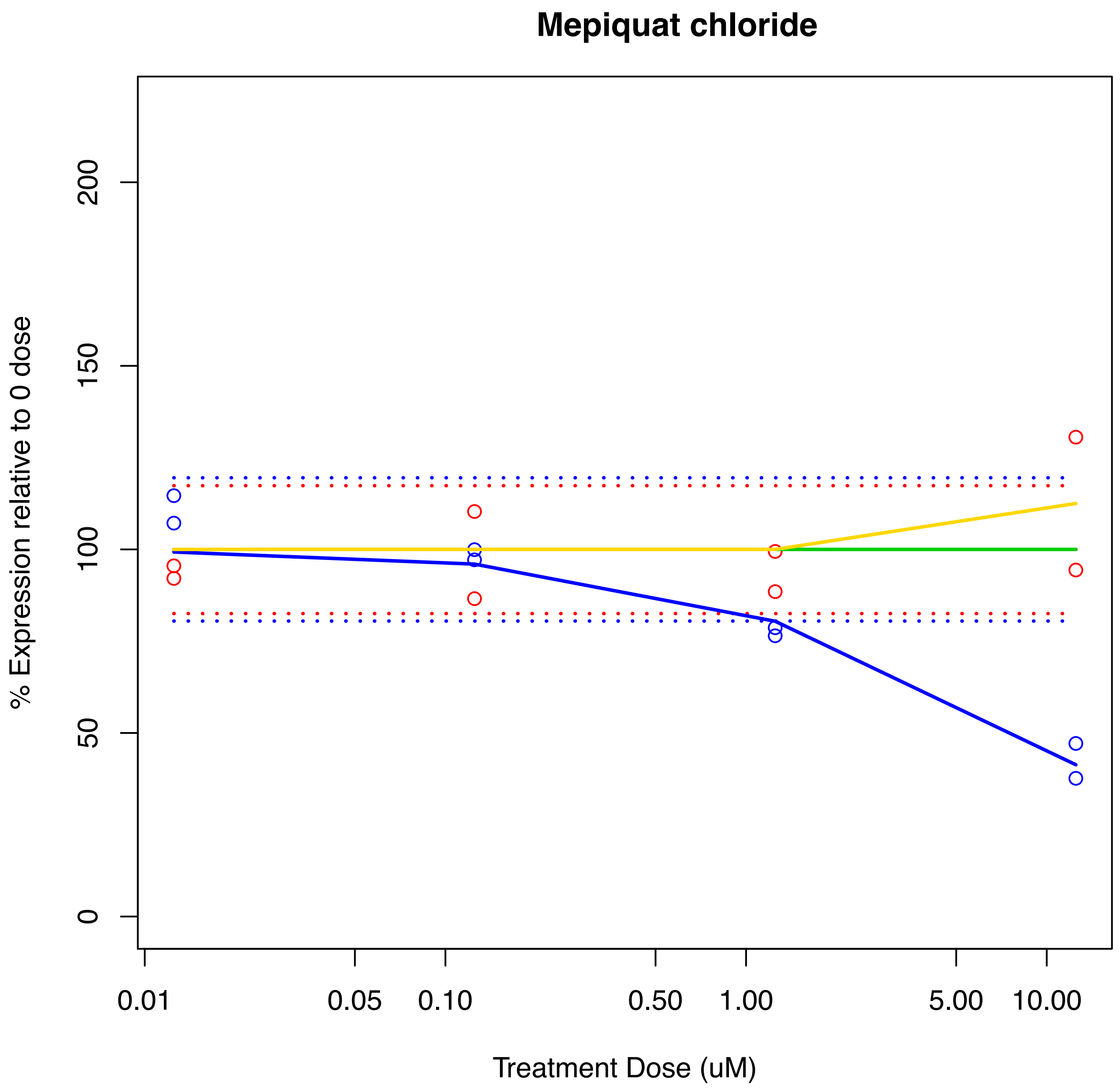

Mepiquat chloride
% Expression relative to 0 dose
0
50
100
150
200
0.01
0.05
0.10
0.50
1.00
5.00
10.00
Treatment Dose (uM)

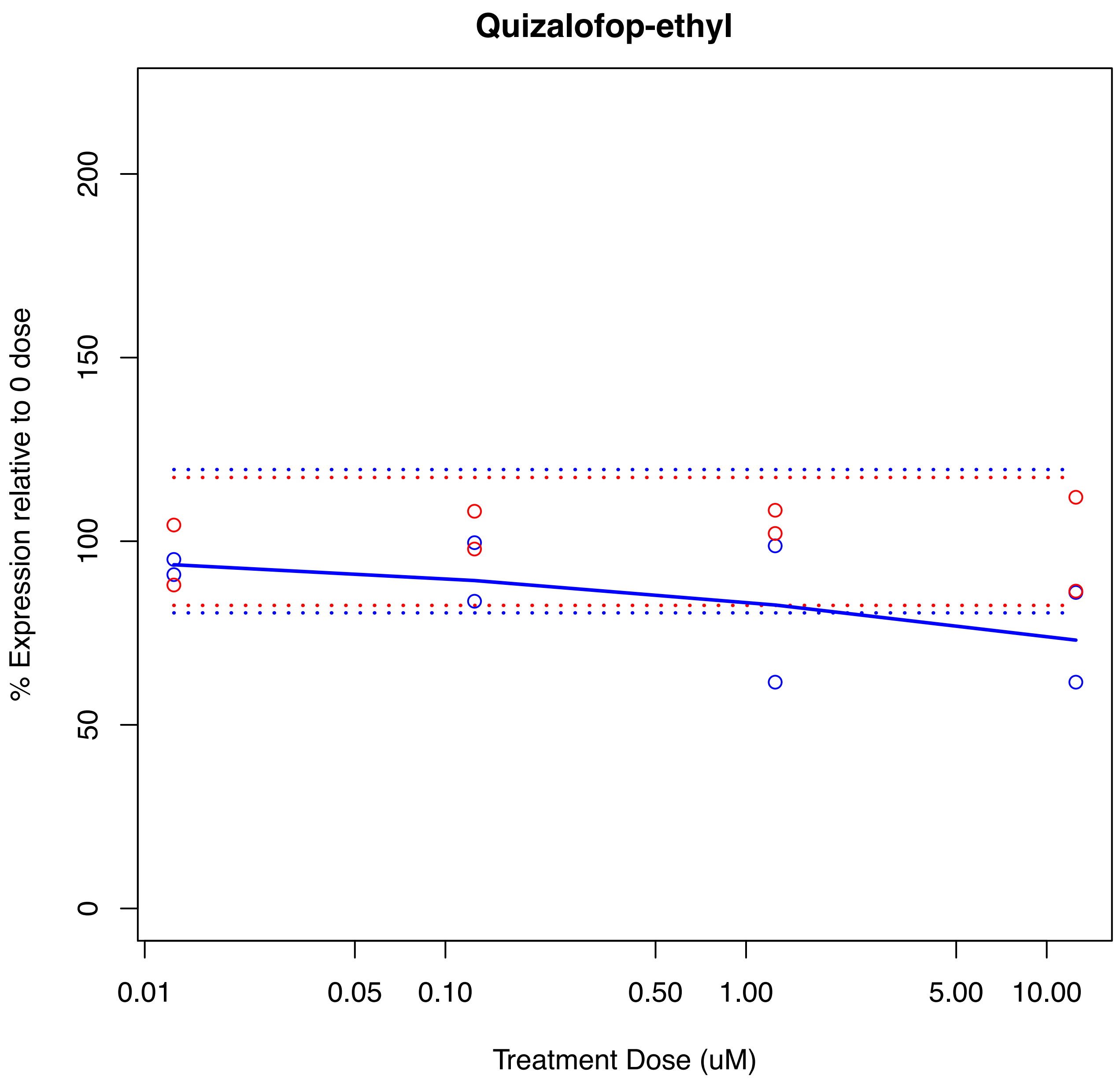
Quizalofop-ethyl
% Expression relative to 0 dose
0
50
100
150
200
0.01
0.05
0.10
0.50
1.00
5.00
10.00
Treatment Dose (uM)

Spirodiclofen

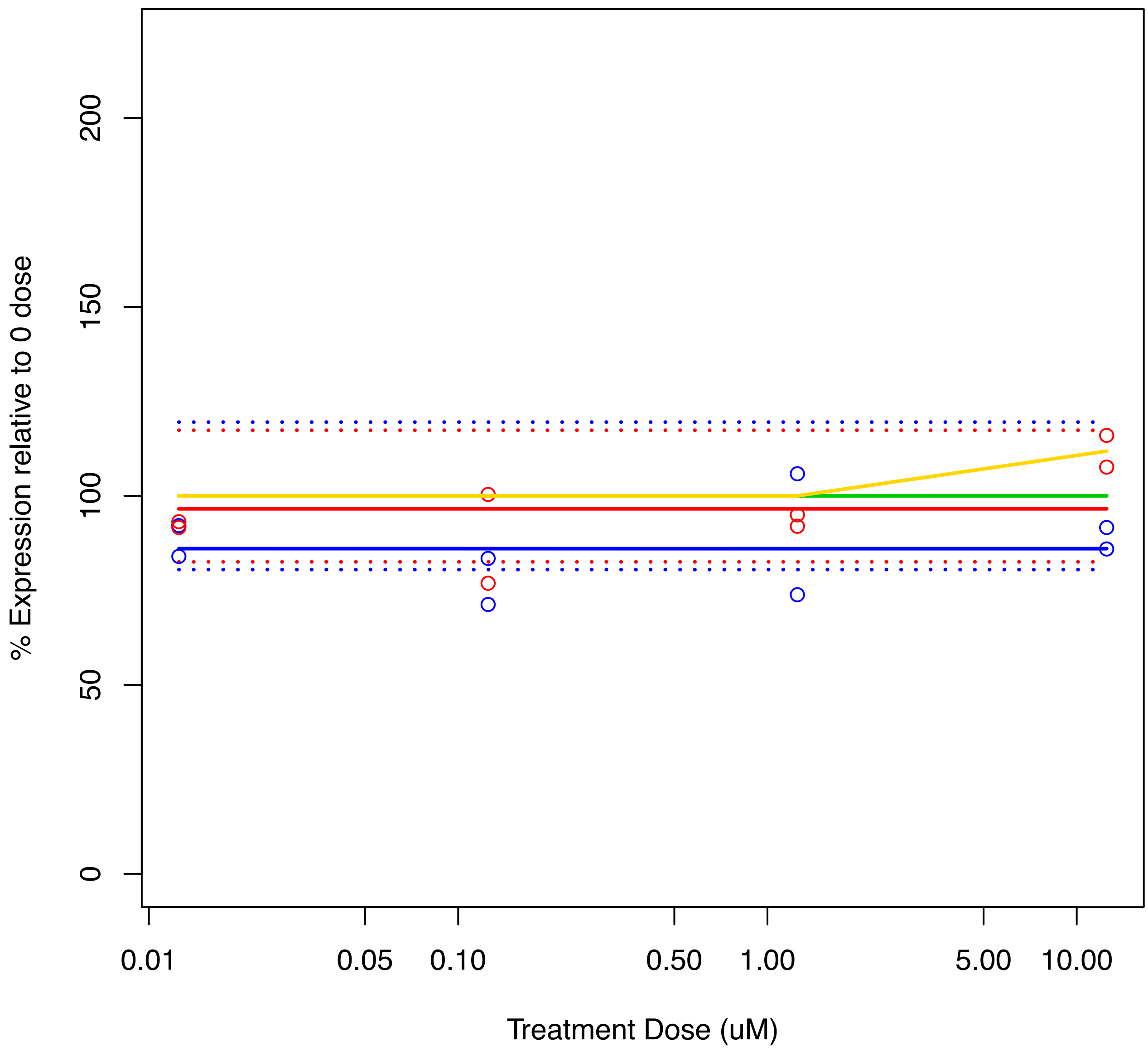

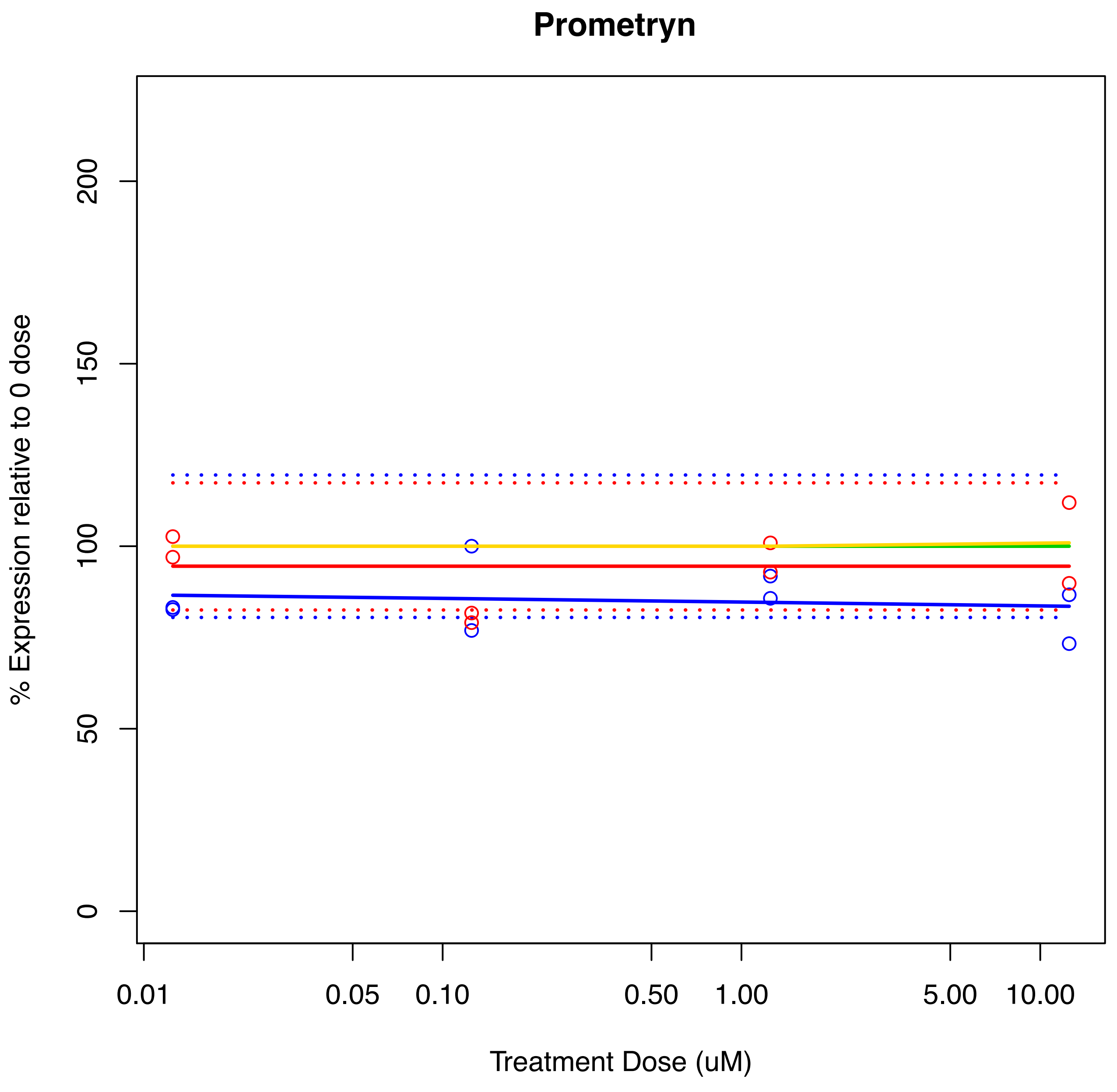

Prometryn
% Expression relative to 0 dose
0
50
100
150
200
0.01
0.05
0.10
0.50
1.00
5.00
10.00
Treatment Dose (uM)

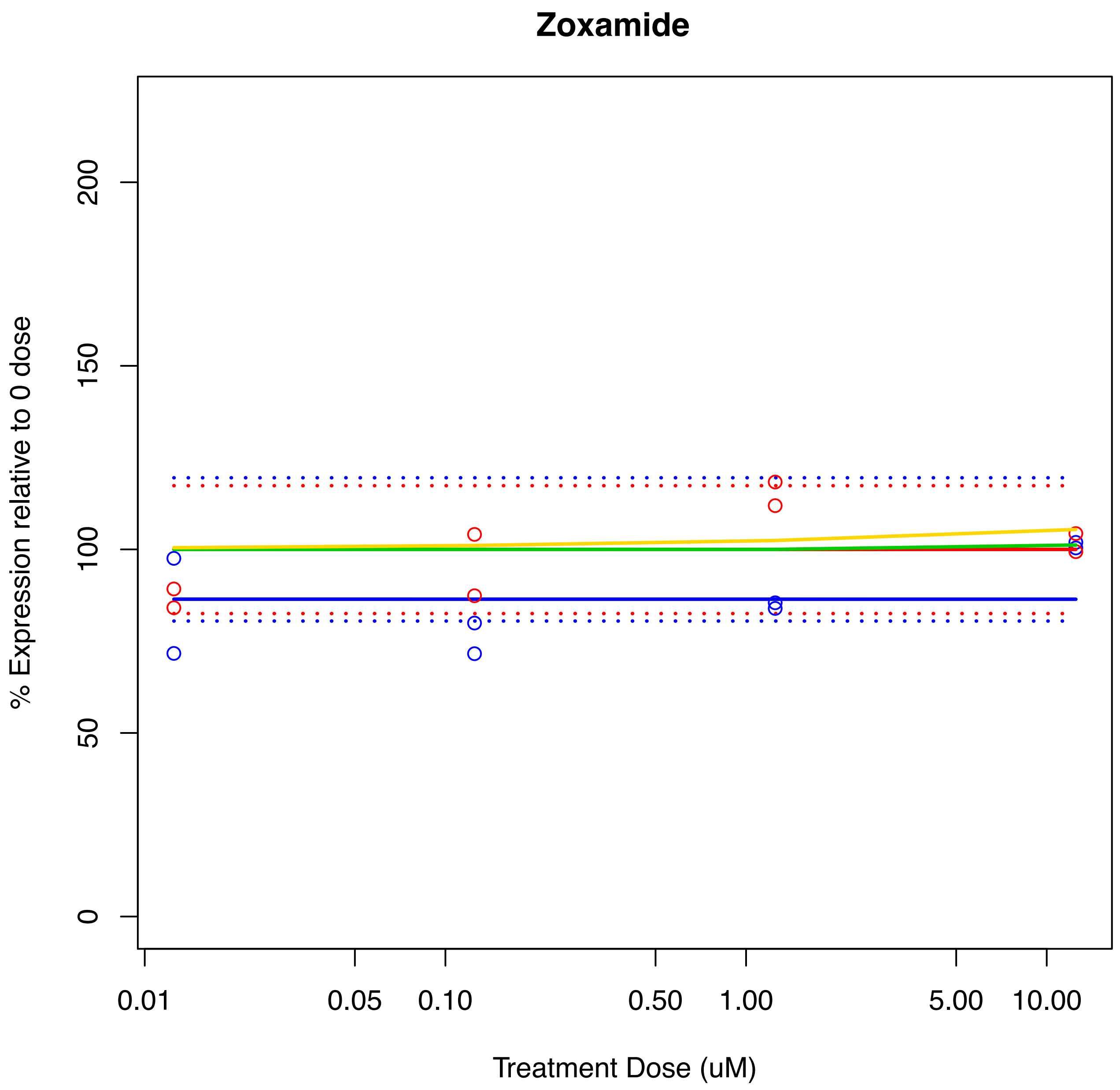
Zoxamide
% Expression relative to 0 dose
0
50
100
150
200
0.01
0.05
0.10
0.50
1.00
5.00
10.00
Treatment Dose (uM)

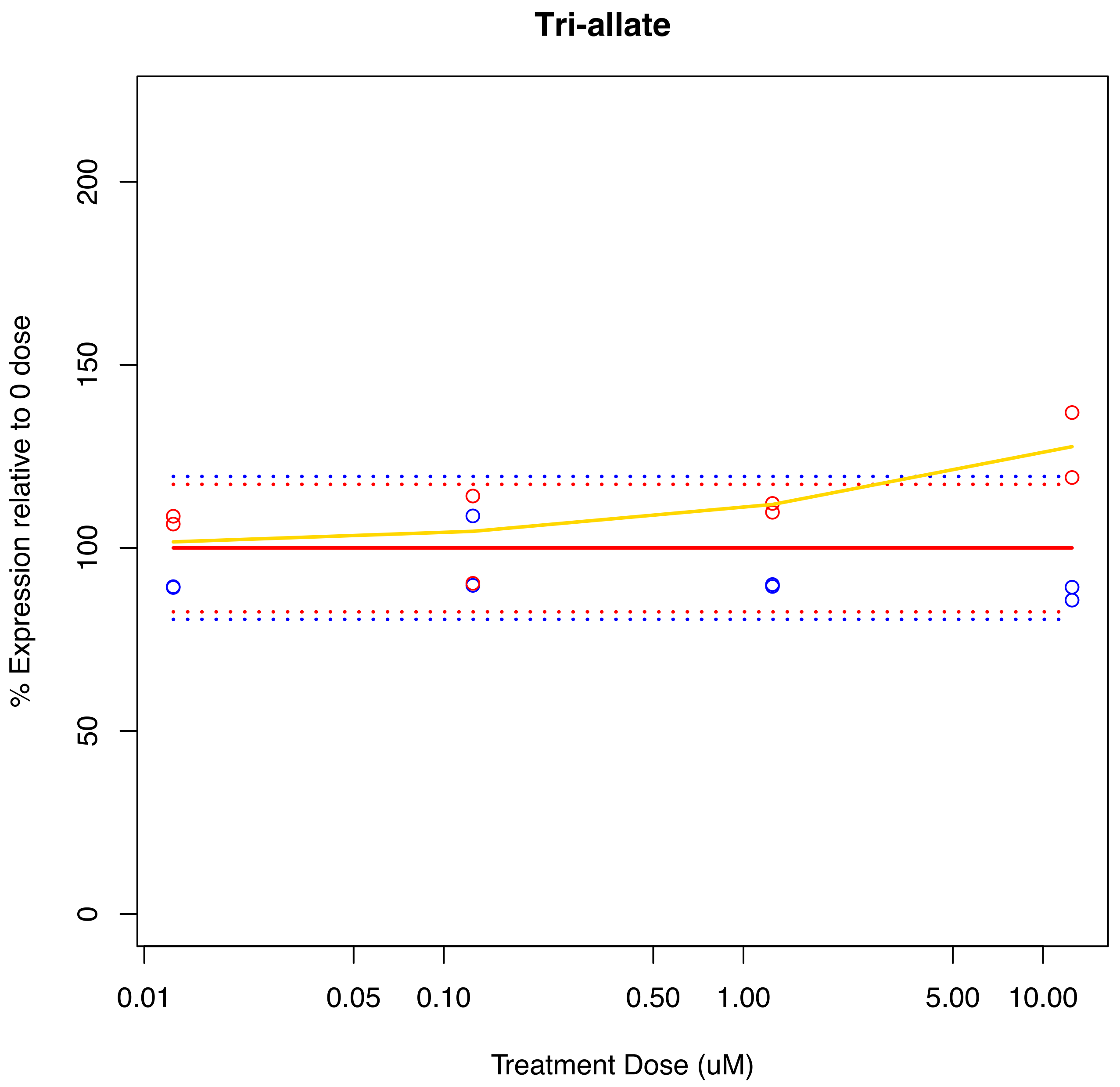

Tri-allate
% Expression relative to 0 dose
0
50
100
150
200
0.01
0.05
0.10
0.50
1.00
5.00
10.00
Treatment Dose (uM)

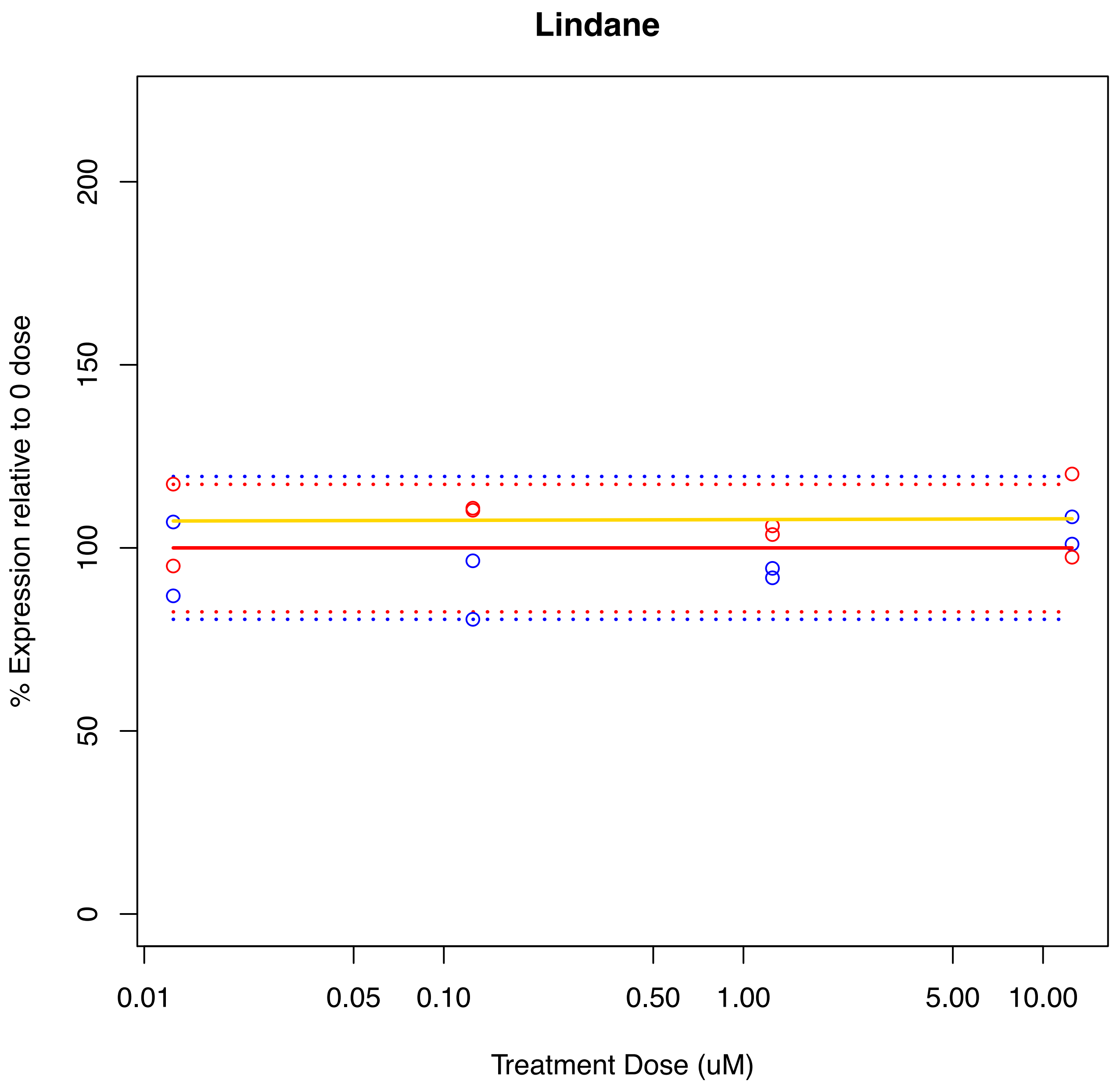
Lindane
% Expression relative to 0 dose
0
50
100
150
200
0.01
0.05
0.10
0.50
1.00
5.00
10.00
Treatment Dose (uM)

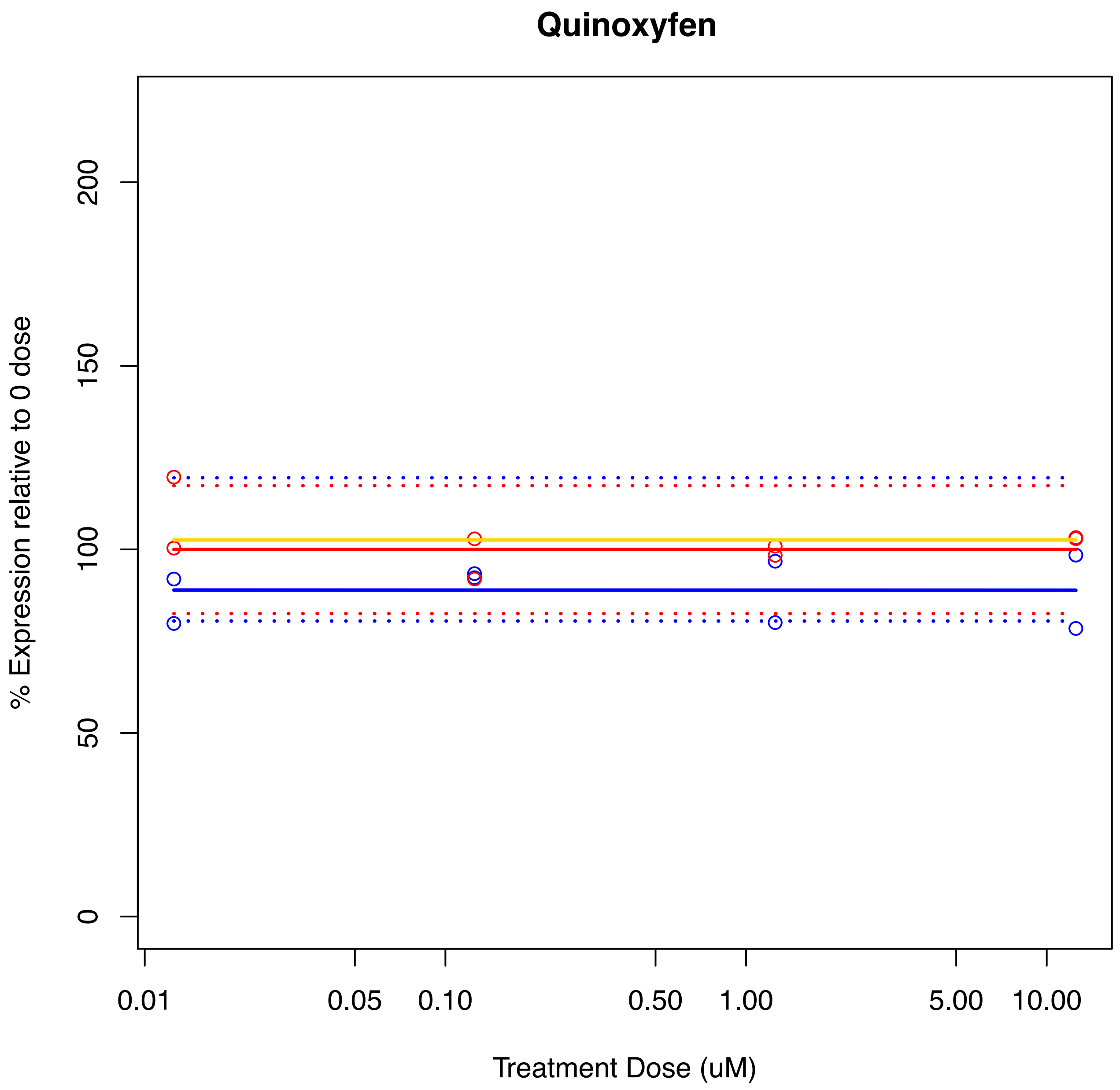
Quinoxyfen
% Expression relative to 0 dose
0
50
100
150
200
0.01
0.05
0.10
0.50
1.00
5.00
10.00
Treatment Dose (uM)

1H-Pyrazole-5-carboxamide,4-chloro-N-((4-(1,1-dimethylethyl)phenyl)methyl)-3-ethyl-1-methyl-

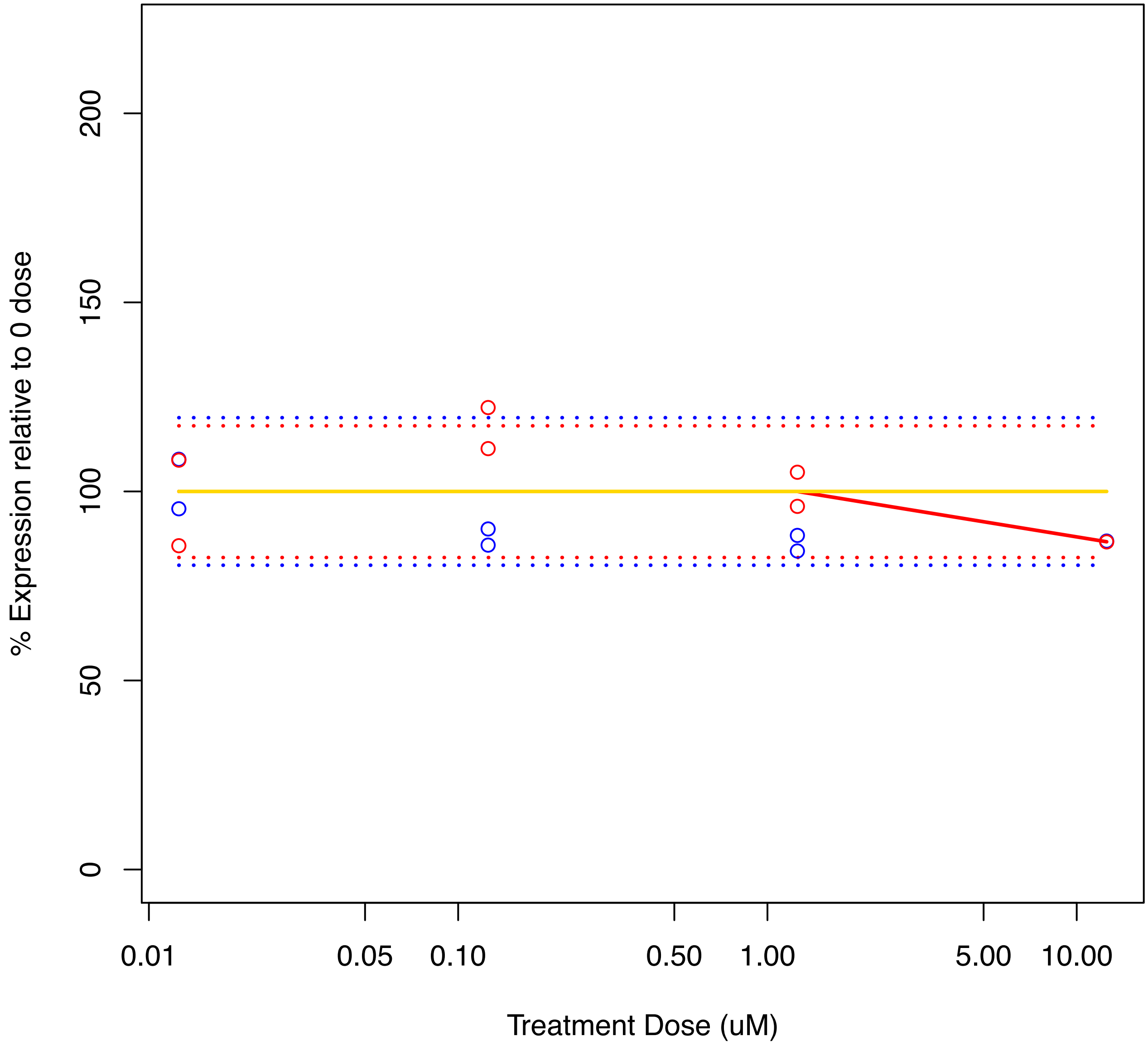

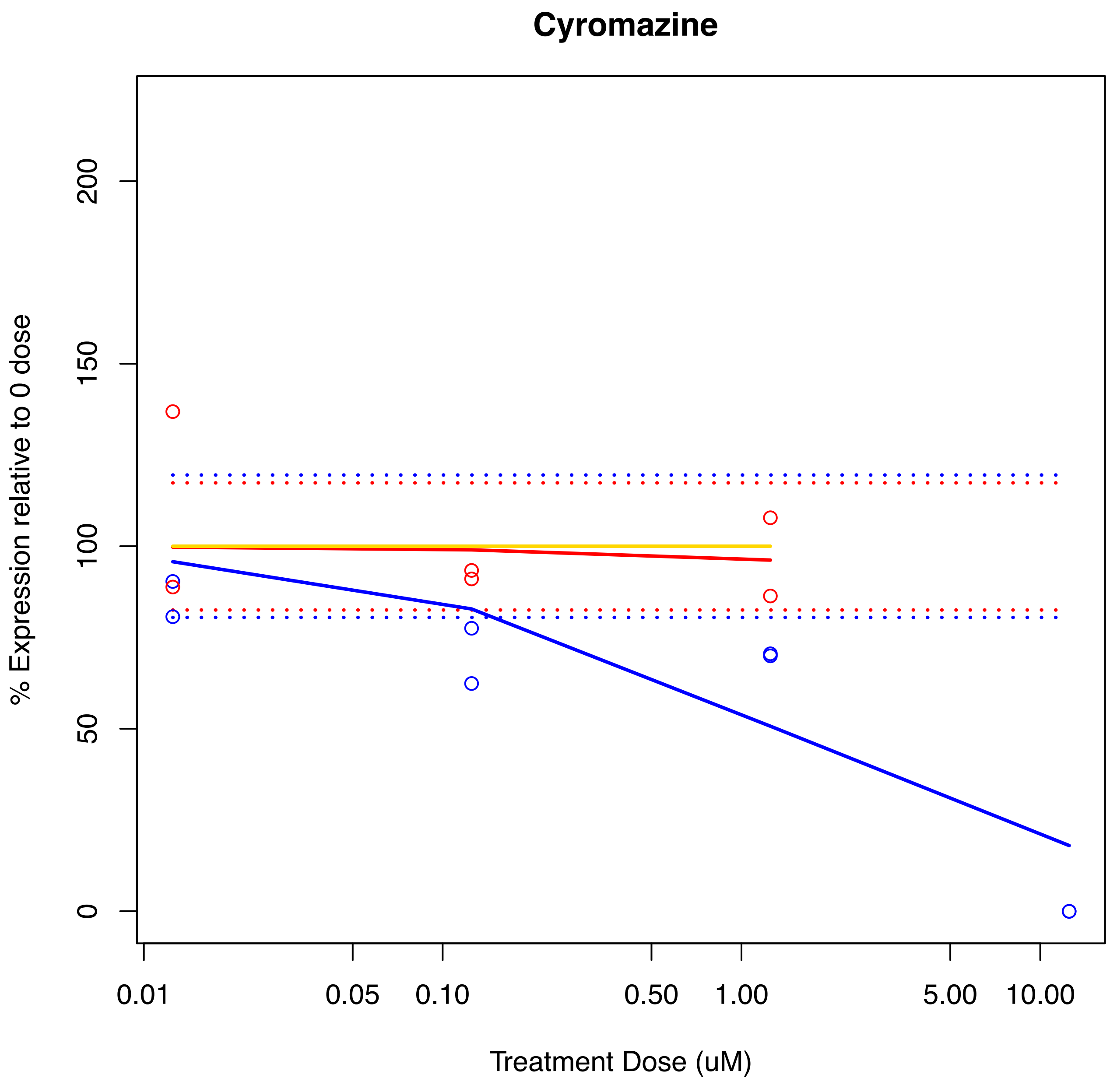
Cyromazine
% Expression relative to 0 dose
0
50
100
150
200
0.01
0.05
0.10
0.50
1.00
5.00
10.00
Treatment Dose (uM)

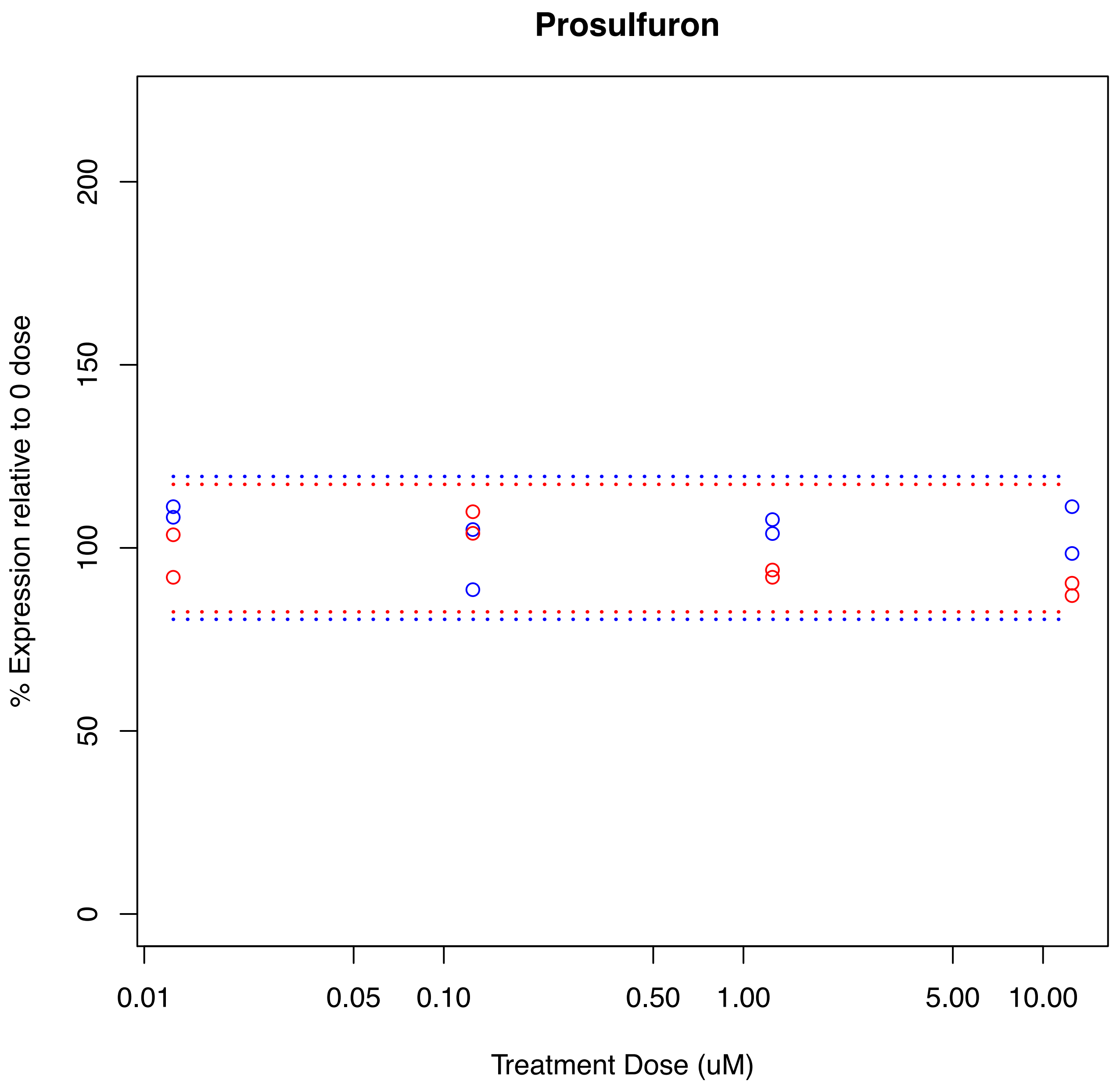

Prosulfuron
% Expression relative to 0 dose
0
50
100
150
200
0.01
0.05
0.10
0.50
1.00
5.00
10.00
Treatment Dose (uM)

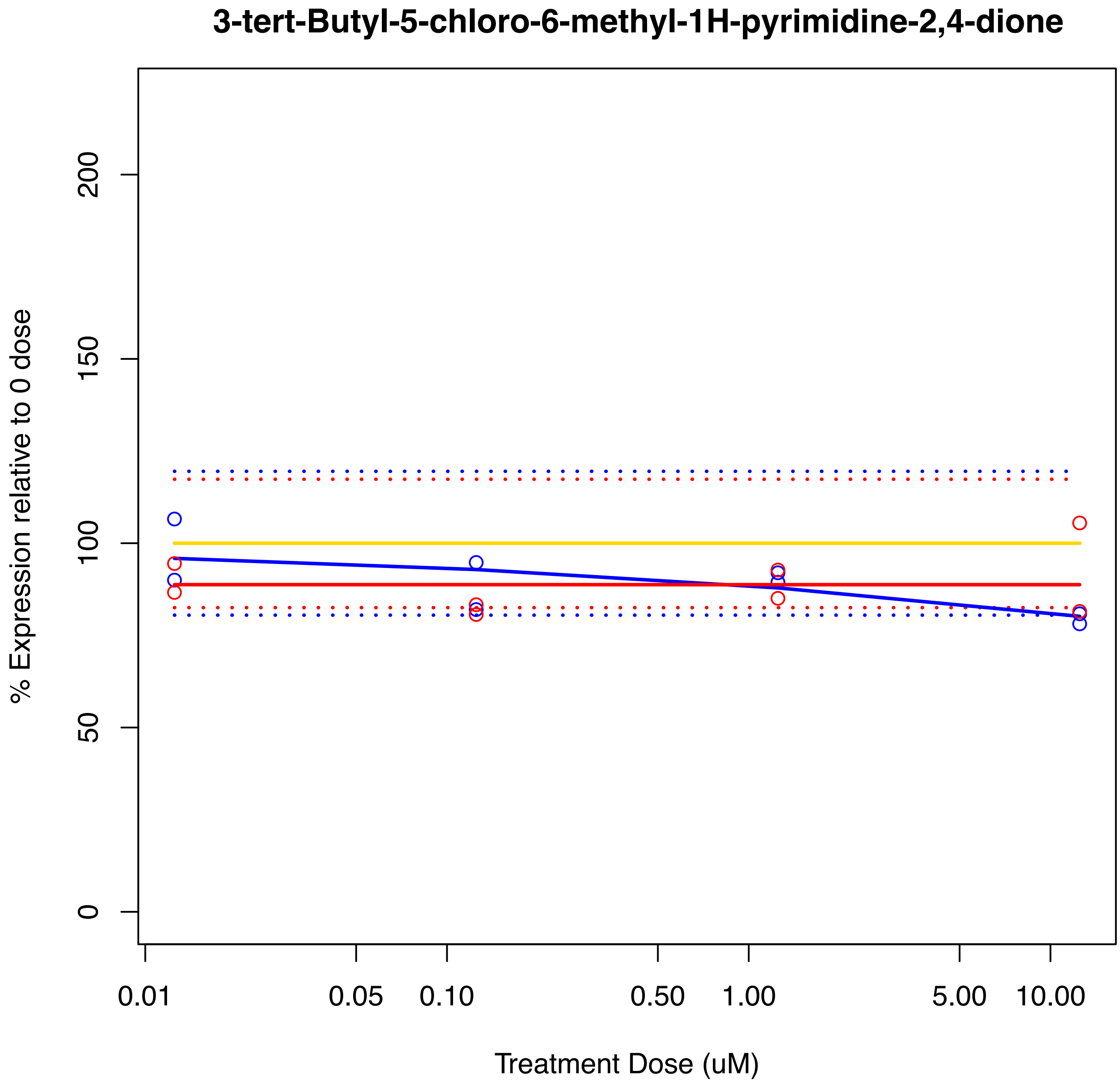
3-tert-Butyl-5-chloro-6-methyl-1H-pyrimidine-2,4-dione
% Expression relative to 0 dose
0
50
100
150
200
0.01
0.05
0.10
0.50
1.00
5.00
10.00
Treatment Dose (uM)

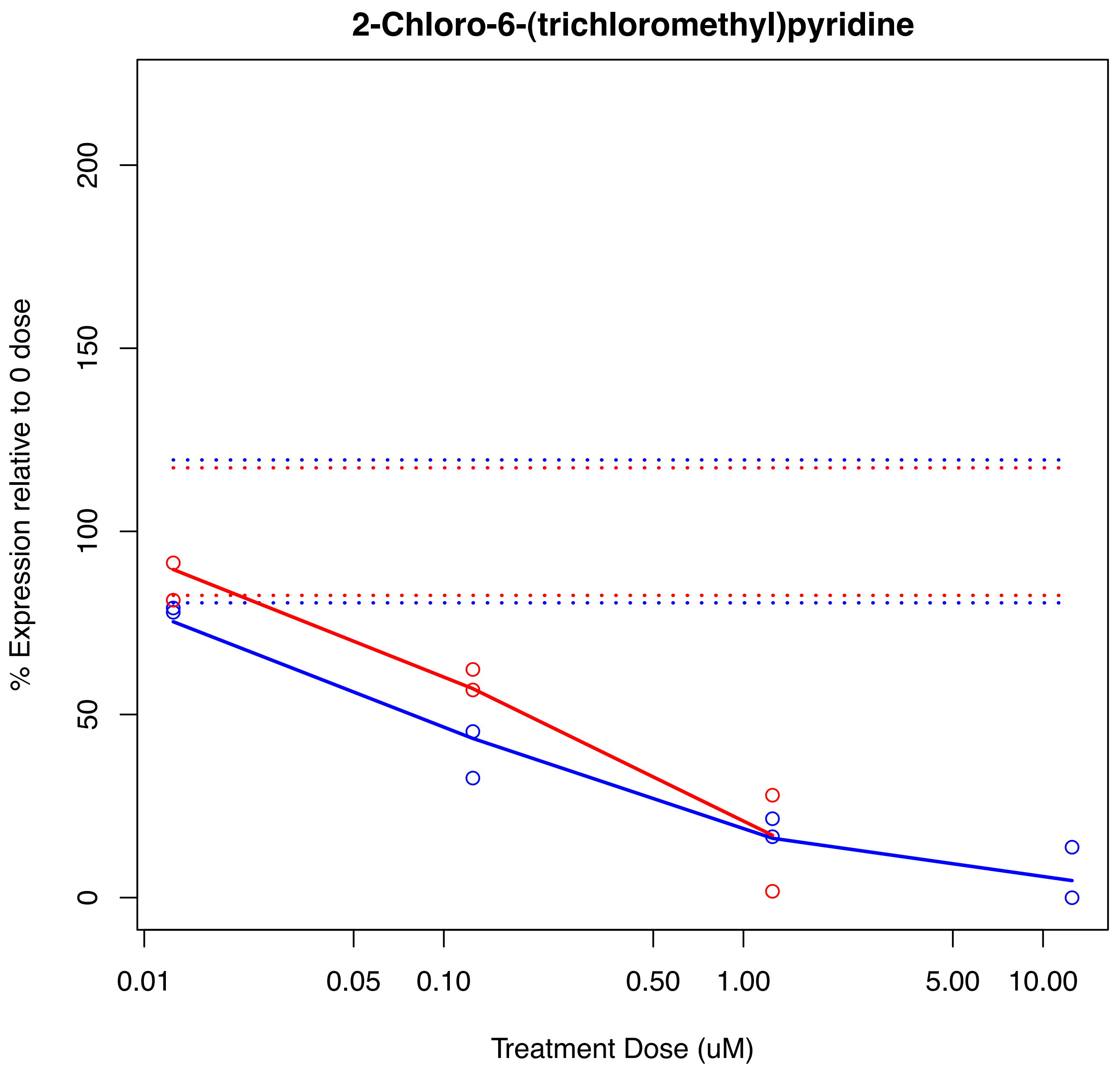
2-Chloro-6-(trichloromethyl)pyridine
% Expression relative to 0 dose
0
50
100
150
200
0.01
0.05
0.10
0.50
1.00
5.00
10.00
Treatment Dose (uM)

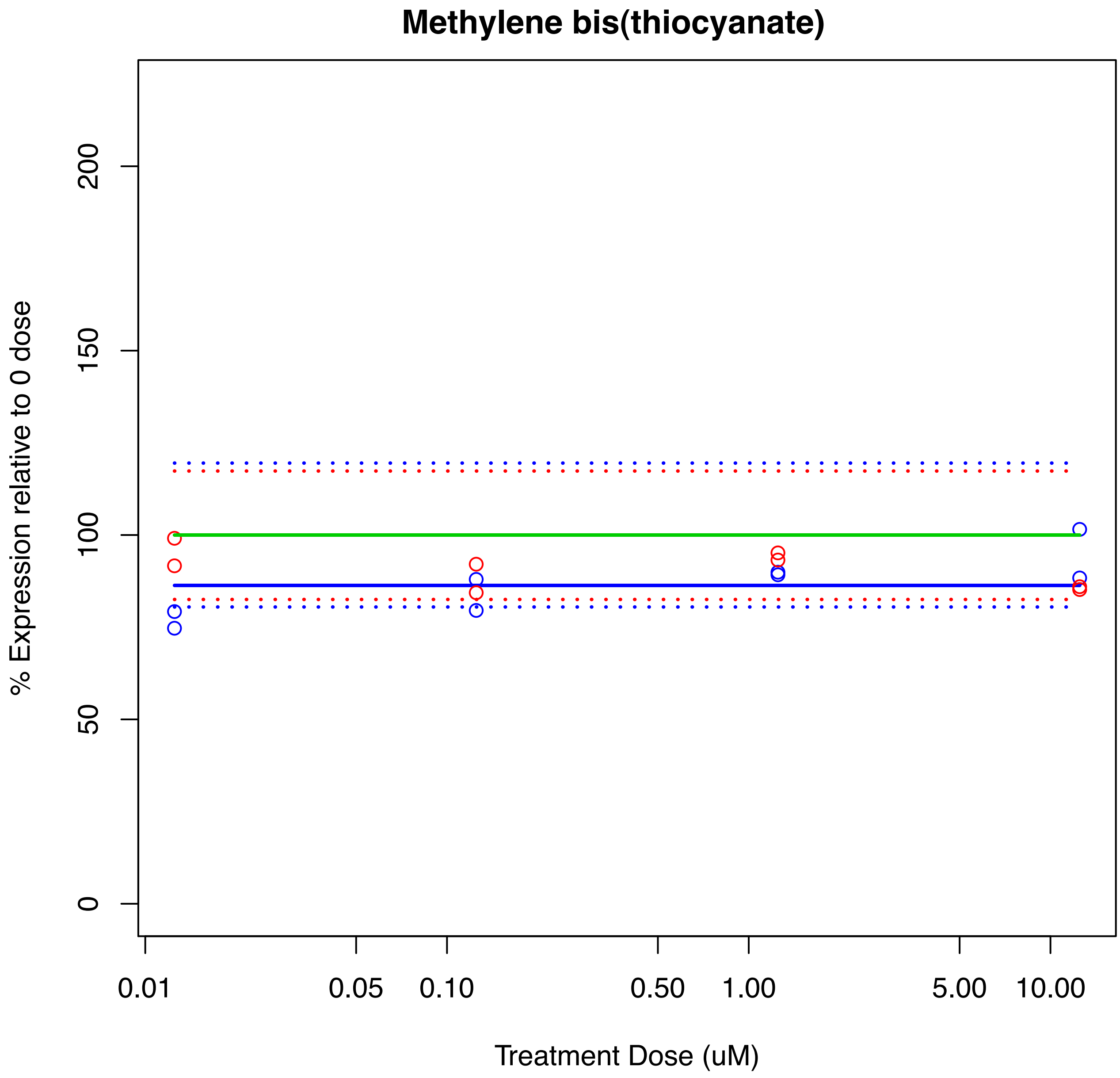

Methylene bis(thiocyanate)
% Expression relative to 0 dose
0
50
100
150
200
0.01
0.05
0.10
0.50
1.00
5.00
10.00
Treatment Dose (uM)

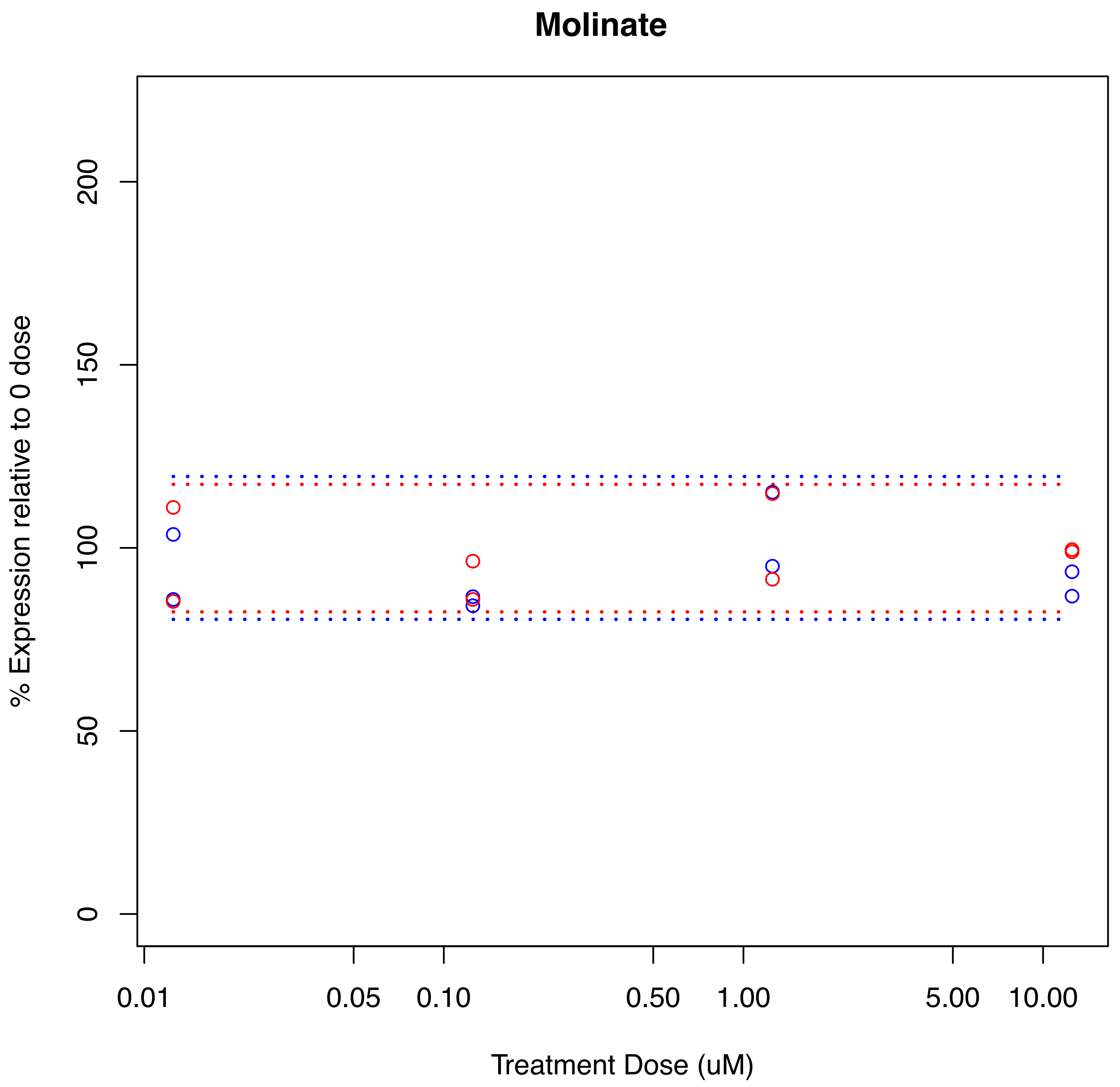
Molinate
% Expression relative to 0 dose
0
50
100
150
200
0.01
0.05
0.10
0.50
1.00
5.00
10.00
Treatment Dose (uM)

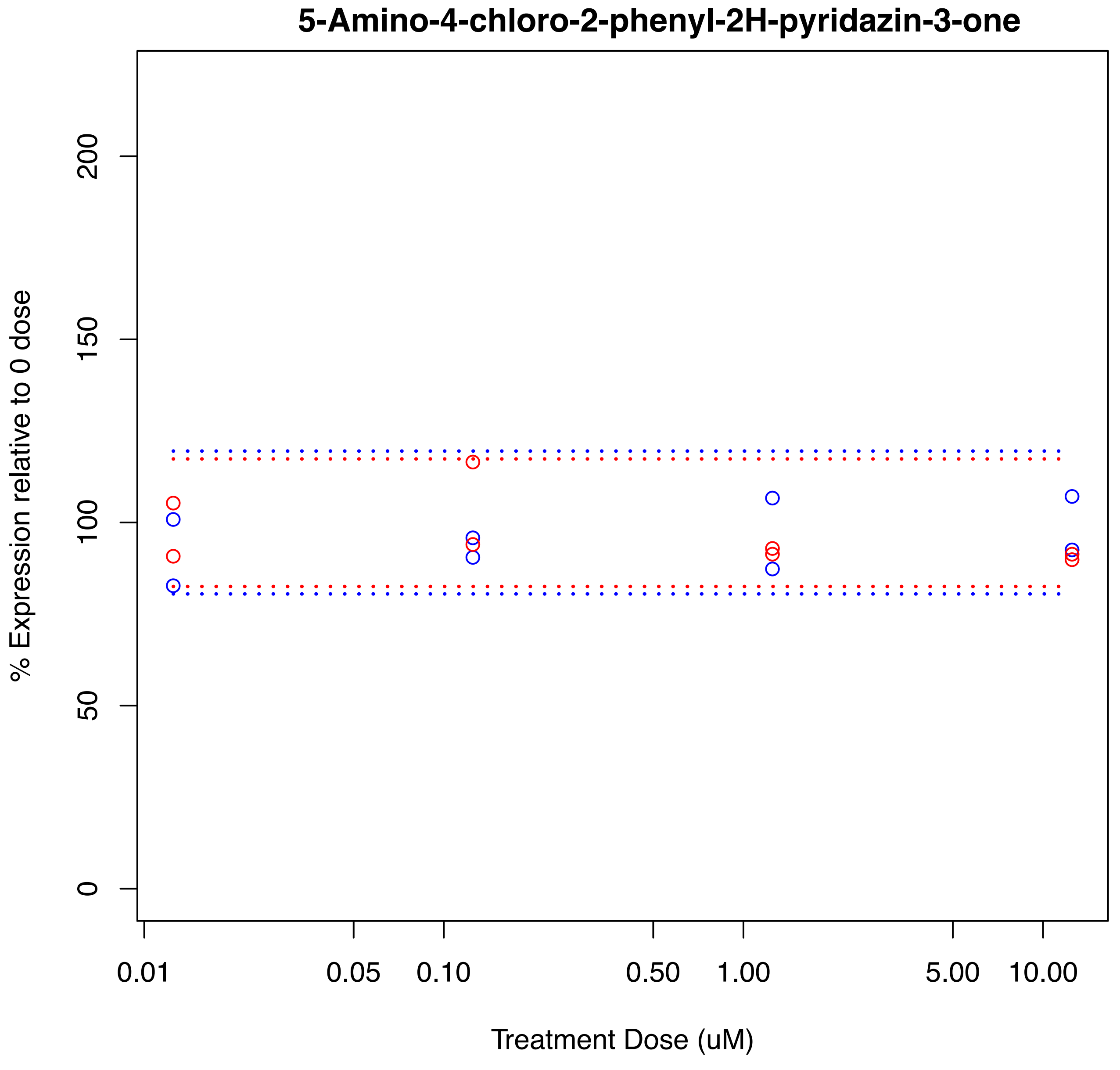
5-Amino-4-chloro-2-phenyl-2H-pyridazin-3-one
% Expression relative to 0 dose
0
50
100
150
200
0.01
0.05
0.10
0.50
1.00
5.00
10.00
Treatment Dose (uM)

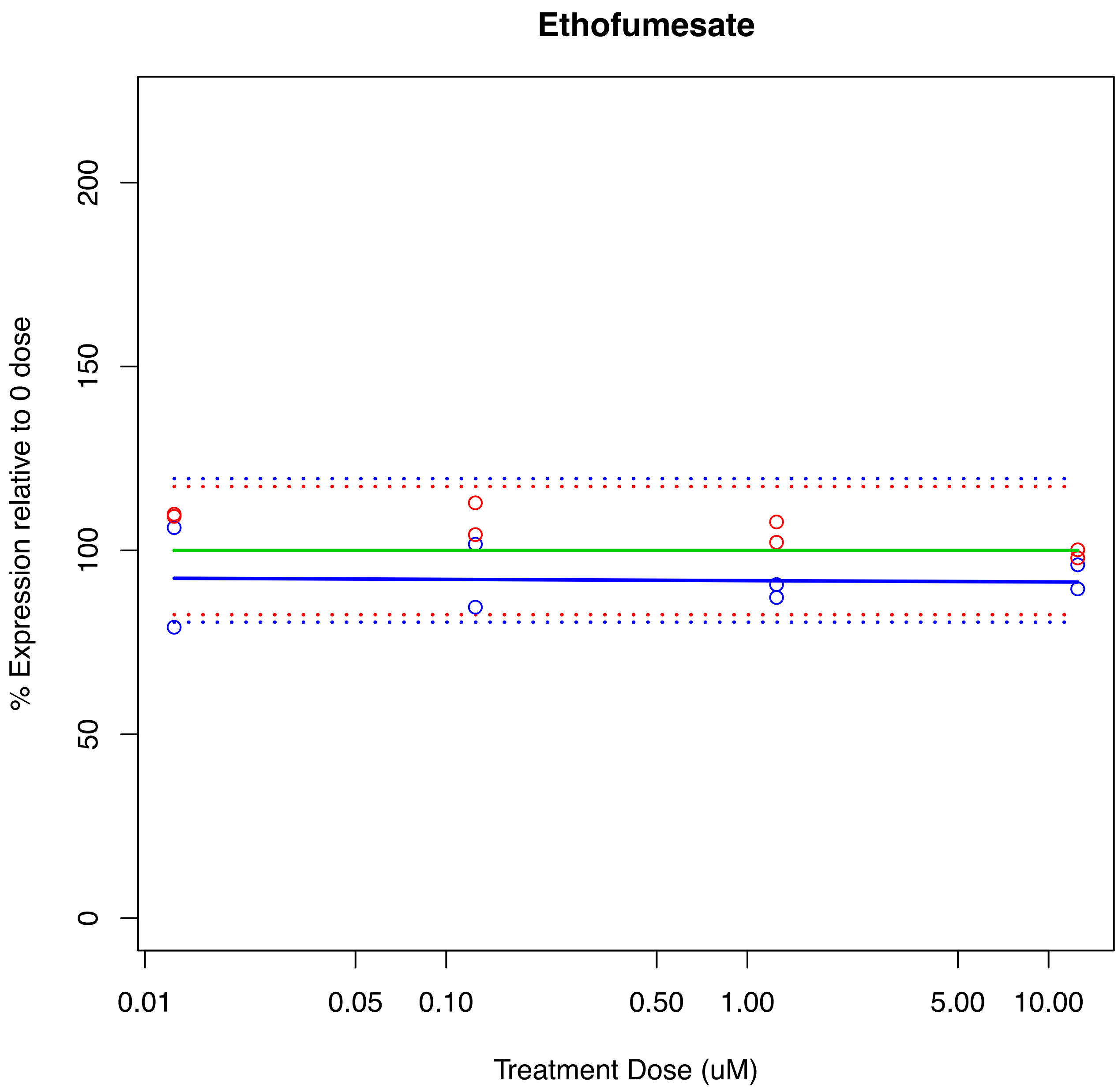

Ethofumesate
% Expression relative to 0 dose
0
50
100
150
200
0.01
0.05
0.10
0.50
1.00
5.00
10.00
Treatment Dose (uM)

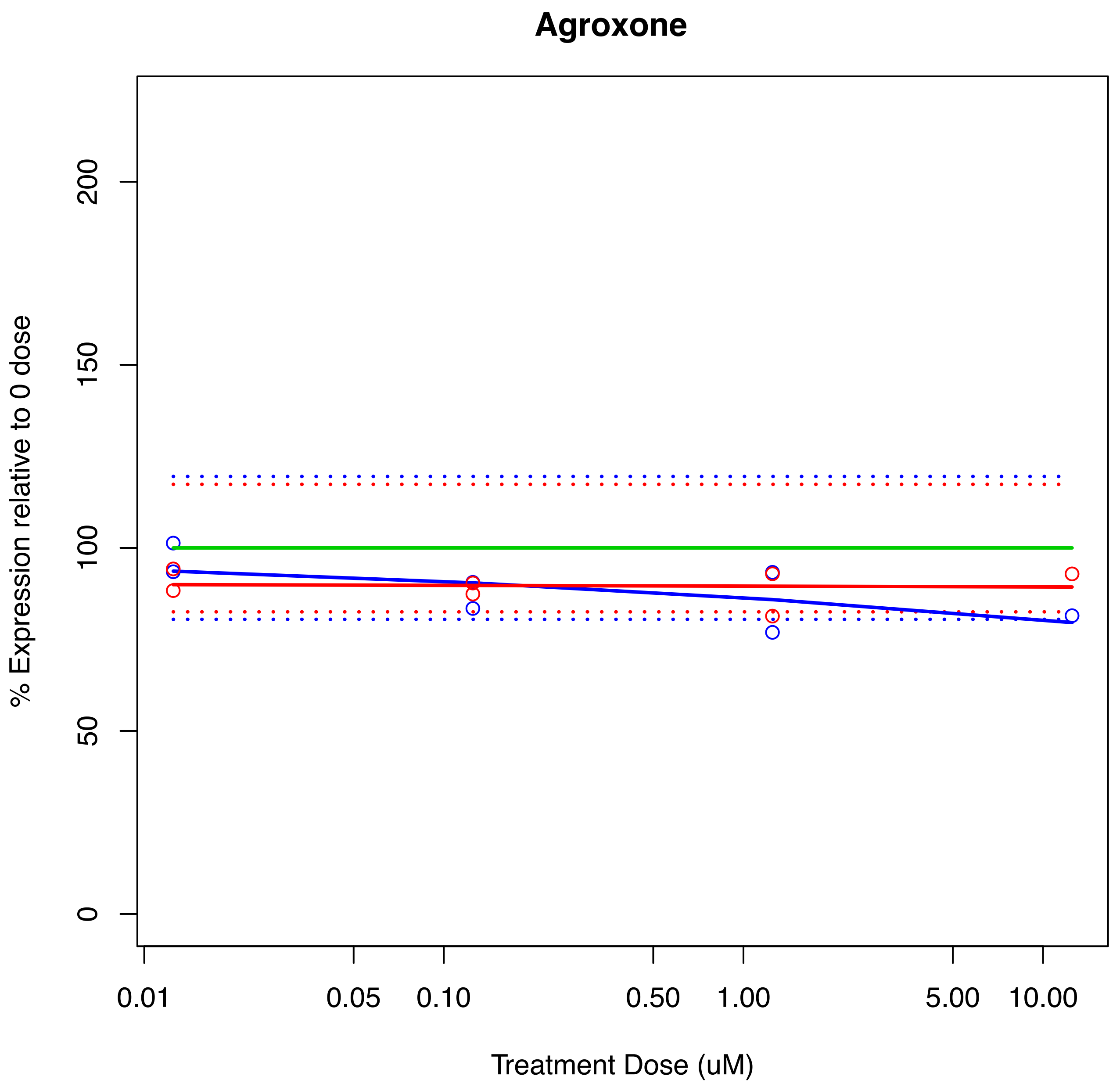
Agroxone
% Expression relative to 0 dose
0
50
100
150
200
0.01
0.05
0.10
0.50
1.00
5.00
10.00
Treatment Dose (uM)

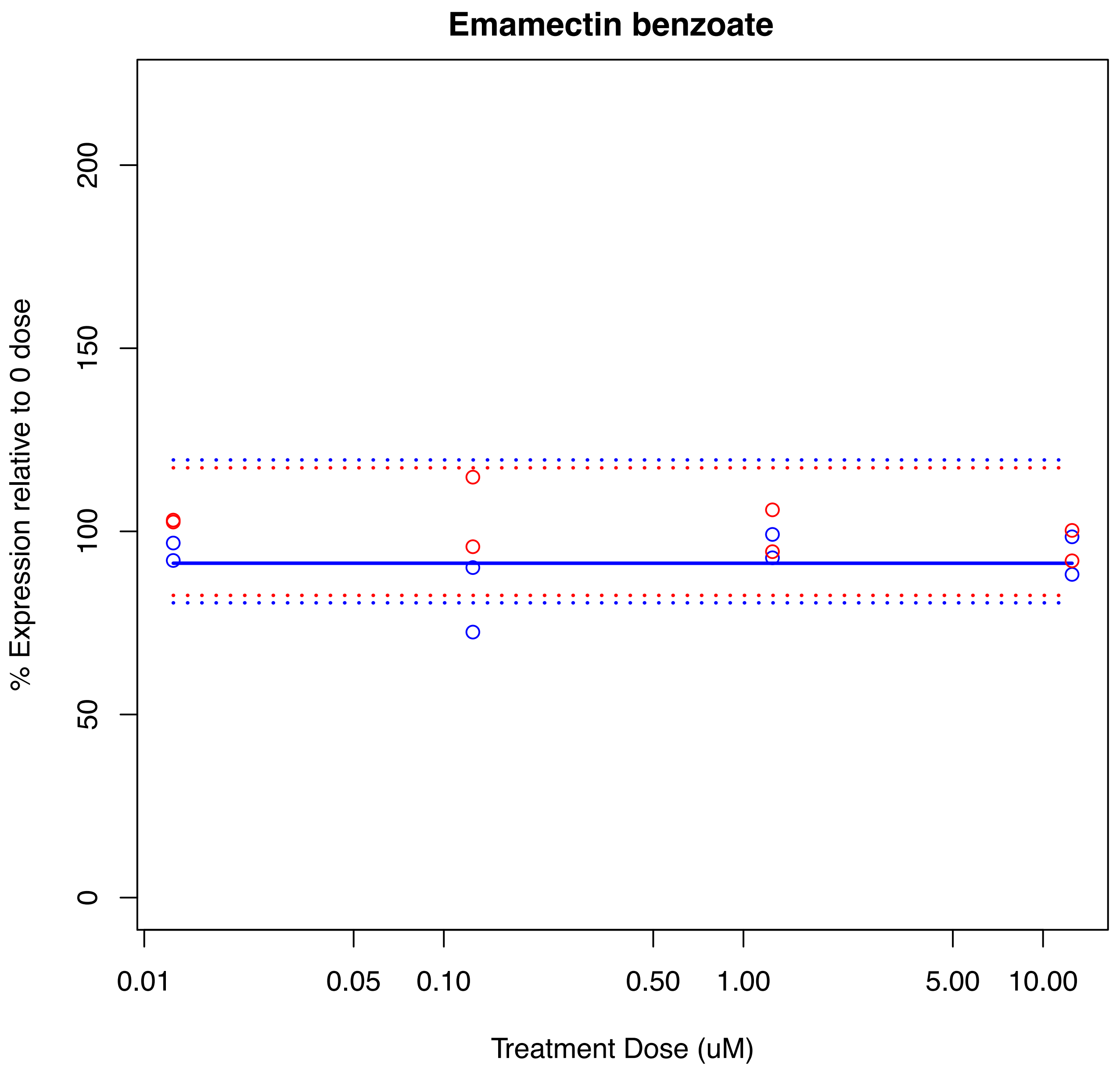
Emamectin benzoate
% Expression relative to 0 dose
0
50
100
150
200
0.01
0.05
0.10
0.50
1.00
5.00
10.00
Treatment Dose (uM)

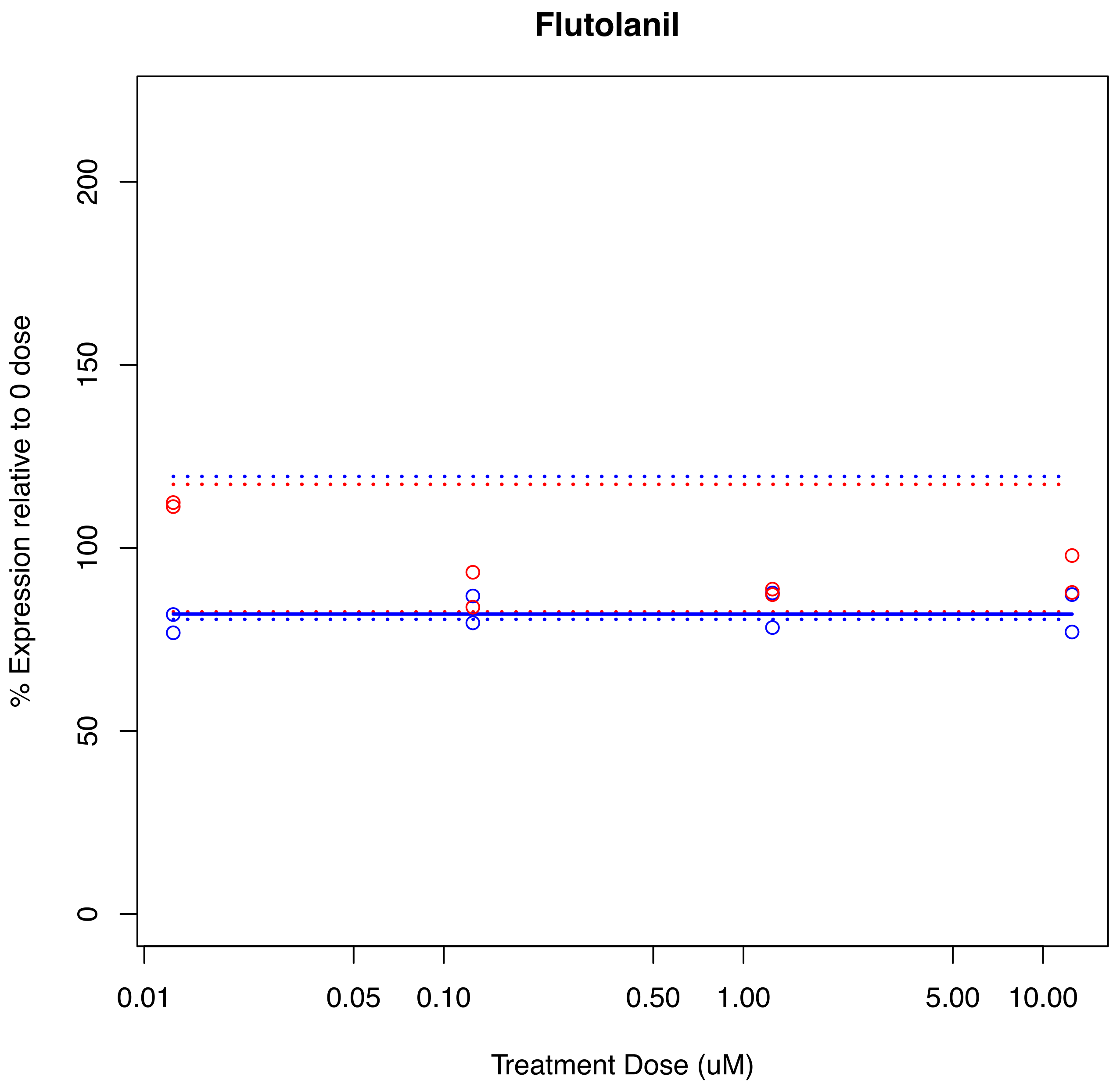

Flutolanil
% Expression relative to 0 dose
0
50
100
150
200
0.01
0.05
0.10
0.50
1.00
5.00
10.00
Treatment Dose (uM)

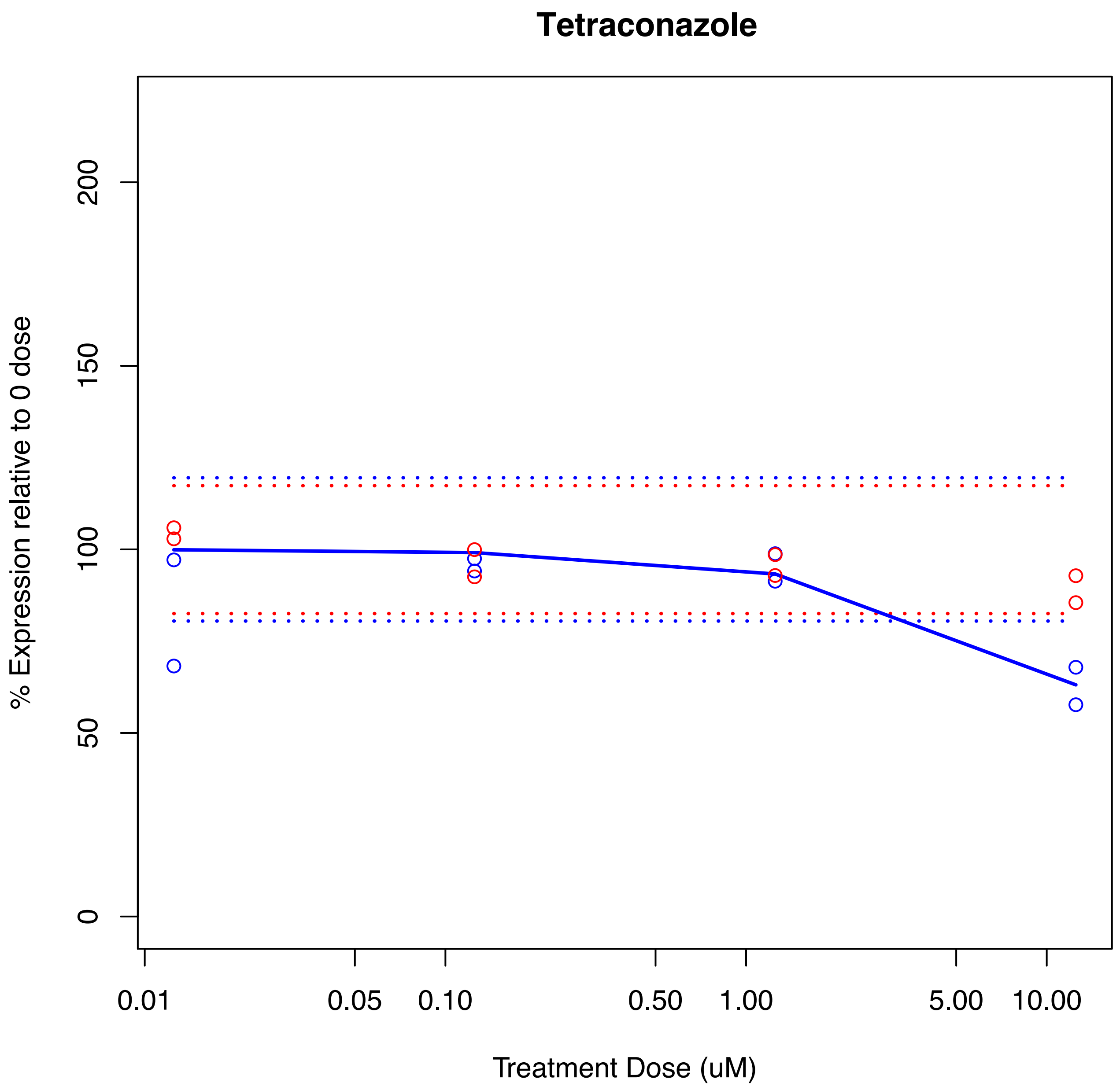

Tetraconazole
% Expression relative to 0 dose
0
50
100
150
200
0.01
0.05
0.10
0.50
1.00
5.00
10.00
Treatment Dose (uM)

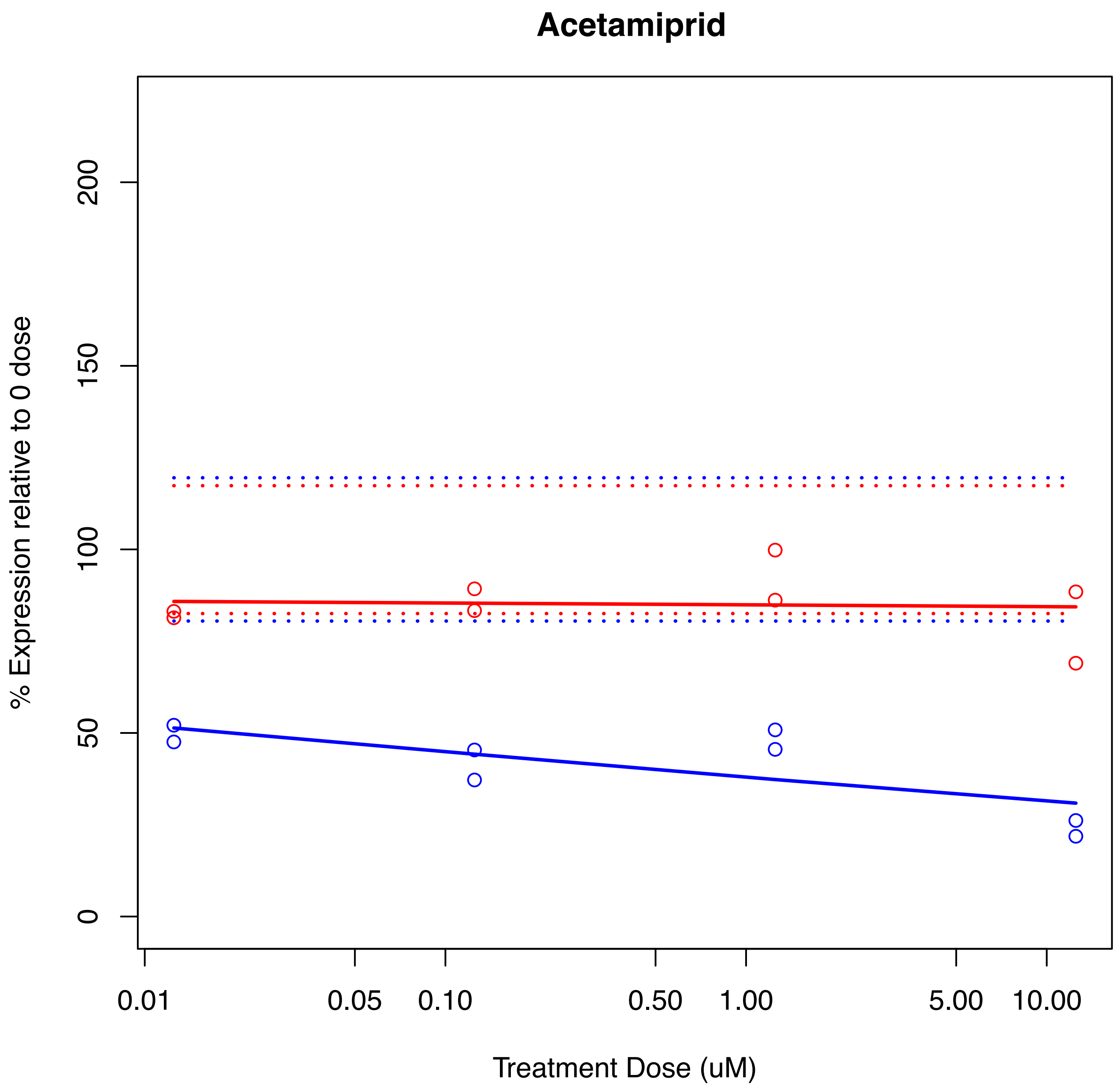

Acetamiprid
% Expression relative to 0 dose
0
50
100
150
200
0.01
0.05
0.10
0.50
1.00
5.00
10.00
Treatment Dose (uM)

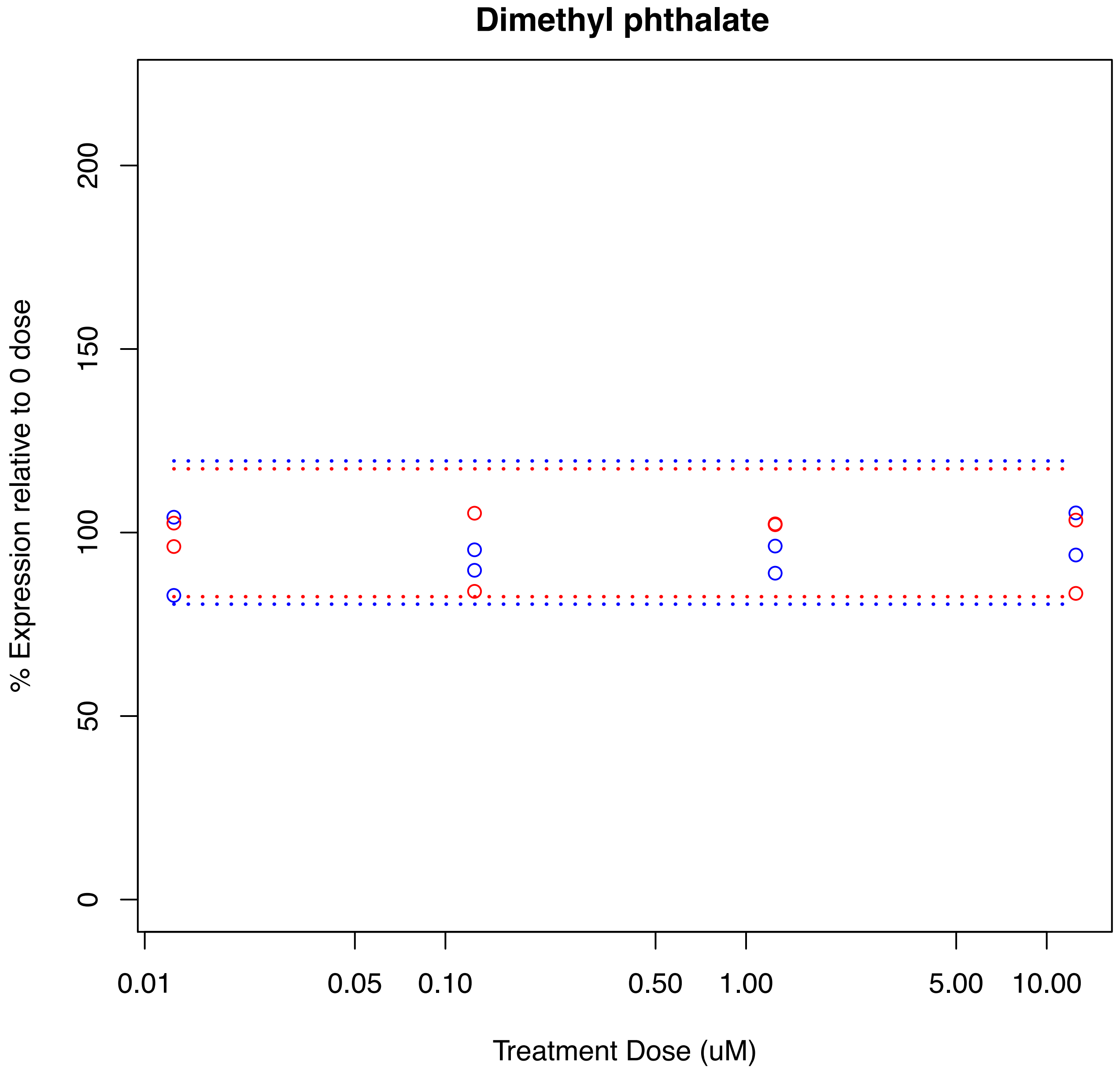
Dimethyl phthalate
% Expression relative to 0 dose
0
50
100
150
200
0.01
0.05
0.10
0.50
1.00
5.00
10.00
Treatment Dose (uM)

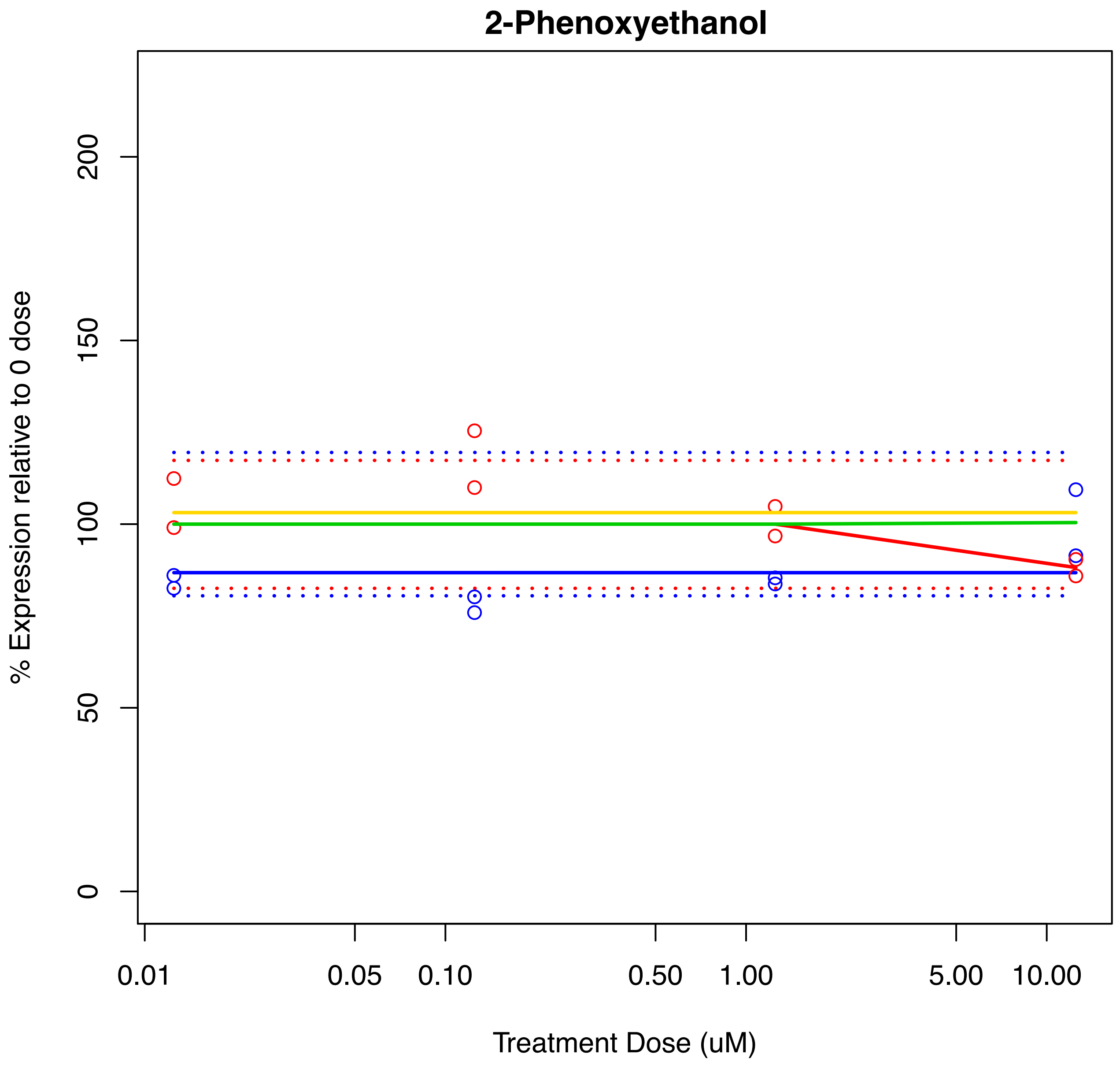
2-Phenoxyethanol
% Expression relative to 0 dose
0
50
100
150
200
0.01
0.05
0.10
0.50
1.00
5.00
10.00
Treatment Dose (uM)

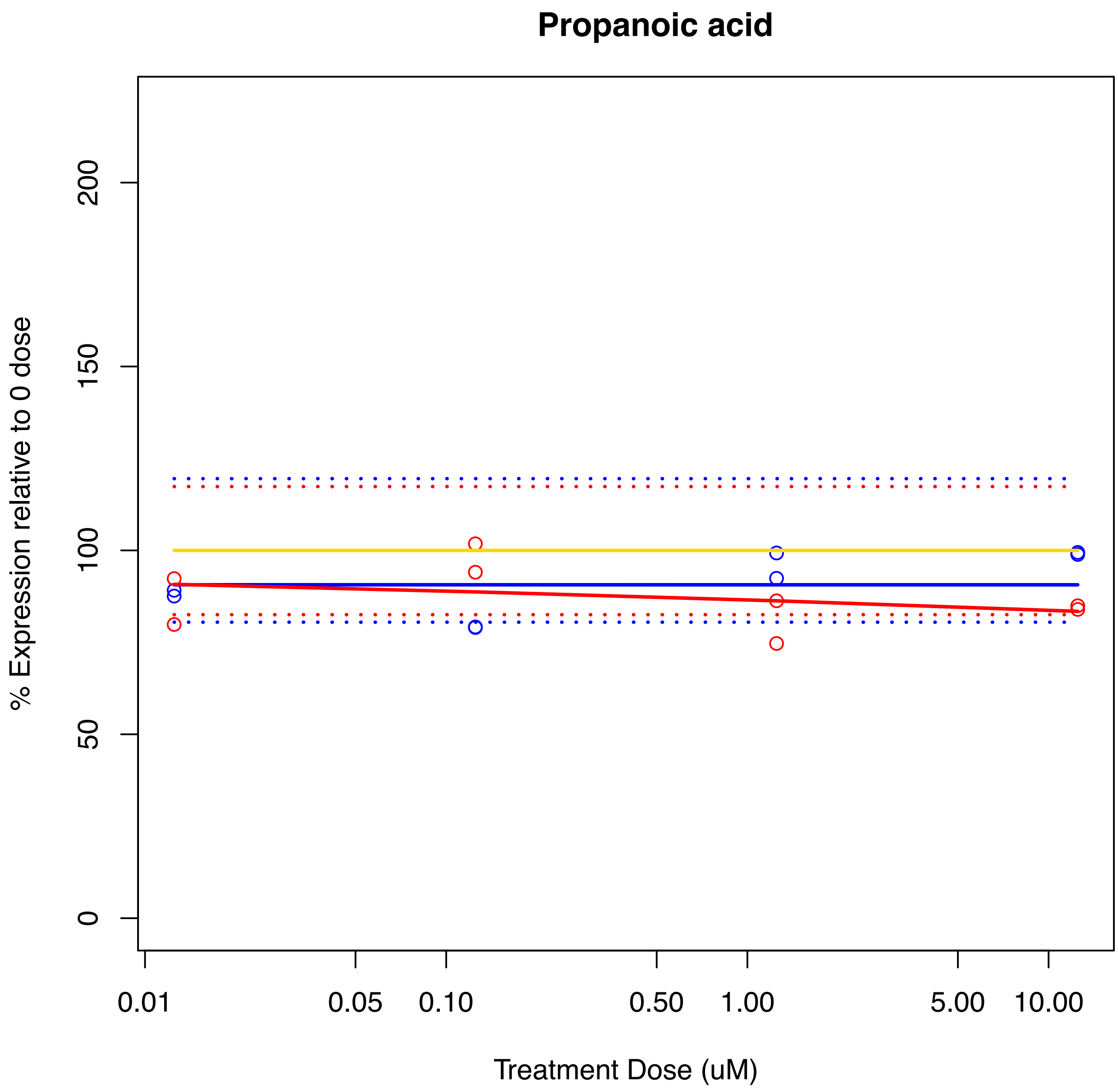
Propanoic acid
% Expression relative to 0 dose
0
50
100
150
200
0.01
0.05
0.10
0.50
1.00
5.00
10.00
Treatment Dose (uM)

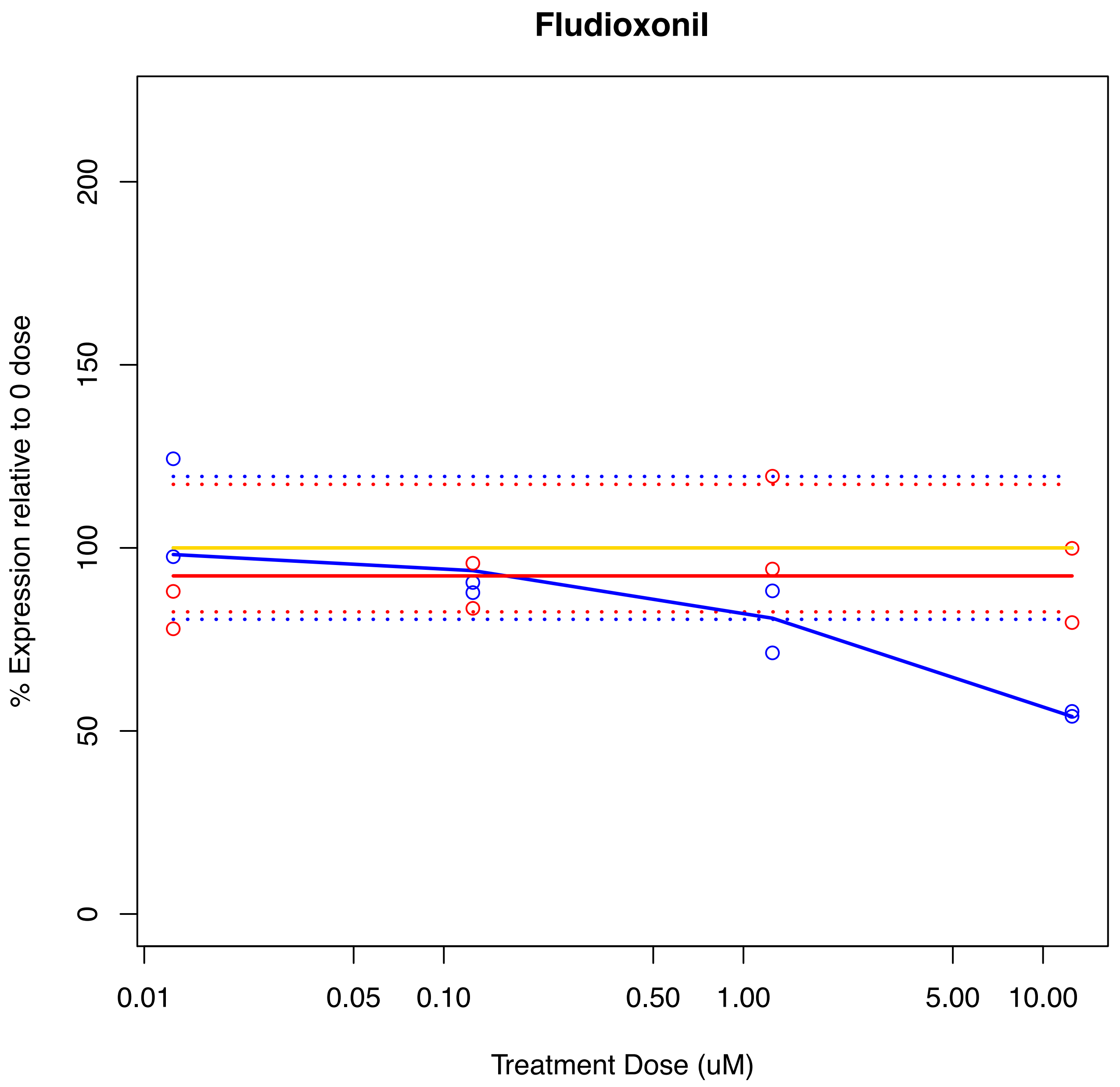
Fludioxonil
% Expression relative to 0 dose
0
50
100
150
200
0.01
0.05
0.10
0.50
1.00
5.00
10.00
Treatment Dose (uM)

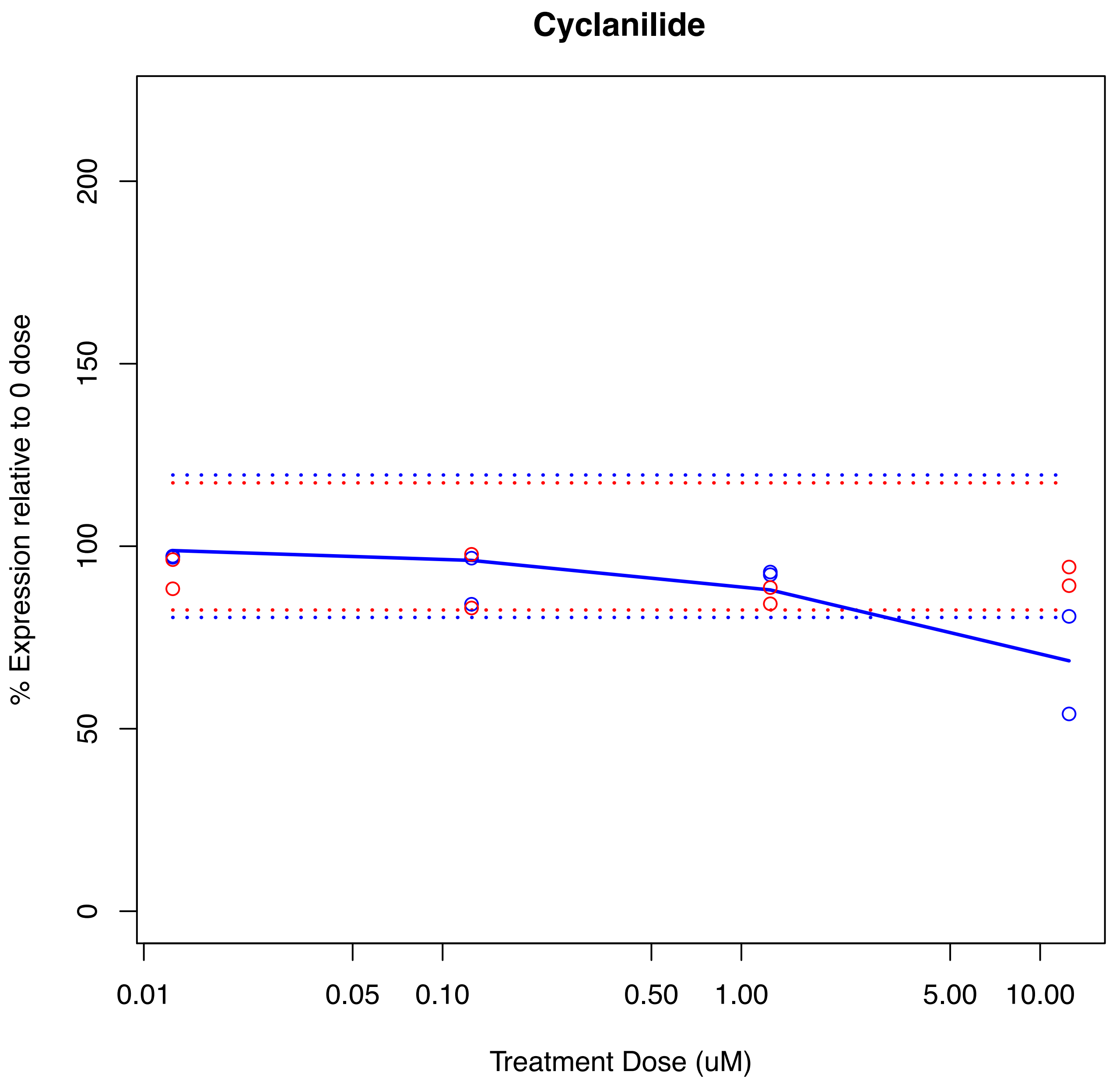
Cyclanilide
% Expression relative to 0 dose
0
50
100
150
200
0.01
0.05
0.10
0.50
1.00
5.00
10.00
Treatment Dose (uM)

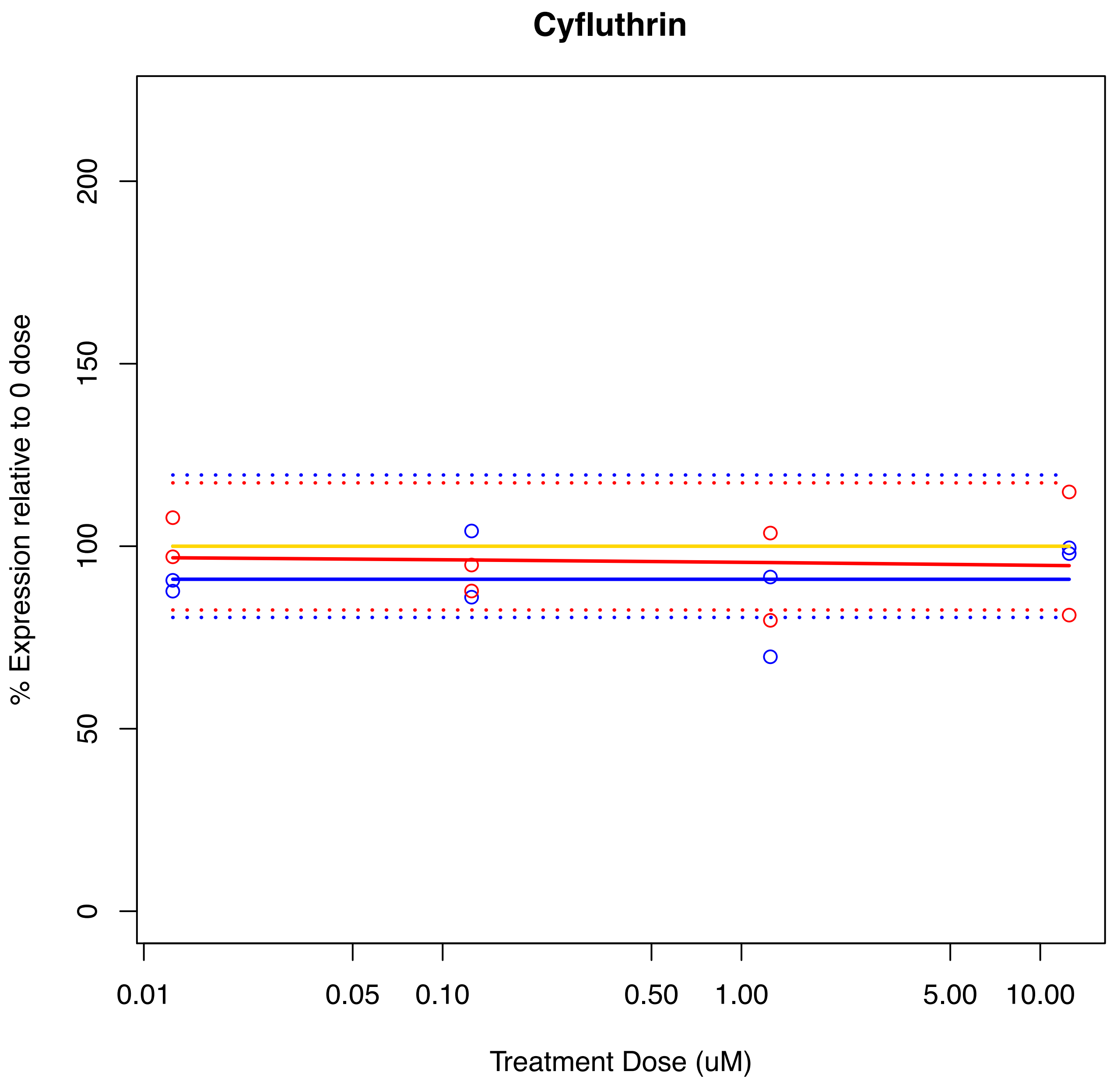
Cyfluthrin
% Expression relative to 0 dose
0
50
100
150
200
0.01
0.05
0.10
0.50
1.00
5.00
10.00
Treatment Dose (uM)

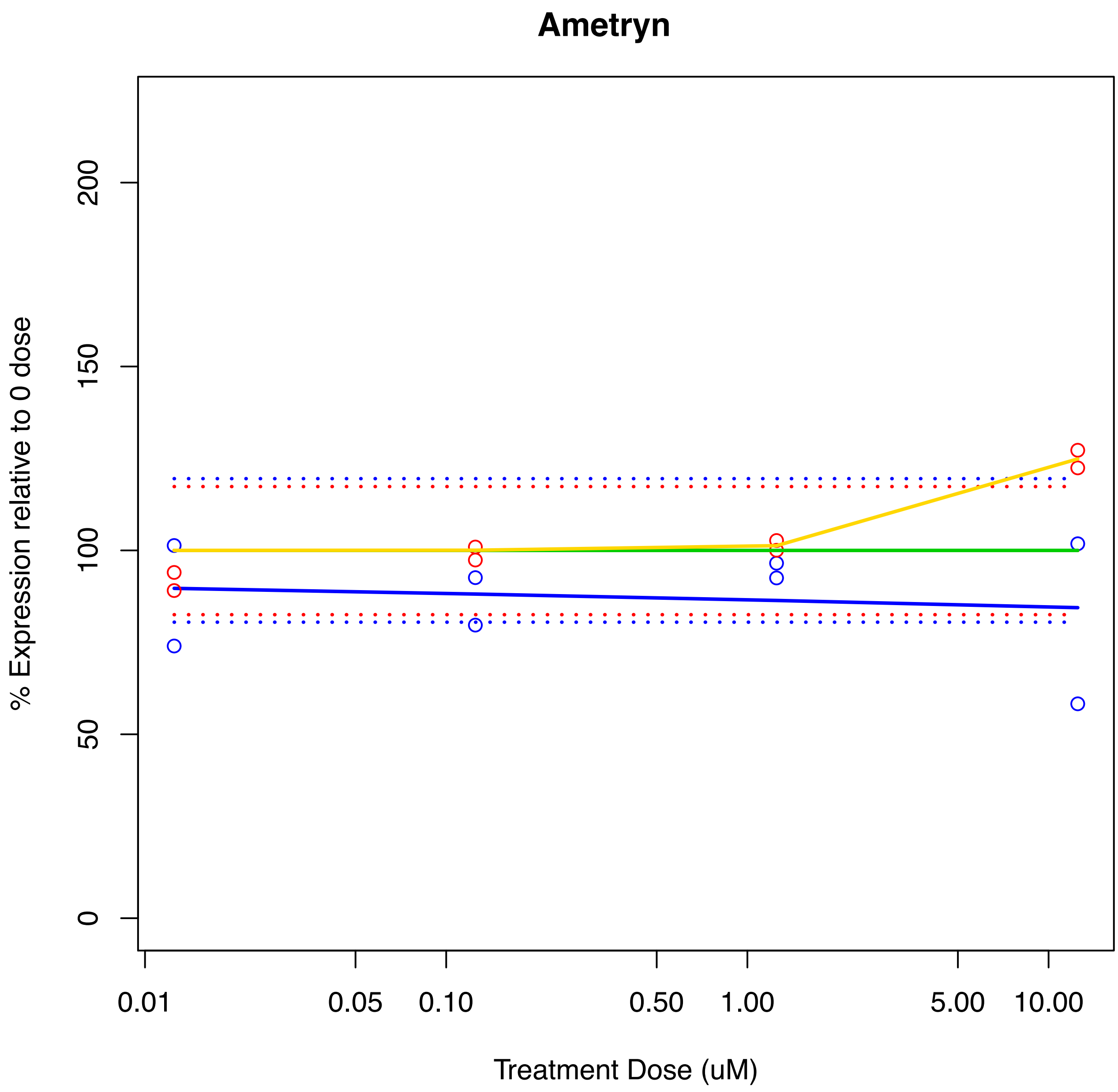

Ametryn
% Expression relative to 0 dose
0
50
100
150
200
0.01
0.05
0.10
0.50
1.00
5.00
10.00
Treatment Dose (uM)

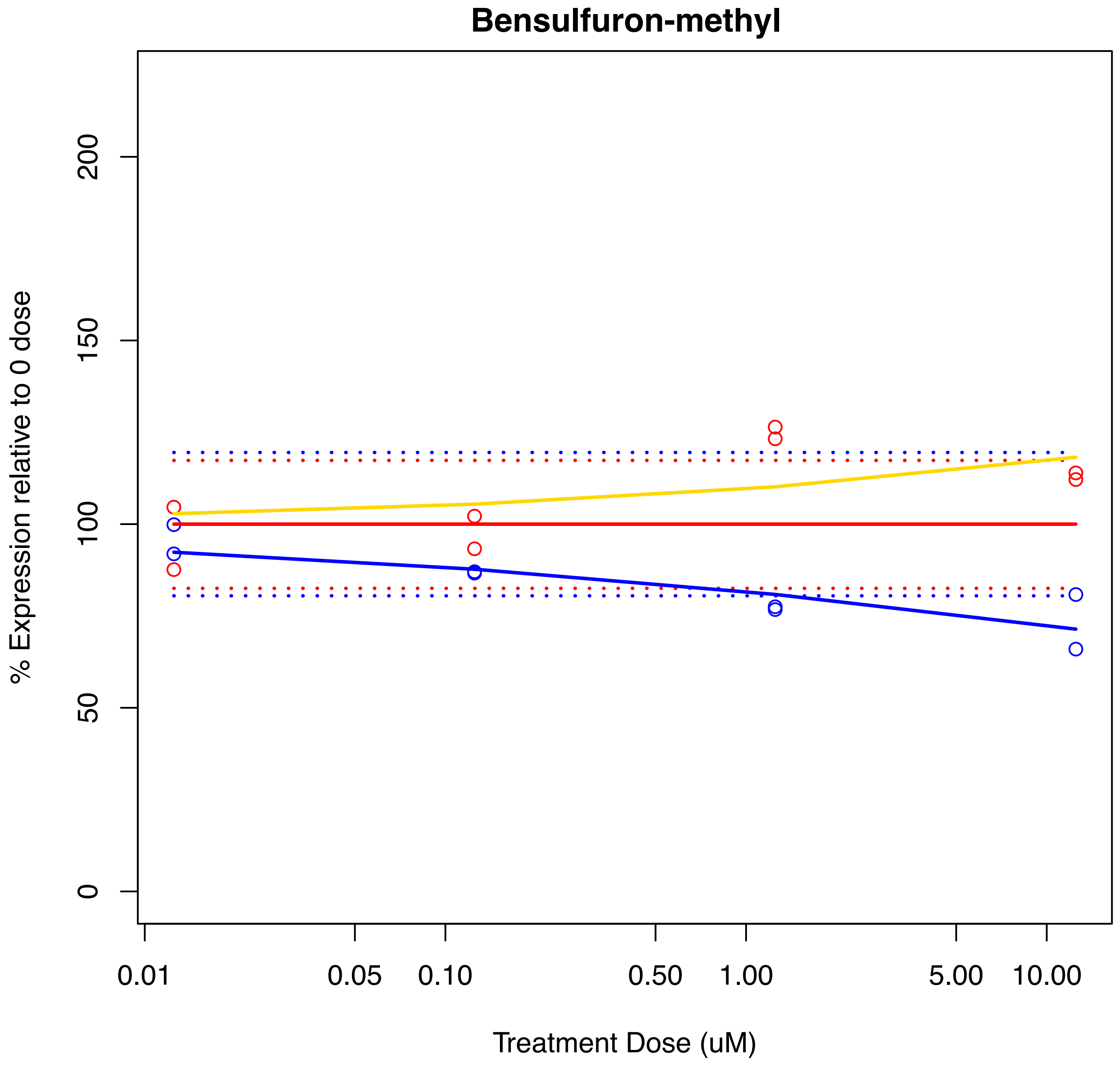
Bensulfuron-methyl
% Expression relative to 0 dose
0
50
100
150
200
0.01
0.05
0.10
0.50
1.00
5.00
10.00
Treatment Dose (uM)

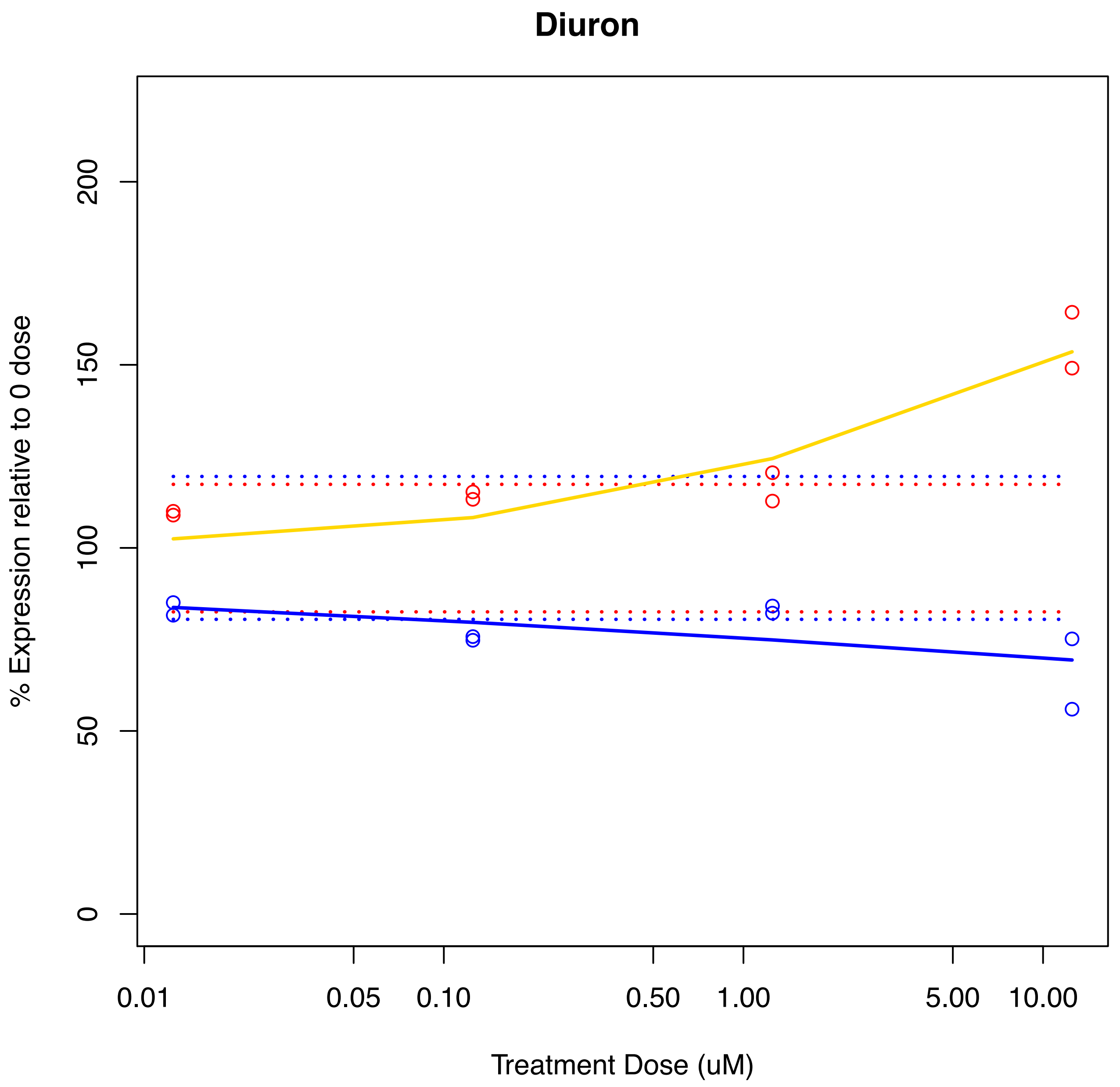

Diuron
% Expression relative to 0 dose
0
50
100
150
200
0.01
0.05
0.10
0.50
1.00
5.00
10.00
Treatment Dose (uM)

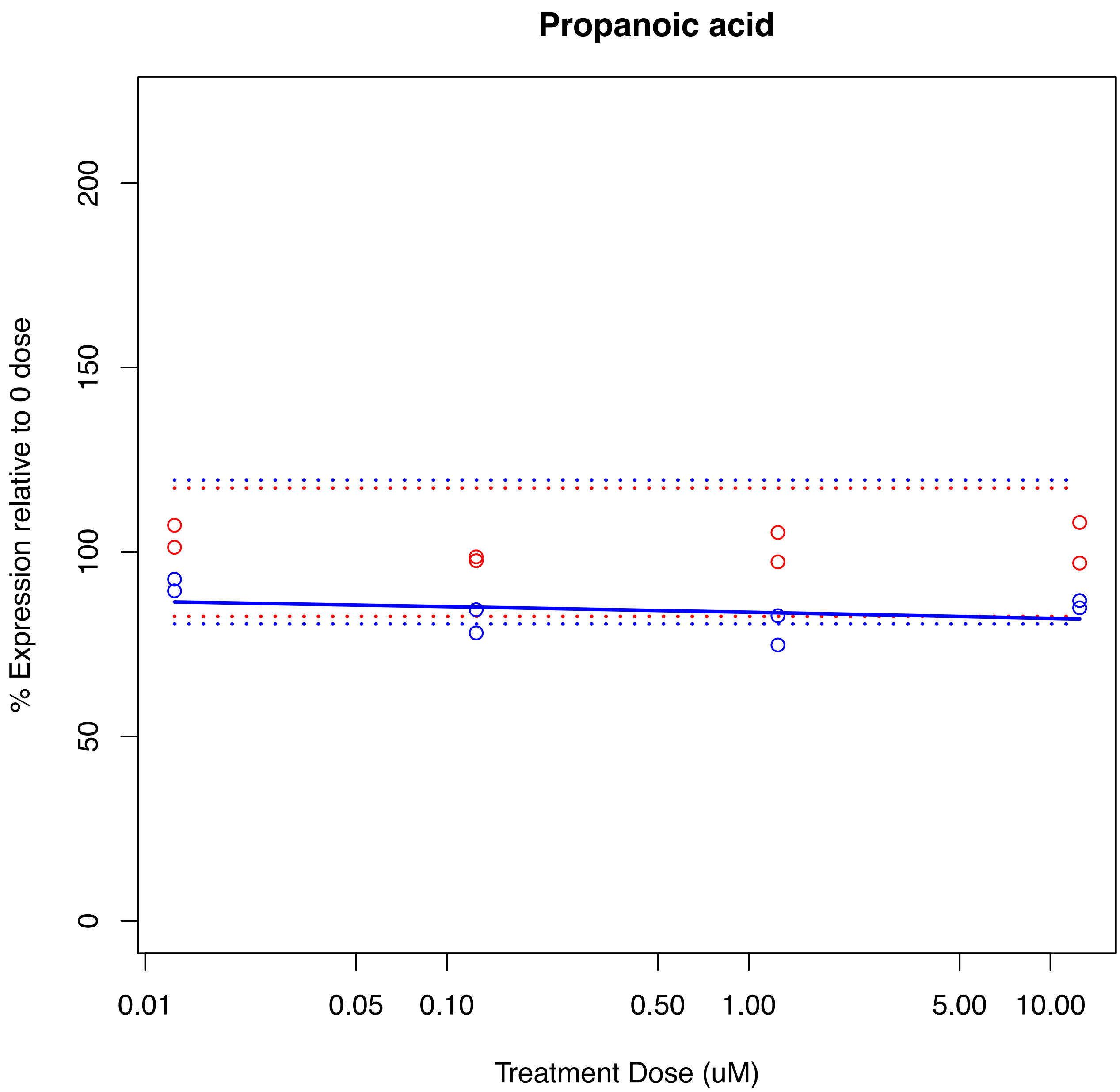

Propanoic acid
% Expression relative to 0 dose
0
50
100
150
200
0.01
0.05
0.10
0.50
1.00
5.00
10.00
Treatment Dose (uM)

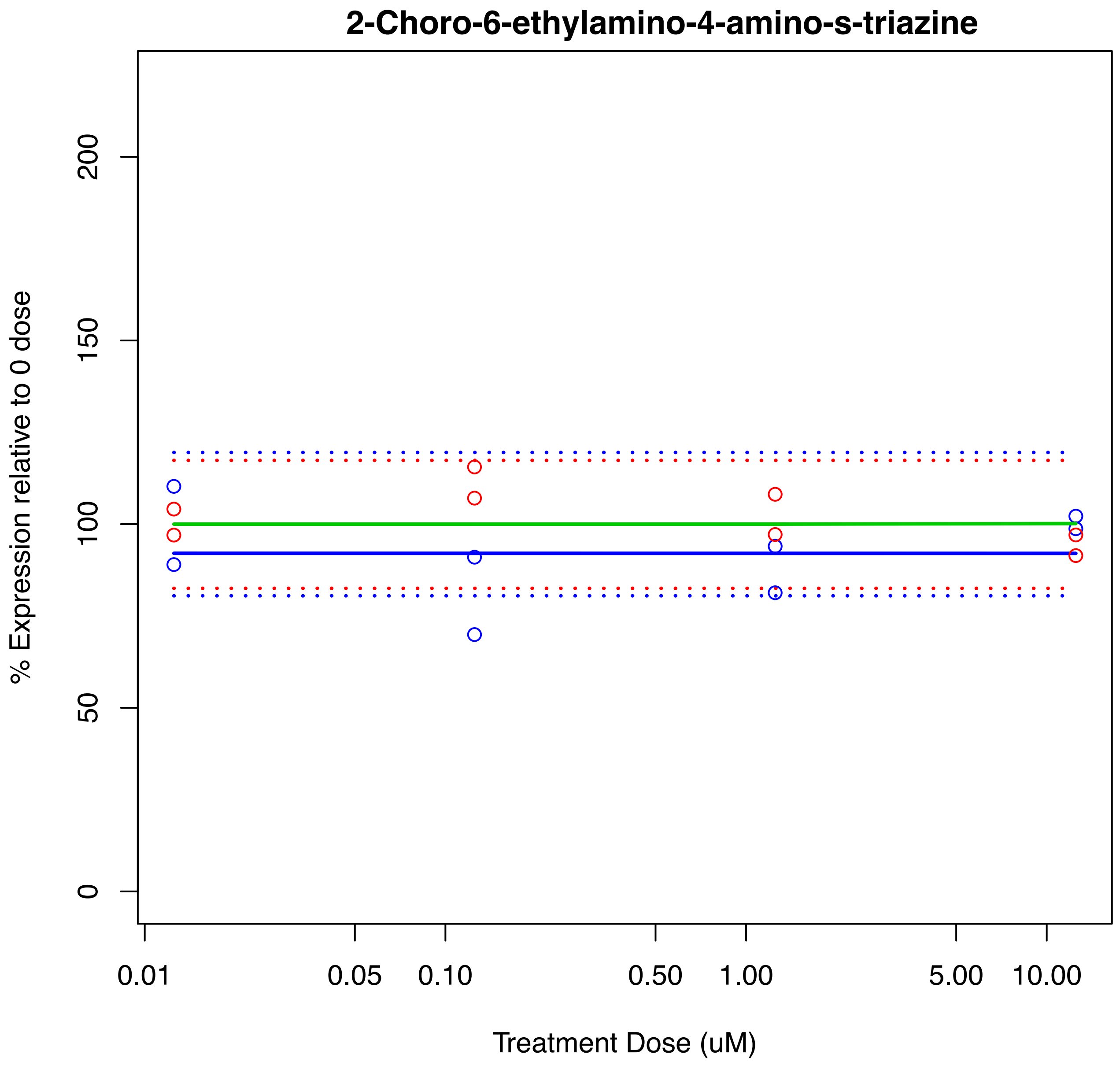
2-Choro-6-ethylamino-4-amino-s-triazine
% Expression relative to 0 dose
0
50
100
150
200
0.01
0.05
0.10
0.50
1.00
5.00
10.00
Treatment Dose (uM)

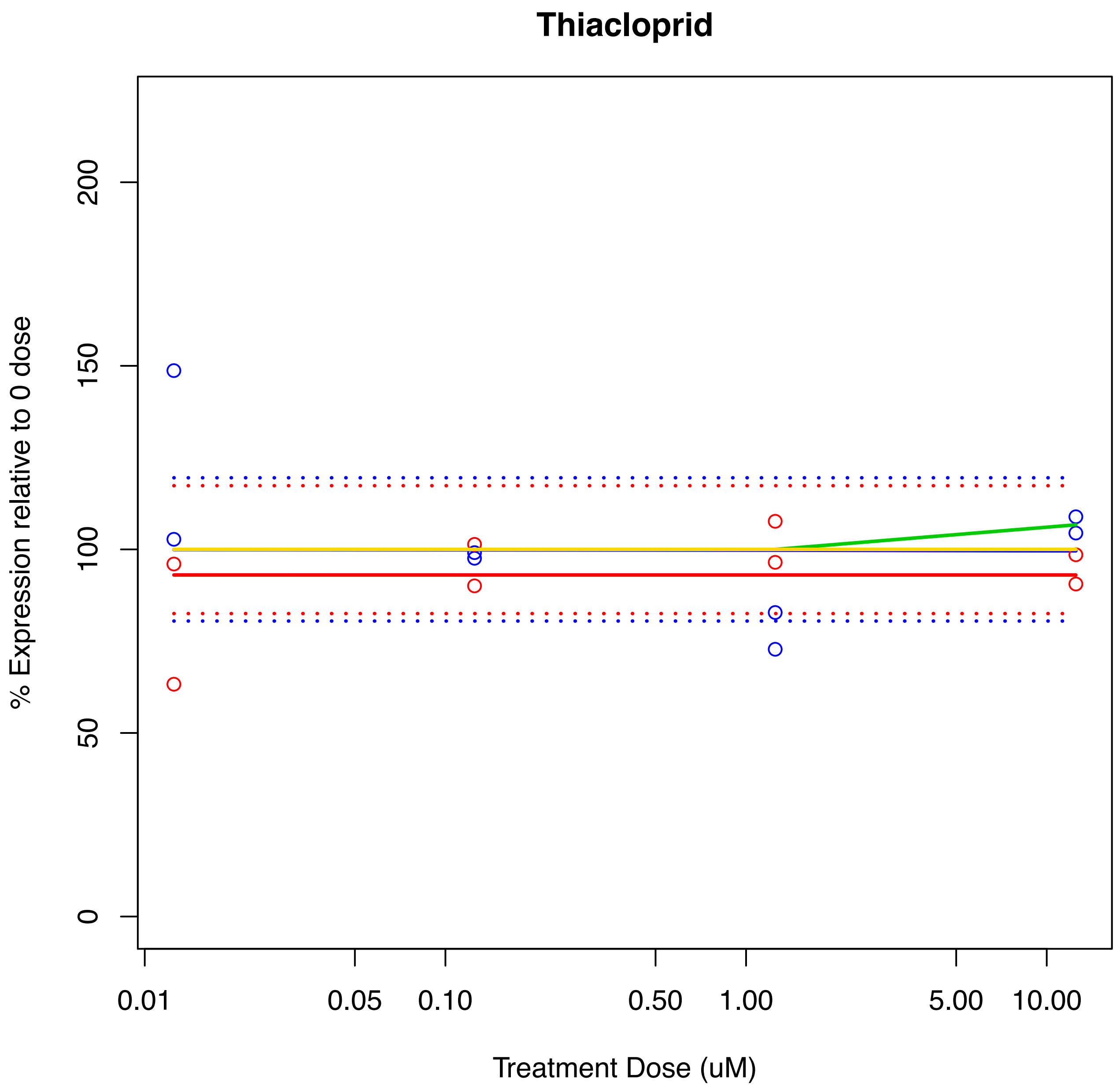
Thiacloprid
% Expression relative to 0 dose
0
50
100
150
200
0.01
0.05
0.10
0.50
1.00
5.00
10.00
Treatment Dose (uM)

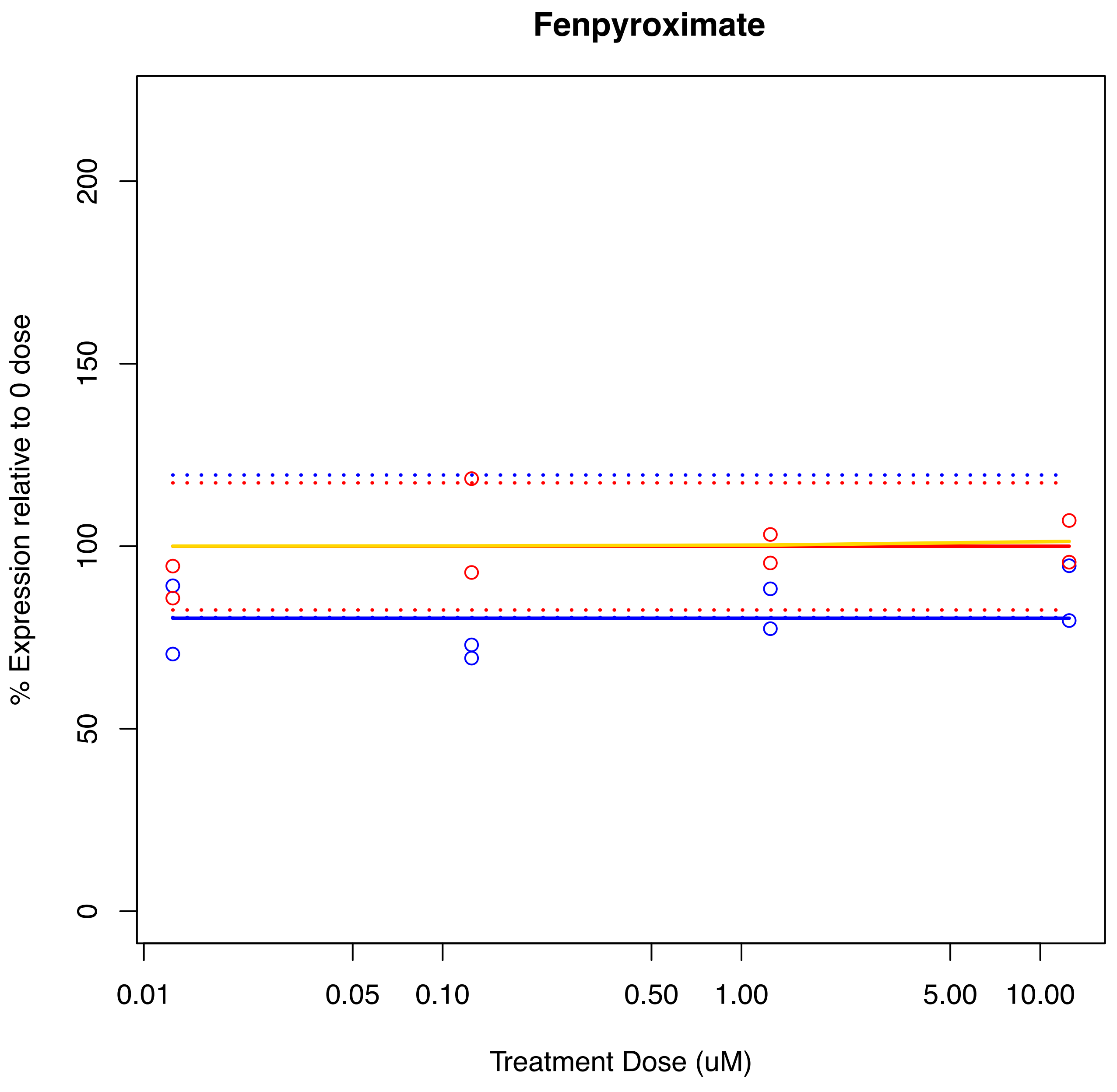

Fenpyroximate
% Expression relative to 0 dose
0
50
100
150
200
0.01
0.05
0.10
0.50
1.00
5.00
10.00
Treatment Dose (uM)

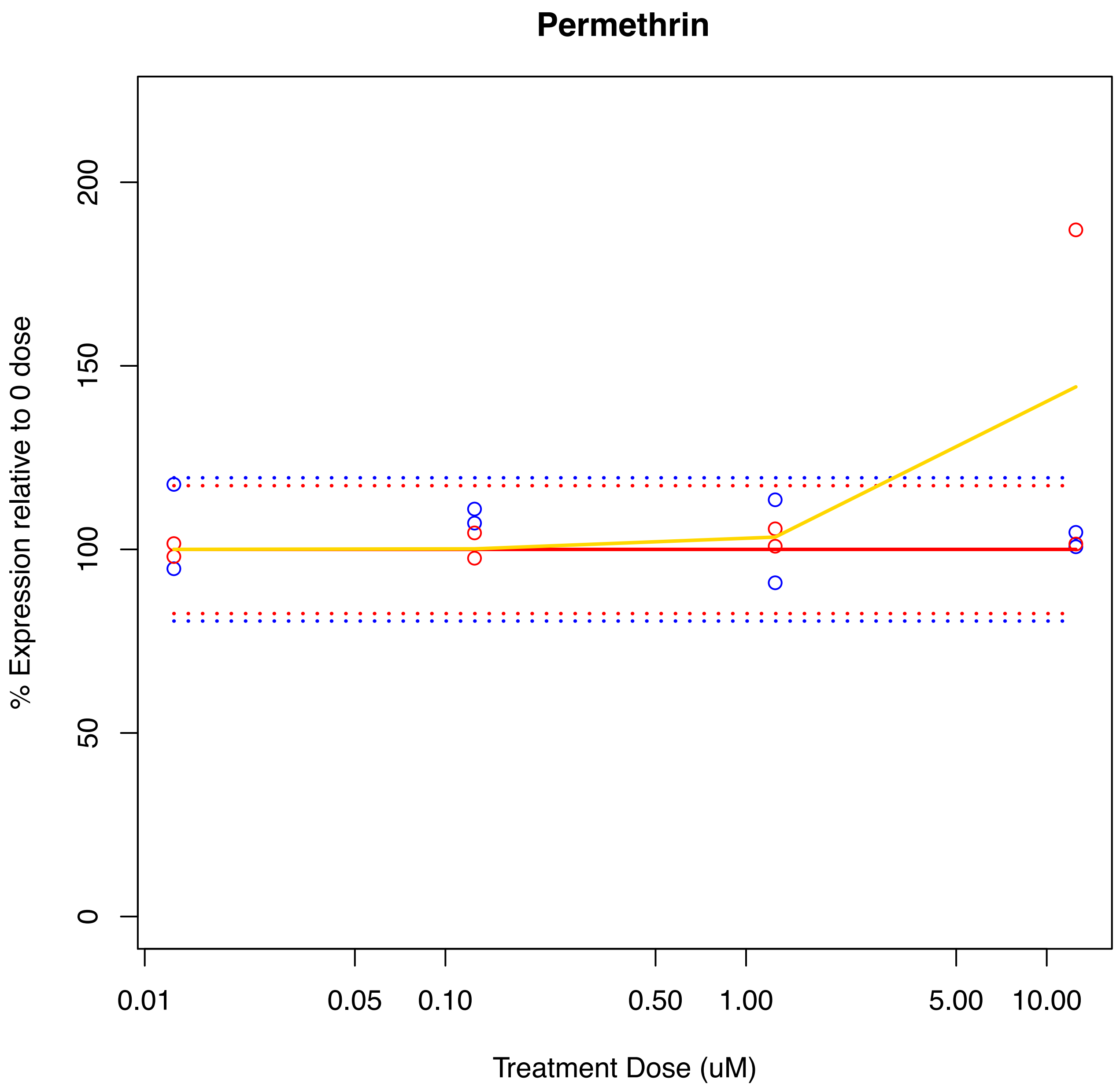
Permethrin
% Expression relative to 0 dose
0
50
100
150
200
0.01
0.05
0.10
0.50
1.00
5.00
10.00
Treatment Dose (uM)

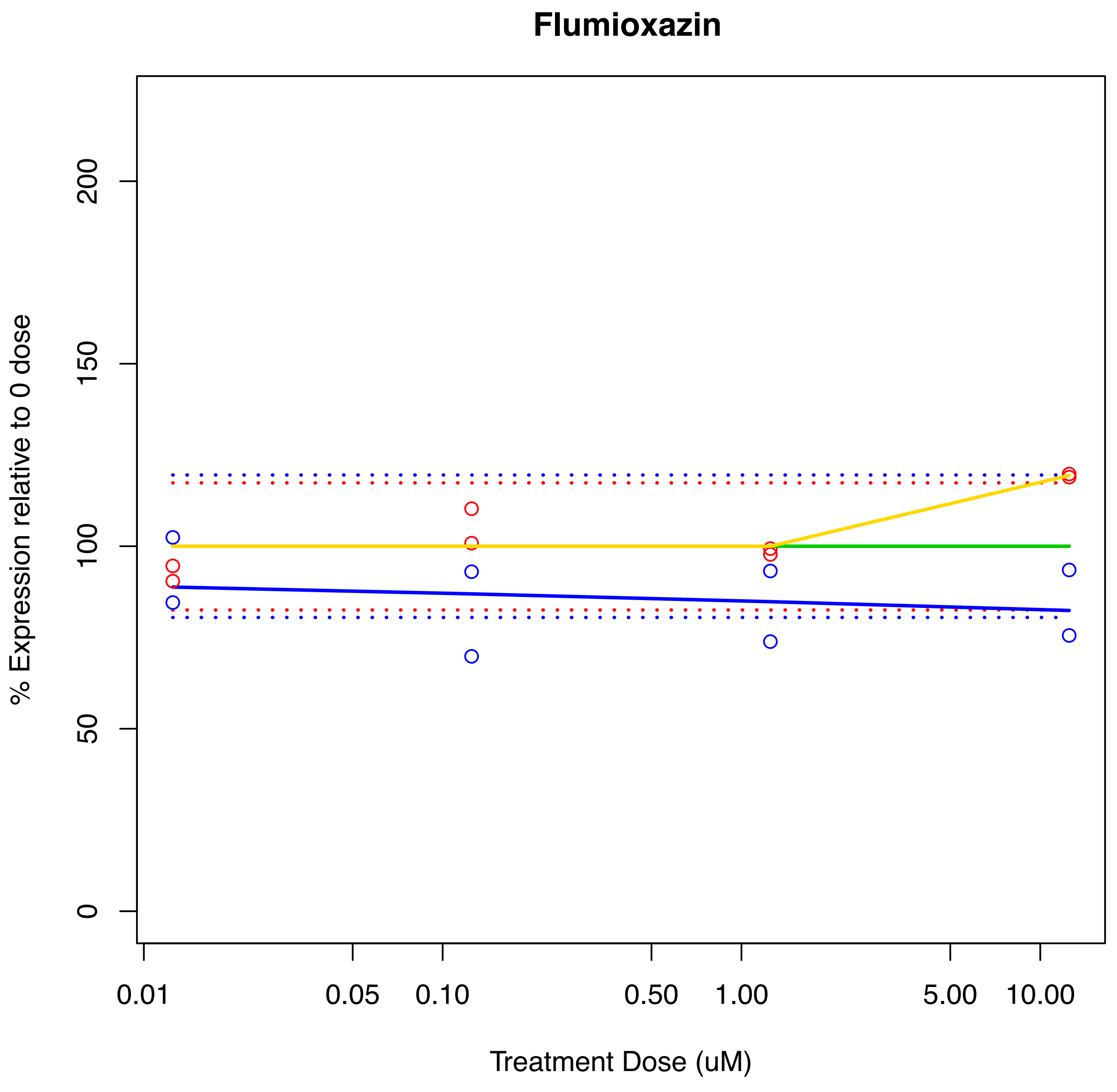
Flumioxazin
% Expression relative to 0 dose
0
50
100
150
200
0.01
0.05
0.10
0.50
1.00
5.00
10.00
Treatment Dose (uM)

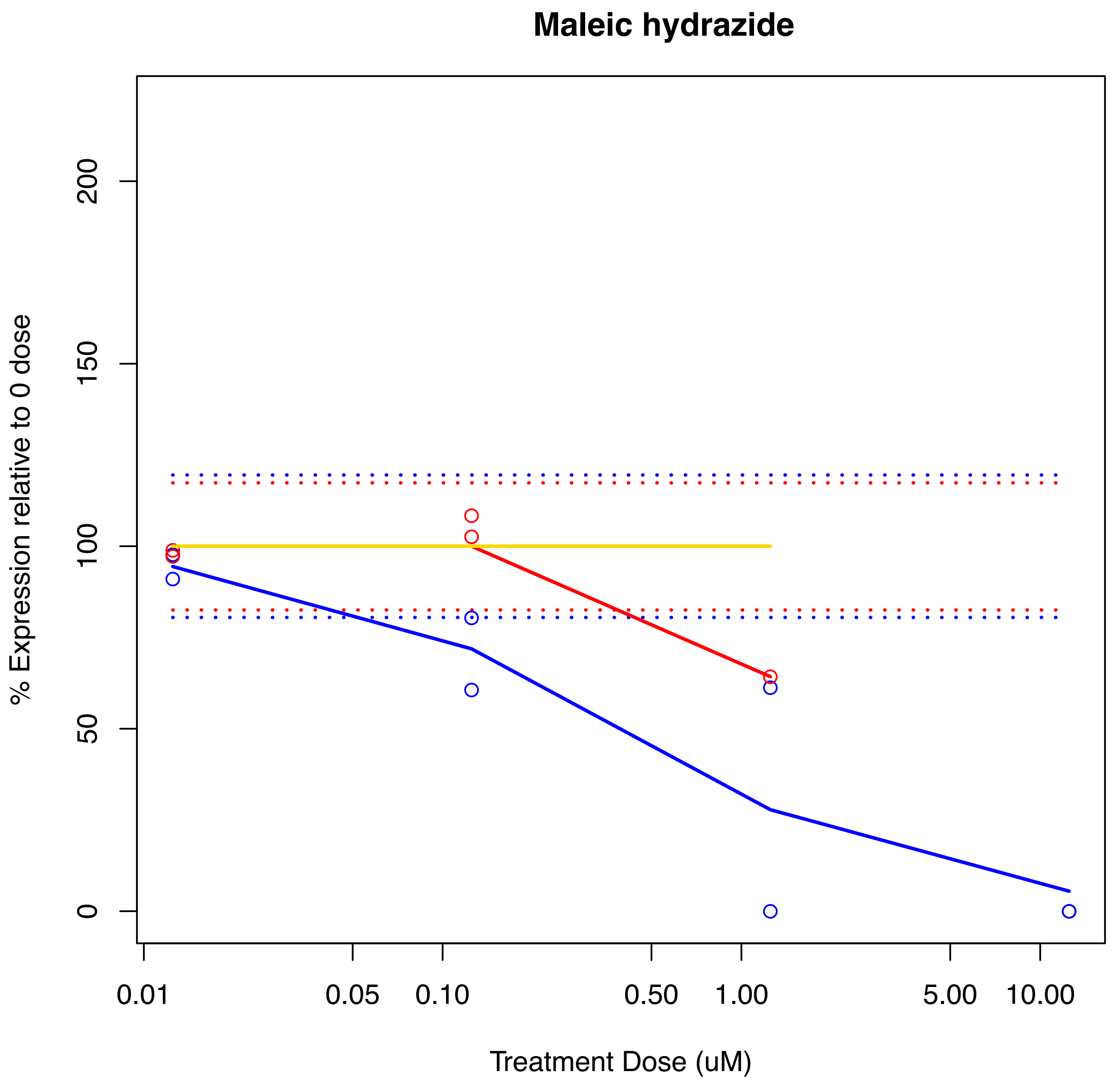

Maleic hydrazide
% Expression relative to 0 dose
0
50
100
150
200
0.01
0.05
0.10
0.50
1.00
5.00
10.00
Treatment Dose (uM)

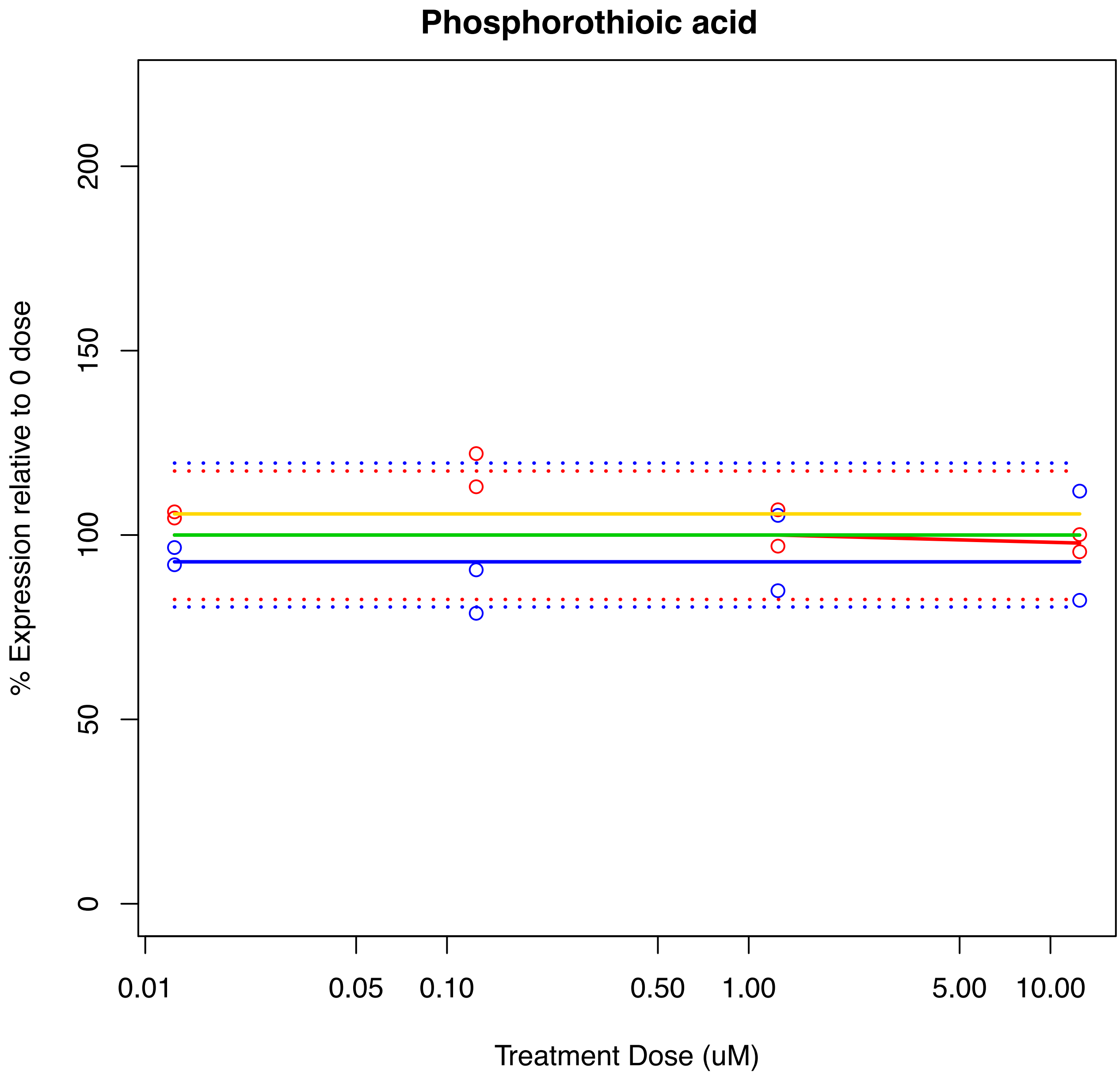

Phosphorothioic acid
% Expression relative to 0 dose
0
50
100
150
200
0.01
0.05
0.10
0.50
1.00
5.00
10.00
Treatment Dose (uM)

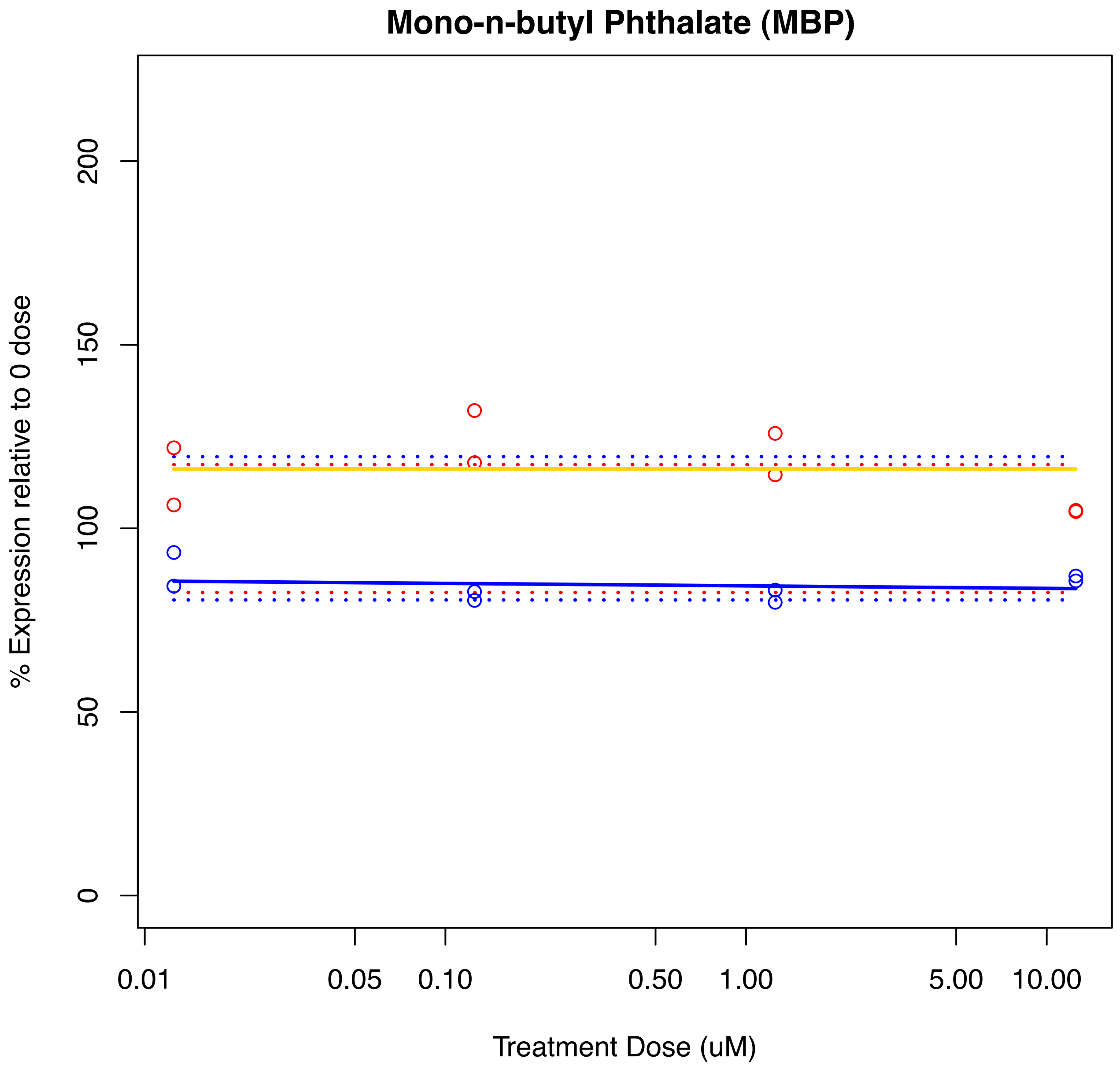

Mono-n-butyl Phthalate (MBP)
% Expression relative to 0 dose
0
50
100
150
200
0.01
0.05
0.10
0.50
1.00
5.00
10.00
Treatment Dose (uM)

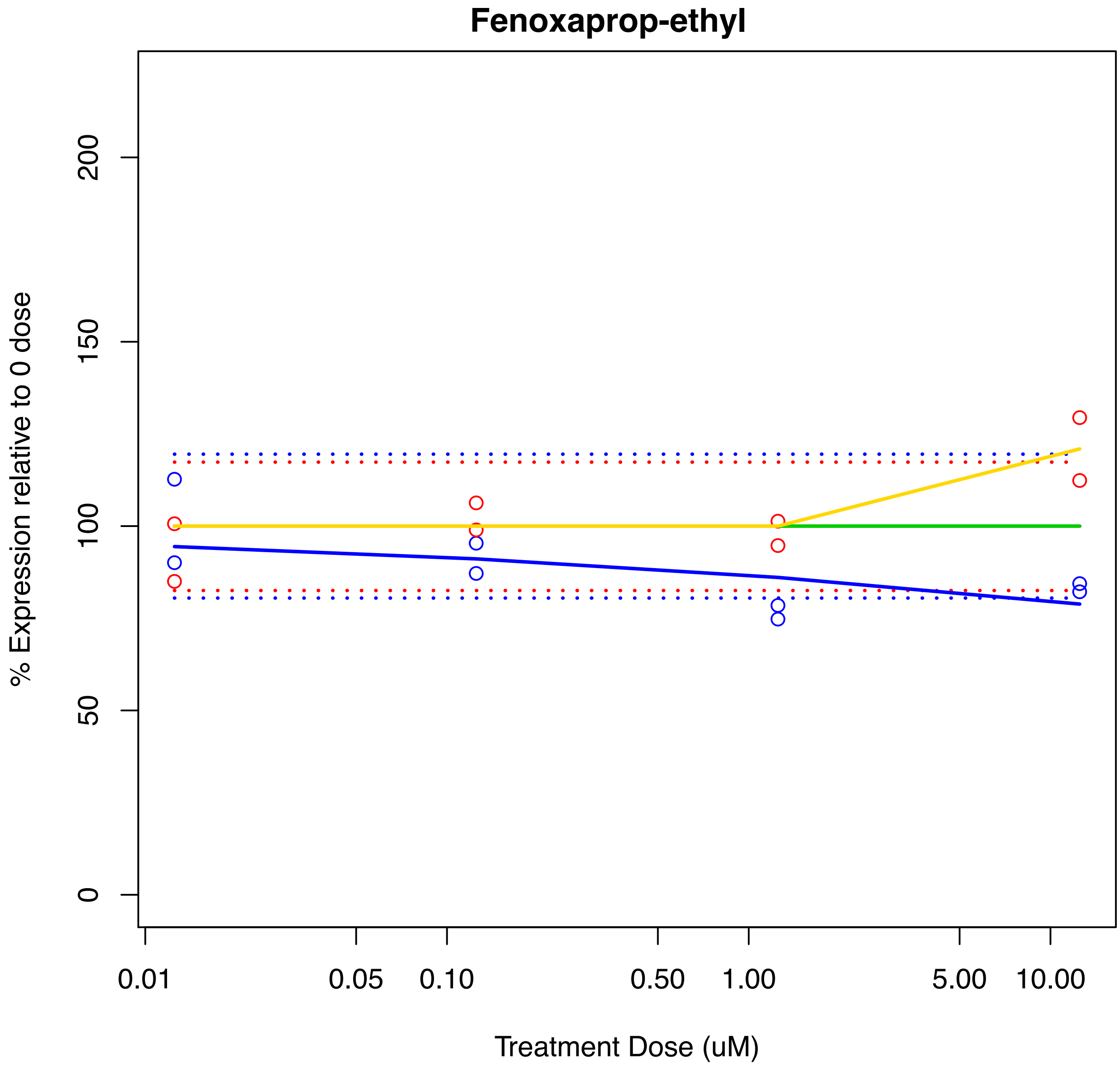

Fenoxaprop-ethyl
% Expression relative to 0 dose
0
50
100
150
200
0.01
0.05
0.10
0.50
1.00
5.00
10.00
Treatment Dose (uM)

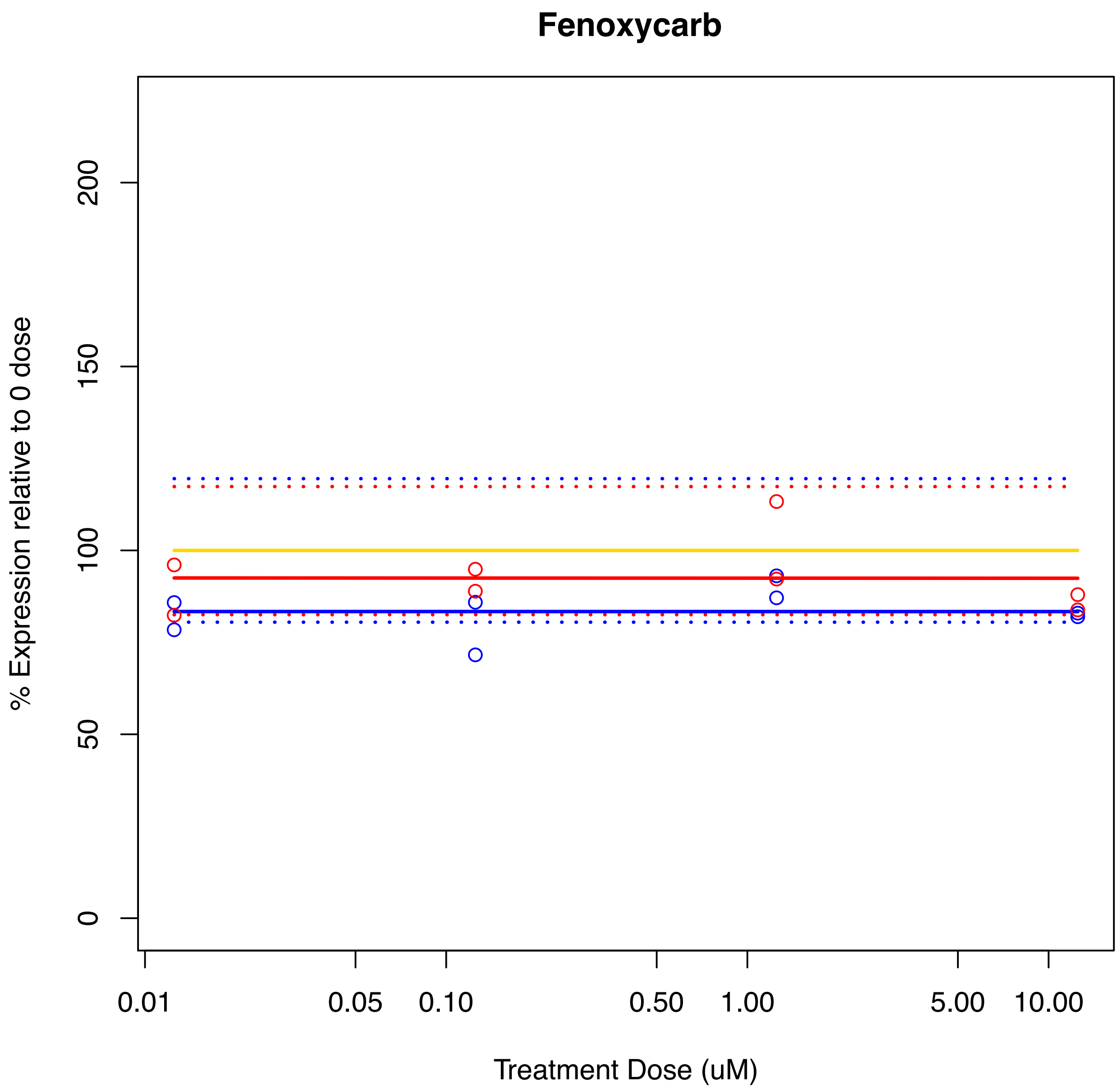
Fenoxycarb
% Expression relative to 0 dose
0
50
100
150
200
0.01
0.05
0.10
0.50
1.00
5.00
10.00
Treatment Dose (uM)

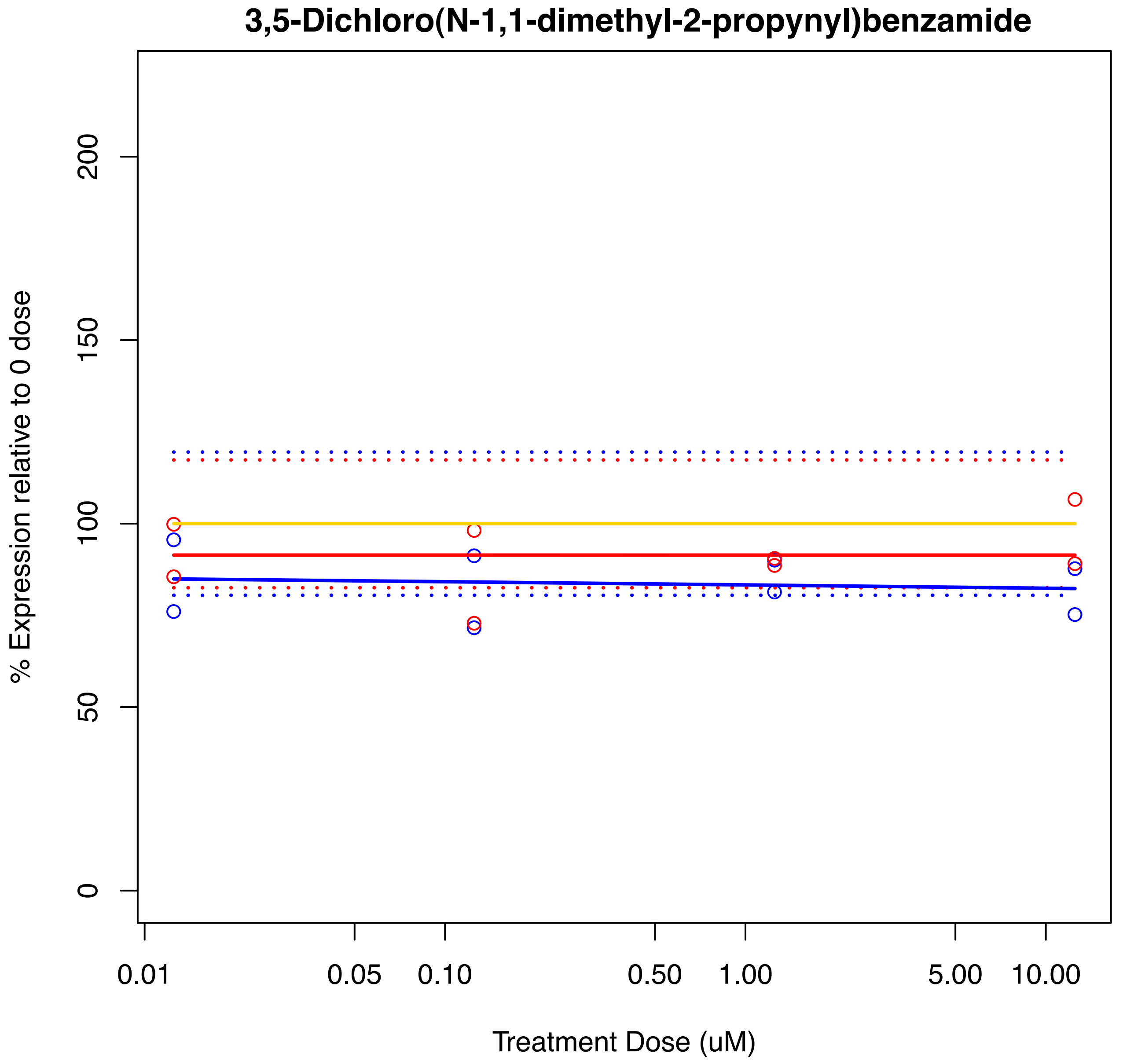

3,5-Dichloro(N-1,1-dimethyl-2-propynyl)benzamide
% Expression relative to 0 dose
0
50
100
150
200
0.01
0.05
0.10
0.50
1.00
5.00
10.00
Treatment Dose (uM)

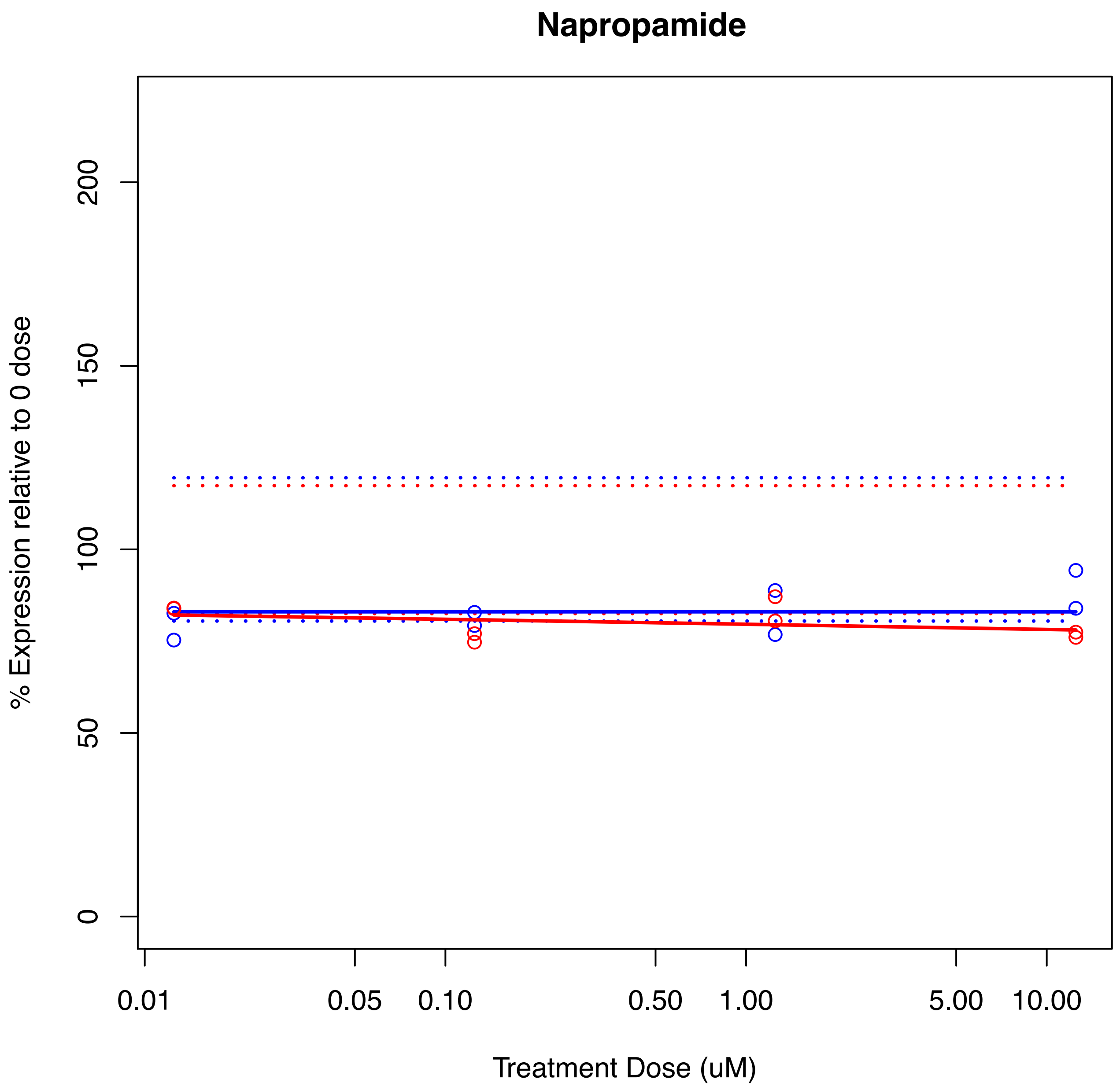
Napropamide
% Expression relative to 0 dose
Treatment Dose (uM)
0
50
100
150
200
0.01
0.05
0.10
0.50
1.00
5.00
10.00

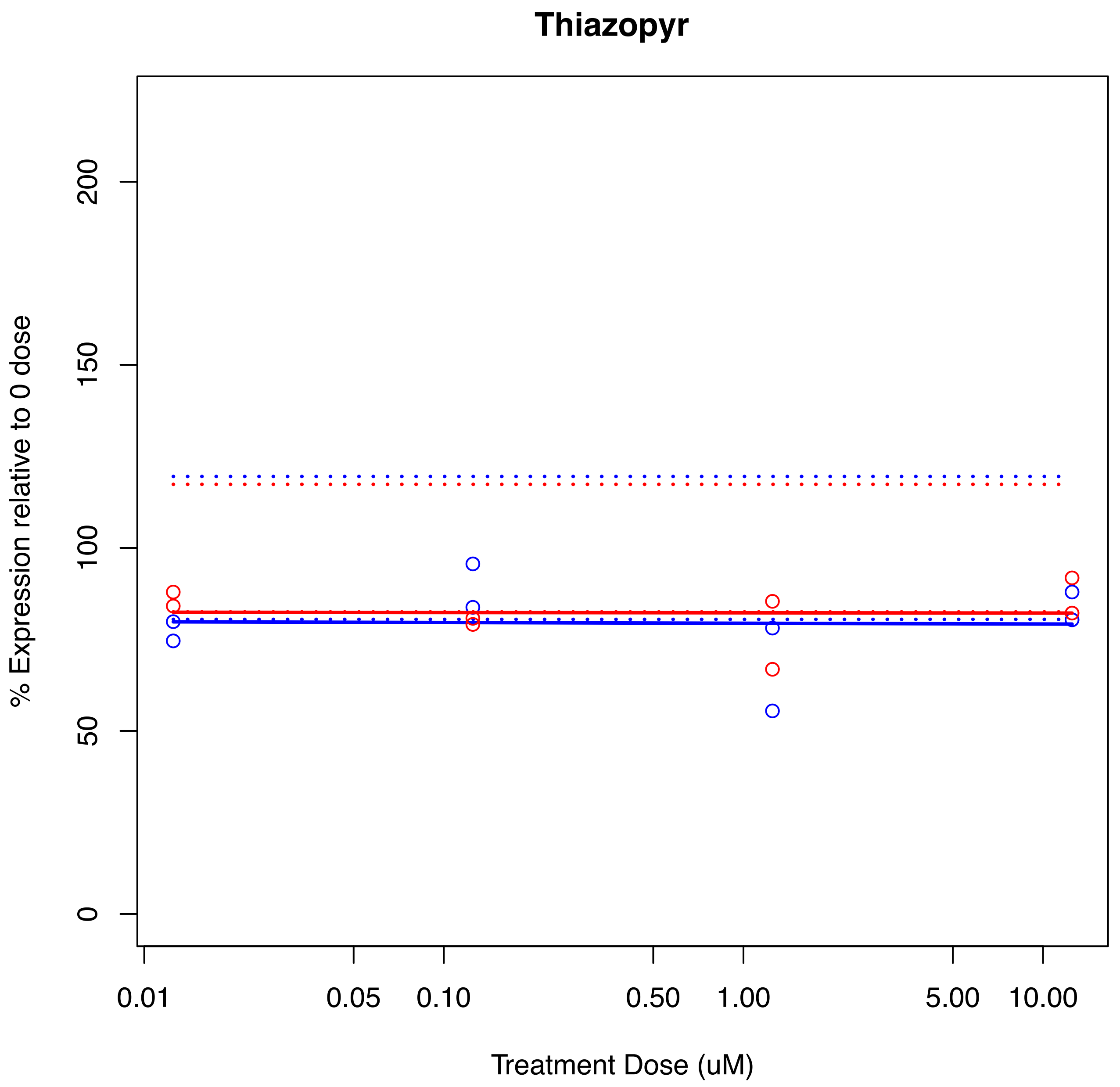
Thiazopyr
% Expression relative to 0 dose
0
50
100
150
200
0.01
0.05
0.10
0.50
1.00
5.00
10.00
Treatment Dose (uM)

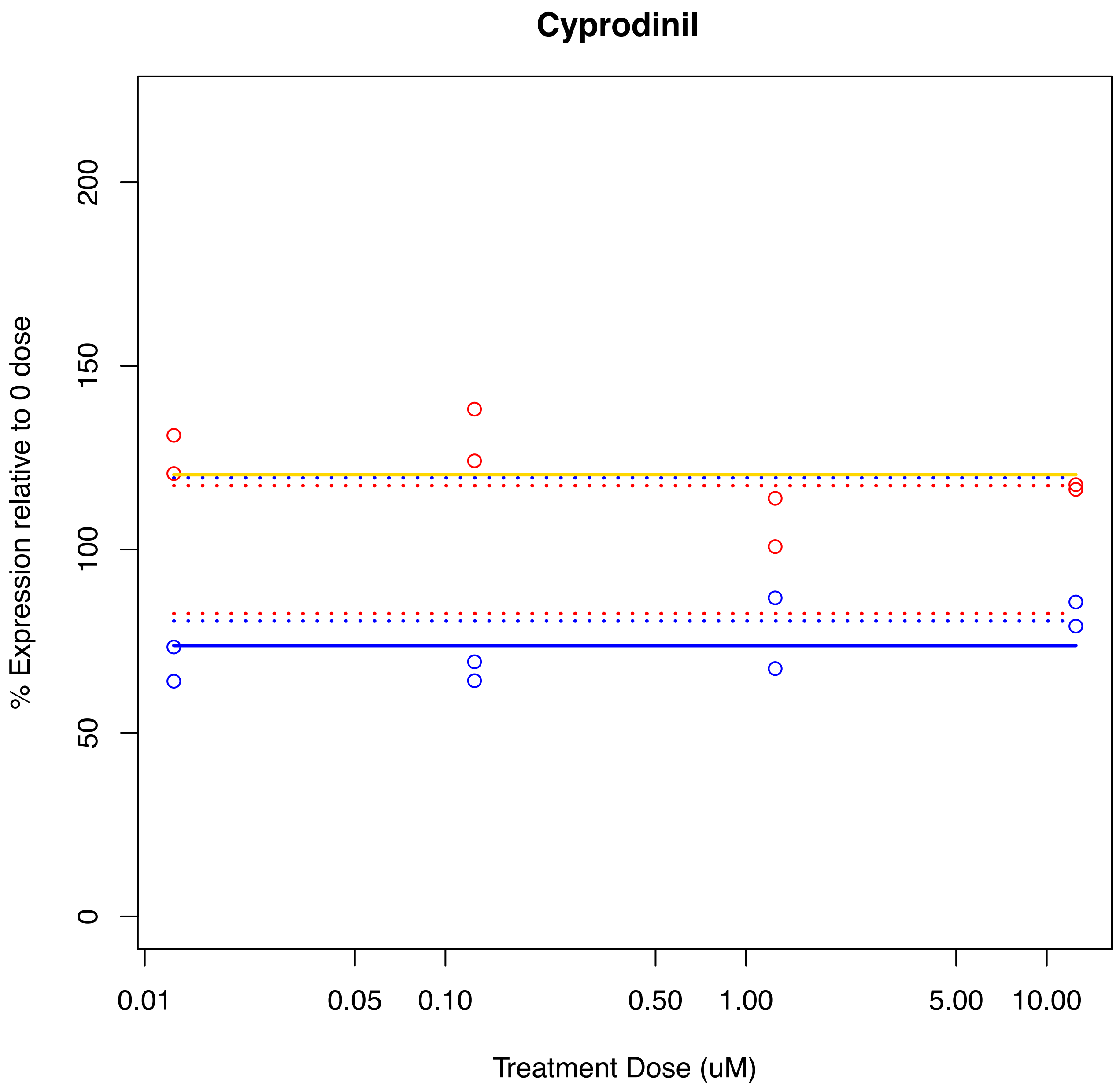
Cyprodinil
% Expression relative to 0 dose
0
50
100
150
200
0.01
0.05
0.10
0.50
1.00
5.00
10.00
Treatment Dose (uM)

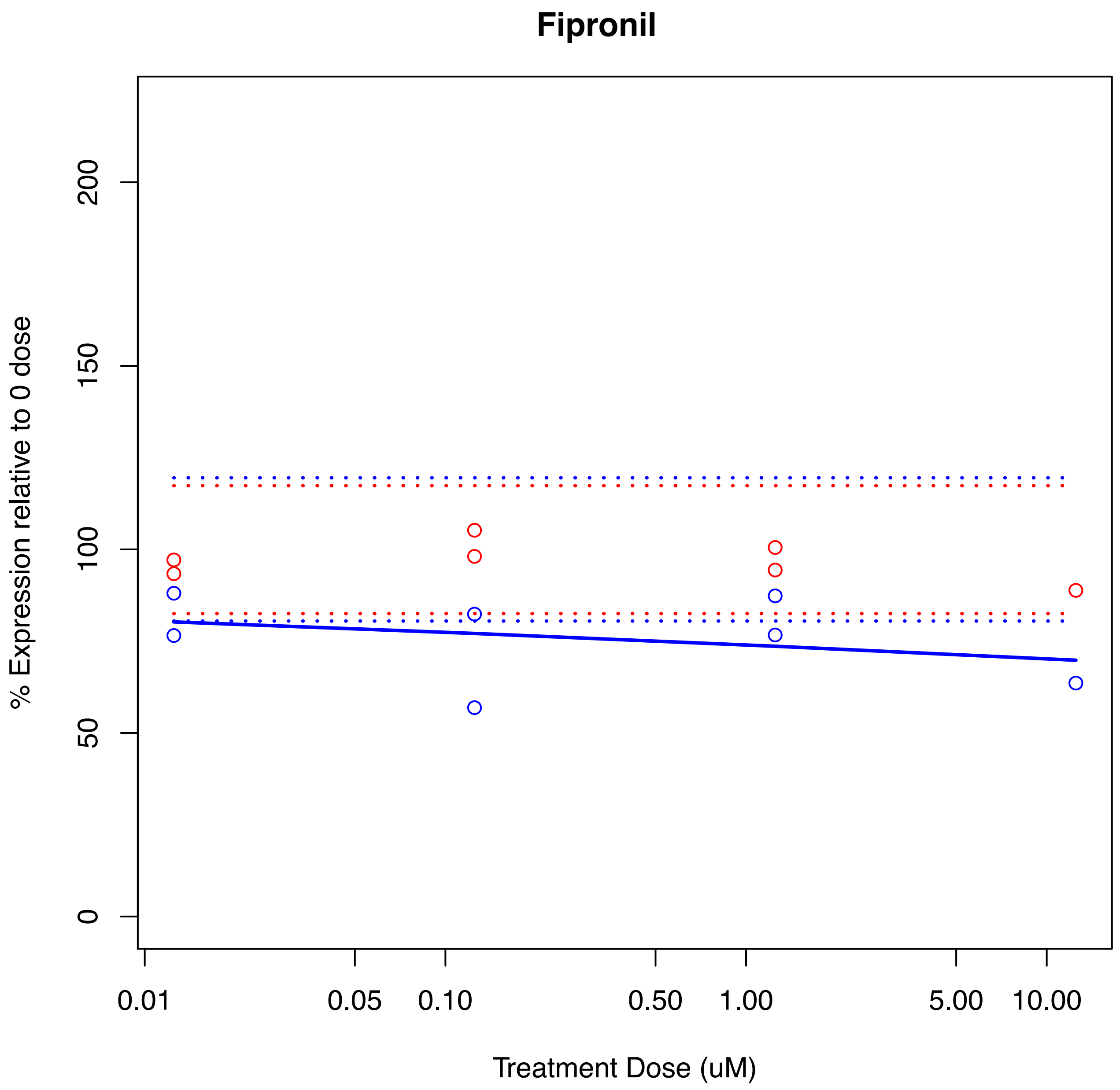
Fipronil
% Expression relative to 0 dose
0
50
100
150
200
0.01
0.05
0.10
0.50
1.00
5.00
10.00
Treatment Dose (uM)

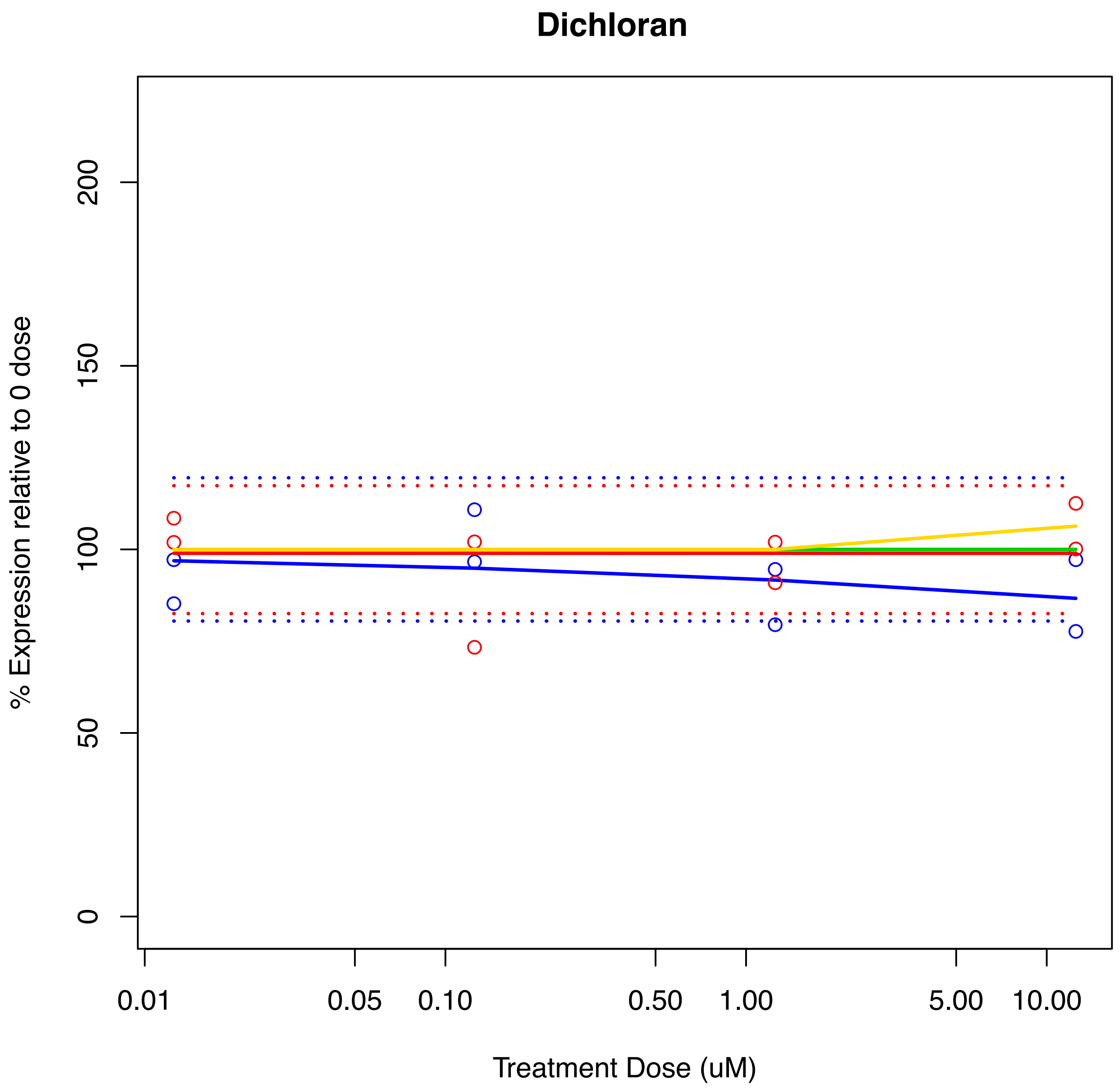

Dichloran
% Expression relative to 0 dose
0
50
100
150
200
0.01
0.05
0.10
0.50
1.00
5.00
10.00
Treatment Dose (uM)

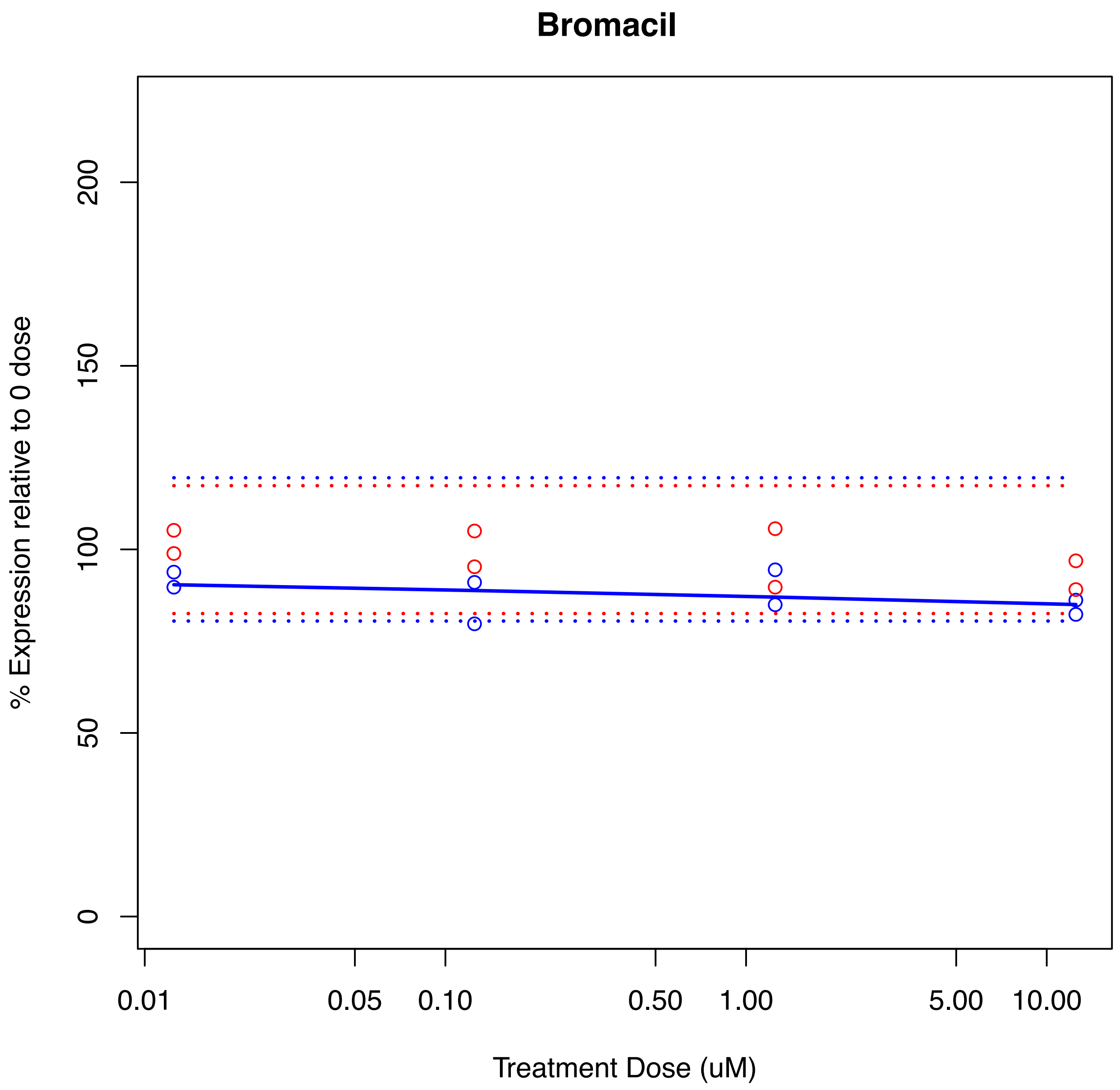

Bromacil
% Expression relative to 0 dose
0
50
100
150
200
0.01
0.05
0.10
0.50
1.00
5.00
10.00
Treatment Dose (uM)

Methanone

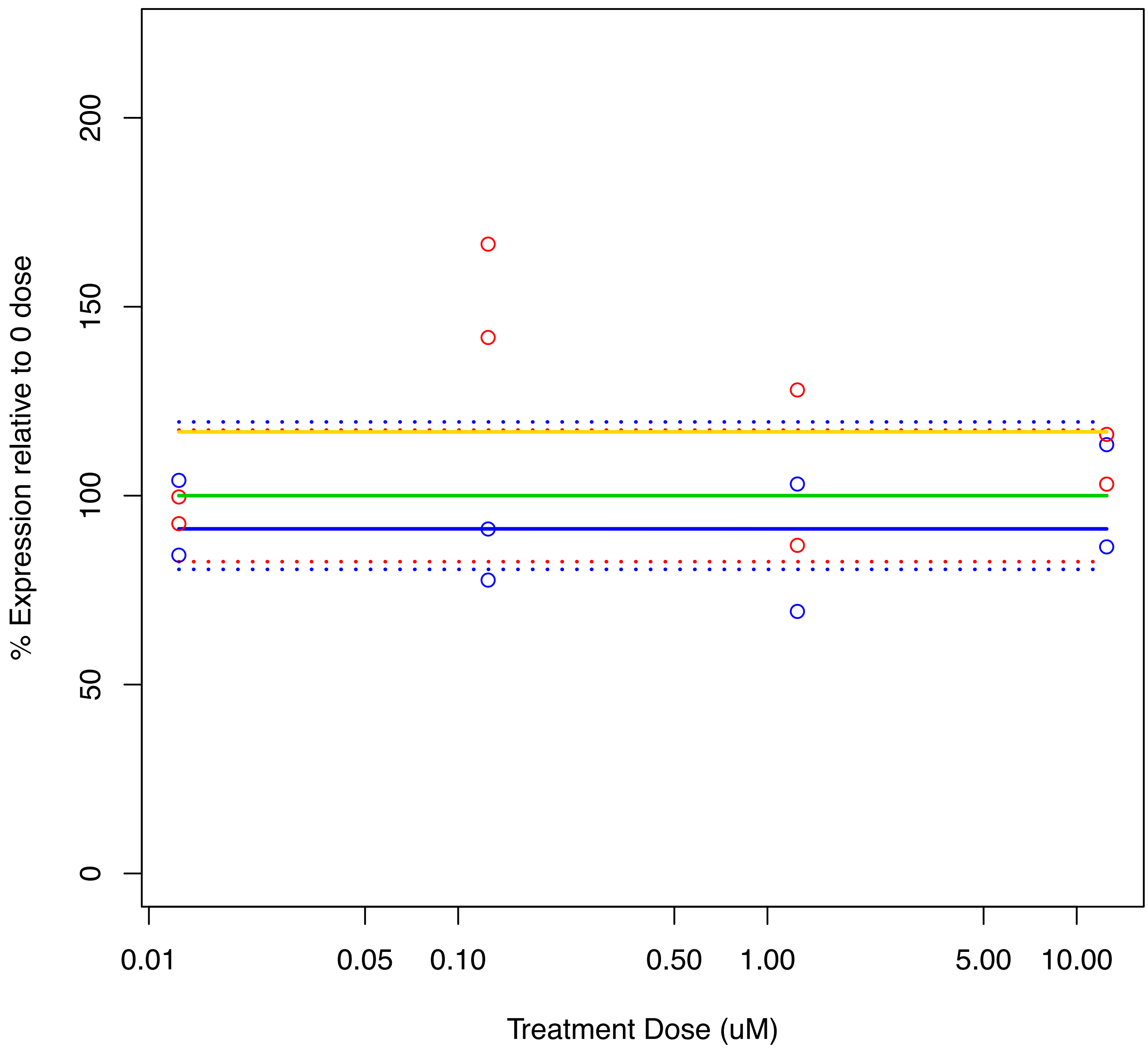

% Expression relative to 0 dose
0
50
100
150
200
0.01
0.05
0.10
0.50
1.00
5.00
10.00
Treatment Dose (uM)

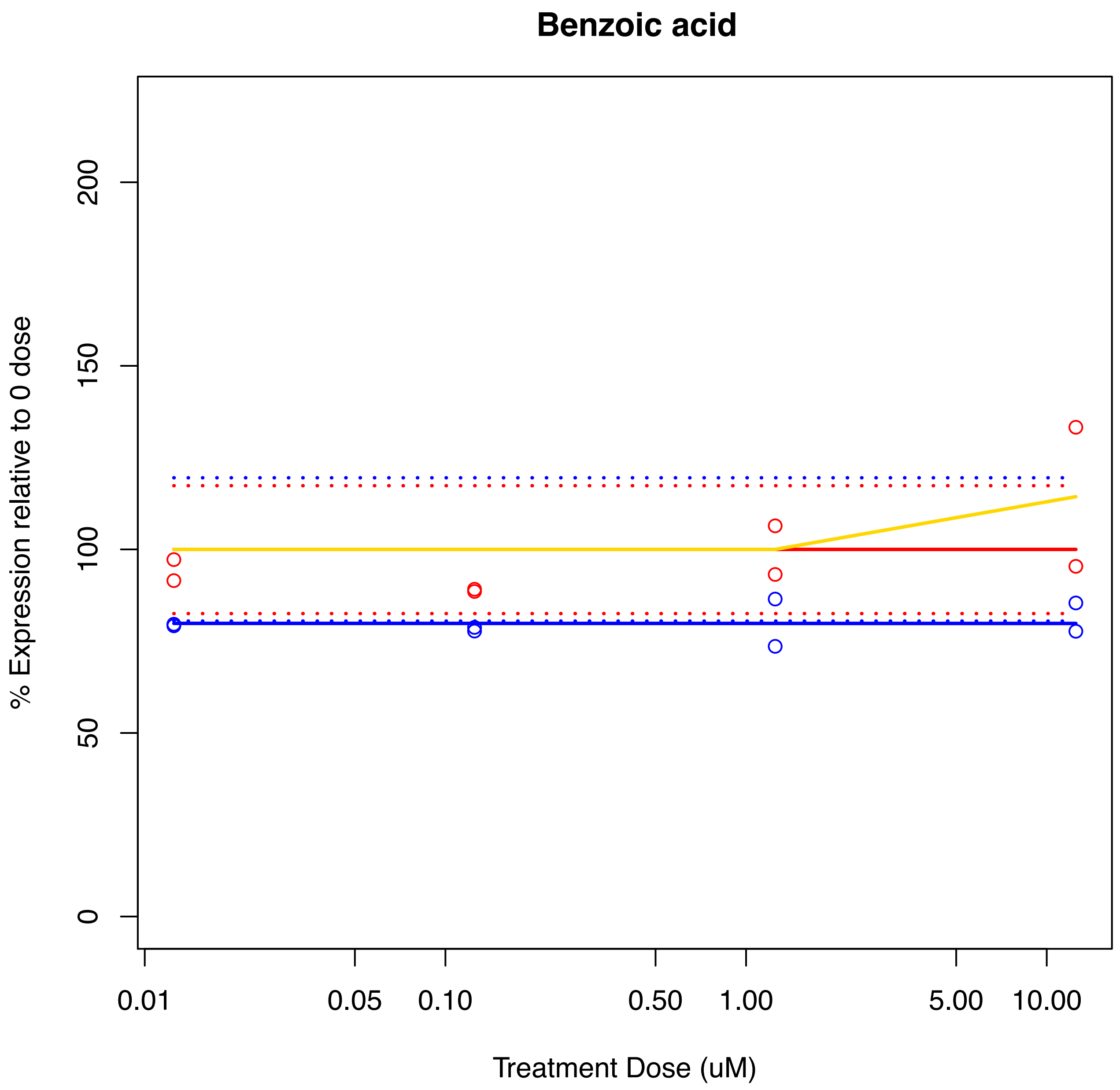
Benzoic acid
% Expression relative to 0 dose
0
50
100
150
200
0.01
0.05
0.10
0.50
1.00
5.00
10.00
Treatment Dose (uM)

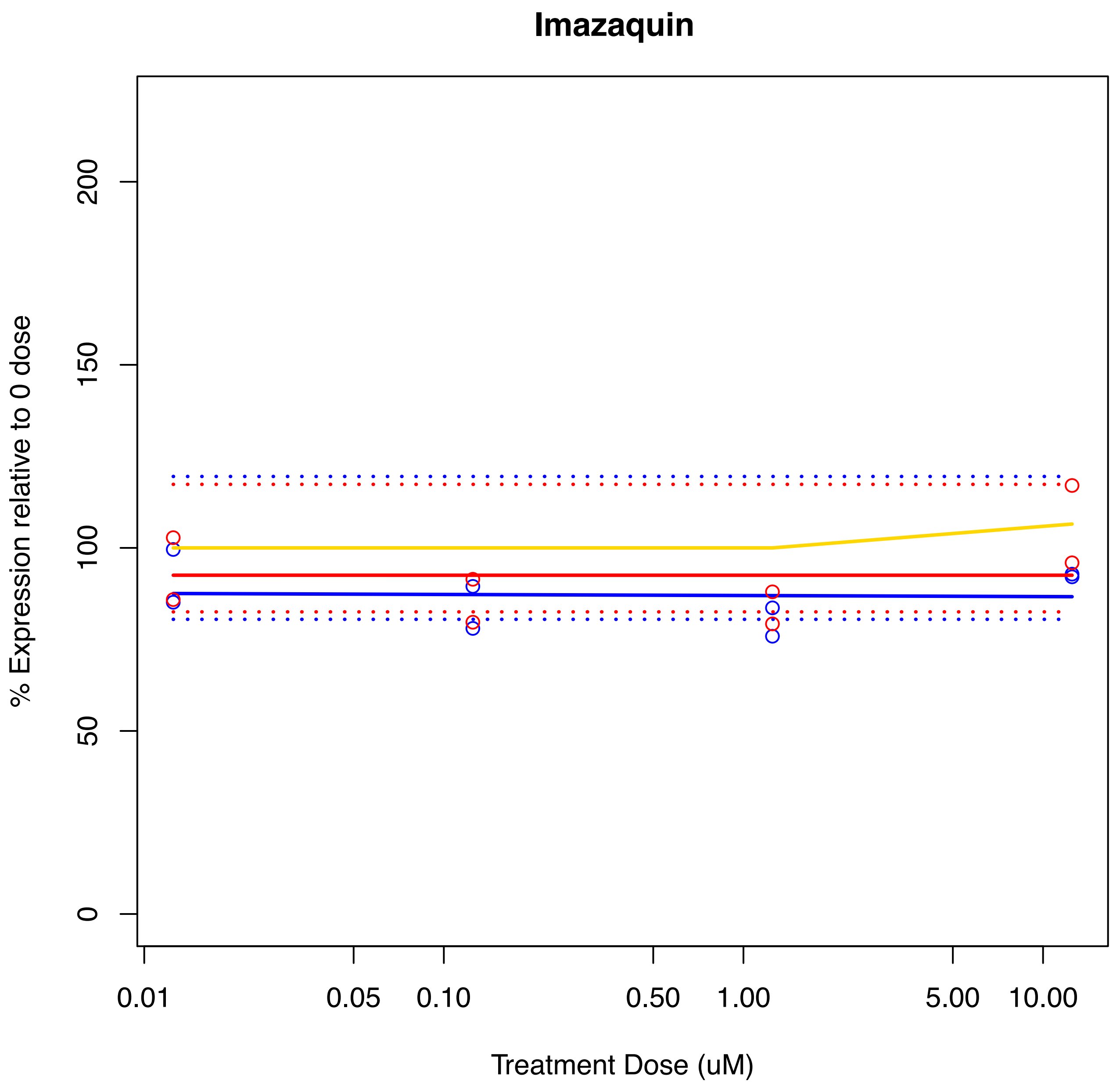

Imazaquin
% Expression relative to 0 dose
0
50
100
150
200
0.01
0.05
0.10
0.50
1.00
5.00
10.00
Treatment Dose (uM)

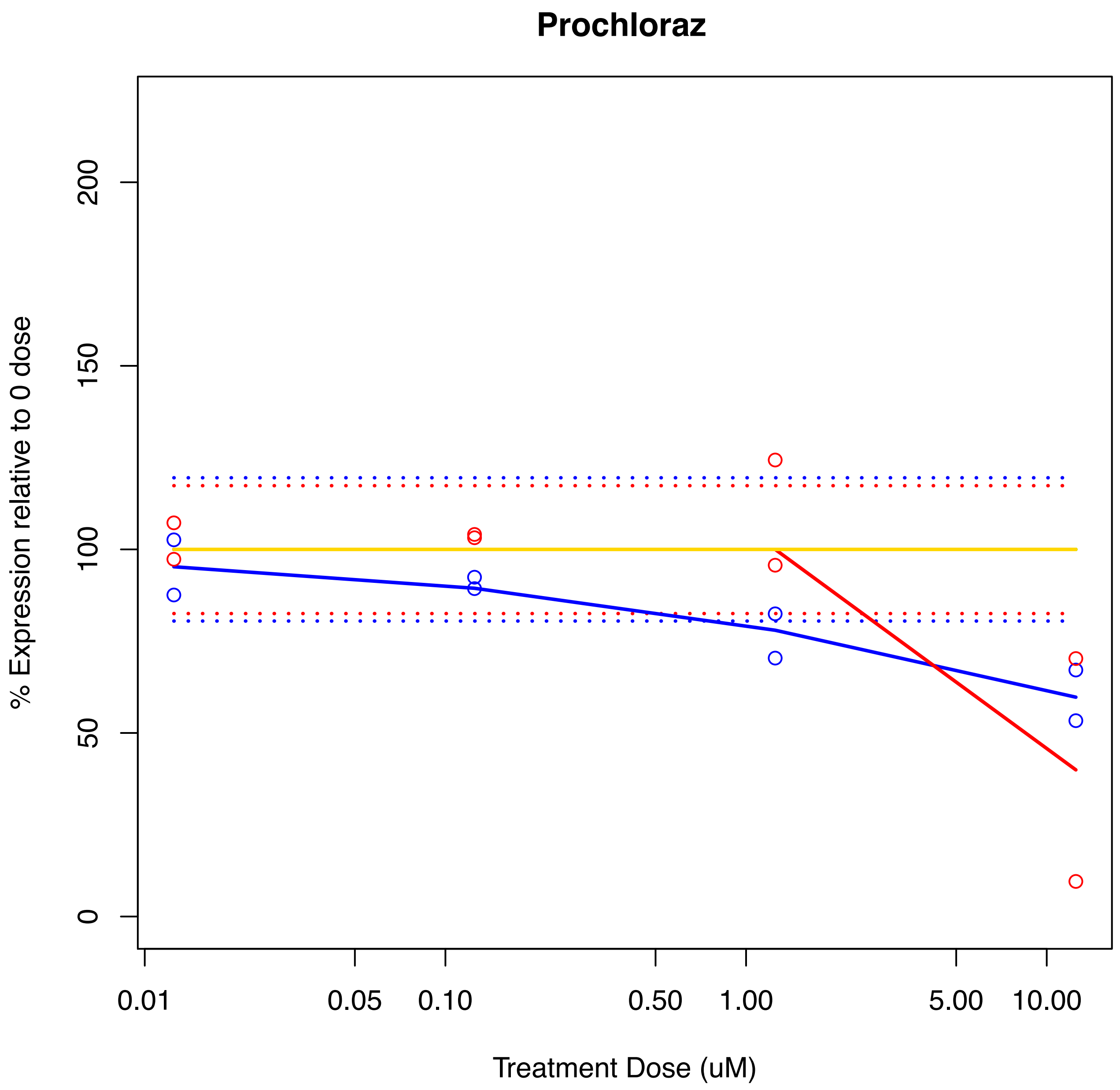
Prochloraz
% Expression relative to 0 dose
0
50
100
150
200
0.01
0.05
0.10
0.50
1.00
5.00
10.00
Treatment Dose (uM)

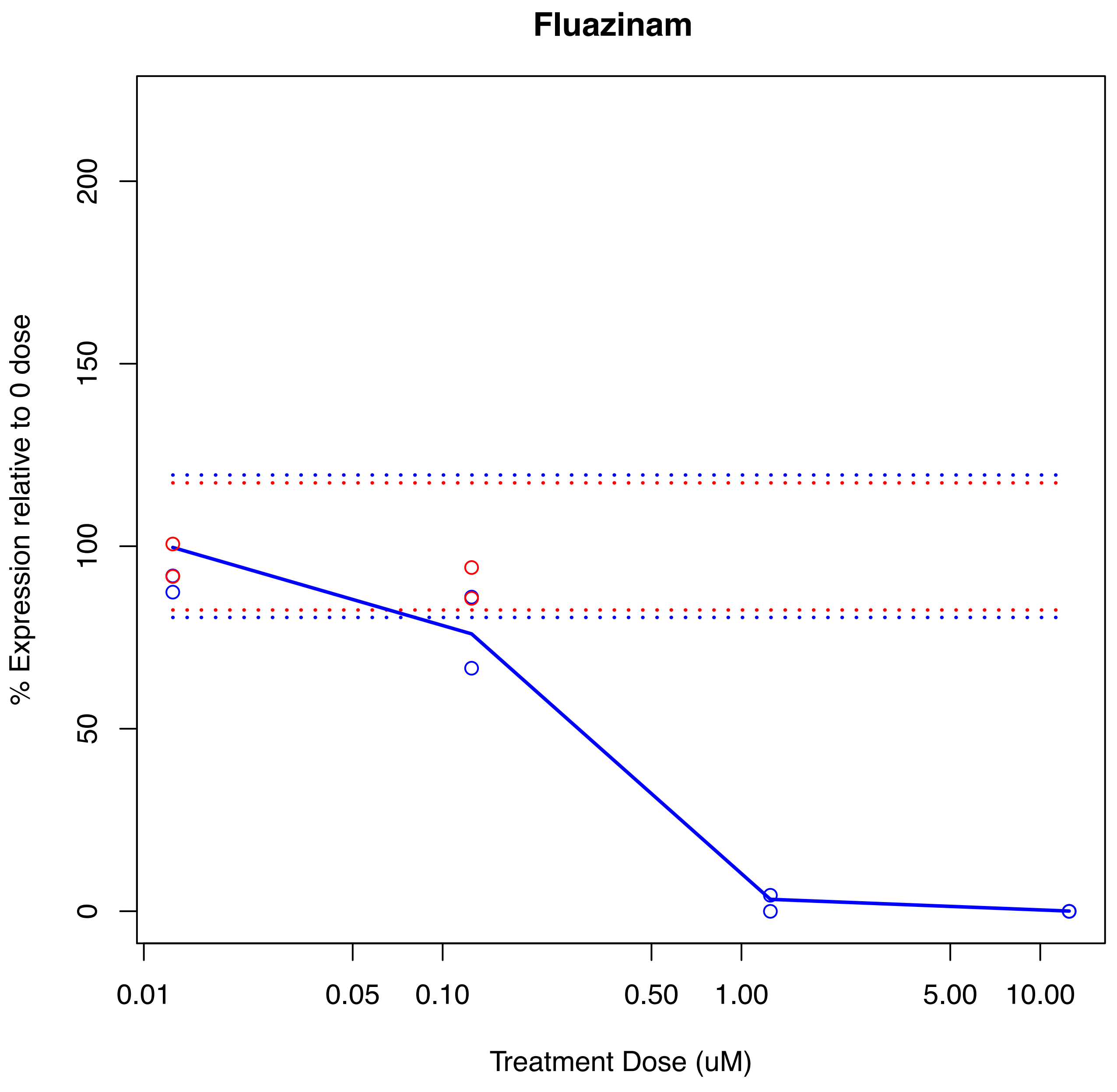

Fluazinam
% Expression relative to 0 dose
0
50
100
150
200
0.01
0.05
0.10
0.50
1.00
5.00
10.00
Treatment Dose (uM)

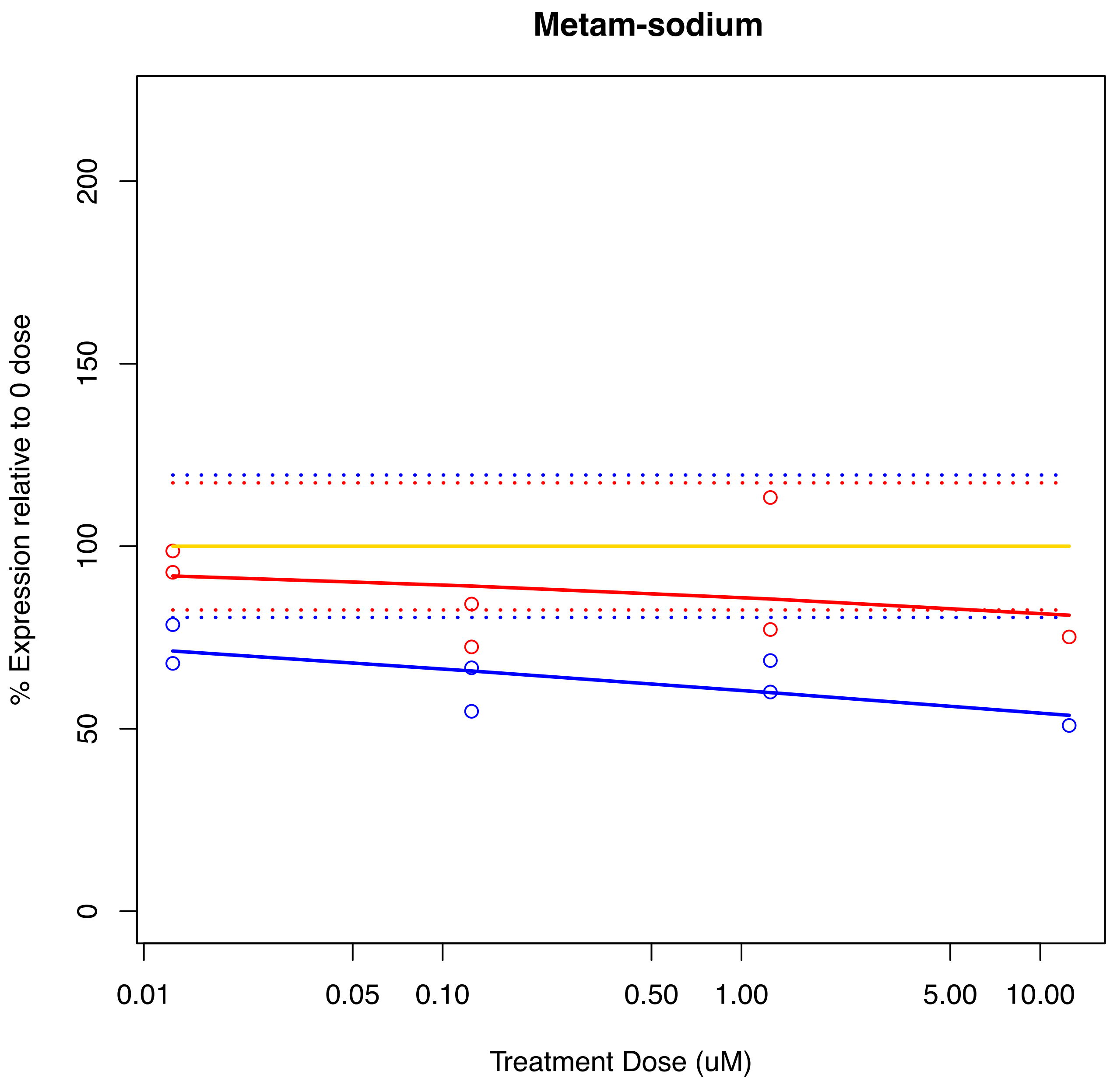
Metam-sodium
% Expression relative to 0 dose
0
50
100
150
200
0.01
0.05
0.10
0.50
1.00
5.00
10.00
Treatment Dose (uM)

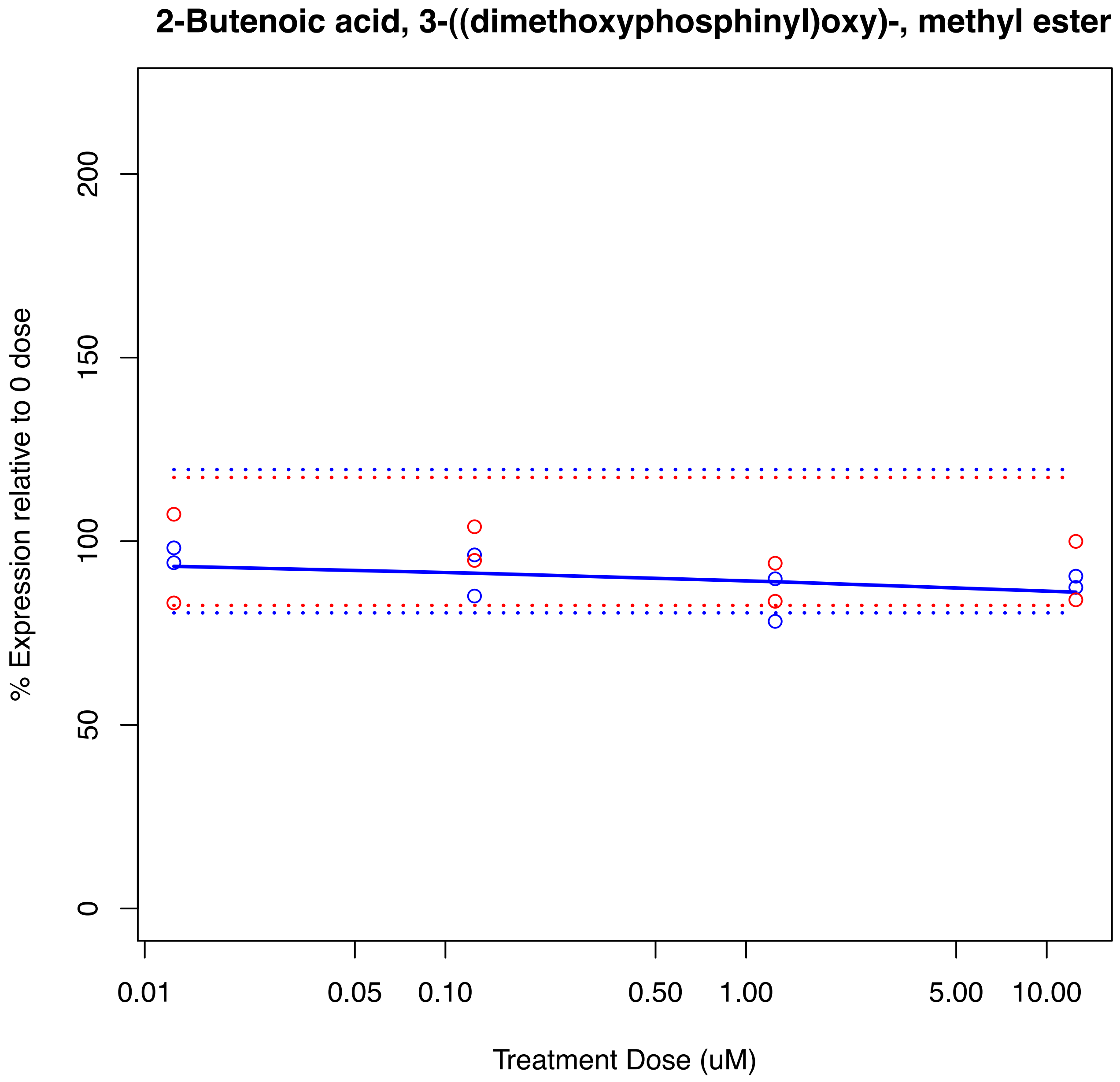
2-Butenoic acid, 3-((dimethoxyphosphinyl)oxy)-, methyl ester
% Expression relative to 0 dose
0
50
100
150
200
0.01
0.05
0.10
0.50
1.00
5.00
10.00
Treatment Dose (uM)

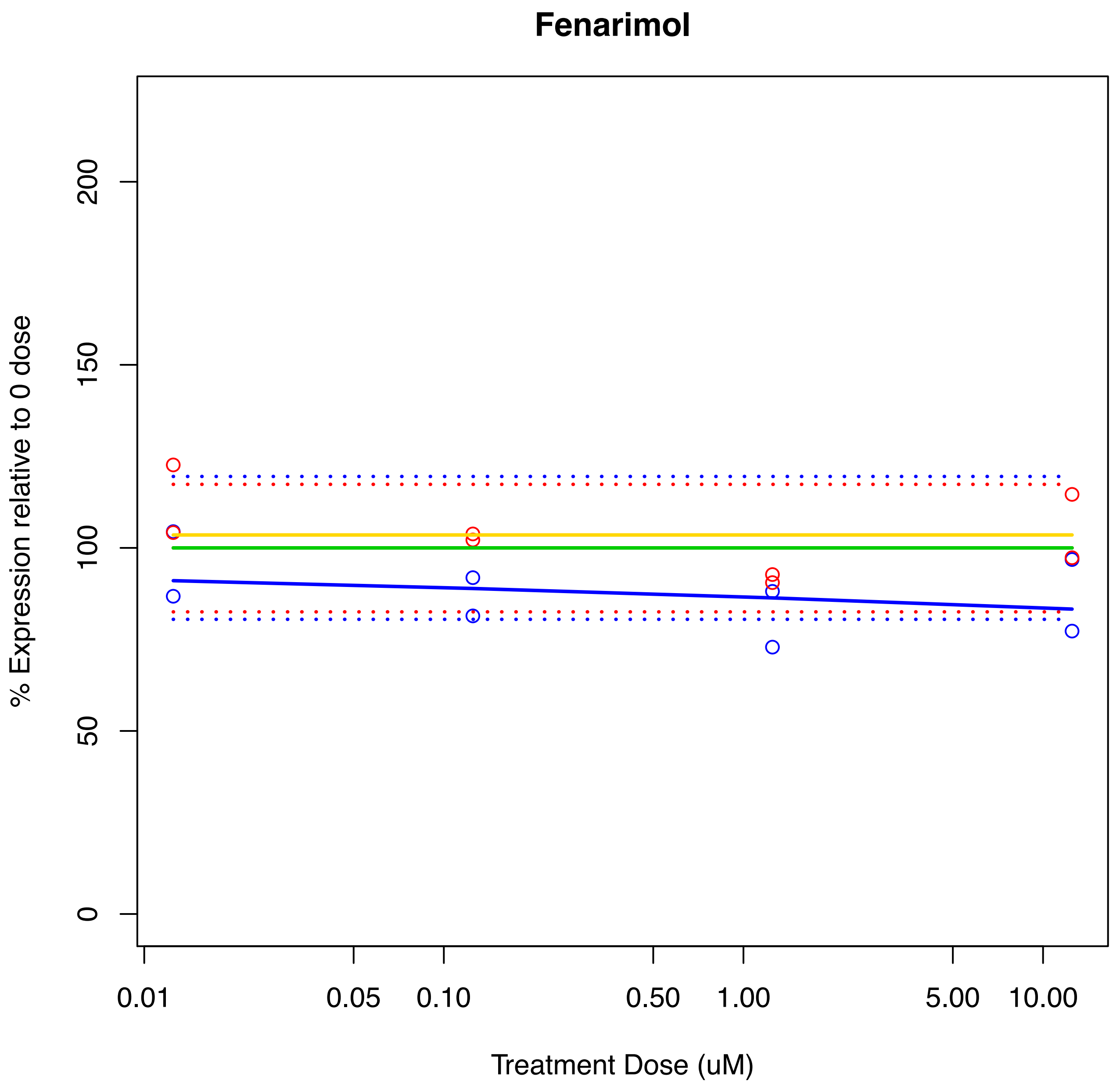

Fenarimol
% Expression relative to 0 dose
0
50
100
150
200
0.01
0.05
0.10
0.50
1.00
5.00
10.00
Treatment Dose (uM)

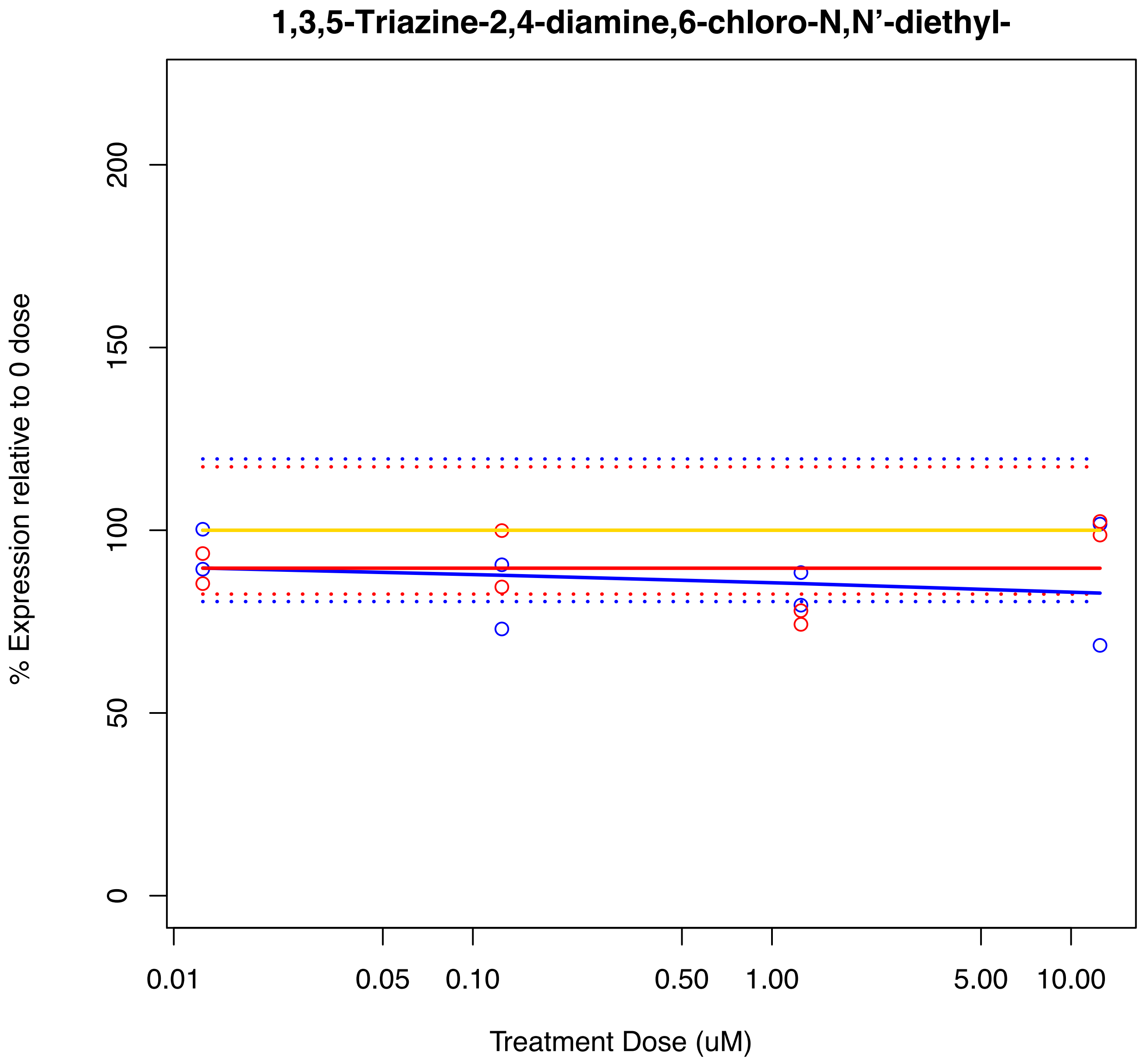

1,3,5-Triazine-2,4-diamine,6-chloro-N,N'-diethyl-
% Expression relative to 0 dose
0
50
100
150
200
0.01
0.05
0.10
0.50
1.00
5.00
10.00
Treatment Dose (uM)

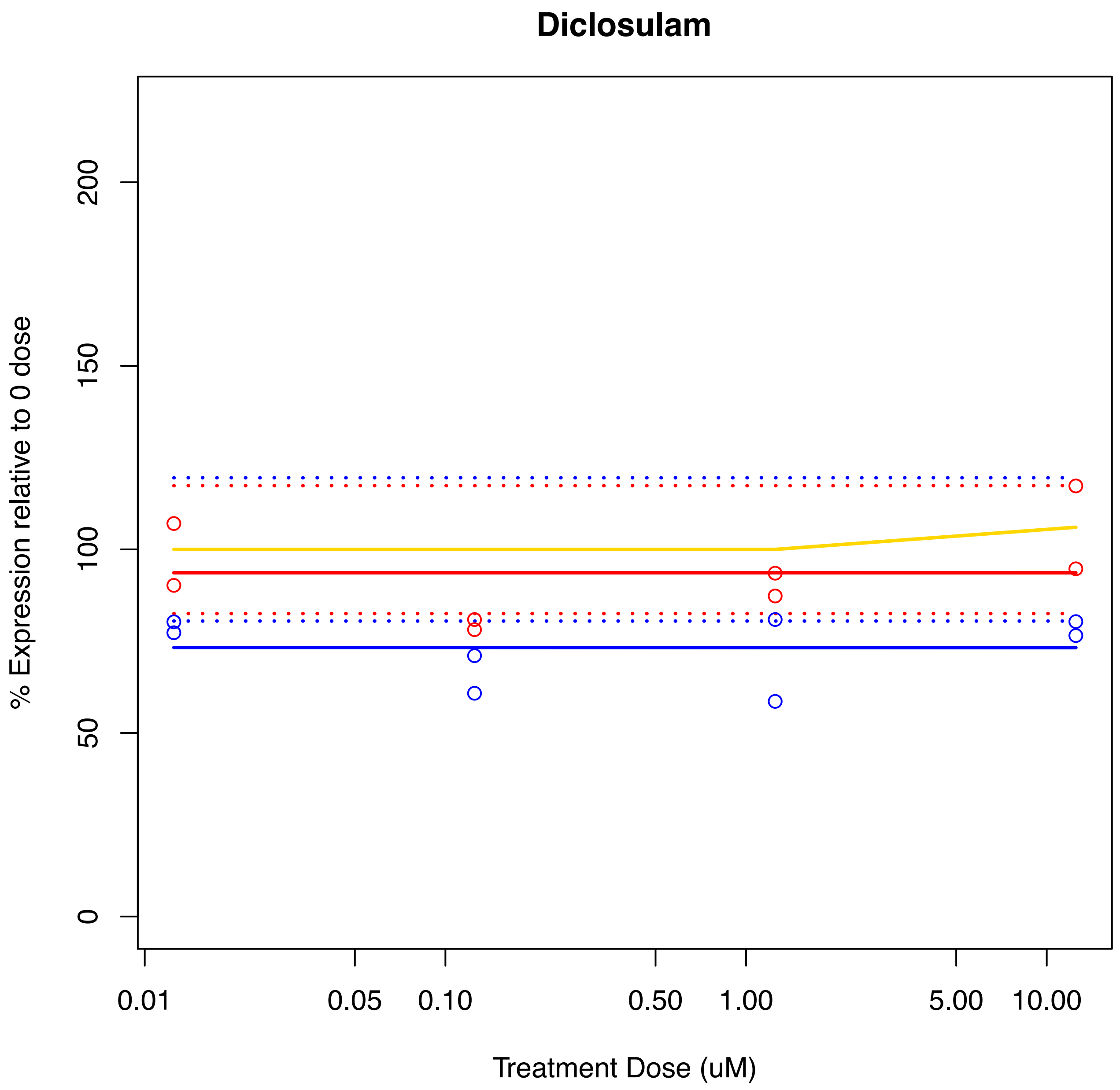

Diclosulam
% Expression relative to 0 dose
0
50
100
150
200
0.01
0.05
0.10
0.50
1.00
5.00
10.00
Treatment Dose (uM)

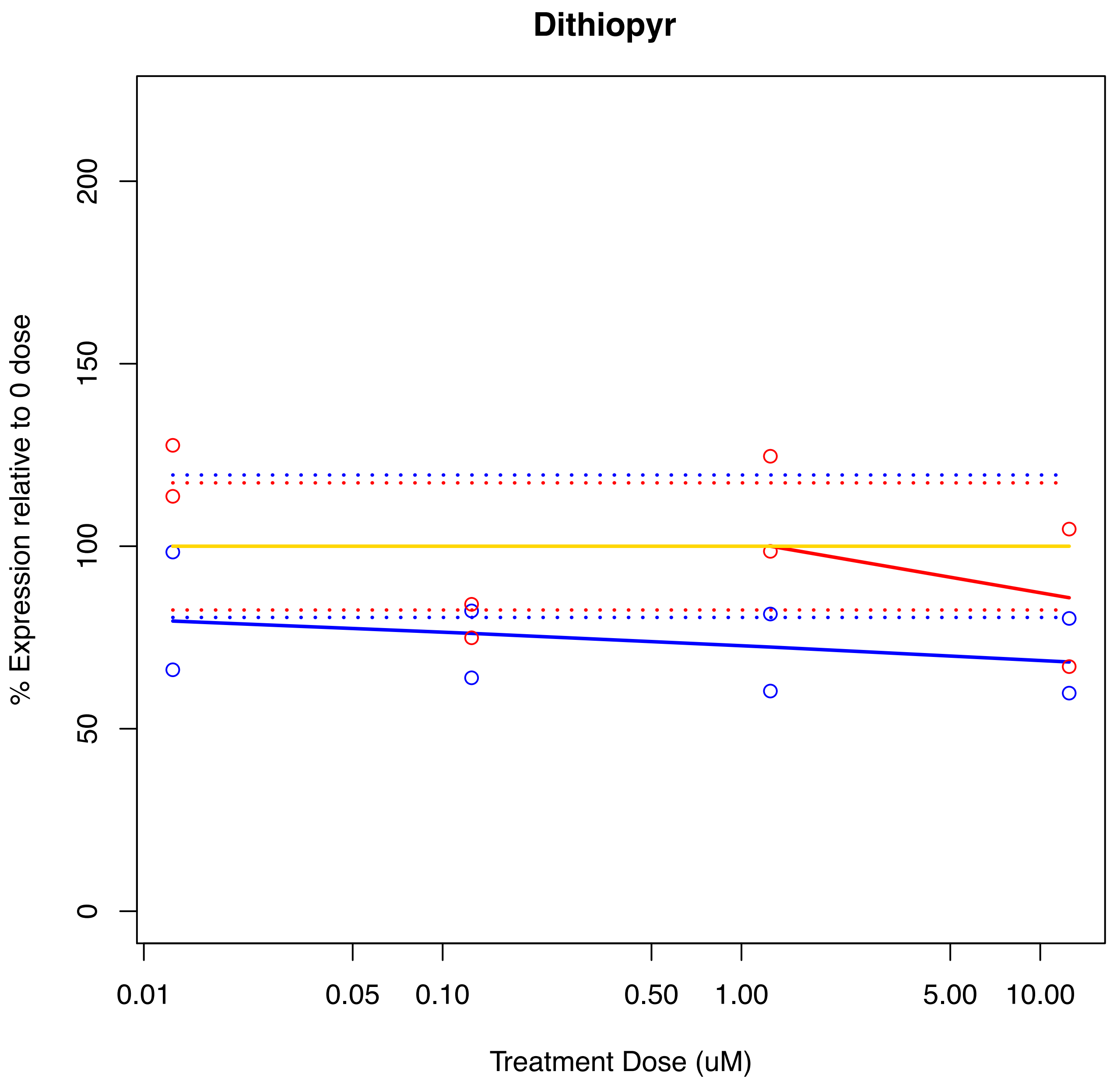

Dithiopyr
% Expression relative to 0 dose
0
50
100
150
200
0.01
0.05
0.10
0.50
1.00
5.00
10.00
Treatment Dose (uM)

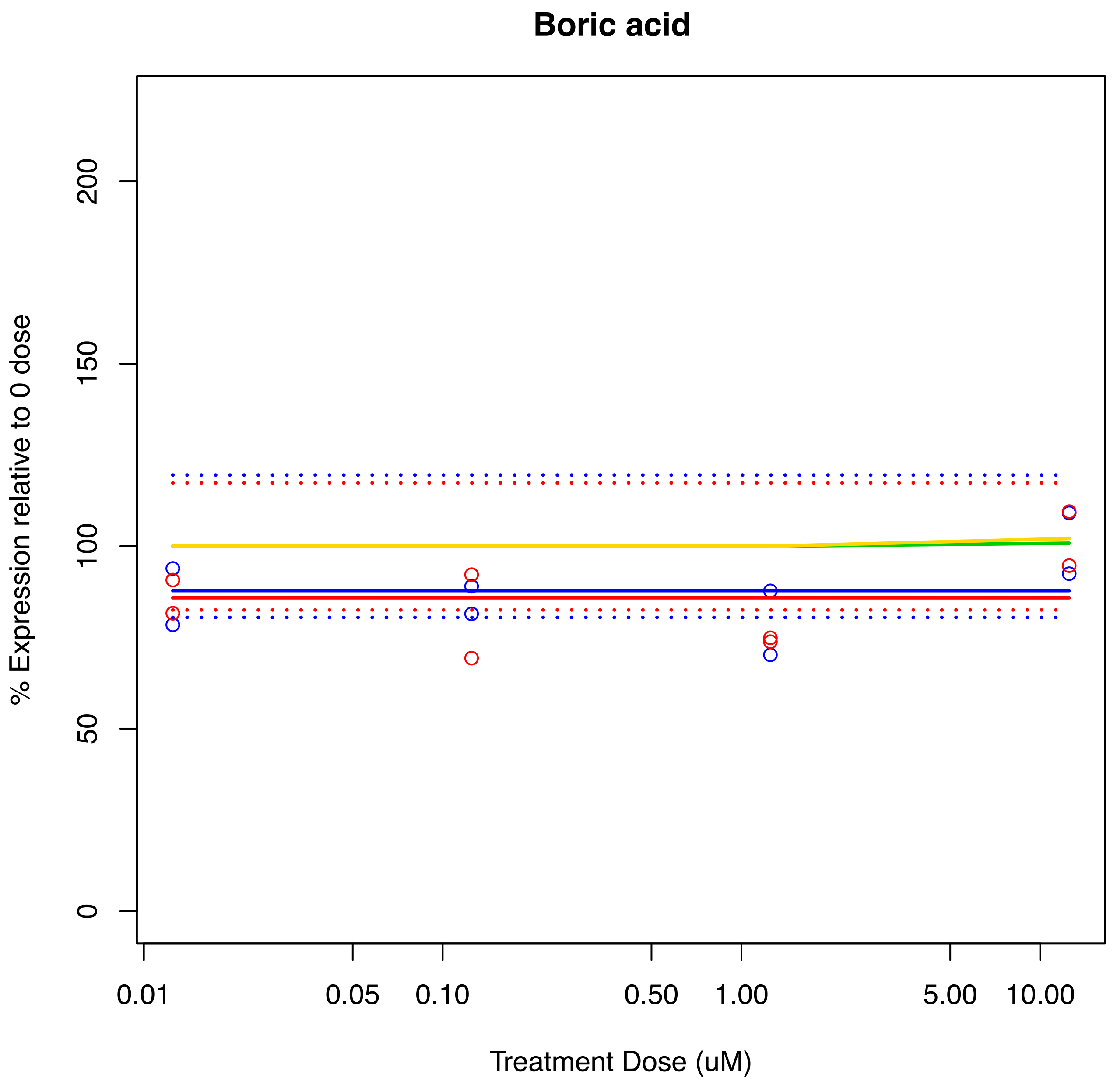
Boric acid
% Expression relative to 0 dose
0
50
100
150
200
0.01
0.05
0.10
0.50
1.00
5.00
10.00
Treatment Dose (uM)

Picloram

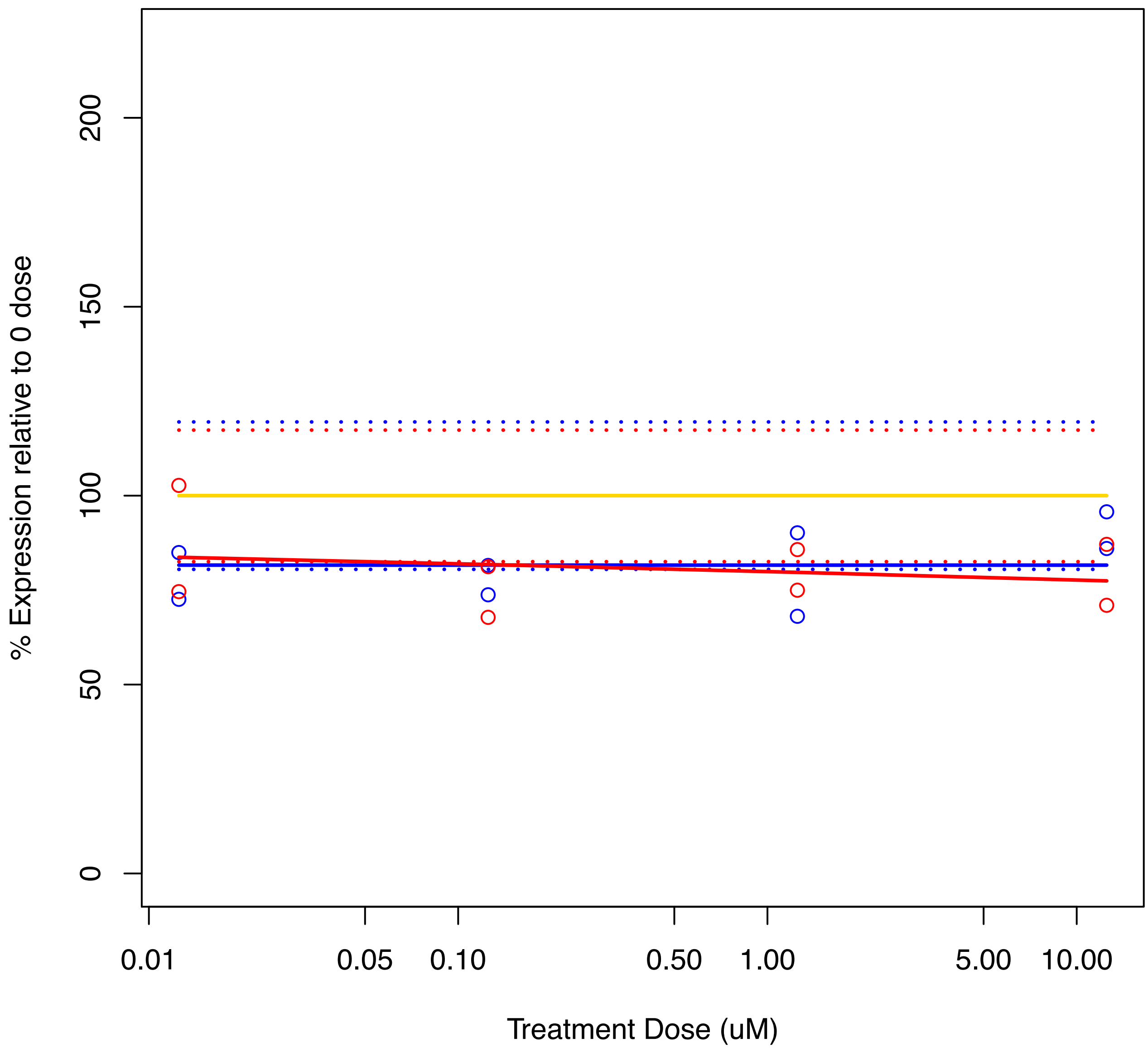

% Expression relative to 0 dose
0
50
100
150
200
0.01
0.05
0.10
0.50
1.00
5.00
10.00
Treatment Dose (uM)

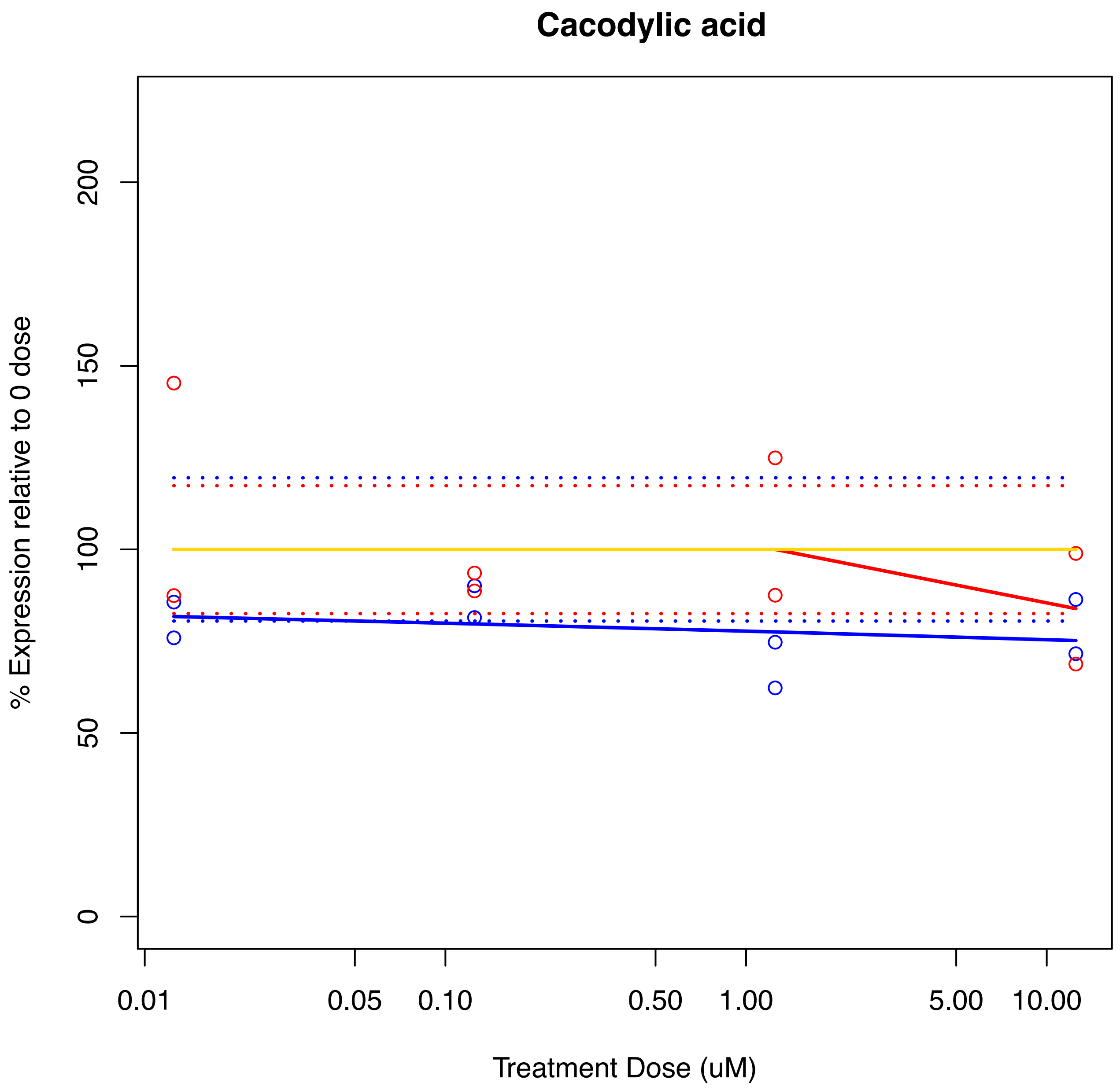

Cacodylic acid
% Expression relative to 0 dose
0
50
100
150
200
0.01
0.05
0.10
0.50
1.00
5.00
10.00
Treatment Dose (uM)

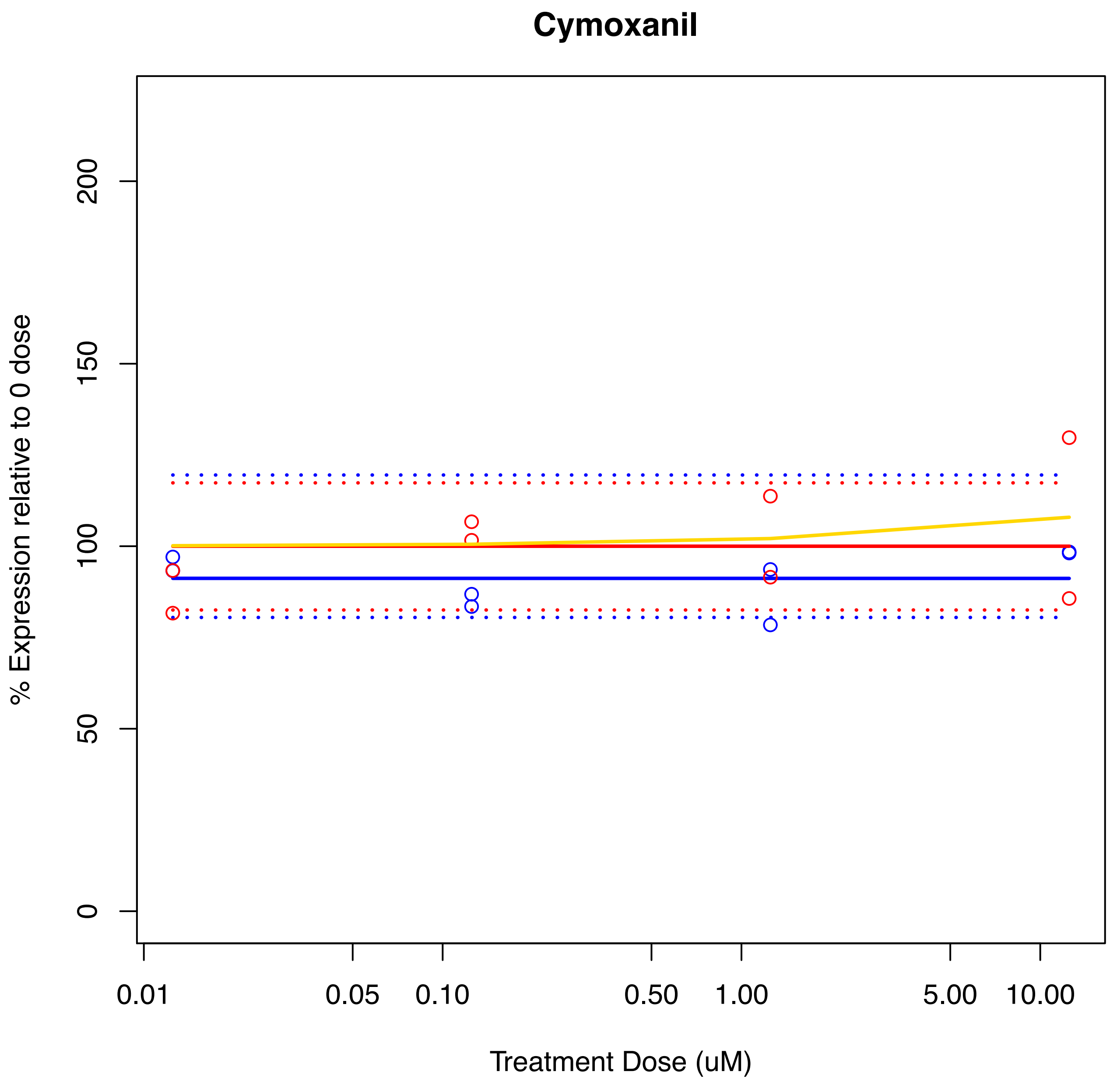
Cymoxanil
% Expression relative to 0 dose
0
50
100
150
200
0.01
0.05
0.10
0.50
1.00
5.00
10.00
Treatment Dose (uM)

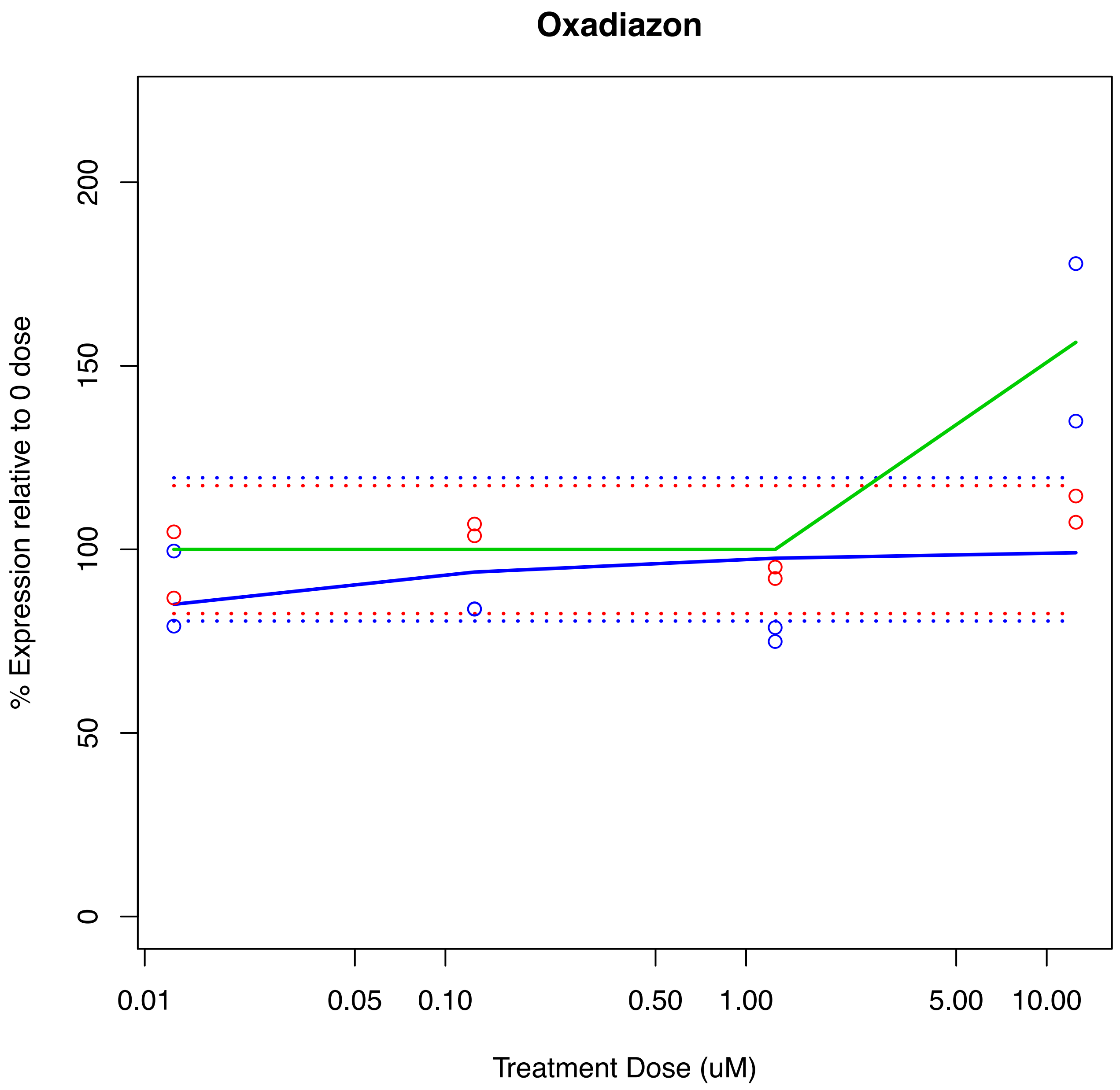
Oxadiazon
% Expression relative to 0 dose
Treatment Dose (uM)
0
50
100
150
200
0.01
0.05
0.10
0.50
1.00
5.00
10.00

Cyclohexanecarboxylic acid, 3,5-dioxo-4-(1-oxopropyl)-, ion(1-),calcium,calcium salt

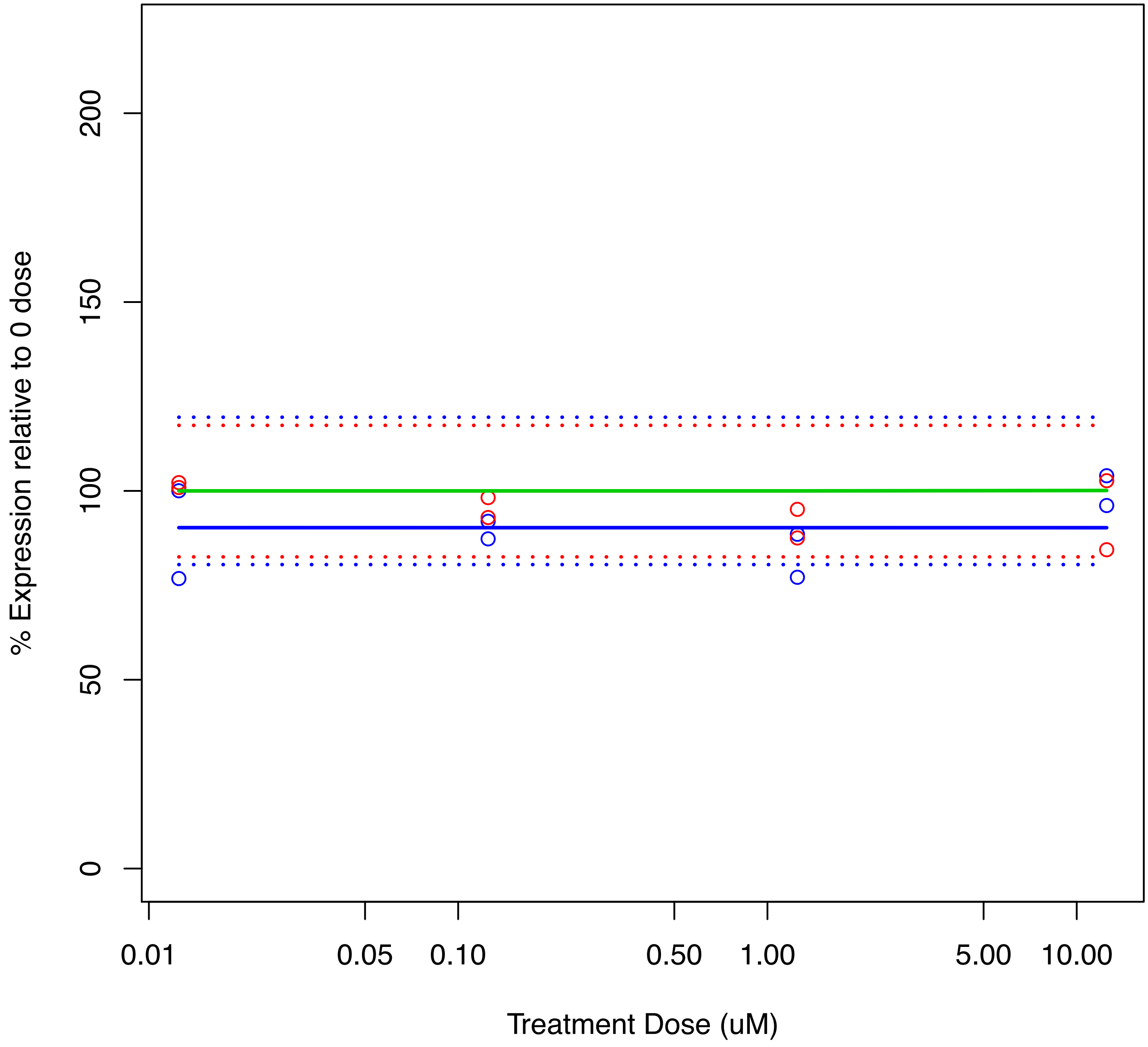

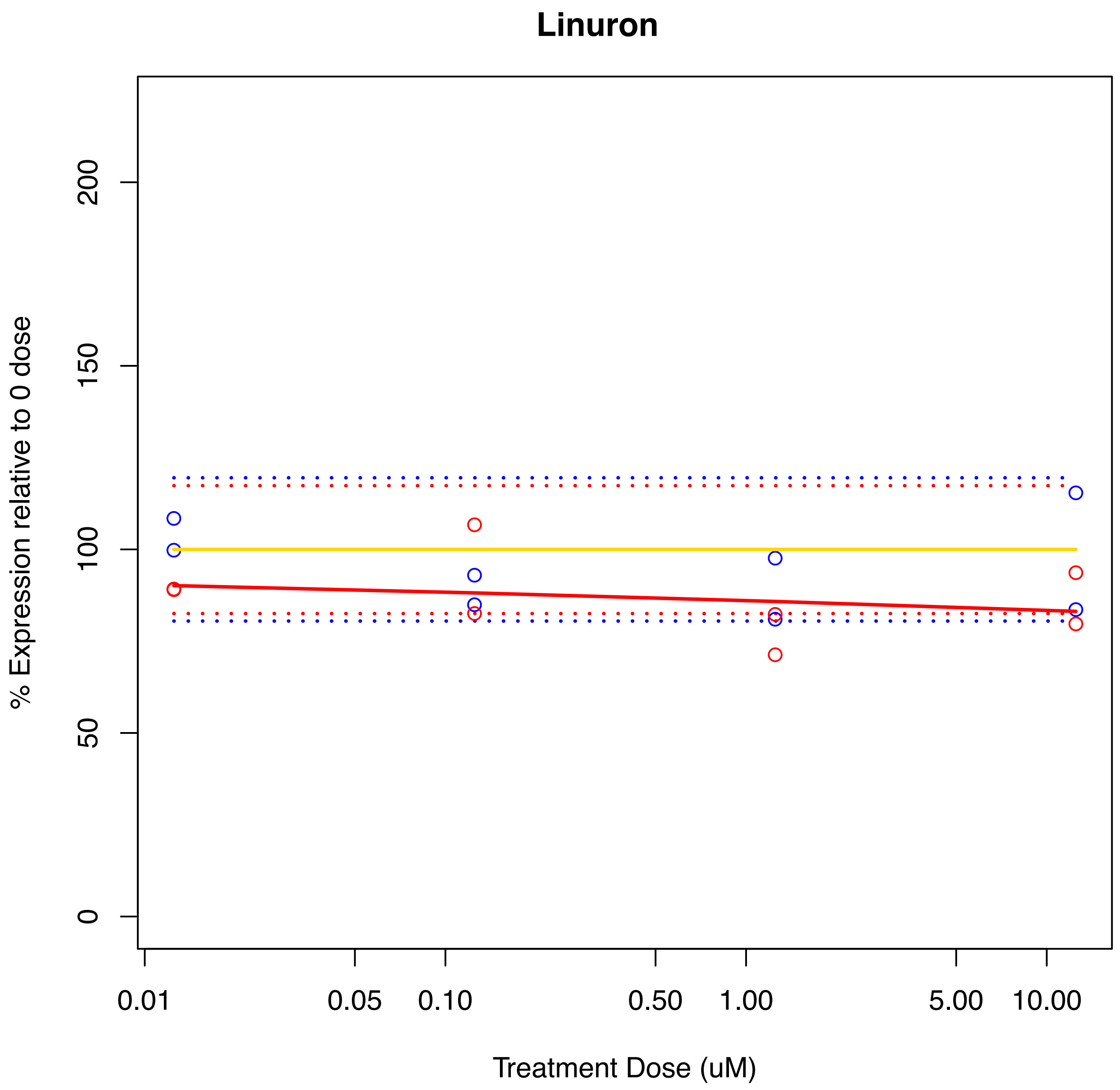
Linuron
% Expression relative to 0 dose
0
50
100
150
200
0.01
0.05
0.10
0.50
1.00
5.00
10.00
Treatment Dose (uM)

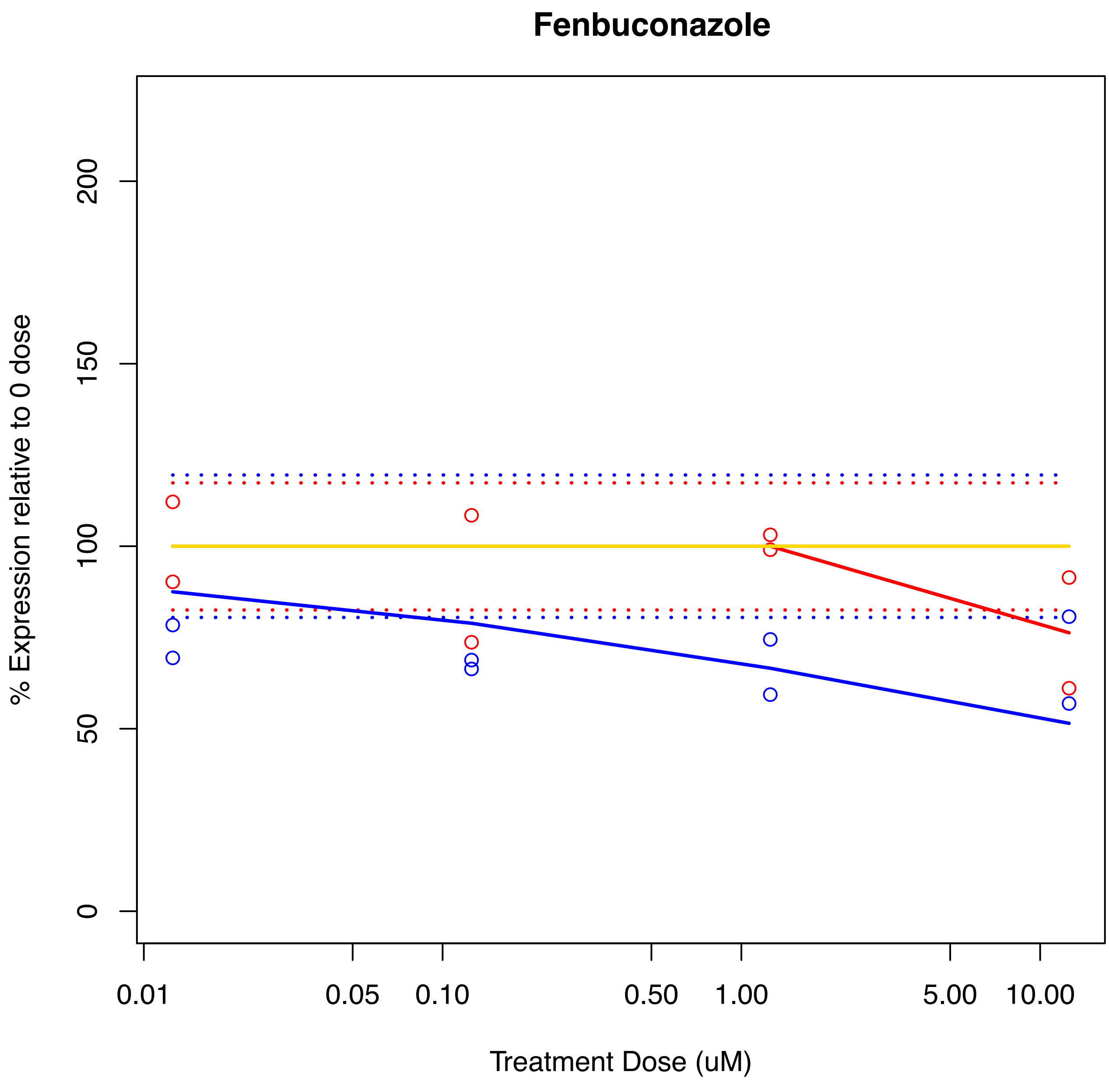

Fenbuconazole
% Expression relative to 0 dose
0
50
100
150
200
0.01
0.05
0.10
0.50
1.00
5.00
10.00
Treatment Dose (uM)

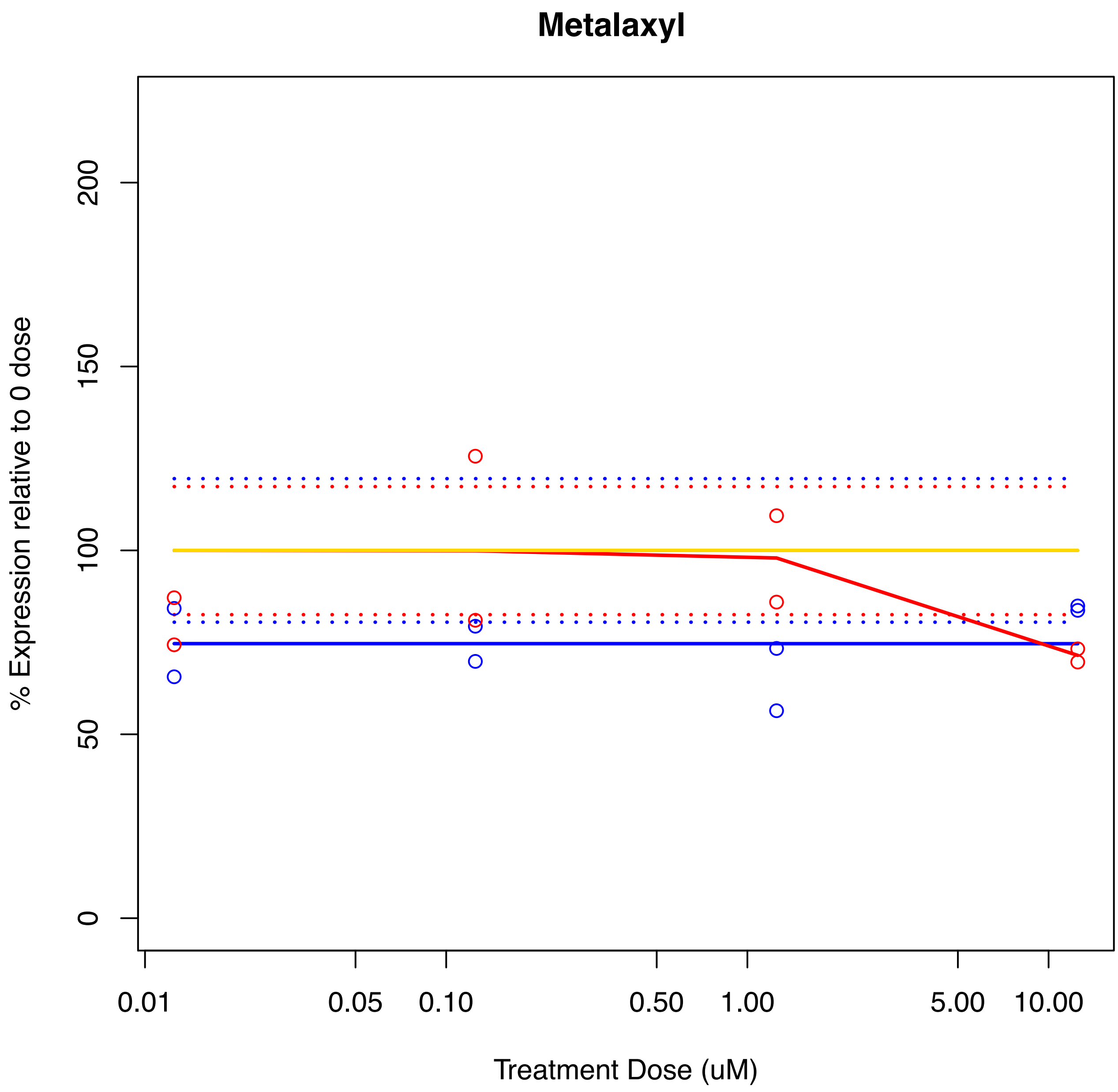
Metalaxyl
% Expression relative to 0 dose
0
50
100
150
200
0.01
0.05
0.10
0.50
1.00
5.00
10.00
Treatment Dose (uM)

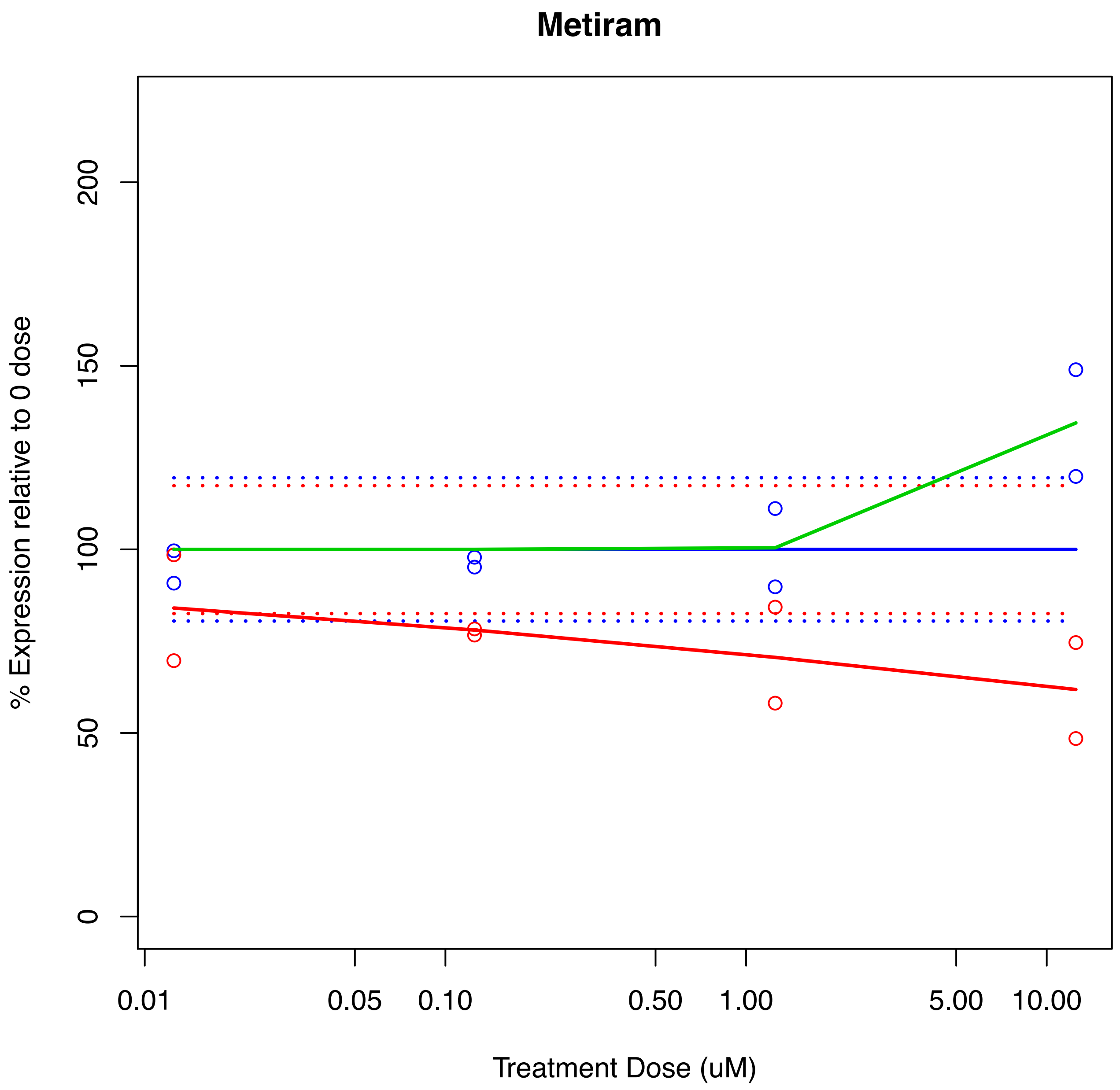
Metiram
% Expression relative to 0 dose
0
50
100
150
200
0.01
0.05
0.10
0.50
1.00
5.00
10.00
Treatment Dose (uM)

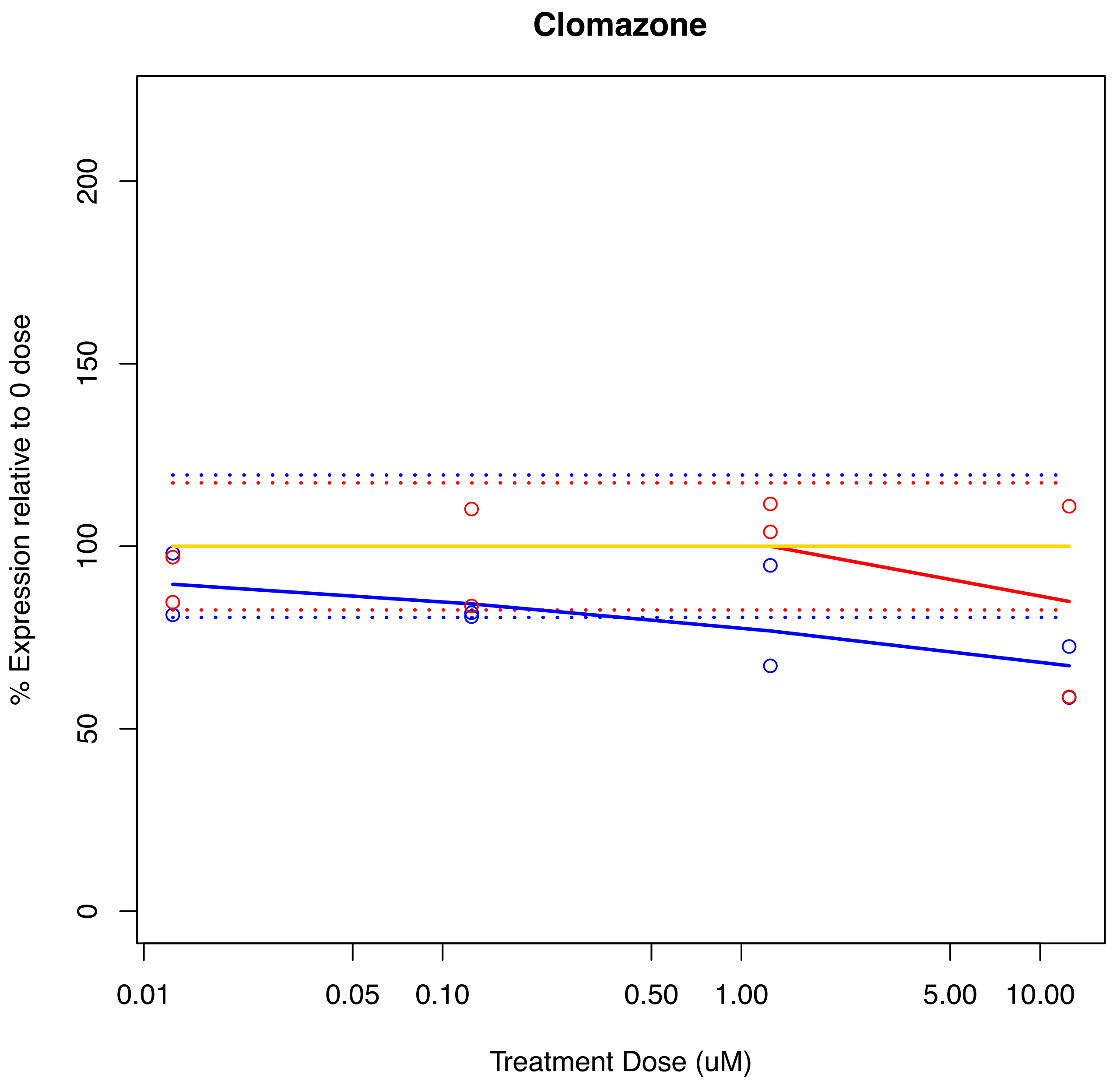
Clomazone
% Expression relative to 0 dose
0
50
100
150
200
0.01
0.05
0.10
0.50
1.00
5.00
10.00
Treatment Dose (uM)

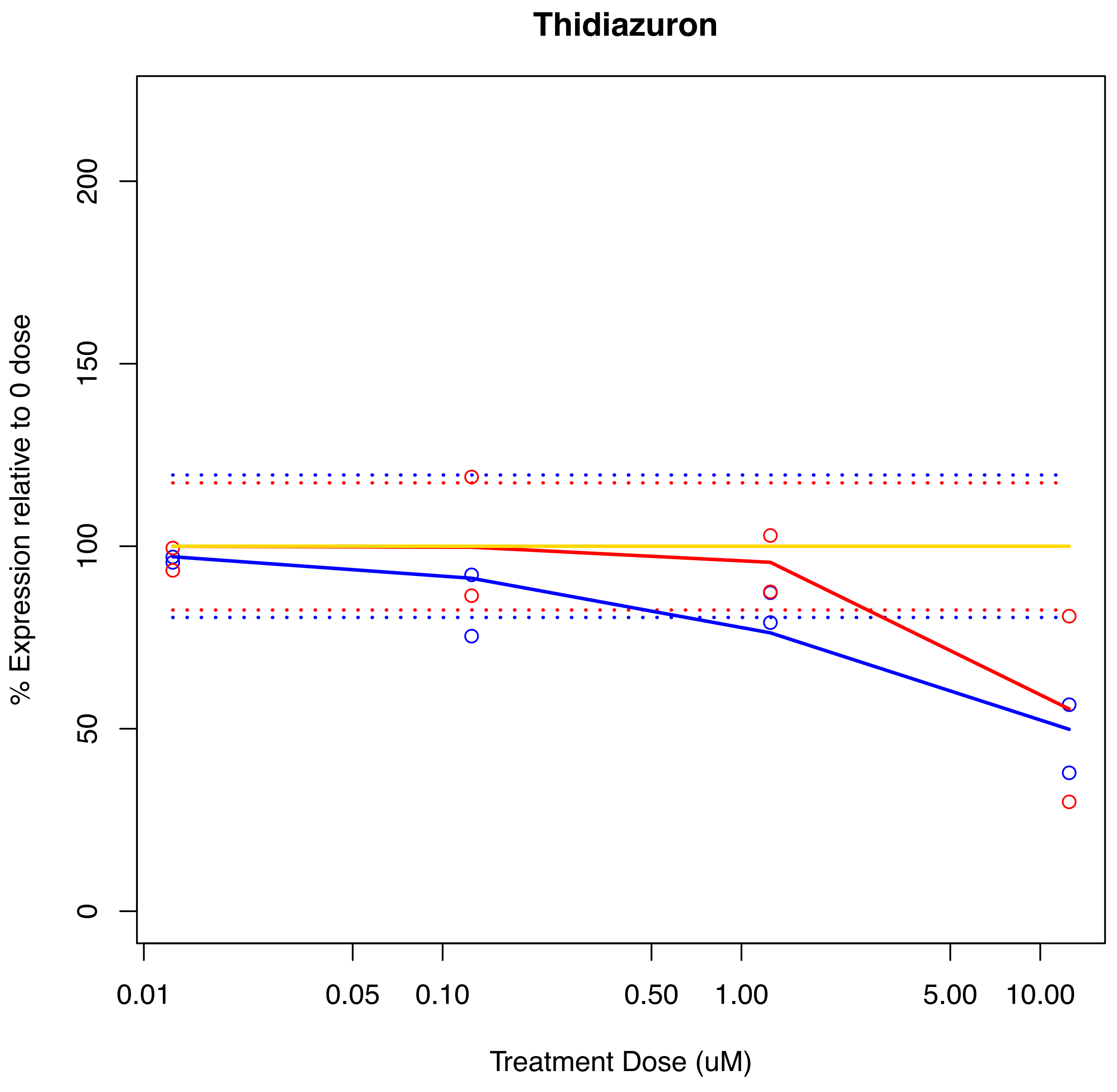

Thidiazuron
% Expression relative to 0 dose
0
50
100
150
200
0.01
0.05
0.10
0.50
1.00
5.00
10.00
Treatment Dose (uM)

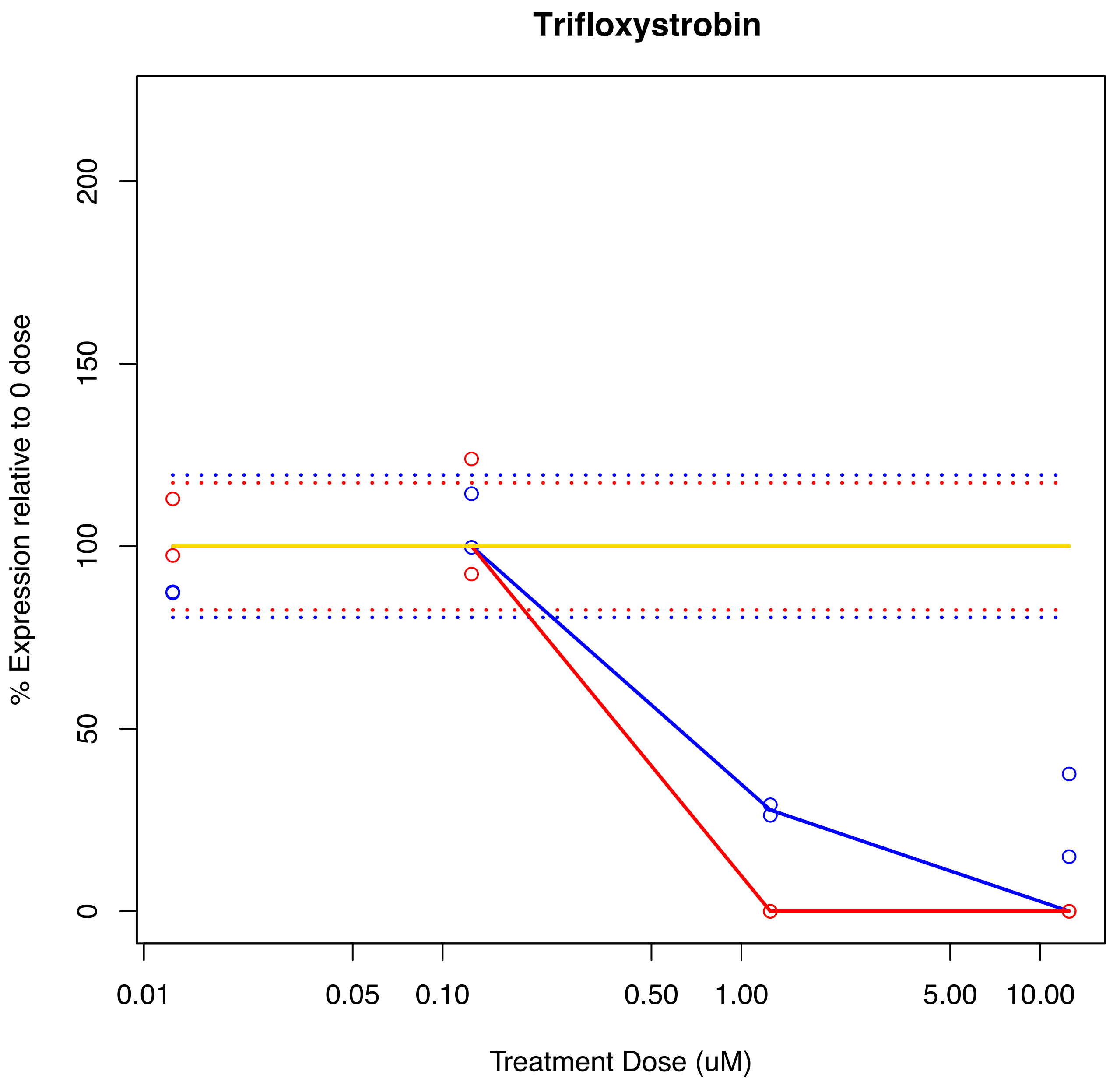
Trifloxystrobin
% Expression relative to 0 dose
0
50
100
150
200
0.01
0.05
0.10
0.50
1.00
5.00
10.00
Treatment Dose (uM)

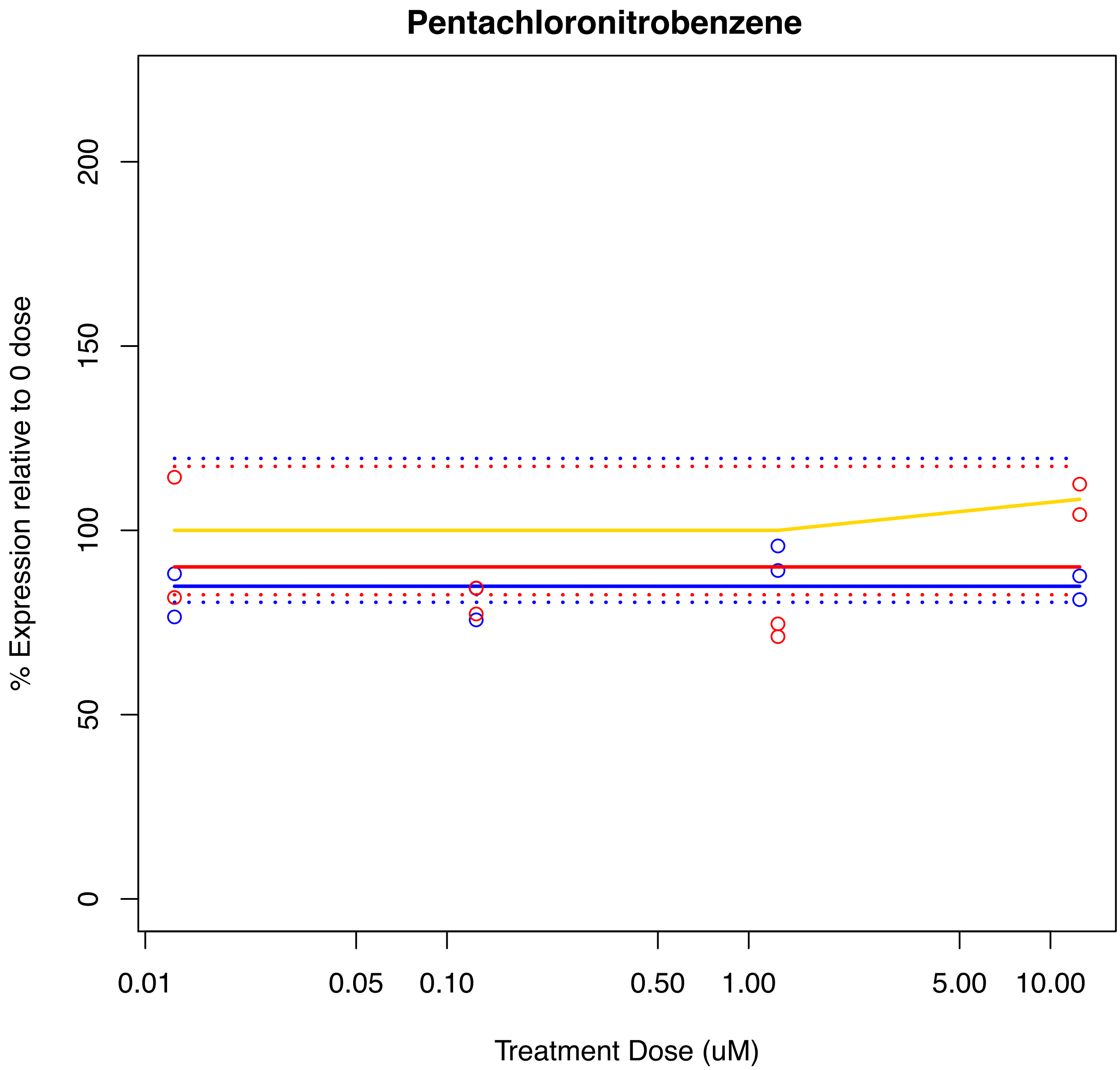
Pentachloronitrobenzene
% Expression relative to 0 dose
0
50
100
150
200
0.01
0.05
0.10
0.50
1.00
5.00
10.00
Treatment Dose (uM)

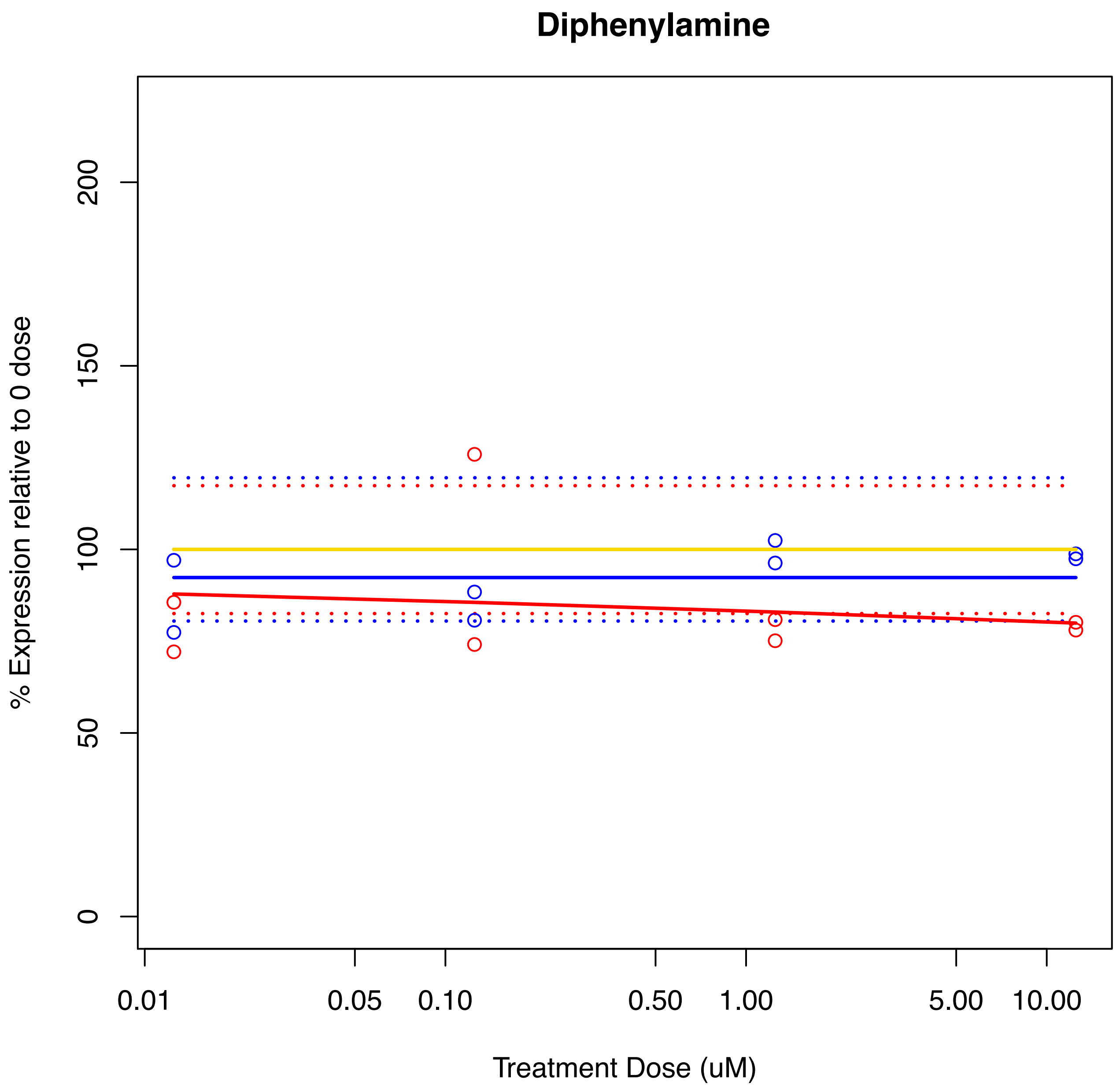

Diphenylamine
% Expression relative to 0 dose
0
50
100
150
200
0.01
0.05
0.10
0.50
1.00
5.00
10.00
Treatment Dose (uM)

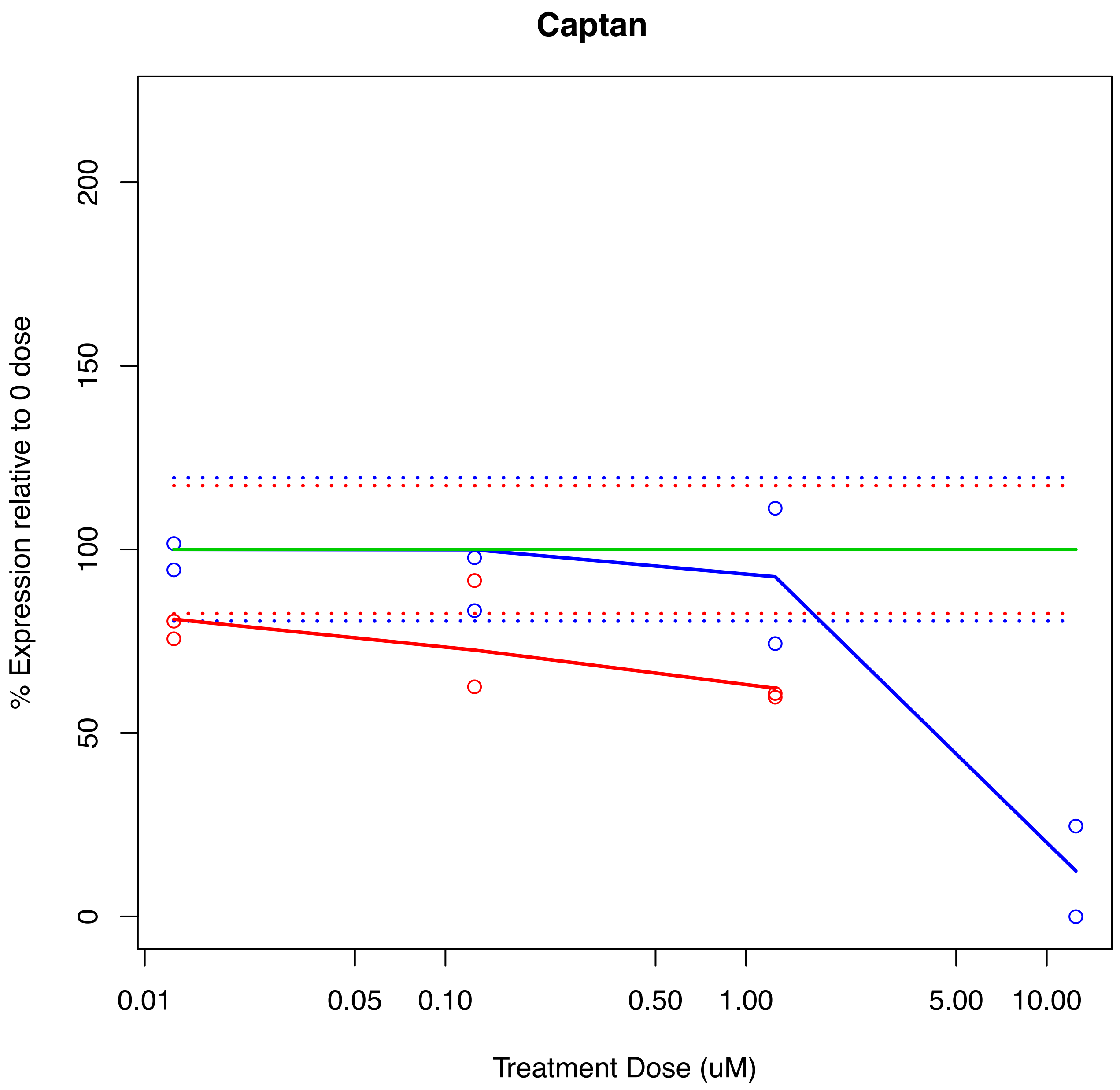
Captan
% Expression relative to 0 dose
0
50
100
150
200
0.01
0.05
0.10
0.50
1.00
5.00
10.00
Treatment Dose (uM)

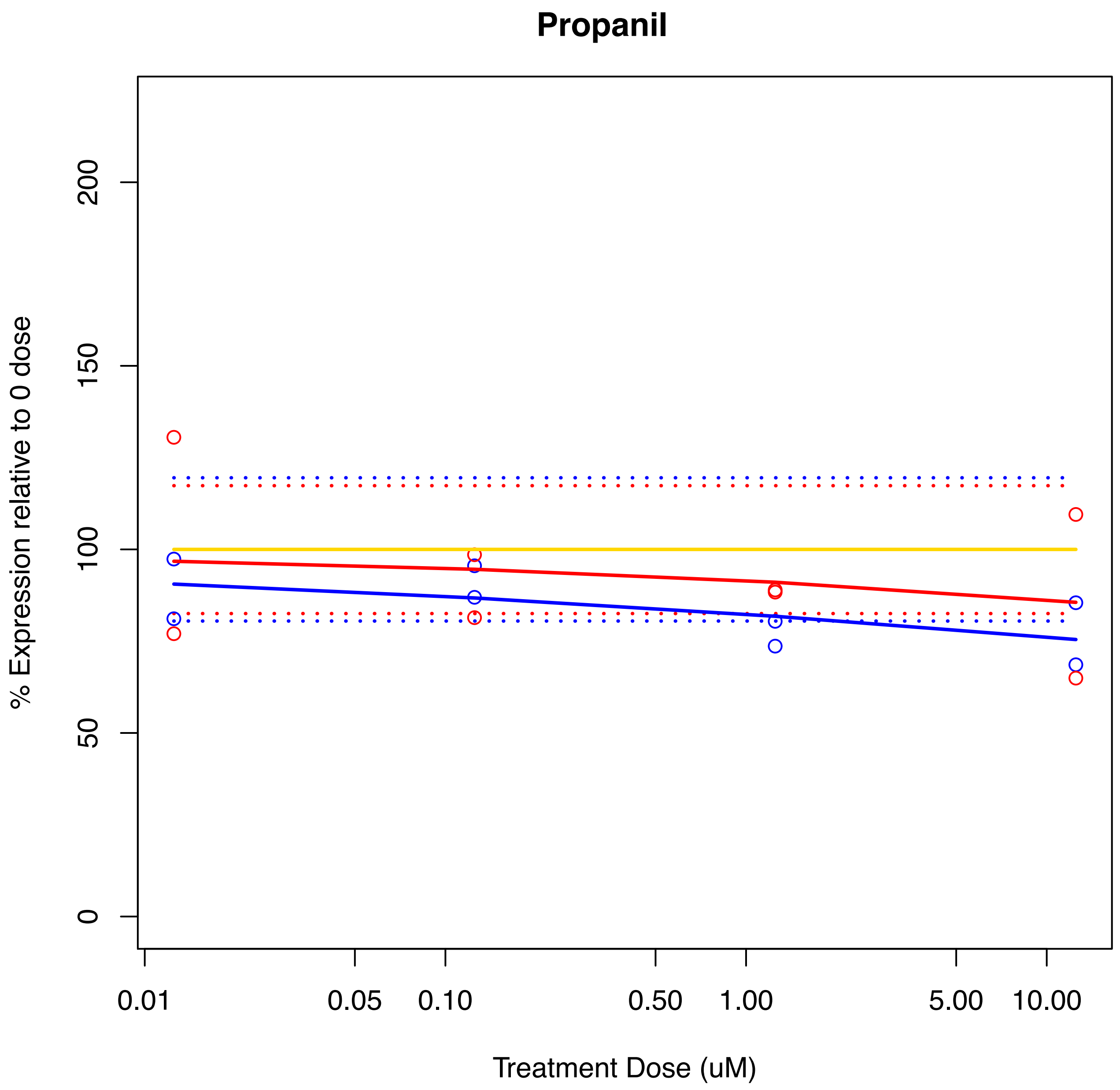
Propanil
% Expression relative to 0 dose
0
50
100
150
200
0.01
0.05
0.10
0.50
1.00
5.00
10.00
Treatment Dose (uM)

o-Phenylphenol

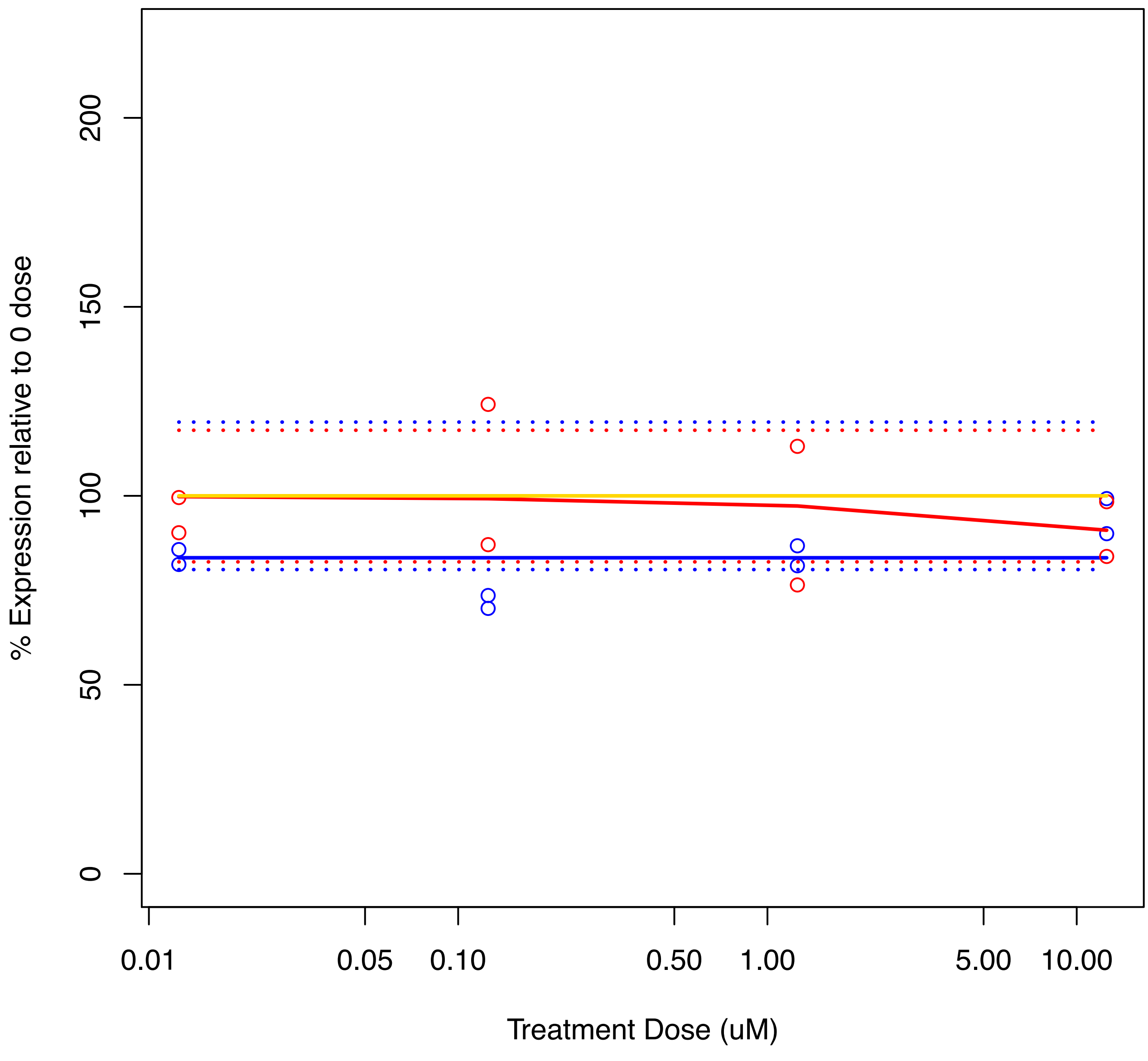

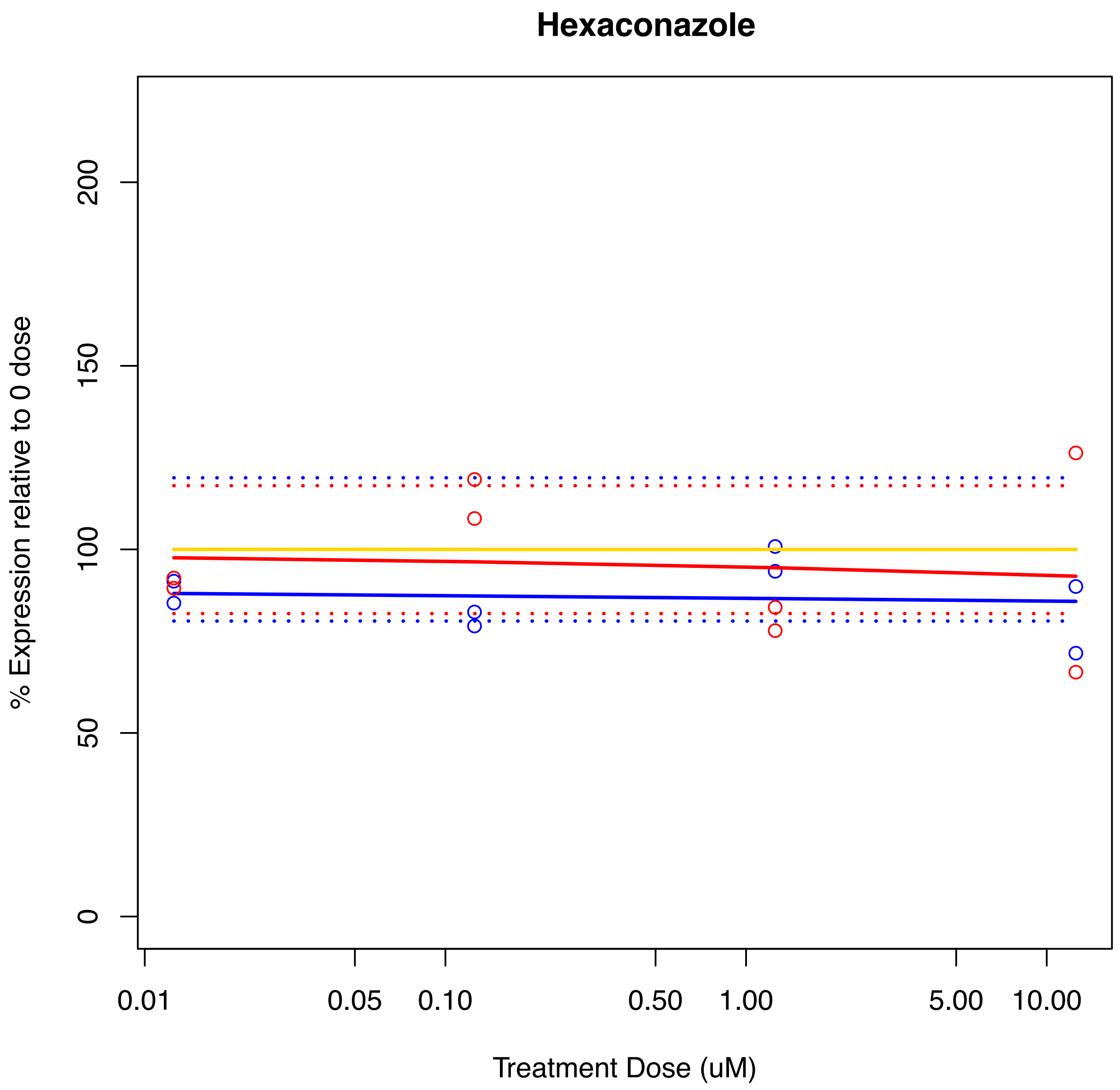
Hexaconazole
% Expression relative to 0 dose
0
50
100
150
200
0.01
0.05
0.10
0.50
1.00
5.00
10.00
Treatment Dose (uM)

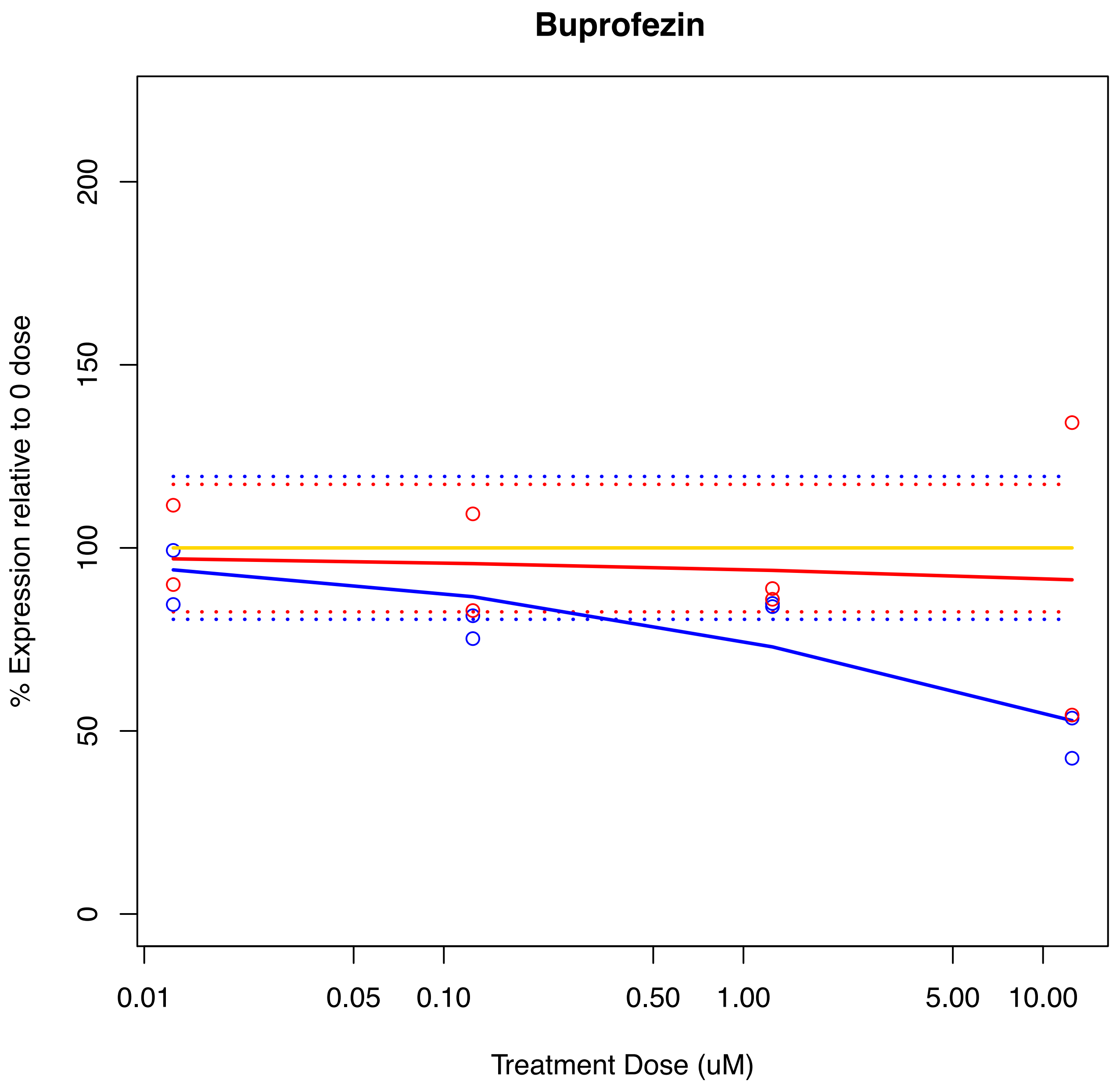
Buprofezin
% Expression relative to 0 dose
0
50
100
150
200
0.01
0.05
0.10
0.50
1.00
5.00
10.00
Treatment Dose (uM)
