# Supplementary figures and images for: Evaluation of 309 Environmental Chemicals Using a Mouse Embryonic Stem Cell Adherent Cell Differentiation and Cytotoxicity Assay
Source: PLoS One. 2011 Jun 7;6(6):e18540. doi: 10.1371/journal.pone.0018540 (PMC3110185; doi:10.1371/journal.pone.0018540)

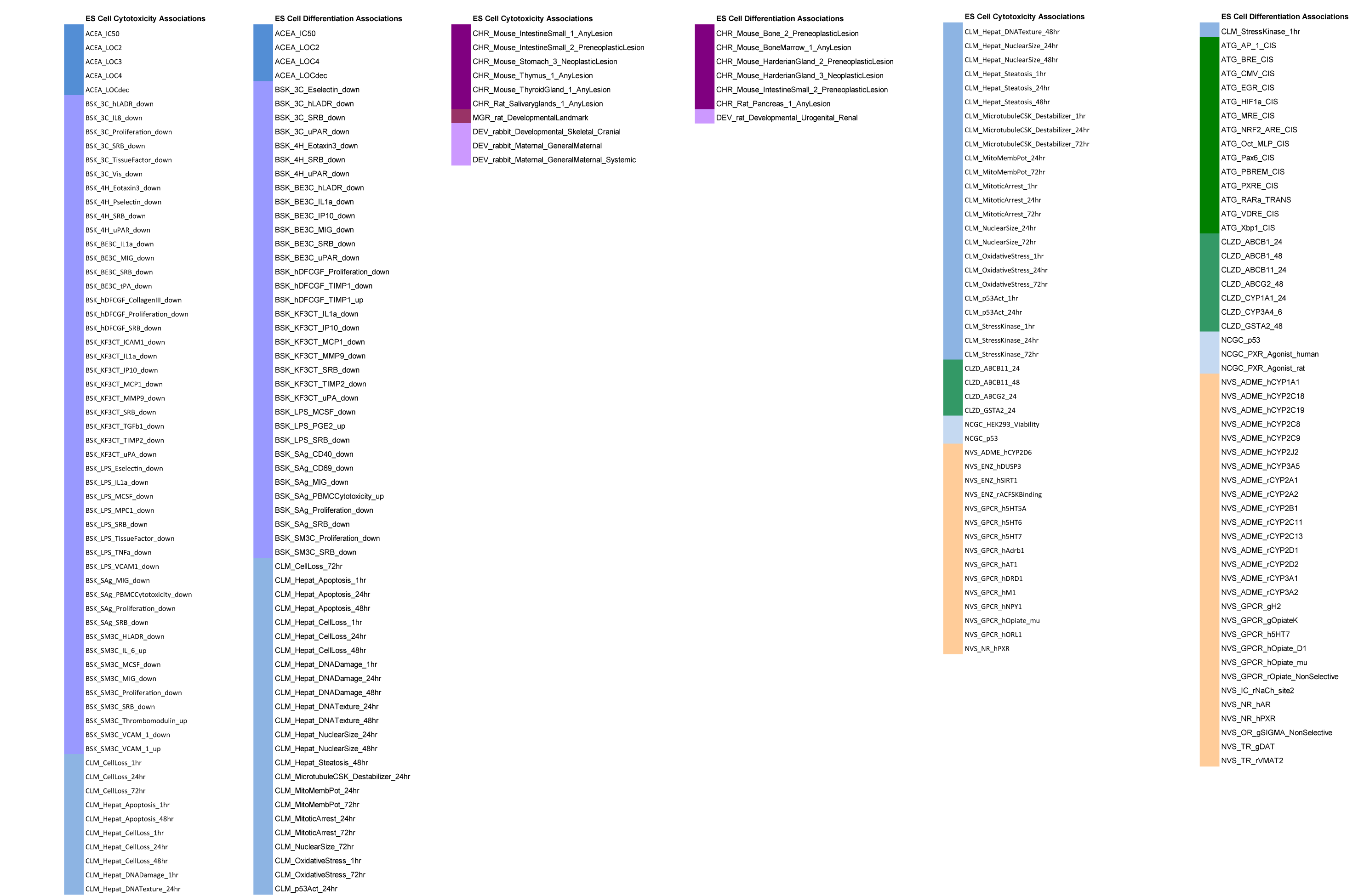

Supplement: Figure S2 — Statistical associations between ES cell endpoints and ToxCast or ToxRefDB targets. (TIF) [file pone.0018540.s002.tif]
